# Organocatalytic asymmetric electrochemical synthesis of epibromohydrins from allylic alcohols

**DOI:** 10.1039/d6sc05589c

**Published:** 2026-08-31

**Authors:** Zhengyu Han, Ming Fang, Peinan Shang, Shuxuan Liu, Hai Huang, Jianwei Sun

**Affiliations:** a Jiangsu Key Laboratory of Advanced Catalytic Materials & Technology, School of Petrochemical Engineering, Changzhou University Changzhou 213164 China huanghai@cczu.edu.cn hanzhengyu@cczu.edu.cn; b Department of Chemistry, The Hong Kong University of Science and Technology Clear Water Bay Kowloon Hong Kong SAR 999077 China sunjw@ust.hk

## Abstract

Optically active epibromohydrins represent a structurally privileged family of chiral building blocks, yet their asymmetric synthesis remains hampered by a reliance on stoichiometric chemical oxidants and multi-step protocols. Herein, we report an organocatalytic asymmetric electrochemical bromoetherification of allylic tertiary alcohols that constructs these densely functionalized scaffolds in a single operational step. By merging interfacial electrosynthesis with a synergistic chiral phase-transfer catalyst (PTC) and chiral phosphoric acid (CPA) counter anion framework, the high reactivity of electrogenerated Br_2_ species is tightly regulated, successfully suppressing unselective, racemic background pathways. Utilizing inexpensive NaBr as the bromine source in a DCM/H_2_O biphasic system, a range of allylic tertiary alcohols were smoothly converted into the corresponding bromoepoxides in high yields and with high enantioselectivities. Control experiments and cyclic voltammetry studies suggested that stereocontrol is sensitive to the balance between anodic bromide generation and phase-transfer-mediated trapping. This strategy is complementary to the conventional synthesis of epibromohydrins by chemical oxidation.

## Abstract 2

An efficient and enantioselective electrochemical bromoetherification of tertiary allylic alcohols has been developed. It provides access to enantioenriched epibromohydrins from readily available materials in a single operational step.

## Graphical abstract

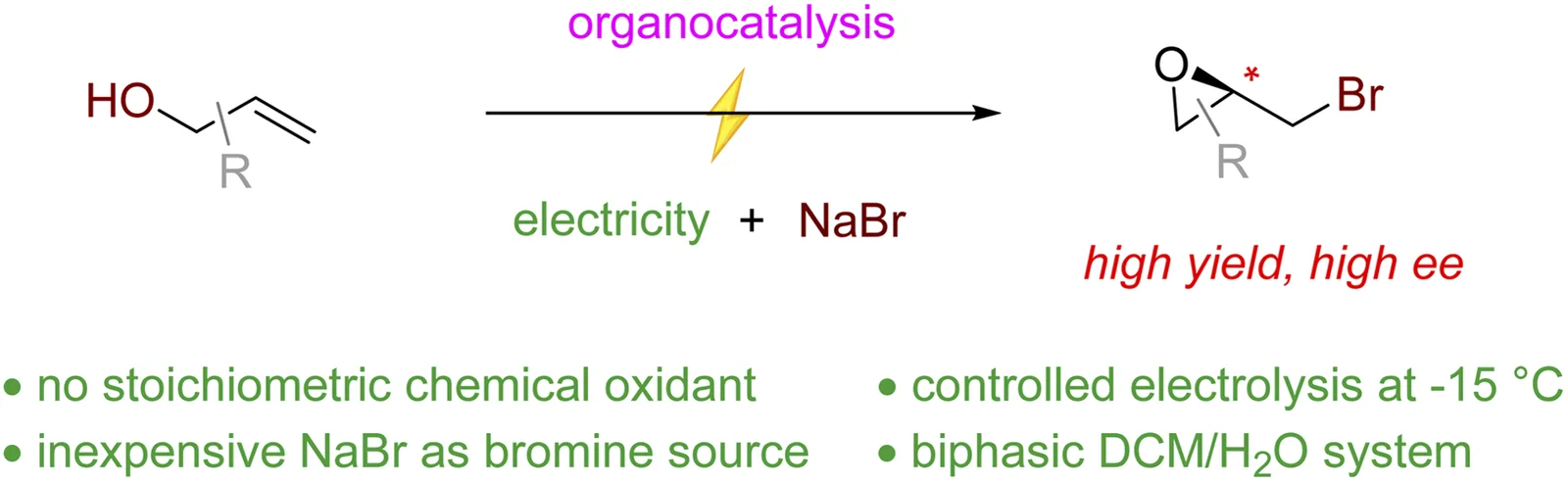

## Introduction

Optically active epihalohydrins belong to a versatile and structurally privileged family of chiral building blocks in modern organic synthesis.^1^ Characterized by the unique juxtaposition of a highly strained, reactive oxirane ring and an excellent halogen leaving group (particularly bromide), these densely functionalized scaffolds serve as indispensable starting materials in both academic and industrial settings.^1,2^ They are extensively utilized in the asymmetric synthesis of blockbuster pharmaceuticals, such as β-adrenergic receptor antagonists (*e.g.*, *S*-propranolol)^2*a*^ and antiviral therapeutics, as well as an array of complex bioactive natural products.^2^ Given their widespread utility, developing highly efficient, step-economical, and stereoselective strategies to access enantioenriched epihalohydrins remains a paramount objective in synthetic organic chemistry.

**Fig. 1:**
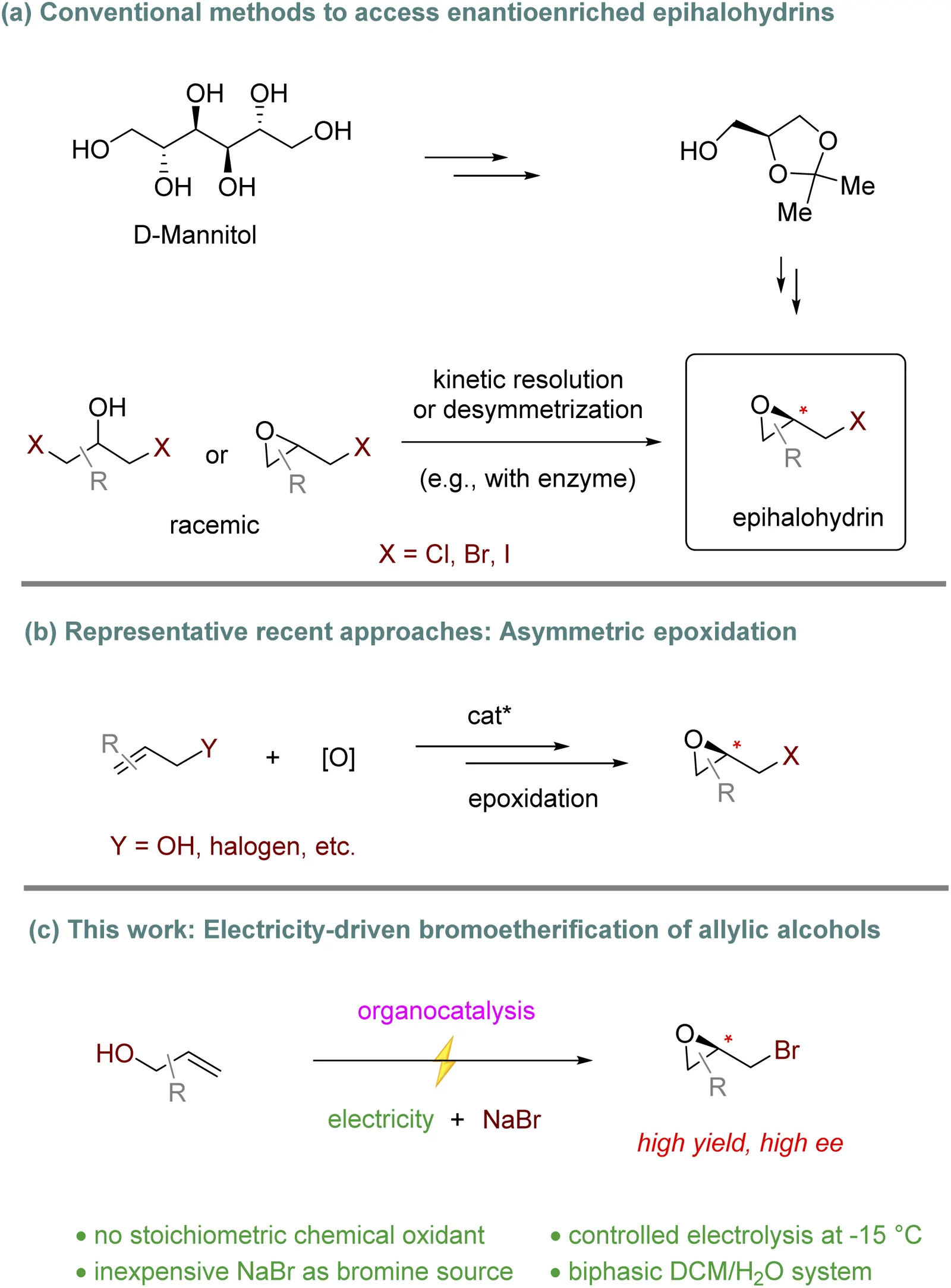
Conventional approaches and reaction design: (a) synthesis from chiral-pool precursors; (b) asymmetric epoxidation of allylic halides or alcohol derivatives; and (c) the present electrochemical bromoetherification of allylic alcohols.

Despite their broad applications, the current asymmetric synthesis of epihalohydrins still poses operational and environmental challenges.^1,4–7^ Traditional methods rely heavily on the multi-step derivatization of natural chiral-pool precursors (*e.g.*, D-mannitol),^3^ or on kinetic resolution and desymmetrizations of racemic halohydrins and epoxides using enzymatic catalysis (Fig. 1a).^4^ Recently, impressive progress has been achieved using small-molecule chiral catalysts.^5,6^ A notable approach is the asymmetric epoxidation of allylic halides (or allylic alcohols and their derivatives followed by halogenation, Fig. 1b), which serves as an attractive alternative to conventional strategies.^6^ However, despite representing milestones in asymmetric catalysis, these protocols inevitably mandate the use of stoichiometric chemical oxidants, raising concerns regarding atom economy, waste generation, and operational safety.^8^

Over the past decade, organic electrochemistry has emerged as a valuable approach in sustainable synthesis, offering an alternative strategy to bypass stoichiometric redox reagents.^9^ By employing the electron as a “traceless” and highly tunable redox agent, electrosynthesis enables precise manipulation of reaction trajectories under mild conditions. Building on these foundational advances, recent breakthroughs have successfully extended this platform to asymmetric catalysis, enabling precise control over enantioselectivity during electrochemical transformations.

In this context, we envisioned that the electrochemical bromoetherification of allylic alcohols would represent an exceptionally elegant and direct avenue to construct the epibromohydrin framework from readily available feedstocks in a single operational step (Fig. 1c). Mechanistically, this process involves the controlled anodic oxidation of an inexpensive bromide salt (*e.g.*, NaBr) to generate a highly reactive, electrophilic bromine species *in situ*. Subsequent electrophilic addition across the alkene moiety of the allylic alcohol, followed by a tandem nucleophilic attack by the tethered hydroxyl group, cleanly delivers the target epibromohydrin architecture.

While the electrochemical generation of bromine electrophiles is inherently clean and well-established, enforcing absolute stereocontrol during the subsequent cyclization event remains a formidable challenge.^10^ The high reactivity of electrogenerated Br_2_ or Br^+^ species often triggers an unselective background pathway, resulting in poor enantioselectivity. To tame this reactivity and induce enantiocontrol, a biphasic system employing the dual actions of a chiral counter anion source and a charged phase-transfer catalyst (PTC) represents a promising solution. However, despite the profound conceptual synergy between interfacial electrosynthesis and chiral phase-transfer catalysis, their joint implementation remains significantly underdeveloped.^10^ The core challenge rests on a delicate kinetic equilibrium: balancing the rate of anodic bromide oxidation with the rate of chiral phase-transfer encapsulation, while maintaining efficient chemical bond formation. Building upon our ongoing interest in this area,^11^ herein we report a highly efficient and enantioselective protocol for the asymmetric synthesis of epibromohydrins.

## Results and discussion

We began our investigation by employing allylic alcohol 1a as the model substrate (Table 1). Following a systematic evaluation of various chiral phosphoric acids (CPAs) as chiral counter anion sources and ammonium salts as phase-transfer catalysts (PTCs), a specific combination of CPA-1 and PTC-1 was identified as optimal (see the SI for full details). Extensive optimization of other reaction parameters was also conducted. Utilizing NaBr as a bromide source, the target bromoepoxide 2a was obtained in 85% yield and 95% ee by electrolyzing the mixture in a DCM/H_2_O biphasic solvent system within an undivided cell at a constant current of 2 mA at −15 °C.^12^ Notably, under these optimized conditions, the formation of side product 2a′, which arises from the direct dibromination of the olefin, was completely suppressed.^13^

**Table 1:** Evaluation of reaction conditions^a^

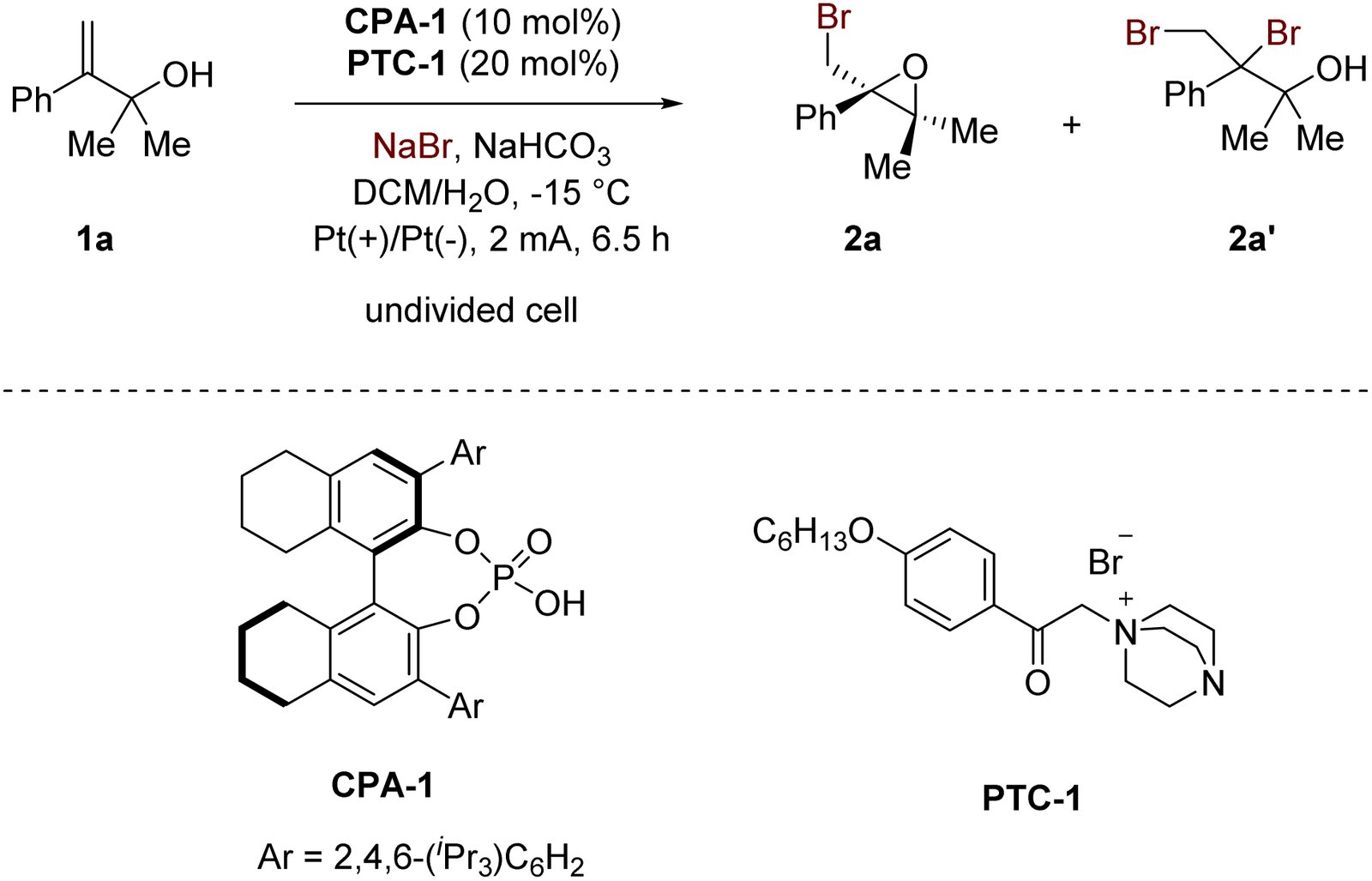			
Entry	Variation of conditions	Yield of^b^2a (%)	ee of^c^2a (%)
1	None	85	95
2	No electricity	0	—
3	No water	0	—
4	No CPA-1	10	0
5	No PTC-1	28	47
6	No NaHCO_3_	56	70
7	1 mA, 12 h	27	83
8	4 mA, 3 h	64	91

a Reaction conditions: undivided cell, Pt anode and cathode (1.0 × 1.0 cm^2^), 2 mA, 1a (0.2 mmol), CPA-1 (10 mol%), PTC-1 (20 mol%), NaBr (5.0 M), NaHCO_3_ (0.1 M), DCM/H_2_O (4 mL/5 mL), −15 °C, 6.5 h.

b Yield was determined by crude ^1^H NMR analysis using CH_2_Br_2_ as an internal standard.

c The ee value was determined by chiral HPLC.

Control experiments revealed that the reaction did not proceed in the absence of electricity or water (entries 2–3). Omitting the CPA catalyst led to a drastic drop in efficiency, affording 2a in only 10% yield with no enantioselectivity (entry 4). This low yield strongly implies that the CPA acts not only as a source of chirality but also as a reaction accelerator, likely by facilitating the interfacial phase-transfer process. Similarly, the omission of the PTC significantly compromised both yield and enantioselectivity (entry 5), highlighting a crucial synergistic effect between the two catalytic components. Furthermore, omitting NaHCO_3_ led to a substantial decrease in both yield and asymmetric induction (entry 6). The essential role of NaHCO_3_ is likely tied to buffering the system within a suitable pH range; because an equivalent of HBr is generated and anodic oxidation is coupled with the cathodic reduction of water, both events can drastically perturb the acidity of the reaction media.

We also investigated the influence of the electrolysis current on the reaction outcome (entries 7–8). Deviating from the optimal current (either higher or lower) resulted in diminished yields and enantioselectivities. This behavior underscores the necessity of matching the rate of electrochemically generated Br_2_ with the intrinsic kinetics of the organocatalyzed bromination pathway. Notably, it remains elusive to achieve high enantioselectivity in related bromoetherification transformations using conventional chemical bromination strategy. Thus, enabled by controlled generation of reactive bromine species, the present electrochemical approach represents a complementary strategy.

With the optimized reaction conditions in hand, we next explored the substrate scope (Table 2). A range of tertiary allylic alcohols participated smoothly in this electrochemical bromoetherification protocol. Substrates bearing various substituents at the *meta*- and *para*-positions of the aryl ring reacted efficiently without compromising reactivity or enantioselectivity. However, substitution at the *ortho*-position exerted a detrimental impact on the enantioselectivity (2h), presumably due to steric hindrance. Notably, tetrasubstituted epoxides incorporating a spirocyclic framework could also be constructed with high efficiency. The electrochemical conditions were compatible with an array of sensitive functional groups, including halides, ethers, tosylamides, and acetals. Furthermore, a benzothiophene heterocycle remained completely intact under these oxidative conditions. When the two substituents at the allylic position were different, the reaction faced diastereoselectivity challenges during oxirane formation. Specifically, product 2u was obtained as a 3.4 : 1 diastereomeric mixture, though both isomers were delivered with excellent enantioselectivity. Unfortunately, the diastereoselectivity was not further improved after brief evaluation of other catalysts and reaction parameters (*e.g.*, temperature). Finally, an aliphatic olefin substrate also underwent the desired transformation (2v), albeit with moderate asymmetric induction.

**Table 2:** Reaction scope^a^

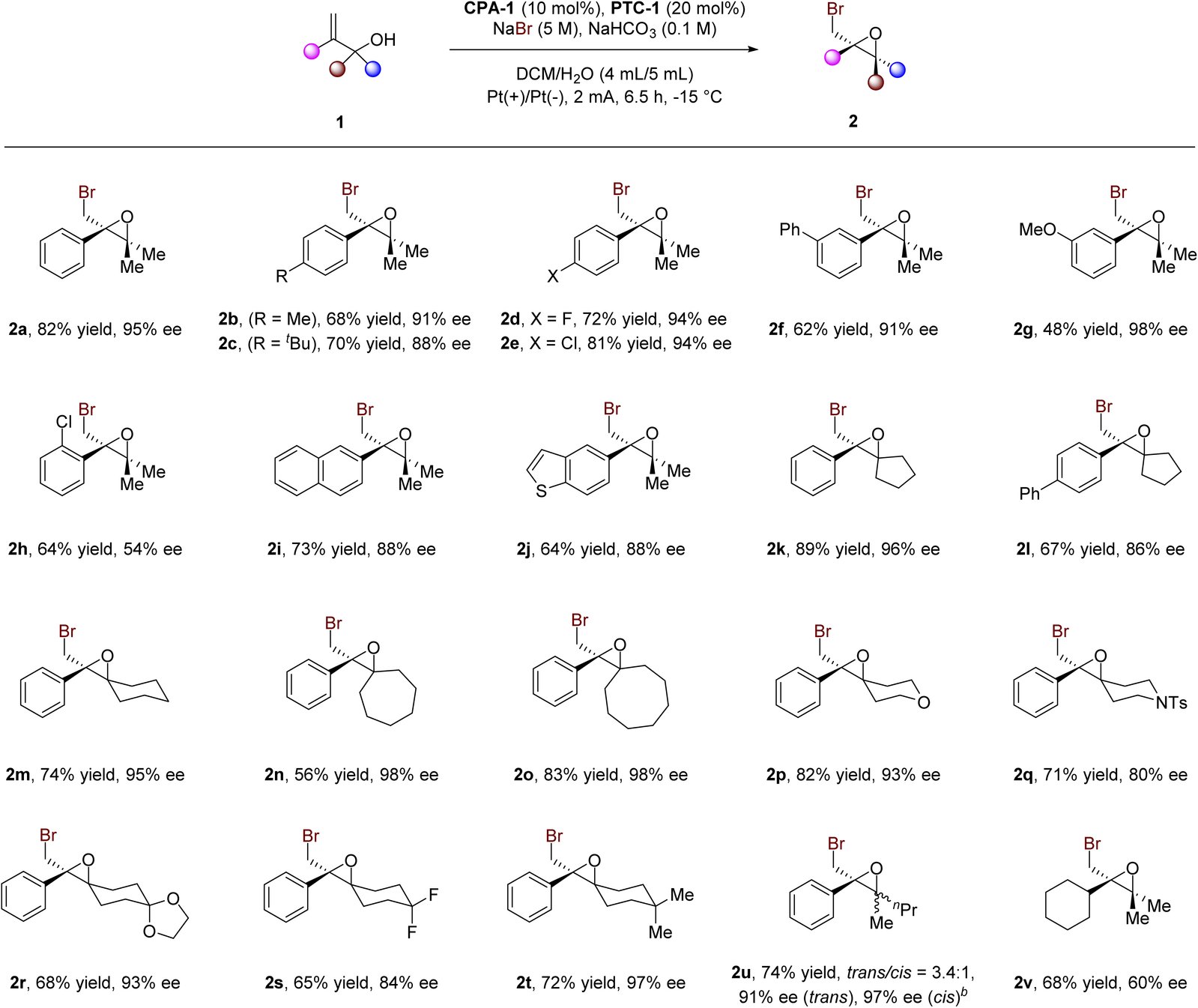

a Pt anode and cathode (1.0 × 1.0 cm^2^), 2 mA, 1 (0.2 mmol), CPA-1 (10 mol%), PTC-1 (20 mol%), NaBr (5.0 M), NaHCO3 (0.1 M), DCM/H2O (4 mL/5 mL), −15 °C, 6.5 h. Isolated yield. The ee value was determined by chiral HPLC.

b Yield and ee of the derived thioester was recorded (see the SI for details).

**Scheme 1:**
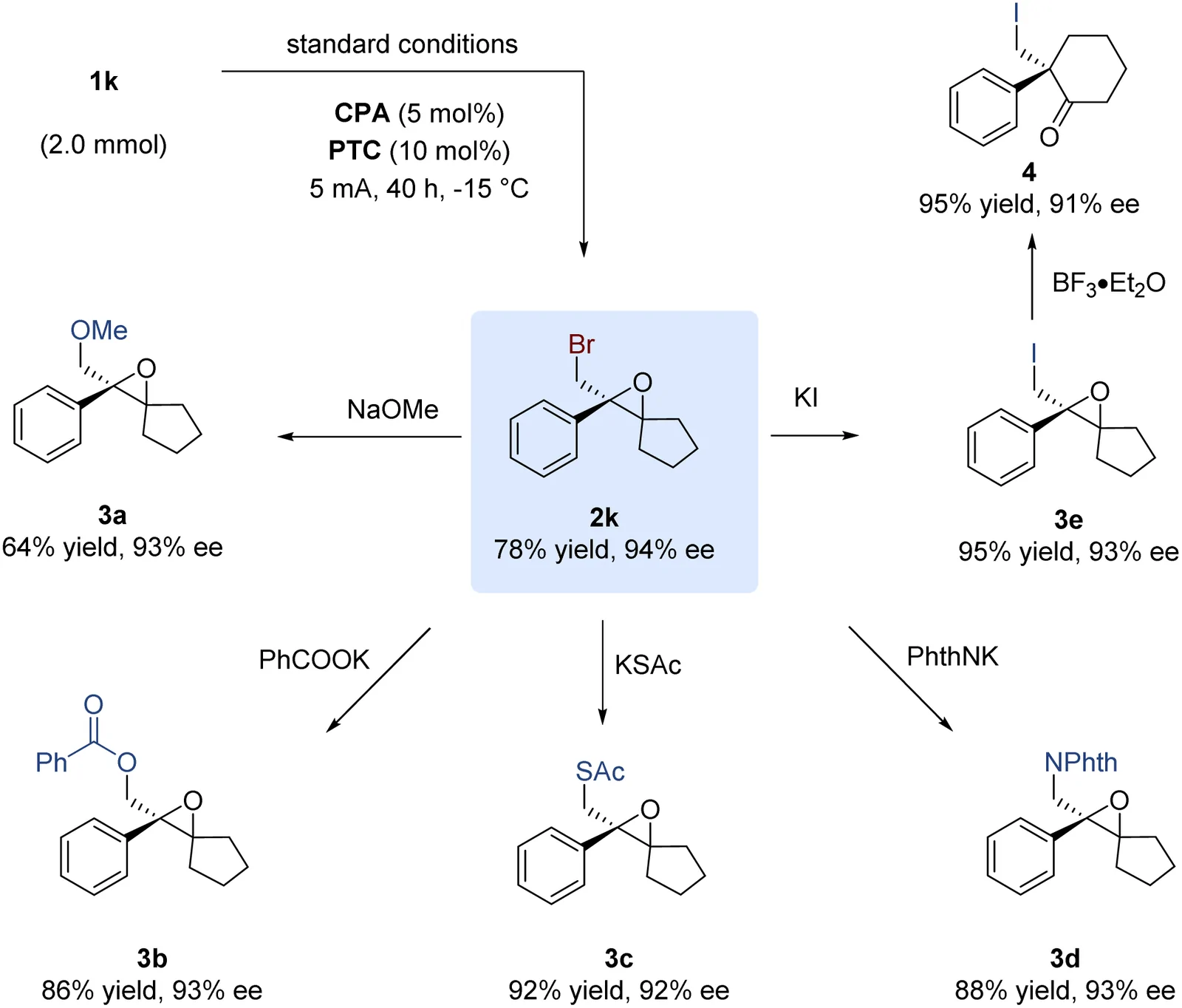
Scale-up reaction and product transformations.

The robustness of this electrochemical protocol was demonstrated by a 2.0 mmol scale reaction of 1k (Scheme 1). Utilizing a reduced loading of both CPA-1 and PTC-1 catalysts, the electrolysis proceeded efficiently at a constant current of 5 mA to deliver 2k in 78% yield and 94% ee. This class of enantioenriched bromoepoxide serves as a versatile platform for accessing a variety of valuable chiral scaffolds. For instance, facile nucleophilic substitution at the primary alkyl bromide motif enabled the synthesis of functionalized chiral epoxides bearing ether (3a), ester (3b), thioester (3c), phthalimide (3d), and iodo (3e) groups. Furthermore, the enantioenriched iodoepoxide 3e underwent a smooth Lewis acid-mediated rearrangement upon treatment with boron trifluoride etherate, affording cyclohexanone 4, which features an α-all-carbon quaternary stereocenter. Importantly, the optical purity of the substrates was retained without obvious loss in these transformations.

**Scheme 2:**
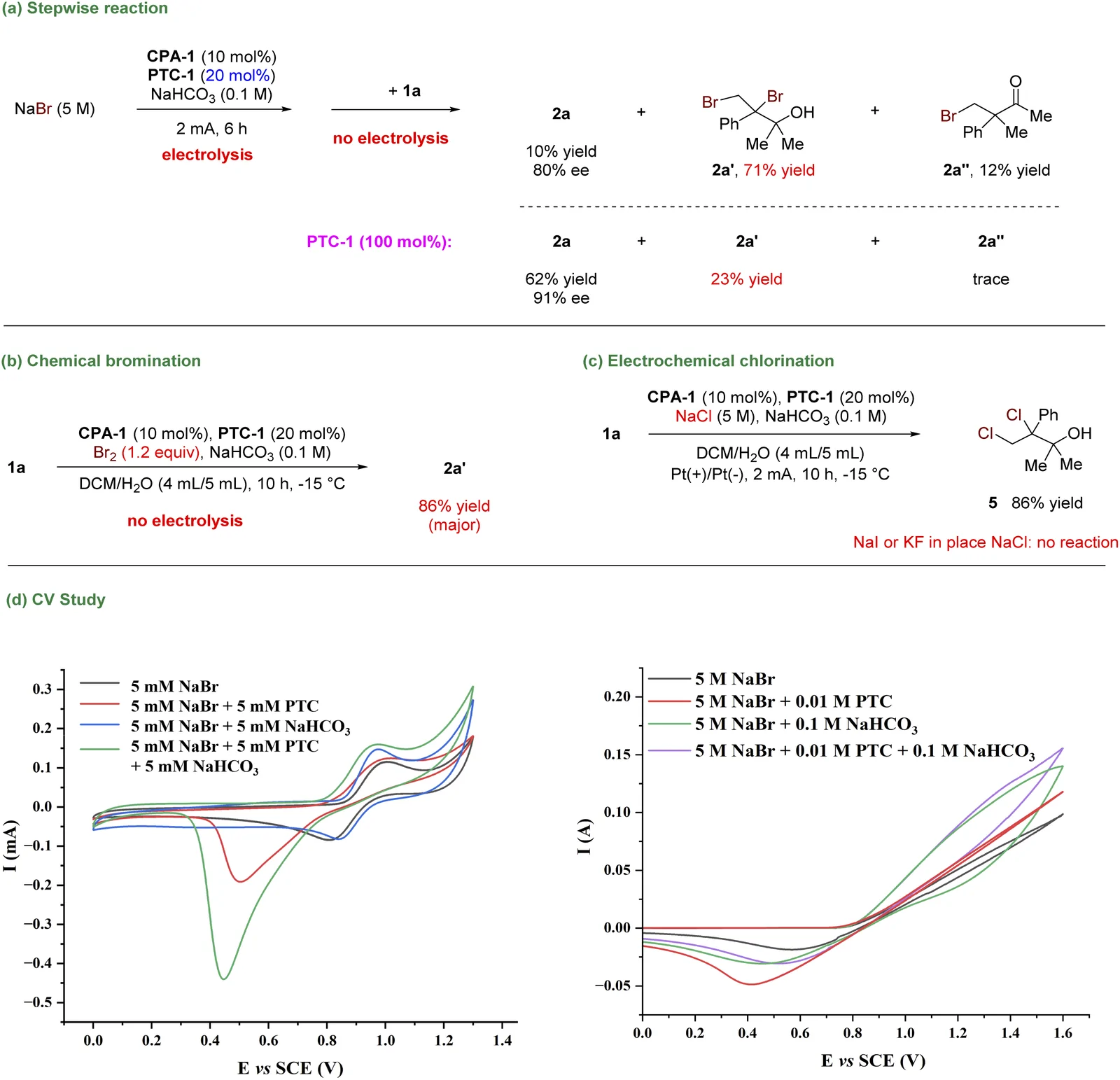
Control experiment and cyclic voltammetry study.

To gain deeper insights into the reaction mechanism, a series of control experiments were performed (Scheme 2). We first sought to evaluate the significance of the temporal synergy between the anodic oxidation of bromide and the subsequent bromoetherification event. To achieve this, these two processes were temporally isolated: electrolysis of NaBr was initially conducted in the absence of substrate 1a for 6 hours to generate a stoichiometric amount of Br_2_*in situ*. Substrate 1a was subsequently introduced to simulate the standard reaction conditions. Under these conditions, the desired product 2a was obtained in a mere 10% yield and 80% *ee*. Instead, the olefin dibromination adduct 2a′ was isolated as the major product (71% yield), accompanied by a trace amount of the semipinacol rearrangement byproduct 2a″. Notably, the use of a stoichiometric amount of PTC-1 led to a dramatic increase in the yield of 2a (62% yield, 91% ee) and marked suppression of the formation of byproducts 2a′ and 2a″ (Scheme 2a). A similar product distribution was observed when commercially available, stoichiometric Br_2_ was used directly (Scheme 2b). These results imply that while free Br_2_ (either electrogenerated or commercially purchased) is highly reactive toward olefin dibromination to form 2a′, it is kinetically less competent to drive the desired asymmetric bromoetherification pathway toward 2a. However, in the presence of the PTC catalyst, the pathway leading to 2a is significantly accelerated, effectively outcompeting the dibromination process. Because the PTC was present in only catalytic quantities during the temporal isolation experiment, the yield of 2a remained low. Conversely, under standard electrolytic conditions, the controlled, slow generation of Br_2_ matches the kinetics of the catalytic cycle and PTC turnover, ensuring that bromoetherification remains the dominant pathway. This hypothesis is consistent with the observed influence of the electrolysis rate (current density) on product distribution.

We next investigated the compatibility of other halogens within this protocol. Under otherwise identical conditions, replacing NaBr with NaCl failed to yield the desired chloroetherification product; instead, the racemic dichloride 5 was isolated in 86% yield. Taking consideration of the results in Scheme 2a, the preference for dichloride formation might be related to distinct reaction kinetics during the halide (chloride *vs.* bromide) attack on the halonium intermediate relative to rearrangement. Furthermore, employing NaI resulted in no reaction, presumably due to the relative instability of the transient electrophilic iodine species, which prevented the desired reaction pathway. The use of KF as halide source did not lead to the desired fluorination product either, which is likely due to high oxidation potential of fluoride.

Finally, cyclic voltammetry (CV) studies were conducted to probe the interfacial electron transfer process (Scheme 2d). Under dilute electrolyte conditions, the addition of PTC or NaHCO_3_ resulted in changes in the bromide oxidation response, suggesting that these components may affect the interfacial electrochemical environment (Scheme 2d, left). Notably, a control CV study using the phase-transfer catalyst bearing an inert counterion (BF_4_^−^ in place Br^−^) was also performed, indicating the cation motif of PTC is not oxidized (see Fig. S3 in SI). To better simulate the standard conditions, additional CV measurements were performed using concentrated NaBr electrolyte in distilled water with DCM as co-solvent (Scheme 2d, right). Although no distinct oxidation peak was observed under these conditions, the presence of PTC and/or NaHCO_3_ altered the anodic current response, which is consistent with the interaction/reaction of these two species with the *in situ* generated reactive bromine species.

**Fig. 2:**
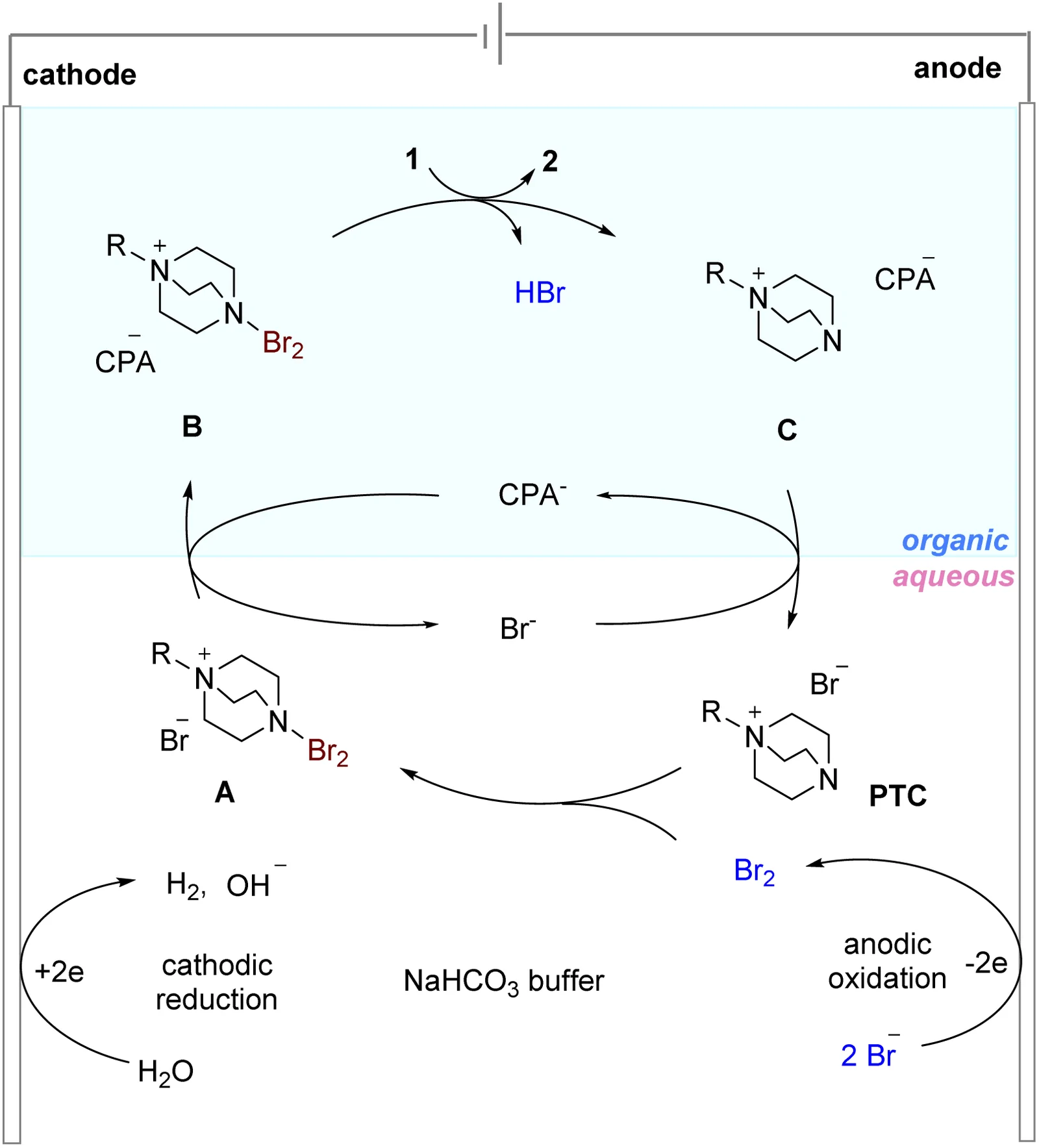
Mechanistic hypothesis for the asymmetric electrochemical bromoetherification.

Based on these results, we proposed a possible mechanism (Fig. 2). The bromide anion is initially oxidized to Br_2_ at the anode, which is trapped by PTC to form complex A. Subsequent anion exchange with the chiral phosphate anion (CPA^−^) produces a more lipophilic, organic-soluble ion pair B, which promotes the enantioselective bromoetherification of substrate 1 in the organic phase. Along with the desired bromo epoxide product 2, this step regenerates the PTC-CPA ion pair C together with a molecule of HBr. HBr is neutralized by the basic environment, which is maintained by the NaHCO_3_ buffer or the OH^−^ ions produced *via* cathodic water reduction. Concurrently, upon anion exchange with Br^−^, intermediate C re-enters the aqueous phase to serve as the PTC catalyst for a new catalytic cycle. The liberated chiral phosphate anion undergoes ion-exchange with another molecule of A to facilitate phase transfer.

## Conclusions

In conclusion, we have developed an efficient and enantioselective electrochemical bromoetherification of allylic tertiary alcohols, providing a step-economical platform to access enantioenriched epibromohydrins. By merging interfacial electrosynthesis with a dual chiral phase-transfer/chiral counter anion catalytic system, this protocol tames the high reactivity of electrogenerated Br_2_ and effectively suppresses unselective background pathways. Without the need for stoichiometric chemical oxidants, the reaction utilizes inexpensive NaBr as the bromine source to deliver the desired bromoepoxides in excellent yields and with high levels of asymmetric induction. Mechanistic investigations and cyclic voltammetry studies provided useful insights into the catalytic cycle. The practical utility of this method was further demonstrated by a successful 2.0 mmol reaction and a series of downstream functionalizations that cleanly forged complex chiral scaffolds, including an α-all-carbon quaternary stereocenter. Overall, this strategy represents a complementary approach for the asymmetric synthesis of the valuable epibromohydrins.

## Author contributions

Z. H. and M. F. designed the experiments and analyzed the data. M. F., P. S. and S. L. contributed to the preparation of substrates. Z. H., H. H. and J. S. supervised the project and wrote the manuscript. All authors contributed to discussions and comments on the manuscript.

## Conflicts of interest

There are no conflicts to declare.

## Supplementary Material

SC-OLF-D6SC05589C-s001

## Data Availability

The data supporting this article have been included as part of the supplementary information (SI). Supplementary information: experimental procedures and characterization data for all new compounds. For SI or other electronic formate included here. See DOI: https://doi.org/10.1039/d6sc05589c.
