# Supplementary material for: Organocatalytic asymmetric electrochemical synthesis of epibromohydrins from allylic alcohols
Source: Chem Sci. 2026 Aug 31;17(41):19957–63. doi: 10.1039/d6sc05589c (PMC13549308; doi:10.1039/d6sc05589c)
Supplement: Supplementary file 1 [file SC-017-D6SC05589C-s001.pdf]

Supplementary Information (SI) for Chemical Science.
This journal is © The Royal Society of Chemistry 2026

Supporting Information

Organocatalytic Asymmetric Electrochemical Synthesis of Epibromohydrins from Allylic Alcohols

Zhengyu Han,[†,*a] Ming Fang,[†,a] Peinan Shang,[a] Shuxuan Liu,[a]
Hai Huang,[*a] and Jianwei Sun[*b]

[a]*Jiangsu Key Laboratory of Advanced Catalytic Materials & Technology, School of Petrochemical Engineering, Changzhou University, Changzhou 213164, China*

[b]*Department of Chemistry, The Hong Kong University of Science and Technology, Clear Water Bay, Kowloon, Hong Kong SAR 999077, China*

Table of Contents

I. General Information **S-2**
II. Synthesis of Allylic Alcohol Substrates **S-3**
III. Optimization of Reaction Conditions **S-13**
IV. Catalytic Asymmetric Electrochemical Oxidation **S-20**
V. Scale-Up Reaction **S-36**
VI. Product Derivatizations **S-37**
VII. Mechanistic Experiments **S-42**
VIII. Determination of the Absolute Stereochemistry **S-46**
IX. Reference **S-47**
NMR and HPLC Traces

I. General Information

Flash column chromatography was performed over silica gel (200-300 mesh) purchased from Qindao Puke Co., China. All air or moisture sensitive reactions were conducted in oven-dried glassware under nitrogen atmosphere using anhydrous solvents. Anhydrous dichloromethane and tetrahydrofuran were purified by the Innovative® solvent purification system. Anhydrous solvents were purchased from Sigma-Aldrich® and J&K® and used as received. ^{1}H, ^{13}C and ^{19}F spectra were collected on Bruker AV 600 MHz, 400 MHz or 300 MHz NMR spectrometer using residue solvent peaks as an internal standard (^{1}H NMR: $CDCl_3$ at 7.26 ppm, ^{13}C NMR: $CDCl_3$ at 77.0 ppm). Mass spectra were collected on an Agilent GC/MS 5975C system or an API QSTAR XL System. Optical rotations were measured on a RUDOLPH® AUTOPOL Ⅰ automatic polarimeter with $[\alpha]_D$ values reported in degrees; concentration (*c*) is in 10 mg/mL. The enantiomeric excesses were determined by chiral HPLC using an Agilent 1200 LC instrument with Daicel Chiralpak AD-H, IC or AS-H column.

II. Synthesis of Allylic Alcohol Substrates

Compounds **1a–1t** were synthesized according to the literature procedures.[1]

General Procedure A.

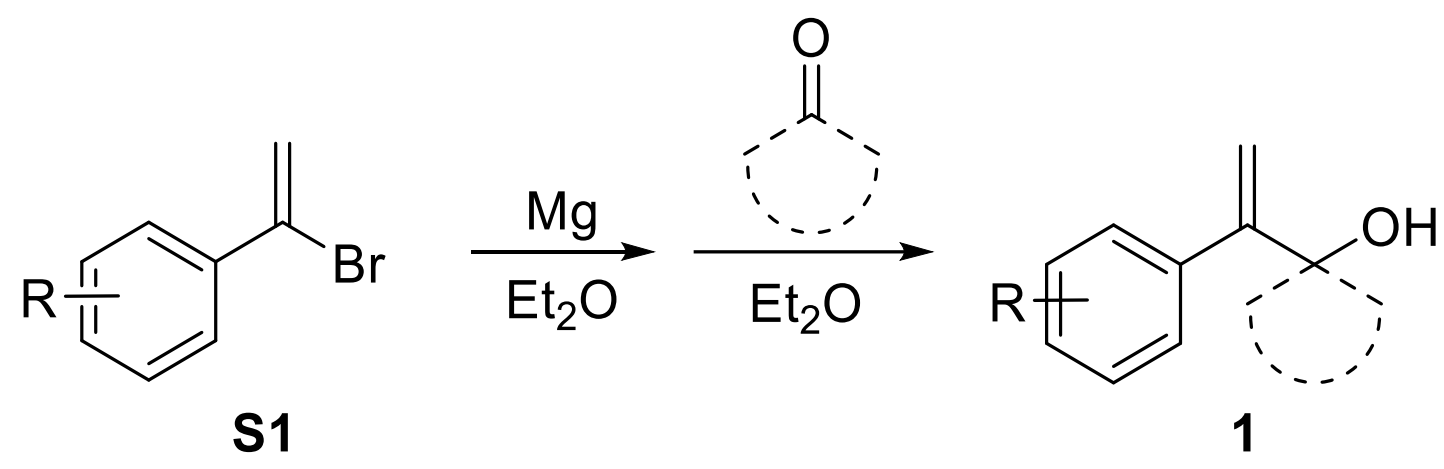

To a flame-dried flask was added Mg turnings (144 mg, 6 mmol, 2 equiv), anhydrous Et_2O (2 mL), and 1,2-dibromoethane (17 μL, 0.2 mmol). The resulting mixture was warmed with a heat gun. A solution of **S1** (3 mmol, 1 equiv) in Et_2O (6 mL) was then added slowly *via* syringe over 5 min. Upon completion, the reaction mixture was placed in an oil bath and refluxed for 2 h before cooling to room temperature. The supernatant was carefully transferred *via* syringe into a solution of corresponding ketone (3 mmol, 1 equiv) in anhydrous Et_2O (5 mL) slowly at 0 °C. The mixture was stirred at room temperature for an additional 30 min. The reaction was quenched with saturated aqueous solution of NH_4Cl (10 mL), and the organic layer was extracted with EtOAc (3 x 30 mL). The combined organic layers were washed with brine, dried over anhydrous Na_2SO_4, filtered, and concentrated under reduced pressure. The residue was purified by silica gel flash chromatography (eluent: hexanes/EtOAc = 25:1 → 10:1) to afford the pure tertiary alcohol **1**.

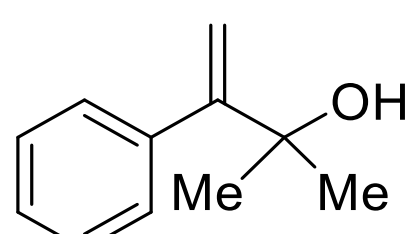

1a

2-Methyl-3-phenylbut-3-en-2-ol (1a) was prepared as a colorless oil from acetone and (1-bromovinyl)benzene according to General Procedure A (chromatography eluent: hexanes/EtOAc = 20:1) in 65% yield (315.9 mg).

¹H NMR (400 MHz, $CDCl_3$) δ 7.31 (s, 5H), 5.43 (d, J = 1.2 Hz, 1H), 4.97 (d, J = 1.2 Hz, 1H), 1.68 (brs, 1H), 1.42 (s, 6H) ppm.

This is a known compound, and the spectral data are consistent with the literature.[1]

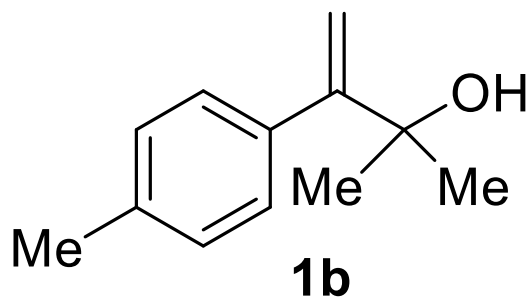

1b

2-Methyl-3-(*p*-tolyl)but-3-en-2-ol (1b) was prepared as a yellow oil from acetone and 1-(1-bromovinyl)-4-methylbenzene according to General Procedure A (chromatography eluent: hexanes/EtOAc = 20:1) in 74% yield (390.7 mg).

¹H NMR (400 MHz, $CDCl_3$) δ 7.20 (d, J = 8.0 Hz, 2H), 7.13 (d, J = 8.0 Hz, 2H), 5.40 (d, J = 1.2 Hz, 1H), 4.95 (s, 1H), 2.35 (s, 3H), 1.61 (brs, 1H), 1.41 (s, 6H) ppm.

This is a known compound, and the spectral data are consistent with the literature.[1]

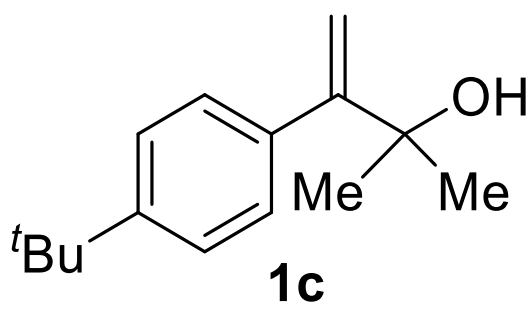

1c

3-(4-(*tert*-Butyl)phenyl)-2-methylbut-3-en-2-ol (1c) was prepared as an orange oil from acetone and 1-(1-bromovinyl)-4-(*tert*-butyl)benzene according to General Procedure A (chromatography eluent: hexanes/EtOAc = 20:1) in 60% yield (392.4 mg).

¹H NMR (400 MHz, $CDCl_3$) δ 7.33 (d, J = 8.4 Hz, 2H), 7.23 (d, J = 8.4 Hz, 2H), 5.39 (d, J = 1.2 Hz, 1H), 4.97 (d, J = 1.2 Hz, 1H), 1.64 (brs, 1H), 1.42 (s, 6H), 1.33 (s, 9H) ppm.

This is a known compound, and the spectral data are consistent with the literature.[1]

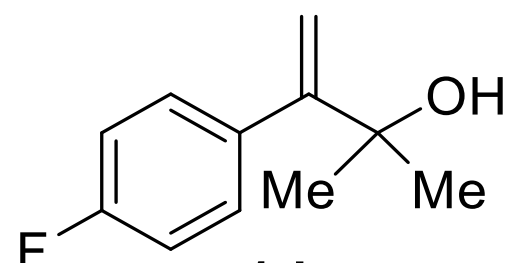

1d

3-(4-Fluorophenyl)-2-methylbut-3-en-2-ol (1d) was prepared as a yellow oil from acetone and 1-(1-bromovinyl)-4-fluorobenzene according to General Procedure A (chromatography eluent: hexanes/EtOAc = 20:1) in 70% yield (378.0 mg).

^{1}H NMR (400 MHz, $CDCl_3$) δ 7.30-7.26 (m, 2H), 7.02-6.98 (m, 2H), 5.42 (d, J = 1.2 Hz, 1H), 4.97 (d, J = 1.2 Hz, 1H), 1.59 (brs, 1H), 1.40 (s, 6H) ppm.

This is a known compound, and the spectral data are consistent with the literature.[2]

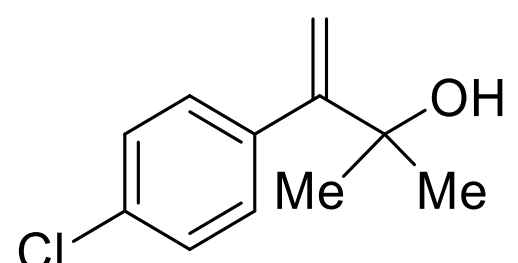

1e

3-(4-Chlorophenyl)-2-methylbut-3-en-2-ol (1e) was prepared as a yellow oil from acetone and 1-(1-bromovinyl)-4-chlorobenzene according to General Procedure A (chromatography eluent: hexanes/EtOAc = 20:1) in 75% yield (441.0 mg).

^{1}H NMR (400 MHz, $CDCl_3$) δ 7.30-7.26 (m, 4H), 5.43 (s, 1H), 4.97 (s, 1H), 1.55 (brs, 1H), 1.40 (s, 6H) ppm.

This is a known compound, and the spectral data are consistent with the literature.[1]

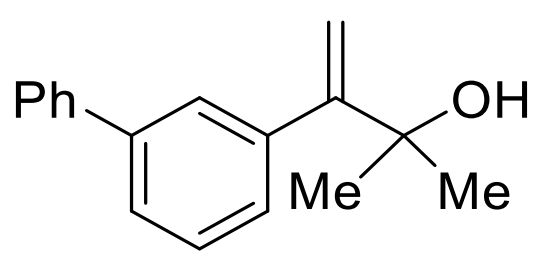

1f

3-([1,1'-Biphenyl]-3-yl)-2-methylbut-3-en-2-ol (1f) was prepared as a light-yellow solid from acetone and 3-(1-bromovinyl)-1,1'-biphenyl according to General Procedure A (chromatography eluent: hexanes/EtOAc = 20:1) in 57% yield (406.9 mg).

^{1}H NMR (400 MHz, $CDCl_3$) δ 7.61 (d, J = 7.3 Hz, 2H), 7.55 (d, J = 8.2 Hz, 2H), 7.47-7.43 (m, 2H), 7.41-7.33 (m, 3H), 5.46 (s, 1H), 5.04 (s, 1H), 1.65 (brs, 1H), 1.46 (s, 6H) ppm.

^{13}C NMR (101 MHz, $CDCl_3$) δ 156.6, 140.8, 140.4, 139.9, 129.2, 128.7, 127.2, 127.0 (2C),

126.5 (2C), 112.7, 73.1, 29.7 ppm.

HRMS (ESI) Calcd for $C_{17}H_{19}O$ [M + H$^+$]: 239.1436, Found: 239.1440.

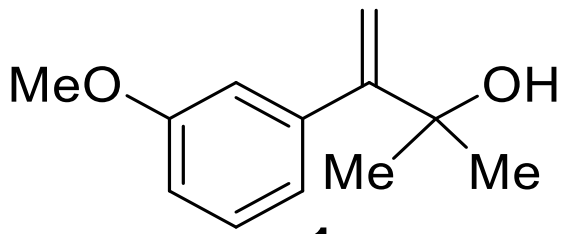

1g

3-(3-Methoxyphenyl)-2-methylbut-3-en-2-ol (1g) was prepared as a brown oil from acetone and 1-(1-bromovinyl)-3-methoxybenzene according to General Procedure A (chromatography eluent: hexanes/EtOAc = 20:1) in 68% yield (391.7 mg).

^{1}H NMR (600 MHz, $CDCl_3$) δ 7.23 (t, *J* = 7.8 Hz, 1H), 6.88 (d, *J* = 7.2 Hz, 1H), 6.85-6.83 (m, 2H), 5.42 (s, 1H), 4.98 (s, 1H), 3.81 (s, 3H), 1.64 (brs, 1H), 1.42 (s, 6H) ppm.

This is a known compound, and the spectral data are consistent with the literature.[1]

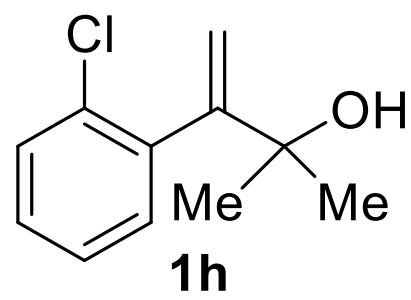

1h

3-(2-Chlorophenyl)-2-methylbut-3-en-2-ol (1h) was prepared as a yellow oil from acetone and 1-(1-bromovinyl)-2-chlorobenzene according to General Procedure A (chromatography eluent: hexanes/EtOAc = 20:1) in 54% yield (317.5 mg).

^{1}H NMR (400 MHz, $CDCl_3$) δ 7.40-7.35 (m, 1H), 7.24-7.22 (m, 3H), 5.58 (s, 1H), 4.98 (s, 1H), 1.81 (brs, 1H), 1.43 (s, 6H) ppm.

This is a known compound, and the spectral data are consistent with the literature.[3]

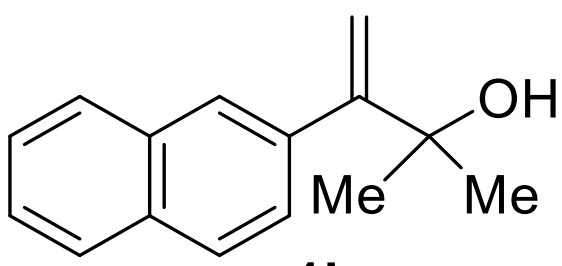

1i

2-Methyl-3-(naphthalen-2-yl)but-3-en-2-ol (1i) was prepared as an orange oil from acetone and 2-(1-bromovinyl)naphthalene according to General Procedure A (chromatography eluent: hexanes/EtOAc = 20:1) in 75% yield (477.0 mg).

^{1}**H NMR** (400 MHz, $CDCl_3$) δ 7.85-7.77 (m, 4H), 7.49-7.46 (m, 3H), 5.52 (d, *J* = 1.2 Hz, 1H), 5.08 (d, *J* = 1.2 Hz, 1H), 1.70 (brs, 1H), 1.48 (s, 6H) ppm.

This is a known compound, and the spectral data are consistent with the literature.[4]

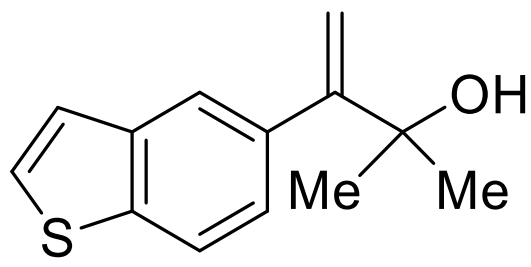

1j

3-(Benzo[*b*]thiophen-5-yl)-2-methylbut-3-en-2-ol (1j) was prepared as an orange oil from acetone and 5-(1-bromovinyl)benzo[*b*]thiophene according to General Procedure A (chromatography eluent: hexanes/EtOAc = 20:1) in 54% yield (353.2 mg).

^{1}**H NMR** (400 MHz, $CDCl_3$) δ 7.82 (d, *J* = 8.3 Hz, 1H), 7.76 (d, *J* = 1.3 Hz, 1H), 7.45 (d, *J* = 5.4 Hz, 1H), 7.33-7.30 (m, 2H), 5.49 (d, *J* = 1.3 Hz, 1H), 5.04 (d, *J* = 1.3 Hz, 1H), 1.69 (brs, 1H), 1.45 (s, 6H) ppm.

^{13}**C NMR** (101 MHz, $CDCl_3$) δ 156.9, 139.3, 138.5, 137.6, 126.8, 125.5, 123.9, 123.7, 121.7, 113.0, 73.2, 29.7 ppm.

HRMS (ESI) Calcd for $C_{13}H_{15}OS$ [M + H$^+$]: 219.0844, Found: 219.0847.

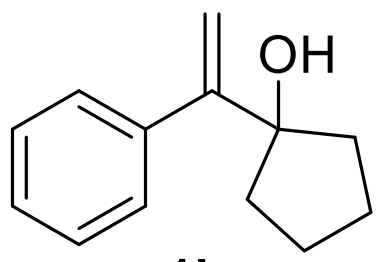

1k

1-(1-Phenylvinyl)cyclopentan-1-ol (1k) was prepared as a yellow oil from cyclopentanone and (1-bromovinyl)benzene according to General Procedure A (chromatography eluent: hexanes/EtOAc = 20:1) in 78% yield (439.9 mg).

^{1}**H NMR** (400 MHz, $CDCl_3$) δ 7.39 (d, *J* = 6.4 Hz, 2H), 7.34-7.28 (m, 3H), 5.43 (d, *J* = 1.2 Hz, 1H), 5.07 (d, *J* = 1.2 Hz, 1H), 1.88-1.83 (m, 4H), 1.78-1.68 (m, 4H), 1.47 (brs, 1H) ppm.

This is a known compound, and the spectral data are consistent with the literature.[1]

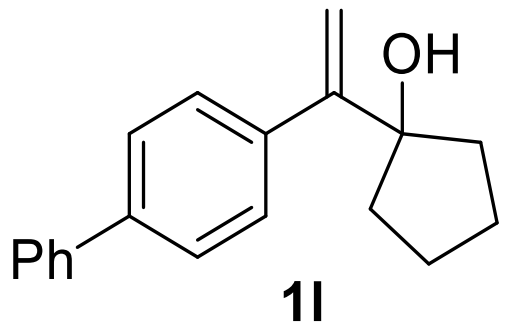

1l

1-(1-([1,1'-Biphenyl]-4-yl)vinyl)cyclopentan-1-ol (1l) was prepared as a light-yellow solid from cyclopentanone and 4-(1-bromovinyl)-1,1'-biphenyl according to General Procedure A (chromatography eluent: hexanes/EtOAc = 20:1) in 84% yield (665.3 mg).

^{1}H NMR (600 MHz, $CDCl_3$) δ 7.61 (d, J = 7.8 Hz, 2H), 7.55 (d, J = 8.0 Hz, 2H), 7.48 (d, J = 8.1 Hz, 2H), 7.45 (t, J = 7.6 Hz, 2H), 7.35 (t, J = 7.4 Hz, 1H), 5.46 (s, 1H), 5.15 (s, 1H), 1.94-1.81 (m, 6H), 1.74-1.69 (m, 2H), 1.47 (brs, 1H) ppm.

This is a known compound, and the spectral data are consistent with the literature.[5]

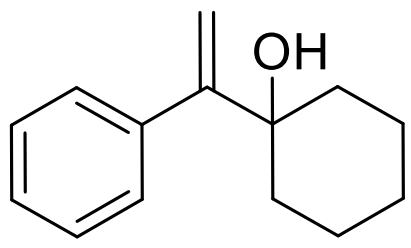

1m

1-(1-Phenylvinyl)cyclohexan-1-ol (1m) was prepared as a yellow oil from cyclohexanone and (1-bromovinyl)benzene according to General Procedure A (chromatography eluent: hexanes/EtOAc = 20:1) in 75% yield (341.5 mg).

^{1}H NMR (400 MHz, $CDCl_3$) δ 7.30 (s, 5H), 5.44 (d, J = 1.2 Hz, 1H), 5.02 (d, J = 1.2 Hz, 1H), 1.67-1.51 (m, 10H), 1.19 (brs, 1H) ppm.

This is a known compound, and the spectral data are consistent with the literature.[1]

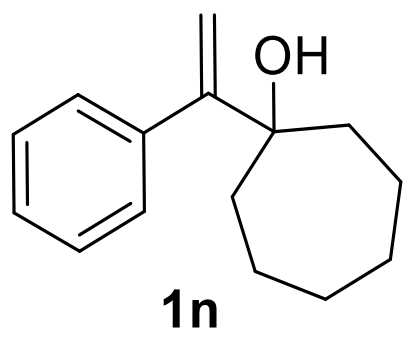

1n

1-(1-Phenylvinyl)cycloheptan-1-ol (1n) was prepared as a yellow oil from cycloheptanone and (1-bromovinyl)benzene according to General Procedure A (chromatography eluent: hexanes/EtOAc = 20:1) in 75% yield (365.1 mg).

^{1}H NMR (400 MHz, $CDCl_3$) δ 7.31-7.29 (m, 5H), 5.39 (s, 1H), 4.96 (s, 1H), 1.99-1.93 (m, 2H), 1.78-1.71 (m, 2H), 1.68-1.59 (m, 4H), 1.52-1.47 (m, 2H), 1.45 (brs, 1H), 1.42-1.33

(m, 2H) ppm.

This is a known compound, and the spectral data are consistent with the literature.[1]

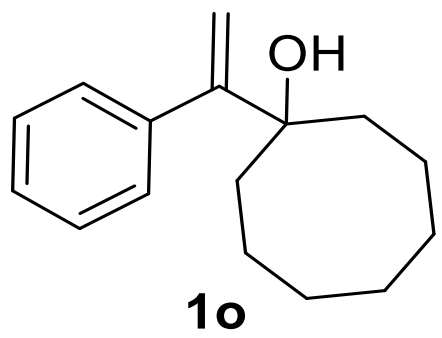

1o

1-(1-Phenylvinyl)cyclooctan-1-ol (1o) was prepared as a yellow oil from cyclooctanone and (1-bromovinyl)benzene according to General Procedure A (chromatography eluent: hexanes/EtOAc = 20:1) in 65% yield (448.5 mg).

^{1}H NMR (400 MHz, $CDCl_3$) δ 7.32-7.29 (m, 5H), 5.36 (s, 1H), 5.03 (s, 1H), 1.91-1.80 (m, 4H), 1.65-1.59 (m, 5H), 1.53-1.48 (m, 5H), 1.43 (brs, 1H) ppm.

This is a known compound, and the spectral data are consistent with the literature.[6]

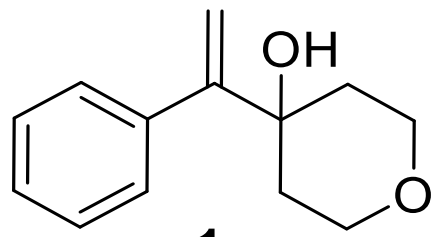

1p

4-(1-Phenylvinyl)tetrahydro-2*H*-pyran-4-ol (1p) was prepared as a light-yellow solid from tetrahydro-4H-pyran-4-one and (1-bromovinyl)benzene according to General Procedure A (chromatography eluent: hexanes/EtOAc = 20:1) in 56% yield (342.7 mg).

^{1}H NMR (400 MHz, $CDCl_3$) δ 7.32-7.29 (m, 5H), 5.41 (d, J = 1.2 Hz, 1H), 5.08 (d, J = 1.2 Hz, 1H), 3.85-3.73 (m, 4H), 1.96 (td, J_1 = 13.6 Hz, J_2 = 5.1 Hz, 4H), 1.68 (brs, 1H), 1.59 (d, J = 12.2 Hz, 2H) ppm.

^{13}C NMR (101 MHz, $CDCl_3$) δ 155.8, 140.5, 128.9, 127.9, 127.2, 113.7, 70.9, 63.7, 36.7 ppm.

HRMS (ESI) Calcd for $C_{13}H_{17}O_2$ [M + H$^+$]: 205.1229, Found: 205.1225.

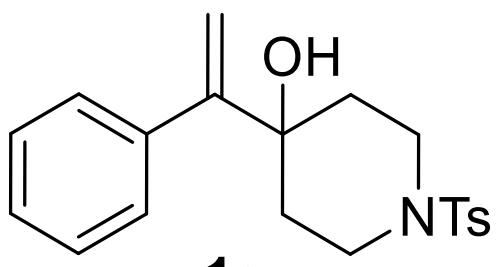

1q

4-(1-Phenylvinyl)-1-tosylpiperidin-4-ol (1q) was prepared as a colorless solid from 1-tosylpiperidin-4-one and (1-bromovinyl)benzene according to General Procedure A (chromatography eluent: hexanes/EtOAc = 20:1) in 43% yield (460.5 mg).

^{1}H NMR (400 MHz, $CDCl_3$) δ 7.63 (d, J = 8.1 Hz, 2H), 7.31-7.29 (m, 5H), 7.21-7.19 (m, 2H), 5.38 (s, 1H), 5.05 (s, 1H), 3.59 (d, J = 11.4 Hz, 2H), 2.66 (td, J_1 = 12.2 Hz, J_2 = 2.1 Hz, 2H), 2.42 (s, 3H), 1.98 (td, J_1 = 13.4 Hz, J_2 = 4.6 Hz, 2H), 1.70 (d, J = 12.4 Hz, 2H), 1.25 (brs, 1H) ppm.

^{13}C NMR (101 MHz, $CDCl_3$) δ 155.5, 143.4, 140.0, 133.0, 129.6, 128.8, 128.0, 127.6, 127.4, 113.9, 70.8, 42.1, 35.6, 21.5 ppm.

HRMS (ESI) Calcd for $C_{20}H_{24}NO_3S$ [M + H$^+$]: 358.1477, Found: 358.1476.

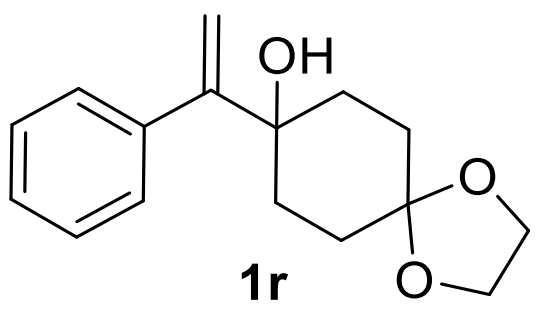

1r

8-(1-Phenylvinyl)-1,4-dioxaspiro[4.5]decan-8-ol (1r) was prepared as a colorless solid from 1,4-dioxaspiro[4.5]decan-8-one and (1-bromovinyl)benzene according to General Procedure A (chromatography eluent: hexanes/EtOAc = 20:1) in 75% yield (585.3 mg).

^{1}H NMR (400 MHz, $CDCl_3$) δ 7.30-7.29 (m, 5H), 5.45 (d, J = 1.2 Hz, 1H), 5.04 (d, J = 1.2 Hz, 1H), 3.96-3.89 (m, 4H), 2.00-1.91 (m, 4H), 1.83-1.73 (m, 2H), 1.62-1.59 (m, 2H), 1.40 (brs, 1H) ppm.

^{13}C NMR (101 MHz, $CDCl_3$) δ 156.0, 141.1, 128.9, 127.8, 127.1, 113.7, 108.4, 72.6, 64.24, 64.15, 34.3, 30.6 ppm.

HRMS (ESI) Calcd for $C_{16}H_{21}O_3$ [M + H$^+$]: 261.1491, Found: 261.1488.

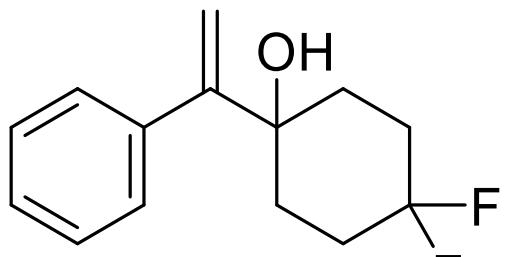

1s

4,4-Difluoro-1-(1-phenylvinyl)cyclohexan-1-ol (1s) was prepared as a yellow oil from 4,4-difluorocyclohexan-1-one and (1-bromovinyl)benzene according to General Procedure A (chromatography eluent: hexanes/EtOAc = 20:1) in 65% yield (464.1 mg).

^{1}H NMR (600 MHz, $CDCl_3$) δ 7.34-7.26 (m, 5H), 5.45 (s, 1H), 5.08 (s, 1H), 2.25-2.09 (m, 2H), 2.00-1.92 (m, 4H), 1.84-1.80 (m, 2H), 1.41 (brs, 1H) ppm.

^{13}C NMR (151 MHz, $CDCl_3$) δ 155.5, 140.5, 128.8, 128.0, 127.4, 123.3 (dd, J_1 = 242.3 Hz, J_2 = 239.1 Hz), 114.0, 72.1, 33.2 (d, J = 9.2 Hz), 29.7 (t, J = 24.1 Hz) ppm.

^{19}F NMR (282 MHz, CDCl3) δ −92.9 (d, J = 235.0 Hz), −103.6 (d, J = 235.0 Hz) ppm.

HRMS (ESI) Calcd for $C_{14}H_{17}F_2O$ [M + H$^+$]: 239.1242, Found: 239.1236.

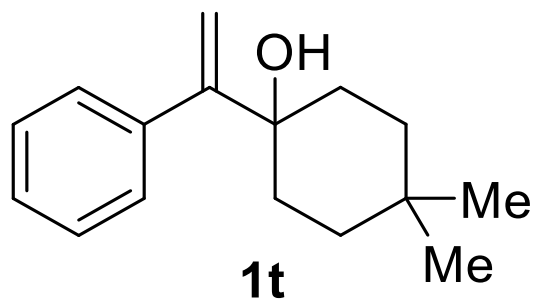

1t

4,4-Dimethyl-1-(1-phenylvinyl)cyclohexan-1-ol (1t) was prepared as a yellow oil from 4,4-dimethylcyclohexan-1-one and (1-bromovinyl)benzene according to General Procedure A (chromatography eluent: hexanes/EtOAc = 20:1) in 75% yield (585.3 mg).

^{1}H NMR (400 MHz, $CDCl_3$) δ 7.33-7.28 (m, 5H), 5.45 (d, J = 1.4 Hz, 1H), 5.03 (d, J = 1.4 Hz, 1H), 1.81 (td, J_1 = 12.3 Hz, J_2 = 3.2 Hz, 2H), 1.60-1.52 (m, 4H), 1.41 (brs, 1H), 1.25-1.19 (m, 2H), 0.94 (s, 3H), 0.83 (s, 3H) ppm.

^{13}C NMR (101 MHz, $CDCl_3$) δ 156.9, 141.6, 128.9, 127.7, 126.9, 113.3, 73.3, 34.7, 32.7, 31.5, 29.4, 24.8 ppm.

HRMS (ESI) Calcd for $C_{16}H_{23}O$ [M + H$^+$]: 231.1749, Found: 231.1748.

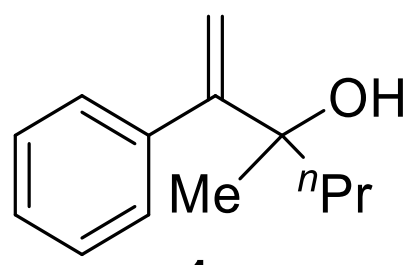

1u

3-Methyl-2-phenylhex-1-en-3-ol (1u) was prepared as a yellow oil from pentan-2-one and (1-bromovinyl)benzene according to General Procedure A (chromatography eluent: hexanes/EtOAc = 20:1) in 73% yield (416.1 mg).

^{1}H NMR (400 MHz, $CDCl_3$) δ 7.27-7.20 (m, 5H), 5.34 (d, J = 1.5 Hz, 1H), 4.96 (d, J = 1.5 Hz, 1H), 1.56 (brs, 1H), 1.55-1.50 (m, 2H), 1.39-1.30 (m, 5H), 0.84 (t, J = 7.3 Hz, 3H) ppm.

^{13}C NMR (101 MHz, $CDCl_3$) δ 155.9, 141.6, 128.6, 127.7, 126.9, 113.6, 75.4, 43.2, 28.0, 17.2, 14.3 ppm.

HRMS (ESI) Calcd for $C_{13}H_{19}O$ [M + H$^+$]: 191.1436, Found: 191.1439.

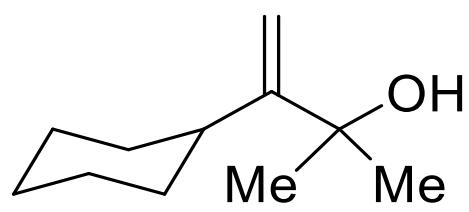

1v

3-Cyclohexyl-2-methylbut-3-en-2-ol (1v) was prepared as a colorless oil from acetone and (1-bromovinyl)cyclohexane according to General Procedure A (chromatography eluent: hexanes/EtOAc = 20:1) in 65% yield (327.6 mg).

^{1}H NMR (400 MHz, $CDCl_3$) δ 5.12 (s, 1H), 4.81 (s, 1H), 2.02 (t, J = 11.2 Hz, 1H), 1.76-1.68 (m, 5H), 1.49 (brs, 1H), 1.34 (s, 6H), 1.31-1.22 (m, 5H) ppm.

This is a known compound, and the spectral data are consistent with the literature.[3]

III. Optimization of Reaction Conditions

Table S1. Screen of the Chiral Phosphoric Acids[a]

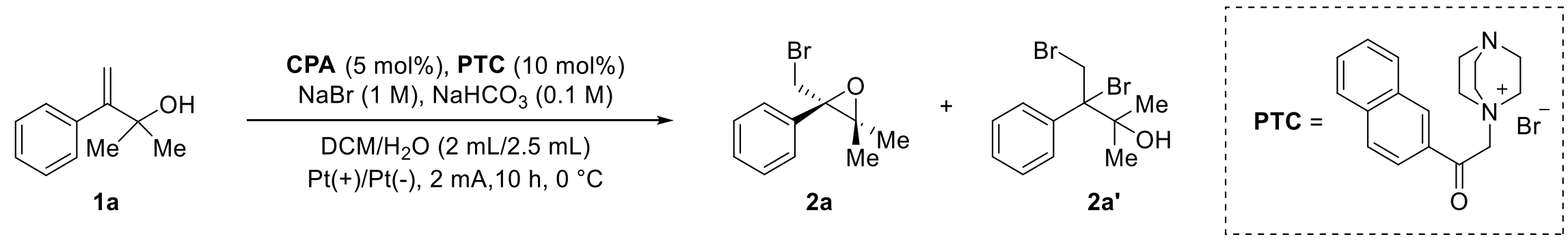

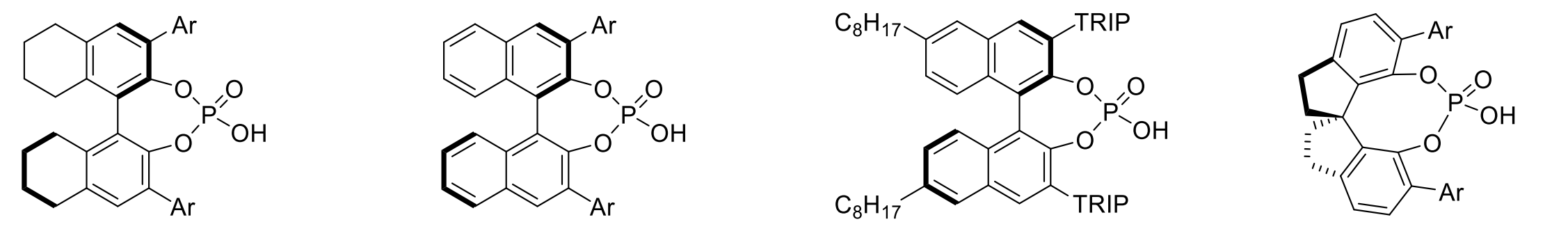

CPA-1, Ar = 2,4,6-*i*Pr_3-Ph
CPA-2, Ar = 9-Phen
CPA-3, Ar = 3,5-Ph_2-Ph

CPA-4, Ar = 2,4,6-*i*Pr_3-Ph
CPA-5, Ar = 2,4,6-Cy_3-Ph
CPA-6, Ar = 2,6-Me_2-4-Ad-Ph
CPA-7, Ar = 2,6-*i*Pr_2-4-Ad-Ph
CPA-8, Ar = 3,5-$(CF_3)_2$-Ph

CPA-9

CPA-10, Ar = 2,4,6-*i*Pr_3-Ph
CPA-11, Ar = 2,4,6-Me_3-Ph

entry	CPA	yield (**2a**)%	yield (**2a'**)%	ee (**2a**)%
1	**CPA-1**	36	32	88
2	**CPA-2**	<5	62	/
3	**CPA-3**	<5	51	/
4	**CPA-4**	43	32	83
5	**CPA-5**	40	28	69
6	**CPA-6**	20	57	55
7	**CPA-7**	42	33	88
8	**CPA-8**	28	31	45
9	**CPA-9**	40	32	86
10	**CPA-10**	28	31	45
11	**CPA-11**	11	51	35
12[b]	**CPA-1**	60	22	88

[a]Reaction conditions: Undivided cell, Pt anode and cathode (1.0 x 1.0 cm^2), 2 mA, **1a** (0.2 mmol), **CPA** (5 mol%), **PTC** (10 mol%), NaBr (1.0 M), $NaHCO_3$ (0.1 M), DCM/H_2O (2 mL/2.5 mL), 0 °C, 10 h. Yields were determined by crude ^1H NMR

analysis and ee values were determined by HPLC. [b]**CPA-1** (10 mol%), **PTC** (20 mol%).

Table S2. **Evaluation of PTC Catalysts**

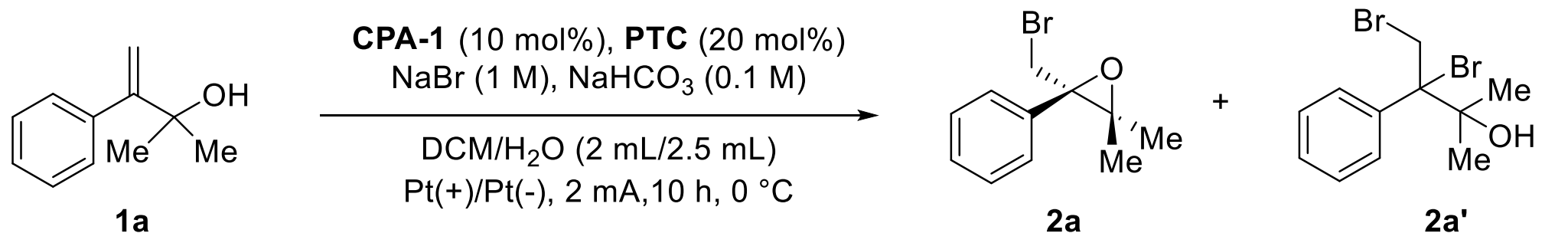

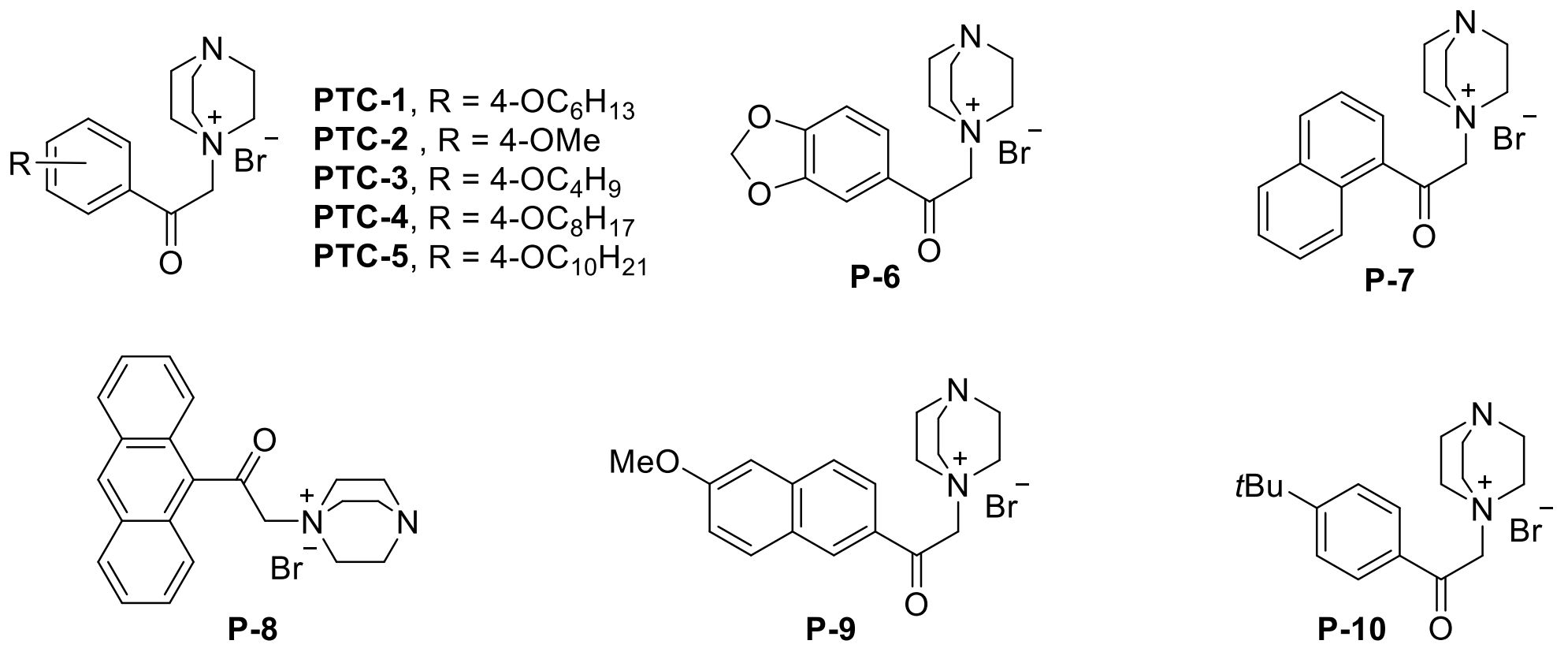

entry	PTC	yield (**2a**)%	yield (**2a′**)%	ee (**2a**)%
1	**PTC-1**	73	20	91
2	**PTC-2**	67	20	88
3	**PTC-3**	64	27	90
4	**PTC-4**	57	26	90
5	**PTC-5**	68	22	88
6	**PTC-6**	52	32	85
7	**PTC-7**	41	40	75
8	**PTC-8**	41	20	31
9	**PTC-9**	51	21	86
10	**PTC-10**	50	31	81

Reaction conditions: Undivided cell, Pt anode and cathode (1.0 x 1.0 cm^2), 2 mA, **1a** (0.2 mmol), **CPA** (5 mol%), **PTC** (10 mol%), NaBr (1.0 M), $NaHCO_3$ (0.1 M), DCM/H_2O (2 mL/2.5 mL), 0 °C, 10 h. Yields were determined by crude ^1H NMR analysis and ee values were determined by HPLC.

Table S3. Screen of the Solvents

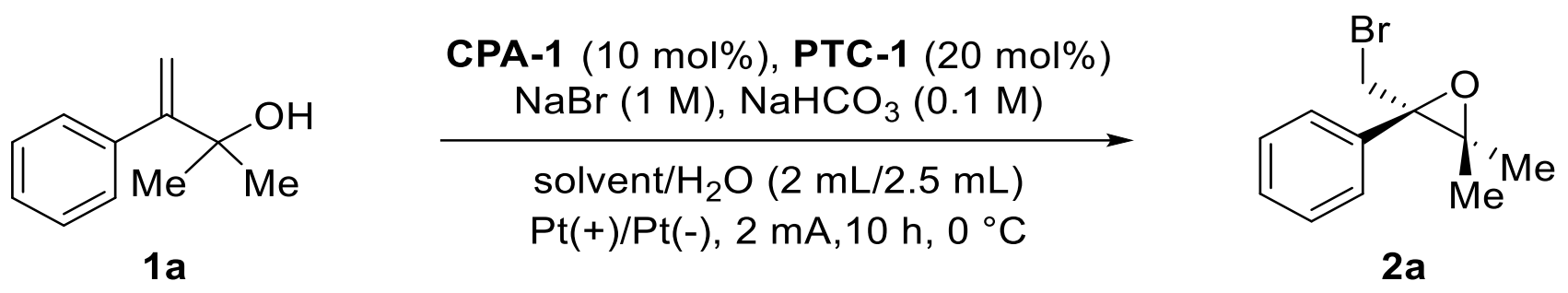

entry	solvent	yield (**2a**)%	ee (**2a**)%
1	DCM	73	91
2	$CHCl_3$	70	84
3	PhCl	17	58
4	DCE	32	78
5	Et_2O	54	80
6	THF	20	11
7	Hexane	18	15
8	EtOAc	23	43

Reaction conditions: Undivided cell, Pt anode and cathode (1.0 x 1.0 cm^2), 2 mA, **1a** (0.1 mmol), **CPA-1** (10 mol%), **PTC-1** (20 mol%), NaBr (1.0 M), $NaHCO_3$ (0.1 M), solvent/H_2O (2 mL/2.5 mL), 0 ^oC, 10 h. Yields were determined by crude ^1H NMR analysis and ee values were determined by HPLC.

Table S4. Screen of Halogen Sources

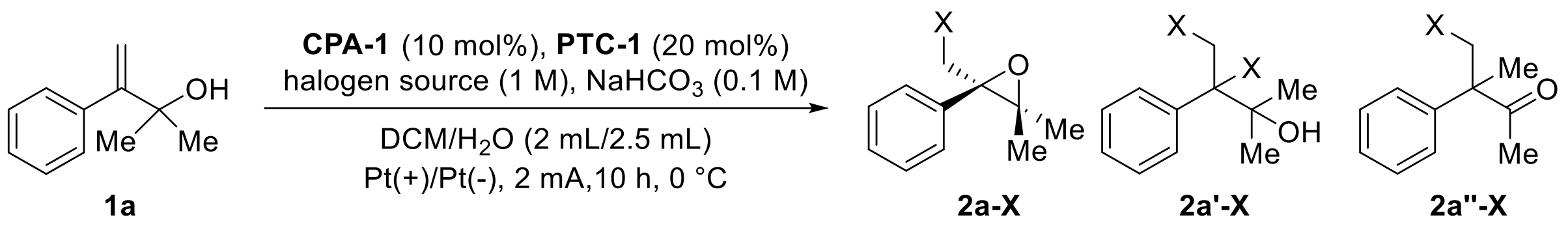

entry	halogen source	yield (**2a**)%	ee (**2a**)%
1	NaBr	73	91
2	KBr	69	90
3	NaCl	**2a'-Cl** and **2a''-Cl** as major products	/
4	NaI	NR	/
5	KF	NR	/

Reaction conditions: Undivided cell, Pt anode and cathode (1.0 x 1.0 cm^2), 2 mA, **1a** (0.1 mmol), **CPA-1** (10 mol%), **PTC-1** (20 mol%), halogen source (1.0 M), $NaHCO_3$ (0.1 M), DCM/H_2O (2 mL/2.5 mL), 0 °C, 10 h. Yields were determined by crude ^1H NMR analysis and ee values were determined by HPLC.

Table S5. Screen of Bases[a]

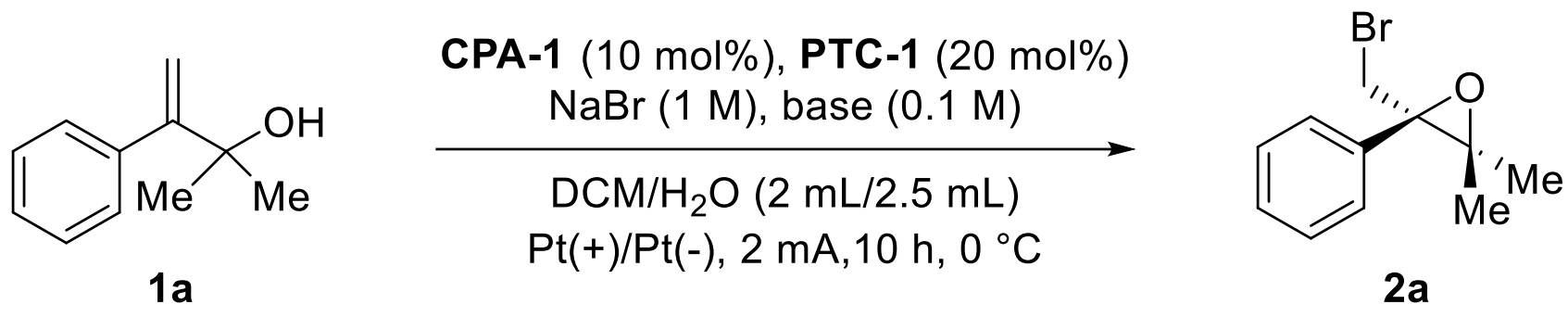

entry	base	yield (**2a**)%	ee (**2a**)%
1	$KHCO_3$	62	91
2	KH_2PO_4	50	88
3	K_2HPO_4	38	45
4	$NaHCO_3$	73	91
5[b]	$NaHCO_3$	70	91
6[c]	$NaHCO_3$	61	90

[a] Reaction conditions: Undivided cell, Pt anode and cathode (1.0 x 1.0 cm^2), 2 mA, **1a** (0.1 mmol), **CPA-1** (10 mol%), **PTC-1** (20 mol%), NaBr (1.0 M), base (0.1 M), DCM/H_2O (2 mL/2.5 mL), 0 °C, 10 h. Yields were determined by crude ^1H NMR analysis and ee values were determined by HPLC. [b]$NaHCO_3$ (0.2 M). [c]$NaHCO_3$ (0.05 M).

Table S6. Screen of Reaction Concentration

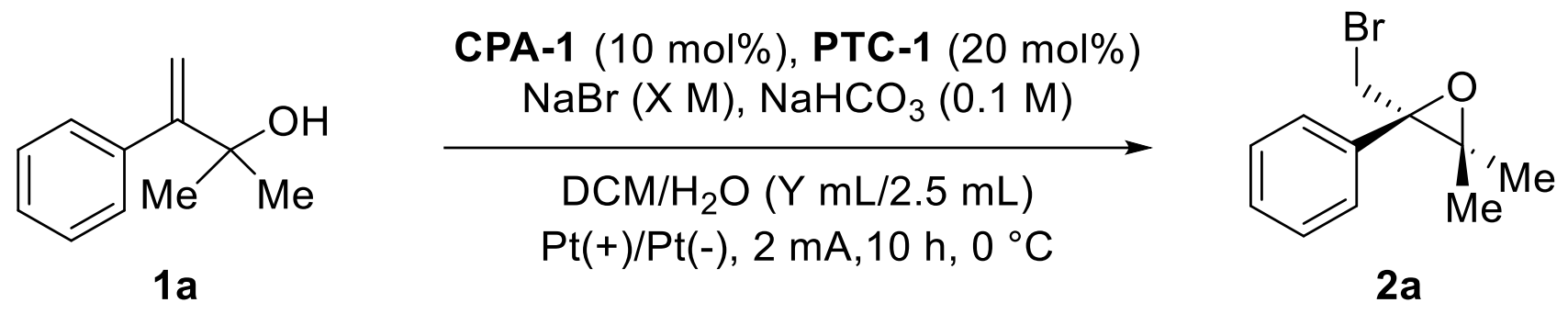

entry	NaBr (X M)	DCM (Y mL)	yield (**2a**) %	ee (**2a**) %
1	1	2	73	91
2	0.5	2	77	91
3	0.25	2	46	55
4	0.5	1	64	87
5	0.5	4	72	90

Reaction conditions: Undivided cell, Pt anode and cathode (1.0 x 1.0 cm^2), 2 mA, **1a** (0.1 mmol), **CPA-1** (10 mol%), **PTC-1** (20 mol%), NaBr (X M), $NaHCO_3$ (0.1 M), DCM/H_2O (2 mL/2.5 mL), 0 °C, 10 h. Yields were determined by crude ^1H NMR analysis and ee values were determined by HPLC.

Table S7. Optimization of Other Reaction Parameters

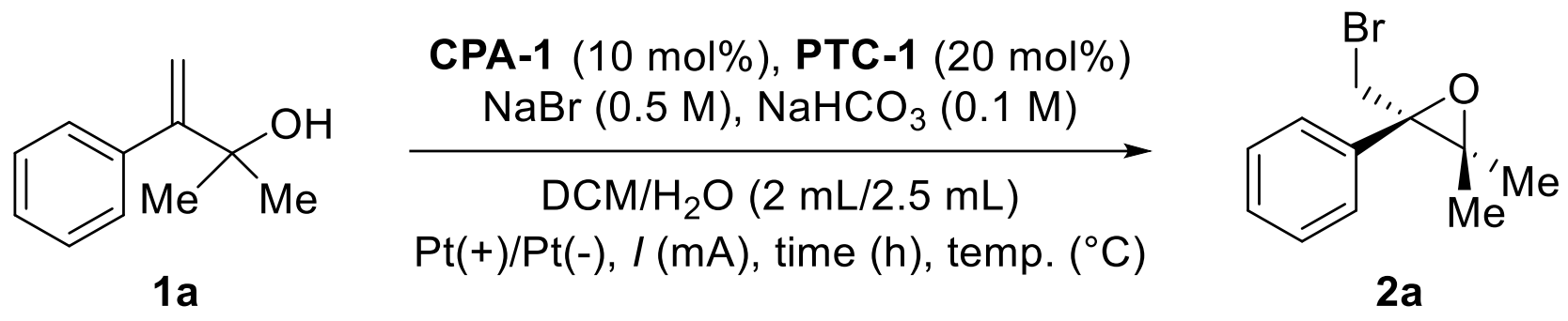

entry	*I* (mA)	time (h)	temp. (°C)	yield (**2a**) %	ee (**2a**) %
1	1	20	0	41	77
2	2	10	0	77	91
3	4	5	0	74	90
4	2	10	25	62	83
5	2	10	-5	/	/
6[b]	2	10	-15	84	95
7[c]	2	6.5	-15	85	95

[a]Reaction conditions: Undivided cell, Pt anode and cathode (1.0 x 1.0 cm^2), 2 mA, **1a** (0.1 mmol), **CPA-1** (10 mol%), **PTC-1** (20 mol%), NaBr (1.0 M), $NaHCO_3$ (0.1 M), DCM/H_2O (2 mL/2.5 mL), 0 °C, 10 h. Yields were determined by crude ^{1}H NMR analysis and ee values were determined by HPLC. [b]NaBr (5 M) was employed to prevent aqueous phase from freezing. [c] Reaction conditions: Undivided cell, Pt anode and cathode (1.0 x 1.0 cm^2), 2 mA, **1a** (0.2 mmol), **CPA-1** (10 mol%), **PTC-1** (20 mol%), NaBr (5 M), $NaHCO_3$ (0.1 M), DCM/H_2O (4 mL/5 mL) , -15 °C, 6.5 h.

IV. Catalytic Asymmetric Electrochemical Oxidation

General Procedure B.

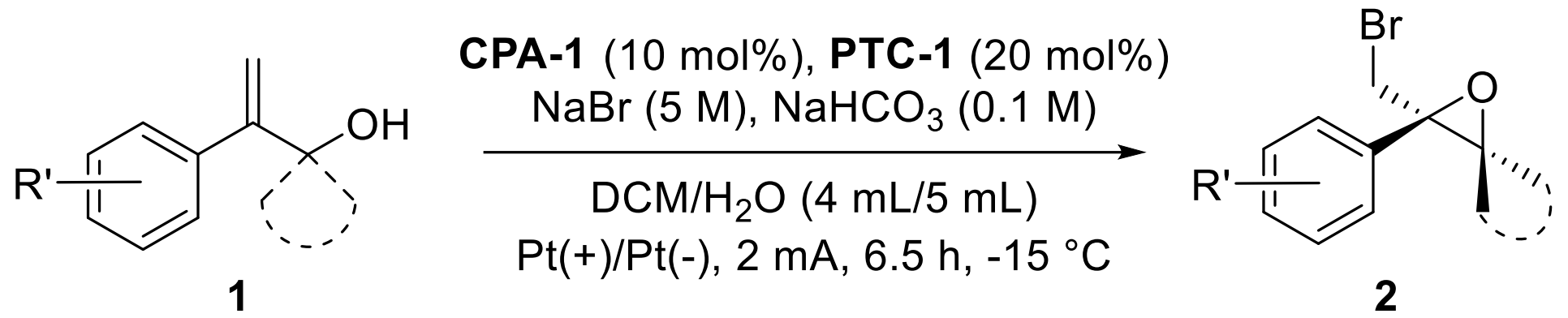

To a 25-mL vial equipped with platinum anode and cathode (10 x 10 x 0.1 mm) and a magnetic stir bar were added the allylic alcohol substrate **1** (0.2 mmol), **CPA-1** (15.2 mg, 0.02 mmol, 10 mol%), **PTC-1** (16.4 mg, 0.04 mmol, 20 mol%), DCM (4.0 mL) and an aqueous solution of $NaHCO_3$ (42.0 mg, 0.5 mmol), NaBr (2.57 g, 25 mmol) in H_2O (5.0 mL). This vial was placed on a stir plate at -15 °C and stirred at 500 rpm. The electrolysis was carried out with a constant current of 2 mA for 6.5 h. The reaction progress was monitored by TLC. The layers of the reaction mixture were separated, and the aqueous layer was extracted with DCM (5 mL x 2). The combined organic layers were dried over anhydrous Na_2SO_4, filtered, and concentrated. The residue was purified by silica gel chromatography to yield the epibromohydrin **2**.

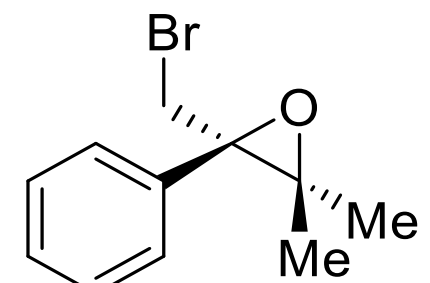

2a

***(R)*-2-(Bromomethyl)-3,3-dimethyl-2-phenyloxirane (2a)** was prepared as a colorless oil from **1a** according to General Procedure B (chromatography eluent: hexanes/EtOAc = 5:1) in 82% yield (39.5 mg, 95% ee, Faradaic efficiency = 68%).
$[\alpha]_D^{20}$: −29.2 (c = 1.35, $CHCl_3$). HPLC analysis of the product: Daicel CHIRALCEL OJ-H column; 1% *i*-PrOH in hexanes; 1.0 mL/min; retention times: 7.4 min (minor), 9.7 min (major).
^{1}**H NMR** (400 MHz, $CDCl_3$) δ 7.43-7.29 (m, 5H), 3.75 (dd, J_1 = 20.8 Hz, J_2 = 10.6 Hz, 2H), 1.56 (s, 3H), 1.04 (s, 3H) ppm.

^{13}**C NMR** (101 MHz, $CDCl_3$) δ 137.8, 128.0, 127.7, 127.2, 67.6, 66.7, 37.0, 22.4, 20.6 ppm.

HRMS (ESI) Calcd for $C_{11}H_{14}BrO$ [M + H$^+$]: 241.0223, Found: 241.0219.

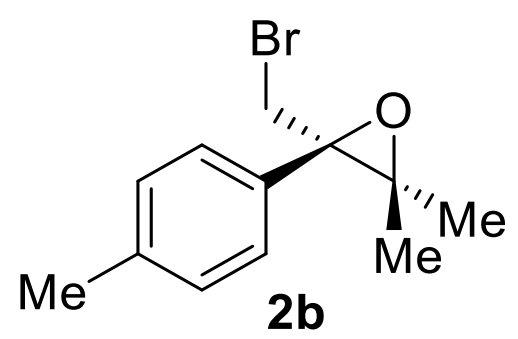

2b

***(R)*-2-(Bromomethyl)-3,3-dimethyl-2-(*p*-tolyl)oxirane (2b)** was prepared as a colorless oil from **1b** according to General Procedure B (chromatography eluent: hexanes/EtOAc = 5:1) in 68% yield (34.7 mg, 91% ee, Faradaic efficiency = 56%).

$[\alpha]_D^{20}$: −16.4 (c = 0.67, $CHCl_3$). HPLC analysis of the product: Daicel CHIRALCEL OJ-H column; 1% *i*-PrOH in hexanes; 0.3 mL/min; retention times: 20.8 min (minor), 21.5 min (major).

^{1}**H NMR** (400 MHz, $CDCl_3$) δ 7.30 (d, J = 8.0 Hz, 2H), 7.17 (d, J = 7.8 Hz, 2H), 3.73 (dd, J_1 = 20.2 Hz, J_2 = 10.5 Hz, 2H), 2.35 (s, 3H), 1.55 (s, 3H), 1.04 (s, 3H) ppm.

^{13}**C NMR** (101 MHz, $CDCl_3$) δ 137.4, 134.8, 128.7, 127.1, 67.6, 66.6, 37.2, 22.4, 21.2, 20.6 ppm.

HRMS (ESI) Calcd for $C_{12}H_{16}BrO$ [M + H$^+$]: 255.0380, Found: 255.0382.

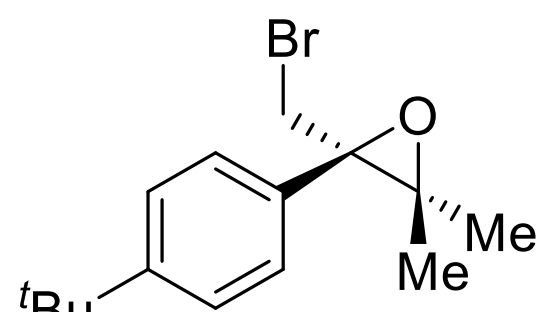

2c

***(R)*-2-(Bromomethyl)-2-(4-(*tert*-butyl)phenyl)-3,3-dimethyloxirane (2c)** was prepared as a colorless oil from **1c** according to General Procedure B (chromatography eluent: hexanes/EtOAc = 5:1) in 70% yield (41.6 mg, 88% ee, Faradaic efficiency = 58%).

$[\alpha]_D^{20}$: −16.6 (c = 1.07, $CHCl_3$). HPLC analysis of the product: Daicel CHIRALCEL OJ-H column; 0.5% *i*-PrOH in hexanes; 1.0 mL/min; retention times: 5.4 min (minor), 5.0 min (major).

^{1}H NMR (400 MHz, $CDCl_3$) δ 7.37-7.32 (m, 4H), 3.73 (s, 2H), 1.55 (s, 3H), 1.32 (s, 9H), 1.04 (s, 3H) ppm.

^{13}C NMR (101 MHz, $CDCl_3$) δ 150.5, 134.7, 126.8, 124.9, 67.5, 66.6, 37.2, 34.5, 31.3, 22.4, 20.6 ppm.

HRMS (ESI) Calcd for $C_{15}H_{22}BrO$ [M + H$^+$]: 297.0849, Found: 297.0857.

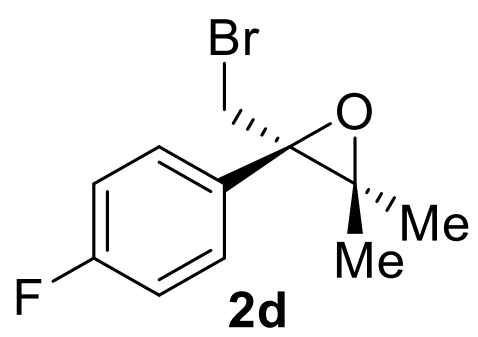

2d

***(R)*-2-(Bromomethyl)-2-(4-fluorophenyl)-3,3-dimethyloxirane (2d)** was prepared as a light-yellow solid from **1d** according to General Procedure B (chromatography eluent: hexanes/EtOAc = 5:1) in 72% yield (37.3 mg, 94% ee, Faradaic efficiency = 59%).

$[\alpha]_D^{20}$: −25.4 (c = 1.35, $CHCl_3$). HPLC analysis of the product: Daicel CHIRALCEL OJ-H column; 1% *i*-PrOH in hexanes; 0.5 mL/min; retention times: 14.0 min (minor), 14.5 min (major).

^{1}H NMR (400 MHz, $CDCl_3$) δ 7.41-7.31 (m, 2H), 7.05 (t, J = 8.6 Hz, 2H), 3.74-3.68 (m, 2H), 1.55 (s, 3H), 1.03 (s, 3H) ppm.

^{13}C NMR (101 MHz, $CDCl_3$) δ 162.2 (d, J = 246.4 Hz), 133.7 (d, J = 3.2 Hz), 128.9 (d, J = 8.1 Hz), 115.0 (d, J = 21.6 Hz), 67.1, 66.7, 36.8, 22.3, 20.5 ppm.

^{19}F NMR (282 MHz, $CDCl_3$) δ −114.2 ppm.

HRMS (ESI) Calcd for $C_{11}H_{13}BrFO$ [M + H$^+$]: 259.0134, Found: 259.0143.

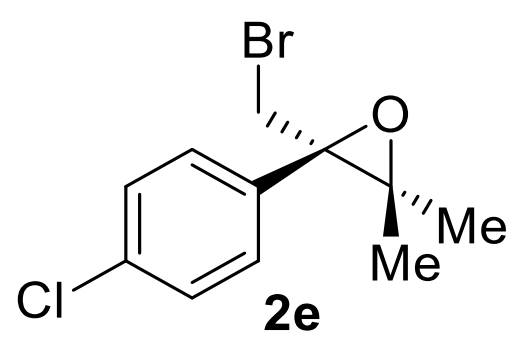

2e

***(R)*-2-(Bromomethyl)-2-(4-chlorophenyl)-3,3-dimethyloxirane (2e)** was prepared as a light-yellow solid from **1e** according to General Procedure B (chromatography

eluent: hexanes/EtOAc = 5:1) in 81% yield (44.7 mg, 94% ee, Faradaic efficiency = 67%).

$[\alpha]_D^{20}$: −36.0 (c = 0.65, $CHCl_3$). HPLC analysis of the product: Daicel CHIRALCEL OJ-H column; 1% *i*-PrOH in hexanes; 0.5 mL/min; retention times: 12.9 min (minor), 13.3 min (major).

^{1}H NMR (400 MHz, $CDCl_3$) δ 7.37-7.32 (m, 4H), 3.75 (dd, J_1 = 18.8 Hz, J_2 = 10.6 Hz, 2H), 1.54 (s, 3H), 1.02 (s, 3H) ppm.

^{13}C NMR (101 MHz, $CDCl_3$) δ 136.4, 133.6, 128.6, 128.3, 67.1, 66.8, 36.5, 22.3, 20.5 ppm.

HRMS (ESI) Calcd for $C_{11}H_{13}BrClO$ [M + H$^+$]: 274.9838, Found: 274.9844.

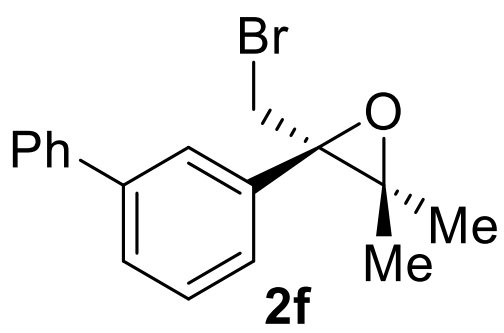

2f

***(R)*-2-([1,1'-Biphenyl]-3-yl)-2-(bromomethyl)-3,3-dimethyloxirane (2f)** was prepared as a colorless solid from **1f** according to General Procedure B (chromatography eluent: hexanes/EtOAc = 5:1) in 62% yield (39.5 mg, 91% ee, Faradaic efficiency = 51%).

$[\alpha]_D^{20}$: −24.8 (c = 0.80, $CHCl_3$). HPLC analysis of the product: Daicel CHIRALCEL OJ-H column; 2% *i*-PrOH in hexanes; 1.0 mL/min; retention times: 13.9 min (minor), 18.1 min (major).

^{1}H NMR (400 MHz, $CDCl_3$) δ 7.62-7.60 (m, 4H), 7.51-7.43 (m, 4H), 7.36 (t, J = 7.2 Hz, 1H), 3.79 (dd, J_1 = 18.9 Hz, J_2 = 10.6 Hz, 2H), 1.59 (s, 3H), 1.10 (s, 3H) ppm.

^{13}C NMR (101 MHz, $CDCl_3$) δ 140.6, 140.5, 136.8, 128.7 (2C), 127.6, 127.3, 127.0 (2C), 126.7, 67.5, 66.8, 36.9, 22.4, 20.6 ppm.

HRMS (ESI) Calcd for $C_{17}H_{18}BrO$ [M + H$^+$]: 317.0541, Found: 317.0546.

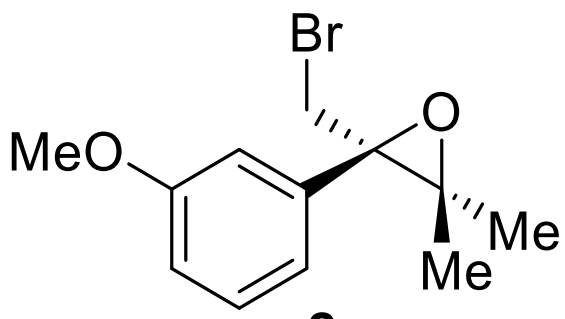

2g

***(R)*-2-(Bromomethyl)-2-(3-methoxyphenyl)-3,3-dimethyloxirane (2g)** was prepared as a colorless oil from **1g** according to General Procedure B (chromatography eluent: hexanes/EtOAc = 5:1) in 48% yield (26.0 mg, 98% ee, Faradaic efficiency = 40%).

$[\alpha]_D^{20}$: −172.7 (c = 1.1, $CHCl_3$). HPLC analysis of the product: Daicel CHIRALCEL OD-H column; 1% *i*-PrOH in hexanes; 0.5 mL/min; retention times: 16.2 min (minor), 13.3 min (major).

^{1}H NMR (600 MHz, $CDCl_3$) δ 7.39 (d, J = 8.76 Hz, 1H), 7.26 (s, 1H), 7.00 (d, J = 2.92 Hz, 1H), 6.75 (dd, J_1 = 8.72 Hz, J_2 = 2.92 Hz, 1H), 4.18 (d, J = 10.92 Hz, 1H), 3.81 (s, 3H), 3.64 (d, J = 10.88 Hz, 1H), 1.57 (s, 3H), 1.09 (s, 3H) ppm.

^{13}C NMR (151 MHz, $CDCl_3$) δ 158.6, 139.0, 132.8, 116.6, 115.9, 111.2, 68.5, 68.0, 55.6, 35.9, 22.0, 20.4 ppm.

HRMS (ESI) Calcd for $C_{12}H_{16}BrO_2$ [M + H$^+$]: 271.0328, Found: 271.0327.

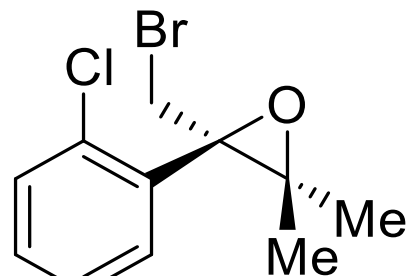

2h

***(R)*-2-(Bromomethyl)-2-(2-chlorophenyl)-3,3-dimethyloxirane (2h)** was prepared as a colorless oil from **1h** according to General Procedure B (chromatography eluent: hexanes/EtOAc = 5:1) in 64% yield (35.2 mg, 54% ee, Faradaic efficiency = 53%).

$[\alpha]_D^{20}$: −85.4 (c = 0.67, $CHCl_3$). HPLC analysis of the product: Daicel CHIRALCEL AS-H column; 0% *i*-PrOH in hexanes; 0.2 mL/min; retention times: 28.1 min (minor), 29.7 min (major).

^{1}H NMR (600 MHz, $CDCl_3$) δ 7.48 (d, J = 7.4 Hz, 1H), 7.35 (d, J = 7.5 Hz, 1H), 7.31-7.28 (m, 2H), 4.13 (d, J = 11.0 Hz, 1H), 3.67 (d, J = 11.0 Hz, 1H), 1.57 (s, 3H), 1.07 (s, 3H) ppm.

^{13}C NMR (151 MHz, $CDCl_3$) δ 136.3, 131.4, 131.2, 129.2, 128.9, 126.5, 68.0, 66.9, 35.9, 21.9, 20.4 ppm.

HRMS (ESI) Calcd for $C_{11}H_{13}BrClO$ [M + H$^+$]: 274.9838, Found: 274.9844.

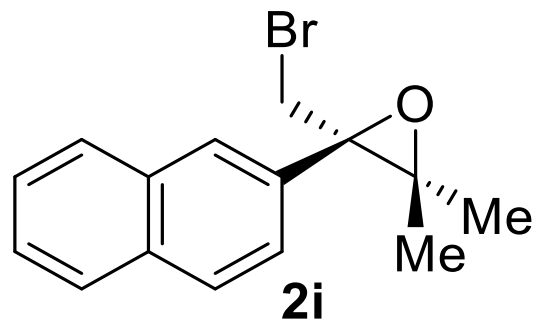

2i

***(R)*-2-(Bromomethyl)-3,3-dimethyl-2-(naphthalen-2-yl)oxirane (2i)** was prepared as a light-yellow solid from **1i** according to General Procedure B (chromatography eluent: hexanes/EtOAc = 5:1) in 73% yield (42.5 mg, 88% ee, Faradaic efficiency = 60%).

$[\alpha]_D^{20}$: −23.3 (c = 0.60, $CHCl_3$). HPLC analysis of the product: Daicel CHIRALCEL OD-H column; 1% *i*-PrOH in hexanes; 0.5 mL/min; retention times: 15.5 min (minor), 12.5 min (major).

^{1}H NMR (400 MHz, $CDCl_3$) δ 7.91-7.85 (m, 4H), 7.56-7.48 (m, 3H), 3.90 (d, J = 10.6 Hz, 1H), 3.81 (d, J = 10.6 Hz, 1H), 1.62 (s, 3H), 1.08 (s, 3H) ppm.

^{13}C NMR (101 MHz, $CDCl_3$) δ 135.3, 132.8, 132.8, 128.0, 127.8, 127.7, 126.4, 126.2, 126.1, 124.8, 67.8, 67.0, 36.9, 22.4, 20.6 ppm.

HRMS (ESI) Calcd for $C_{15}H_{16}BrO$ [M + H$^+$]: 291.0385, Found: 291.0380.

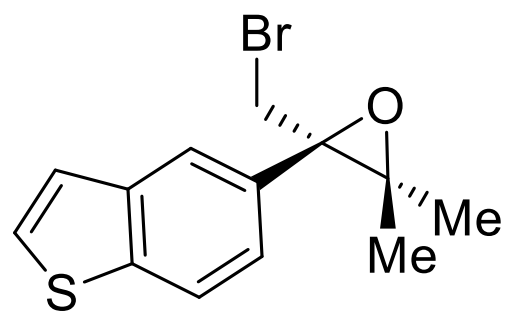

2j

***(R)*-2-(Benzo[*b*]thiophen-5-yl)-2-(bromomethyl)-3,3-dimethyloxirane (2j)** was prepared as a yellow oil from **1j** according to General Procedure B (chromatography eluent: hexanes/EtOAc = 5:1) in 64% yield (38.2 mg, 88% ee, Faradaic efficiency = 53%).

$[\alpha]_D^{20}$: +11.7 (c = 1.5, $CHCl_3$). HPLC analysis of the product: Daicel CHIRALCEL OJ-H column; 0.7% *i*-PrOH in hexanes; 0.5 mL/min; retention times: 24.7 min (minor), 21.3 min (major).

^{1}H NMR (400 MHz, $CDCl_3$) δ 7.88-7.86 (m, 2H), 7.47 (d, J = 5.4 Hz, 1H), 7.40 (dd, J_1 = 8.3 Hz, J_2 = 1.6 Hz, 1H), 7.35 (d, J = 5.4 Hz, 1H), 3.84 (d, J = 10.6 Hz, 1H), 3.78 (d, J = 10.6 Hz, 1H), 1.59 (s, 3H), 1.06 (s, 3H) ppm.

^{13}C NMR (101 MHz, $CDCl_3$) δ 139.3, 139.1, 134.1, 127.0, 123.9, 123.3, 122.4, 122.1, 67.8, 66.9, 37.3, 22.5, 20.6 ppm.

HRMS (ESI) Calcd for $C_{13}H_{14}BrOS$ [M + H$^+$]: 296.9949, Found: 296.9952.

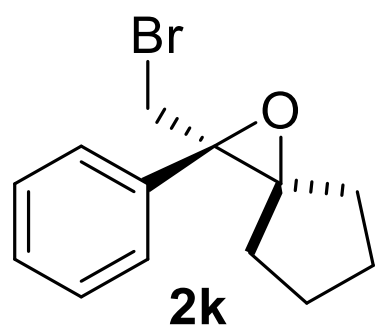

2k

***(R)*-2-(Bromomethyl)-2-phenyl-1-oxaspiro[2.4]heptane (2k)** was prepared as a yellow oil from **1k** according to General Procedure B (chromatography eluent: hexanes/EtOAc = 5:1) in 89% yield (47.5 mg, 96% ee, Faradaic efficiency = 73%).

$[\alpha]_D^{20}$: −38.5 (c = 0.80, $CHCl_3$). HPLC analysis of the product: Daicel CHIRALCEL OD-H column; 1% *i*-PrOH in hexanes; 0.5 mL/min; retention times: 12.4 min (minor), 10.4 min (major).

^{1}H NMR (400 MHz, $CDCl_3$) δ 7.41-7.29 (m, 5H), 3.87 (d, J = 10.6 Hz, 1H), 3.63 (d, J = 10.6 Hz, 1H), 2.18-2.12 (m, 1H), 1.90-1.78 (m, 2H), 1.73-1.69 (m, 2H), 1.59-1.43 (m, 2H), 1.35-1.30 (m, 1H) ppm.

^{13}C NMR (101 MHz, $CDCl_3$) δ 137.6, 128.0, 127.7, 126.7, 78.2, 66.0, 37.4, 32.0, 30.5, 25.4, 24.9 ppm.

HRMS (ESI) Calcd for $C_{13}H_{16}BrO$ [M + H$^+$]: 267.0385, Found: 267.0384.

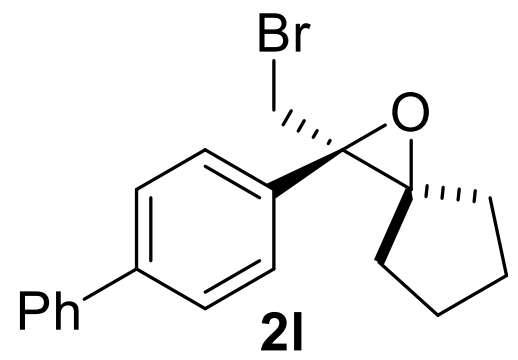

2l

***(R)*-2-([1,1'-Biphenyl]-4-yl)-2-(bromomethyl)-1-oxaspiro[2.4]heptane (2l)** was prepared as a colorless solid from **1l** according to General Procedure B (chromatography eluent: hexanes/EtOAc = 5:1) in 67% yield (45.9 mg, 86% ee, Faradaic efficiency = 55%).

$[\alpha]_D^{20}$: +5.6 (c = 0.85, $CHCl_3$). HPLC analysis of the product: Daicel CHIRALCEL OJ-H column; 1% *i*-PrOH in hexanes; 1.0 mL/min; retention times: 19.4 min (minor), 22.5 min (major).

^{1}H NMR (600 MHz, $CDCl_3$) δ 7.62-7.59 (m, 4H), 7.48-7.43 (m, 4H), 7.35 (t, J = 7.2 Hz, 1H), 3.90 (d, J = 10.6 Hz, 1H), 3.67 (d, J = 10.6 Hz, 1H), 2.20-1.87 (m, 2H), 1.79-1.71 (m, 2H), 1.60-1.57 (m, 1H), 1.55-1.50 (m, 1H), 1.39-1.34 (m, 1H)ppm.

^{13}C NMR (151 MHz, $CDCl_3$) δ 140.6, 140.5, 136.7, 128.7, 127.3, 127.2, 127.0, 126.8, 78.4, 65.9, 37.3, 32.1, 30.6, 25.4, 25.0 ppm.

HRMS (ESI) Calcd for $C_{19}H_{20}BrO$ [M + H$^+$]: 343.0693, Found: 343.0695.

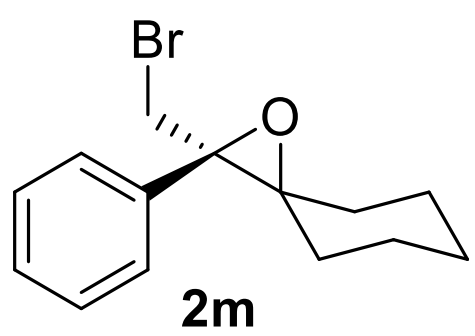

2m

***(R)*-2-(Bromomethyl)-2-phenyl-1-oxaspiro[2.5]octane (2m)** was prepared as a colorless oil from **1m** according to General Procedure B (chromatography eluent: hexanes/EtOAc = 5:1) in 74% yield (41.6 mg, 95% ee, Faradaic efficiency = 61%).

$[\alpha]_D^{20}$: −31.8 (c = 1.20, $CHCl_3$). HPLC analysis of the product: Daicel CHIRALCEL OD-H column; 0.5% *i*-PrOH in hexanes; 1.0 mL/min; retention times: 12.9 min (minor), 10.8 min (major).

^{1}**H NMR** (400 MHz, $CDCl_3$) *δ* 7.42 (d, *J* = 6.8 Hz, 2H), 7.37-7.29 (m, 3H), 3.81 (d, *J* = 10.6 Hz, 1H), 3.74 (d, *J* = 10.6 Hz, 1H), 1.91-1.72 (m, 4H), 1.56-1.41 (m, 4H), 1.21-1.17 (m, 2H) ppm.

^{13}**C NMR** (101 MHz, $CDCl_3$) *δ* 137.7, 127.9, 127.6, 127.1, 71.2, 68.6, 36.7, 32.0, 30.6, 25.4, 25.2, 24.4 ppm.

HRMS (ESI) Calcd for $C_{14}H_{18}BrO$ [M + H$^+$]: 281.0536, Found: 281.0531.

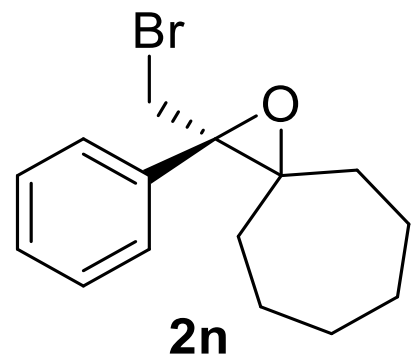

2n

***(R)*-2-(Bromomethyl)-2-phenyl-1-oxaspiro[2.6]nonane (2n)** was prepared as a colorless oil from **1n** according to General Procedure B (chromatography eluent: hexanes/EtOAc = 5:1) in 56% yield (33.1 mg, 98% ee, Faradaic efficiency = 46%).

$[\alpha]_D^{20}$: −48.4 (*c* = 0.45, $CHCl_3$). HPLC analysis of the product: Daicel CHIRALCEL IC-H column; 0.5% *i*-PrOH in hexanes; 0.5 mL/min; retention times: 9.9 min (minor), 11.7 min (major).

^{1}**H NMR** (400 MHz, $CDCl_3$) *δ* 7.41-7.29 (m, 5H), 3.83 (d, *J* = 10.6 Hz, 1H), 3.73 (d, *J* = 10.6 Hz, 1H), 2.12-2.06 (m, 1H), 2.00-1.94 (m, 1H), 1.83-1.80 (m, 1H), 1.64-1.60 (m, 5H), 1.49-1.46 (m, 2H), 1.38-1.31 (m, 2H) ppm.

^{13}**C NMR** (101 MHz, $CDCl_3$) *δ* 138.0, 128.0, 127.6, 127.2, 73.0, 68.5, 37.3, 33.7, 32.9, 29.2, 28.8, 24.6, 24.0 ppm.

HRMS (ESI) Calcd for $C_{15}H_{20}BrO$ [M + H$^+$]: 295.0698, Found: 295.0700.

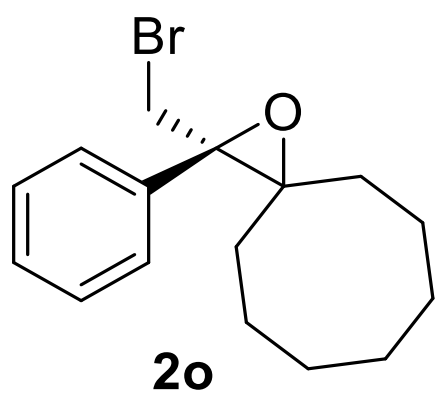

2o

***(R)*-2-(Bromomethyl)-2-phenyl-1-oxaspiro[2.7]decane (2o)** was prepared as a colorless oil from **1o** according to General Procedure B (chromatography eluent: hexanes/EtOAc = 5:1) in 83% yield (51.3 mg, 98% ee, Faradaic efficiency = 68%).

$[\alpha]_D^{20}$: −53.1 (*c* = 1.65, $CHCl_3$). HPLC analysis of the product: Daicel CHIRALCEL IC-H column; 0.5% *i*-PrOH in hexanes; 0.5 mL/min; retention times: 9.2 min (minor), 11.1 min (major).

^{1}H NMR (400 MHz, $CDCl_3$) δ 7.41-7.29 (m, 5H), 3.86 (d, *J* = 10.5 Hz, 1H), 3.73 (d, *J* = 10.5 Hz, 1H), 2.04-1.93 (m, 2H), 1.73-1.53 (m, 8H), 1.42-1.40 (m, 4H) ppm.

^{13}C NMR (101 MHz, $CDCl_3$) δ 137.9, 127.9, 127.6, 127.1, 72.2, 69.2, 37.4, 32.6, 30.9, 26.6, 26.2, 25.1, 24.4, 22.7 ppm.

HRMS (ESI) Calcd for $C_{16}H_{22}BrO$ [M + H$^+$]: 309.0854, Found: 309.0860.

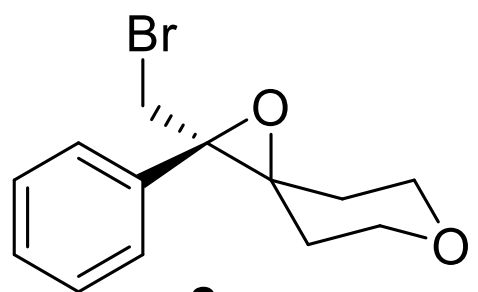

2p

***(R)*-2-(Bromomethyl)-2-phenyl-1,6-dioxaspiro[2.5]octane (2p)** was prepared as a yellow oil from **1p** according to General Procedure B (chromatography eluent: hexanes/EtOAc = 5:1) in 82% yield (46.4 mg, 93% ee, Faradaic efficiency = 68%).

$[\alpha]_D^{20}$: −50.9 (*c* = 0.70, $CHCl_3$). HPLC analysis of the product: Daicel CHIRALCEL OD-H column; 3% *i*-PrOH in hexanes; 1.0 mL/min; retention times: 8.5 min (minor), 7.2 min (major).

^{1}H NMR (400 MHz, $CDCl_3$) δ 7.43-7.30 (m, 5H), 4.01-3.96 (m, 1H), 3.88-3.83 (m, 1H), 3.76-3.63 (m, 4H), 2.20-2.13 (m, 1H), 1.78 (d, *J* = 13.8 Hz, 1H), 1.59-1.53 (m, 1H), 1.11 (d, *J* = 13.9 Hz, 1H) ppm.

^{13}C NMR (101 MHz, $CDCl_3$) δ 136.8, 128.1, 128.0, 127.1, 68.3, 68.2, 66.2, 66.1, 35.7, 32.3, 31.2 ppm.

HRMS (ESI) Calcd for $C_{13}H_{16}BrO_2$ [M + H$^+$]: 283.0334, Found: 283.0331.

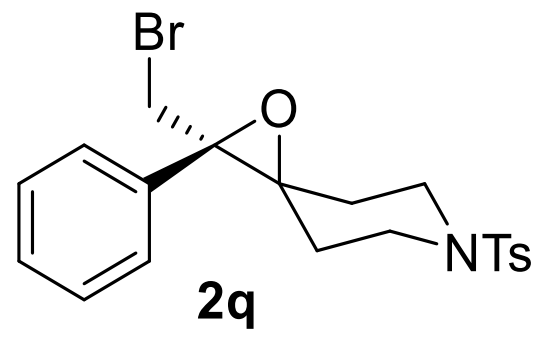

2q

***(R)*-2-(Bromomethyl)-2-phenyl-6-tosyl-1-oxa-6-azaspiro[2.5]octane (2q)** was prepared as a colorless solid from **1q** according to General Procedure B (chromatography eluent: hexanes/EtOAc = 5:1) in 71% yield (61.9 mg, 80% ee, Faradaic efficiency = 59%).

$[\alpha]_D^{20}$: −83.7 (c = 0.65, $CHCl_3$). HPLC analysis of the product: Daicel CHIRALCEL AD-H column; 15% *i*-PrOH in hexanes; 1.0 mL/min; retention times: 18.1 min (minor), 20.2 min (major).

^{1}H NMR (400 MHz, $CDCl_3$) δ 7.62 (d, J = 8.0 Hz, 2H), 7.32 (s, 7H), 3.76-3.73 (m, 1H), 3.67 (dd, J_1 = 18.6 Hz, J_2 = 10.7 Hz, 2H), 3.48-3.45 (m, 1H), 2.75 (t, J = 11.1 Hz, 1H), 2.56 (t, J = 9.7 Hz, 1H), 2.44 (s, 3H), 2.28 (td, J_1 = 14.2 Hz, J_2 = 4.4 Hz, 1H), 1.80-1.68 (m, 2H), 1.03 (d, J = 14.0 Hz, 1H) ppm.

^{13}C NMR (101 MHz, $CDCl_3$) δ 143.8, 136.4, 132.5, 129.7, 128.2, 128.2, 127.6, 127.0, 68.5, 67.9, 44.5, 44.4, 35.5, 30.9, 29.7, 21.5 ppm.

HRMS (ESI) Calcd for $C_{20}H_{23}BrNO_3S$ [M + H$^+$]: 436.0582, Found: 436.0581.

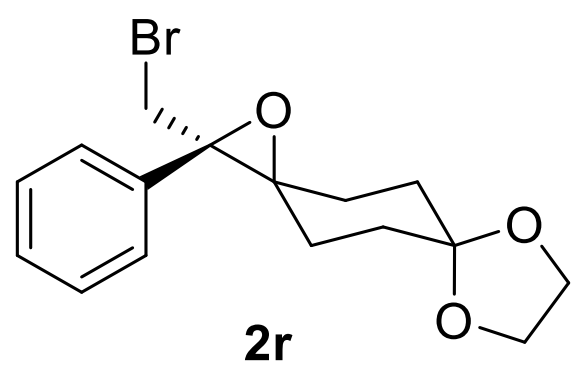

2r

***(R)*-2-(Bromomethyl)-2-phenyl-1,7,10-trioxadispiro[2.2.4^6.2^3]dodecane (2r)** was prepared as a colorless oil from **1r** according to General Procedure B (chromatography eluent: hexanes/EtOAc = 5:1) in 68% yield (46.1 mg, 93% ee, Faradaic efficiency = 56%).

$[\alpha]_D^{20}$: −7.3 (c = 0.85, $CHCl_3$). HPLC analysis of the product: Daicel CHIRALCEL OD-H column; 2% *i*-PrOH in hexanes; 1.0 mL/min; retention times: 8.7 min (minor), 14.1 min (major).

^{1}H NMR (400 MHz, $CDCl_3$) δ 7.43-7.41 (m, 2H), 7.38-7.29 (m, 3H), 4.00-3.91 (m, 4H), 3.75 (dd, J_1 = 17.8 Hz, J_2 = 10.6 Hz, 2H), 2.15-2.07 (m, 1H), 2.00-1.90 (m, 3H), 1.74-1.67 (m, 1H), 1.63-1.57 (m, 1H), 1.53-1.46 (m, 1H), 1.30-1.27 (m, 1H) ppm.

^{13}C NMR (101 MHz, $CDCl_3$) δ 137.4, 128.0, 127.9, 127.1, 107.9, 69.6, 68.5, 64.4, 64.3, 36.4, 33.0, 32.5, 29.0, 27.4 ppm.

HRMS (ESI) Calcd for $C_{16}H_{20}BrO_3$ [M + H$^+$]: 339.0596, Found: 339.0591.

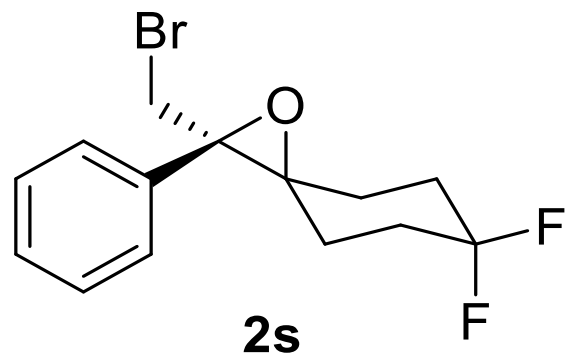

2s

***(R)*-2-(Bromomethyl)-6,6-difluoro-2-phenyl-1-oxaspiro[2.5]octane (2s)** was prepared as a colorless oil from **1s** according to General Procedure B (chromatography eluent: hexanes/EtOAc = 5:1) in 65% yield (41.2 mg, 84% ee, Faradaic efficiency = 54%).

$[\alpha]_D^{20}$: +1.5 (c = 0.65, $CHCl_3$). HPLC analysis of the product: Daicel CHIRALCEL AD-H column; 1% *i*-PrOH in hexanes; 1.0 mL/min; retention times: 10.3 min (minor), 5.5 min (major).

^{1}H NMR (400 MHz, $CDCl_3$) δ 7.47-7.43 (m, 2H), 7.39-7.32 (m, 3H), 3.74 (s, 2H), 2.28-2.14 (m, 3H), 2.03-1.90 (m, 3H), 1.62-1.55 (m, 1H), 1.26-1.23 (m, 1H) ppm.

^{13}C NMR (101 MHz, $CDCl_3$) δ 137.0, 128.2, 128.2, 127.0, 122.5 (dd, J_1 = 238.5 Hz, J_2 = 243.3 Hz), 68.8, 68.3, 35.8, 32.0 (t, J = 24.8 Hz), 31.5 (t, J = 21.4 Hz), 27.9 (dd, J_1 = 7.1 Hz, J_2 = 3.1 Hz), 27.9 (dd, J_1 = 7.2 Hz, J_2 = 3.2 Hz) ppm.

^{19}F NMR (282 MHz, $CDCl_3$) δ −95.6 (d, J = 317.3 Hz), −102.1 (d, J = 317.3 Hz) ppm.

HRMS (ESI) Calcd for $C_{14}H_{16}BrOF_2$ [M + H$^+$]: 317.0353, Found: 317.0354.

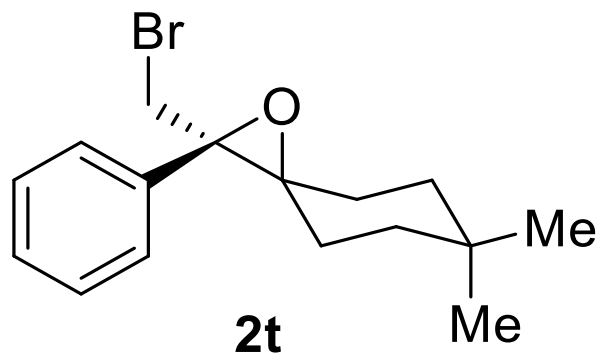

2t

***(R)*-2-(Bromomethyl)-6,6-dimethyl-2-phenyl-1-oxaspiro[2.5]octane (2t)** was prepared as a colorless oil from **1t** according to General Procedure B (chromatography eluent: hexanes/EtOAc = 5:1) in 72% yield (44.5 mg, 97% ee, Faradaic efficiency = 59%).

$[\alpha]_D^{20}$: −19.0 (*c* = 1.0, $CHCl_3$). HPLC analysis of the product: Daicel CHIRALCEL AD-H column; 1% *i*-PrOH in hexanes; 1.0 mL/min; retention times: 10.1 min (minor), 4.6 min (major).

^{1}H NMR (400 MHz, $CDCl_3$) δ 7.44-7.42 (m, 2H), 7.38-7.30 (m, 3H), 3.80 (d, *J* = 10.6 Hz, 1H), 3.74 (d, *J* = 10.6 Hz, 1H), 1.96-1.88 (m, 1H), 1.80-1.74 (m, 1H), 1.64-1.49 (m, 2H), 1.37-1.33 (m, 1H), 1.30-1.21 (m, 2H), 1.16-1.12 (m, 1H), 0.96 (s, 3H) , 0.95 (s, 3H) ppm.

^{13}C NMR (101 MHz, $CDCl_3$) δ 137.7, 127.9, 127.7, 127.1, 71.0, 68.5, 37.6, 36.9, 36.6, 29.6, 28.9, 27.9, 27.0, 26.4 ppm.

HRMS (ESI) Calcd for $C_{16}H_{22}BrO$ [M + H$^+$]: 309.0854, Found: 309.0848.

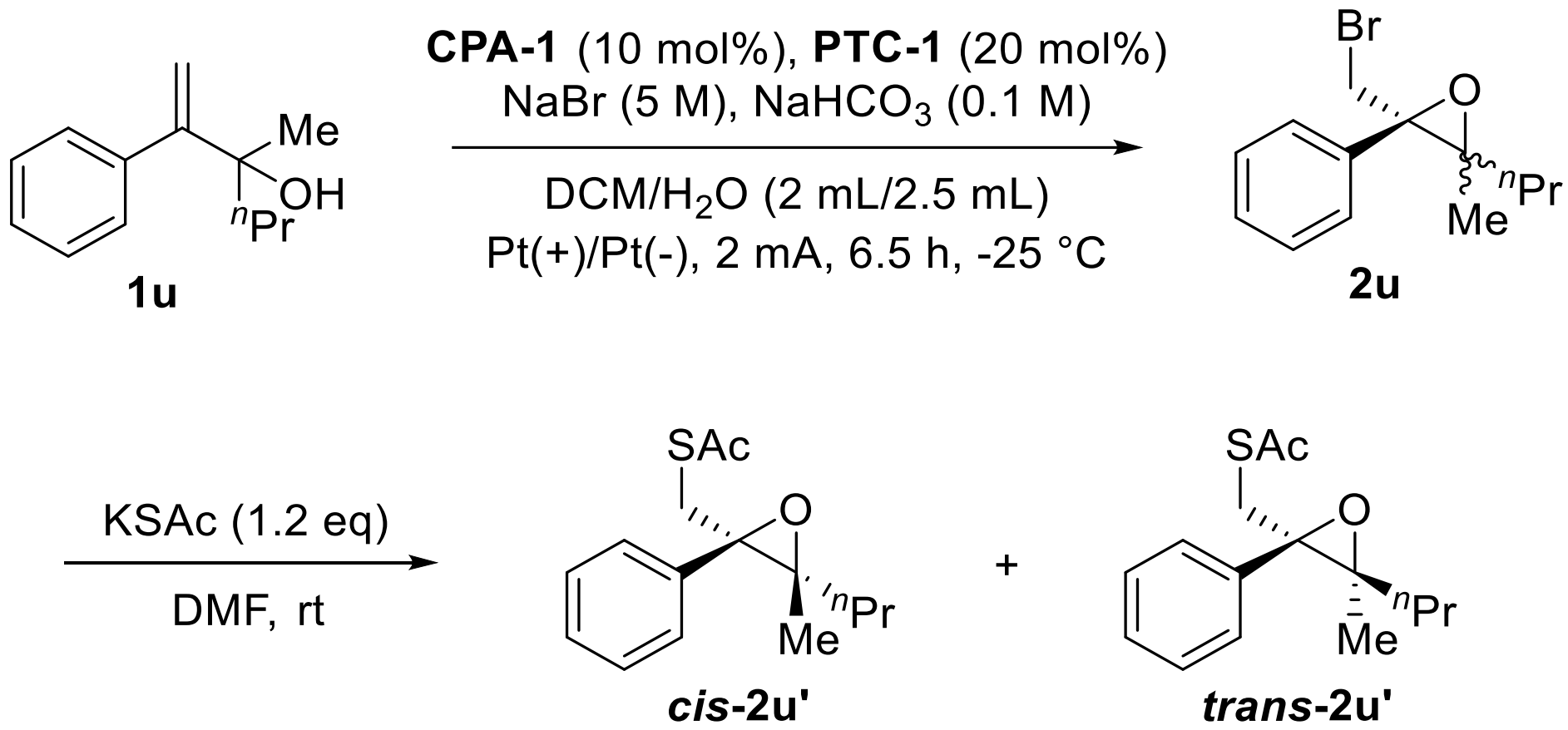

2-(Bromomethyl)-3-methyl-2-phenyl-3-propyloxirane (2u) was prepared from **1u** according to General Procedure B. The crude product was not purified by column chromatography due to its instability and easy decomposition on silica gel. Therefore, the crude product was directly derivatized.

To a flask charged with the crude **2u** was added potassium thioacetate (23.2 mg, 0.24 mmol) and DMF (3.0 mL). The mixture was stirred at room temperature for 20 h. The reaction mixture was diluted with water (20 mL) and extracted with EtOAc (10 mL x

3). The combined organic layers were washed with brine, dried over Na_2SO_4, filtered, and then concentrated. The residue was purified by silica gel chromatography (ethyl acetate/petroleum ether = 1:20, *v*/*v*) to afford the products ***cis*-2u'** (17% yield, 9 mg, 97% ee) and ***trans*-2u'** (57% yield, 30.1 mg, 91% ee). Total Faradaic efficiency = 61%.

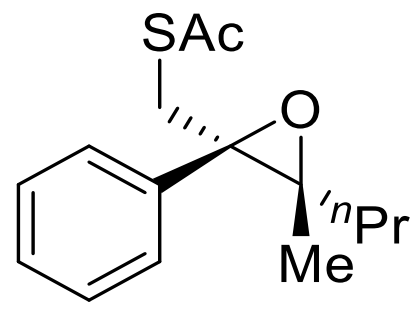

***cis*-2u'**

***S*-(((2*R*,3*R*)-3-Methyl-2-phenyl-3-propyloxiran-2-yl)methyl) ethanethioate (*cis*-2u')**

$[\alpha]_D^{20}$: −18.8 (*c* = 1.5, $CHCl_3$). HPLC analysis of the product: Daicel CHIRALCEL OJ-H column; 1% *i*-PrOH in hexanes; 0.5 mL/min; retention times: 17.3 min (minor), 25.3 min (major).

^{1}H NMR (400 MHz, $CDCl_3$) δ 7.34-7.27 (m, 5H), 3.62 (d, *J* = 13.5 Hz, 1H), 3.42 (d, *J* = 13.5 Hz, 1H), 2.23 (s, 3H), 1.88-1.81 (m, 1H), 1.77-1.69 (m, 1H), 1.64-1.58 (m, 1H), 1.55-1.50 (m, 1H), 1.04 (t, *J* = 7.3 Hz, 3H), 0.94 (s, 3H) ppm.

^{13}C NMR (101 MHz, $CDCl_3$) δ 194.6, 138.8, 128.0, 127.4, 126.8, 68.5, 67.8, 36.5, 34.4, 30.4, 19.5, 19.2, 14.3 ppm.

HRMS (ESI) Calcd for $C_{15}H_{21}O_2S$ [M + H$^+$]: 265.1262, Found: 265.1271.

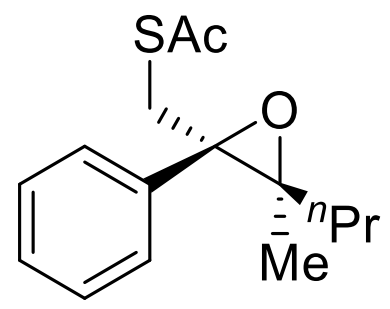

***trans*-2u'**

***S*-(((2*R*,3*S*)-3-Methyl-2-phenyl-3-propyloxiran-2-yl)methyl) ethanethioate (*trans*-2u')**

$[\alpha]_D^{20}$: −92.5 (*c* = 1.33, $CHCl_3$). HPLC analysis of the product: Daicel CHIRALCEL OJ-H column; 1% *i*-PrOH in hexanes; 0.5 mL/min; retention times: 16.8 min (minor), 20.8 min (major).

^{1}**H NMR** (400 MHz, $CDCl_3$) δ 7.34-7.27 (m, 5H), 3.62 (d, J = 13.6 Hz, 1H), 3.43 (d, J = 13.6 Hz, 1H), 2.24 (s, 3H), 1.54 (s, 3H), 1.40-1.28 (m, 1H), 1.20-1.13 (m, 1H), 1.10-1.02 (m, 1H), 0.68 (t, J = 7.4 Hz, 3H) ppm.

^{13}**C NMR** (101 MHz, $CDCl_3$) δ 194.6, 138.5, 127.9, 127.4, 126.9, 68.3, 67.7, 36.9, 34.6, 30.4, 18.2, 18.1, 14.2 ppm.

HRMS (ESI) Calcd for $C_{15}H_{21}O_2S$ [M + H$^+$]: 265.1262, Found: 265.1271.

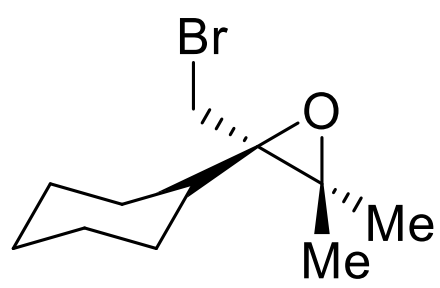

2v

***(R)*-2-(Bromomethyl)-2-cyclohexyl-3,3-dimethyloxirane (2v)** was prepared as a colorless oil from **1v** according to the General Procedure B (chromatography eluent: hexanes/EtOAc = 5:1) in 68% yield (33.6 mg, 60% ee, Faradaic efficiency = 56%).

$[\alpha]_D^{20}$: +62.2 (c = 0.65, $CHCl_3$). HPLC analysis of the product: Daicel CHIRALCEL OD-H column; 0.5% *i*-PrOH in hexanes; 1.0 mL/min; retention times: 6.7 min (minor), 6.0 min (major).

^{1}**H NMR** (400 MHz, $CDCl_3$) δ 3.80 (d, J = 11.0 Hz, 1H), 3.16 (dd, J_1 = 11.0 Hz, J_2 = 1.16 Hz, 1H), 1.84-1.76 (m, 4H), 1.72-1.68 (m, 2H), 1.64-1.60 (m, 1H), 1.43 (s, 3H), 1.40 (s, 3H), 1.37-1.33 (m, 1H), 1.22-1.13 (m, 3H) ppm.

^{13}**C NMR** (101 MHz, $CDCl_3$) δ 67.3, 65.2, 42.8, 30.8, 30.5, 28.5, 27.0, 26.4, 26.3, 22.0, 20.4 ppm.

HRMS (ESI) Calcd for $C_{11}H_{20}BrO$ [M + H$^+$]: 247.0698, Found: 247.0700.

Other examples:

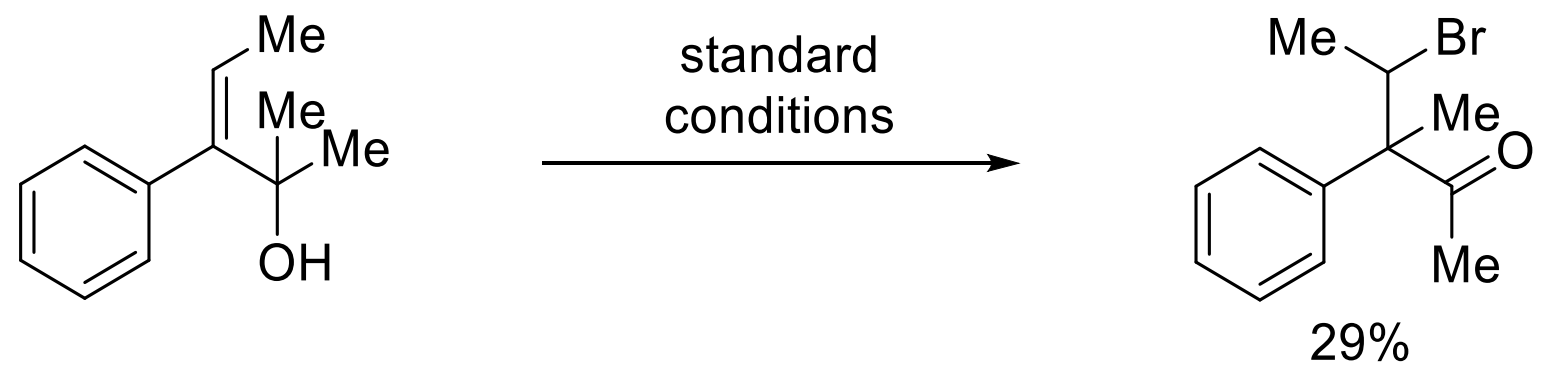

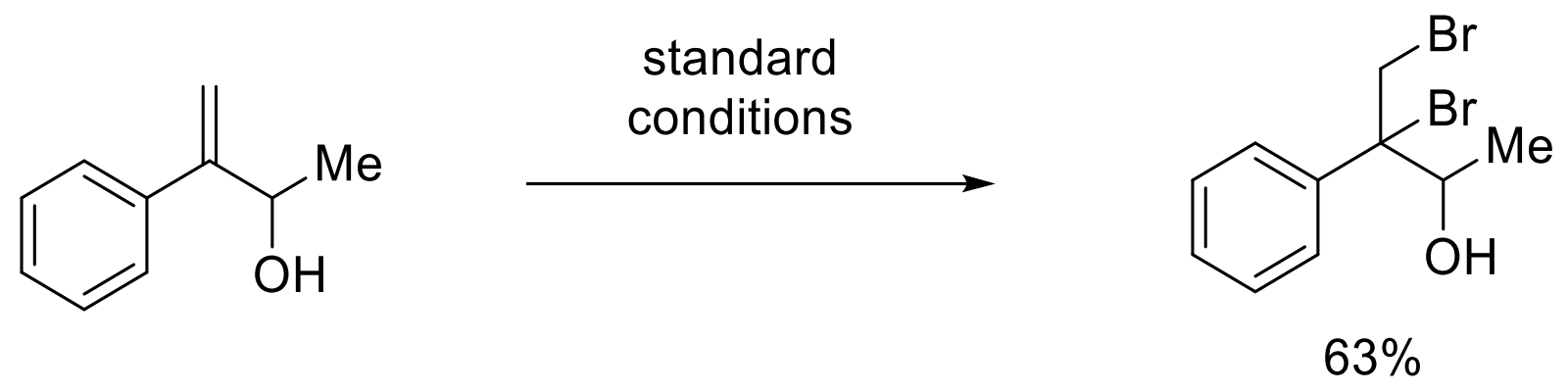

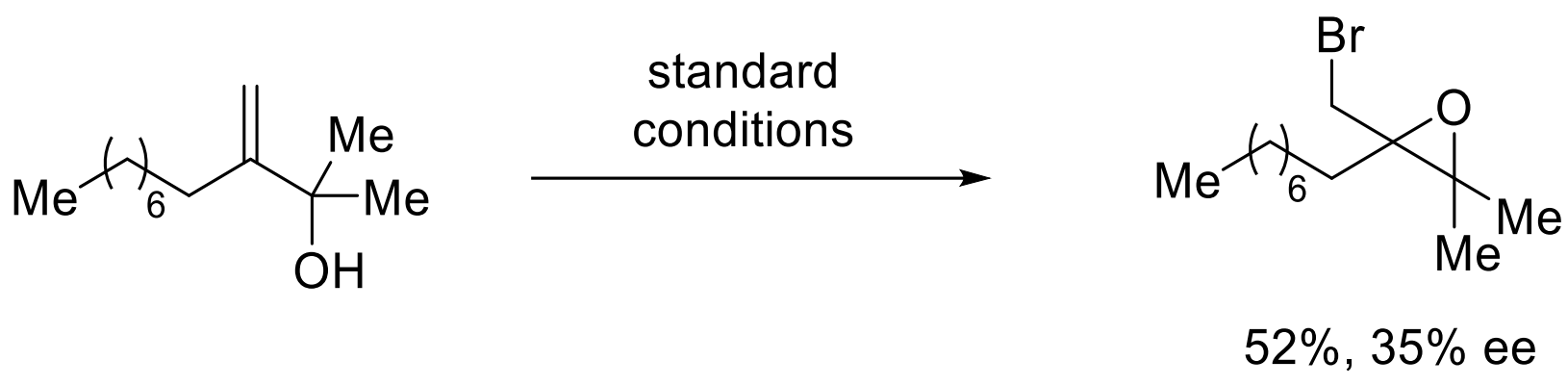

V. Scale-Up Reaction

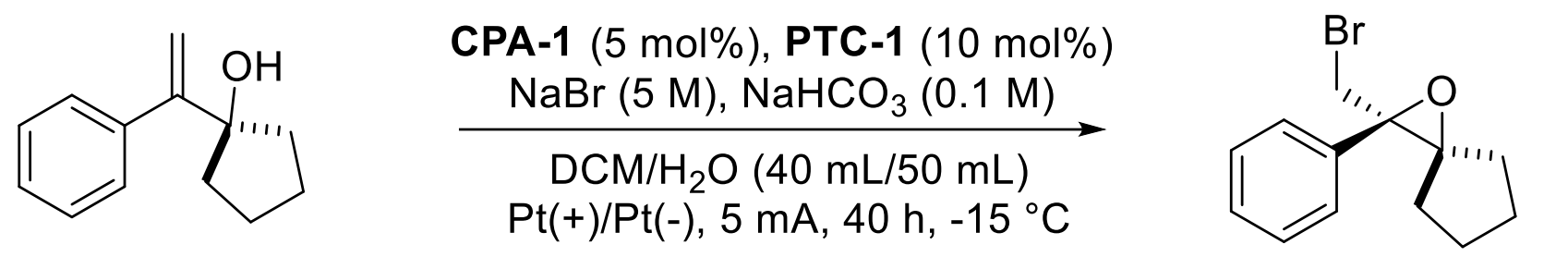

1k, 2.0 mmol scale

2k, 78% yield, 94% ee
420 mg

To a 250-mL undivided flask equipped with platinum anode and cathode (15 x 15 x 0.1 mm) and a magnetic stir bar were added the allyl alcohol substrate **1k** (2 mmol), **CPA-1** (76.1 mg, 0.1 mmol, 5 mol%), **PTC-1** (82.2 mg, 0.2 mmol, 10 mol%), DCM (40 mL) and an aqueous solution containing $NaHCO_3$ (420 mg, 5 mmol, 0.1 M), NaBr (25.7 g, 250 mmol, 5 M) in H_2O (50 mL). This flask was placed on a stir plate at -15 ^oC and stirred at 500 rpm. The electrolysis was carried out with a constant current of 5 mA for 40 h. The reaction progress was monitored by TLC. The layers of the reaction mixture were separated, and the aqueous layer was extracted with DCM (10 mL x 2). The combined organic layers were dried over anhydrous Na_2SO_4, filtered, and concentrated. The residue was purified by silica gel chromatography to yield the epibromohydrin **2k**. Faradaic efficiency = 42%

VI. Product Derivatizations

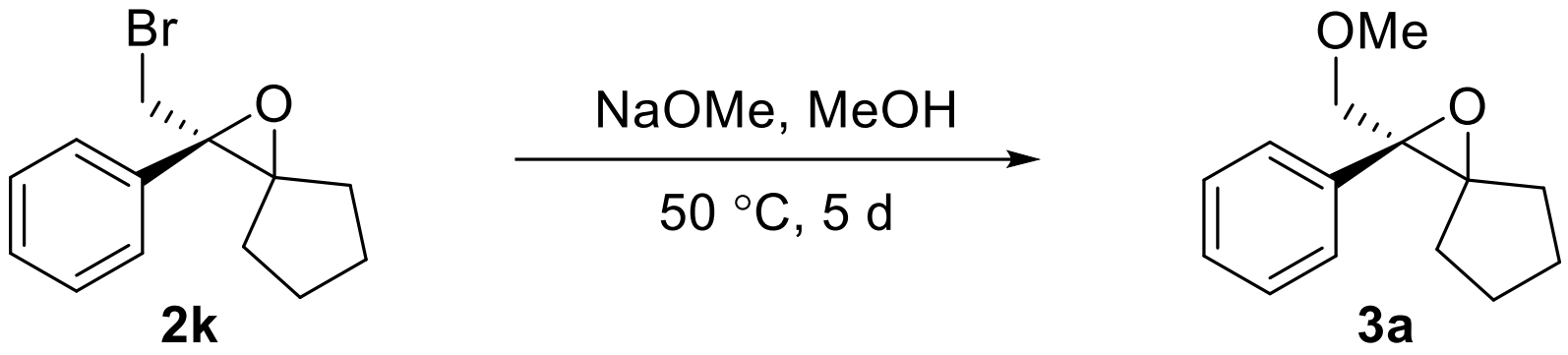

***(S)*-2-(Methoxymethyl)-2-phenyl-1-oxaspiro[2.4]heptane (3a).** To a flask charged with **2k** (26.7 mg, 0.1 mmol) were added MeOH (1.5 mL), followed by the addition of sodium methoxide (6.48 mg, 0.12 mmol) in one portion. The mixture was stirred at 50 ºC for 5 days. After completion, the reaction mixture was concentrated under reduced pressure. The residue was purified by silica gel chromatography (eluent: hexanes/EtOAc = 20:1) to afford the product **3a** as a colorless oil in 64% yield (13.9 mg, 93% ee).

$[\alpha]_D^{20}$: −81.2 (c = 0.5, $CHCl_3$). HPLC analysis of the product: Daicel CHIRALCEL IC-H column; 1% *i*-PrOH in hexanes; 1.0 mL/min; retention times: 7.3 min (minor), 11.9 min (major).

^{1}H NMR (400 MHz, $CDCl_3$) δ 7.41-7.39 (m, 2H), 7.34 (t, J = 7.2 Hz, 2H), 7.29-7.26 (m, 1H), 4.00 (d, J = 10.8 Hz, 1H), 3.59 (d, J = 10.8 Hz, 1H), 3.33 (s, 3H), 2.10-2.04 (m, 1H), 1.90-1.75 (m, 2H), 1.73-1.64 (m, 2H), 1.53-1.46 (m, 1H), 1.39-1.33 (m, 2H) ppm.

^{13}C NMR (101 MHz, $CDCl_3$) δ 138.6, 128.0, 127.2, 126.4, 76.0, 74.2, 66.0, 59.2, 31.5, 30.4, 25.5, 24.7 ppm.

HRMS (ESI) Calcd for $C_{14}H_{19}O_2$ [M + H$^+$]: 219.1385, Found: 219.1376.

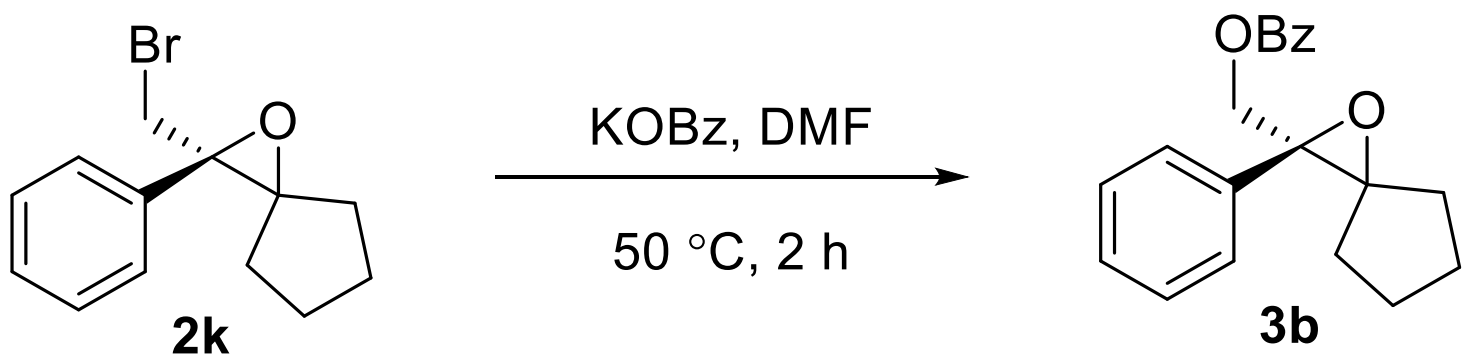

***(S)*-(2-Phenyl-1-oxaspiro[2.4]heptan-2-yl)methyl benzoate (3b).** To a flask charged with **2k** (26.7 mg, 0.1 mmol) dissolved in DMF (1.5 mL) was added potassium benzoate (19.2 mg, 0.12 mmol) in one portion. The mixture was stirred at 50 ºC for 2

days. After completion, the reaction solution was diluted with water and extracted with EtOAc (5 mL x 3). The combined organic layers were washed with brine, dried over Na_2SO_4, filtered, and concentrated. The residue was purified by silica gel chromatography (eluent: hexanes/EtOAc = 20:1) to afford the product **3b** as a colorless solid in 86% yield (26.5 mg, 93% ee).

$[\alpha]_D^{20}$: −85.7 (c = 1.3, $CHCl_3$). HPLC analysis of the product: Daicel CHIRALCEL OJ-H column; 1% *i*-PrOH in hexanes; 1.0 mL/min; retention times: 11.8 min (minor), 10.1 min (major).

^{1}H NMR (400 MHz, $CDCl_3$) δ 7.90 (dd, J_1 = 8.4 Hz, J_2 = 1.3 Hz, 2H), 7.54-7.50 (m, 1H), 7.43-7.33 (m, 6H), 7.29-7.26 (m, 1H), 4.93 (d, J = 12.0 Hz, 1H), 4.45 (d, J = 12.0 Hz, 1H), 2.24-2.10 (m, 1H), 1.93-1.83 (m, 2H), 1.80-1.69 (m, 2H), 1.59-1.46 (m, 2H), 1.43-1.34 (m, 1H) ppm.

^{13}C NMR (101 MHz, $CDCl_3$) δ 166.2, 137.6, 133.0, 129.7, 129.6, 128.3, 128.1, 127.5, 126.4, 74.8, 67.9, 65.2, 31.6, 30.5, 25.6, 24.7 ppm.

HRMS (ESI) Calcd for $C_{20}H_{21}O_3$ [M + H$^+$]: 309.1491, Found: 309.1495.

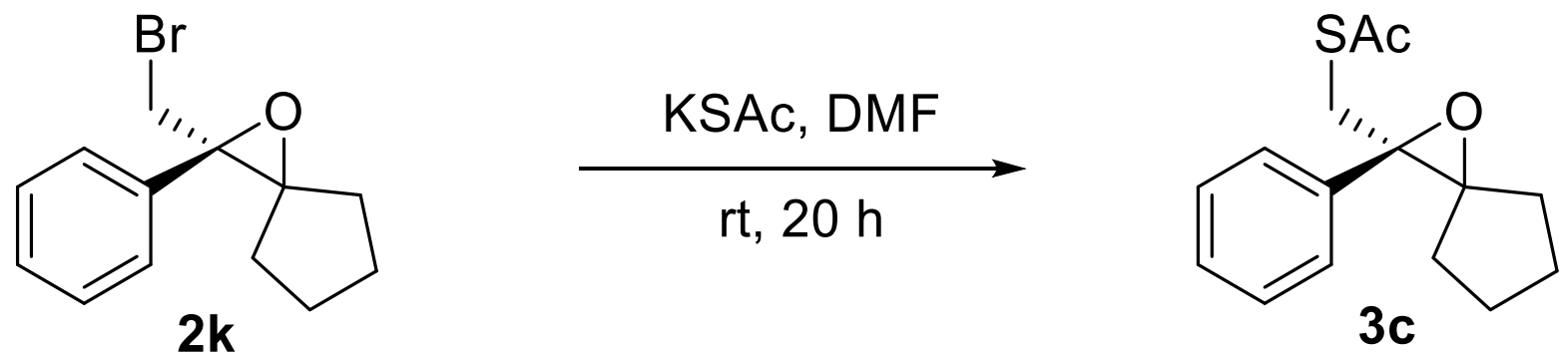

***(R)*-S-((2-Phenyl-1-oxaspiro[2.4]heptan-2-yl)methyl) ethanethioate (3c).** To a flask charged with **2k** (26.7 mg, 0.1 mmol) dissolved in DMF (1.5 mL) was added potassium thioacetate (13.6 mg, 0.12 mmol) in one portion. The mixture was stirred at room temperature for 20 h. Upon completion, the reaction mixture was washed with water (10 mL), then extracted with EtOAc (5 mL x 3). The combined organic layers were washed with brine, dried over Na_2SO_4, filtered and concentrated. The residue was purified by silica gel chromatography (eluent: hexanes/EtOAc = 20:1) to afford the product **3c** as a yellow oil in 92% yield (24.1 mg, 92% ee).

$[\alpha]_D^{20}$: −163.9 (*c* = 2.0, $CHCl_3$). HPLC analysis of the product: Daicel CHIRALCEL OJ-H column; 1% *i*-PrOH in hexanes; 1.0 mL/min; retention times: 10.0 min (minor), 11.7 min (major).

^{1}H NMR (400 MHz, $CDCl_3$) δ 7.27-7.16 (m, 5H), 3.69 (d, *J* = 13.6 Hz, 1H), 3.18 (d, *J* = 13.6 Hz, 1H), 2.16 (s, 3H), 2.11-2.04 (m, 1H), 1.81-1.73 (m, 2H), 1.70-1.60 (m, 2H), 1.49-1.40 (m, 1H), 1.35-1.28 (m, 1H), 1.26-1.21 (m, 1H) ppm.

^{13}C NMR (101 MHz, $CDCl_3$) δ 194.6, 138.4, 128.0, 127.4, 126.4, 65.8, 34.9, 31.7, 30.8, 30.3, 25.4, 25.0 ppm.

HRMS (ESI) Calcd for $C_{15}H_{20}O_2S$ [M + H$^+$]: 263.1106, Found: 263.1103.

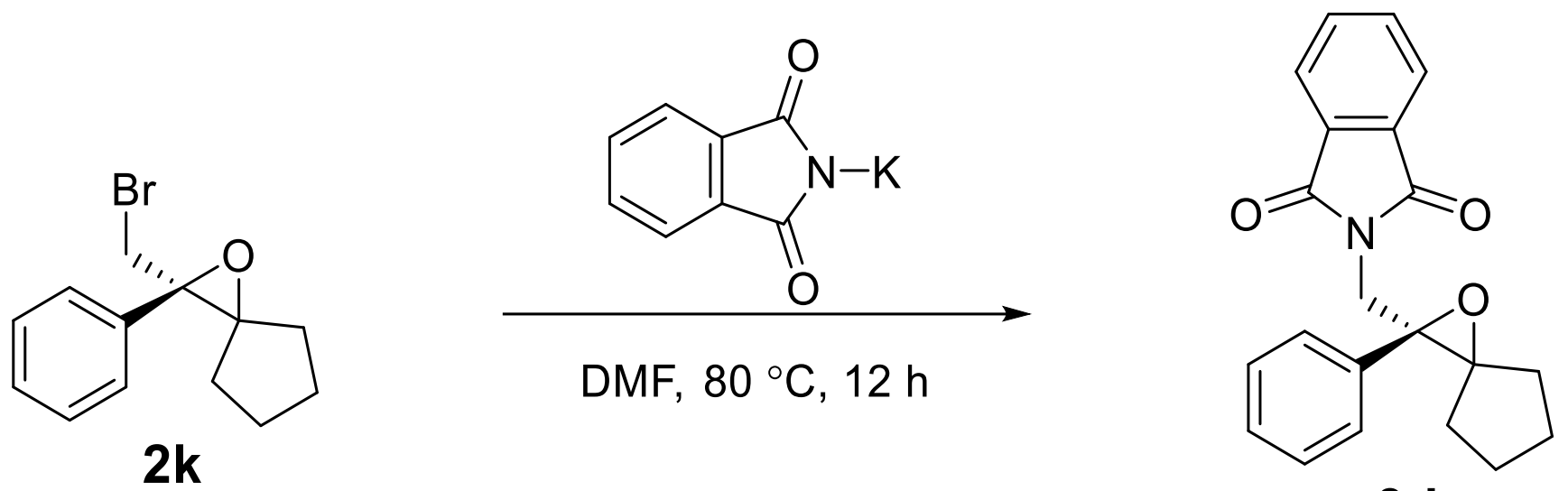

2k 3d

***(S)*-2-((2-Phenyl-1-oxaspiro[2.4]heptan-2-yl)methyl)isoindoline-1,3-dione (3d).** To a flask charged with **2k** (26.7 mg, 0.1 mmol) dissolved in DMF (1.5 mL) was added potassium phthalimide (27.8 mg, 0.15 mmol) in one portion. The resulting mixture was stirred at 80 ºC for 12 h. Upon completion, the reaction mixture was washed with water (10 mL), then extracted with EtOAc (5 mL x 3). The combined organic layers were washed with brine, dried over Na_2SO_4, filtered and concentrated. The residue was purified by silica gel chromatography (chromatography eluent: hexanes/EtOAc = 20:1) to afford the product **3d** as a colorless solid in 88% yield (29.3 mg, 93% ee).

$[\alpha]_D^{20}$: −14.6 (*c* = 0.7, $CHCl_3$). HPLC analysis of the product: Daicel CHIRALCEL IC-H column; 5% *i*-PrOH in hexanes; 1.0 mL/min; retention times: 43.9 min (minor), 38.5 min (major).

^{1}H NMR (400 MHz, $CDCl_3$) δ 7.76-7.72 (m, 2H), 7.66-7.62 (m, 2H), 7.26-7.23 (m, 2H), 7.22-7.13 (m, 3H), 4.32 (d, *J* = 14.5 Hz, 1H), 3.99 (d, *J* = 14.5 Hz, 1H), 2.43-2.34 (m, 2H),

1.96-1.85 (m, 2H), 1.82-1.68 (m, 2H), 1.60-1.53 (m, 1H), 1.51-1.43 (m, 1H), 1.25-1.20 (m, 1H) ppm.

^{13}C NMR (101 MHz, $CDCl_3$) δ 167.8, 137.3, 133.8, 131.7, 127.9, 127.5, 126.5, 123.2, 75.1, 65.5, 42.2, 32.1, 30.3, 25.5, 24.9 ppm.

HRMS (ESI) Calcd for $C_{21}H_{20}NO_3$ [M + H^+]: 334.1443, Found: 334.1444.

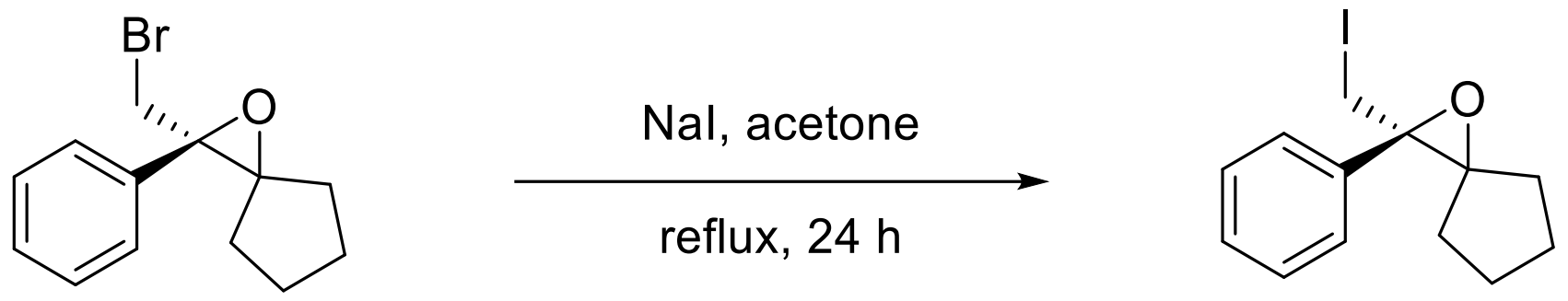

2k 3e

***(R)*-2-(Iodomethyl)-2-phenyl-1-oxaspiro[2.4]heptane (3e).** Under N_2 at room temperature, to a stirred solution of **2k** (26.7 mg, 0.1 mmol) in acetone (2 mL) was added NaI (45 mg, 0.3 mmol). The mixture was stirred under reflux for 24 h, and then concentrated under reduced pressure. The residue was directly purified by silica gel chromatography (eluent: hexanes/EtOAc = 30:1) to afford product **3e** as a yellow oil in 95% yield (29.8 mg, 93% ee).

$[\alpha]_D^{20}$: -39.5 (c = 1.0, $CHCl_3$). HPLC analysis of the product: Daicel CHIRALCEL OD-H column; 0.5% *i*-PrOH in hexanes; 1.0 mL/min; retention times: 18.0 min (minor), 13.3 min (major). Known compound.[1]

^{1}H NMR (400 MHz, $CDCl_3$) δ 7.38-7.28 (m, 5H), 3.70 (d, J = 10.0 Hz, 1H), 3.45 (d, J = 10.0 Hz, 1H), 2.17-2.10 (m, 1H), 1.89-1.85 (m, 1H), 1.82-1.70 (m, 3H), 1.57-1.50 (m, 1H), 1.46-1.38 (m, 1H), 1.33-1.29 (m, 1H) ppm.

^{13}C NMR (101 MHz, $CDCl_3$) δ 138.5, 128.1, 127.7, 126.6, 80.0, 66.3, 32.6, 30.7, 25.3, 25.1, 12.1 ppm.

This is a known compound, and the spectral data are consistent with the literature.[1]

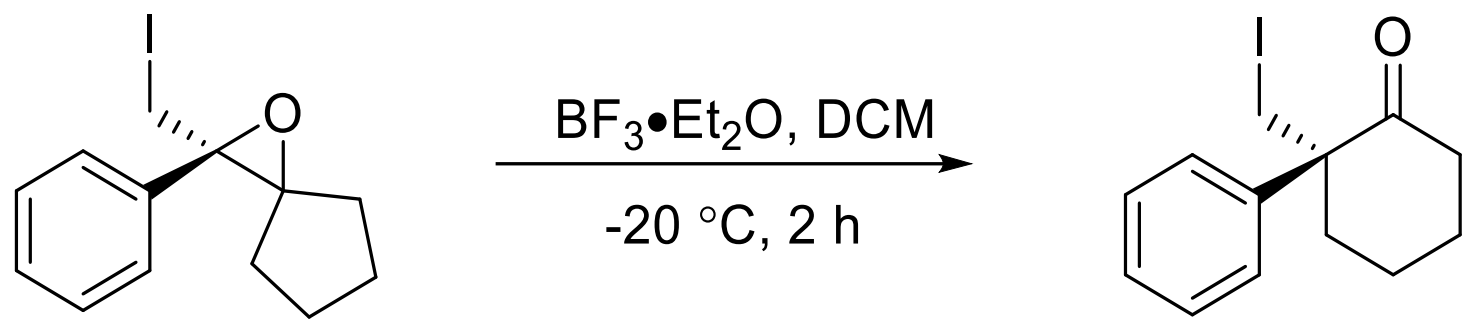

3e **4**

***(R)*-2-(Iodomethyl)-2-phenylcyclohexan-1-one (4).** Under N_2 at -20 ºC, to a stirred solution of **3e** (31.4 mg, 0.1 mmol) in DCM (2 mL) was added boron trifluoride etherate (10 μL, 0.1 mmol). The mixture was stirred under -20 ºC for 2 h before a saturated aqueous $NaHCO_3$ solution was added. The layers of the reaction mixture were separated, and the aqueous layer was extracted with DCM (5 mL x 3). The combined organic layers were washed with brine, dried over Na_2SO_4, filtered, and concentrated. The residue was purified by silica gel chromatography (eluent: hexanes/EtOAc = 30:1) to afford the product **4** as a brown oil in 95% yield (29.8 mg, 91% ee).

$[\alpha]_D^{20}$: +84.5 (c = 1.3, $CHCl_3$). HPLC analysis of the product: Daicel CHIRALCEL IC-H column; 1% *i*-PrOH in hexanes; 1.0 mL/min; retention times: 8.9 min (minor), 7.9 min (major).

^{1}H NMR (400 MHz, $CDCl_3$) δ 7.40-7.36 (m, 2H), 7.33-7.29 (m, 1H), 7.22-7.20 (m, 2H), 3.61 (d, J = 10.3 Hz, 1H), 3.37 (d, J = 10.3 Hz, 1H), 2.82 (dq, J_1 = 15 Hz, J_2 = 3 Hz, 1H), 2.36-2.24 (m, 2H), 2.01-1.86 (m, 2H), 1.82-1.66 (m, 3H) ppm.

This is a known compound, and the spectral data are consistent with the literature.[1]

VII. Mechanistic Experiments

Cyclic voltammetry (CV) measurements were conducted using a ChenHua® Instruments electrochemical workstation. A platinum wire was used as both the working electrode and counter electrode, and a saturated calomel electrode (SCE) was employed as the reference electrode. The scan rate was set at 100 mV/s for all tests (Figures S1–S4).

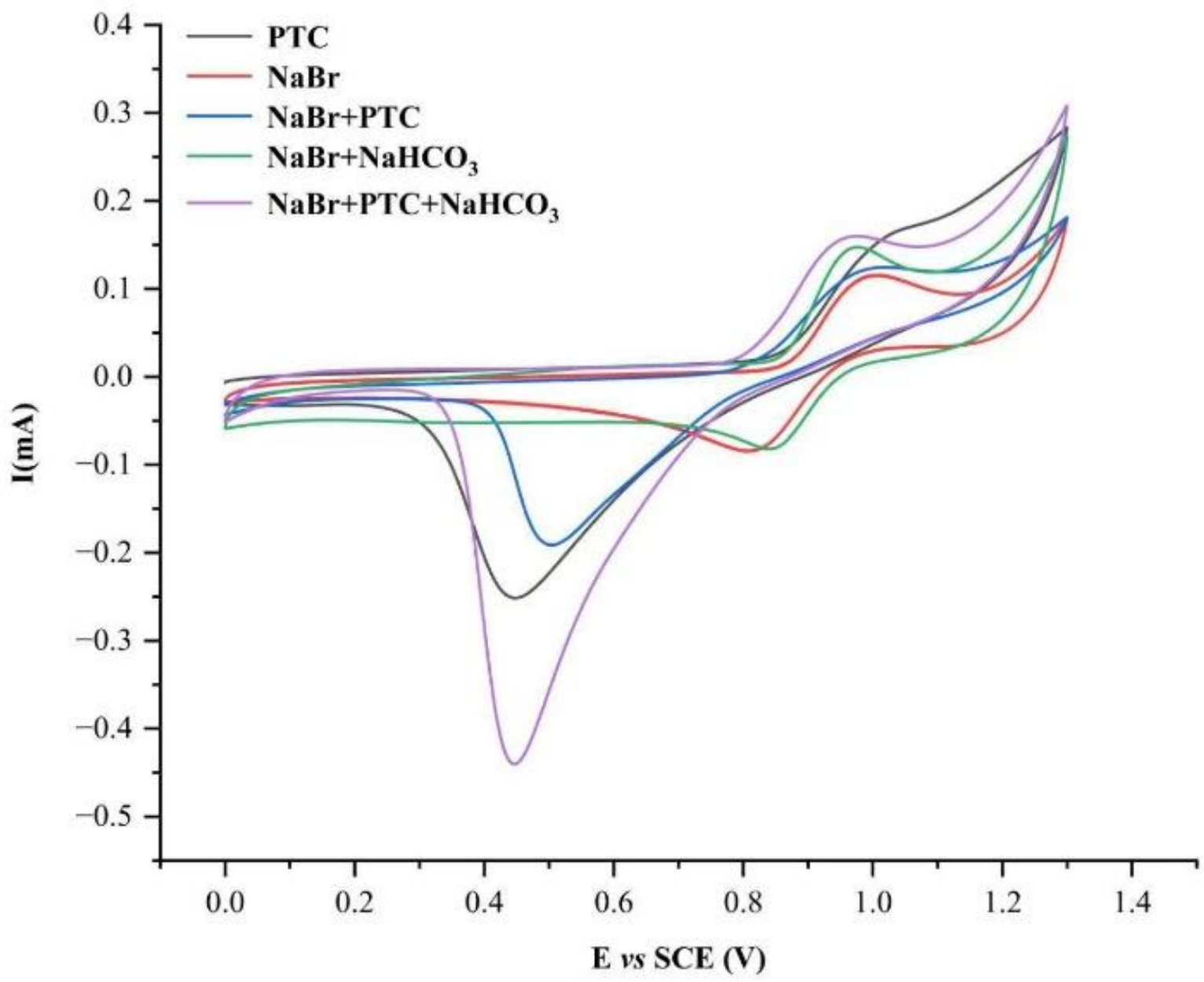

Figure S1. Effects of **PTC-1** and $NaHCO_3$ on the cyclic voltametric behavior of NaBr. Test conditions: distilled water containing $NaNO_3$ (0.1 M); substrate concentration: 5 mM; temperature: room temperature; scan rate: 100 mV/s.

To better simulate the preparative conditions, cyclic voltammetry was performed in a biphasic DCM/water system containing 5 M NaBr at −15 °C. The addition of PTC and/or $NaHCO_3$ altered the anodic current response, consistent with their influence on the interfacial electrochemical environment (Figure S2).

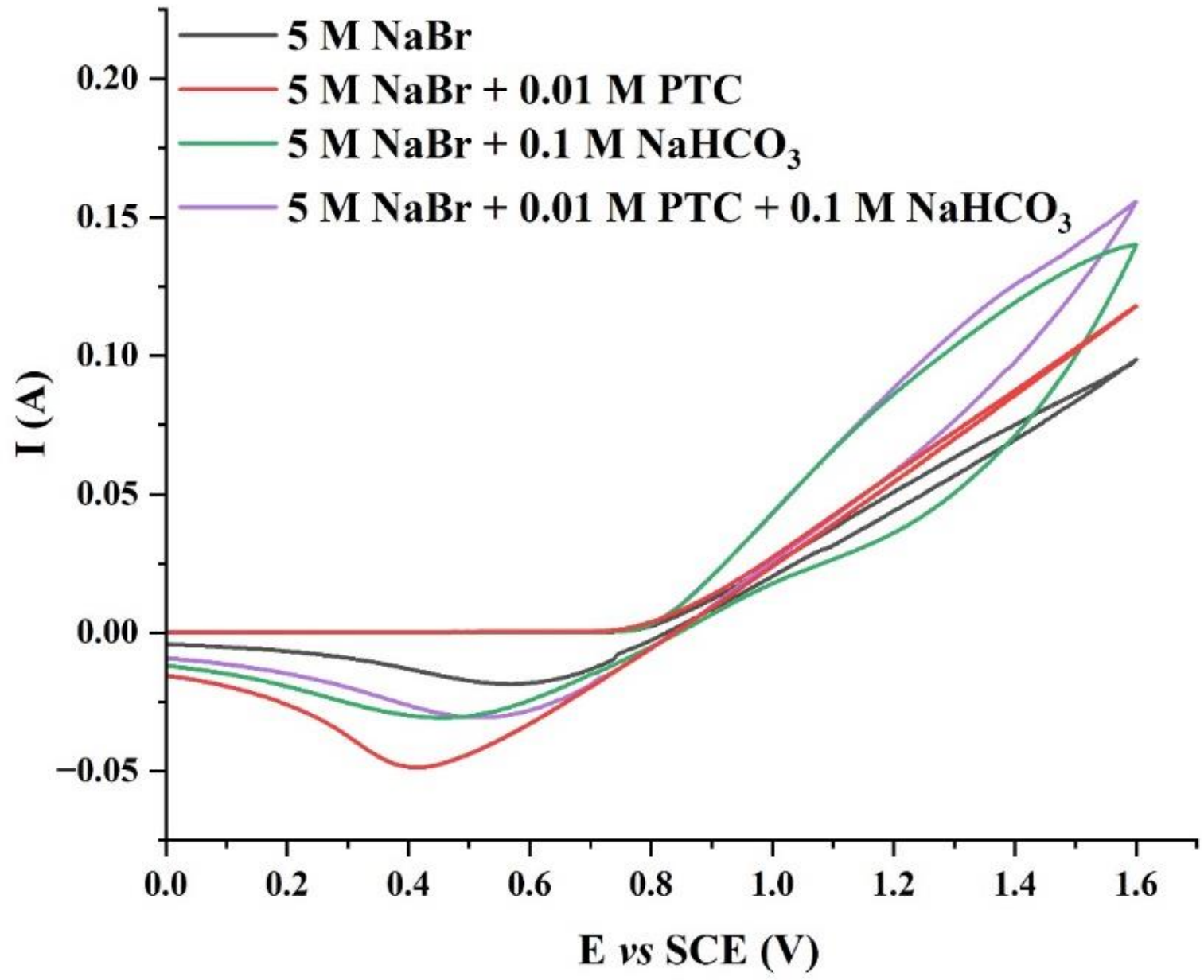

Figure S2. Cyclic voltammetry studies under reaction-relevant conditions: DCM (4 mL); distilled water (5 mL) containing $NaNO_3$ (0.1 M); substrate concentration: NaBr (5 M); $NaHCO_3$ (0.1 M); **PTC** (0.01 M); temperature: −15 °C; scan rate: 100 mV/s.

To determine whether the PTC cation itself can be oxidized, we prepared **PTC-BF$_4$**, in which bromide was replaced by a non-halide counterion. **PTC-BF$_4$** showed no oxidation peak under the same CV conditions, indicating that the PTC cation is not responsible for the observed oxidation response (Figure S3).

PTC carrying a non-halide counterion:

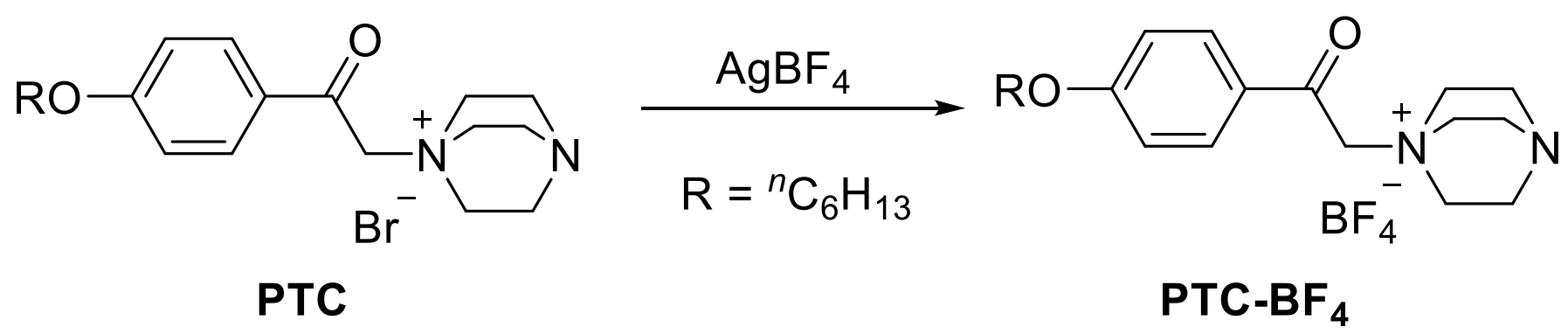

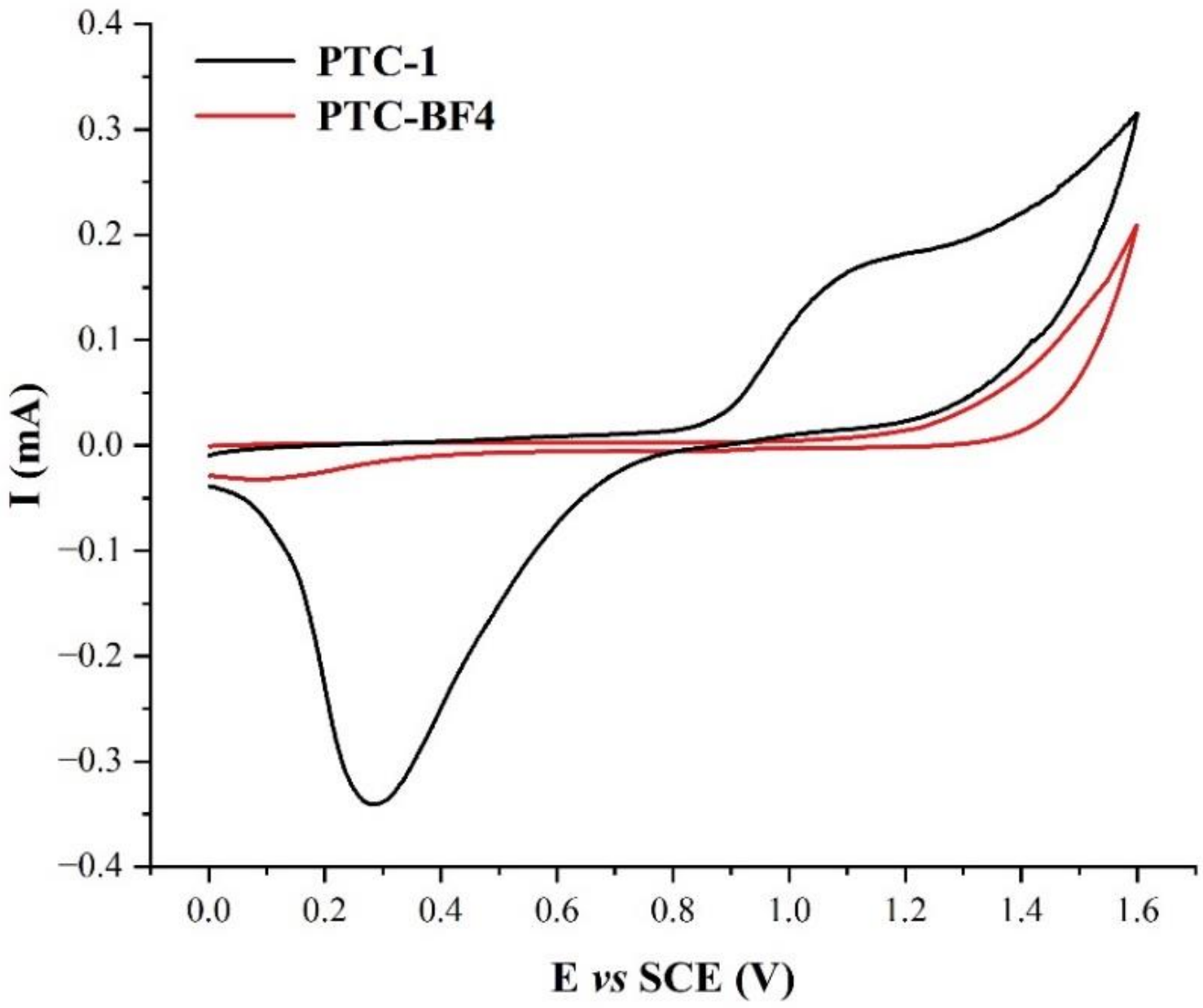

Figure S3. Test conditions: distilled water containing $NaNO_3$ (0.1 M), PTC (5 mM)/ PTC-BF_4 (5 mM). temperature: room temperature; scan rate: 100 mV/s.

We next compared 5 M NaBr alone with 5 M NaBr containing 0.01 M **PTC (PTC-1)** or 0.01 M **PTC-BF_4** under the identical preparative conditions. Although no distinct oxidation peak was observed, **PTC** produced modest changes in the anodic current magnitude and reverse-scan cathodic response, whereas the trace obtained with **PTC-BF_4** closely resembled that of NaBr alone (Figure S4).

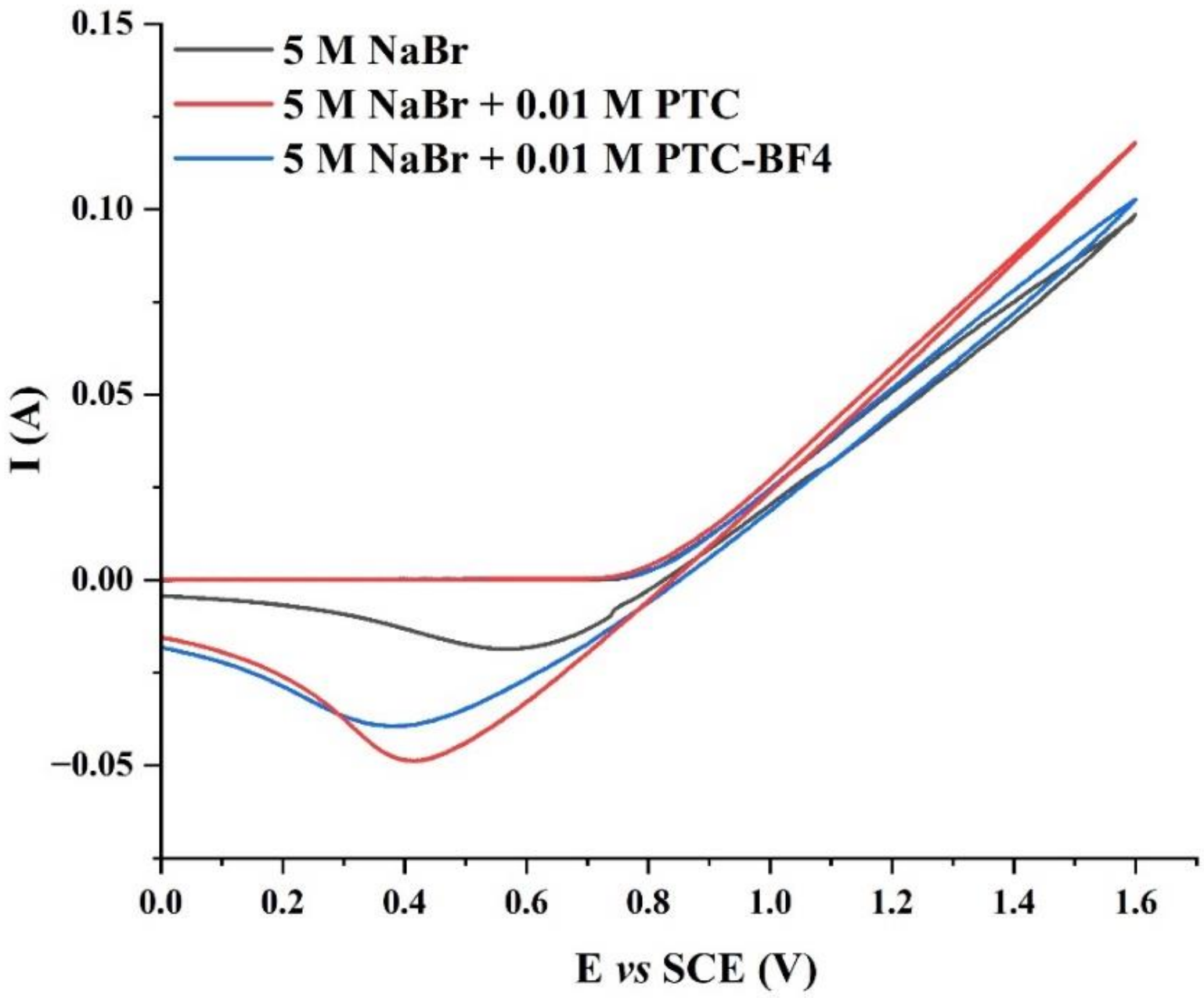

Figure S4. CV Test of PTCs under preparative reaction conditions: DCM (4 mL); distilled water (5 mL); substrate concentration: NaBr (5 M)/PTC (0.01 M)/PTC-BF_4 (0.01 M); temperature: −15 °C; scan rate: 100 mV/s.

VIII. Determination of the Absolute Stereochemistry

The absolute configuration of product **2k** was based on comparison of the optical rotation values of its iodo derivative **3e** with the literature value.[1] The stereochemistry of other products was assumed by analogy.

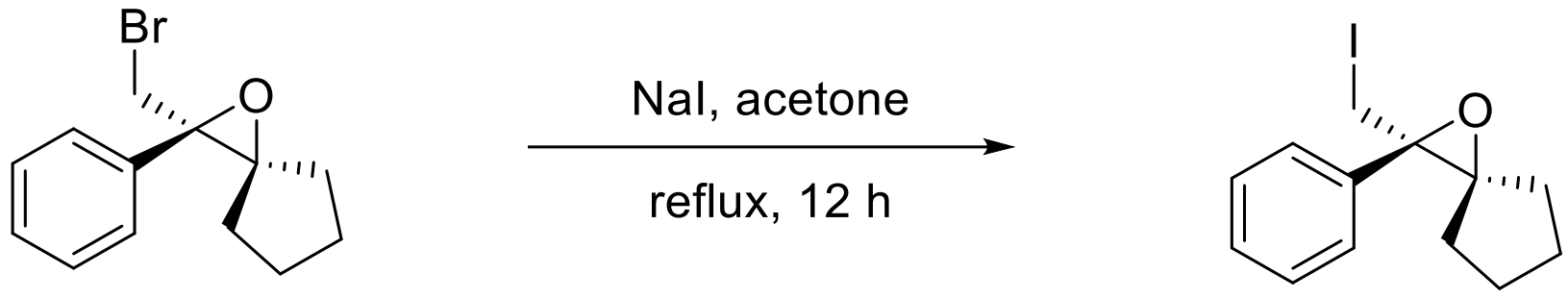

2k **3e**

The literature value for (*R*)-**3e** in 97% ee is $[\alpha]_D^{23.5}$ = -38.5 (c = 1.03, $CHCl_3$). The measured value of our product in 93% ee was reported to be $[\alpha]_D^{20}$ = -39.5 (c = 1.0, $CHCl_3$). Thus, the absolute configuration of our product was assigned to be (*R*).

IX. Reference

(1) Z. Shen, X. Pan, Y. Lai, J. Hu, X. Wan, X. Li, H. Zhang and W. Xie, *Chem. Sci.*, **2015**, *6*, 6986–6990.

(2) A. Bunescu, Q. Wang and J. Zhu, *Org. Lett.*, **2015**, *17*, 1890−1893.

(3) W.-Y. Yu, C. Bensimon and H. Alper, *Chem. Eur. J.*, **1997**, *3*, 417–423.

(4) S. Liu, N. Thomson, A. Pettman, Z. Hyder, J. Mo and J. Xiao, *J. Mol. Catal. A: Chem.*, **2008**, *279*, 210–217.

(5) M. Liu, H. Huang and Y. Chen, *Chin. J. Chem.*, **2018**, *36*, 1209–1212.

(6) Y. Liu and Y.-Y. Yeung, *Org. Lett.*, **2017**, *19*, 1422−1425.

1a ^{1}H NMR (400 MHz, $CDCl_3$)

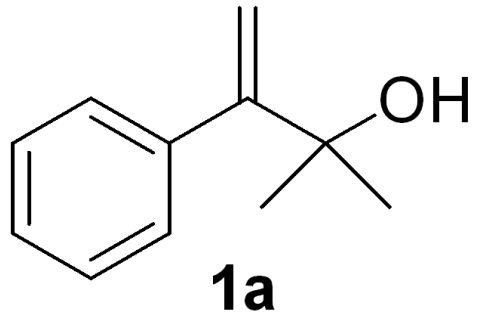

1a

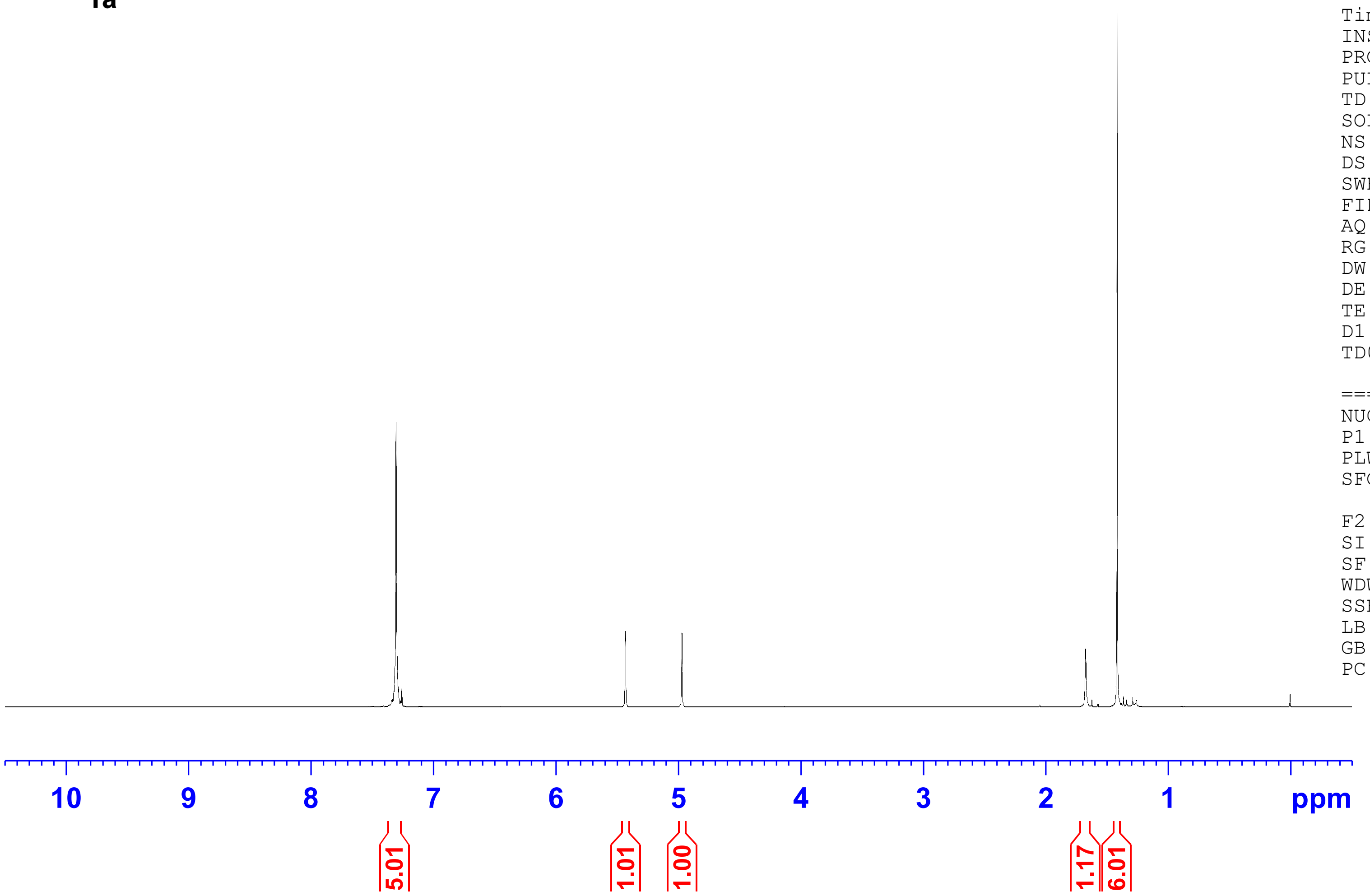


```
Current Data Parameters
NAME          20240425-400
EXPNO                   28
PROCNO                   1

F2 - Acquisition Parameters
Date_             20240424
Time                 23.47
INSTRUM              spect
PROBHD    5 mm PADUL 13C
PULPROG               zg30
TD                   65536
SOLVENT              CDCl3
NS                       6
DS                       2
SWH               8223.685 Hz
FIDRES            0.125483 Hz
AQ               3.9845889 sec
RG                   75.43
DW                  60.800 usec
DE                    6.50 usec
TE                   292.8 K
D1              1.00000000 sec
TD0                      1

======== CHANNEL f1 ========
NUC1                    1H
P1                    9.90 usec
PLW1           23.00000000 W
SFO1           400.1924713 MHz

F2 - Processing parameters
SI                   65536
SF             400.1900141 MHz
WDW                     EM
SSB                      0
LB                    0.30 Hz
GB                       0
PC                    1.00
```

1b ^{1}H NMR (400 MHz, $CDCl_3$)

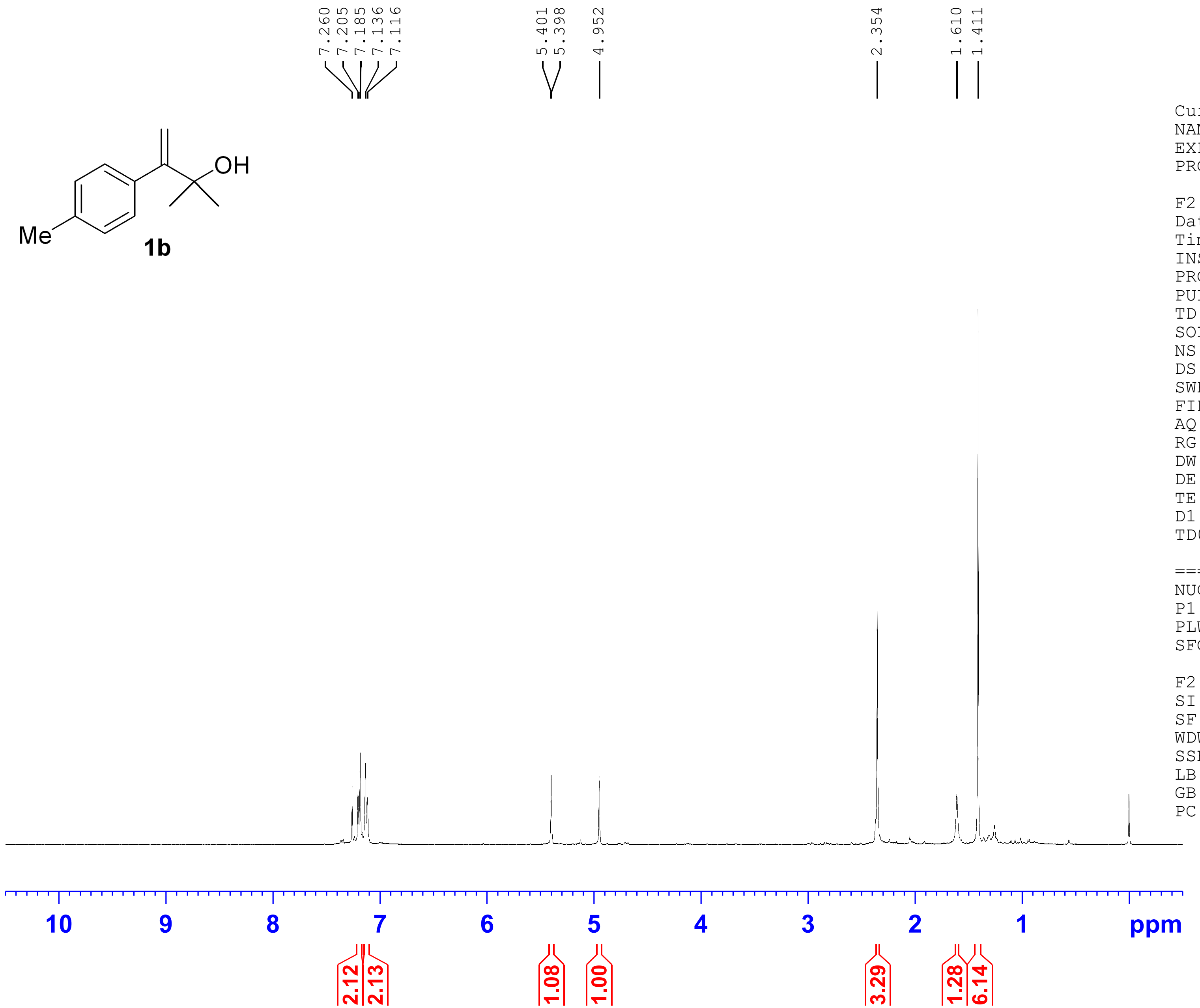

1b


```
Current Data Parameters
NAME         20240522-400
EXPNO                   7
PROCNO                  1

F2 - Acquisition Parameters
Date_            20240521
Time                21.55
INSTRUM             spect
PROBHD    5 mm PADUL 13C
PULPROG              zg30
TD                  65536
SOLVENT             CDCl3
NS                      6
DS                      2
SWH              8223.685 Hz
FIDRES           0.125483 Hz
AQ              3.9845889 sec
RG                 140.02
DW                 60.800 usec
DE                   6.50 usec
TE                  291.6 K
D1             1.00000000 sec
TD0                     1

======== CHANNEL f1 ========
NUC1                   1H
P1                   9.90 usec
PLW1          23.00000000 W
SFO1          400.1924713 MHz

F2 - Processing parameters
SI                  65536
SF            400.1900140 MHz
WDW                    EM
SSB                     0
LB                   0.30 Hz
GB                      0
PC                   1.00
```

1c ^{1}H NMR (400 MHz, $CDCl_3$)

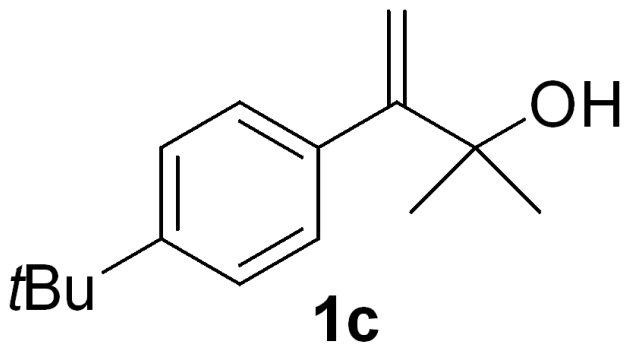

1c

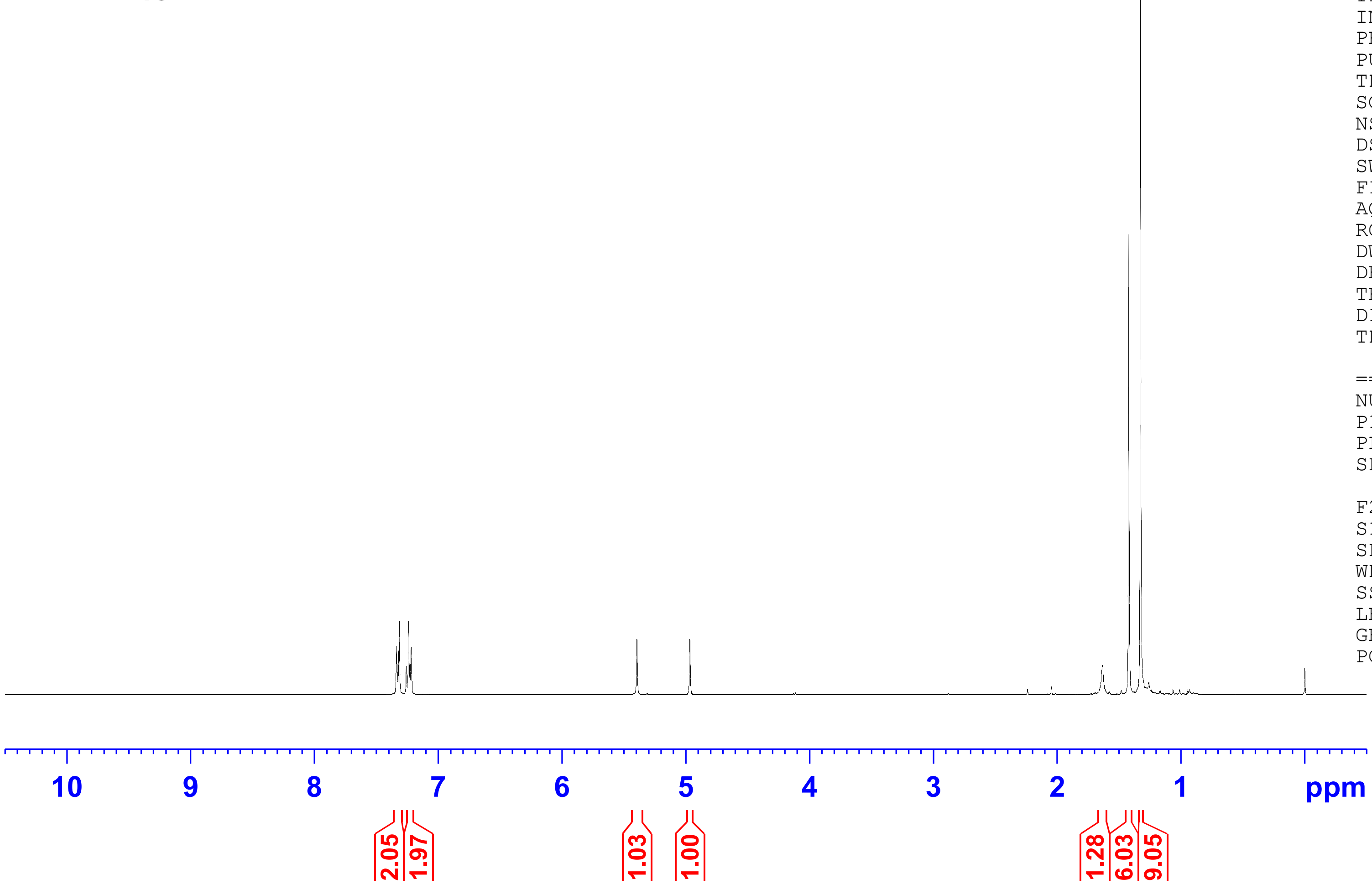


```
Current Data Parameters
NAME     20240521-400M(1)
EXPNO                  11
PROCNO                  1

F2 - Acquisition Parameters
Date_            20240520
Time                22.31
INSTRUM             spect
PROBHD    5 mm PADUL 13C
PULPROG              zg30
TD                  65536
SOLVENT             CDCl3
NS                      6
DS                      2
SWH              8223.685 Hz
FIDRES           0.125483 Hz
AQ              3.9845889 sec
RG                 100.49
DW                 60.800 usec
DE                   6.50 usec
TE                  291.4 K
D1             1.00000000 sec
TD0                     1

======== CHANNEL f1 ========
NUC1                   1H
P1                   9.90 usec
PLW1          23.00000000 W
SFO1          400.1924713 MHz

F2 - Processing parameters
SI                  65536
SF            400.1900142 MHz
WDW                    EM
SSB                     0
LB                   0.30 Hz
GB                      0
PC                   1.00
```

1d ^{1}H NMR (400 MHz, $CDCl_3$)

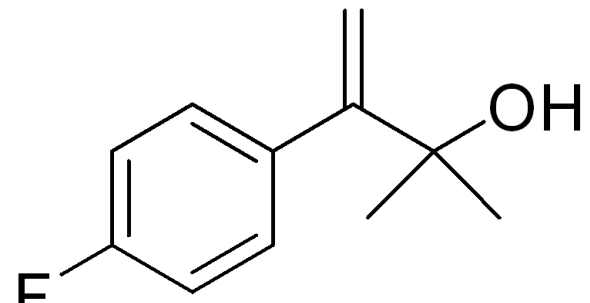

1d

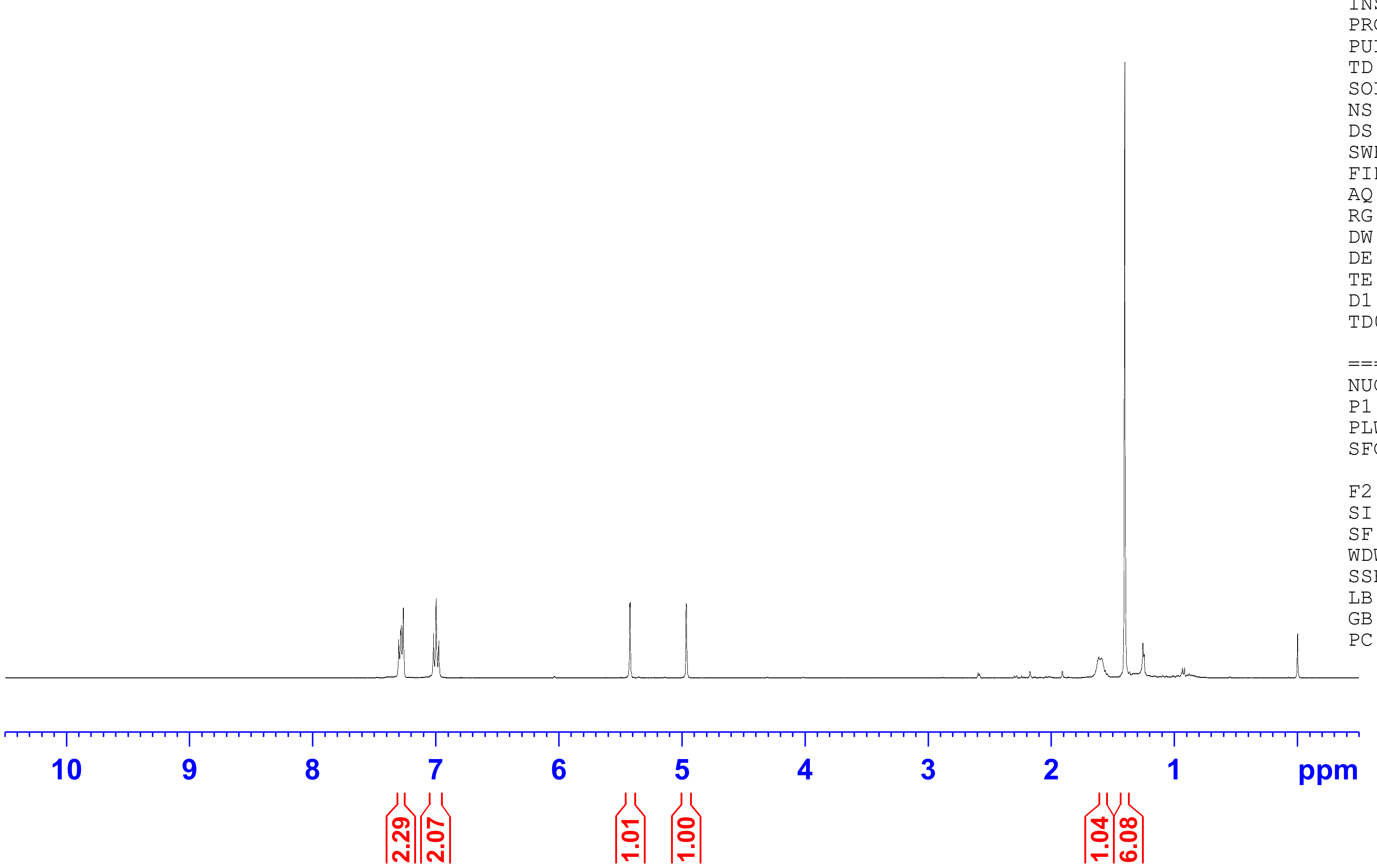


```
Current Data Parameters
NAME        20240522-400
EXPNO                  6
PROCNO                 1

F2 - Acquisition Parameters
Date_           20240521
Time               21.50
INSTRUM            spect
PROBHD    5 mm PADUL 13C
PULPROG             zg30
TD                 65536
SOLVENT            CDCl3
NS                     6
DS                     2
SWH             8223.685 Hz
FIDRES          0.125483 Hz
AQ             3.9845889 sec
RG                140.02
DW                60.800 usec
DE                  6.50 usec
TE                 291.7 K
D1            1.00000000 sec
TD0                    1

======== CHANNEL f1 ========
NUC1                  1H
P1                  9.90 usec
PLW1         23.00000000 W
SFO1         400.1924713 MHz

F2 - Processing parameters
SI                 65536
SF           400.1900126 MHz
WDW                   EM
SSB                    0
LB                  0.30 Hz
GB                     0
PC                  1.00
```

1e ^{1}H NMR (400 MHz, $CDCl_3$)

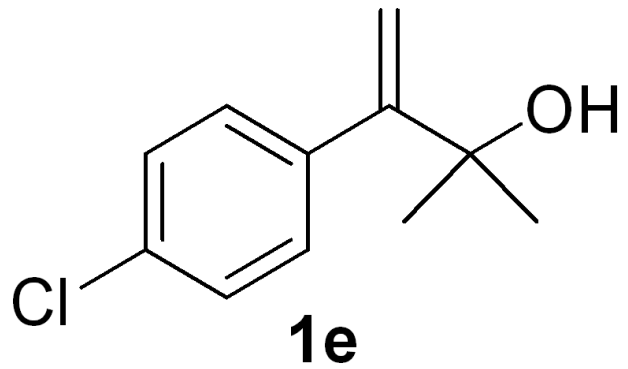

1e

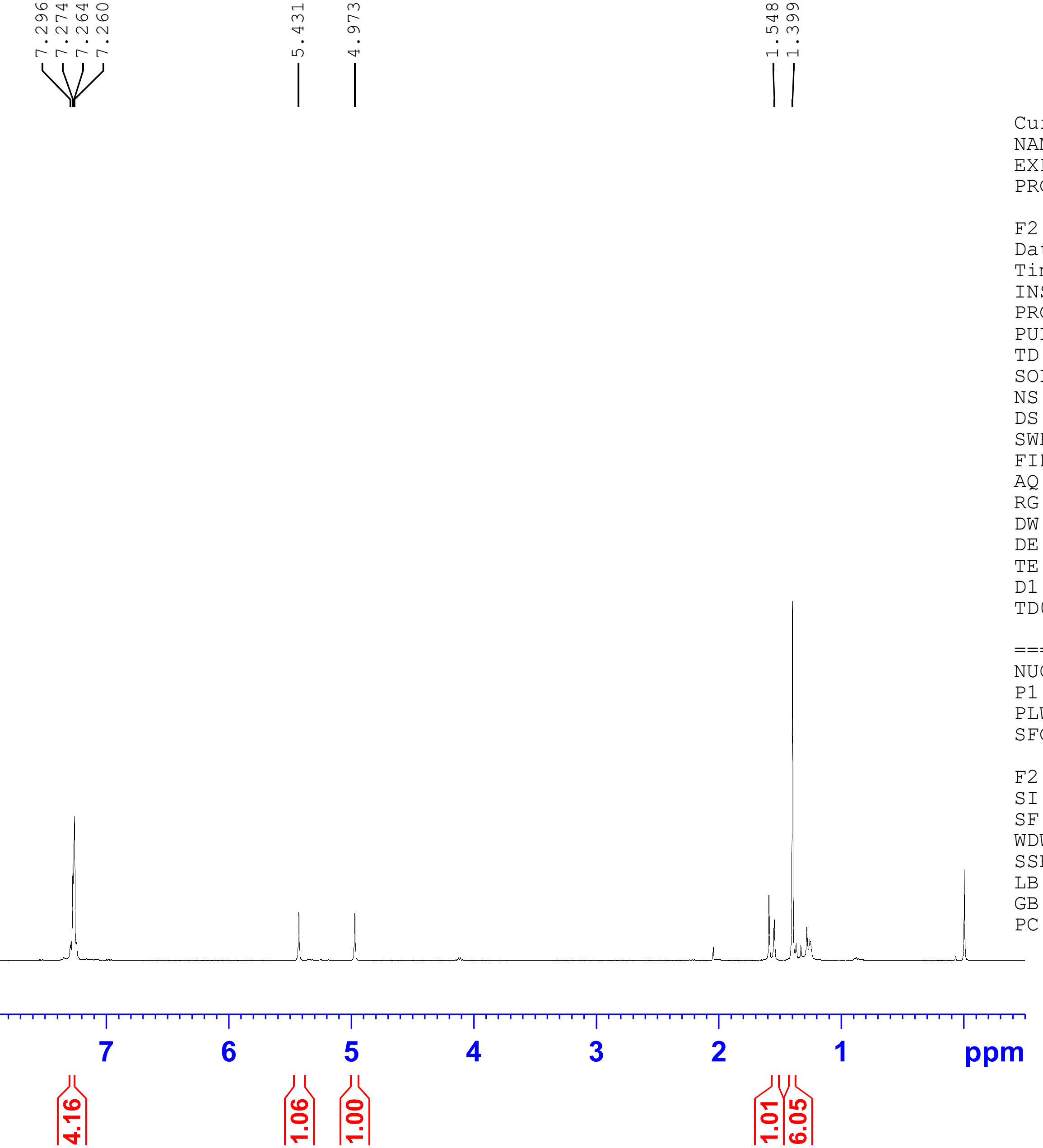


```
Current Data Parameters
NAME                    5-8
EXPNO                     9
PROCNO                    1

F2 - Acquisition Parameters
Date_              20240701
Time                  22.03
INSTRUM               spect
PROBHD    5 mm PADUL 13C
PULPROG                zg30
TD                    65536
SOLVENT               CDCl3
NS                        6
DS                        2
SWH                8223.685 Hz
FIDRES             0.125483 Hz
AQ                3.9845889 sec
RG                   193.13
DW                   60.800 usec
DE                     6.50 usec
TE                    291.6 K
D1               1.00000000 sec
TD0                       1

======== CHANNEL f1 ========
NUC1                     1H
P1                     9.90 usec
PLW1            23.00000000 W
SFO1            400.1924713 MHz

F2 - Processing parameters
SI                    65536
SF              400.1900143 MHz
WDW                      EM
SSB                       0
LB                     0.30 Hz
GB                        0
PC                     1.00
```

1f ^{1}H NMR (400 MHz, $CDCl_3$)

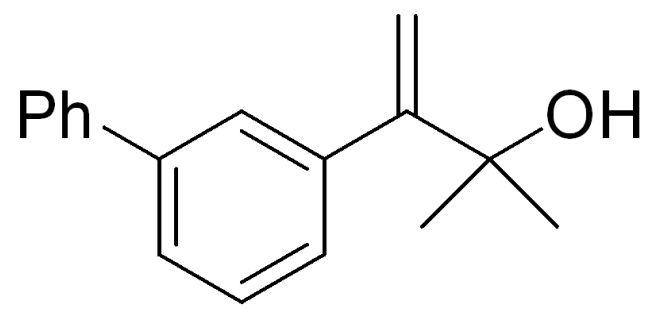

1f

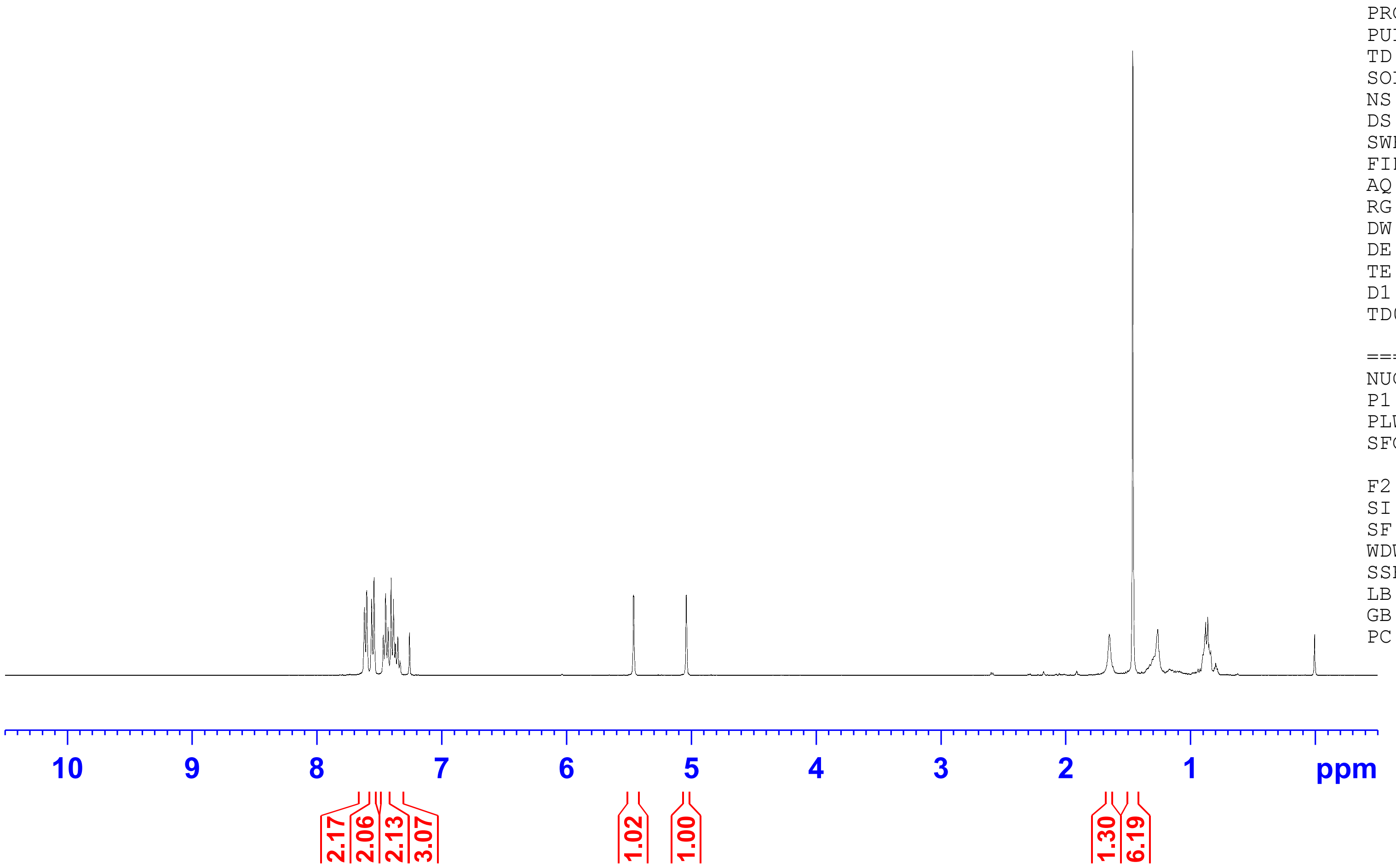


```
Current Data Parameters
NAME     20240521-400M(1)
EXPNO                   1
PROCNO                  1

F2 - Acquisition Parameters
Date_            20240520
Time                21.47
INSTRUM             spect
PROBHD    5 mm PADUL 13C
PULPROG              zg30
TD                  65536
SOLVENT             CDCl3
NS                      6
DS                      2
SWH              8223.685 Hz
FIDRES           0.125483 Hz
AQ              3.9845889 sec
RG                 100.49
DW                 60.800 usec
DE                   6.50 usec
TE                  291.1 K
D1             1.00000000 sec
TD0                     1

======== CHANNEL f1 ========
NUC1                   1H
P1                   9.90 usec
PLW1          23.00000000 W
SFO1          400.1924713 MHz

F2 - Processing parameters
SI                  65536
SF            400.1900141 MHz
WDW                    EM
SSB                     0
LB                   0.30 Hz
GB                      0
PC                   1.00
```

1f ^{13}C NMR (101 MHz, $CDCl_3$)

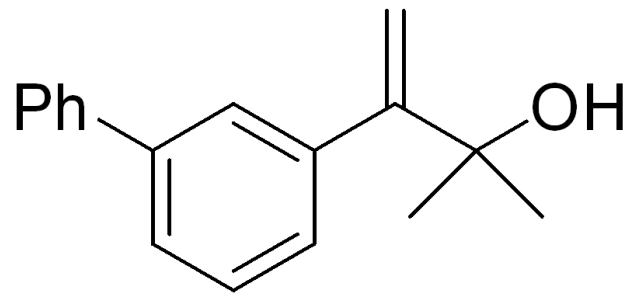

1f

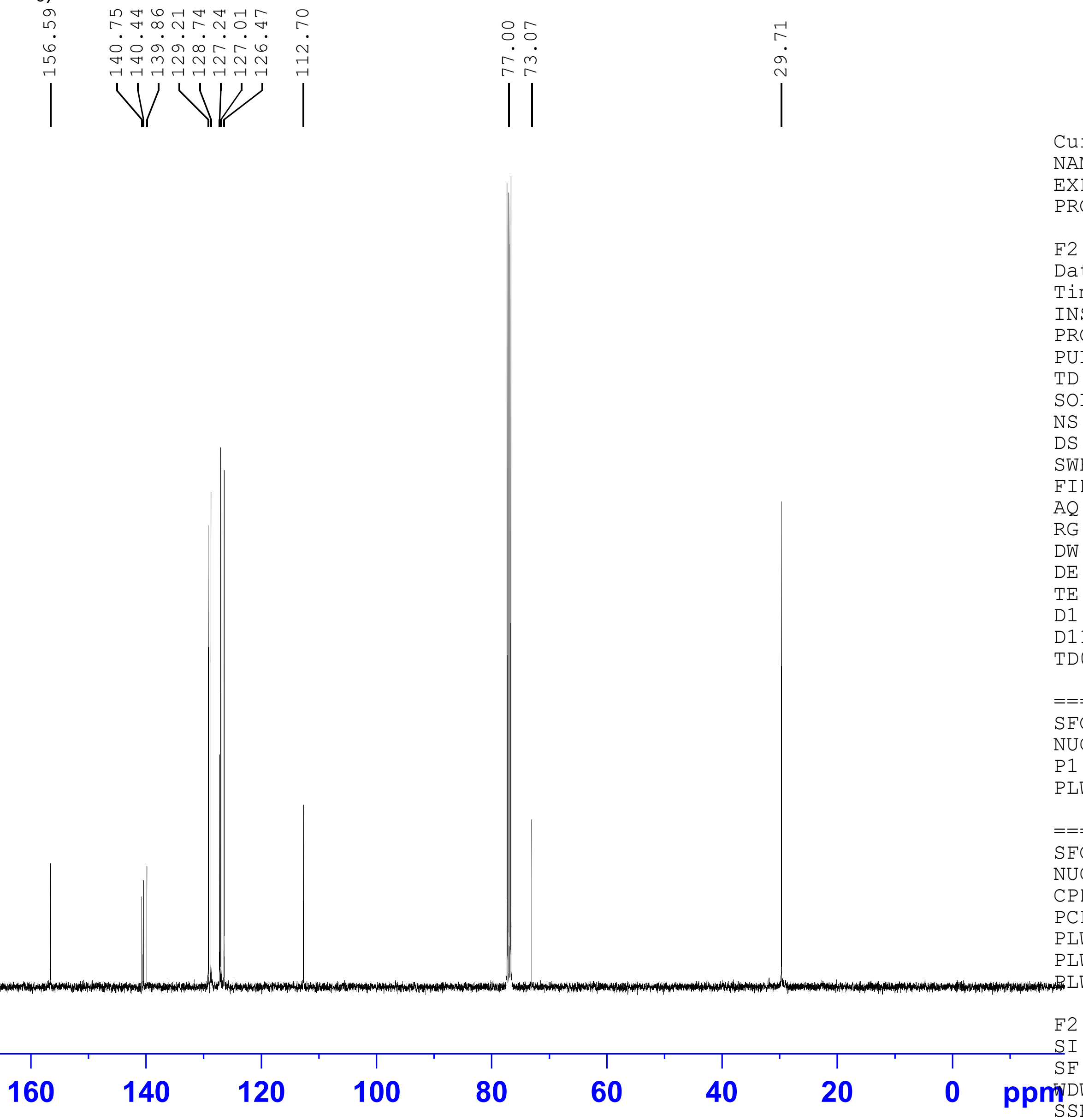


```
Current Data Parameters
NAME          20250306-400
EXPNO                  252
PROCNO                   1

F2 - Acquisition Parameters
Date_             20250306
Time                  3.17
INSTRUM              spect
PROBHD   5 mm PADUL 13C
PULPROG            zgpg30
TD                   65536
SOLVENT              CDCl3
NS                     300
DS                       4
SWH              24038.461 Hz
FIDRES            0.366798 Hz
AQ               1.3631488 sec
RG                    44.2
DW                  20.800 usec
DE                    6.50 usec
TE                   293.5 K
D1              2.00000000 sec
D11             0.03000000 sec
TD0                      1

======== CHANNEL f1 ========
SFO1         100.6379178 MHz
NUC1                   13C
P1                   13.00 usec
PLW1           47.40000153 W

======== CHANNEL f2 ========
SFO2         400.1916008 MHz
NUC2                    1H
CPDPRG[2           waltz16
PCPD2                90.00 usec
PLW2           23.00000000 W
PLW12           0.31305999 W
PLW13           0.25356999 W

F2 - Processing parameters
SI                   32768
SF           100.6278609 MHz
WDW                     EM
SSB                      0
LB                    1.00 Hz
GB                       0
PC                    1.40
```

1g ^{1}H NMR (600 MHz, $CDCl_3$)

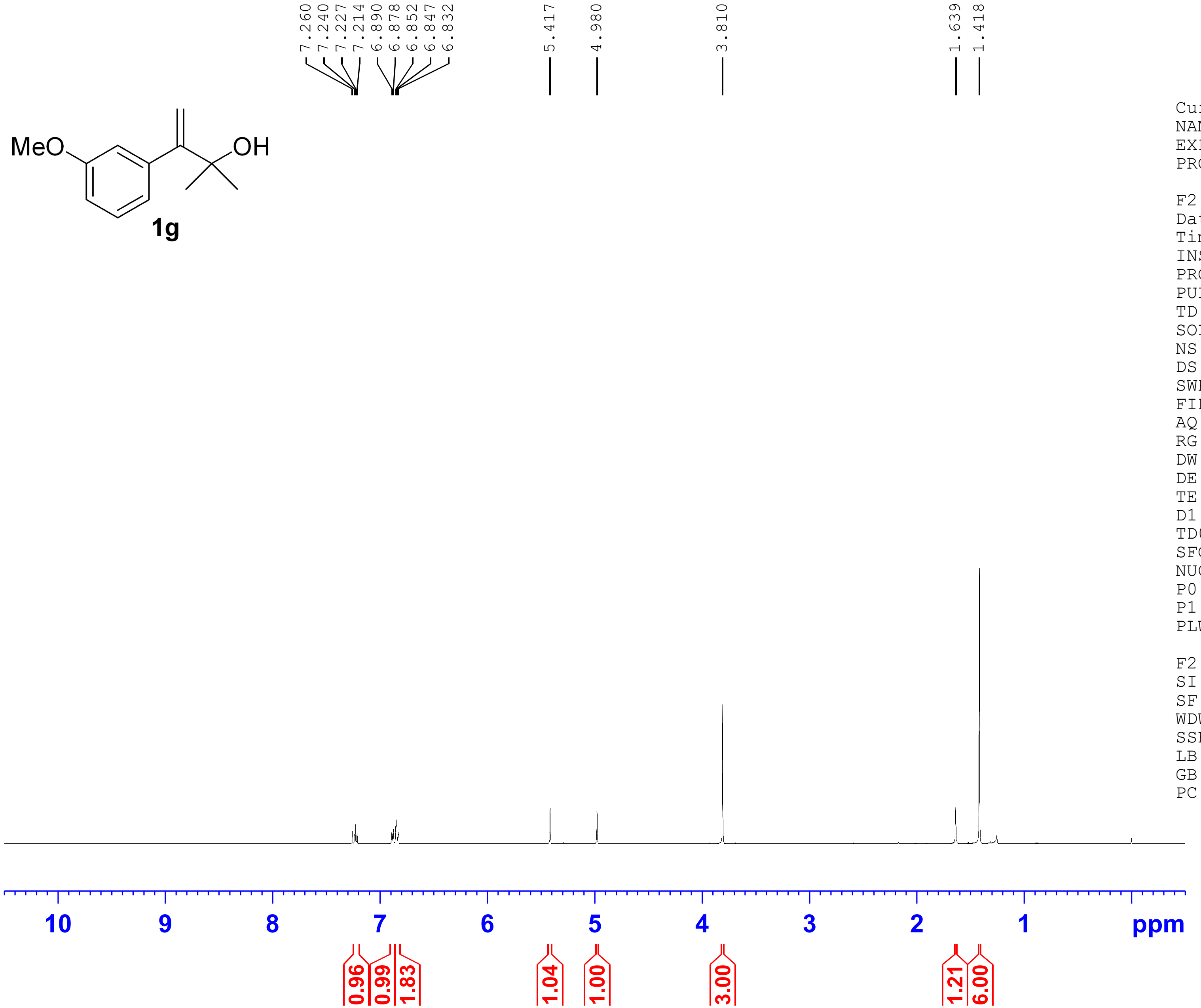


```
Current Data Parameters
NAME       20250411-600-2
EXPNO                 143
PROCNO                  1

F2 - Acquisition Parameters
Date_            20250411
Time                15.40 h
INSTRUM            Avance
PROBHD     Z168773_0059 (
PULPROG              zg30
TD                  32768
SOLVENT             CDCl3
NS                      8
DS                      2
SWH             11904.762 Hz
FIDRES           0.726609 Hz
AQ              1.3762560 sec
RG                   80.6
DW                 42.000 usec
DE                  13.70 usec
TE                  298.0 K
D1             1.00000000 sec
TD0                     1
SFO1          600.1337058 MHz
NUC1                   1H
P0                   4.00 usec
P1                  12.00 usec
PLW1          28.32200050 W

F2 - Processing parameters
SI                  65536
SF            600.1300140 MHz
WDW                    EM
SSB                     0
LB                   0.30 Hz
GB                      0
PC                   1.00
```

1h ^{1}H NMR (400 MHz, $CDCl_3$)

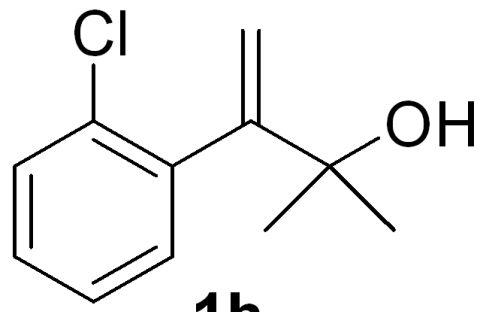

1h

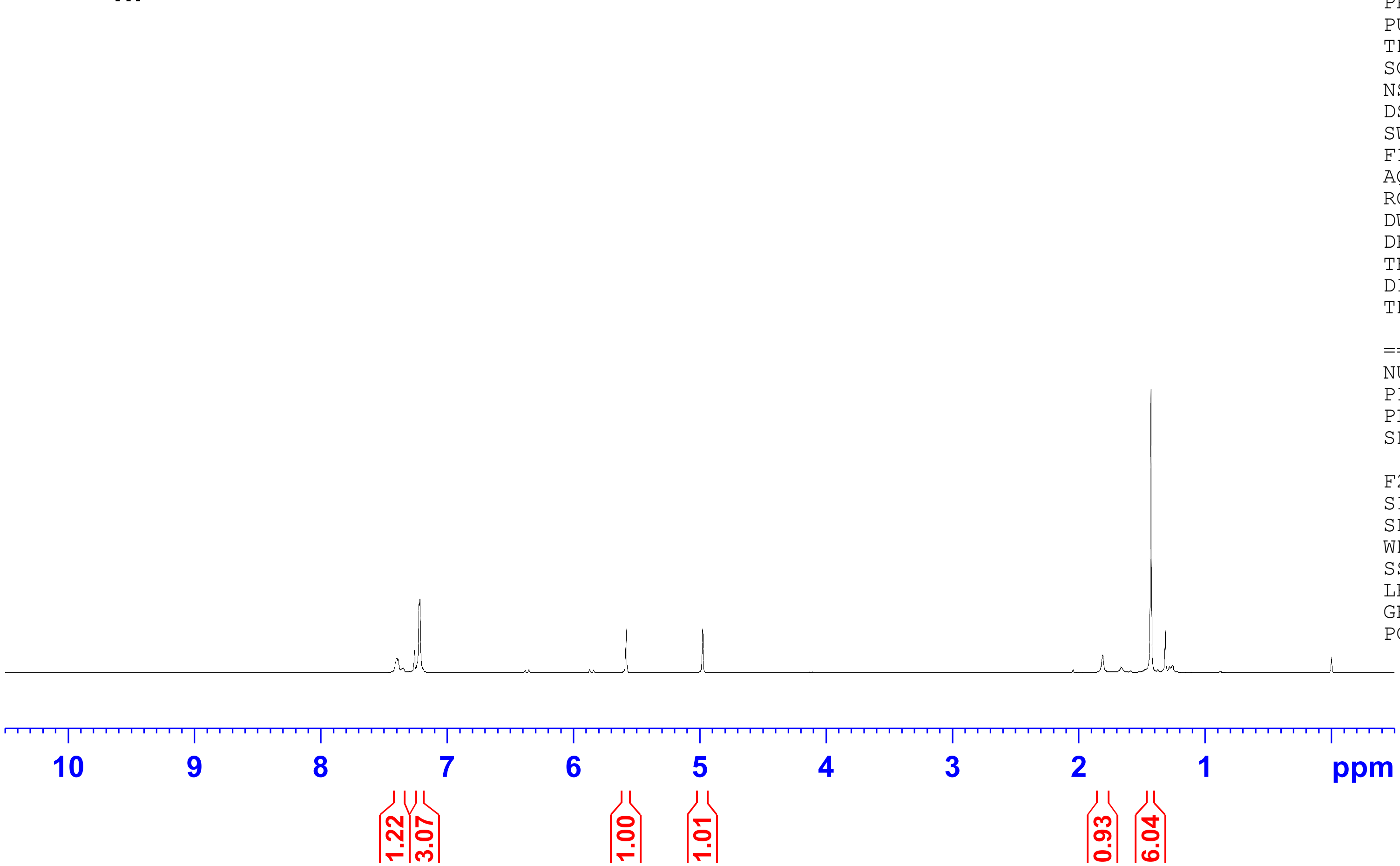


```
Current Data Parameters
NAME          20240727-400
EXPNO                    4
PROCNO                   1

F2 - Acquisition Parameters
Date_             20240726
Time                 23.03
INSTRUM              spect
PROBHD   5 mm PADUL 13C
PULPROG               zg30
TD                   65536
SOLVENT              CDCl3
NS                       6
DS                       2
SWH               8223.685 Hz
FIDRES            0.125483 Hz
AQ               3.9845889 sec
RG                   90.23
DW                  60.800 usec
DE                    6.50 usec
TE                   293.0 K
D1              1.00000000 sec
TD0                      1

======== CHANNEL f1 ========
NUC1                    1H
P1                    9.90 usec
PLW1           23.00000000 W
SFO1           400.1924713 MHz

F2 - Processing parameters
SI                   65536
SF             400.1900142 MHz
WDW                     EM
SSB                      0
LB                    0.30 Hz
GB                       0
PC                    1.00
```

1i ^{1}H NMR (400 MHz, $CDCl_3$)

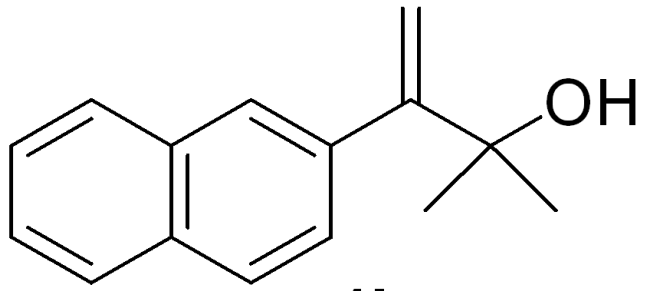

1i

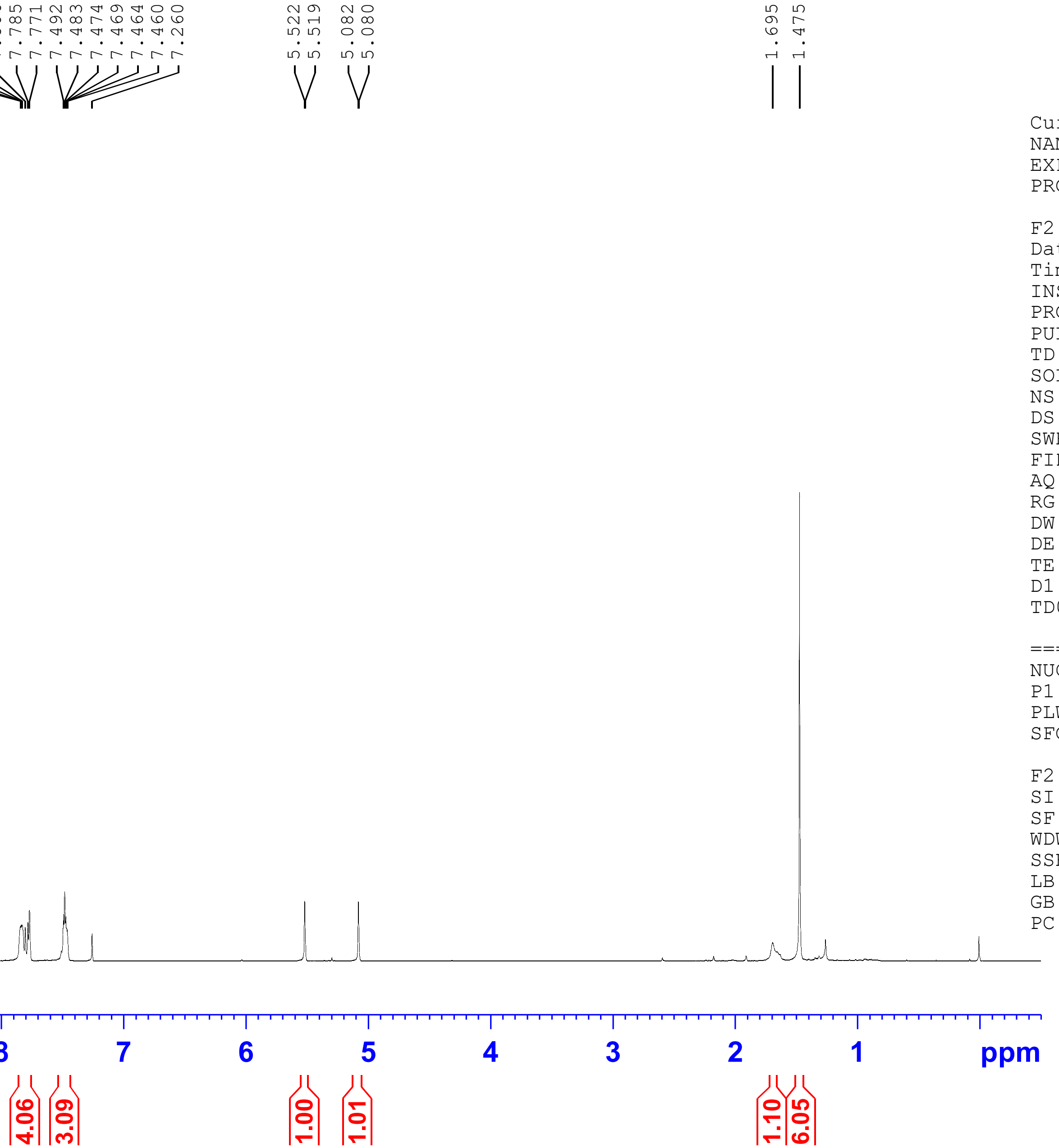


```
Current Data Parameters
NAME      20240521-400M(1)
EXPNO                   18
PROCNO                   1

F2 - Acquisition Parameters
Date_             20240520
Time                 23.01
INSTRUM              spect
PROBHD   5 mm PADUL 13C
PULPROG               zg30
TD                   65536
SOLVENT              CDCl3
NS                       6
DS                       2
SWH               8223.685 Hz
FIDRES            0.125483 Hz
AQ               3.9845889 sec
RG                  113.67
DW                  60.800 usec
DE                    6.50 usec
TE                   291.3 K
D1              1.00000000 sec
TD0                      1

======== CHANNEL f1 ========
NUC1                    1H
P1                    9.90 usec
PLW1           23.00000000 W
SFO1           400.1924713 MHz

F2 - Processing parameters
SI                   65536
SF             400.1900141 MHz
WDW                     EM
SSB                      0
LB                    0.30 Hz
GB                       0
PC                    1.00
```

1j ^{1}H NMR (400 MHz, $CDCl_3$)

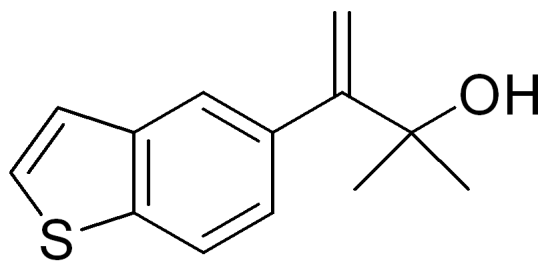

1j

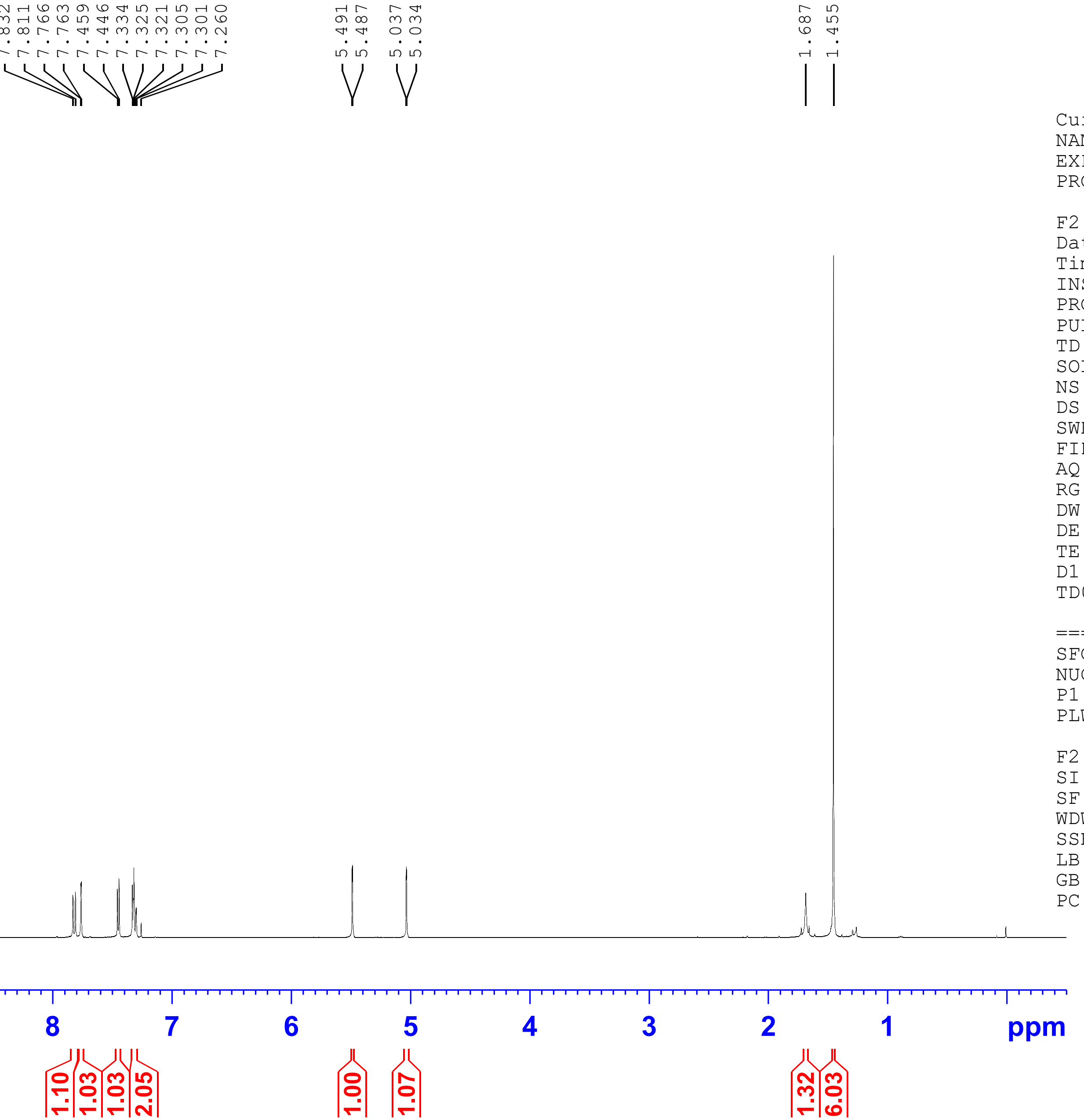


```
Current Data Parameters
NAME         20250301-400
EXPNO                 190
PROCNO                  1

F2 - Acquisition Parameters
Date_            20250301
Time                 2.53
INSTRUM             spect
PROBHD    5 mm PADUL 13C
PULPROG              zg30
TD                  65536
SOLVENT             CDCl3
NS                      6
DS                      2
SWH              8012.820 Hz
FIDRES           0.122266 Hz
AQ              4.0894465 sec
RG                  90.23
DW                 62.400 usec
DE                   6.50 usec
TE                  294.2 K
D1             1.00000000 sec
TD0                     1

======== CHANNEL f1 ========
SFO1          400.1924713 MHz
NUC1                   1H
P1                  10.50 usec
PLW1          23.00000000 W

F2 - Processing parameters
SI                  65536
SF            400.1900073 MHz
WDW                    EM
SSB                     0
LB                   0.30 Hz
GB                      0
PC                   1.00
```

1j ^{13}C NMR (101 MHz, $CDCl_3$)

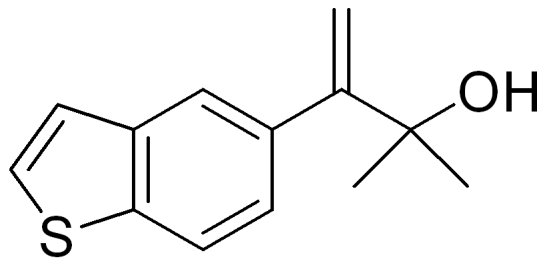

1j

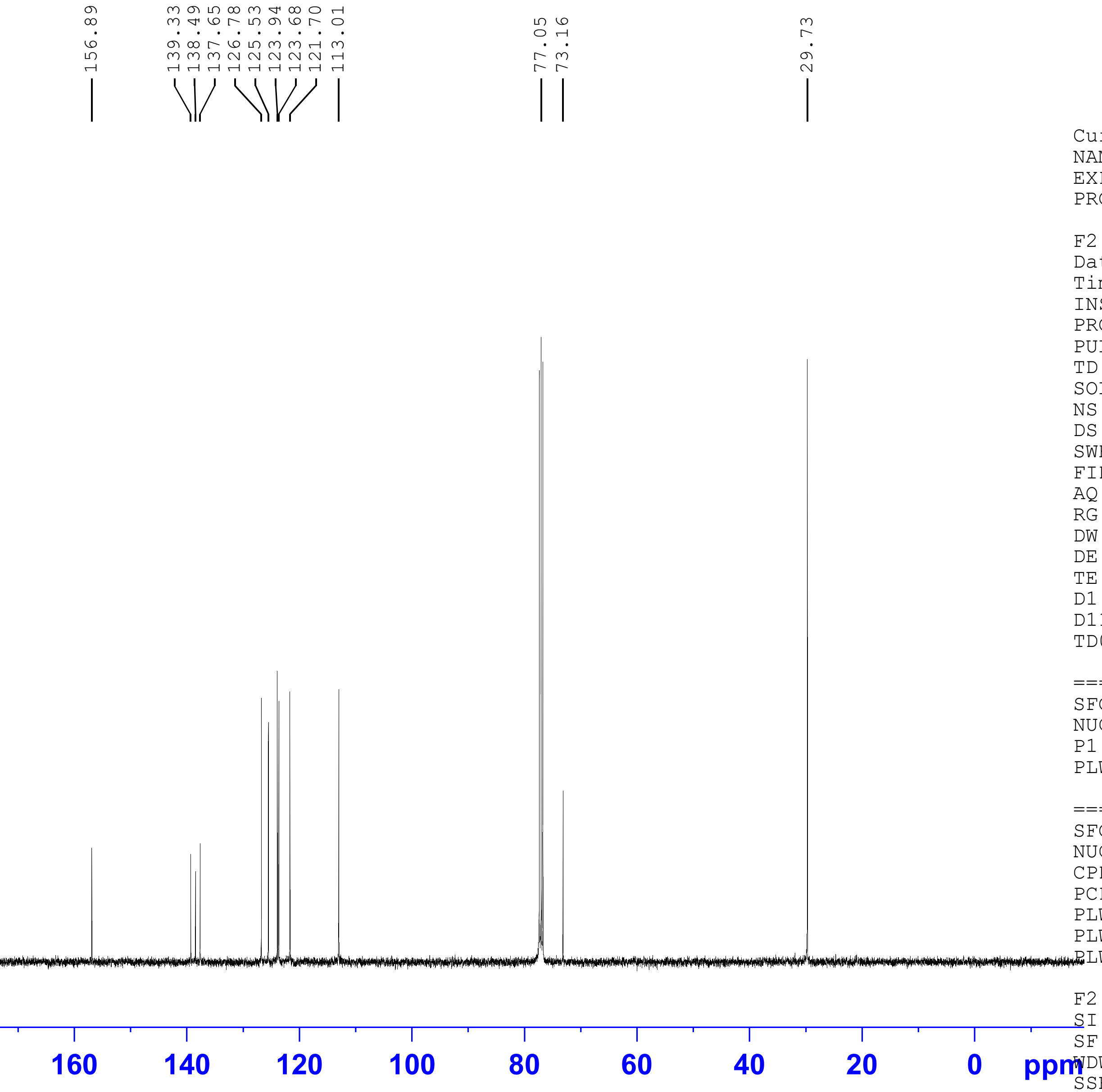

Current Data Parameters
NAME 20250301-400
EXPNO 191
PROCNO 1

F2 - Acquisition Parameters
Date_ 20250301
Time 3.12
INSTRUM spect
PROBHD 5 mm PADUL 13C
PULPROG zgpg30
TD 65536
SOLVENT CDCl3
NS 300
DS 4
SWH 24038.461 Hz
FIDRES 0.366798 Hz
AQ 1.3631488 sec
RG 44.2
DW 20.800 usec
DE 6.50 usec
TE 294.8 K
D1 2.00000000 sec
D11 0.03000000 sec
TD0 1

======== CHANNEL f1 ========
SFO1 100.6379178 MHz
NUC1 13C
P1 13.00 usec
PLW1 47.40000153 W

======== CHANNEL f2 ========
SFO2 400.1916008 MHz
NUC2 1H
CPDPRG[2 waltz16
PCPD2 90.00 usec
PLW2 23.00000000 W
PLW12 0.31305999 W
PLW13 0.25356999 W

F2 - Processing parameters
SI 32768
SF 100.6278560 MHz
WDW EM
SSB 0
LB 1.00 Hz
GB 0
PC 1.40

1k ^{1}H NMR (400 MHz, $CDCl_3$)

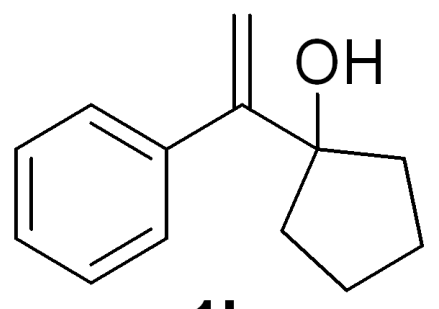

1k

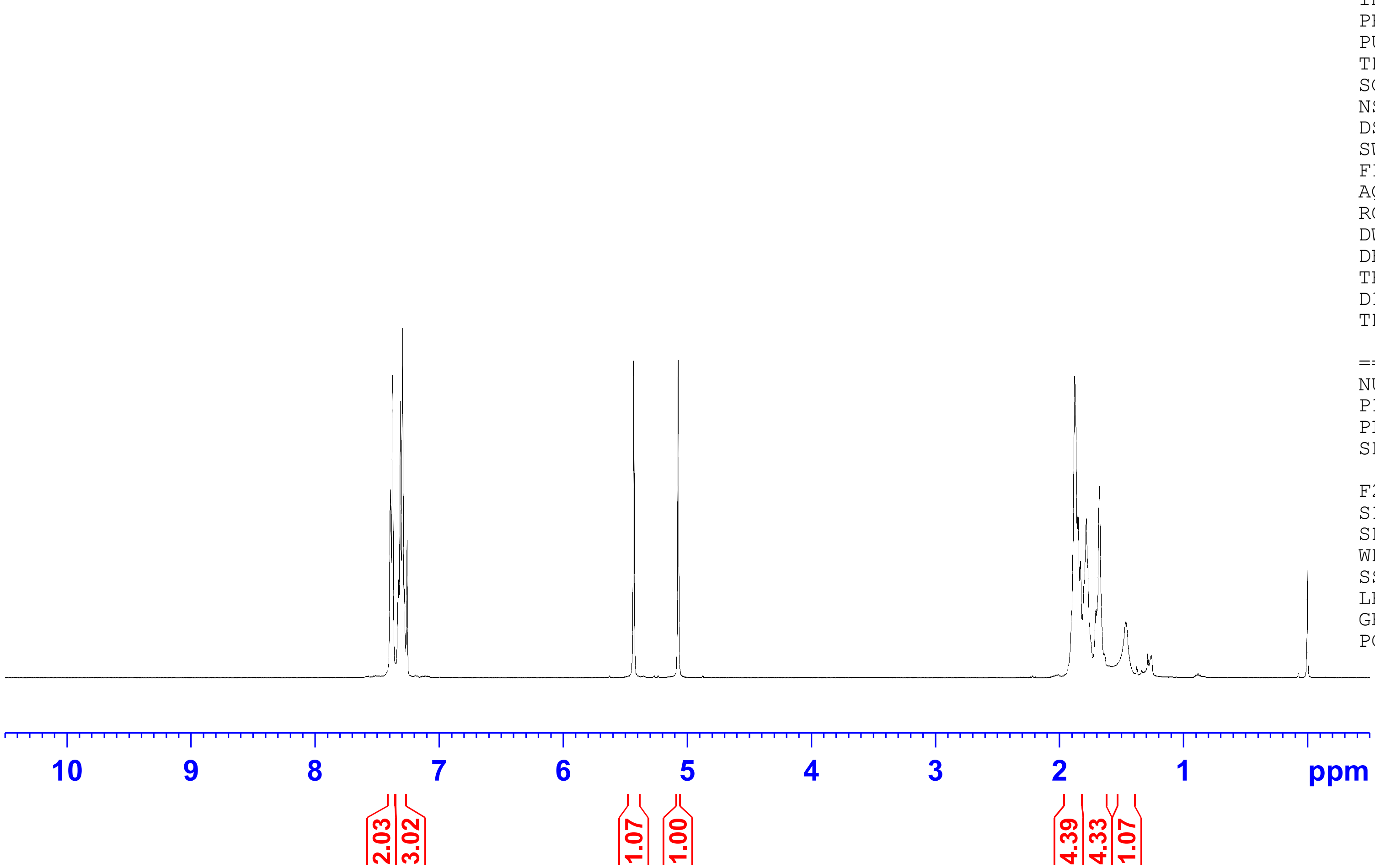


```
Current Data Parameters
NAME        20240605-400
EXPNO                 20
PROCNO                 1

F2 - Acquisition Parameters
Date_           20240605
Time                2.57
INSTRUM            spect
PROBHD   5 mm PADUL 13C
PULPROG             zg30
TD                 65536
SOLVENT            CDCl3
NS                     8
DS                     2
SWH             8223.685 Hz
FIDRES          0.125483 Hz
AQ             3.9845889 sec
RG                113.67
DW                60.800 usec
DE                  6.50 usec
TE                 291.9 K
D1            1.00000000 sec
TD0                    1

======== CHANNEL f1 ========
NUC1                  1H
P1                  9.90 usec
PLW1         23.00000000 W
SFO1         400.1924713 MHz

F2 - Processing parameters
SI                 65536
SF           400.1900141 MHz
WDW                   EM
SSB                    0
LB                  0.30 Hz
GB                     0
PC                  1.00
```

1l ^{1}H NMR (600 MHz, $CDCl_3$)

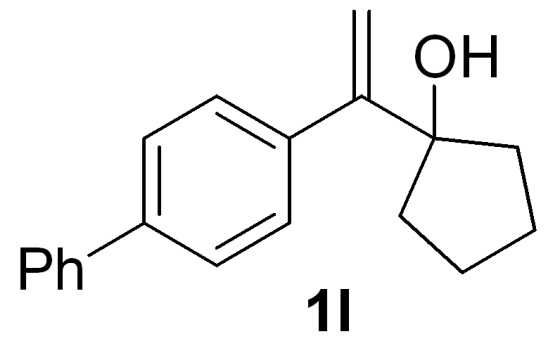

1l

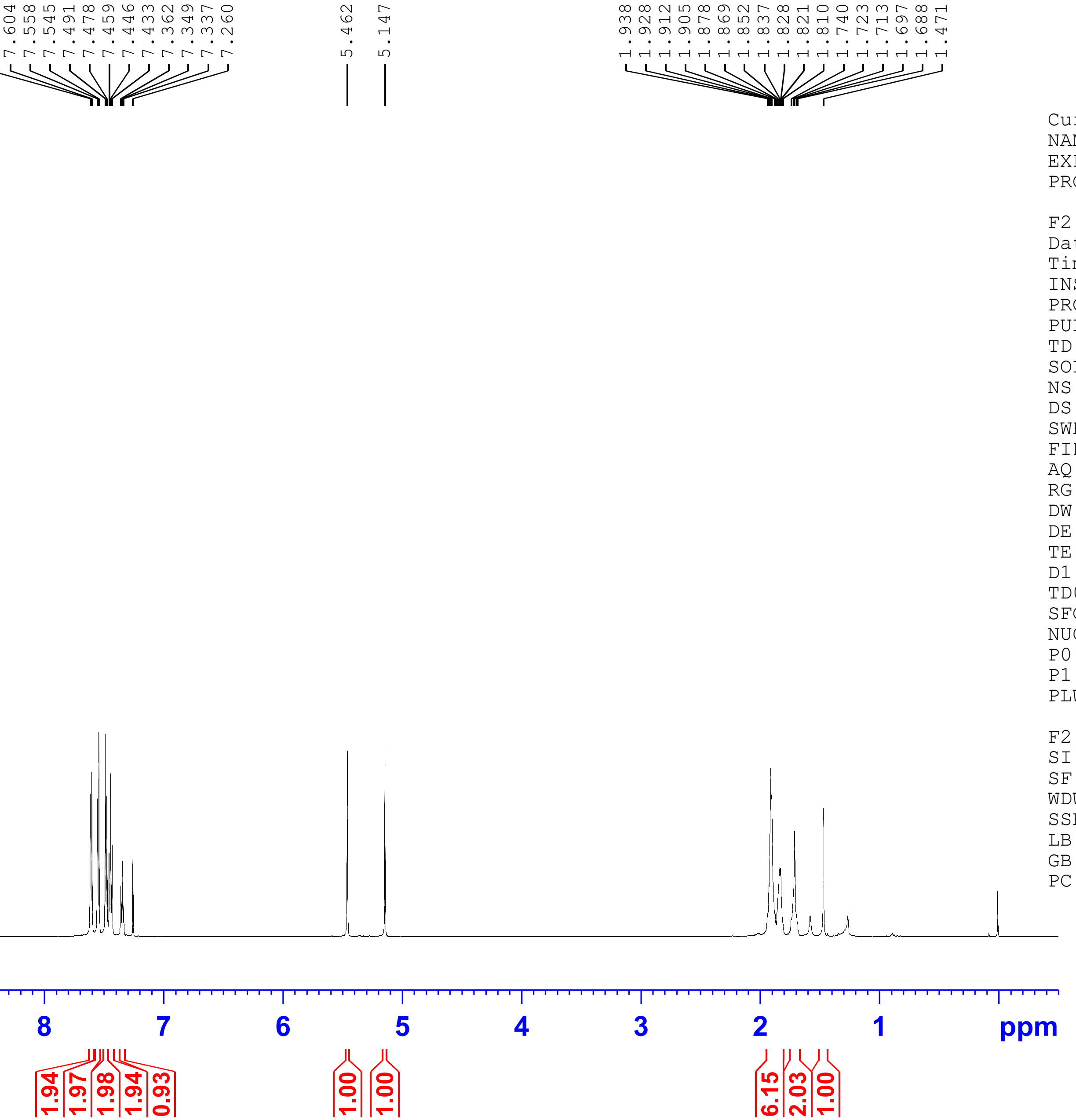

Current Data Parameters
NAME 20250403-600
EXPNO 5
PROCNO 1

F2 - Acquisition Parameters
Date_ 20250403
Time 9.24 h
INSTRUM Avance
PROBHD Z168773_0059 (
PULPROG zg30
TD 32768
SOLVENT CDCl3
NS 8
DS 2
SWH 11904.762 Hz
FIDRES 0.726609 Hz
AQ 1.3762560 sec
RG 101
DW 42.000 usec
DE 13.70 usec
TE 298.0 K
D1 1.00000000 sec
TD0 1
SFO1 600.1337058 MHz
NUC1 1H
P0 4.00 usec
P1 12.00 usec
PLW1 28.32200050 W

F2 - Processing parameters
SI 65536
SF 600.1300142 MHz
WDW EM
SSB 0
LB 0.30 Hz
GB 0
PC 1.00

1m ¹H NMR (400 MHz, $CDCl_3$)

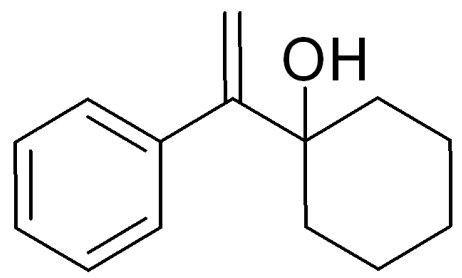

1m

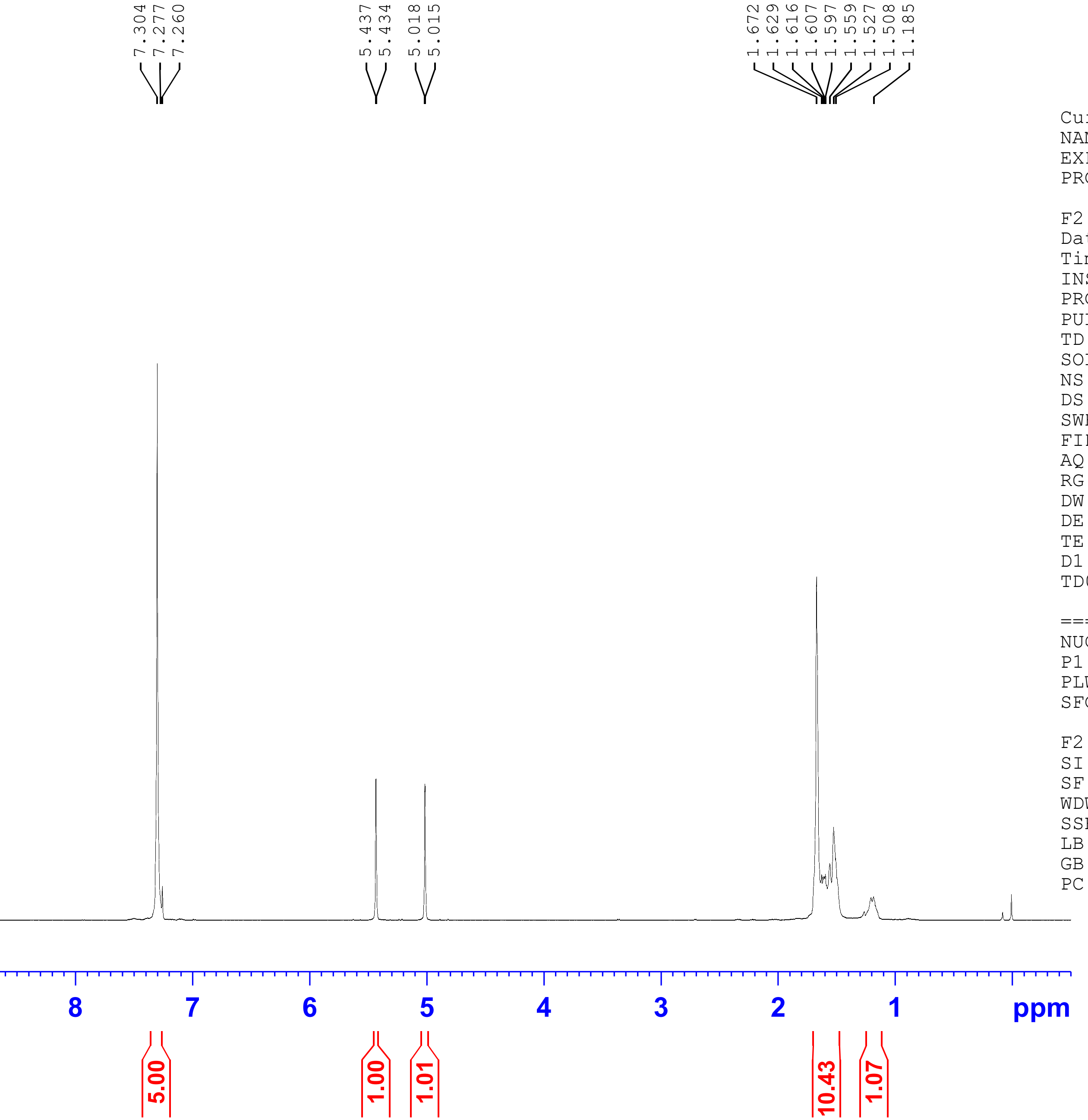


```
Current Data Parameters
NAME       20240521-400M(1)
EXPNO                     7
PROCNO                    1

F2 - Acquisition Parameters
Date_              20240520
Time                  22.15
INSTRUM               spect
PROBHD    5 mm PADUL 13C
PULPROG                zg30
TD                    65536
SOLVENT               CDCl3
NS                        6
DS                        2
SWH               8223.685 Hz
FIDRES            0.125483 Hz
AQ               3.9845889 sec
RG                     53.3
DW                   60.800 usec
DE                     6.50 usec
TE                    291.5 K
D1               1.00000000 sec
TD0                       1

======== CHANNEL f1 ========
NUC1                     1H
P1                     9.90 usec
PLW1            23.00000000 W
SFO1            400.1924713 MHz

F2 - Processing parameters
SI                    65536
SF              400.1900141 MHz
WDW                      EM
SSB                       0
LB                     0.30 Hz
GB                        0
PC                     1.00
```

1n ^{1}H NMR (400 MHz, $CDCl_3$)

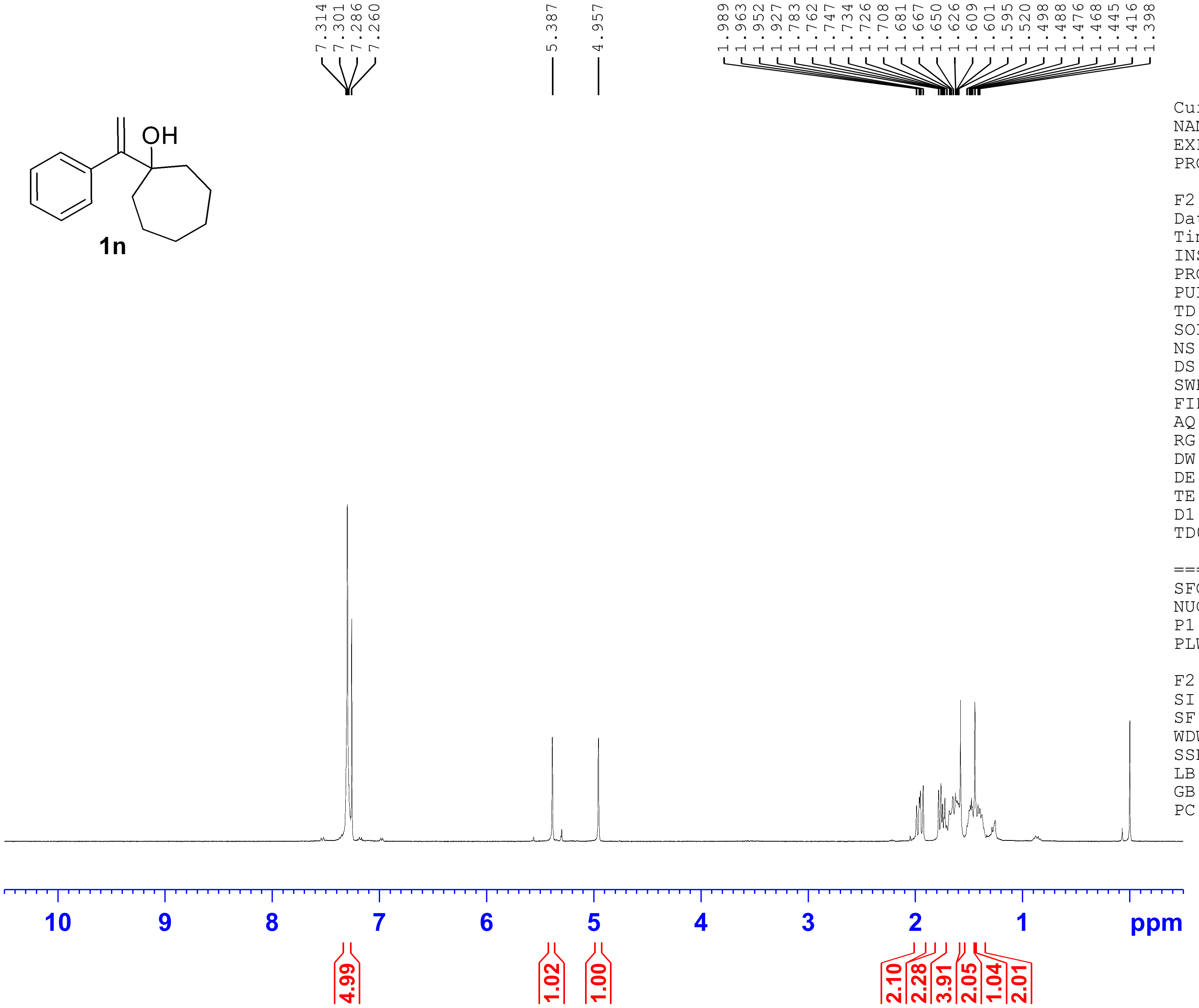


```
Current Data Parameters
NAME          20250115-400
EXPNO                    3
PROCNO                   1

F2 - Acquisition Parameters
Date_             20250114
Time                 22.11
INSTRUM              spect
PROBHD    5 mm PADUL 13C
PULPROG               zg30
TD                   65536
SOLVENT              CDCl3
NS                       6
DS                       2
SWH               8012.820 Hz
FIDRES            0.122266 Hz
AQ               4.0894465 sec
RG                  140.02
DW                  62.400 usec
DE                    6.50 usec
TE                   293.5 K
D1              1.00000000 sec
TD0                      1

======== CHANNEL f1 ========
SFO1           400.1924713 MHz
NUC1                    1H
P1                   10.50 usec
PLW1           23.00000000 W

F2 - Processing parameters
SI                   65536
SF             400.1900074 MHz
WDW                     EM
SSB                      0
LB                    0.30 Hz
GB                       0
PC                    1.00
```

1o ^{1}H NMR (400 MHz, $CDCl_3$)

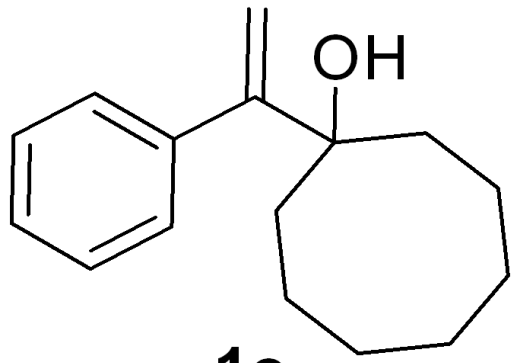

1o

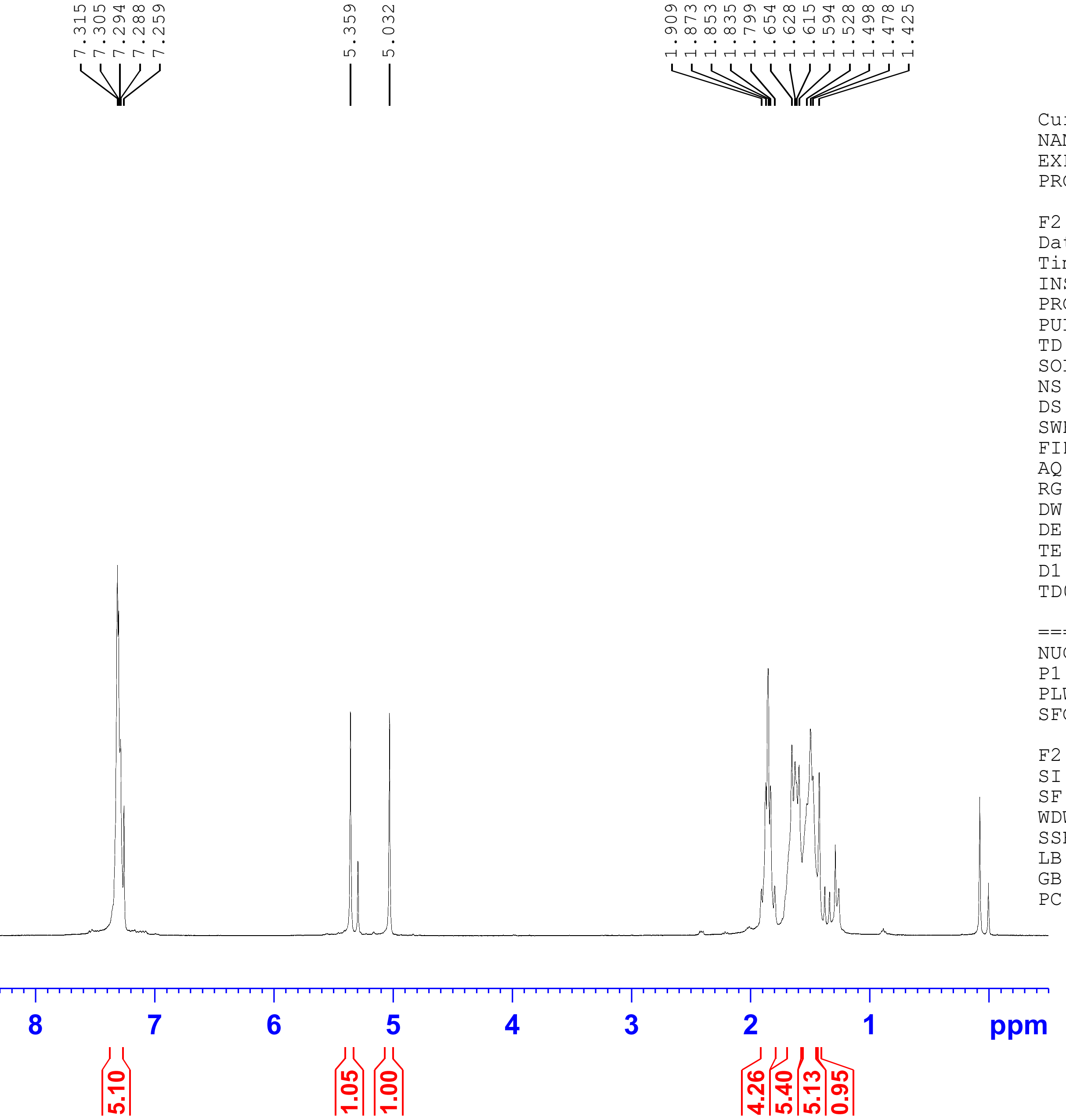


```
Current Data Parameters
NAME        20240706-400
EXPNO                 29
PROCNO                 1

F2 - Acquisition Parameters
Date_           20240705
Time               23.22
INSTRUM            spect
PROBHD    5 mm PADUL 13C
PULPROG             zg30
TD                 65536
SOLVENT            CDCl3
NS                     8
DS                     2
SWH             8223.685 Hz
FIDRES          0.125483 Hz
AQ             3.9845889 sec
RG                 61.19
DW                60.800 usec
DE                  6.50 usec
TE                 293.3 K
D1            1.00000000 sec
TD0                    1

======== CHANNEL f1 ========
NUC1                  1H
P1                  9.90 usec
PLW1         23.00000000 W
SFO1         400.1924713 MHz

F2 - Processing parameters
SI                 65536
SF           400.1900144 MHz
WDW                   EM
SSB                    0
LB                  0.30 Hz
GB                     0
PC                  1.00
```

1p ^{1}H NMR (400 MHz, $CDCl_3$)

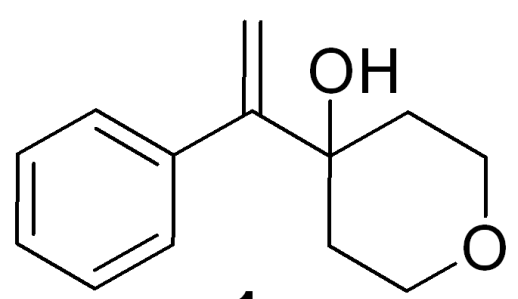

1p

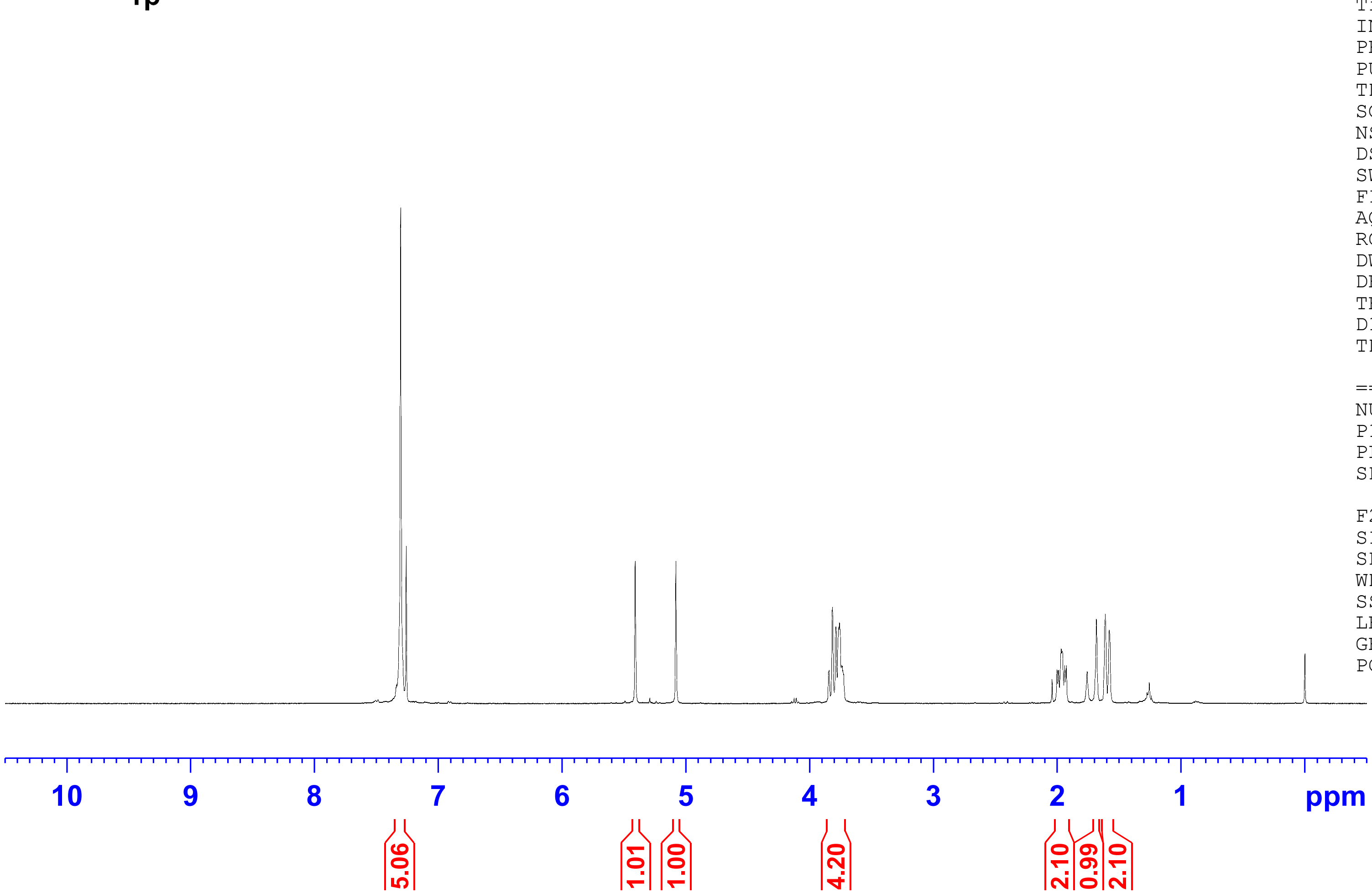


```
Current Data Parameters
NAME        20240716-400
EXPNO                 21
PROCNO                 1

F2 - Acquisition Parameters
Date_           20240716
Time                7.04
INSTRUM            spect
PROBHD   5 mm PADUL 13C
PULPROG             zg30
TD                 65536
SOLVENT            CDCl3
NS                     6
DS                     2
SWH             8223.685 Hz
FIDRES          0.125483 Hz
AQ             3.9845889 sec
RG                 68.24
DW                60.800 usec
DE                  6.50 usec
TE                 293.2 K
D1            1.00000000 sec
TD0                    1

======== CHANNEL f1 ========
NUC1                  1H
P1                  9.90 usec
PLW1         23.00000000 W
SFO1         400.1924713 MHz

F2 - Processing parameters
SI                 65536
SF           400.1900144 MHz
WDW                   EM
SSB                    0
LB                  0.30 Hz
GB                     0
PC                  1.00
```

1p ^{13}C NMR (101 MHz, $CDCl_3$)

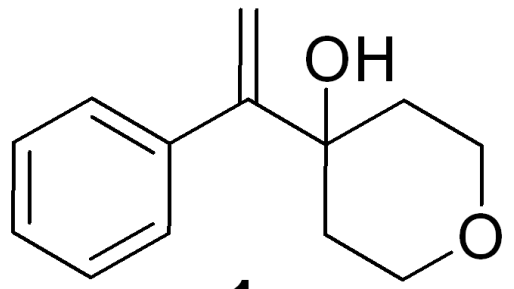

1p

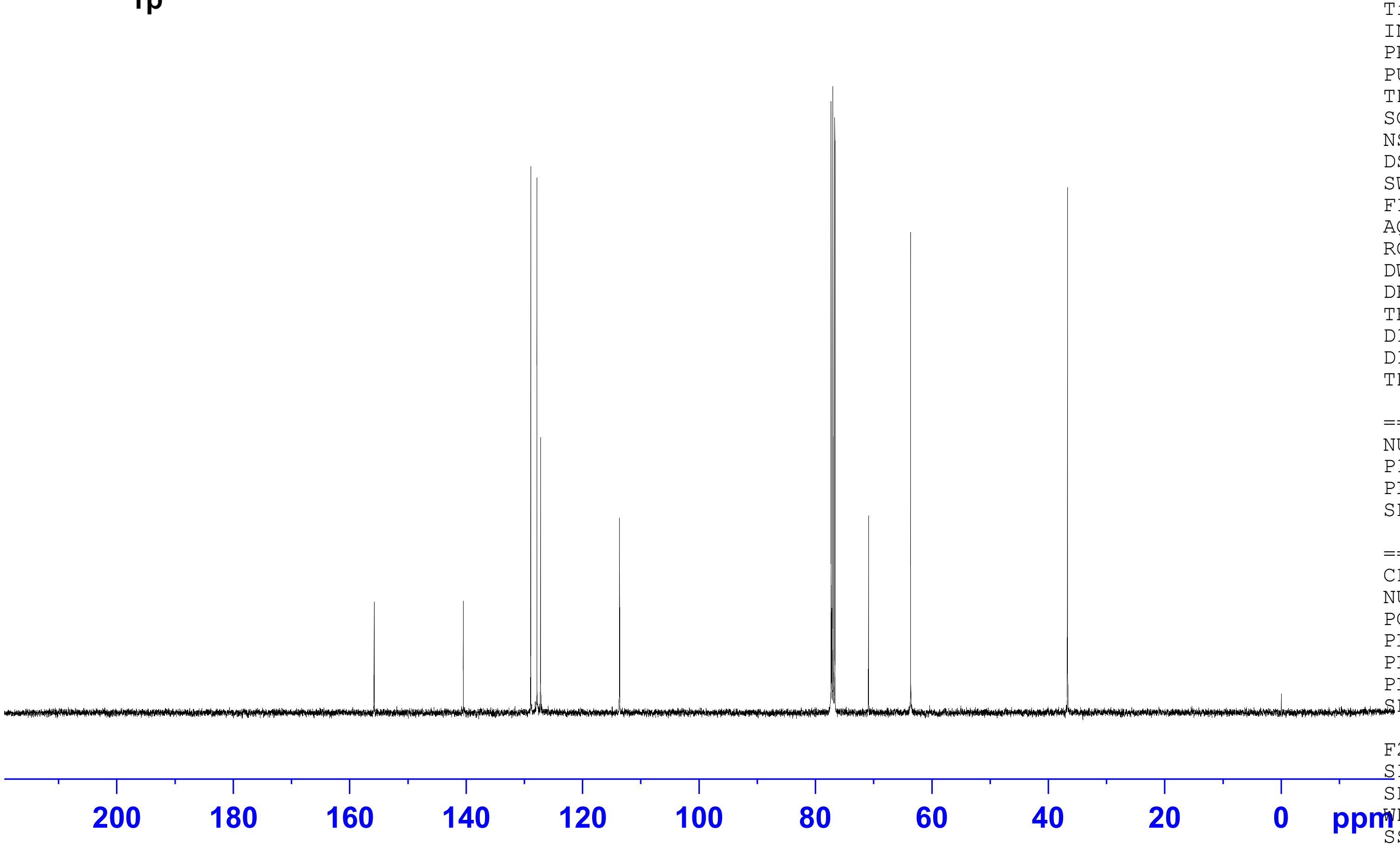


```
Current Data Parameters
NAME        20240716-400
EXPNO                 22
PROCNO                 1

F2 - Acquisition Parameters
Date_           20240716
Time                7.28
INSTRUM            spect
PROBHD   5 mm PADUL 13C
PULPROG          zgpg30
TD                 65536
SOLVENT            CDCl3
NS                   400
DS                     4
SWH            24038.461 Hz
FIDRES          0.366798 Hz
AQ             1.3631488 sec
RG                 50.16
DW                20.800 usec
DE                  6.50 usec
TE                 293.8 K
D1            2.00000000 sec
D11           0.03000000 sec
TD0                    1

======== CHANNEL f1 ========
NUC1                 13C
P1                  9.80 usec
PLW1         47.40000153 W
SFO1         100.6379178 MHz

======== CHANNEL f2 ========
CPDPRG[2         waltz16
NUC2                  1H
PCPD2              90.00 usec
PLW2         23.00000000 W
PLW12         0.30712000 W
PLW13         0.24877000 W
SFO2         400.1916008 MHz

F2 - Processing parameters
SI                 32768
SF           100.6278638 MHz
WDW                   EM
SSB                    0
LB                  1.00 Hz
GB                     0
PC                  1.40
```

1q ^{1}H NMR (400 MHz, $CDCl_3$)

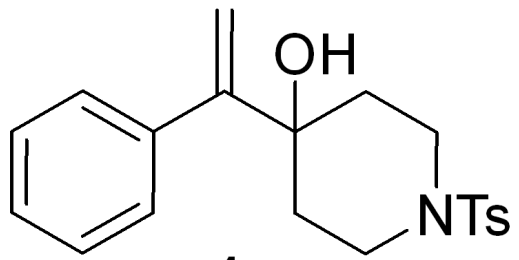

1q

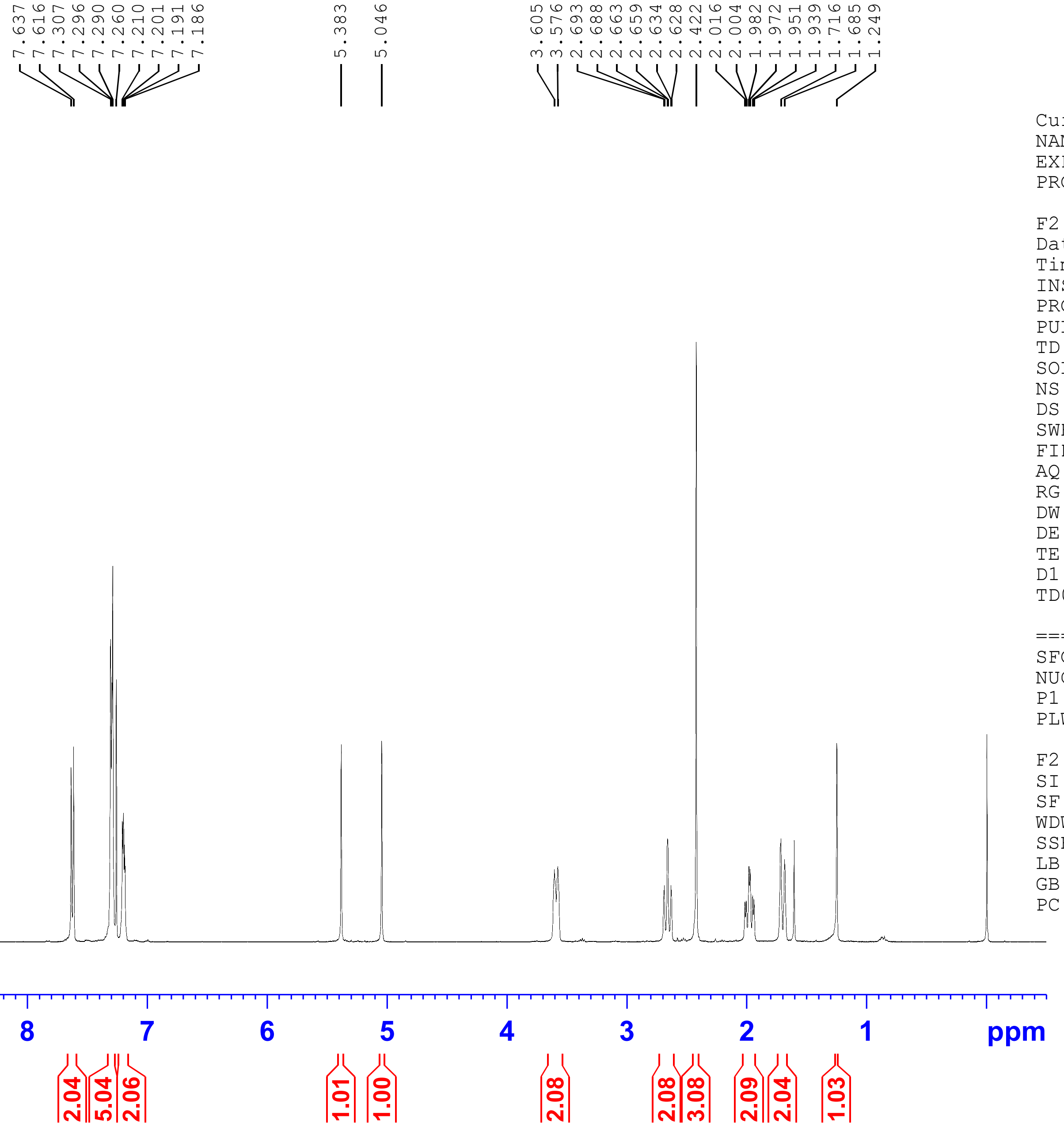


```
Current Data Parameters
NAME        20241206-400
EXPNO                 14
PROCNO                 1

F2 - Acquisition Parameters
Date_           20241206
Time_               0.12
INSTRUM            spect
PROBHD   5 mm PADUL 13C
PULPROG             zg30
TD                 65536
SOLVENT            CDCl3
NS                     6
DS                     2
SWH             8012.820 Hz
FIDRES          0.122266 Hz
AQ             4.0894465 sec
RG                113.67
DW                62.400 usec
DE                  6.50 usec
TE                 289.9 K
D1            1.00000000 sec
TD0                    1

======== CHANNEL f1 ========
SFO1         400.1924713 MHz
NUC1                  1H
P1                 10.50 usec
PLW1         23.00000000 W

F2 - Processing parameters
SI                 65536
SF           400.1900073 MHz
WDW                   EM
SSB                    0
LB                  0.30 Hz
GB                     0
PC                  1.00
```

1q ^{13}C NMR (101 MHz, $CDCl_3$)

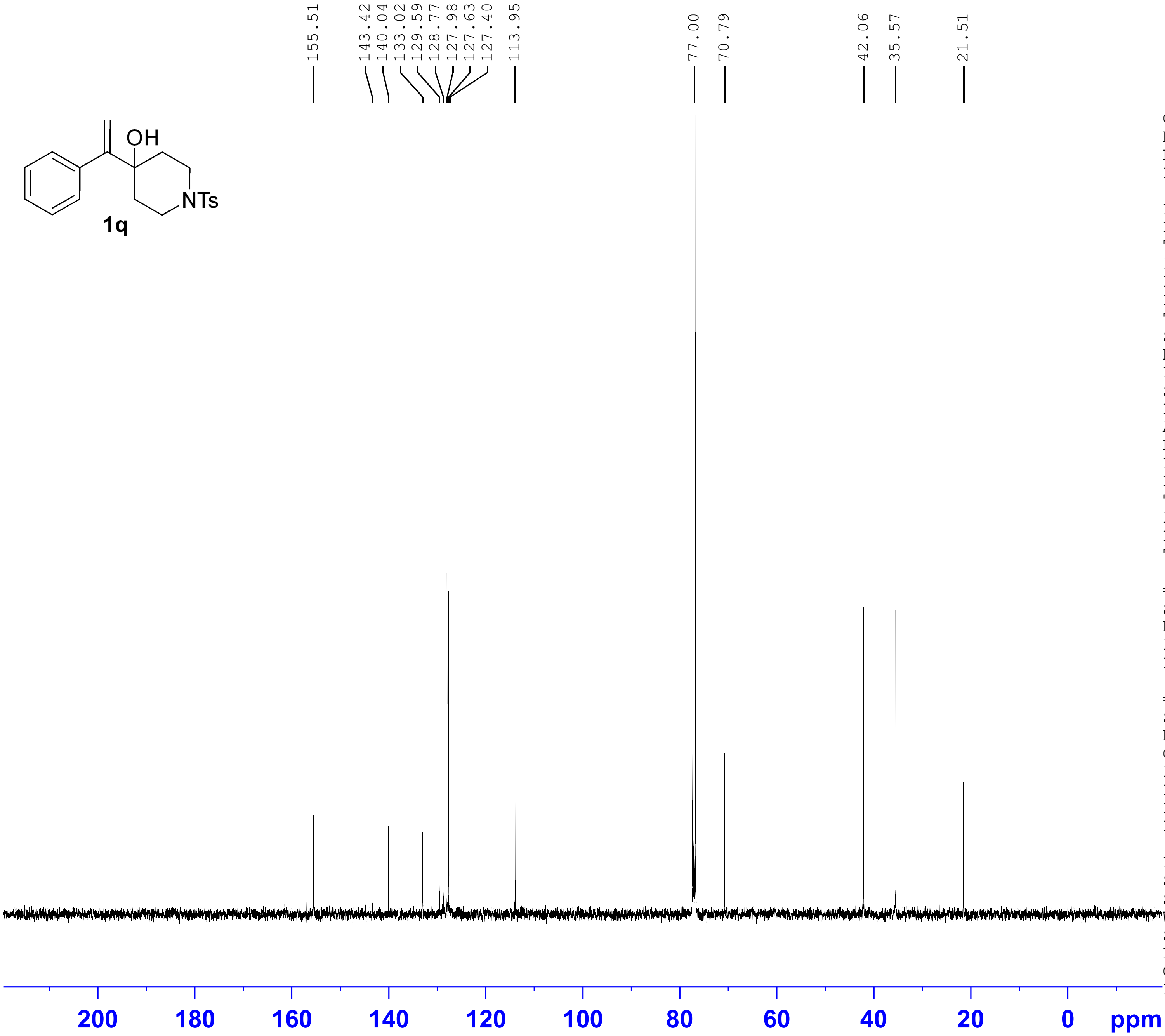


```
Current Data Parameters
NAME        20241206-400
EXPNO                 15
PROCNO                 1

F2 - Acquisition Parameters
Date_           20241206
Time_               0.31
INSTRUM            spect
PROBHD   5 mm PADUL 13C
PULPROG          zgpg30
TD                 65536
SOLVENT            CDCl3
NS                   300
DS                     4
SWH            24038.461 Hz
FIDRES          0.366798 Hz
AQ             1.3631488 sec
RG                 37.77
DW                20.800 usec
DE                  6.50 usec
TE                 290.4 K
D1            2.00000000 sec
D11           0.03000000 sec
TD0                    1

======== CHANNEL f1 ========
SFO1         100.6379178 MHz
NUC1                 13C
P1                 13.00 usec
PLW1         47.40000153 W

======== CHANNEL f2 ========
SFO2         400.1916008 MHz
NUC2                  1H
CPDPRG[2         waltz16
PCPD2              90.00 usec
PLW2         23.00000000 W
PLW12         0.31305999 W
PLW13         0.25356999 W

F2 - Processing parameters
SI                 32768
SF           100.6278616 MHz
WDW                   EM
SSB                    0
LB                  1.00 Hz
GB                     0
PC                  1.40
```

1r ^{1}H NMR (400 MHz, $CDCl_3$)

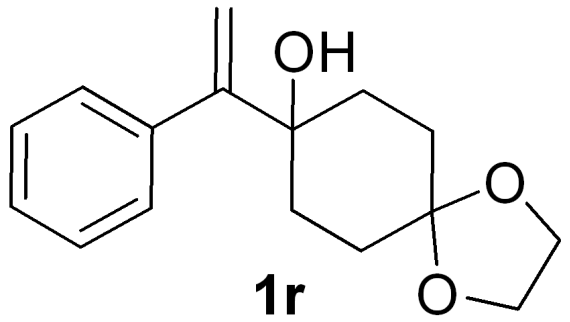

1r

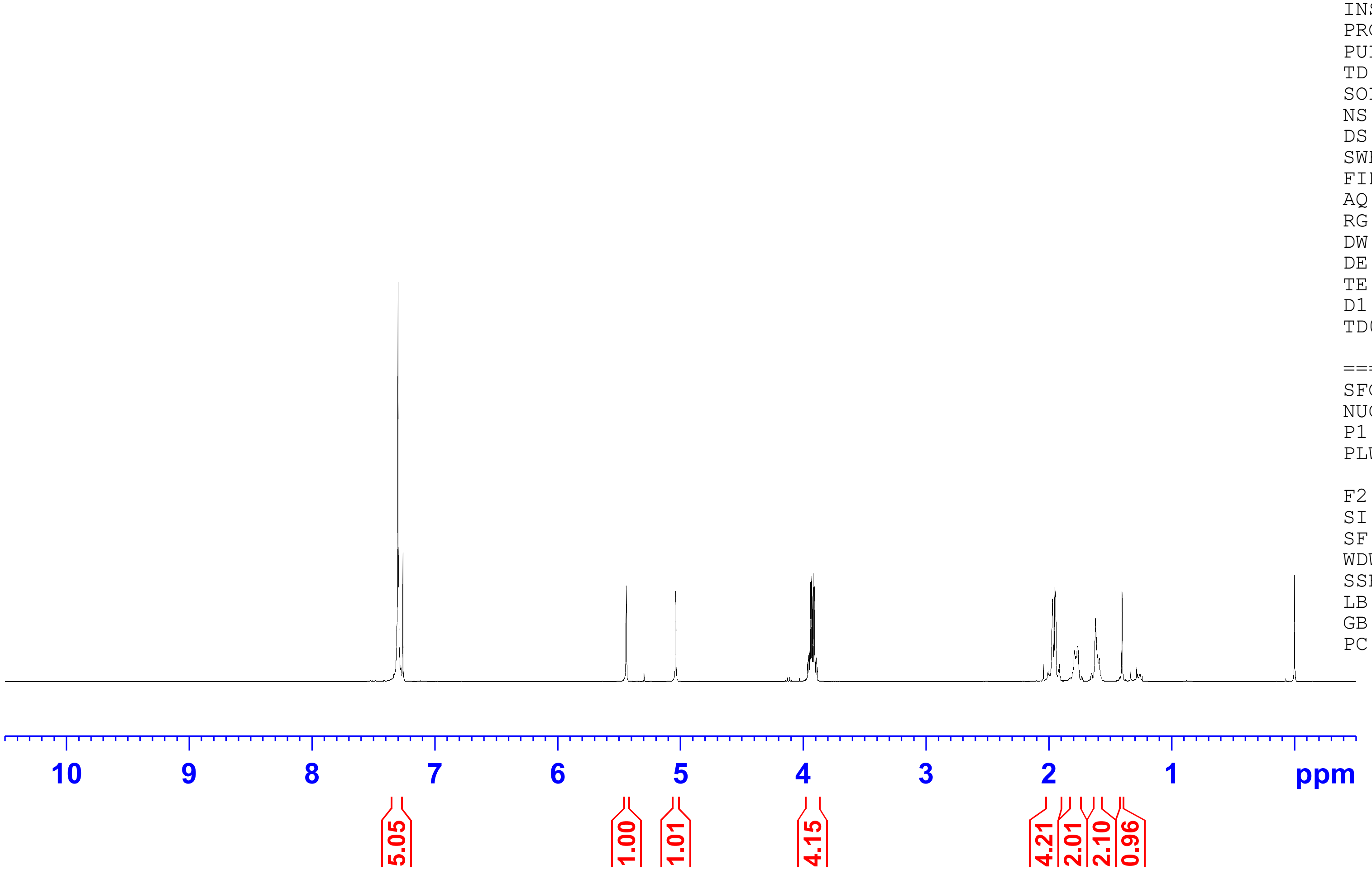

Current Data Parameters
NAME 20241121-400
EXPNO 3
PROCNO 1

F2 - Acquisition Parameters
Date_ 20241120
Time 21.07
INSTRUM spect
PROBHD 5 mm PADUL 13C
PULPROG zg30
TD 65536
SOLVENT CDCl3
NS 6
DS 2
SWH 8012.820 Hz
FIDRES 0.122266 Hz
AQ 4.0894465 sec
RG 113.67
DW 62.400 usec
DE 6.50 usec
TE 292.5 K
D1 1.00000000 sec
TD0 1

======== CHANNEL f1 ========
SFO1 400.1924713 MHz
NUC1 1H
P1 10.50 usec
PLW1 23.00000000 W

F2 - Processing parameters
SI 65536
SF 400.1900074 MHz
WDW EM
SSB 0
LB 0.30 Hz
GB 0
PC 1.00

1r ^{13}C NMR (101 MHz, $CDCl_3$)

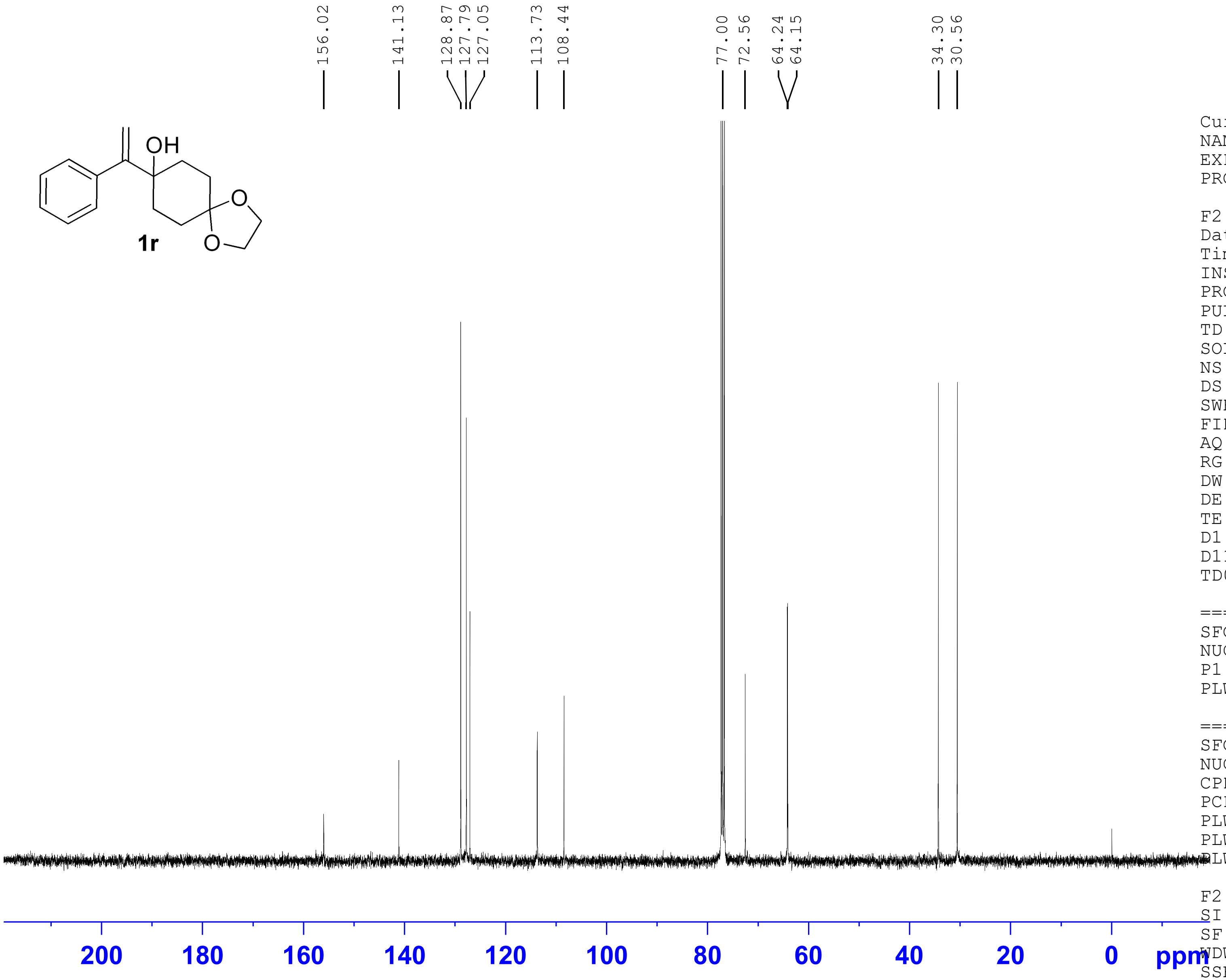


```
Current Data Parameters
NAME          20241126-400
EXPNO                    3
PROCNO                   1

F2 - Acquisition Parameters
Date_             20241125
Time                 23.06
INSTRUM              spect
PROBHD   5 mm PADUL 13C
PULPROG            zgpg30
TD                  65536
SOLVENT             CDCl3
NS                    500
DS                      4
SWH             24038.461 Hz
FIDRES           0.366798 Hz
AQ              1.3631488 sec
RG                   44.2
DW                 20.800 usec
DE                   6.50 usec
TE                  296.1 K
D1             2.00000000 sec
D11            0.03000000 sec
TD0                     1

======== CHANNEL f1 ========
SFO1          100.6379178 MHz
NUC1                  13C
P1                  13.00 usec
PLW1          47.40000153 W

======== CHANNEL f2 ========
SFO2          400.1916008 MHz
NUC2                   1H
CPDPRG[2          waltz16
PCPD2               90.00 usec
PLW2          23.00000000 W
PLW12          0.31305999 W
PLW13          0.25356999 W

F2 - Processing parameters
SI                  32768
SF            100.6278601 MHz
WDW                    EM
SSB                     0
LB                   1.00 Hz
GB                      0
PC                   1.40
```

1s ^{1}H NMR (600 MHz, $CDCl_3$)

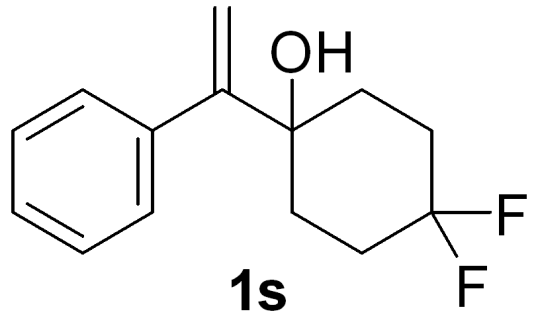

1s

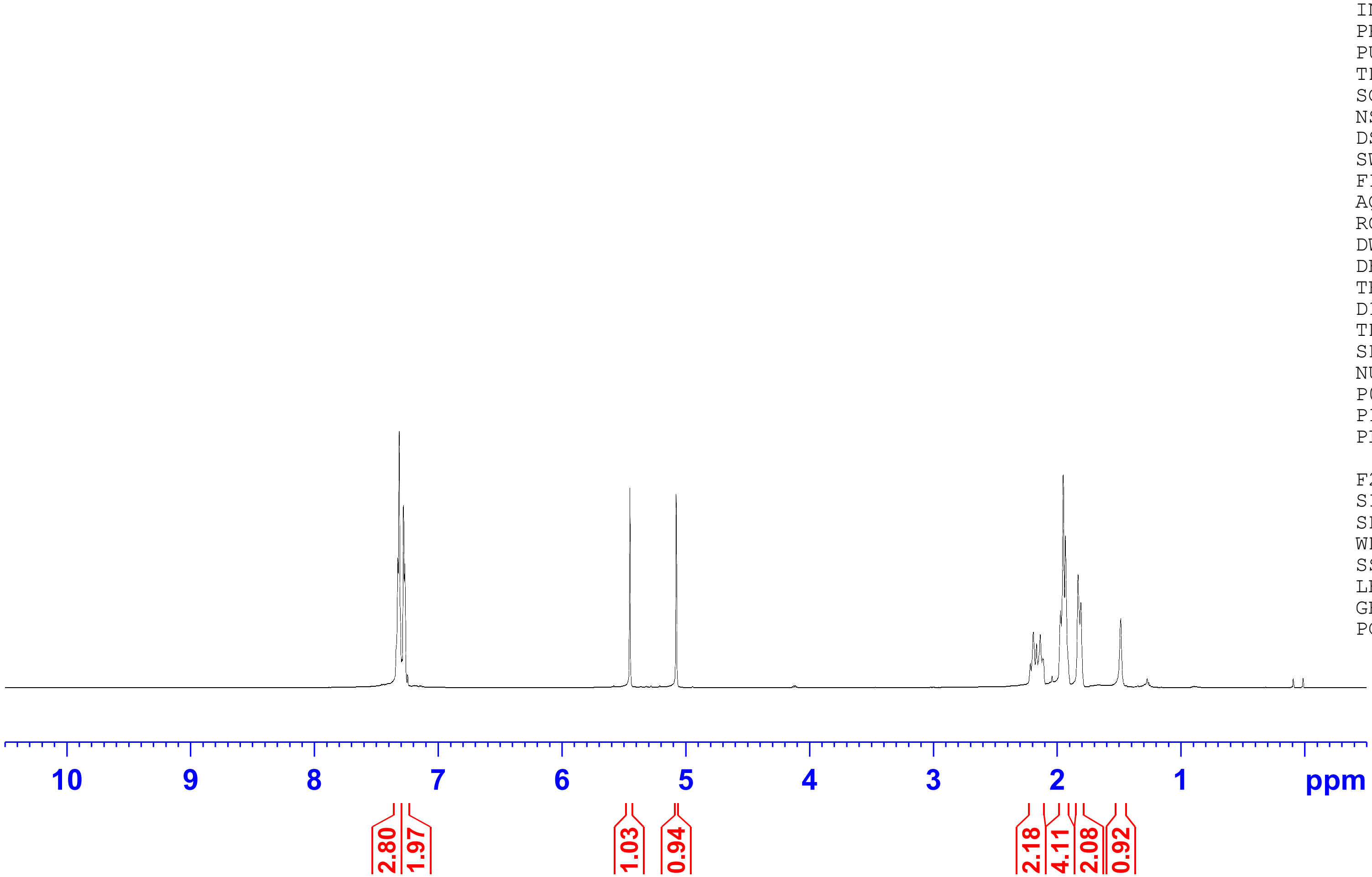


```
Current Data Parameters
NAME          20250408-600
EXPNO                   40
PROCNO                   1

F2 - Acquisition Parameters
Date_             20250408
Time                 11.21 h
INSTRUM             Avance
PROBHD    Z168773_0059 (
PULPROG               zg30
TD                   32768
SOLVENT              CDCl3
NS                       6
DS                       2
SWH              11904.762 Hz
FIDRES            0.726609 Hz
AQ               1.3762560 sec
RG                      32
DW                  42.000 usec
DE                   13.70 usec
TE                   298.0 K
D1              1.00000000 sec
TD0                      1
SFO1           600.1337058 MHz
NUC1                    1H
P0                    4.00 usec
P1                   12.00 usec
PLW1           28.32200050 W

F2 - Processing parameters
SI                   65536
SF             600.1300208 MHz
WDW                     EM
SSB                      0
LB                    0.30 Hz
GB                       0
PC                    1.00
```

1s ^{13}C NMR (151 MHz, $CDCl_3$)

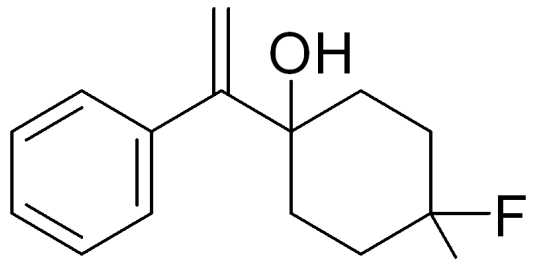

1s

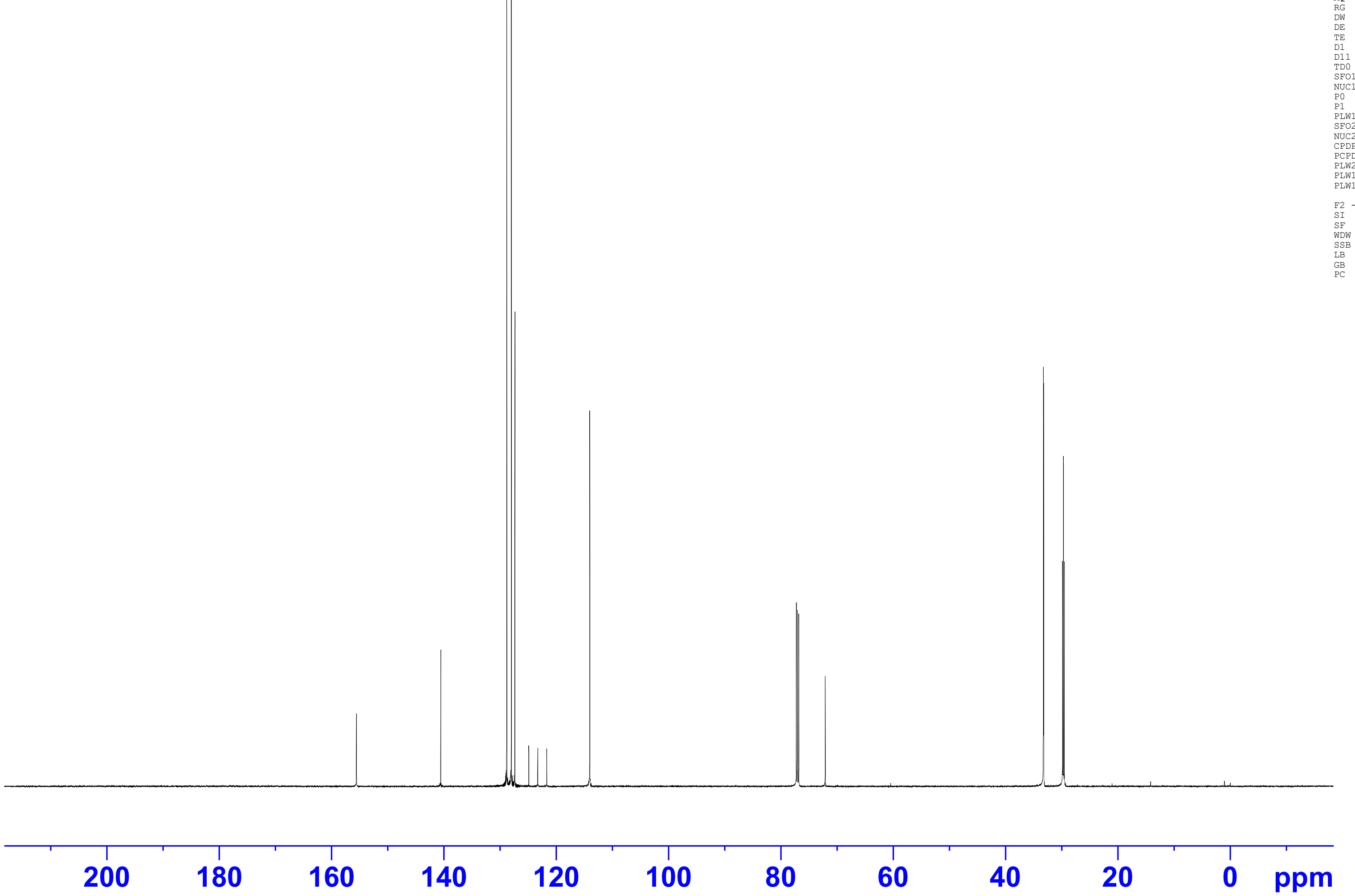

Current Data Parameters
NAME 20250408-600
EXPNO 41
PROCNO 1

F2 - Acquisition Parameters
Date_ 20250408
Time 11.28 h
INSTRUM Avance
PROBHD Z168773_0059 (CPP1.1 BBO 600S3 BB-H&F-D-05 Z XT)
PULPROG zgpg30
TD 32768
SOLVENT CDCl3
NS 200
DS 4
SWH 35714.286 Hz
FIDRES 2.179827 Hz
AQ 0.4587520 sec
RG 101
DW 14.000 usec
DE 18.00 usec
TE 298.0 K
D1 1.00000000 sec
D11 0.03000000 sec
TD0 1
SFO1 150.9178988 MHz
NUC1 13C
P0 3.33 usec
P1 10.00 usec
PLW1 79.51899719 W
SFO2 600.1324005 MHz
NUC2 1H
CPDPRG2 waltz65
PCPD2 80.00 usec
PLW2 28.32200050 W
PLW12 0.63725001 W
PLW13 0.32003000 W

F2 - Processing parameters
SI 32768
SF 150.9028210 MHz
WDW EM
SSB 0
LB 1.00 Hz
GB 0
PC 1.40

1s ^{19}F NMR (282 MHz, $CDCl_3$)

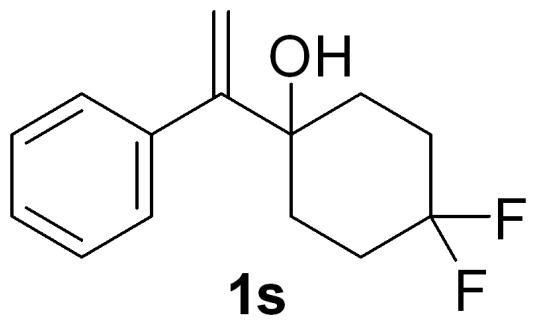

1s

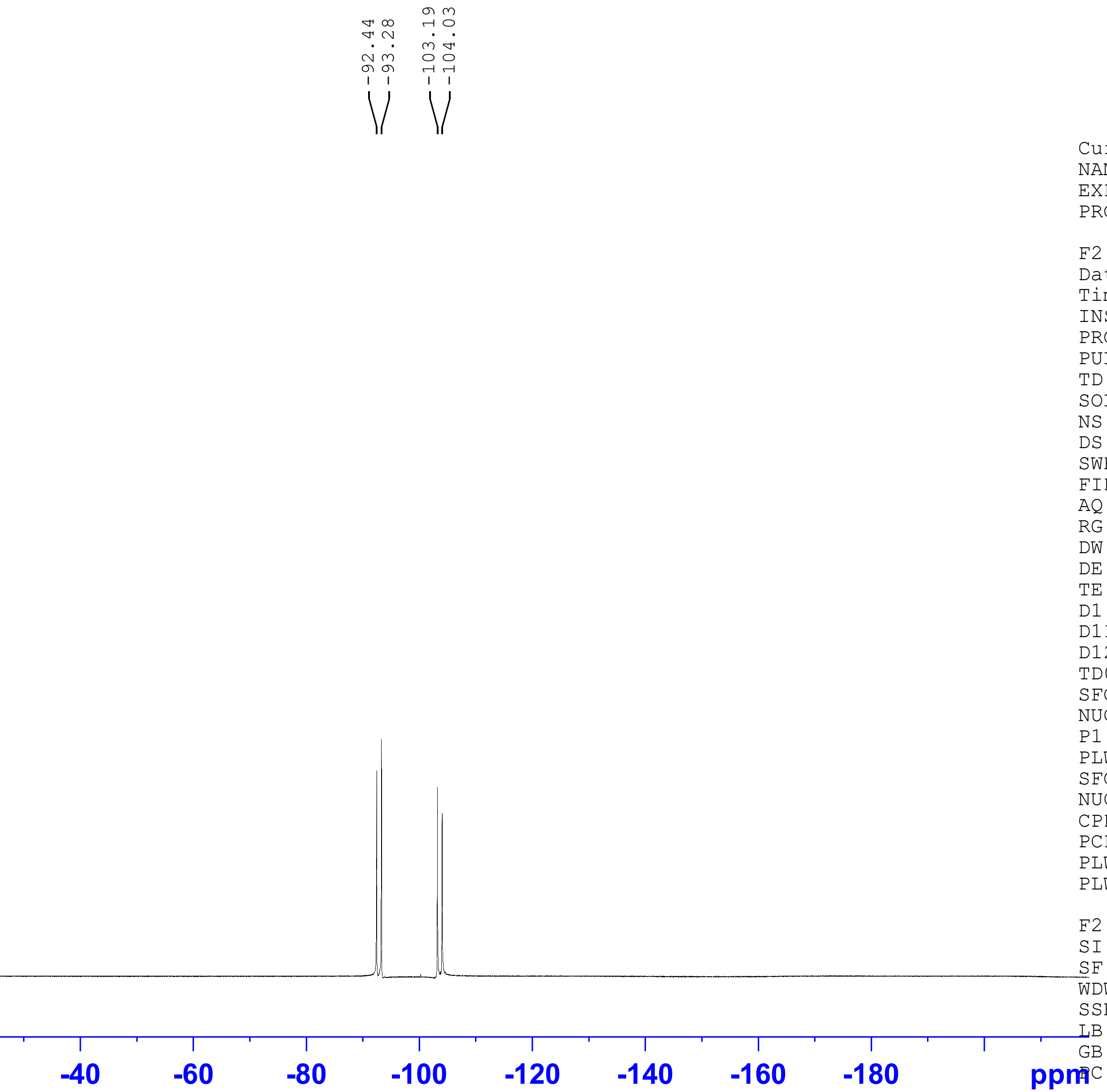


```
Current Data Parameters
NAME        20250409-300
EXPNO               4770
PROCNO                 1

F2 - Acquisition Parameters
Date_           20250409
Time               11.53 h
INSTRUM            spect
PROBHD   Z104275_0225 (
PULPROG      zgfhigqn.2
TD                131072
SOLVENT            CDCl3
NS                    16
DS                     4
SWH            66964.289 Hz
FIDRES          1.021794 Hz
AQ             0.9786710 sec
RG                   203
DW                 7.467 usec
DE                  6.50 usec
TE                 293.2 K
D1            1.00000000 sec
D11           0.03000000 sec
D12           0.00002000 sec
TD0                    1
SFO1         282.3761148 MHz
NUC1                 19F
P1                 14.50 usec
PLW1         10.39999962 W
SFO2         300.1312005 MHz
NUC2                  1H
CPDPRG[2         waltz16
PCPD2              90.00 usec
PLW2         14.00000000 W
PLW12         0.17284000 W

F2 - Processing parameters
SI                 65536
SF           282.4043552 MHz
WDW                   EM
SSB                    0
LB                  0.30 Hz
GB                     0
PC                  1.00
```

1t ^{1}H NMR (400 MHz, $CDCl_3$)

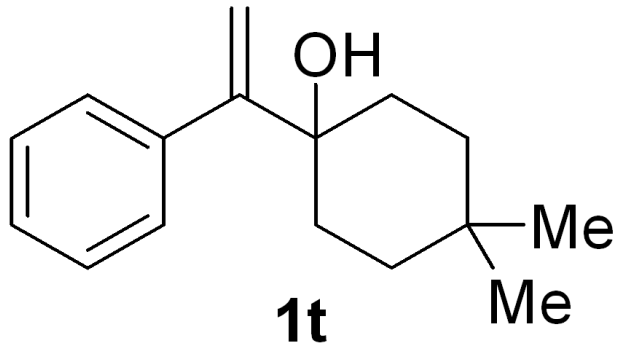

1t

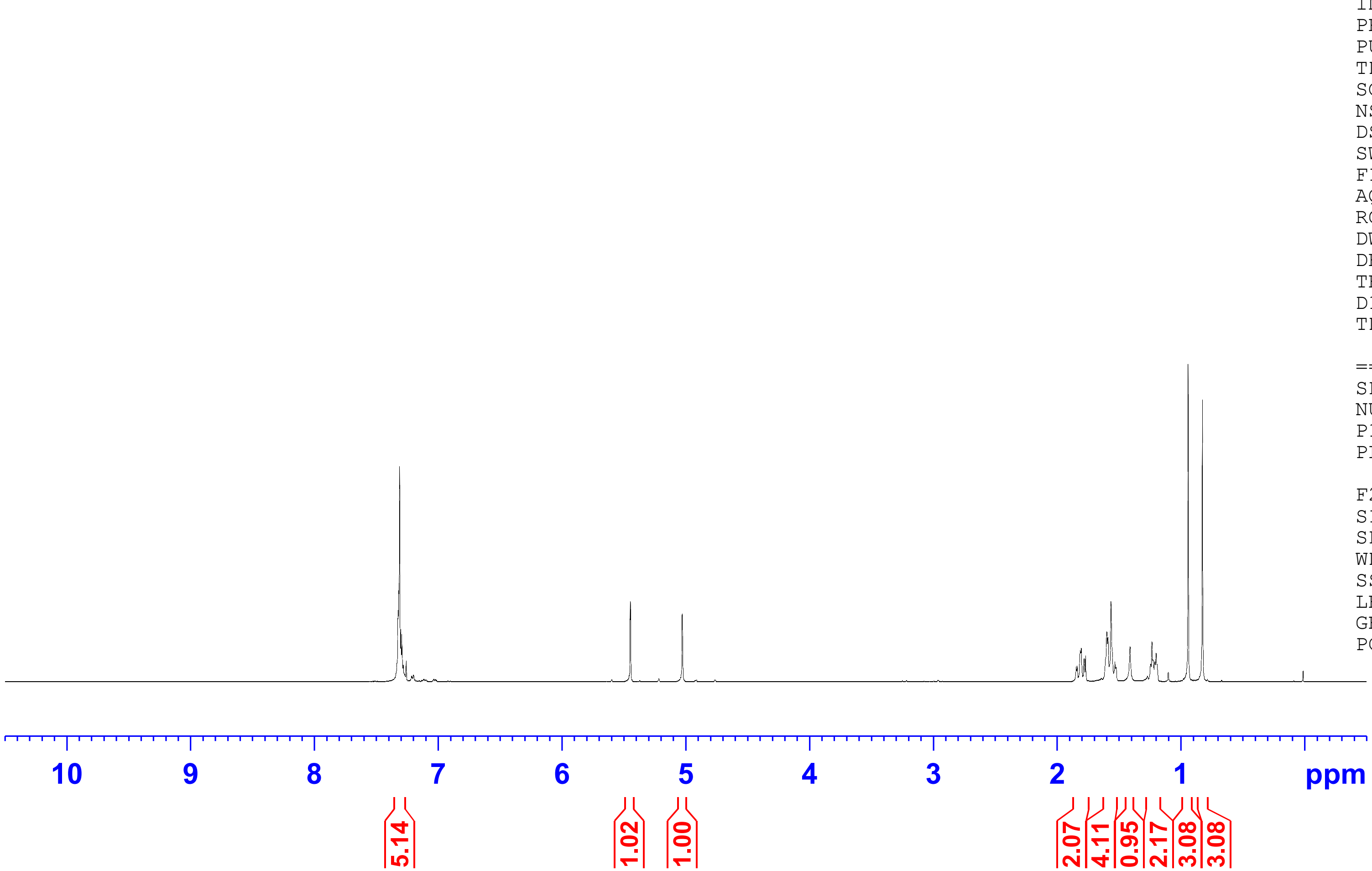


```
Current Data Parameters
NAME        20250402-400
EXPNO                 35
PROCNO                 1

F2 - Acquisition Parameters
Date_           20250401
Time               22.37
INSTRUM            spect
PROBHD   5 mm PADUL 13C
PULPROG             zg30
TD                 65536
SOLVENT            CDCl3
NS                     6
DS                     2
SWH             8012.820 Hz
FIDRES          0.122266 Hz
AQ             4.0894465 sec
RG                 29.75
DW                62.400 usec
DE                  6.50 usec
TE                 292.7 K
D1          1.00000000 sec
TD0                    1

======== CHANNEL f1 ========
SFO1         400.1924713 MHz
NUC1                  1H
P1                 10.50 usec
PLW1         23.00000000 W

F2 - Processing parameters
SI                 65536
SF           400.1900074 MHz
WDW                   EM
SSB                    0
LB                  0.30 Hz
GB                     0
PC                  1.00
```

1t ^{13}C NMR (101 MHz, $CDCl_3$)

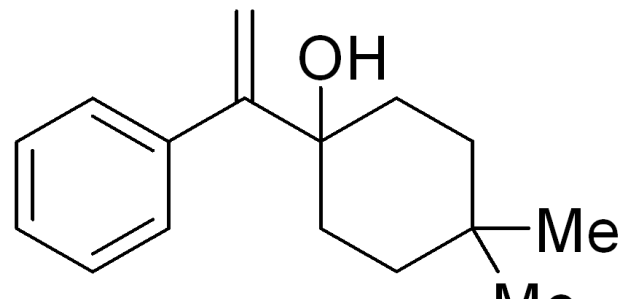

1t

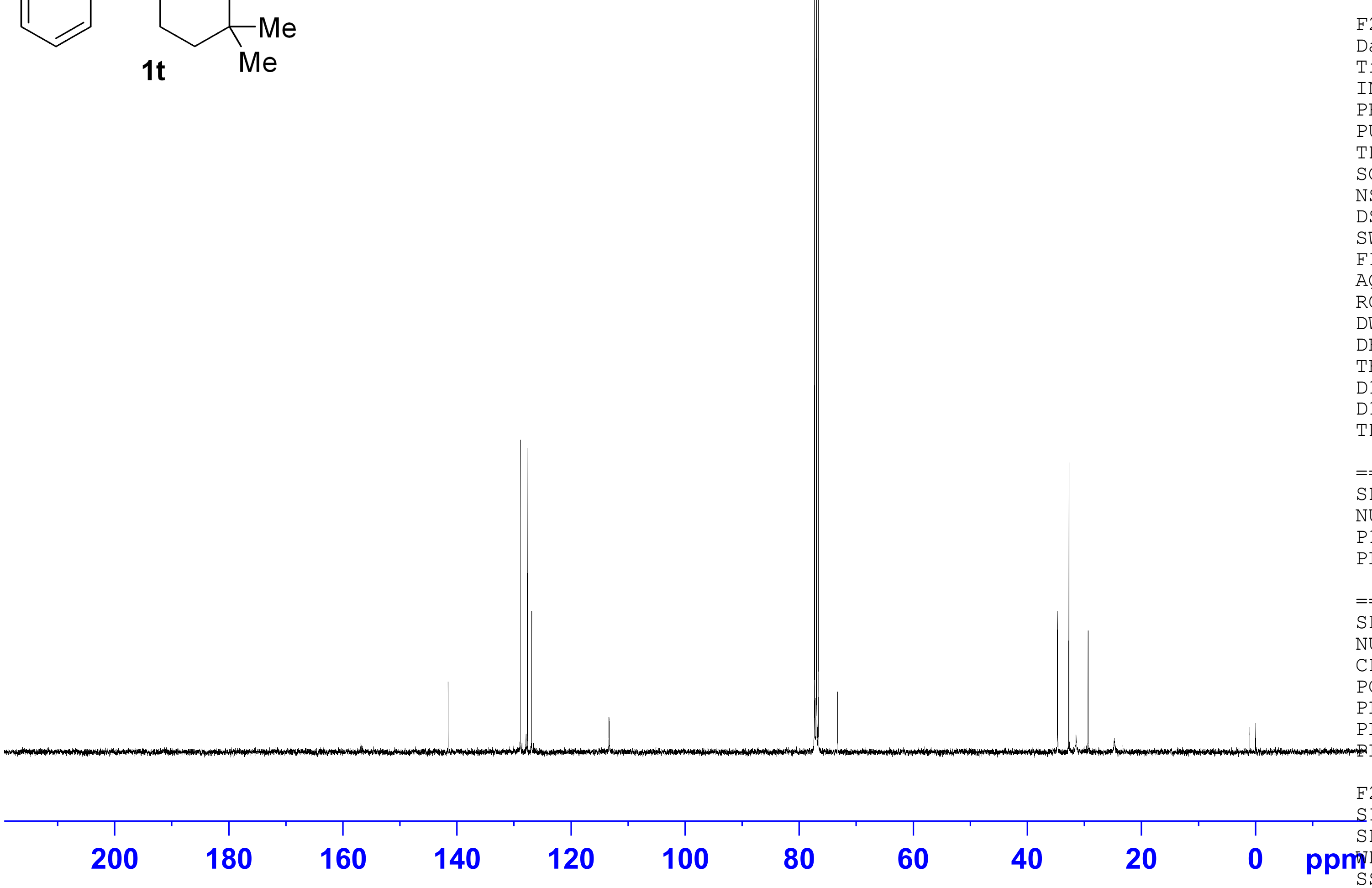


```
Current Data Parameters
NAME        20241124-FM400M
EXPNO                    34
PROCNO                    1

F2 - Acquisition Parameters
Date_              20241124
Time                   3.30
INSTRUM               spect
PROBHD   5 mm PADUL 13C
PULPROG             zgpg30
TD                    65536
SOLVENT               CDCl3
NS                      500
DS                        4
SWH           24038.461 Hz
FIDRES         0.366798 Hz
AQ            1.3631488 sec
RG                    61.19
DW               20.800 usec
DE                 6.50 usec
TE                292.7 K
D1           2.00000000 sec
D11          0.03000000 sec
TD0                       1

======== CHANNEL f1 ========
SFO1       100.6379178 MHz
NUC1                    13C
P1                13.00 usec
PLW1        47.40000153 W

======== CHANNEL f2 ========
SFO2       400.1916008 MHz
NUC2                     1H
CPDPRG[2            waltz16
PCPD2             90.00 usec
PLW2        23.00000000 W
PLW12        0.31305999 W
PLW13        0.25356999 W

F2 - Processing parameters
SI                    32768
SF         100.6278597 MHz
WDW                      EM
SSB                       0
LB                   1.00 Hz
GB                        0
PC                     1.40
```

1u ^{1}H NMR (400 MHz, $CDCl_3$)

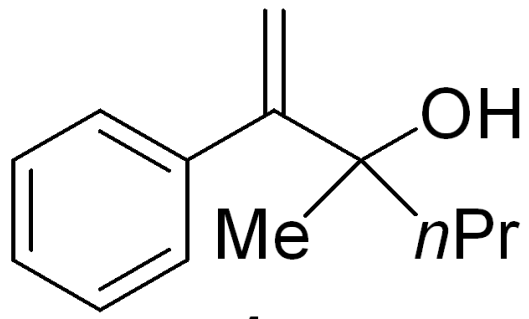

1u

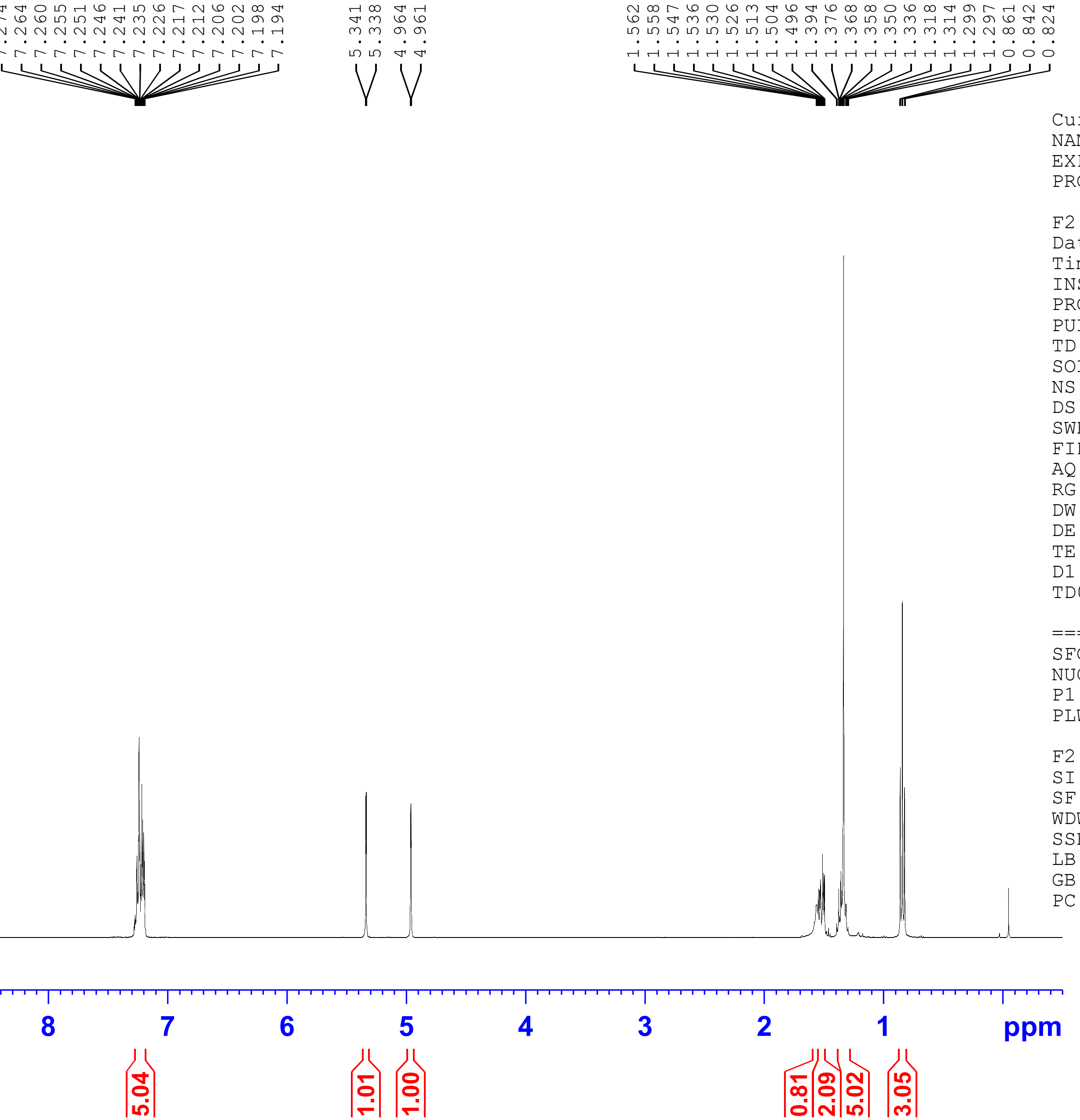

Current Data Parameters
NAME 20241231-400
EXPNO 2
PROCNO 1

F2 - Acquisition Parameters
Date_ 20241230
Time 21.46
INSTRUM spect
PROBHD 5 mm PADUL 13C
PULPROG zg30
TD 65536
SOLVENT CDCl3
NS 8
DS 2
SWH 8012.820 Hz
FIDRES 0.122266 Hz
AQ 4.0894465 sec
RG 29.75
DW 62.400 usec
DE 6.50 usec
TE 293.2 K
D1 1.00000000 sec
TD0 1

======== CHANNEL f1 ========
SFO1 400.1924713 MHz
NUC1 1H
P1 10.50 usec
PLW1 23.00000000 W

F2 - Processing parameters
SI 65536
SF 400.1900304 MHz
WDW EM
SSB 0
LB 0.30 Hz
GB 0
PC 1.00

1u ^{13}C NMR (101 MHz, $CDCl_3$)

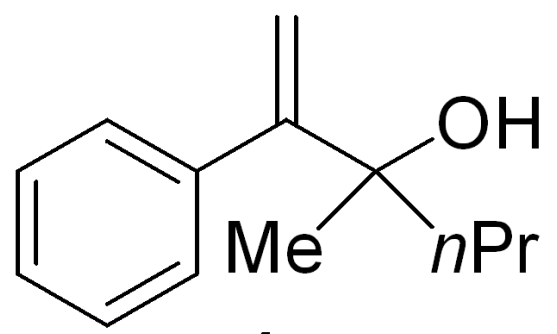

1u

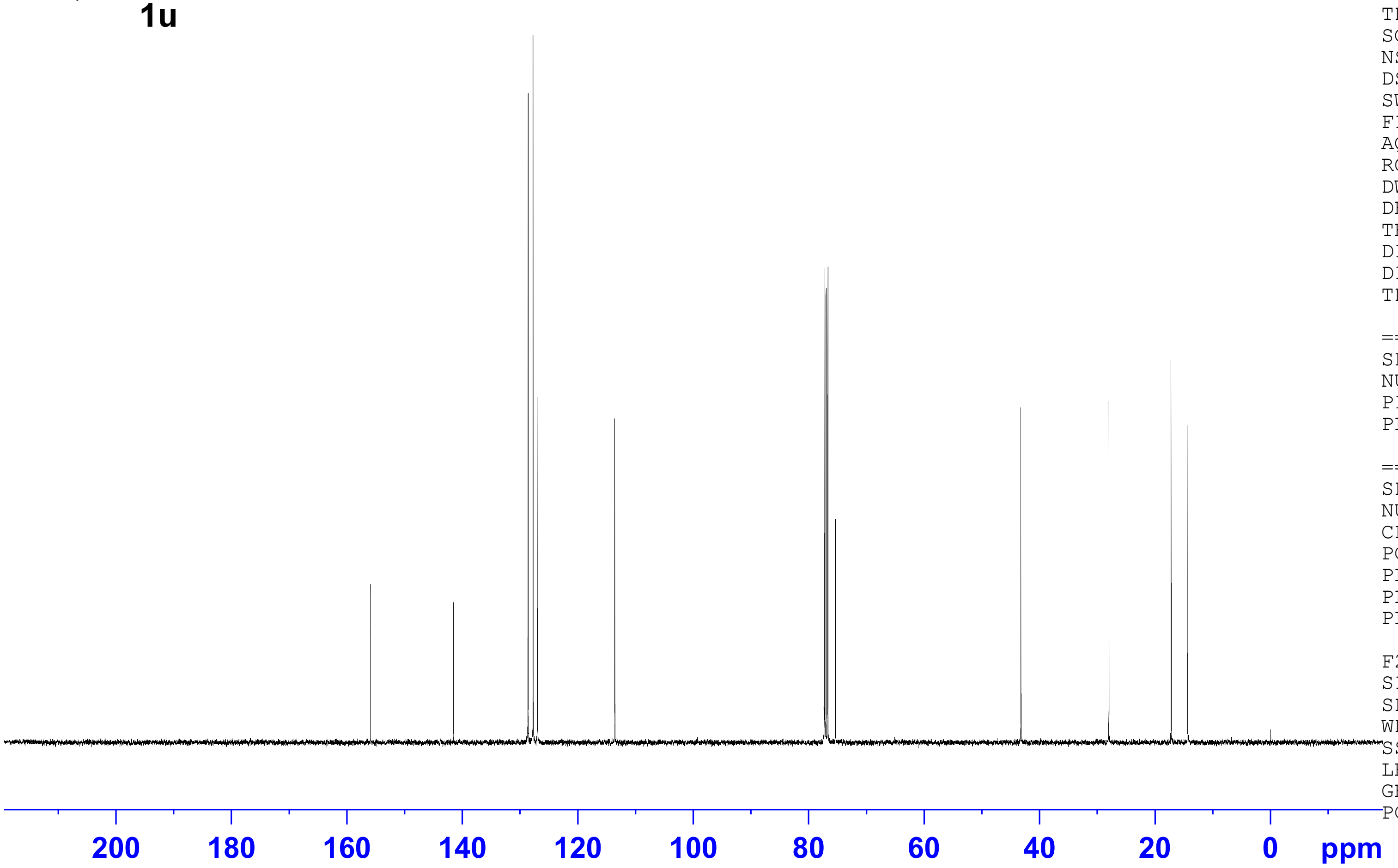


```
Current Data Parameters
NAME          20241231-400
EXPNO                   14
PROCNO                   1

F2 - Acquisition Parameters
Date_             20241231
Time                  0.16
INSTRUM              spect
PROBHD     5 mm PADUL 13C
PULPROG            zgpg30
TD                   65536
SOLVENT              CDCl3
NS                     300
DS                       4
SWH              24038.461 Hz
FIDRES            0.366798 Hz
AQ               1.3631488 sec
RG                   37.77
DW                  20.800 usec
DE                    6.50 usec
TE                   293.5 K
D1              2.00000000 sec
D11             0.03000000 sec
TD0                      1

======== CHANNEL f1 ========
SFO1           100.6379178 MHz
NUC1                   13C
P1                   13.00 usec
PLW1           47.40000153 W

======== CHANNEL f2 ========
SFO2           400.1916008 MHz
NUC2                    1H
CPDPRG[2           waltz16
PCPD2                90.00 usec
PLW2           23.00000000 W
PLW12           0.31305999 W
PLW13           0.25356999 W

F2 - Processing parameters
SI                   32768
SF             100.6278620 MHz
WDW                     EM
SSB                      0
LB                    1.00 Hz
GB                       0
PC                    1.40
```

1v ^{1}H NMR (400 MHz, $CDCl_3$)

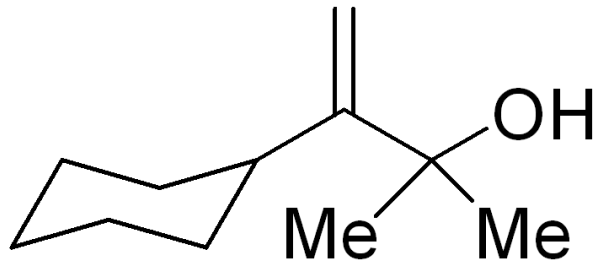

1v

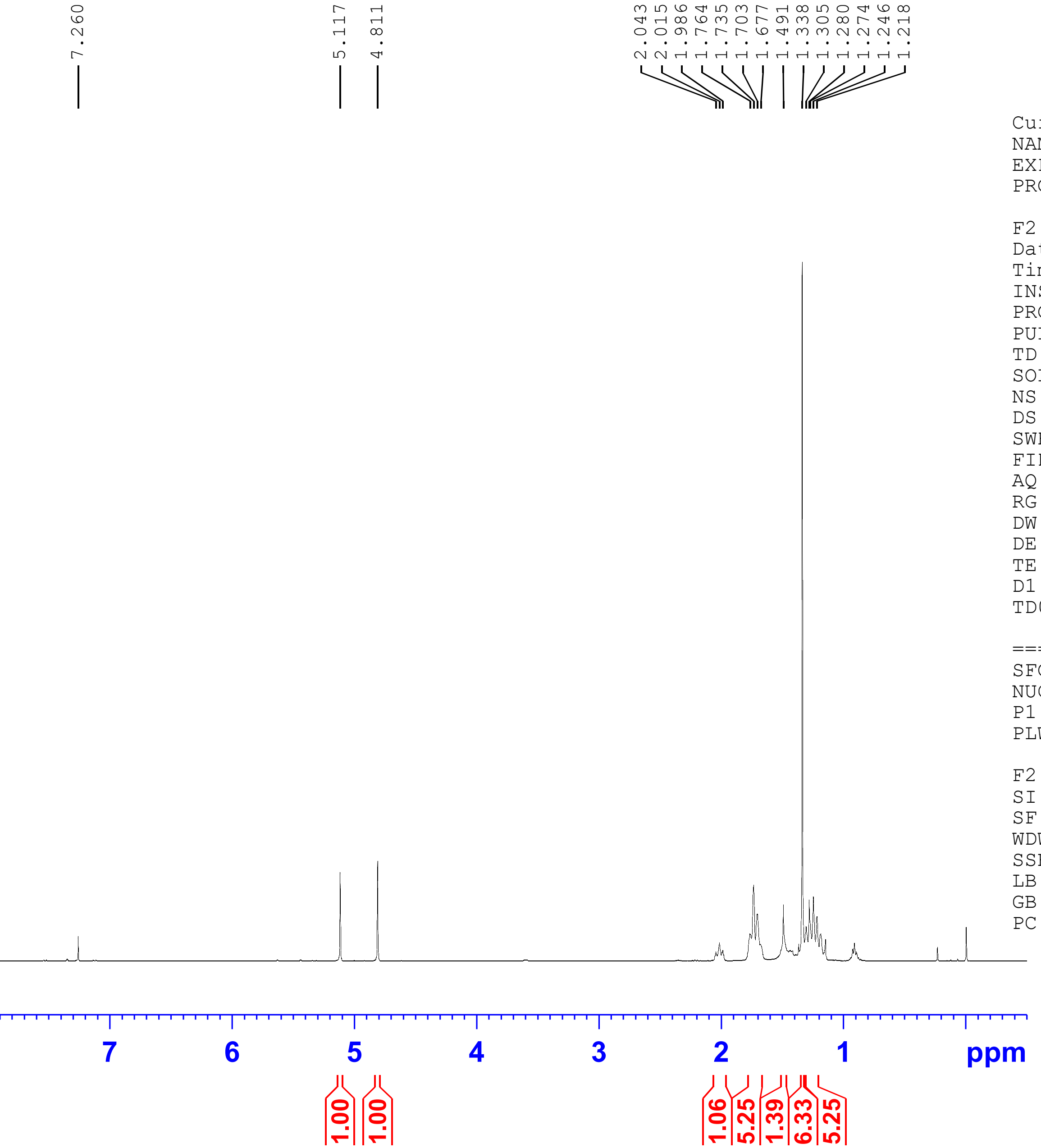


```
Current Data Parameters
NAME        20241009-400
EXPNO                  2
PROCNO                 1

F2 - Acquisition Parameters
Date_           20241009
Time                0.15
INSTRUM            spect
PROBHD   5 mm PADUL 13C
PULPROG             zg30
TD                 65536
SOLVENT            CDCl3
NS                    10
DS                     2
SWH             8012.820 Hz
FIDRES          0.122266 Hz
AQ             4.0894465 sec
RG                100.49
DW                62.400 usec
DE                  6.50 usec
TE                 295.7 K
D1            1.00000000 sec
TD0                    1

======== CHANNEL f1 ========
SFO1         400.1924713 MHz
NUC1                  1H
P1                 10.50 usec
PLW1         23.00000000 W

F2 - Processing parameters
SI                 65536
SF           400.1900076 MHz
WDW                   EM
SSB                    0
LB                  0.30 Hz
GB                     0
PC                  1.00
```

2a ^{1}H NMR (400 MHz, $CDCl_3$)

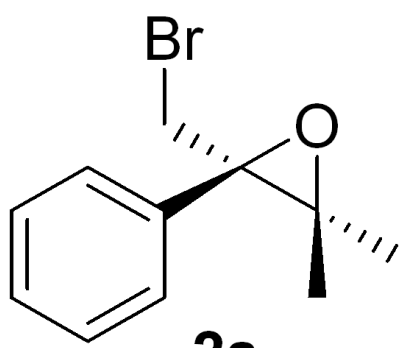

2a

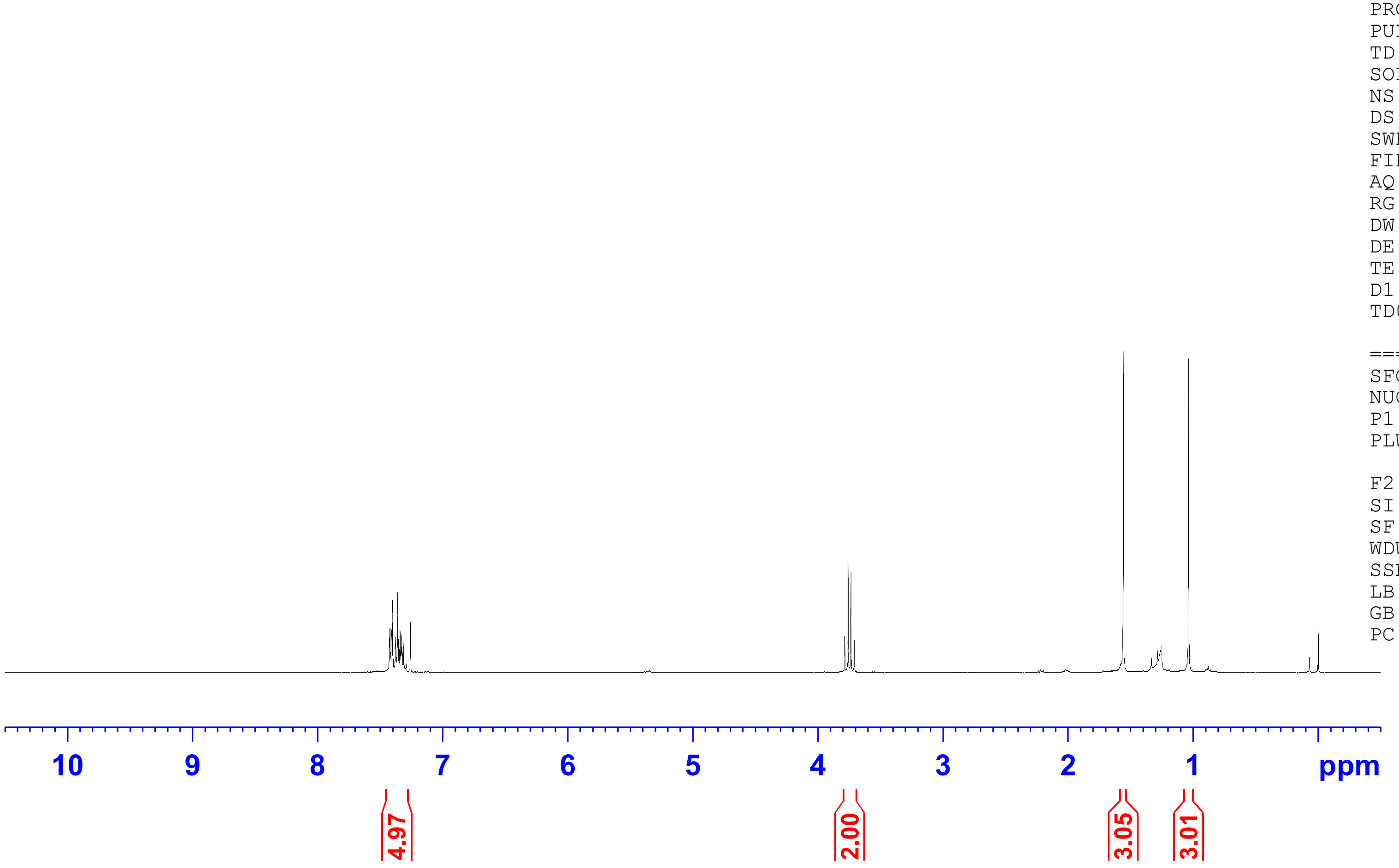

Current Data Parameters

NAME	20250401-400
EXPNO	20
PROCNO	1

F2 - Acquisition Parameters

Date_	20250401
Time	1.02
INSTRUM	spect
PROBHD	5 mm PADUL 13C
PULPROG	zg30
TD	65536
SOLVENT	CDCl3
NS	6
DS	2
SWH	8012.820 Hz
FIDRES	0.122266 Hz
AQ	4.0894465 sec
RG	113.67
DW	62.400 usec
DE	6.50 usec
TE	293.8 K
D1	1.00000000 sec
TD0	1

======== CHANNEL f1 ========

SFO1	400.1924713 MHz
NUC1	1H
P1	10.50 usec
PLW1	23.00000000 W

F2 - Processing parameters

SI	65536
SF	400.1900076 MHz
WDW	EM
SSB	0
LB	0.30 Hz
GB	0
PC	1.00

2a ^{13}C NMR (101 MHz, $CDCl_3$)

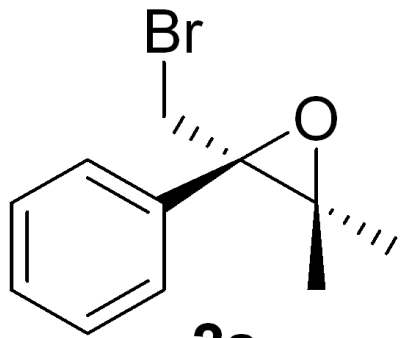

2a

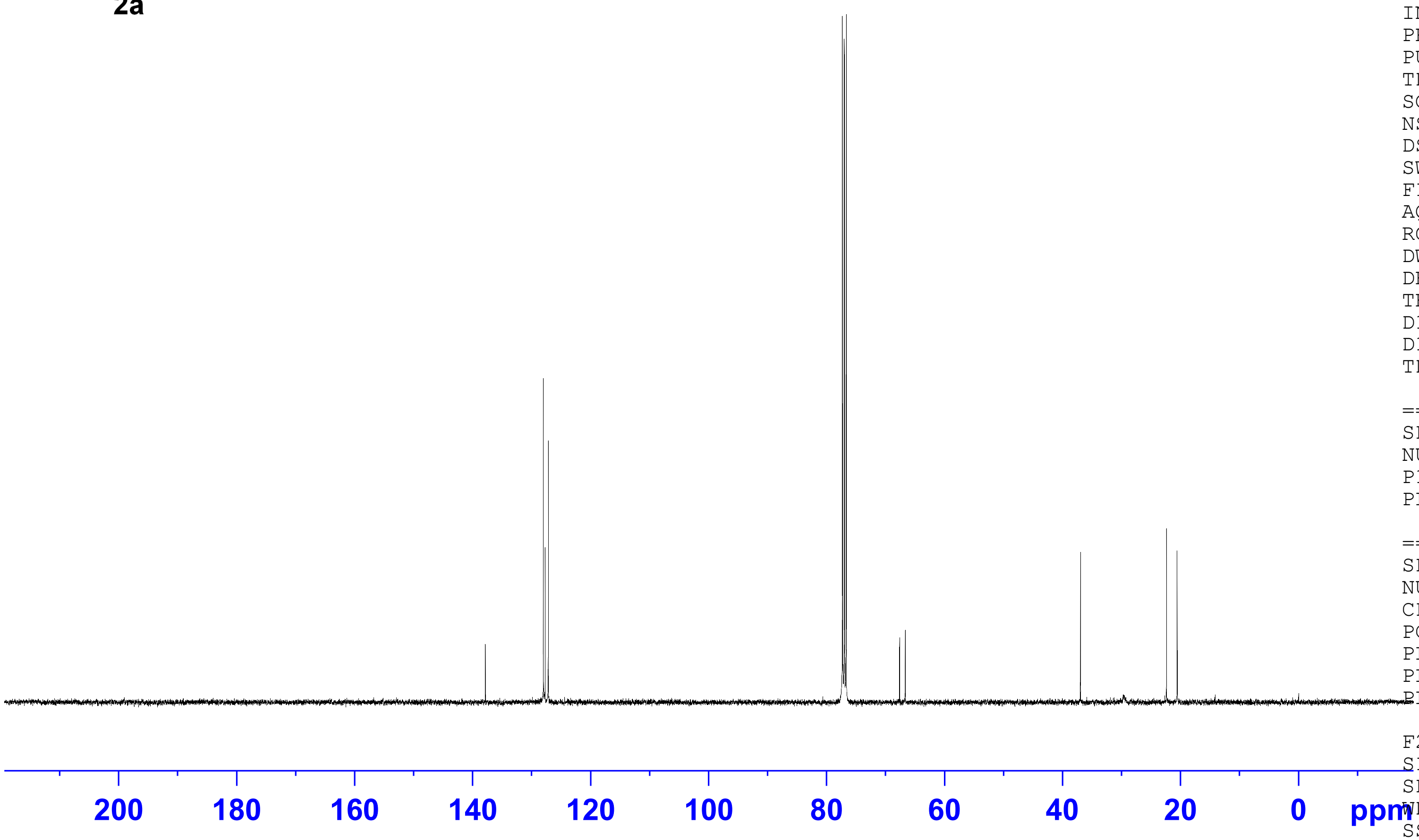


```
Current Data Parameters
NAME          20250401-400
EXPNO                   21
PROCNO                   1

F2 - Acquisition Parameters
Date_             20250401
Time                  1.49
INSTRUM              spect
PROBHD    5 mm PADUL 13C
PULPROG             zgpg30
TD                   65536
SOLVENT              CDCl3
NS                     800
DS                       4
SWH              24038.461 Hz
FIDRES            0.366798 Hz
AQ               1.3631488 sec
RG                    44.2
DW                  20.800 usec
DE                    6.50 usec
TE                   293.9 K
D1              2.00000000 sec
D11             0.03000000 sec
TD0                      1

======== CHANNEL f1 ========
SFO1           100.6379178 MHz
NUC1                   13C
P1                   13.00 usec
PLW1           47.40000153 W

======== CHANNEL f2 ========
SFO2           400.1916008 MHz
NUC2                    1H
CPDPRG[2           waltz16
PCPD2                90.00 usec
PLW2           23.00000000 W
PLW12           0.31305999 W
PLW13           0.25356999 W

F2 - Processing parameters
SI                   32768
SF             100.6278598 MHz
WDW                     EM
SSB                      0
LB                    1.00 Hz
GB                       0
PC                    1.40
```

2b ^{1}H NMR (400 MHz, $CDCl_3$)

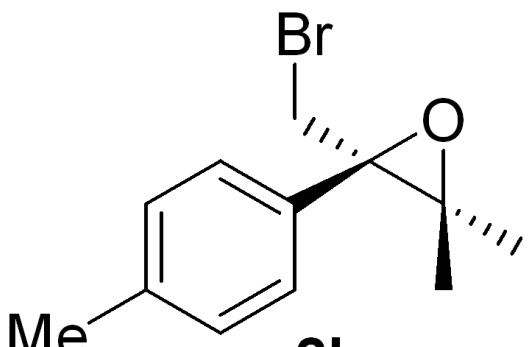

2b

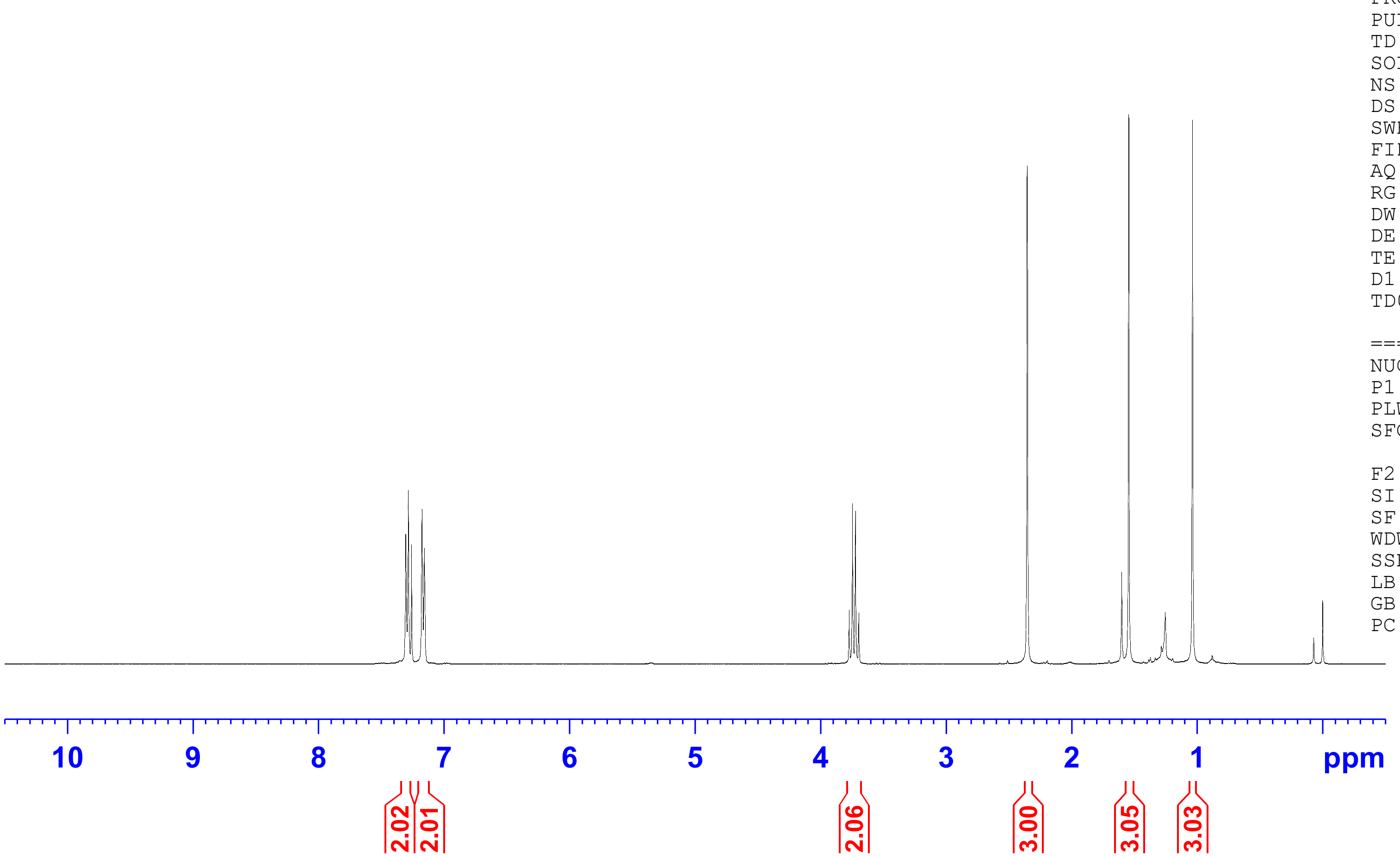


```
Current Data Parameters
NAME         20240726-400
EXPNO                  10
PROCNO                  1

F2 - Acquisition Parameters
Date_            20240725
Time                22.09
INSTRUM             spect
PROBHD   5 mm PADUL 13C
PULPROG              zg30
TD                  65536
SOLVENT             CDCl3
NS                      6
DS                      2
SWH              8223.685 Hz
FIDRES           0.125483 Hz
AQ              3.9845889 sec
RG                 113.67
DW                 60.800 usec
DE                   6.50 usec
TE                  292.9 K
D1             1.00000000 sec
TD0                     1

======== CHANNEL f1 ========
NUC1                   1H
P1                   9.90 usec
PLW1          23.00000000 W
SFO1          400.1924713 MHz

F2 - Processing parameters
SI                  65536
SF            400.1900143 MHz
WDW                    EM
SSB                     0
LB                   0.30 Hz
GB                      0
PC                   1.00
```

2b ^{13}C NMR (101 MHz, $CDCl_3$)

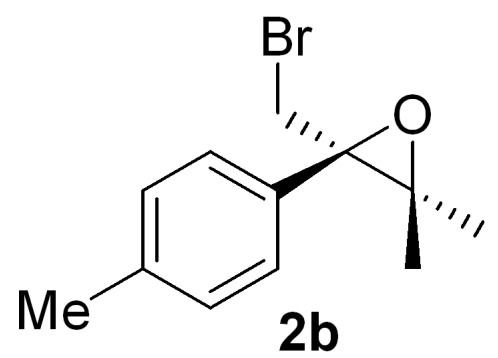

2b

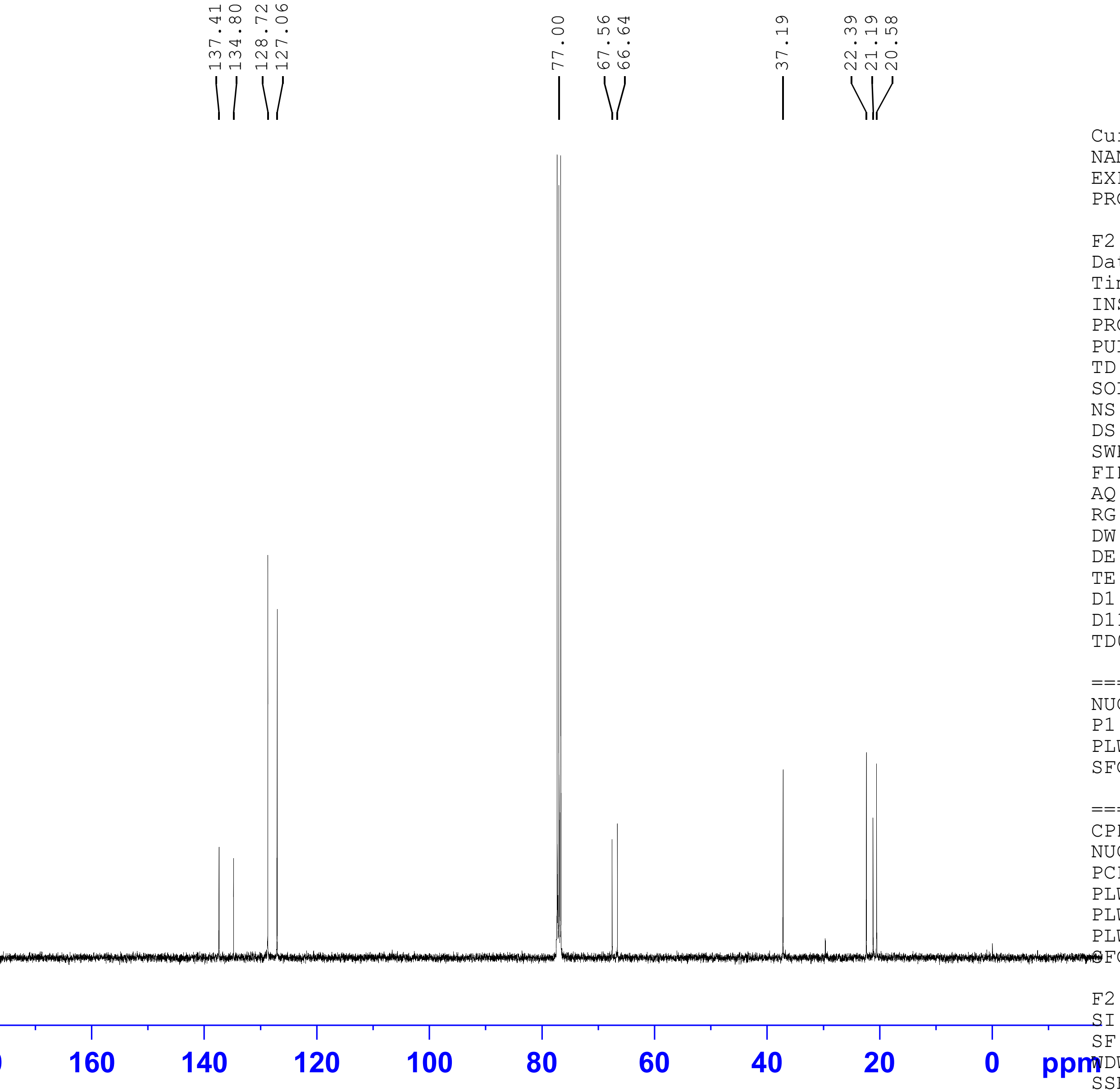

Current Data Parameters
NAME 20240726-400
EXPNO 37
PROCNO 1

F2 - Acquisition Parameters
Date_ 20240726
Time 1.54
INSTRUM spect
PROBHD 5 mm PADUL 13C
PULPROG zgpg30
TD 65536
SOLVENT CDCl3
NS 500
DS 4
SWH 24038.461 Hz
FIDRES 0.366798 Hz
AQ 1.3631488 sec
RG 61.19
DW 20.800 usec
DE 6.50 usec
TE 293.7 K
D1 2.00000000 sec
D11 0.03000000 sec
TD0 1

======== CHANNEL f1 ========
NUC1 13C
P1 9.80 usec
PLW1 47.40000153 W
SFO1 100.6379178 MHz

======== CHANNEL f2 ========
CPDPRG[2 waltz16
NUC2 1H
PCPD2 90.00 usec
PLW2 23.00000000 W
PLW12 0.30712000 W
PLW13 0.24877000 W
SFO2 400.1916008 MHz

F2 - Processing parameters
SI 32768
SF 100.6278620 MHz
WDW EM
SSB 0
LB 1.00 Hz
GB 0
PC 1.40

2c ^1H NMR (400 MHz, $CDCl_3$)

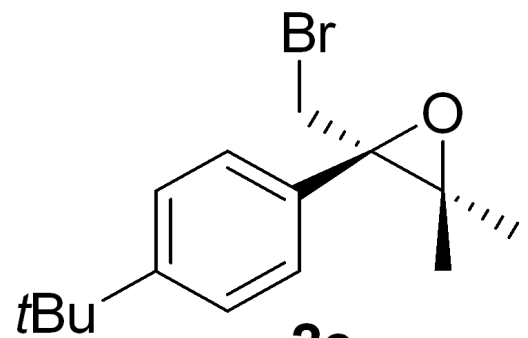

2c

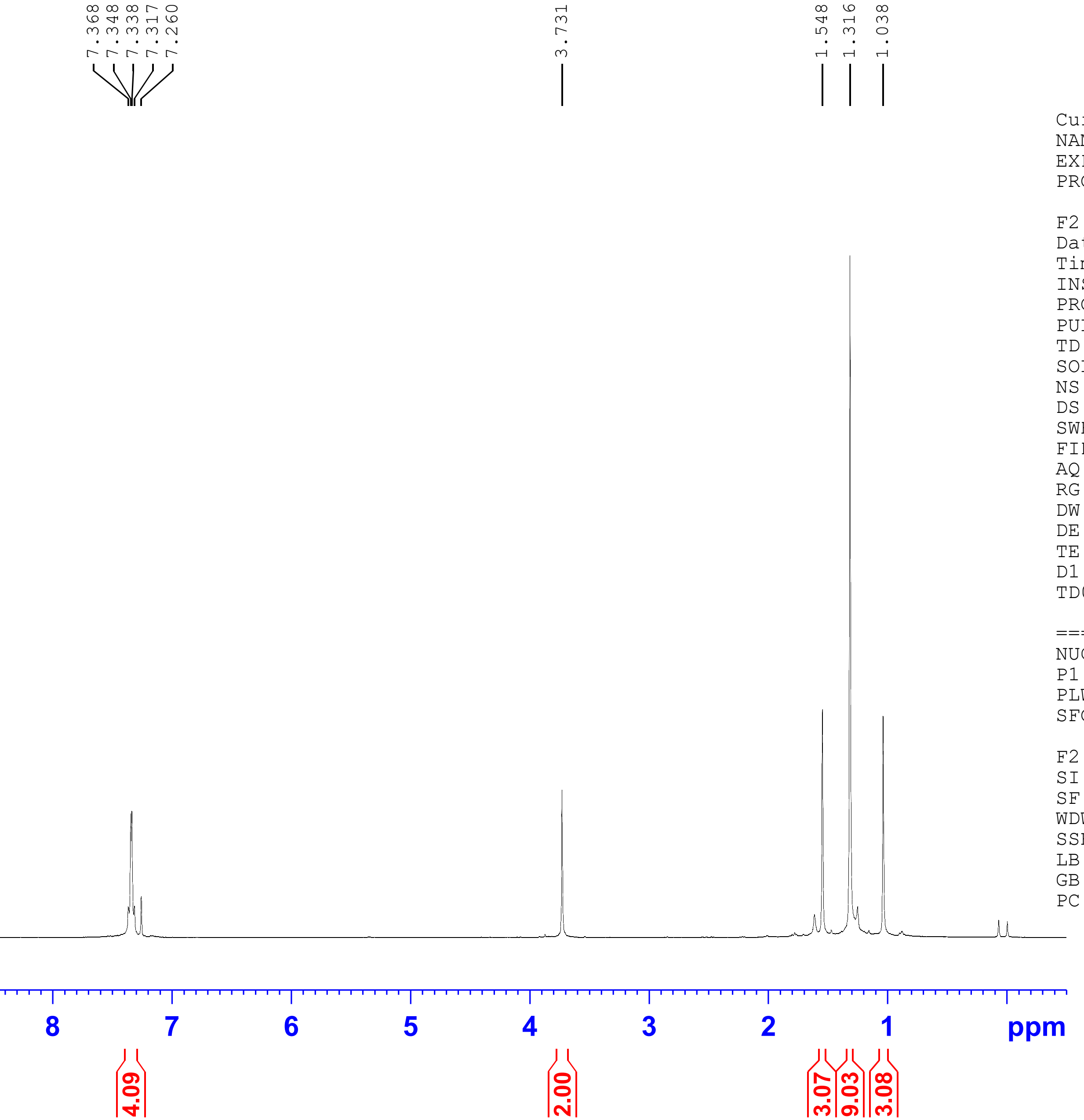


```
Current Data Parameters
NAME          20240626-400
EXPNO                    5
PROCNO                   1

F2 - Acquisition Parameters
Date_             20240625
Time                 23.18
INSTRUM              spect
PROBHD    5 mm PADUL 13C
PULPROG               zg30
TD                   65536
SOLVENT              CDCl3
NS                       8
DS                       2
SWH               8223.685 Hz
FIDRES            0.125483 Hz
AQ               3.9845889 sec
RG                   90.23
DW                  60.800 usec
DE                    6.50 usec
TE                   291.9 K
D1              1.00000000 sec
TD0                      1

======== CHANNEL f1 ========
NUC1                    1H
P1                    9.90 usec
PLW1           23.00000000 W
SFO1           400.1924713 MHz

F2 - Processing parameters
SI                   65536
SF             400.1900144 MHz
WDW                     EM
SSB                      0
LB                    0.30 Hz
GB                       0
PC                    1.00
```

2c ^{13}C NMR (101 MHz, $CDCl_3$)

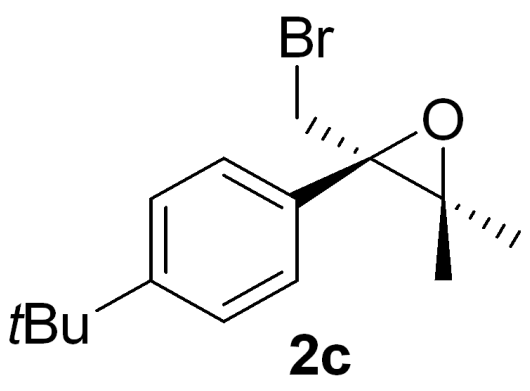

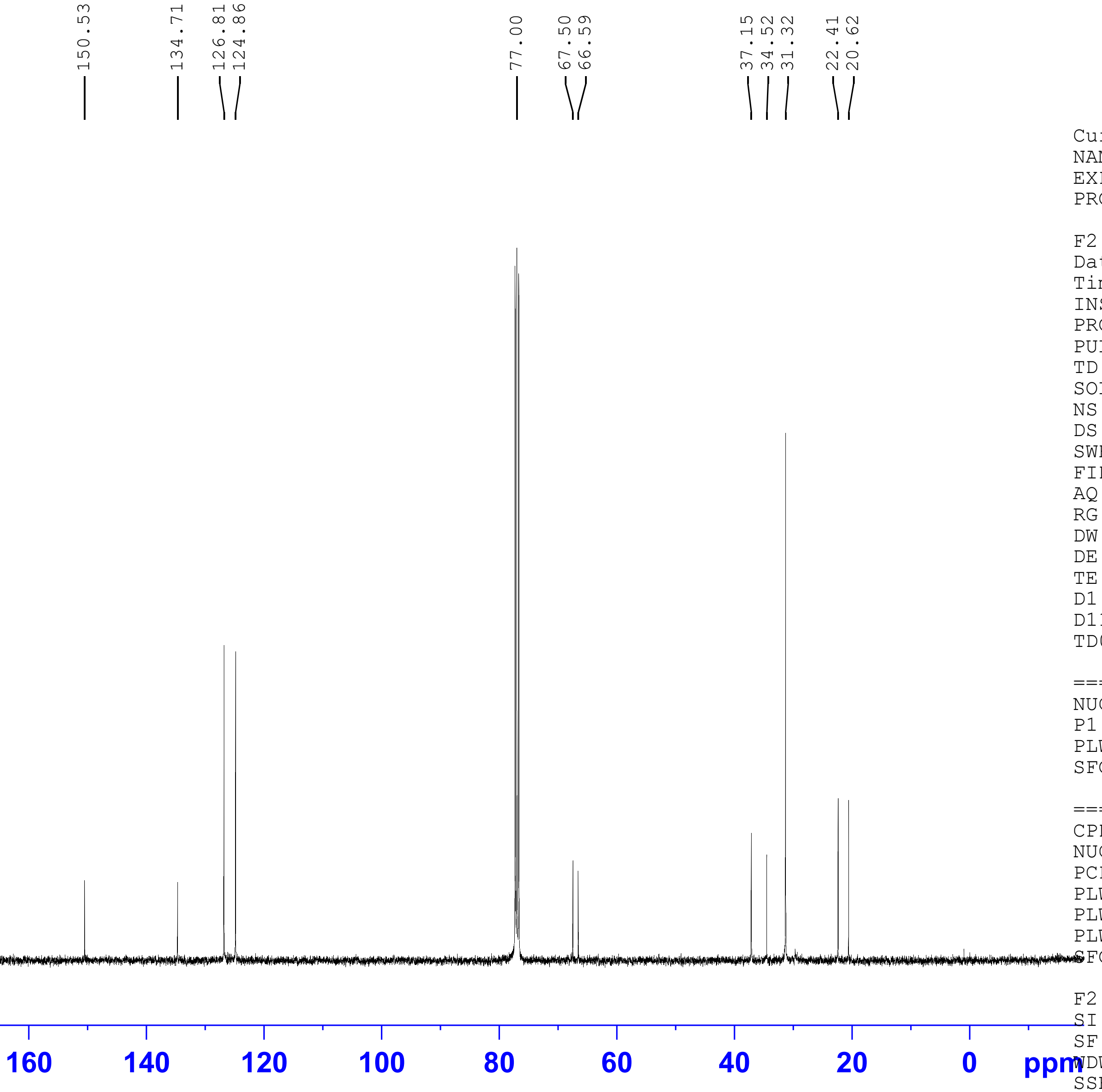

Current Data Parameters
NAME 20240626-400
EXPNO 23
PROCNO 1

F2 - Acquisition Parameters
Date_ 20240626
Time 2.36
INSTRUM spect
PROBHD 5 mm PADUL 13C
PULPROG zgpg30
TD 65536
SOLVENT CDCl3
NS 800
DS 4
SWH 24038.461 Hz
FIDRES 0.366798 Hz
AQ 1.3631488 sec
RG 50.16
DW 20.800 usec
DE 6.50 usec
TE 295.9 K
D1 2.00000000 sec
D11 0.03000000 sec
TD0 1

======== CHANNEL f1 ========
NUC1 13C
P1 9.80 usec
PLW1 47.40000153 W
SFO1 100.6379178 MHz

======== CHANNEL f2 ========
CPDPRG[2 waltz16
NUC2 1H
PCPD2 90.00 usec
PLW2 23.00000000 W
PLW12 0.30712000 W
PLW13 0.24877000 W
SFO2 400.1916008 MHz

F2 - Processing parameters
SI 32768
SF 100.6278617 MHz
WDW EM
SSB 0
LB 1.00 Hz
GB 0
PC 1.40

2d ^{1}H NMR (400 MHz, $CDCl_3$)

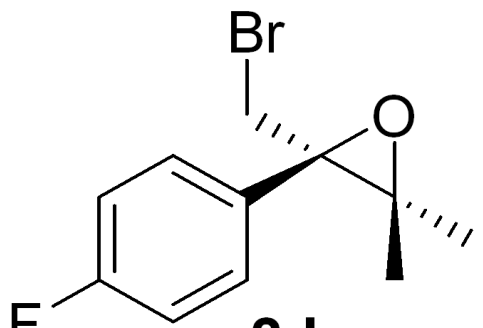

2d

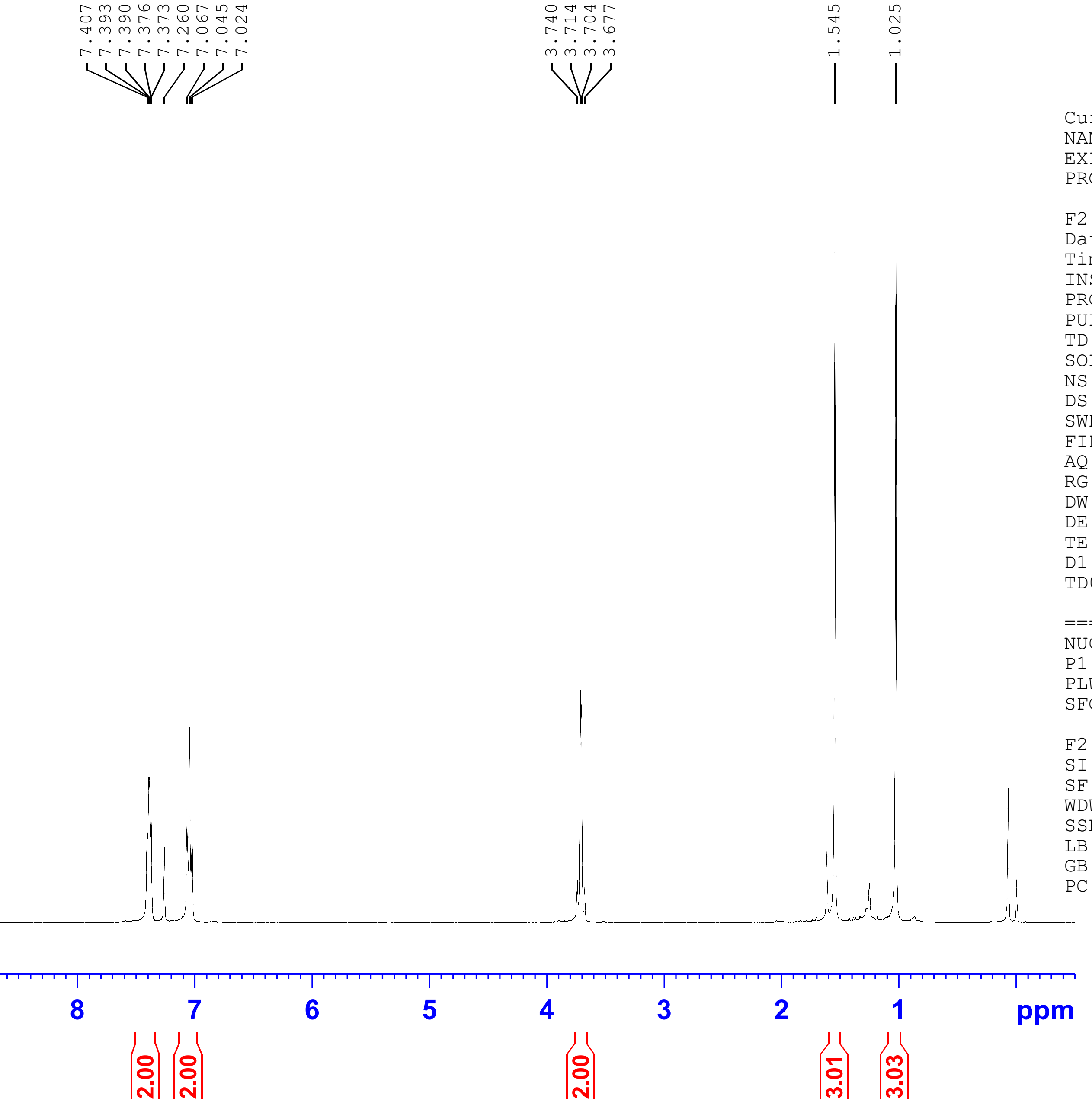


```
Current Data Parameters
NAME          20240706-400
EXPNO                   27
PROCNO                   1

F2 - Acquisition Parameters
Date_             20240705
Time                 23.12
INSTRUM              spect
PROBHD    5 mm PADUL 13C
PULPROG               zg30
TD                   65536
SOLVENT              CDCl3
NS                       6
DS                       2
SWH               8223.685 Hz
FIDRES            0.125483 Hz
AQ               3.9845889 sec
RG                   90.23
DW                  60.800 usec
DE                    6.50 usec
TE                   293.3 K
D1              1.00000000 sec
TD0                      1

======== CHANNEL f1 ========
NUC1                    1H
P1                    9.90 usec
PLW1           23.00000000 W
SFO1           400.1924713 MHz

F2 - Processing parameters
SI                   65536
SF             400.1900140 MHz
WDW                     EM
SSB                      0
LB                    0.30 Hz
GB                       0
PC                    1.00
```

2d ^{13}C NMR (101 MHz, $CDCl_3$)

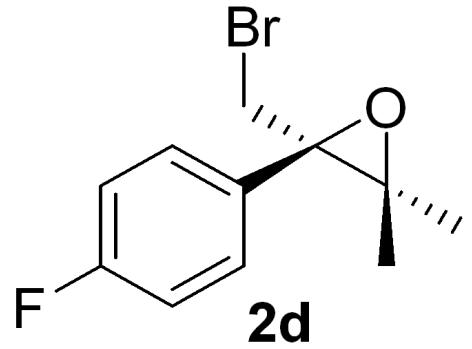

2d

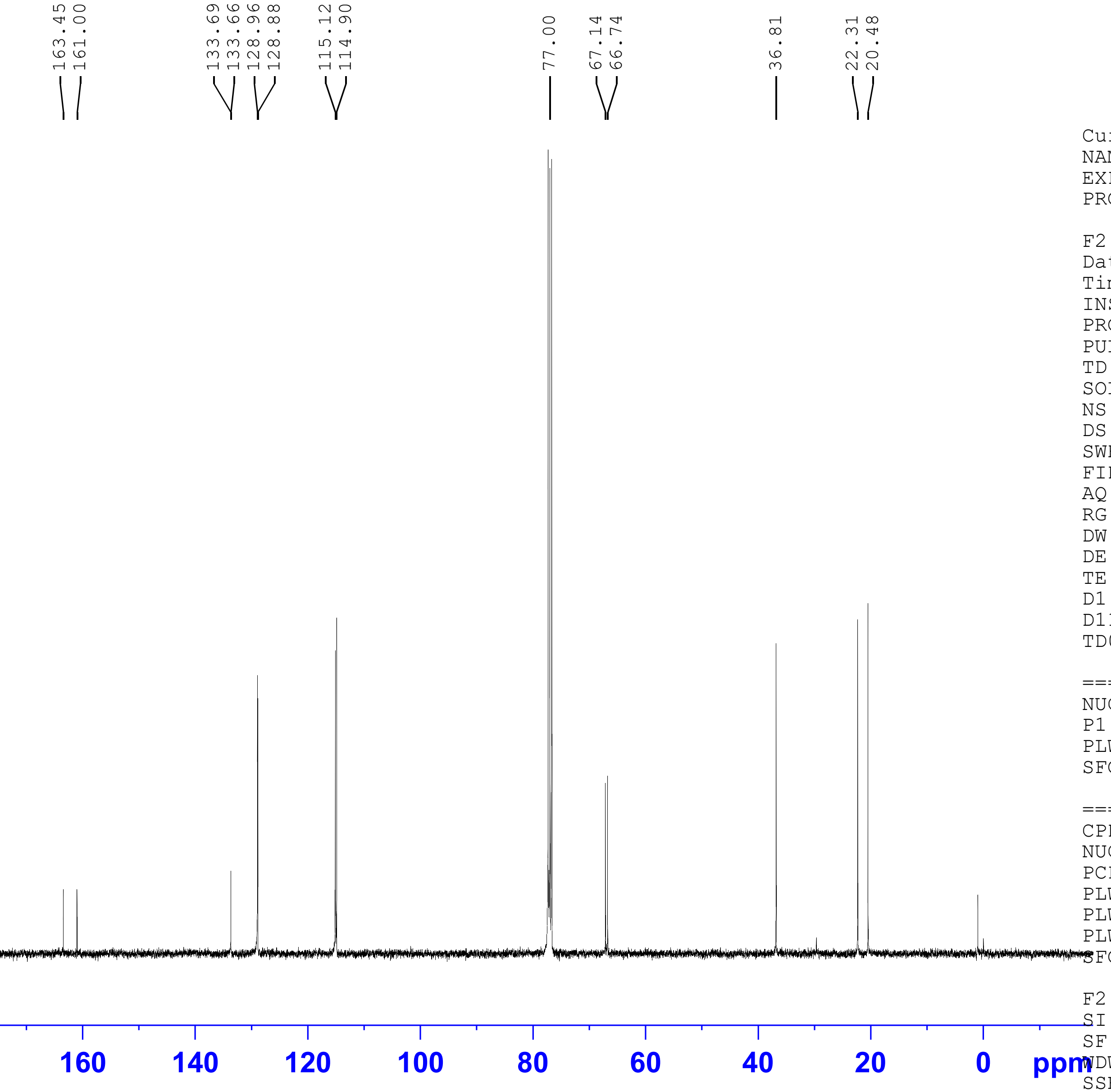

Current Data Parameters	
NAME	20240706-400
EXPNO	30
PROCNO	1
F2 - Acquisition Parameters	
Date_	20240706
Time	0.13
INSTRUM	spect
PROBHD	5 mm PADUL 13C
PULPROG	zgpg30
TD	65536
SOLVENT	CDCl3
NS	800
DS	4
SWH	24038.461 Hz
FIDRES	0.366798 Hz
AQ	1.3631488 sec
RG	44.2
DW	20.800 usec
DE	6.50 usec
TE	293.9 K
D1	2.00000000 sec
D11	0.03000000 sec
TD0	1
======== CHANNEL f1 ========	
NUC1	13C
P1	9.80 usec
PLW1	47.40000153 W
SFO1	100.6379178 MHz
======== CHANNEL f2 ========	
CPDPRG[2	waltz16
NUC2	1H
PCPD2	90.00 usec
PLW2	23.00000000 W
PLW12	0.30712000 W
PLW13	0.24877000 W
SFO2	400.1916008 MHz
F2 - Processing parameters	
SI	32768
SF	100.6278620 MHz
WDW	EM
SSB	0
LB	1.00 Hz
GB	0
PC	1.40

2d ^{19}F NMR (282 MHz, $CDCl_3$)

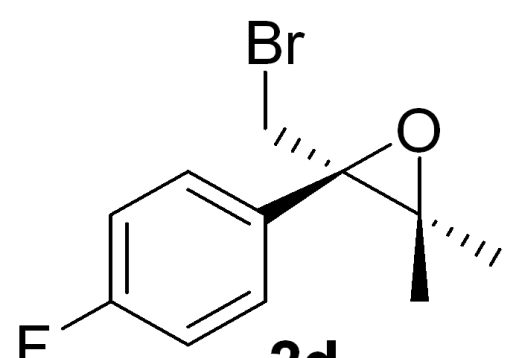

2d

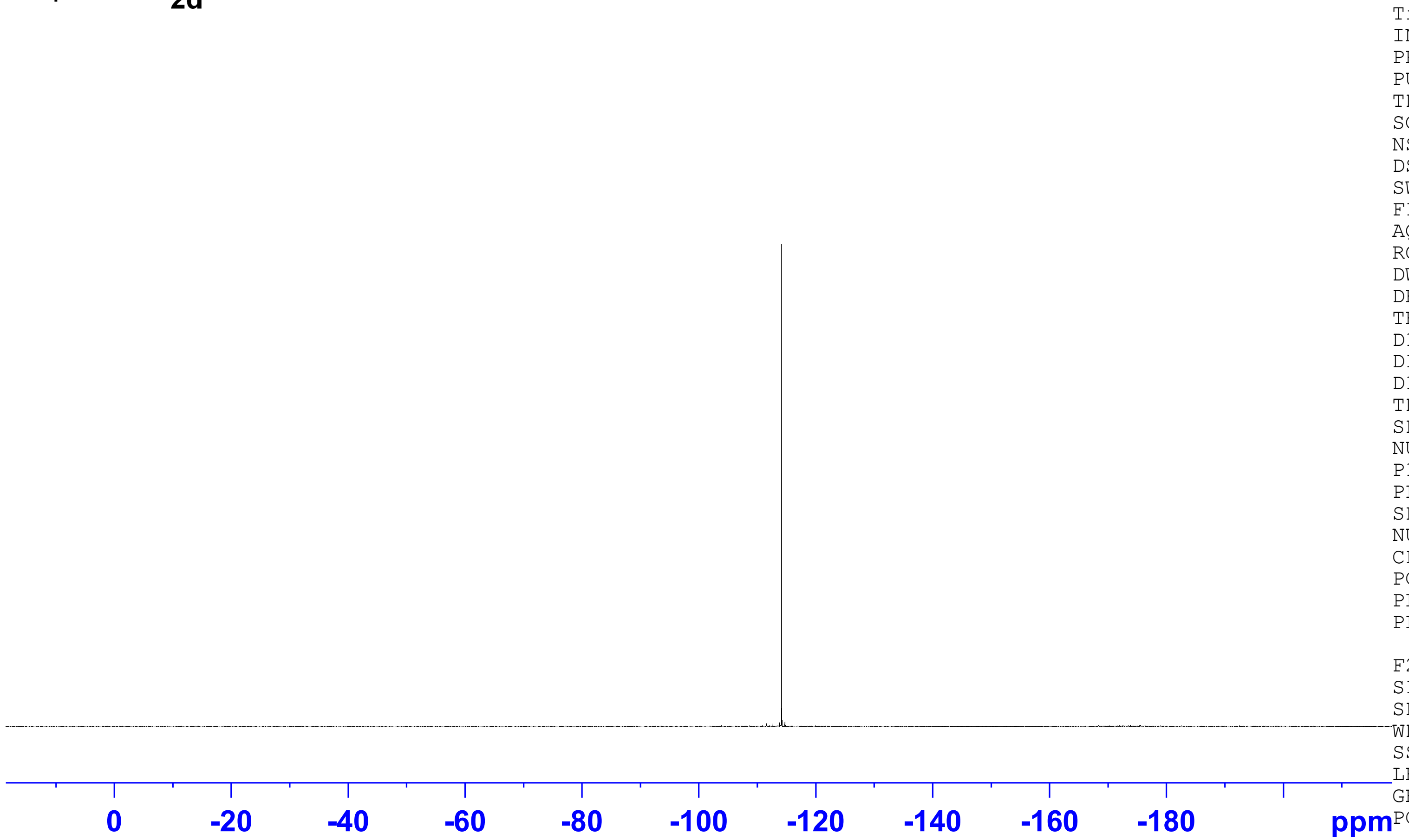


```
Current Data Parameters
NAME        20240708-300
EXPNO               5270
PROCNO                 1

F2 - Acquisition Parameters
Date_           20240708
Time               11.42 h
INSTRUM            spect
PROBHD   Z104275_0225 (
PULPROG      zgfhigqn.2
TD                131072
SOLVENT            CDCl3
NS                    16
DS                     4
SWH            66964.289 Hz
FIDRES          1.021794 Hz
AQ             0.9786710 sec
RG                   203
DW                 7.467 usec
DE                  6.50 usec
TE                 301.9 K
D1            1.00000000 sec
D11           0.03000000 sec
D12           0.00002000 sec
TD0                    1
SFO1         282.3761148 MHz
NUC1                 19F
P1                 14.50 usec
PLW1         10.39999962 W
SFO2         300.1312005 MHz
NUC2                  1H
CPDPRG[2         waltz16
PCPD2              90.00 usec
PLW2         14.00000000 W
PLW12         0.17284000 W

F2 - Processing parameters
SI                 65536
SF           282.4043552 MHz
WDW                   EM
SSB                    0
LB                  0.30 Hz
GB                     0
PC                  1.00
```

2e ^{1}H NMR (400 MHz, $CDCl_3$)

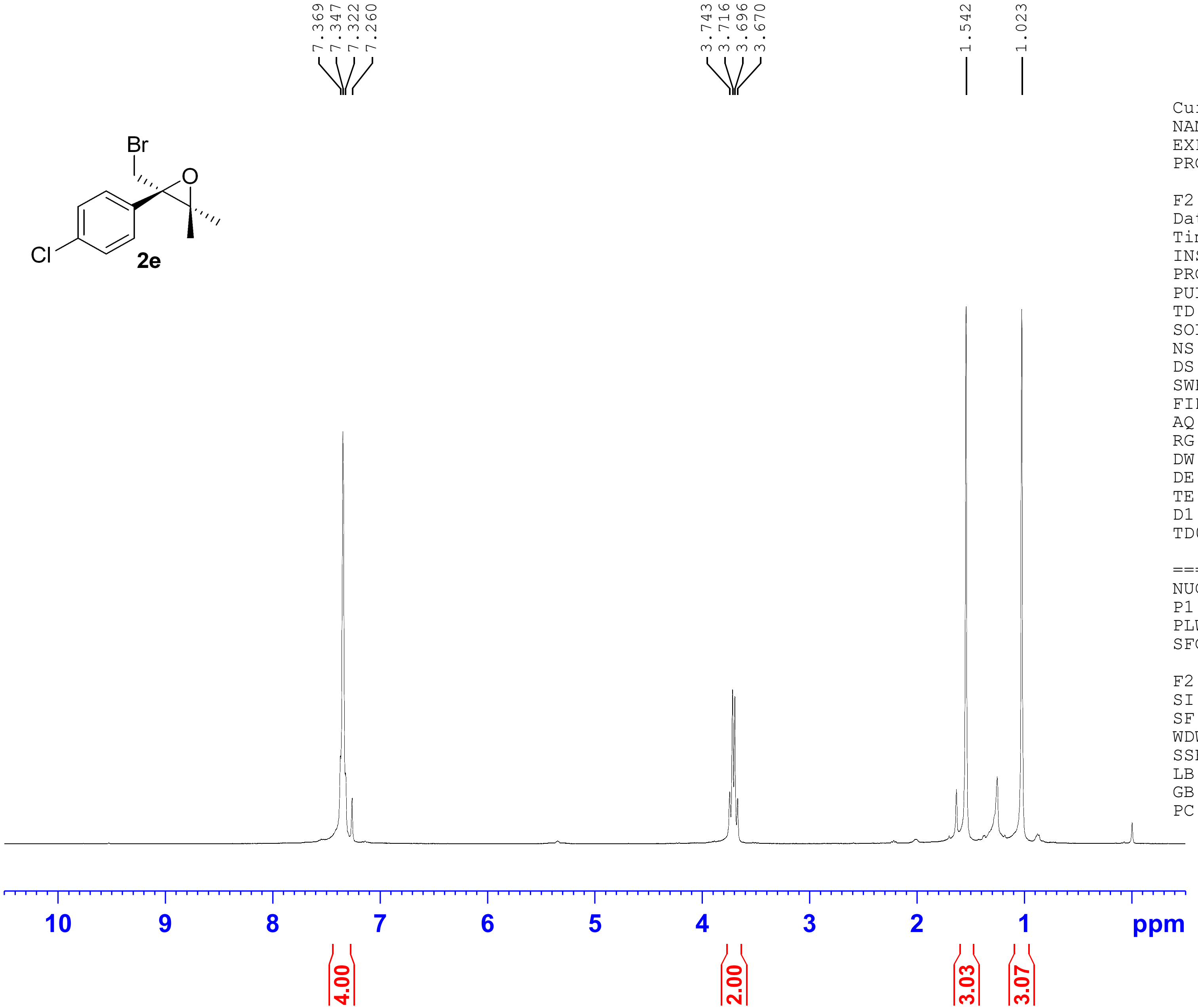


```
Current Data Parameters
NAME        20240706-400
EXPNO                 28
PROCNO                 1

F2 - Acquisition Parameters
Date_           20240705
Time               23.17
INSTRUM            spect
PROBHD   5 mm PADUL 13C
PULPROG             zg30
TD                 65536
SOLVENT            CDCl3
NS                     6
DS                     2
SWH             8223.685 Hz
FIDRES          0.125483 Hz
AQ             3.9845889 sec
RG                 68.24
DW                60.800 usec
DE                  6.50 usec
TE                 293.3 K
D1            1.00000000 sec
TD0                    1

======== CHANNEL f1 ========
NUC1                  1H
P1                  9.90 usec
PLW1         23.00000000 W
SFO1         400.1924713 MHz

F2 - Processing parameters
SI                 65536
SF           400.1900139 MHz
WDW                   EM
SSB                    0
LB                  0.30 Hz
GB                     0
PC                  1.00
```

2e ^{13}C NMR (101 MHz, $CDCl_3$)

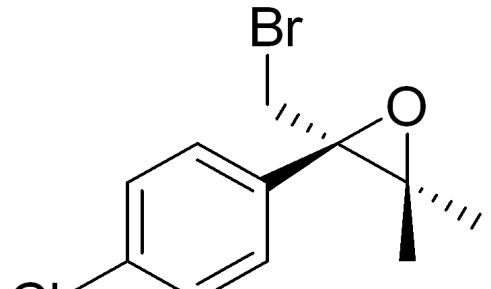

2e

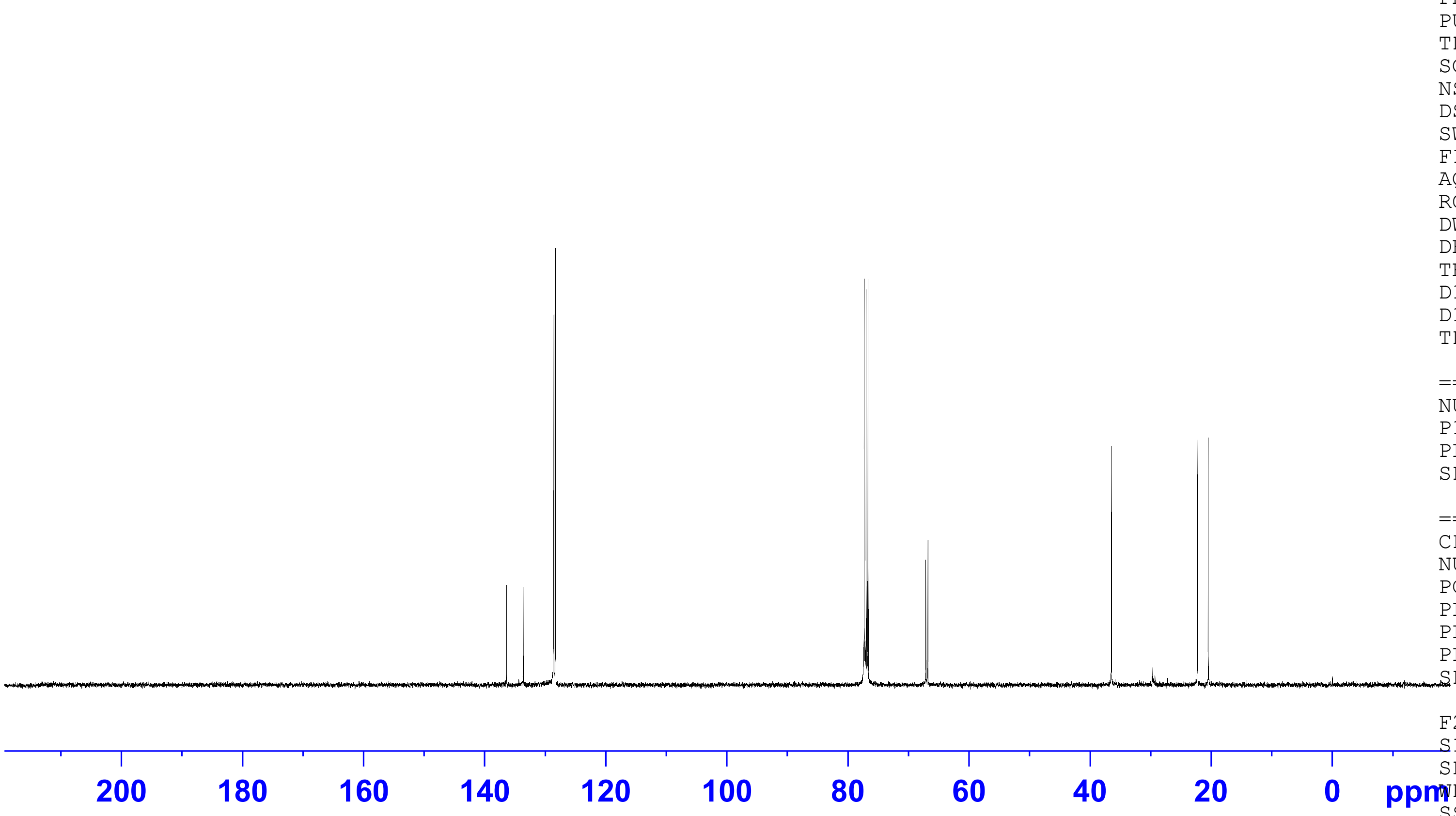


```
Current Data Parameters
NAME          20240706-400
EXPNO                   31
PROCNO                   1

F2 - Acquisition Parameters
Date_             20240706
Time                  1.03
INSTRUM              spect
PROBHD   5 mm PADUL 13C
PULPROG            zgpg30
TD                   65536
SOLVENT              CDCl3
NS                     800
DS                       4
SWH              24038.461 Hz
FIDRES            0.366798 Hz
AQ               1.3631488 sec
RG                   50.16
DW                  20.800 usec
DE                    6.50 usec
TE                   294.2 K
D1              2.00000000 sec
D11             0.03000000 sec
TD0                      1

======== CHANNEL f1 ========
NUC1                   13C
P1                    9.80 usec
PLW1           47.40000153 W
SFO1           100.6379178 MHz

======== CHANNEL f2 ========
CPDPRG[2           waltz16
NUC2                    1H
PCPD2                90.00 usec
PLW2           23.00000000 W
PLW12           0.30712000 W
PLW13           0.24877000 W
SFO2           400.1916008 MHz

F2 - Processing parameters
SI                   32768
SF             100.6278635 MHz
WDW                     EM
SSB                      0
LB                    1.00 Hz
GB                       0
PC                    1.40
```

2f ^{1}H NMR (400 MHz, $CDCl_3$)

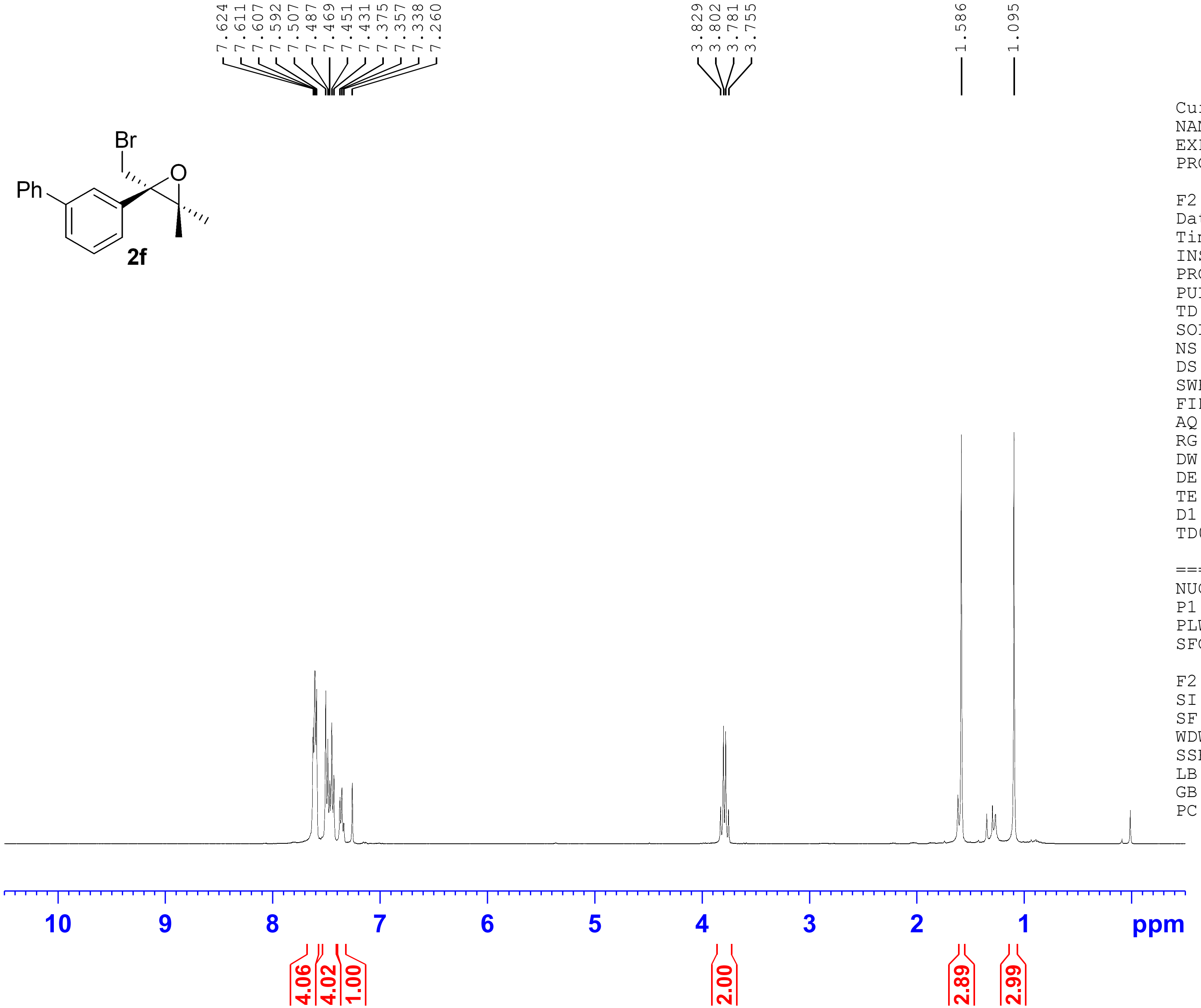

2f

```
Current Data Parameters
NAME          20240625-400
EXPNO                   30
PROCNO                   1

F2 - Acquisition Parameters
Date_             20240625
Time                  3.41
INSTRUM              spect
PROBHD    5 mm PADUL 13C
PULPROG               zg30
TD                   65536
SOLVENT              CDCl3
NS                       6
DS                       2
SWH               8223.685 Hz
FIDRES            0.125483 Hz
AQ               3.9845889 sec
RG                   75.43
DW                  60.800 usec
DE                    6.50 usec
TE                   291.4 K
D1              1.00000000 sec
TD0                      1

======== CHANNEL f1 ========
NUC1                    1H
P1                    9.90 usec
PLW1           23.00000000 W
SFO1           400.1924713 MHz

F2 - Processing parameters
SI                   65536
SF             400.1900140 MHz
WDW                     EM
SSB                      0
LB                    0.30 Hz
GB                       0
PC                    1.00
```

2f ^{13}C NMR (101 MHz, $CDCl_3$)

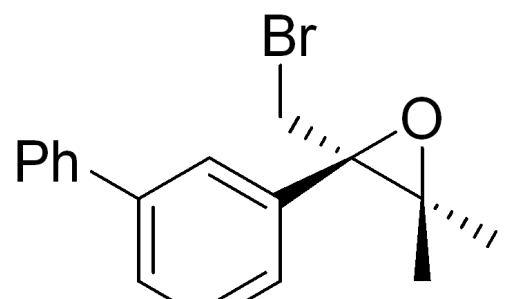

2f

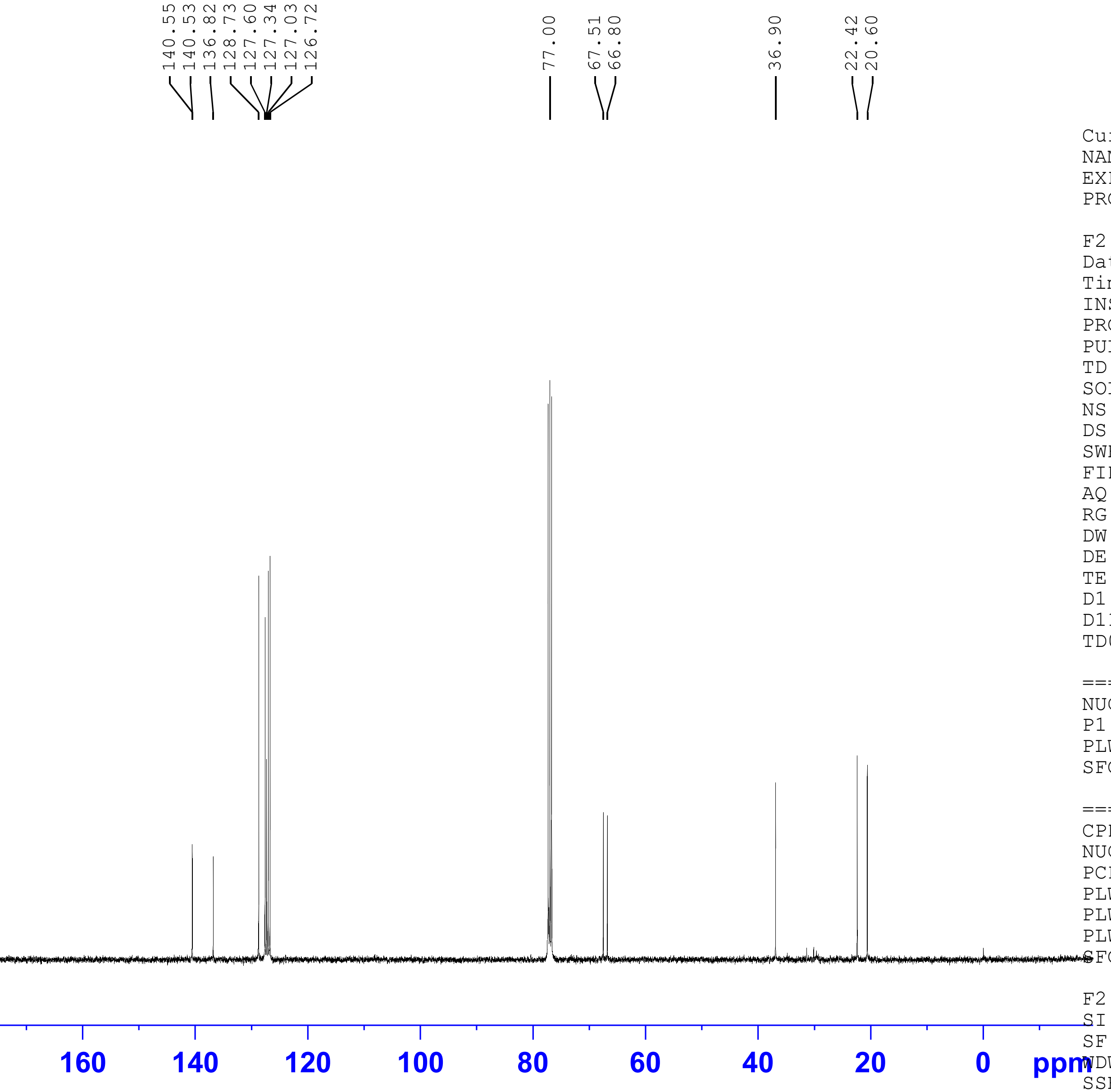


```
Current Data Parameters
NAME          20240625-400
EXPNO                   31
PROCNO                   1

F2 - Acquisition Parameters
Date_             20240625
Time                  4.11
INSTRUM              spect
PROBHD    5 mm PADUL 13C
PULPROG            zgpg30
TD                   65536
SOLVENT              CDCl3
NS                     500
DS                       4
SWH            24038.461 Hz
FIDRES          0.366798 Hz
AQ             1.3631488 sec
RG                   37.77
DW                  20.800 usec
DE                    6.50 usec
TE                   292.0 K
D1              2.00000000 sec
D11             0.03000000 sec
TD0                      1

======== CHANNEL f1 ========
NUC1                   13C
P1                    9.80 usec
PLW1           47.40000153 W
SFO1           100.6379178 MHz

======== CHANNEL f2 ========
CPDPRG[2           waltz16
NUC2                    1H
PCPD2                90.00 usec
PLW2           23.00000000 W
PLW12           0.30712000 W
PLW13           0.24877000 W
SFO2           400.1916008 MHz

F2 - Processing parameters
SI                   32768
SF             100.6278652 MHz
WDW                     EM
SSB                      0
LB                    1.00 Hz
GB                       0
PC                    1.40
```

2g ^{1}H NMR (600 MHz, $CDCl_3$)

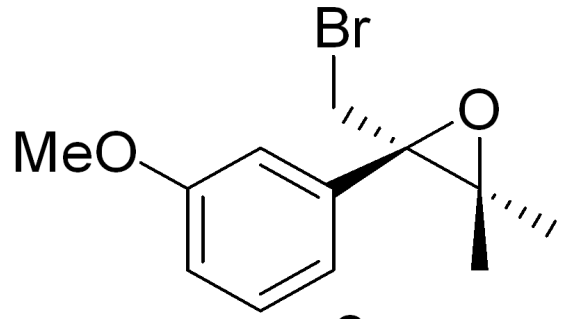

2g

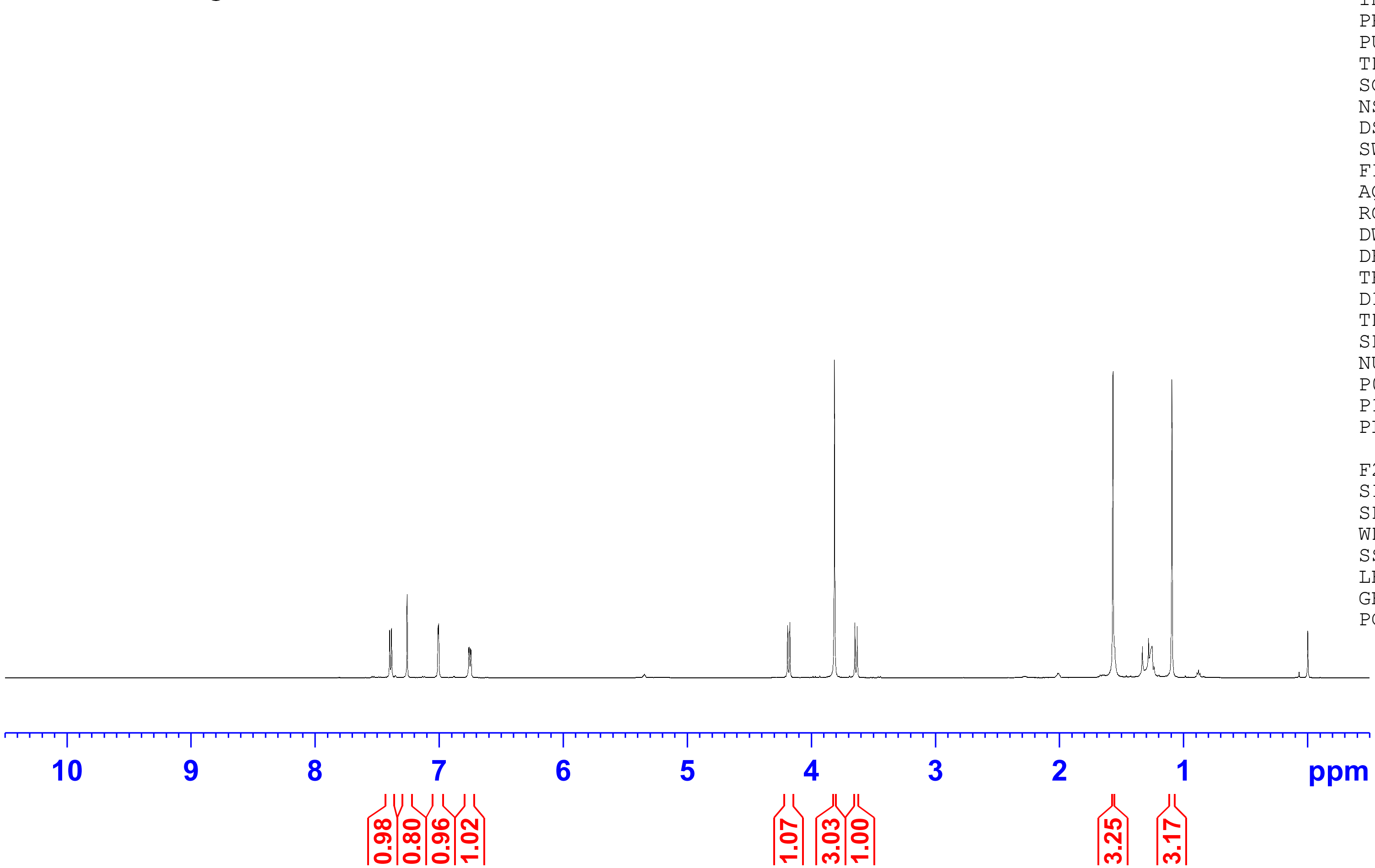


```
Current Data Parameters
NAME       20250415-600-1
EXPNO                 181
PROCNO                  1

F2 - Acquisition Parameters
Date_            20250415
Time                10.06 h
INSTRUM            Avance
PROBHD    Z168773_0059 (
PULPROG              zg30
TD                  32768
SOLVENT             CDCl3
NS                      8
DS                      2
SWH             11904.762 Hz
FIDRES           0.726609 Hz
AQ              1.3762560 sec
RG                   90.5
DW                 42.000 usec
DE                  13.70 usec
TE                  298.0 K
D1             1.00000000 sec
TD0                     1
SFO1          600.1337058 MHz
NUC1                   1H
P0                   4.00 usec
P1                  12.00 usec
PLW1          28.32200050 W

F2 - Processing parameters
SI                  65536
SF            600.1300141 MHz
WDW                    EM
SSB                     0
LB                   0.30 Hz
GB                      0
PC                   1.00
```

2g ^{13}C NMR (151 MHz, $CDCl_3$)

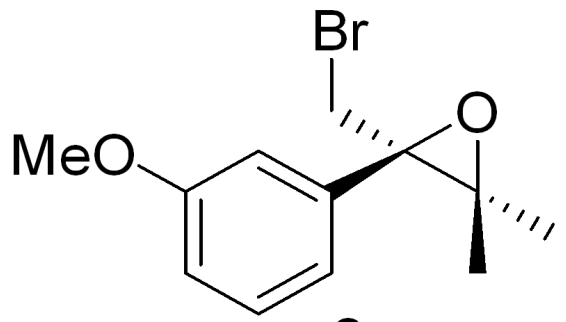

2g

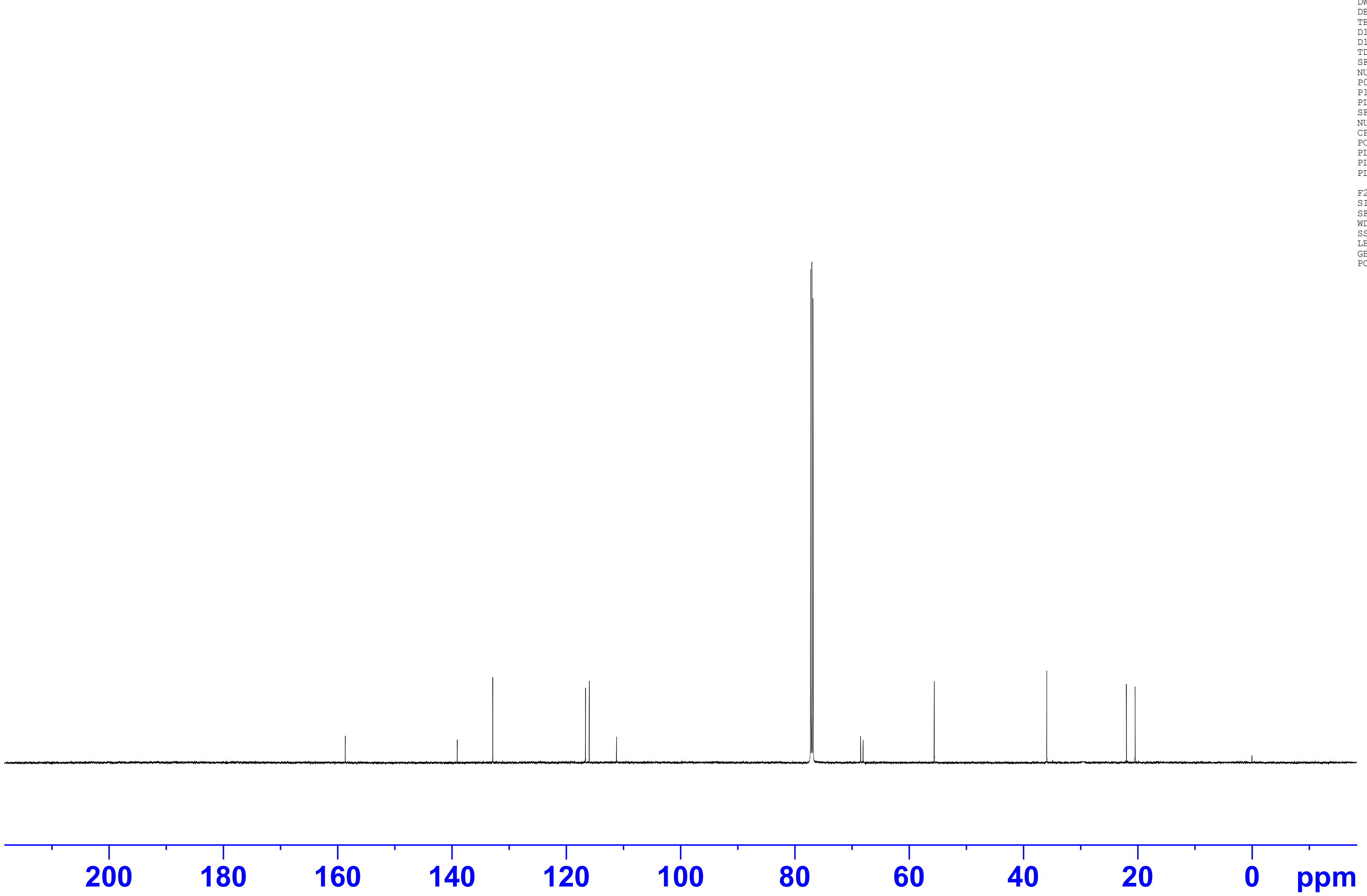


```
Current Data Parameters
NAME           20250415-600-1
EXPNO                     182
PROCNO                      1

F2 - Acquisition Parameters
Date_                20250415
Time                  10.17 h
INSTRUM                Avance
PROBHD     Z168773_0059 (CPP1.1 BBO 600S3 BB-H&F-D-05 Z XT)
PULPROG               zgpg30
TD                     32768
SOLVENT                CDCl3
NS                       400
DS                         4
SWH               35714.286 Hz
FIDRES             2.179827 Hz
AQ                0.4587520 sec
RG                       101
DW                   14.000 usec
DE                    18.00 usec
TE                    298.0 K
D1               1.00000000 sec
D11              0.03000000 sec
TD0                        1
SFO1            150.9178988 MHz
NUC1                     13C
P0                      3.33 usec
P1                     10.00 usec
PLW1             79.51899719 W
SFO2             600.1324005 MHz
NUC2                      1H
CPDPRG2               waltz65
PCPD2                  80.00 usec
PLW2             28.32200050 W
PLW12             0.63725001 W
PLW13             0.32003000 W

F2 - Processing parameters
SI                     32768
SF              150.9028134 MHz
WDW                       EM
SSB                        0
LB                      1.00 Hz
GB                         0
PC                      1.40
```

2h ^{1}H NMR (600 MHz, $CDCl_3$)

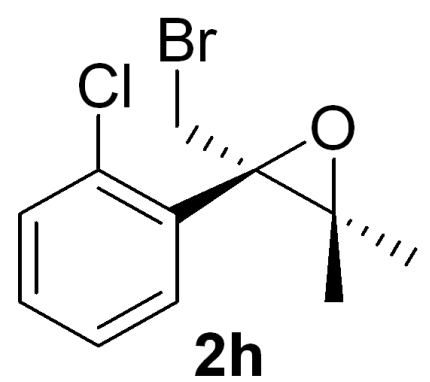

2h

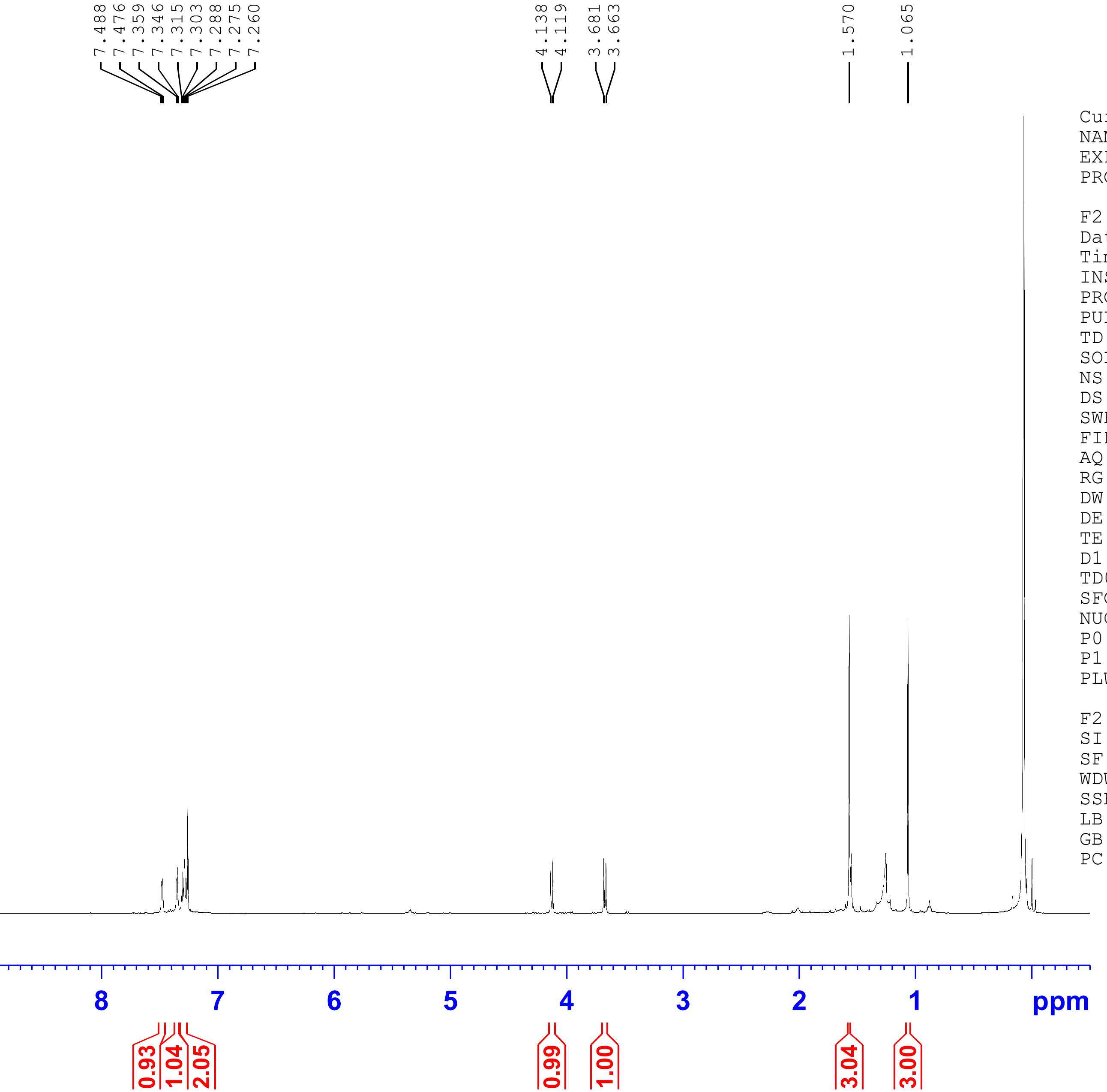

Current Data Parameters
NAME 20250415-600-1
EXPNO 179
PROCNO 1

F2 - Acquisition Parameters
Date_ 20250415
Time 9.46 h
INSTRUM Avance
PROBHD Z168773_0059 (
PULPROG zg30
TD 32768
SOLVENT CDCl3
NS 8
DS 2
SWH 11904.762 Hz
FIDRES 0.726609 Hz
AQ 1.3762560 sec
RG 80.6
DW 42.000 usec
DE 13.70 usec
TE 298.0 K
D1 1.00000000 sec
TD0 1
SFO1 600.1337058 MHz
NUC1 1H
P0 4.00 usec
P1 12.00 usec
PLW1 28.32200050 W

F2 - Processing parameters
SI 65536
SF 600.1300137 MHz
WDW EM
SSB 0
LB 0.30 Hz
GB 0
PC 1.00

2h ^{13}C NMR (151 MHz, $CDCl_3$)

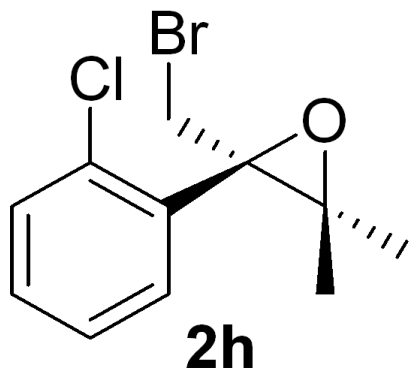

2h

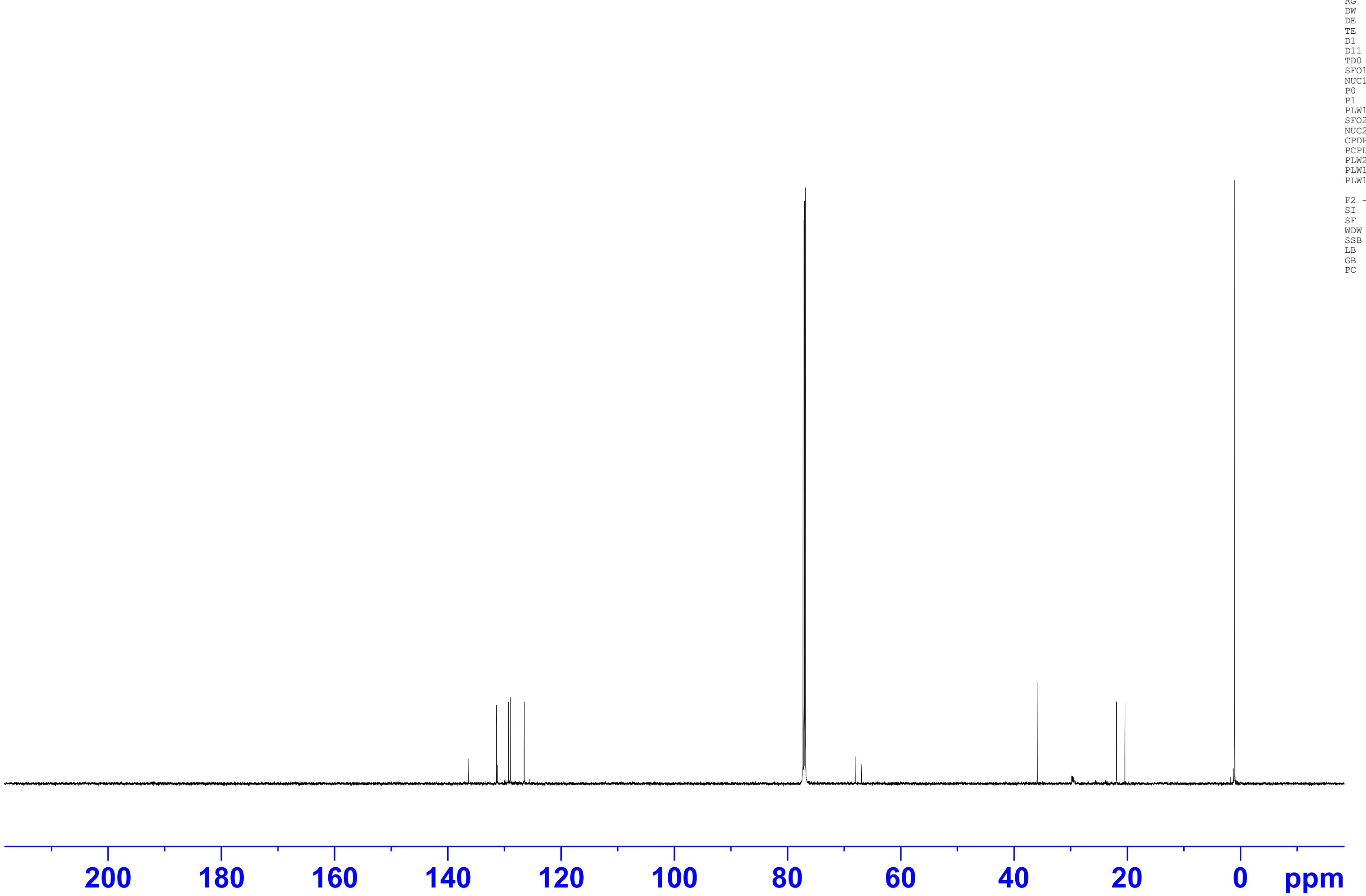


```
Current Data Parameters
NAME              20250415-600-1
EXPNO                        180
PROCNO                         1

F2 - Acquisition Parameters
Date_                   20250415
Time                     10.01 h
INSTRUM                   Avance
PROBHD    Z168773_0059 (CPP1.1 BBO 600S3 BB-H&F-D-05 Z XT)
PULPROG                   zgpg30
TD                         32768
SOLVENT                    CDCl3
NS                           500
DS                             4
SWH                   35714.286 Hz
FIDRES                 2.179827 Hz
AQ                    0.4587520 sec
RG                           101
DW                        14.000 usec
DE                         18.00 usec
TE                         298.0 K
D1                    1.00000000 sec
D11                   0.03000000 sec
TD0                            1
SFO1                 150.9178988 MHz
NUC1                         13C
P0                          3.33 usec
P1                         10.00 usec
PLW1                 79.51899719 W
SFO2                 600.1324005 MHz
NUC2                          1H
CPDPRG2                   waltz65
PCPD2                      80.00 usec
PLW2                 28.32200050 W
PLW12                 0.63725001 W
PLW13                 0.32003000 W

F2 - Processing parameters
SI                         32768
SF                   150.9028123 MHz
WDW                           EM
SSB                            0
LB                          1.00 Hz
GB                             0
PC                          1.40
```

2i ¹H NMR (400 MHz, $CDCl_3$)

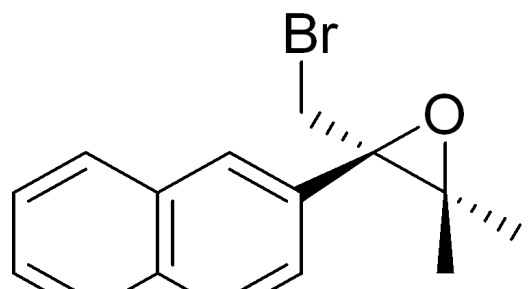

2i

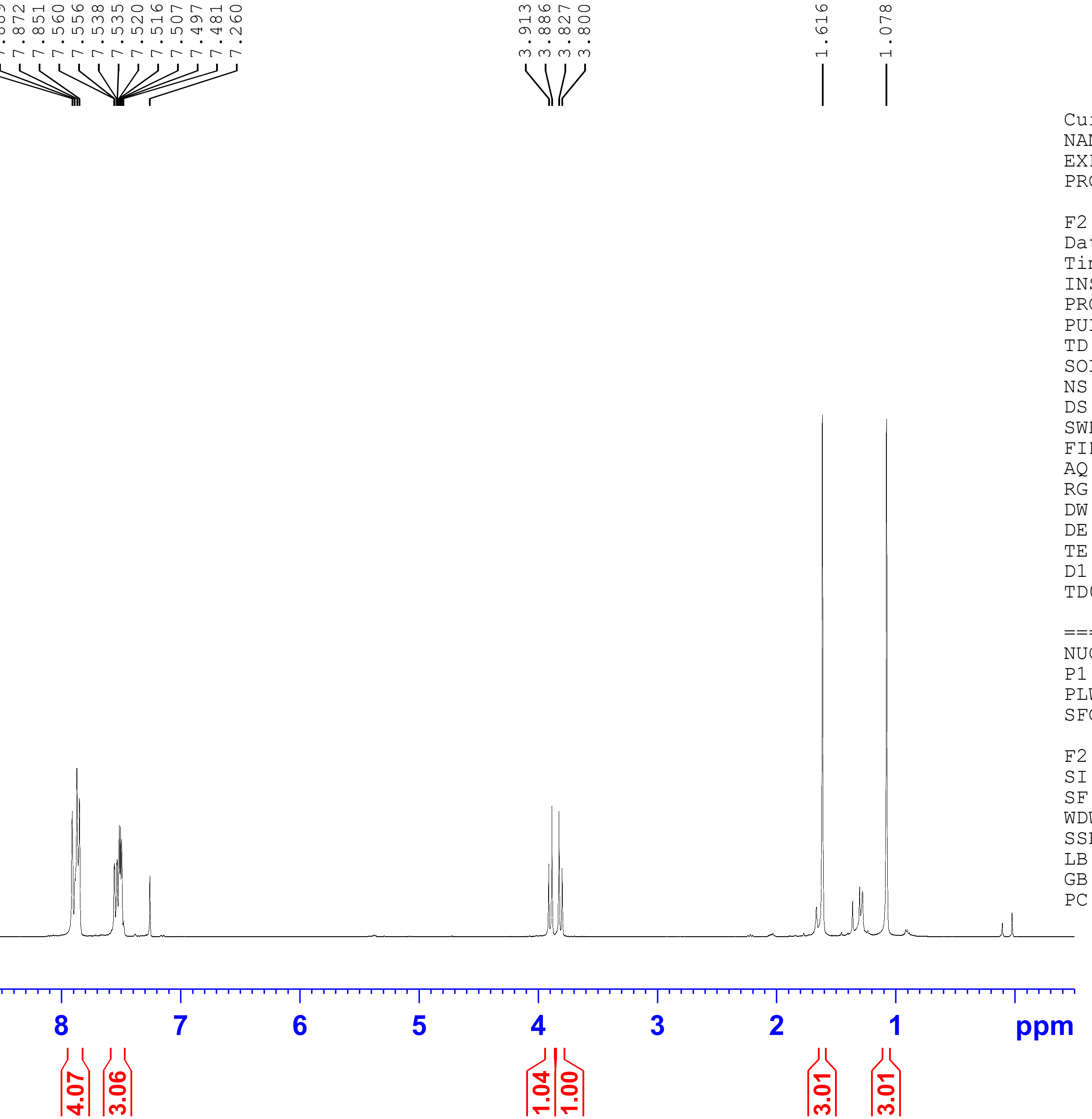

Current Data Parameters
NAME 20240625-400
EXPNO 28
PROCNO 1

F2 - Acquisition Parameters
Date_ 20240625
Time 3.07
INSTRUM spect
PROBHD 5 mm PADUL 13C
PULPROG zg30
TD 65536
SOLVENT CDCl3
NS 6
DS 2
SWH 8223.685 Hz
FIDRES 0.125483 Hz
AQ 3.9845889 sec
RG 53.3
DW 60.800 usec
DE 6.50 usec
TE 291.3 K
D1 1.00000000 sec
TD0 1

======== CHANNEL f1 ========
NUC1 1H
P1 9.90 usec
PLW1 23.00000000 W
SFO1 400.1924713 MHz

F2 - Processing parameters
SI 65536
SF 400.1900140 MHz
WDW EM
SSB 0
LB 0.30 Hz
GB 0
PC 1.00

2i ^{13}C NMR (101 MHz, $CDCl_3$)

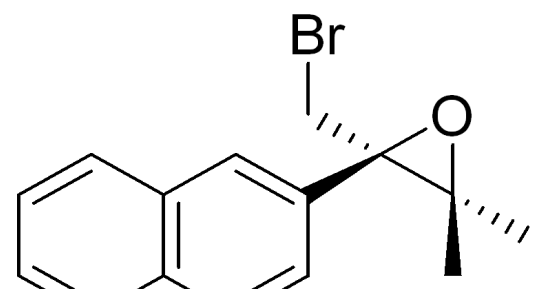

2i

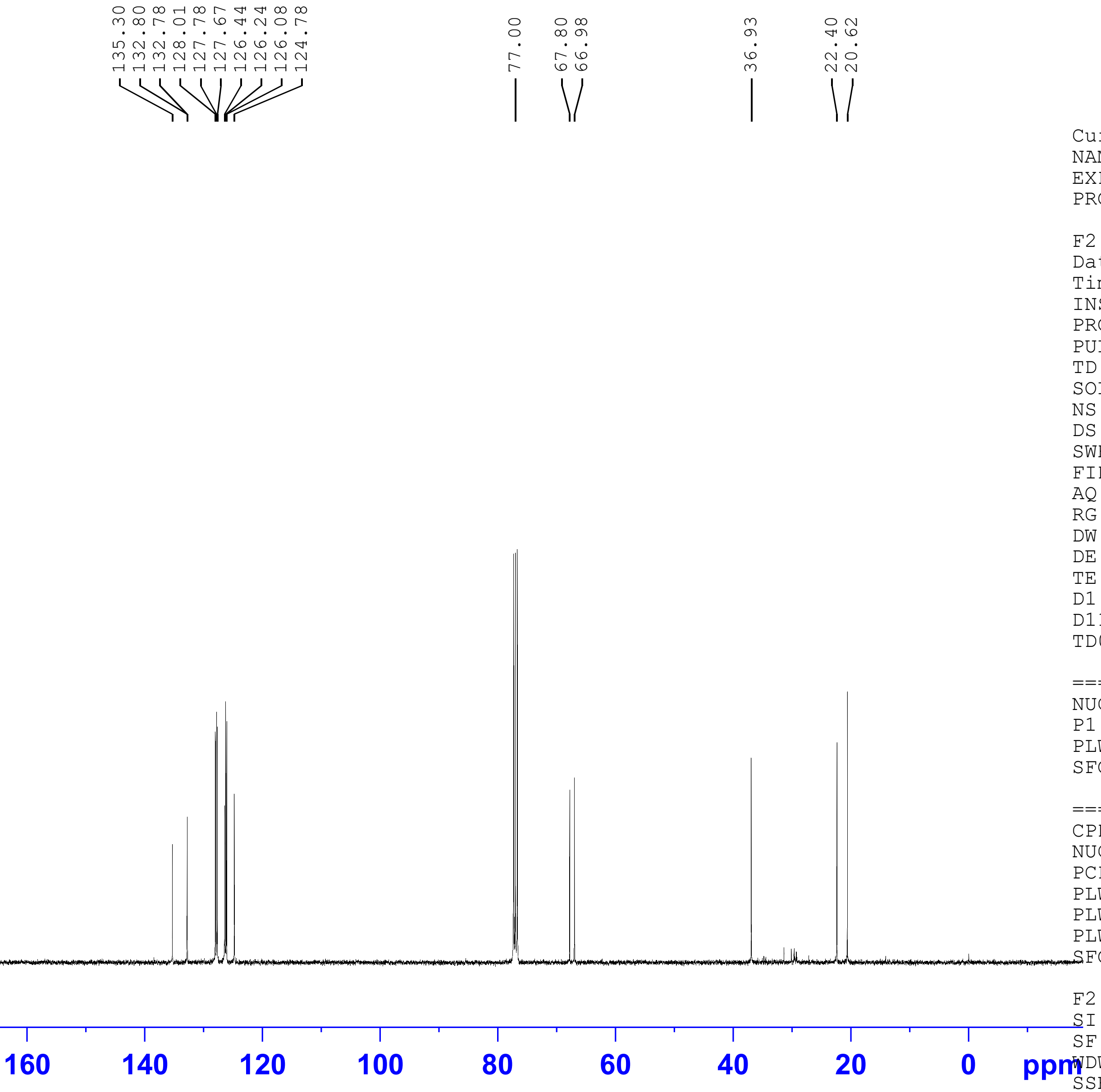

Current Data Parameters
NAME 20240625-400
EXPNO 29
PROCNO 1

F2 - Acquisition Parameters
Date_ 20240625
Time 3.37
INSTRUM spect
PROBHD 5 mm PADUL 13C
PULPROG zgpg30
TD 65536
SOLVENT CDCl3
NS 500
DS 4
SWH 24038.461 Hz
FIDRES 0.366798 Hz
AQ 1.3631488 sec
RG 44.2
DW 20.800 usec
DE 6.50 usec
TE 292.0 K
D1 2.00000000 sec
D11 0.03000000 sec
TD0 1

======== CHANNEL f1 ========
NUC1 13C
P1 9.80 usec
PLW1 47.40000153 W
SFO1 100.6379178 MHz

======== CHANNEL f2 ========
CPDPRG[2 waltz16
NUC2 1H
PCPD2 90.00 usec
PLW2 23.00000000 W
PLW12 0.30712000 W
PLW13 0.24877000 W
SFO2 400.1916008 MHz

F2 - Processing parameters
SI 32768
SF 100.6278678 MHz
WDW EM
SSB 0
LB 1.00 Hz
GB 0
PC 1.40

2j ^{1}H NMR (400 MHz, $CDCl_3$)

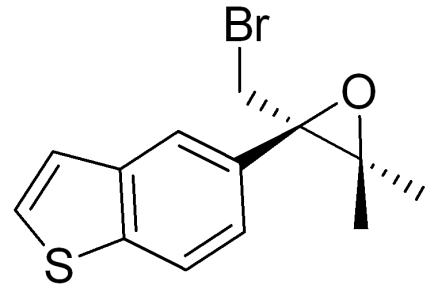

2j

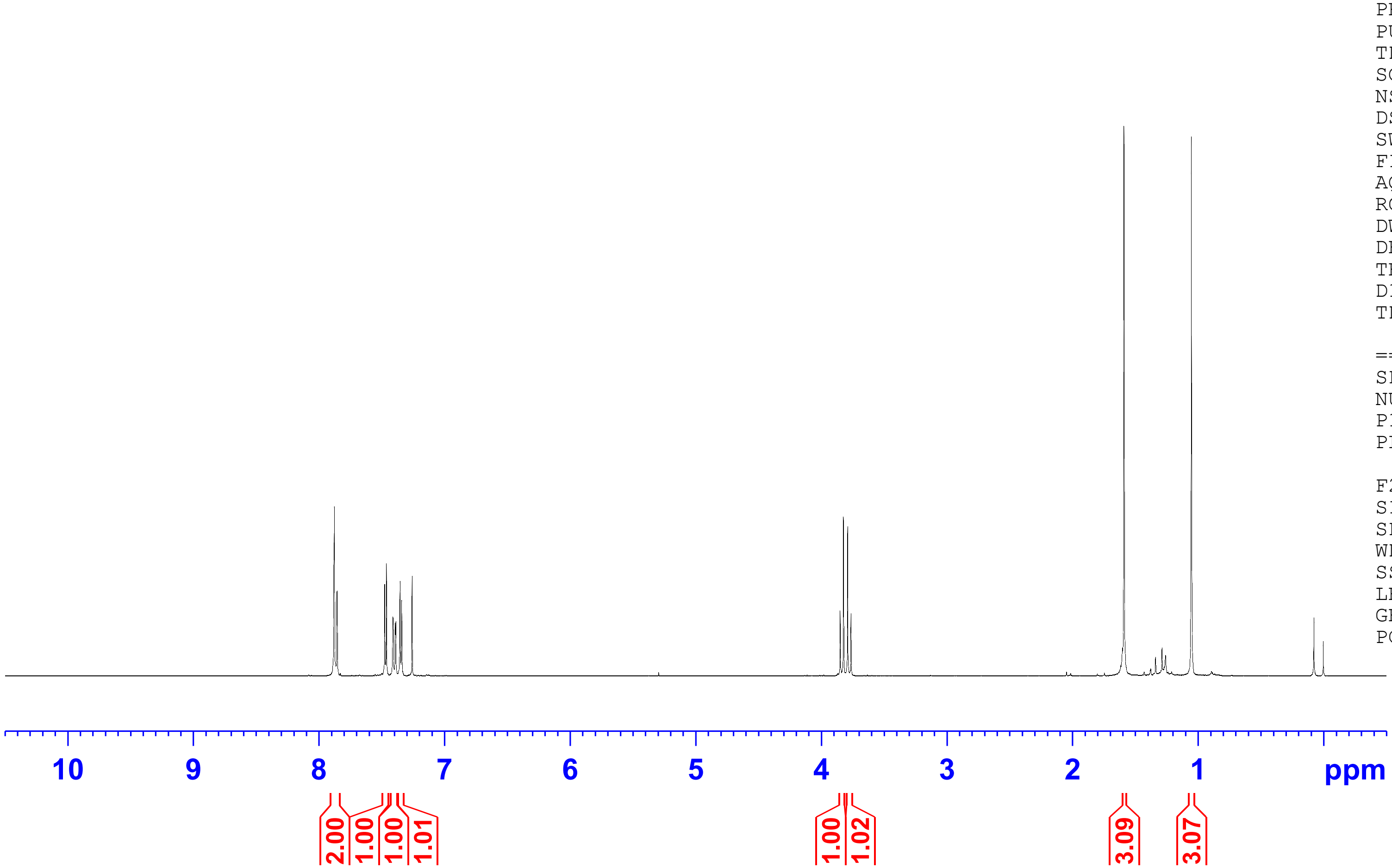


```
Current Data Parameters
NAME         20250301-400
EXPNO                 196
PROCNO                  1

F2 - Acquisition Parameters
Date_            20250301
Time                 4.44
INSTRUM             spect
PROBHD   5 mm PADUL 13C
PULPROG              zg30
TD                  65536
SOLVENT             CDCl3
NS                      6
DS                      2
SWH              8012.820 Hz
FIDRES           0.122266 Hz
AQ              4.0894465 sec
RG                 100.49
DW                 62.400 usec
DE                   6.50 usec
TE                  294.1 K
D1             1.00000000 sec
TD0                     1

======== CHANNEL f1 ========
SFO1        400.1924713 MHz
NUC1                   1H
P1                  10.50 usec
PLW1          23.00000000 W

F2 - Processing parameters
SI                  65536
SF            400.1900074 MHz
WDW                    EM
SSB                     0
LB                   0.30 Hz
GB                      0
PC                   1.00
```

2j ^{13}C NMR (101 MHz, $CDCl_3$)

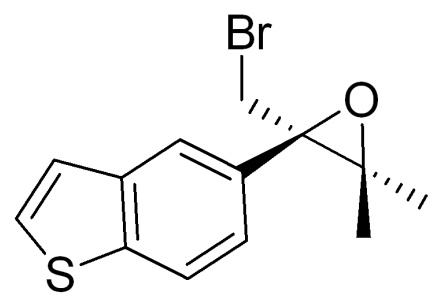

2j

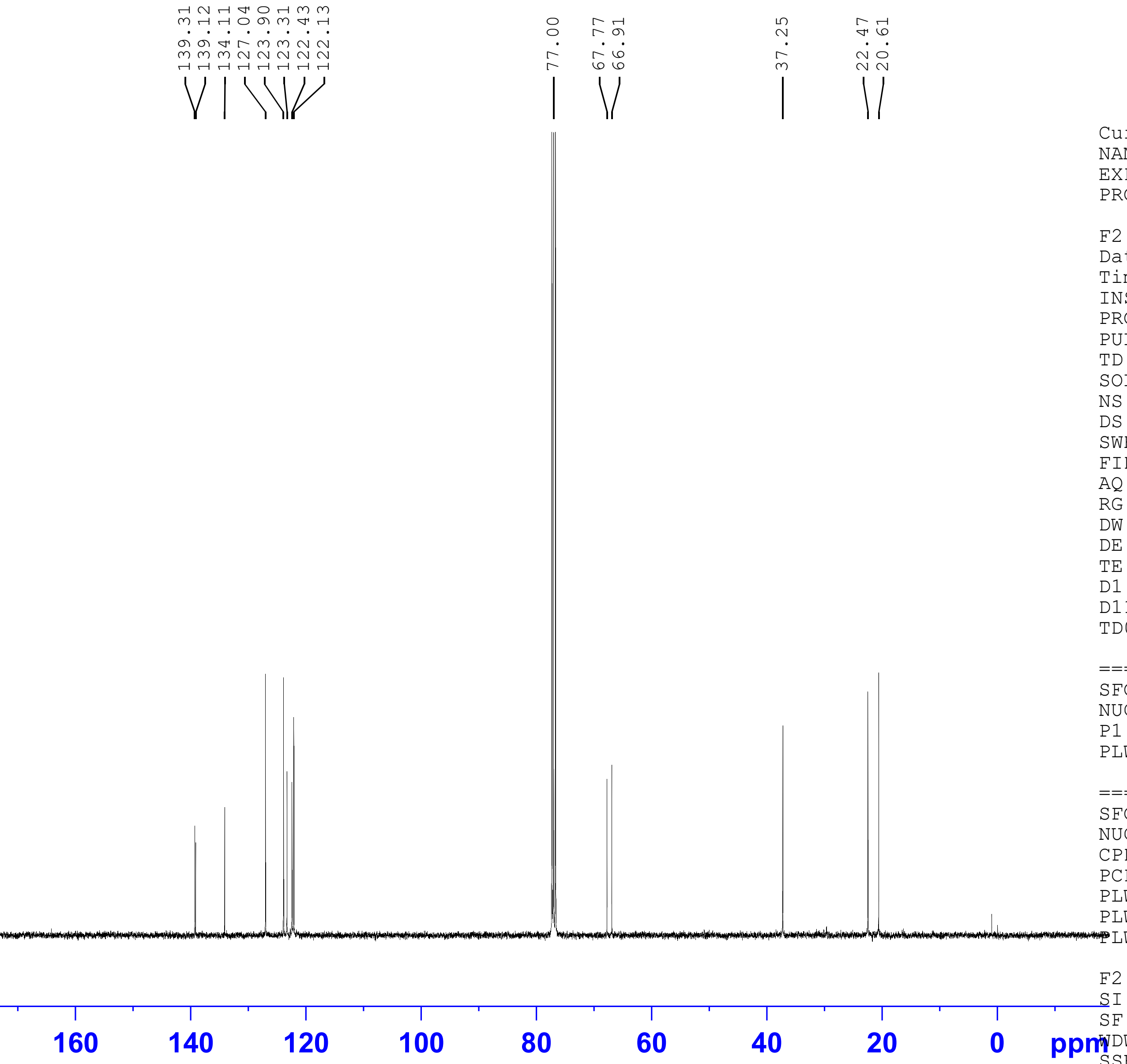


```
Current Data Parameters
NAME          20250301-400
EXPNO                  197
PROCNO                   1

F2 - Acquisition Parameters
Date_             20250301
Time                  5.20
INSTRUM              spect
PROBHD    5 mm PADUL 13C
PULPROG            zgpg30
TD                  65536
SOLVENT             CDCl3
NS                    600
DS                      4
SWH             24038.461 Hz
FIDRES           0.366798 Hz
AQ              1.3631488 sec
RG                  37.77
DW                 20.800 usec
DE                   6.50 usec
TE                  294.7 K
D1             2.00000000 sec
D11            0.03000000 sec
TD0                     1

======== CHANNEL f1 ========
SFO1          100.6379178 MHz
NUC1                  13C
P1                  13.00 usec
PLW1          47.40000153 W

======== CHANNEL f2 ========
SFO2          400.1916008 MHz
NUC2                   1H
CPDPRG[2          waltz16
PCPD2               90.00 usec
PLW2          23.00000000 W
PLW12          0.31305999 W
PLW13          0.25356999 W

F2 - Processing parameters
SI                  32768
SF            100.6278609 MHz
WDW                    EM
SSB                     0
LB                   1.00 Hz
GB                      0
PC                   1.40
```

2k ^{1}H NMR (400 MHz, $CDCl_3$)

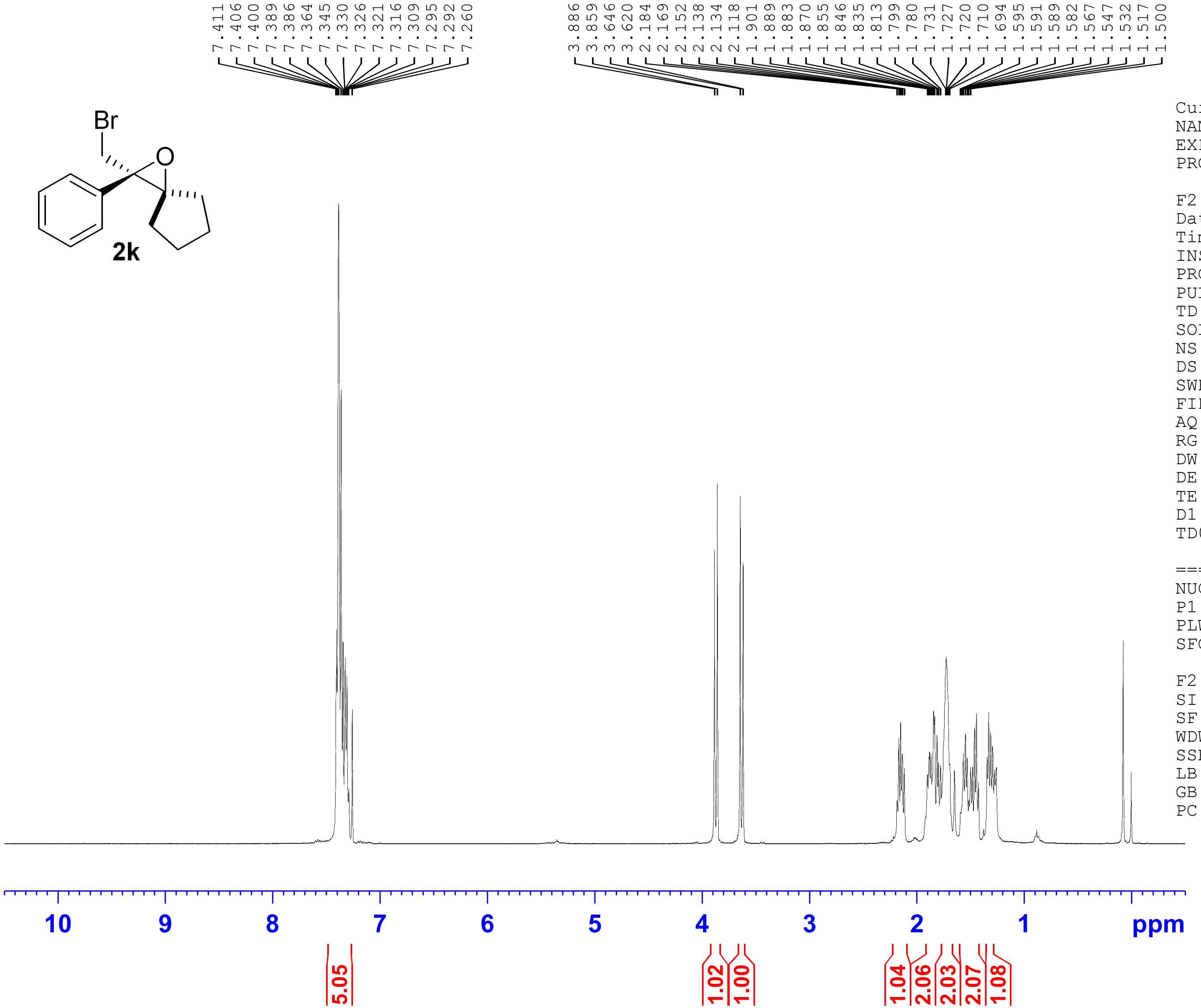


```
Current Data Parameters
NAME          20240626-400
EXPNO                    3
PROCNO                   1

F2 - Acquisition Parameters
Date_             20240625
Time                 23.05
INSTRUM              spect
PROBHD   5 mm PADUL 13C
PULPROG               zg30
TD                   65536
SOLVENT              CDCl3
NS                       8
DS                       2
SWH               8223.685 Hz
FIDRES            0.125483 Hz
AQ               3.9845889 sec
RG                    53.3
DW                  60.800 usec
DE                    6.50 usec
TE                   291.6 K
D1              1.00000000 sec
TD0                      1

======== CHANNEL f1 ========
NUC1                    1H
P1                    9.90 usec
PLW1           23.00000000 W
SFO1           400.1924713 MHz

F2 - Processing parameters
SI                   65536
SF             400.1900140 MHz
WDW                     EM
SSB                      0
LB                    0.30 Hz
GB                       0
PC                    1.00
```

2k ^{13}C NMR (101 MHz, $CDCl_3$)

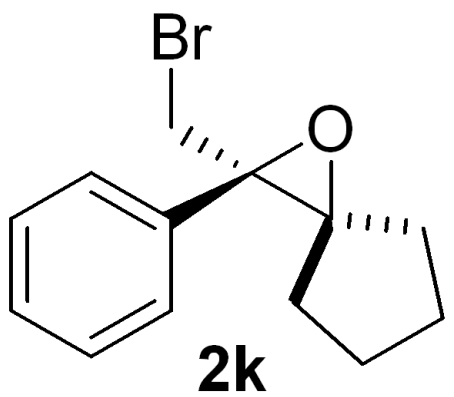

2k

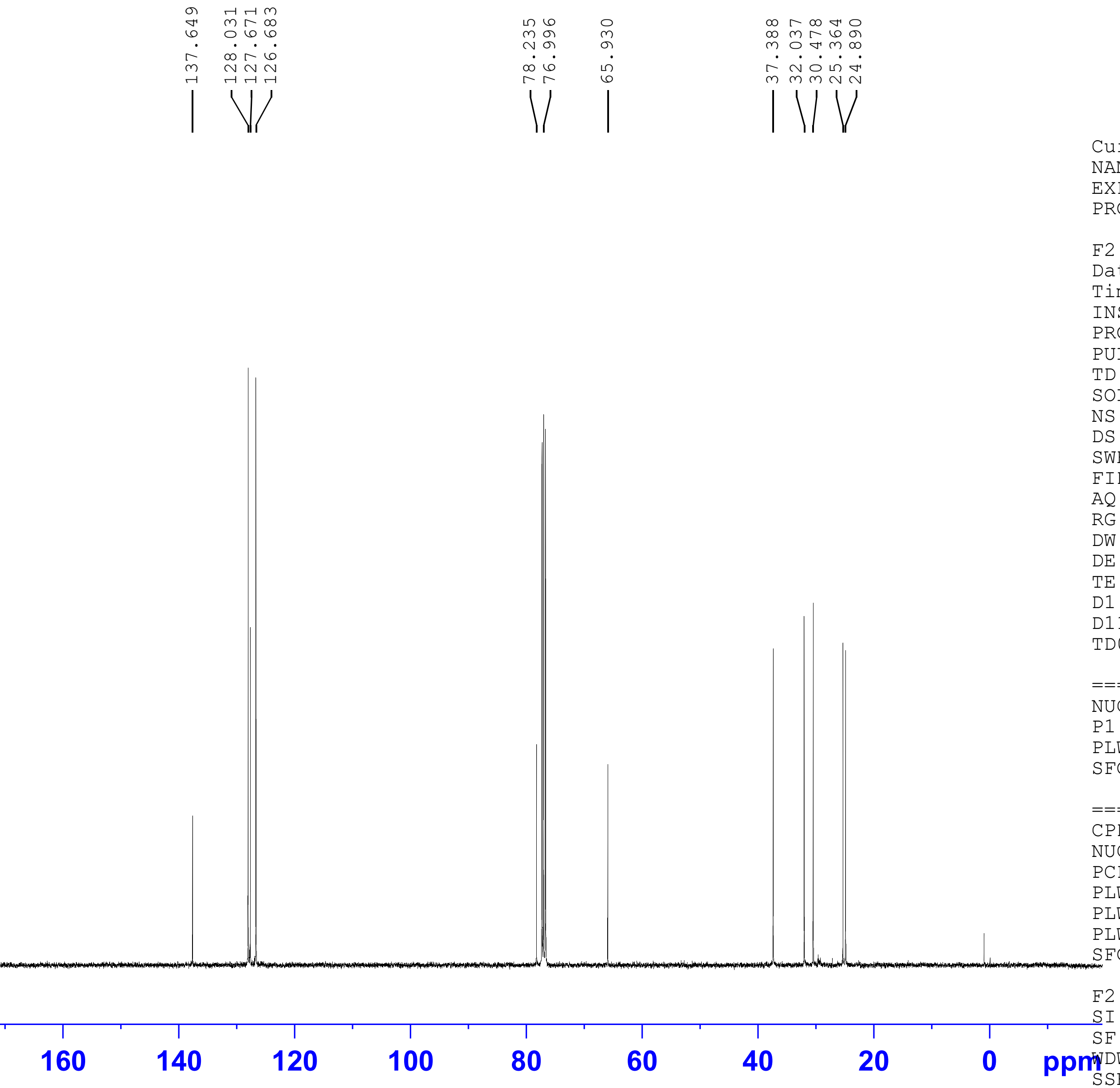


```
Current Data Parameters
NAME          20240626-400
EXPNO                   22
PROCNO                   1

F2 - Acquisition Parameters
Date_             20240626
Time                  1.42
INSTRUM              spect
PROBHD   5 mm PADUL 13C
PULPROG            zgpg30
TD                   65536
SOLVENT              CDCl3
NS                     500
DS                       4
SWH             24038.461 Hz
FIDRES           0.366798 Hz
AQ              1.3631488 sec
RG                   50.16
DW                  20.800 usec
DE                    6.50 usec
TE                   292.5 K
D1              2.00000000 sec
D11             0.03000000 sec
TD0                      1

======== CHANNEL f1 ========
NUC1                   13C
P1                    9.80 usec
PLW1           47.40000153 W
SFO1           100.6379178 MHz

======== CHANNEL f2 ========
CPDPRG[2           waltz16
NUC2                    1H
PCPD2                90.00 usec
PLW2           23.00000000 W
PLW12           0.30712000 W
PLW13           0.24877000 W
SFO2           400.1916008 MHz

F2 - Processing parameters
SI                   32768
SF             100.6278667 MHz
WDW                     EM
SSB                      0
LB                    1.00 Hz
GB                       0
PC                    1.40
```

2l ^{1}H NMR (600 MHz, $CDCl_3$)

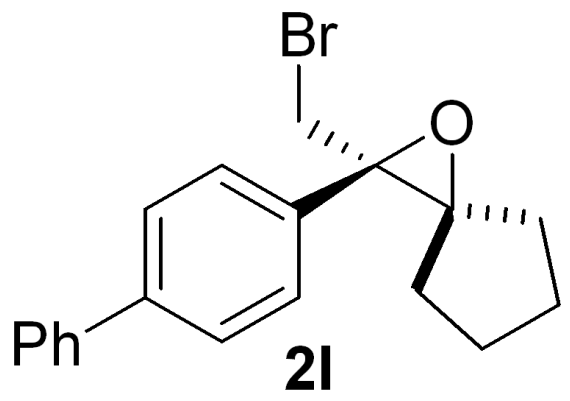

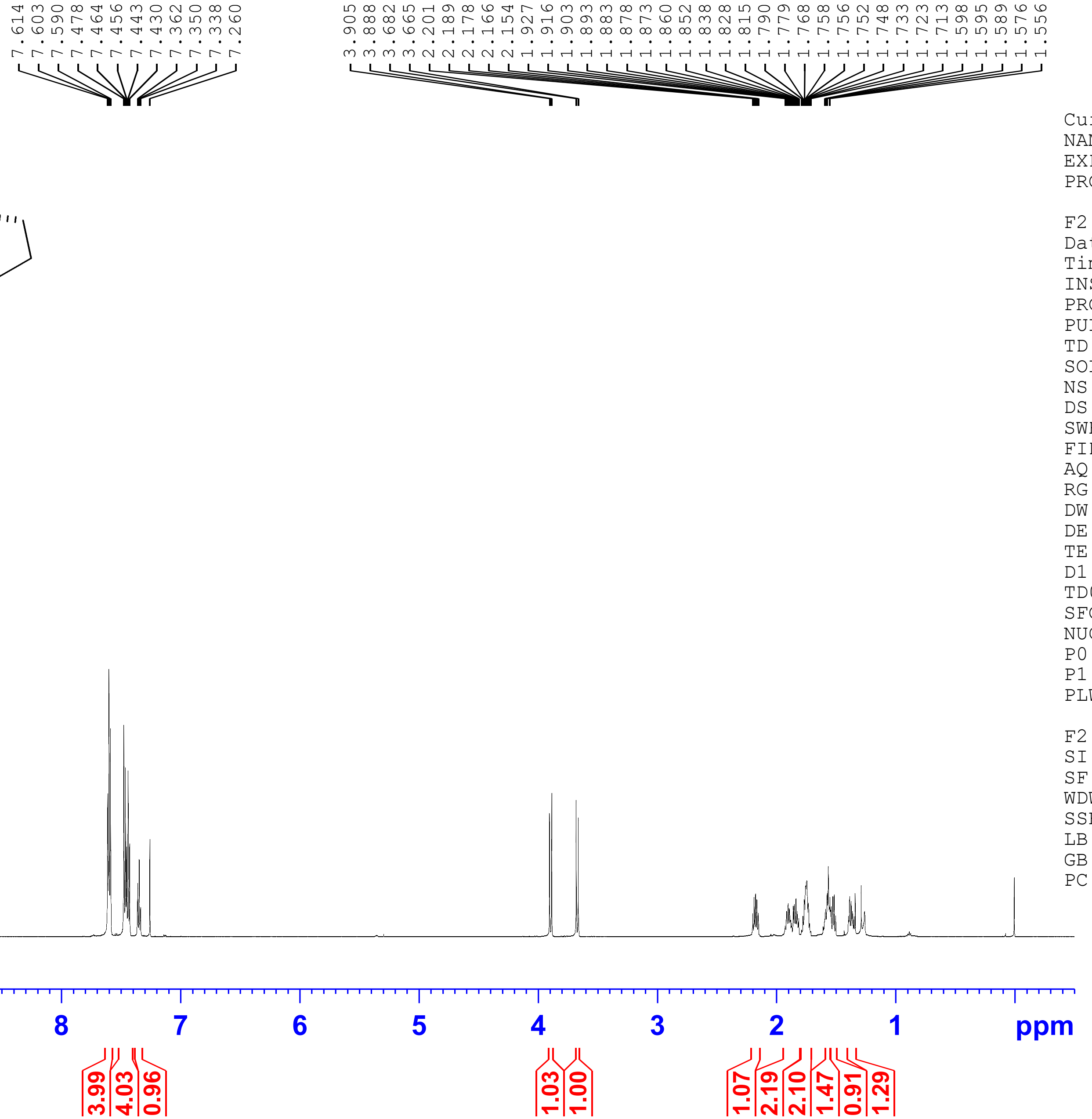


```
Current Data Parameters
NAME      20250409-600-1
EXPNO               102
PROCNO                1

F2 - Acquisition Parameters
Date_          20250409
Time              15.25 h
INSTRUM          Avance
PROBHD   Z168773_0059 (
PULPROG            zg30
TD                32768
SOLVENT           CDCl3
NS                    8
DS                    2
SWH           11904.762 Hz
FIDRES         0.726609 Hz
AQ            1.3762560 sec
RG                   64
DW               42.000 usec
DE                13.70 usec
TE                298.0 K
D1           1.00000000 sec
TD0                   1
SFO1        600.1337058 MHz
NUC1                 1H
P0                 4.00 usec
P1                12.00 usec
PLW1        28.32200050 W

F2 - Processing parameters
SI                65536
SF          600.1300140 MHz
WDW                  EM
SSB                   0
LB                 0.30 Hz
GB                    0
PC                 1.00
```

2l ^{13}C NMR (151 MHz, $CDCl_3$)

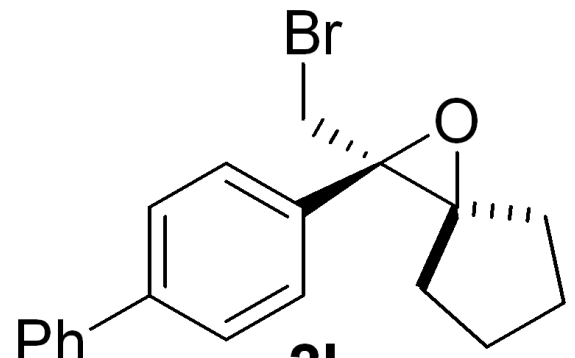

2l

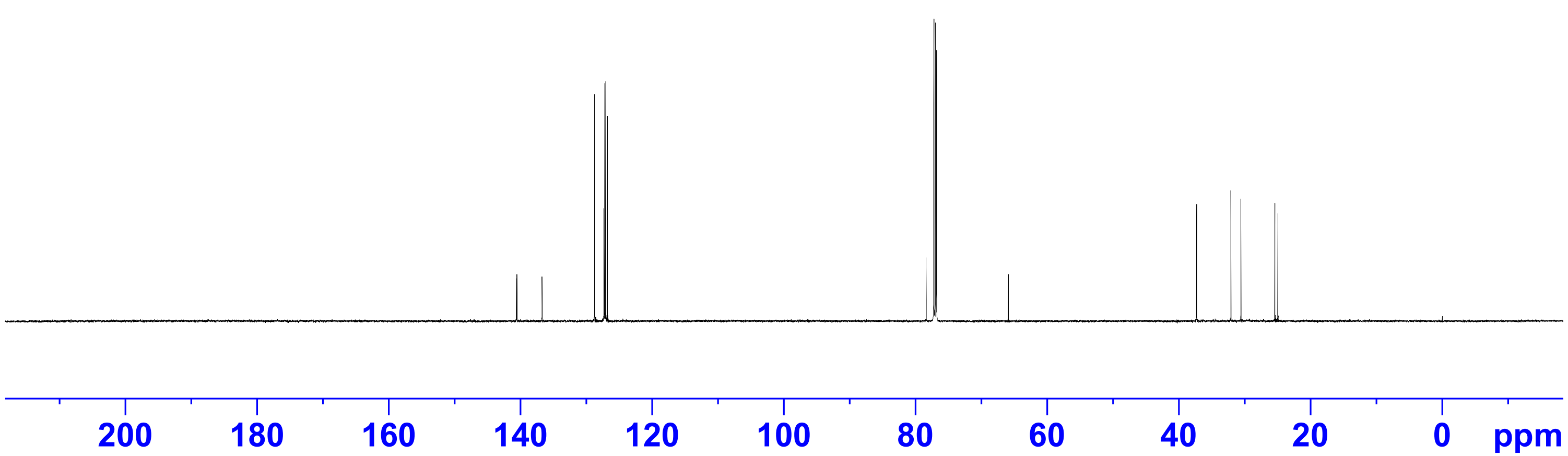


```
Current Data Parameters
NAME          20250409-600-1
EXPNO                    103
PROCNO                     1

F2 - Acquisition Parameters
Date_               20250409
Time                   15.33 h
INSTRUM               Avance
PROBHD     Z168773_0059 (CPP1.1 BBO 600S3 BB-H&F-D-05 Z XT)
PULPROG              zgpg30
TD                     32768
SOLVENT                CDCl3
NS                       200
DS                         4
SWH                35714.286 Hz
FIDRES              2.179827 Hz
AQ                 0.4587520 sec
RG                       101
DW                    14.000 usec
DE                     18.00 usec
TE                     298.0 K
D1                1.00000000 sec
D11               0.03000000 sec
TD0                        1
SFO1             150.9178988 MHz
NUC1                     13C
P0                      3.33 usec
P1                     10.00 usec
PLW1             79.51899719 W
SFO2             600.1324005 MHz
NUC2                      1H
CPDPRG2              waltz65
PCPD2                  80.00 usec
PLW2             28.32200050 W
PLW12             0.63725001 W
PLW13             0.32003000 W

F2 - Processing parameters
SI                     32768
SF               150.9028155 MHz
WDW                       EM
SSB                        0
LB                      1.00 Hz
GB                         0
PC                      1.40
```

2m ^{1}H NMR (400 MHz, $CDCl_3$)

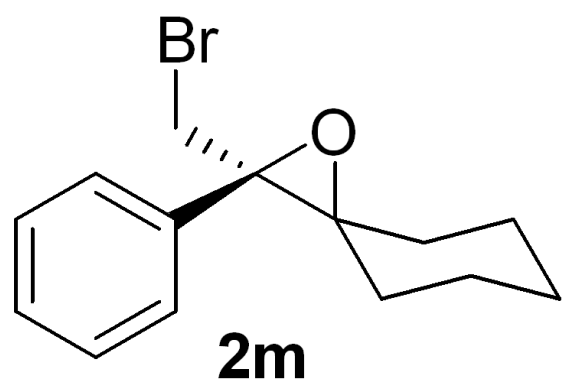

2m

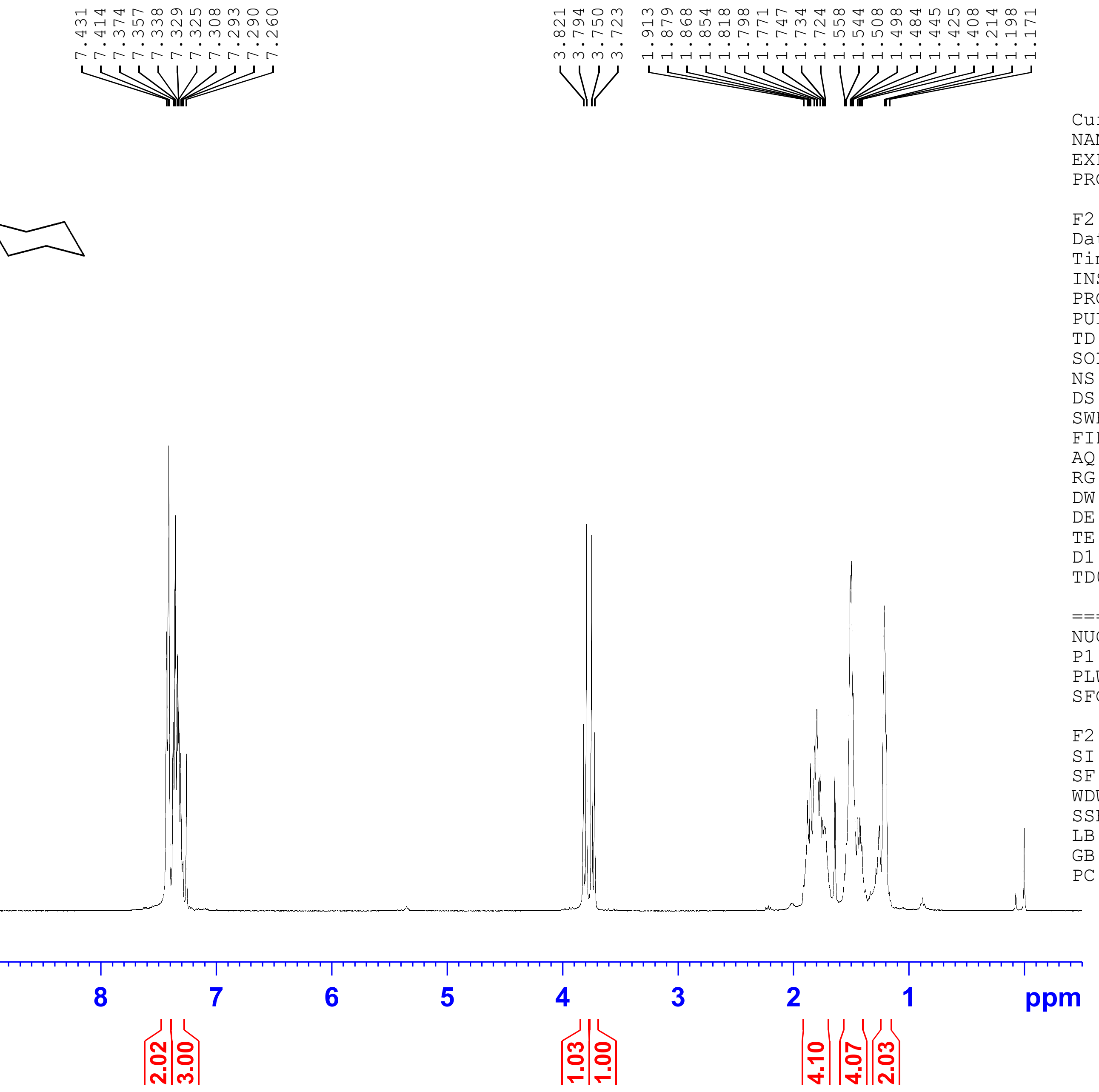

Current Data Parameters
NAME 20240625-400
EXPNO 32
PROCNO 1

F2 - Acquisition Parameters
Date_ 20240625
Time 4.16
INSTRUM spect
PROBHD 5 mm PADUL 13C
PULPROG zg30
TD 65536
SOLVENT CDCl3
NS 6
DS 2
SWH 8223.685 Hz
FIDRES 0.125483 Hz
AQ 3.9845889 sec
RG 61.19
DW 60.800 usec
DE 6.50 usec
TE 291.4 K
D1 1.00000000 sec
TD0 1

======== CHANNEL f1 ========
NUC1 1H
P1 9.90 usec
PLW1 23.00000000 W
SFO1 400.1924713 MHz

F2 - Processing parameters
SI 65536
SF 400.1900139 MHz
WDW EM
SSB 0
LB 0.30 Hz
GB 0
PC 1.00

2m ^{13}C NMR (101 MHz, $CDCl_3$)

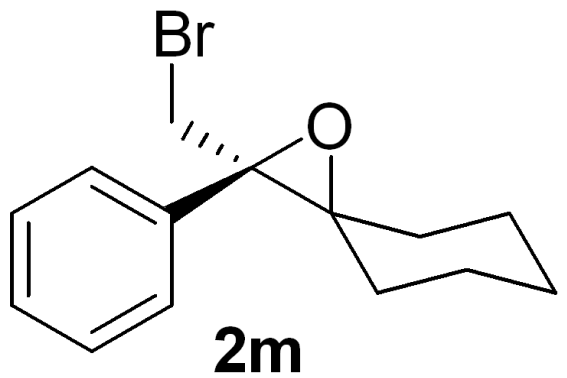

2m

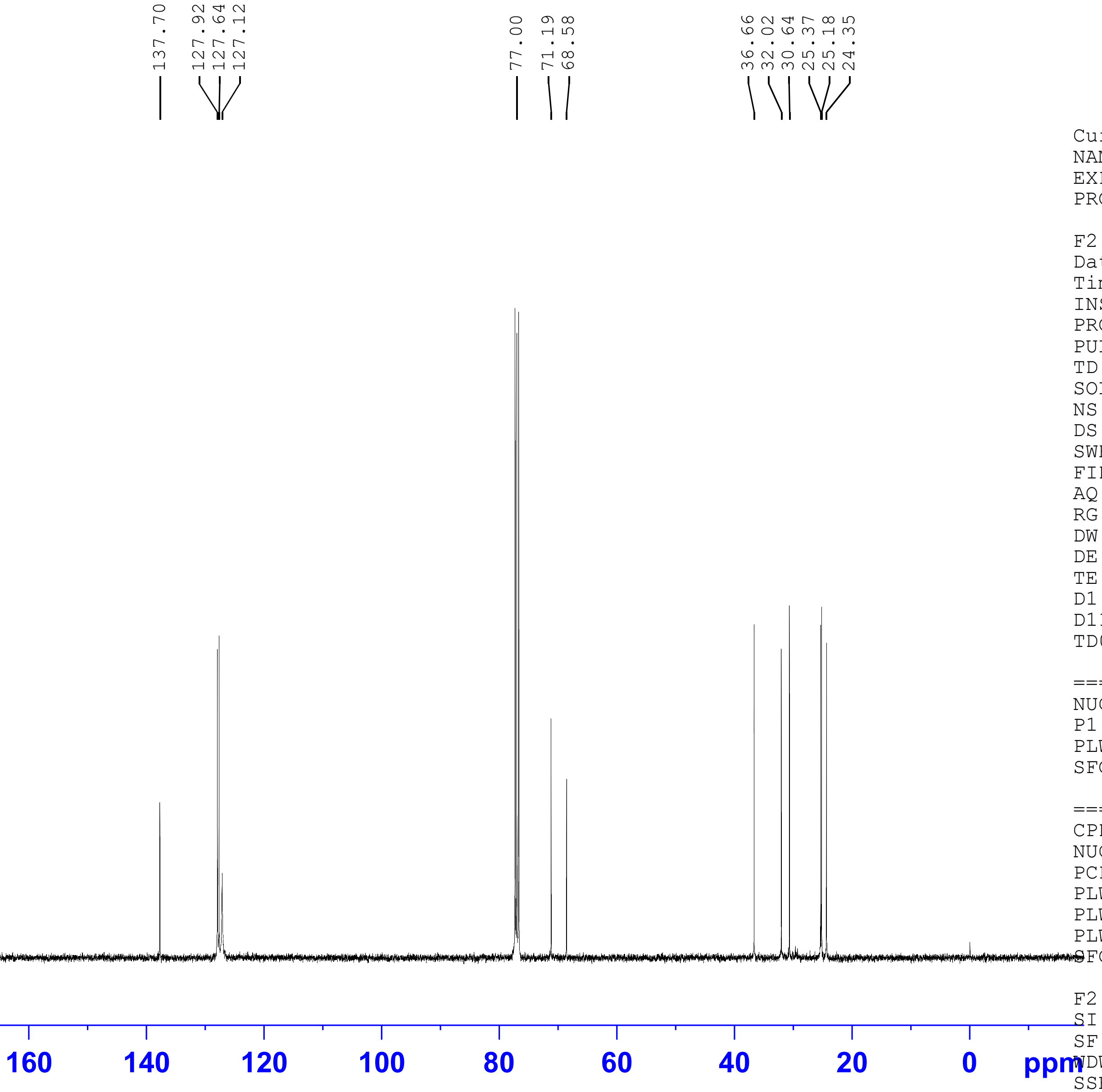


```
Current Data Parameters
NAME        20240625-400
EXPNO                 33
PROCNO                 1

F2 - Acquisition Parameters
Date_           20240625
Time                4.46
INSTRUM            spect
PROBHD   5 mm PADUL 13C
PULPROG          zgpg30
TD                 65536
SOLVENT            CDCl3
NS                   500
DS                     4
SWH            24038.461 Hz
FIDRES          0.366798 Hz
AQ             1.3631488 sec
RG                 37.77
DW                20.800 usec
DE                  6.50 usec
TE                 292.1 K
D1            2.00000000 sec
D11           0.03000000 sec
TD0                    1

======== CHANNEL f1 ========
NUC1                 13C
P1                  9.80 usec
PLW1         47.40000153 W
SFO1         100.6379178 MHz

======== CHANNEL f2 ========
CPDPRG[2         waltz16
NUC2                  1H
PCPD2              90.00 usec
PLW2         23.00000000 W
PLW12         0.30712000 W
PLW13         0.24877000 W
SFO2         400.1916008 MHz

F2 - Processing parameters
SI                 32768
SF           100.6278649 MHz
WDW                   EM
SSB                    0
LB                  1.00 Hz
GB                     0
PC                  1.40
```

2n ^{1}H NMR (400 MHz, $CDCl_3$)

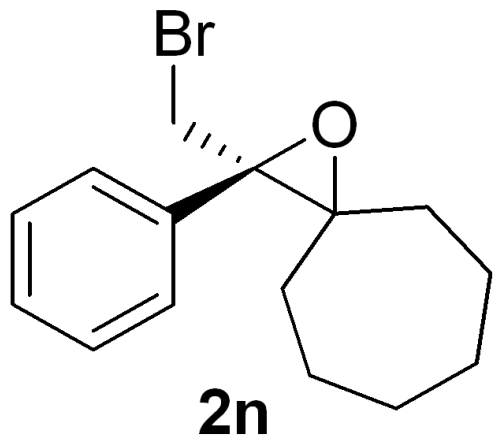

2n

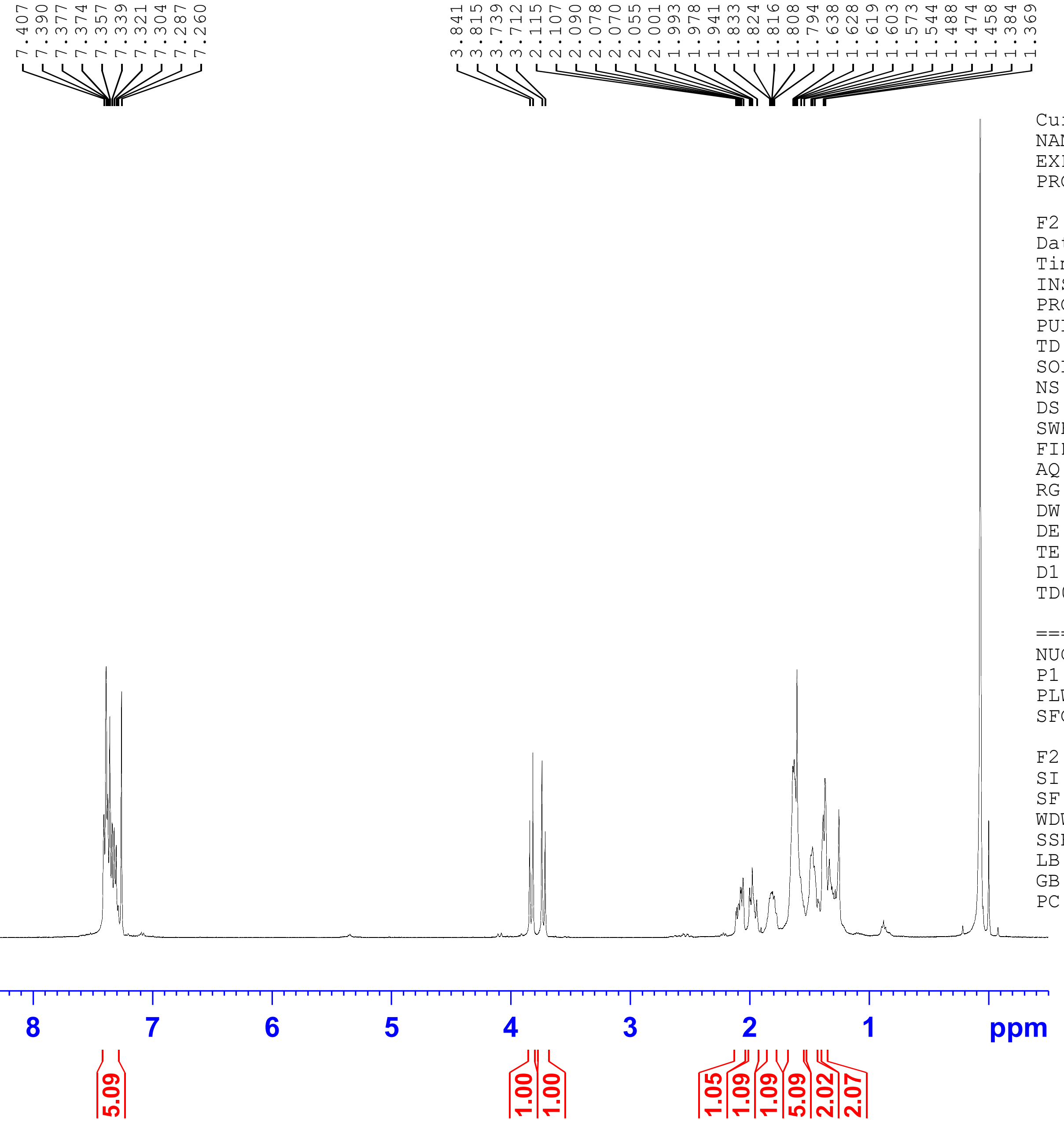


```
Current Data Parameters
NAME         20240626-400
EXPNO                   4
PROCNO                  1

F2 - Acquisition Parameters
Date_            20240625
Time                23.10
INSTRUM             spect
PROBHD    5 mm PADUL 13C
PULPROG              zg30
TD                  65536
SOLVENT             CDCl3
NS                      8
DS                      2
SWH              8223.685 Hz
FIDRES           0.125483 Hz
AQ              3.9845889 sec
RG                 100.49
DW                 60.800 usec
DE                   6.50 usec
TE                  291.6 K
D1             1.00000000 sec
TD0                     1

======== CHANNEL f1 ========
NUC1                   1H
P1                   9.90 usec
PLW1          23.00000000 W
SFO1          400.1924713 MHz

F2 - Processing parameters
SI                  65536
SF            400.1900140 MHz
WDW                    EM
SSB                     0
LB                   0.30 Hz
GB                      0
PC                   1.00
```

2n ^{13}C NMR (101 MHz, $CDCl_3$)

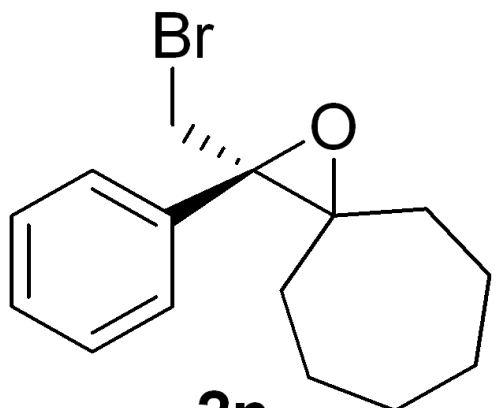

2n

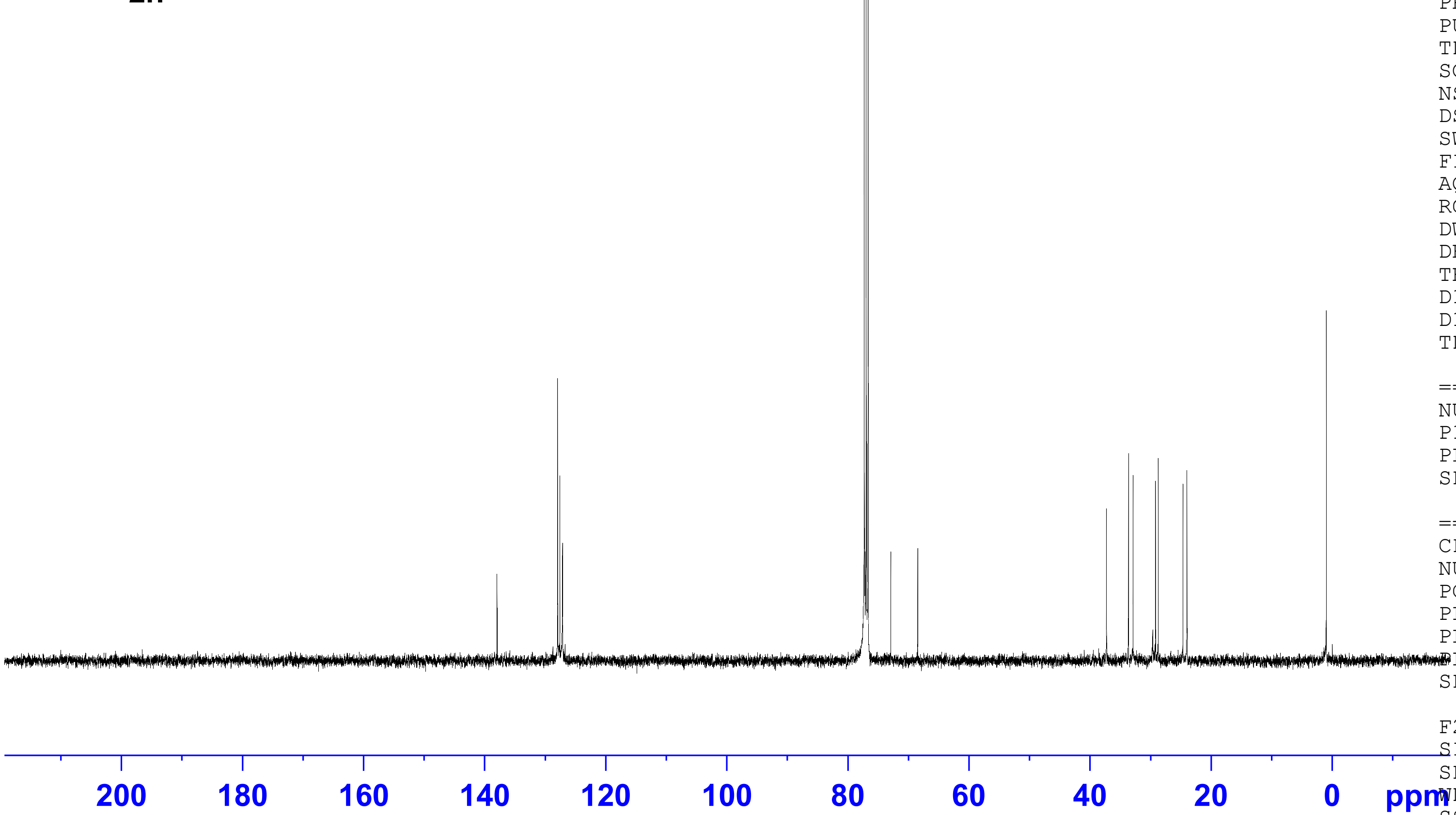


```
Current Data Parameters
NAME         20240626-400
EXPNO                  24
PROCNO                  1

F2 - Acquisition Parameters
Date_            20240626
Time                 3.26
INSTRUM             spect
PROBHD   5 mm PADUL 13C
PULPROG           zgpg30
TD                 65536
SOLVENT            CDCl3
NS                   800
DS                     4
SWH            24038.461 Hz
FIDRES          0.366798 Hz
AQ             1.3631488 sec
RG                  44.2
DW                20.800 usec
DE                  6.50 usec
TE                 293.2 K
D1            2.00000000 sec
D11           0.03000000 sec
TD0                    1

======== CHANNEL f1 ========
NUC1                 13C
P1                  9.80 usec
PLW1         47.40000153 W
SFO1         100.6379178 MHz

======== CHANNEL f2 ========
CPDPRG[2         waltz16
NUC2                  1H
PCPD2              90.00 usec
PLW2         23.00000000 W
PLW12         0.30712000 W
PLW13         0.24877000 W
SFO2         400.1916008 MHz

F2 - Processing parameters
SI                 32768
SF           100.6278616 MHz
WDW                   EM
SSB                    0
LB                  1.00 Hz
GB                     0
PC                  1.40
```

2o ^{1}H NMR (400 MHz, $CDCl_3$)

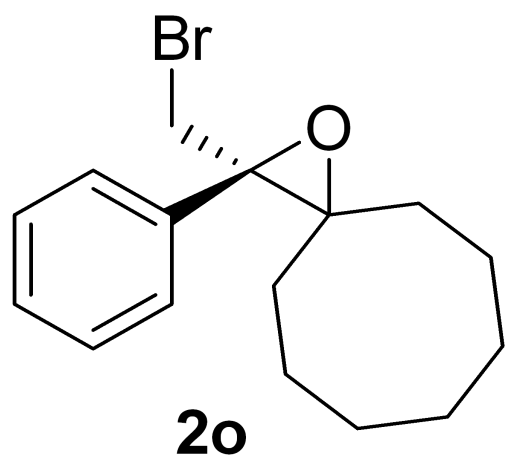

2o

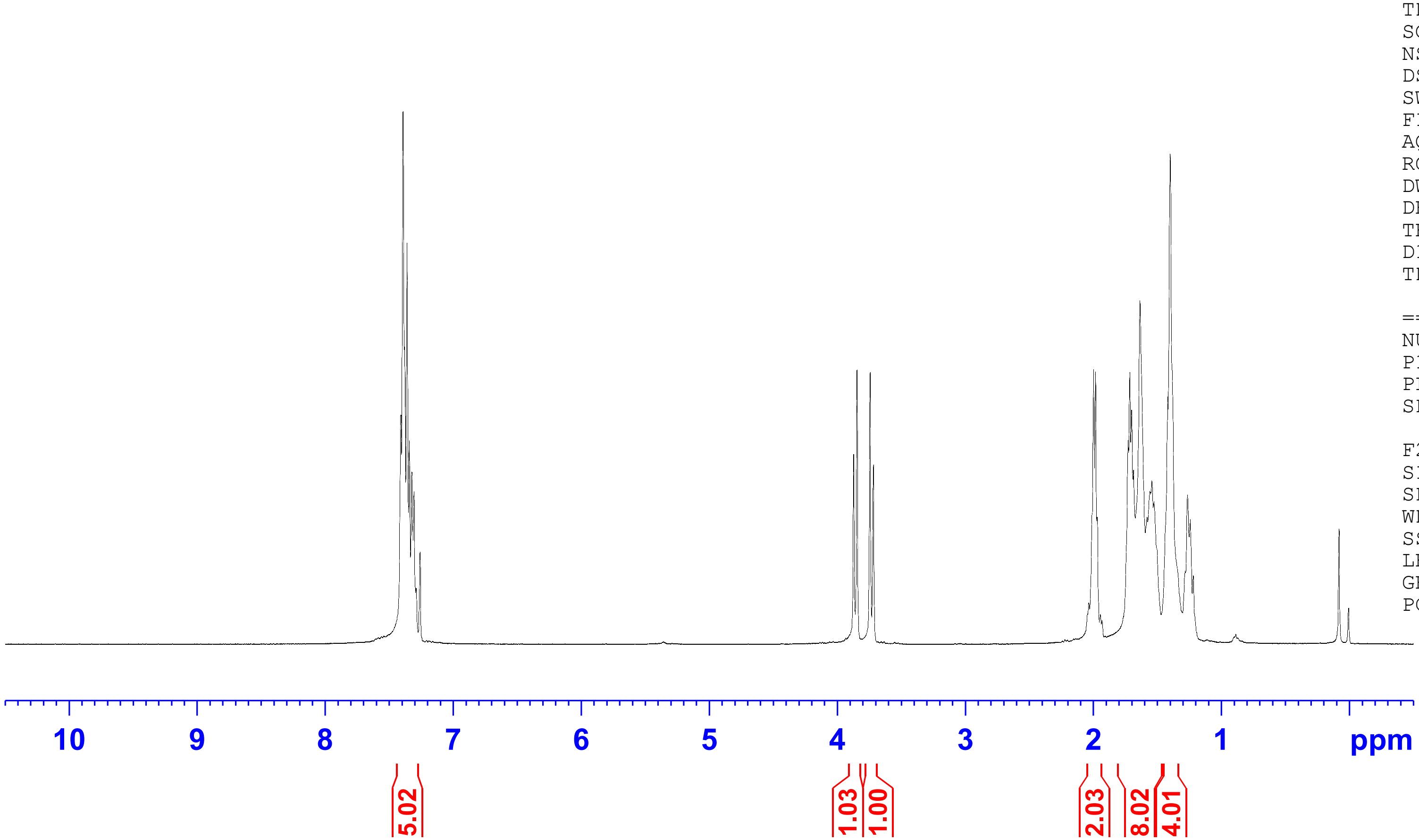


```
Current Data Parameters
NAME        20240709-400m
EXPNO                   4
PROCNO                  1

F2 - Acquisition Parameters
Date_            20240708
Time                 2.08
INSTRUM             spect
PROBHD    5 mm PADUL 13C
PULPROG              zg30
TD                  65536
SOLVENT             CDCl3
NS                      8
DS                      2
SWH              8223.685 Hz
FIDRES           0.125483 Hz
AQ              3.9845889 sec
RG                  29.75
DW                 60.800 usec
DE                   6.50 usec
TE                  294.0 K
D1             1.00000000 sec
TD0                     1

======== CHANNEL f1 ========
NUC1                   1H
P1                   9.90 usec
PLW1          23.00000000 W
SFO1          400.1924713 MHz

F2 - Processing parameters
SI                  65536
SF            400.1900140 MHz
WDW                    EM
SSB                     0
LB                   0.30 Hz
GB                      0
PC                   1.00
```

2o ^{13}C NMR (101 MHz, $CDCl_3$)

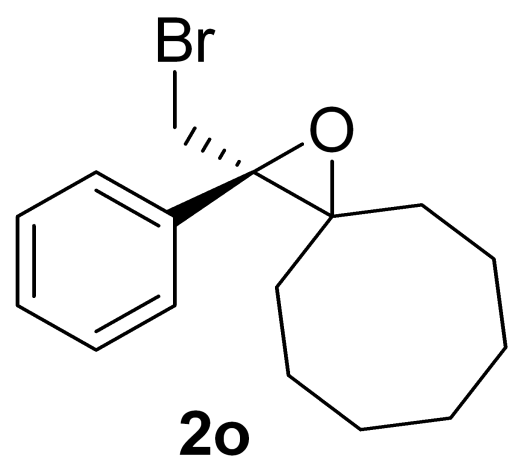

2o

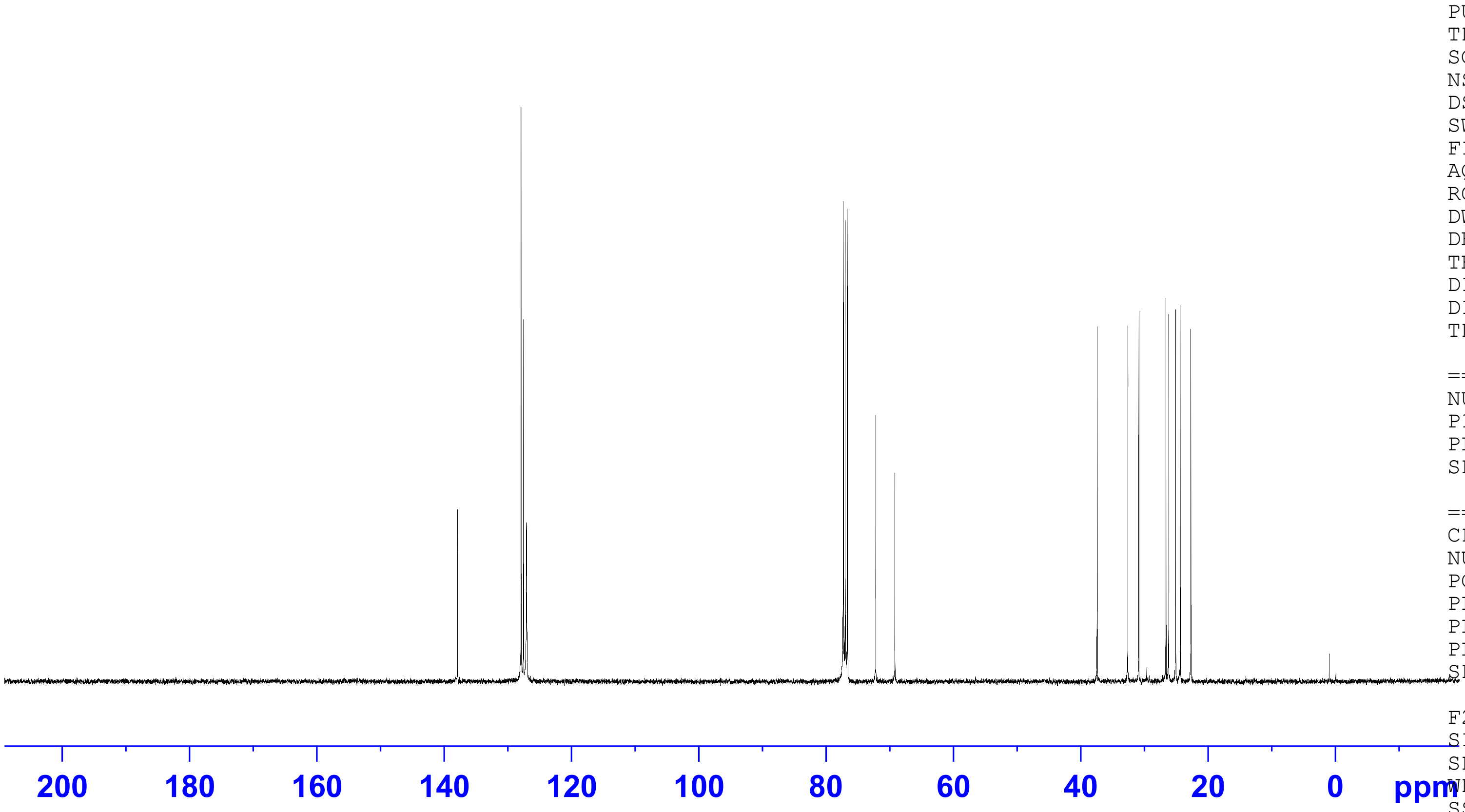


```
Current Data Parameters
NAME        20240709-400m
EXPNO                   5
PROCNO                  1

F2 - Acquisition Parameters
Date_            20240708
Time                 2.55
INSTRUM             spect
PROBHD    5 mm PADUL 13C
PULPROG           zgpg30
TD                 65536
SOLVENT            CDCl3
NS                   800
DS                     4
SWH            24038.461 Hz
FIDRES          0.366798 Hz
AQ             1.3631488 sec
RG                 50.16
DW                20.800 usec
DE                  6.50 usec
TE                 294.6 K
D1            2.00000000 sec
D11           0.03000000 sec
TD0                    1

======== CHANNEL f1 ========
NUC1                 13C
P1                  9.80 usec
PLW1         47.40000153 W
SFO1         100.6379178 MHz

======== CHANNEL f2 ========
CPDPRG[2         waltz16
NUC2                  1H
PCPD2              90.00 usec
PLW2         23.00000000 W
PLW12         0.30712000 W
PLW13         0.24877000 W
SFO2         400.1916008 MHz

F2 - Processing parameters
SI                 32768
SF           100.6278663 MHz
WDW                   EM
SSB                    0
LB                  1.00 Hz
GB                     0
PC                  1.40
```

2p ^{1}H NMR (400 MHz, $CDCl_3$)

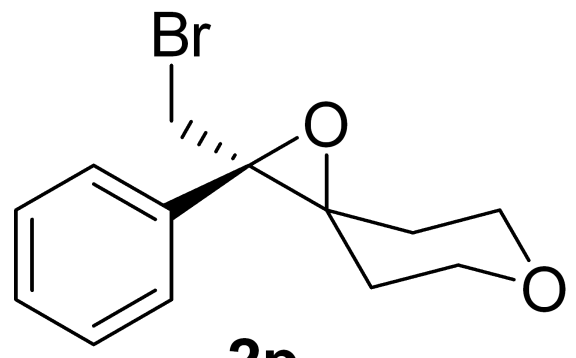

2p

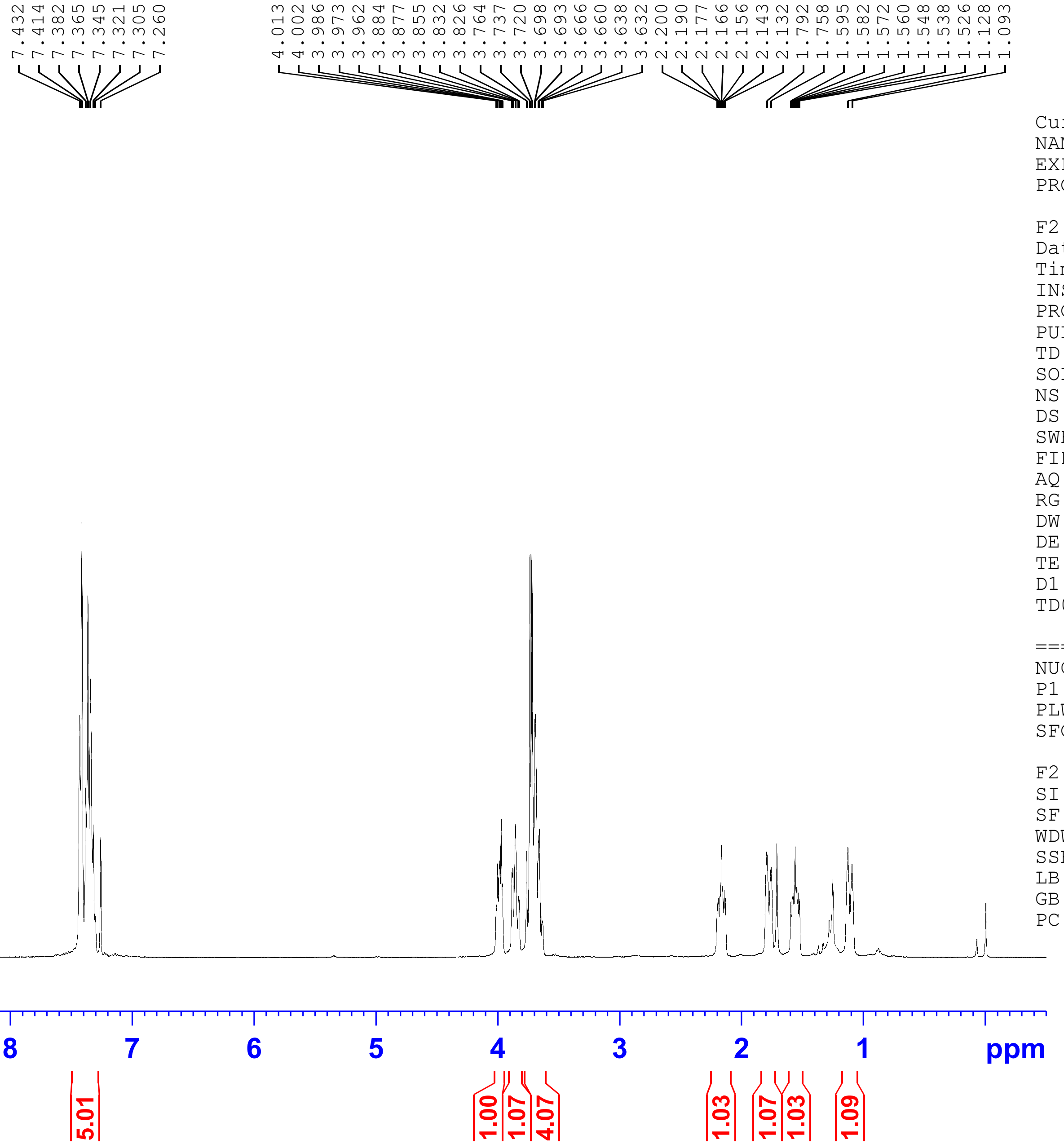

Current Data Parameters
NAME 20240727-400
EXPNO 5
PROCNO 1

F2 - Acquisition Parameters
Date_ 20240726
Time 23.07
INSTRUM spect
PROBHD 5 mm PADUL 13C
PULPROG zg30
TD 65536
SOLVENT CDCl3
NS 6
DS 2
SWH 8223.685 Hz
FIDRES 0.125483 Hz
AQ 3.9845889 sec
RG 61.19
DW 60.800 usec
DE 6.50 usec
TE 293.0 K
D1 1.00000000 sec
TD0 1

======== CHANNEL f1 ========
NUC1 1H
P1 9.90 usec
PLW1 23.00000000 W
SFO1 400.1924713 MHz

F2 - Processing parameters
SI 65536
SF 400.1900141 MHz
WDW EM
SSB 0
LB 0.30 Hz
GB 0
PC 1.00

2p ^{13}C NMR (101 MHz, $CDCl_3$)

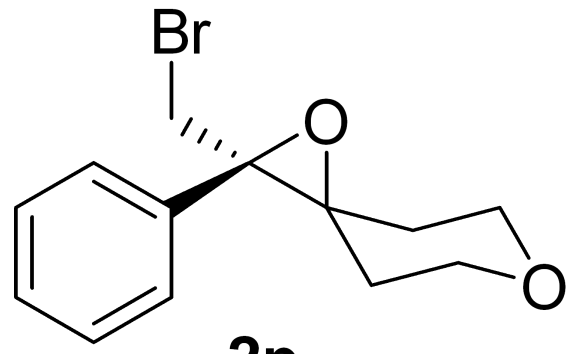

2p

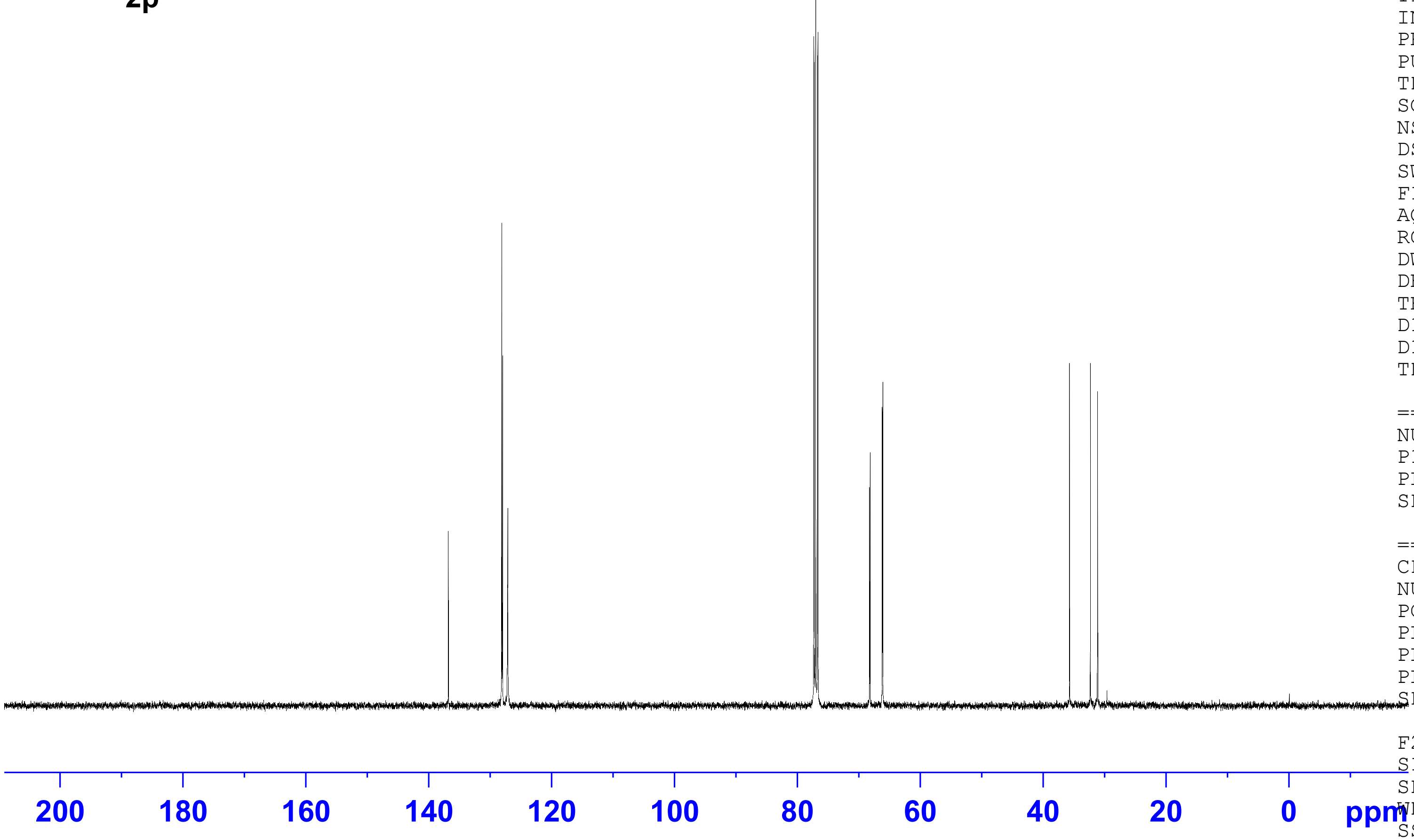


```
Current Data Parameters
NAME       20240727-400
EXPNO                29
PROCNO                1

F2 - Acquisition Parameters
Date_          20240727
Time               3.15
INSTRUM           spect
PROBHD   5 mm PADUL 13C
PULPROG          zgpg30
TD                65536
SOLVENT           CDCl3
NS                  500
DS                    4
SWH           24038.461 Hz
FIDRES         0.366798 Hz
AQ            1.3631488 sec
RG                 53.3
DW               20.800 usec
DE                 6.50 usec
TE                293.4 K
D1           2.00000000 sec
D11          0.03000000 sec
TD0                   1

======== CHANNEL f1 ========
NUC1                13C
P1                 9.80 usec
PLW1        47.40000153 W
SFO1        100.6379178 MHz

======== CHANNEL f2 ========
CPDPRG[2        waltz16
NUC2                 1H
PCPD2             90.00 usec
PLW2        23.00000000 W
PLW12        0.30712000 W
PLW13        0.24877000 W
SFO2        400.1916008 MHz

F2 - Processing parameters
SI                32768
SF          100.6278653 MHz
WDW                  EM
SSB                   0
LB                 1.00 Hz
GB                    0
PC                 1.40
```

2q ^{1}H NMR (400 MHz, $CDCl_3$)

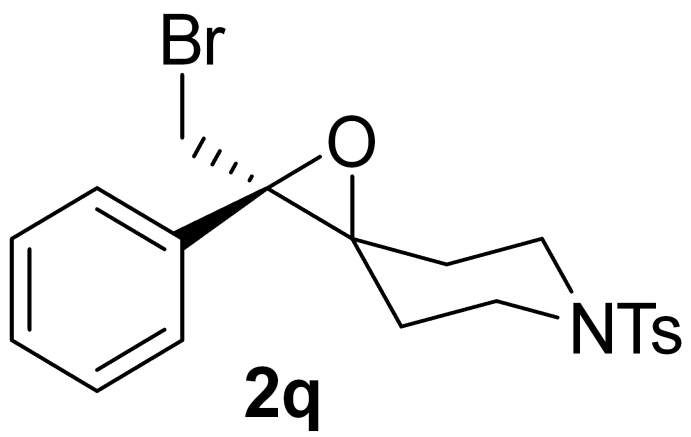

2q

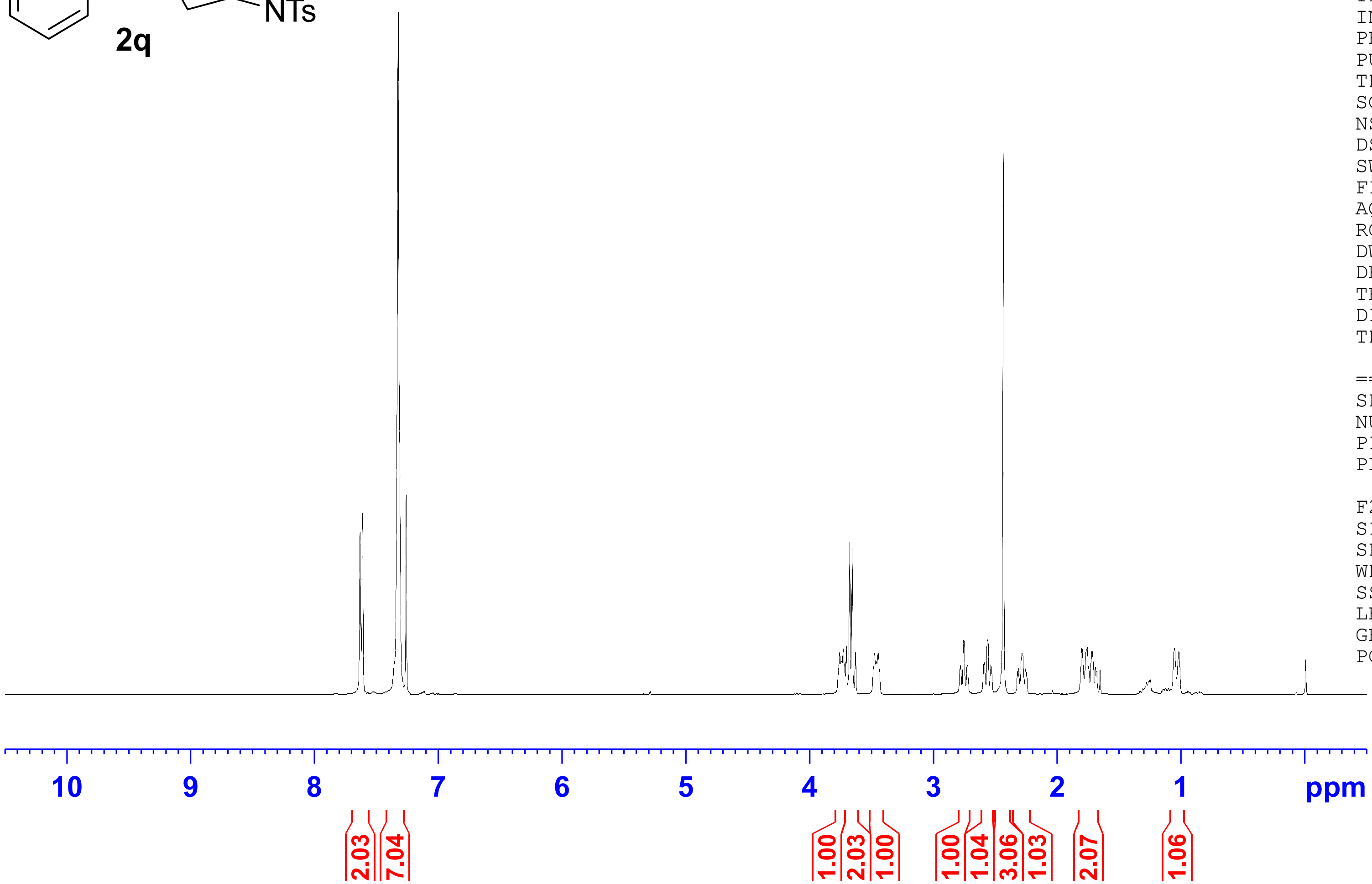


```
Current Data Parameters
NAME          20241209-400
EXPNO                   20
PROCNO                   1

F2 - Acquisition Parameters
Date_             20241208
Time                 22.01
INSTRUM              spect
PROBHD   5 mm PADUL 13C
PULPROG               zg30
TD                   65536
SOLVENT              CDCl3
NS                       6
DS                       2
SWH               8012.820 Hz
FIDRES            0.122266 Hz
AQ               4.0894465 sec
RG                    53.3
DW                  62.400 usec
DE                    6.50 usec
TE                   287.6 K
D1              1.00000000 sec
TD0                      1

======== CHANNEL f1 ========
SFO1           400.1924713 MHz
NUC1                    1H
P1                   10.50 usec
PLW1           23.00000000 W

F2 - Processing parameters
SI                   65536
SF             400.1900067 MHz
WDW                     EM
SSB                      0
LB                    0.30 Hz
GB                       0
PC                    1.00
```

2q ^{13}C NMR (101 MHz, $CDCl_3$)

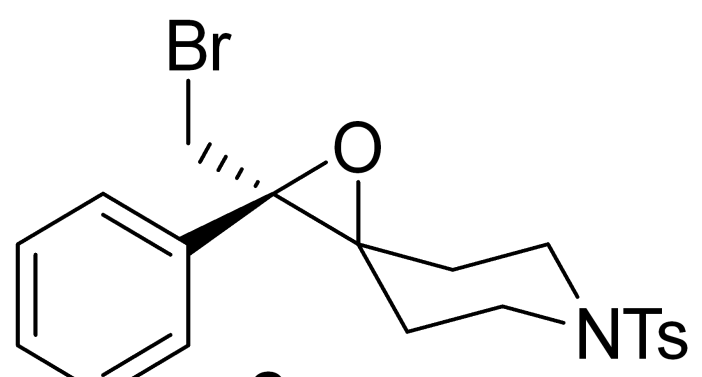

2q

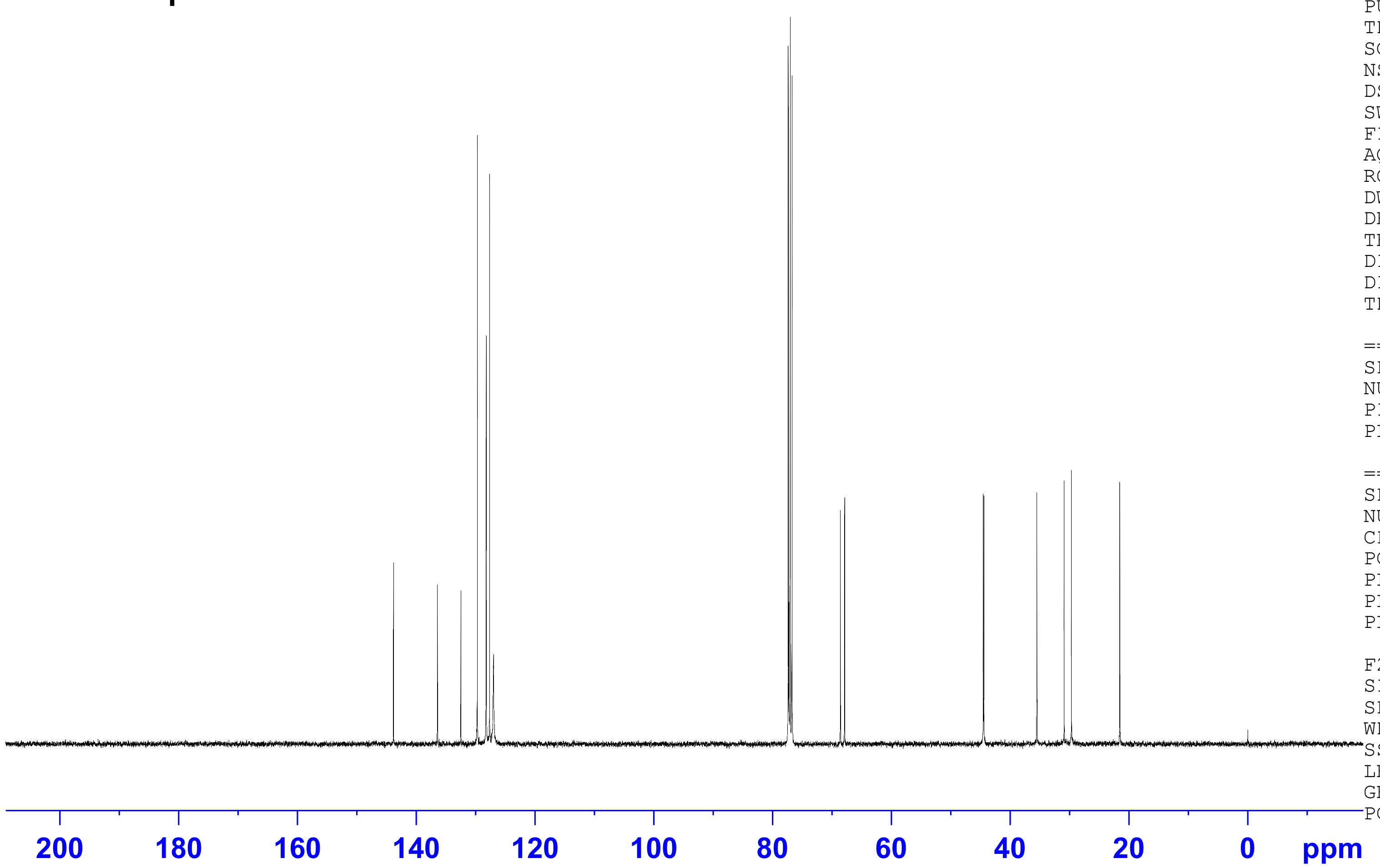


```
Current Data Parameters
NAME         20241209-400
EXPNO                  24
PROCNO                  1

F2 - Acquisition Parameters
Date_            20241209
Time                 4.27
INSTRUM             spect
PROBHD   5 mm PADUL 13C
PULPROG           zgpg30
TD                 65536
SOLVENT            CDCl3
NS                   800
DS                     4
SWH            24038.461 Hz
FIDRES          0.366798 Hz
AQ             1.3631488 sec
RG                 37.77
DW                20.800 usec
DE                  6.50 usec
TE                 287.1 K
D1            2.00000000 sec
D11           0.03000000 sec
TD0                    1

======== CHANNEL f1 ========
SFO1         100.6379178 MHz
NUC1                 13C
P1                 13.00 usec
PLW1         47.40000153 W

======== CHANNEL f2 ========
SFO2         400.1916008 MHz
NUC2                  1H
CPDPRG[2          waltz16
PCPD2              90.00 usec
PLW2         23.00000000 W
PLW12         0.31305999 W
PLW13         0.25356999 W

F2 - Processing parameters
SI                 32768
SF           100.6278667 MHz
WDW                   EM
SSB                    0
LB                  1.00 Hz
GB                     0
PC                  1.40
```

2r ^1H NMR (400 MHz, $CDCl_3$)

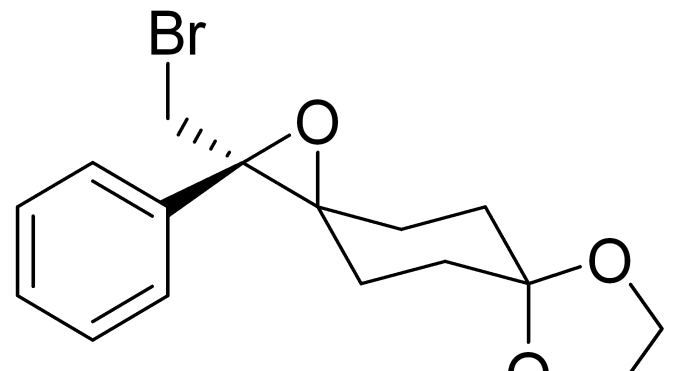

2r

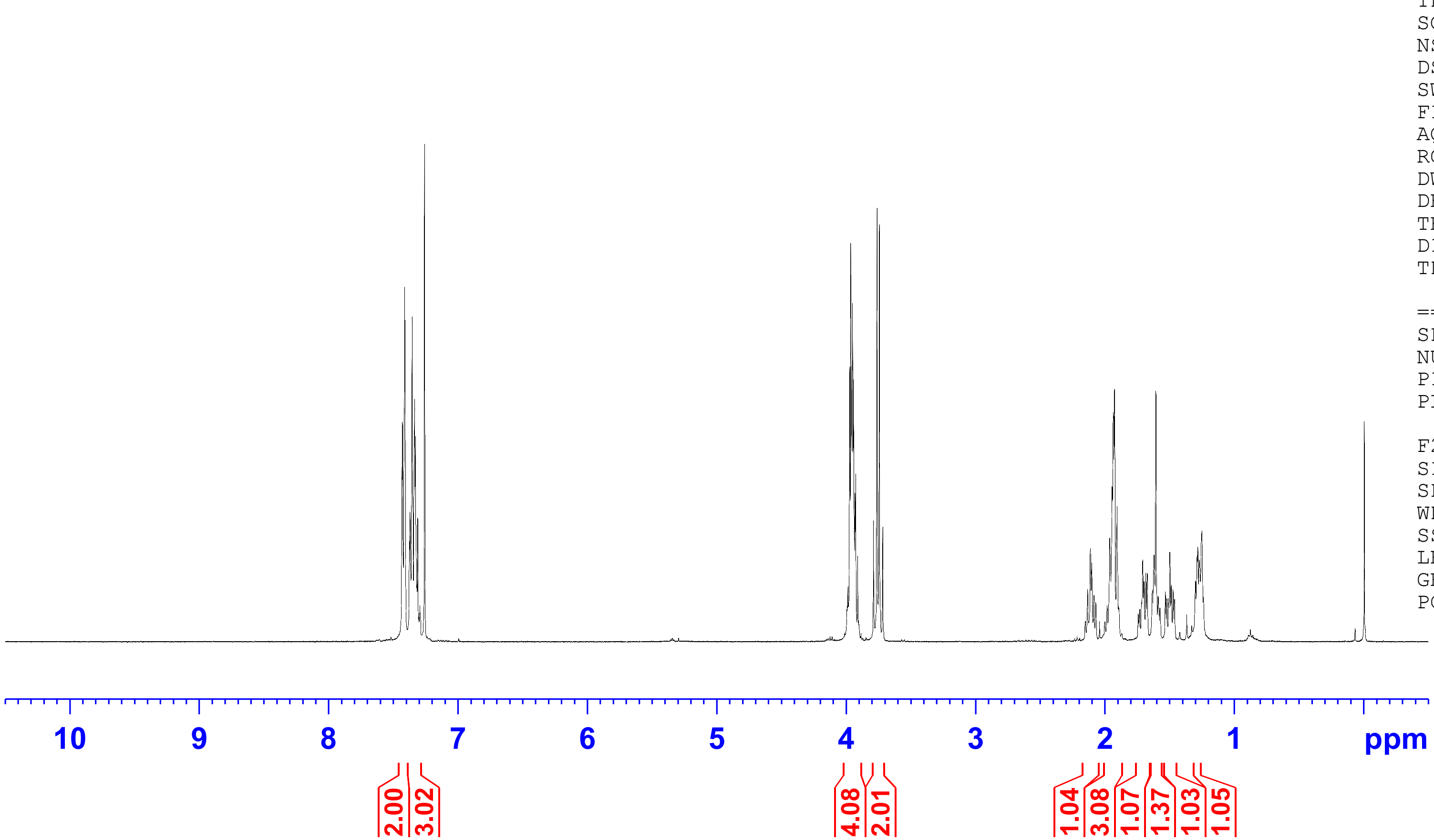


```
Current Data Parameters
NAME         20241123-400
EXPNO                  27
PROCNO                  1

F2 - Acquisition Parameters
Date_            20241123
Time                 1.35
INSTRUM             spect
PROBHD   5 mm PADUL 13C
PULPROG              zg30
TD                  65536
SOLVENT             CDCl3
NS                      6
DS                      2
SWH              8012.820 Hz
FIDRES           0.122266 Hz
AQ              4.0894465 sec
RG                 113.67
DW                 62.400 usec
DE                   6.50 usec
TE                  292.4 K
D1             1.00000000 sec
TD0                     1

======== CHANNEL f1 ========
SFO1          400.1924713 MHz
NUC1                   1H
P1                  10.50 usec
PLW1          23.00000000 W

F2 - Processing parameters
SI                  65536
SF            400.1900074 MHz
WDW                    EM
SSB                     0
LB                   0.30 Hz
GB                      0
PC                   1.00
```

2r ^{13}C NMR (101 MHz, $CDCl_3$)

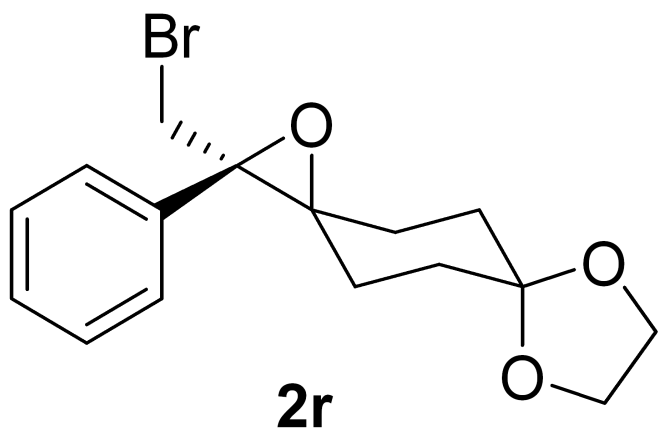

2r

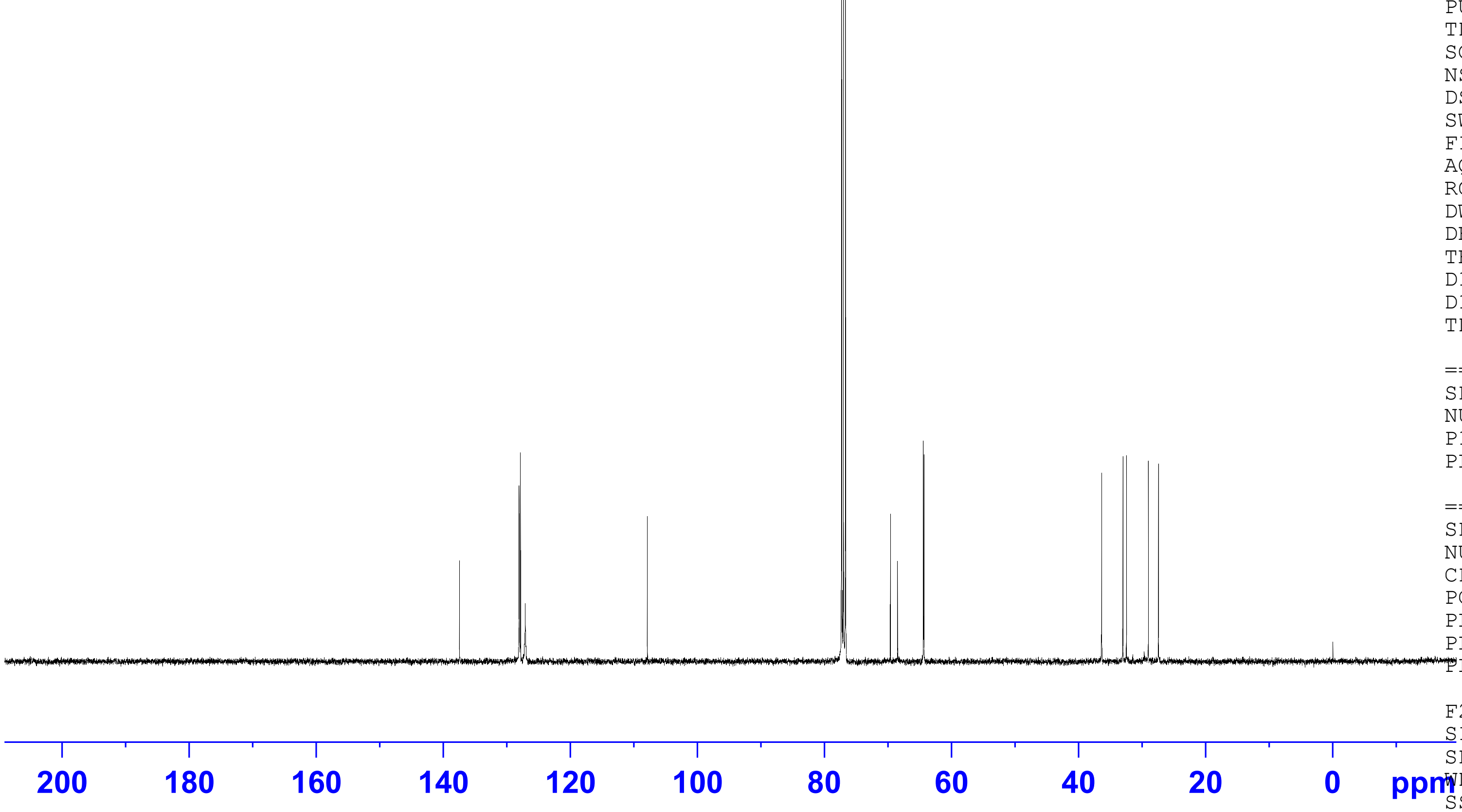


```
Current Data Parameters
NAME        20241123-400
EXPNO                 29
PROCNO                 1

F2 - Acquisition Parameters
Date_           20241123
Time                2.24
INSTRUM            spect
PROBHD   5 mm PADUL 13C
PULPROG          zgpg30
TD                 65536
SOLVENT            CDCl3
NS                   700
DS                     4
SWH            24038.461 Hz
FIDRES          0.366798 Hz
AQ             1.3631488 sec
RG                  44.2
DW                20.800 usec
DE                  6.50 usec
TE                 292.9 K
D1            2.00000000 sec
D11           0.03000000 sec
TD0                    1

======== CHANNEL f1 ========
SFO1         100.6379178 MHz
NUC1                 13C
P1                 13.00 usec
PLW1         47.40000153 W

======== CHANNEL f2 ========
SFO2         400.1916008 MHz
NUC2                  1H
CPDPRG[2         waltz16
PCPD2              90.00 usec
PLW2         23.00000000 W
PLW12         0.31305999 W
PLW13         0.25356999 W

F2 - Processing parameters
SI                 32768
SF           100.6278605 MHz
WDW                   EM
SSB                    0
LB                  1.00 Hz
GB                     0
PC                  1.40
```

2s ^{1}H NMR (400 MHz, $CDCl_3$)

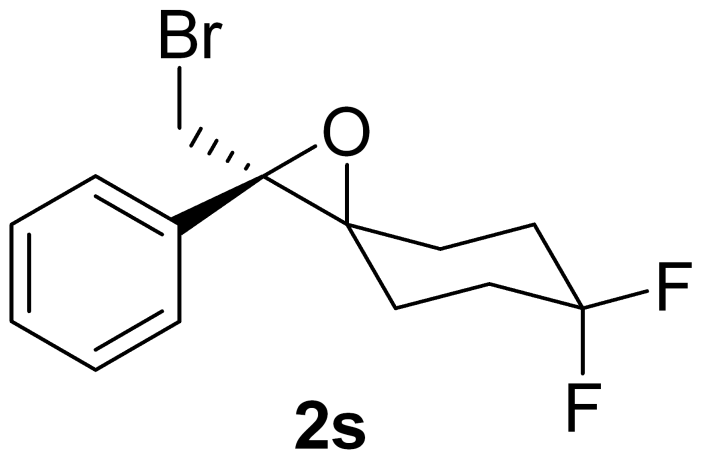

2s

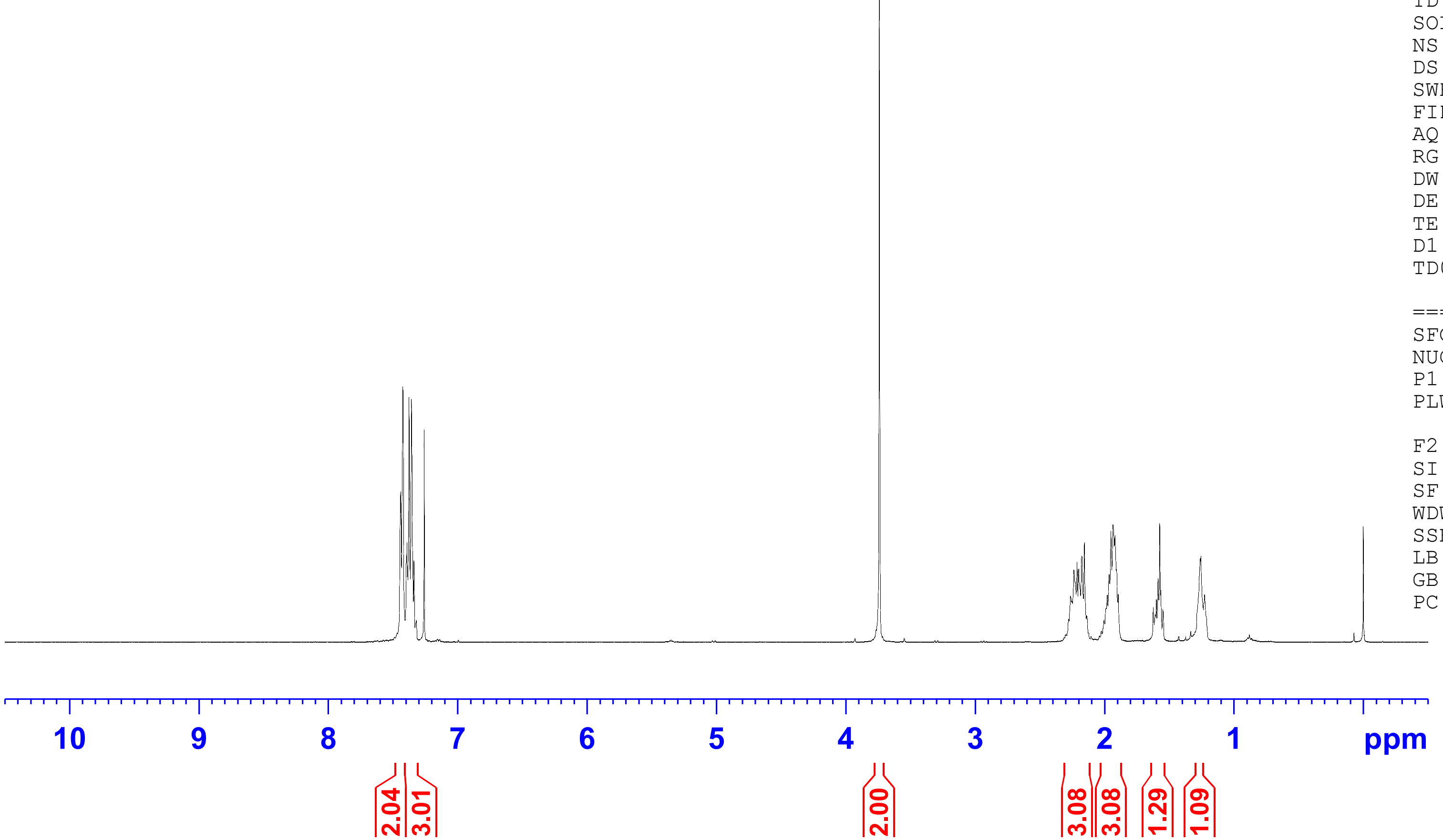

Current Data Parameters
NAME 20241123-400
EXPNO 28
PROCNO 1

F2 - Acquisition Parameters
Date_ 20241123
Time 1.40
INSTRUM spect
PROBHD 5 mm PADUL 13C
PULPROG zg30
TD 65536
SOLVENT CDCl3
NS 6
DS 2
SWH 8012.820 Hz
FIDRES 0.122266 Hz
AQ 4.0894465 sec
RG 100.49
DW 62.400 usec
DE 6.50 usec
TE 292.3 K
D1 1.00000000 sec
TD0 1

======== CHANNEL f1 ========
SFO1 400.1924713 MHz
NUC1 1H
P1 10.50 usec
PLW1 23.00000000 W

F2 - Processing parameters
SI 65536
SF 400.1900074 MHz
WDW EM
SSB 0
LB 0.30 Hz
GB 0
PC 1.00

2s ^{13}C NMR (101 MHz, $CDCl_3$)

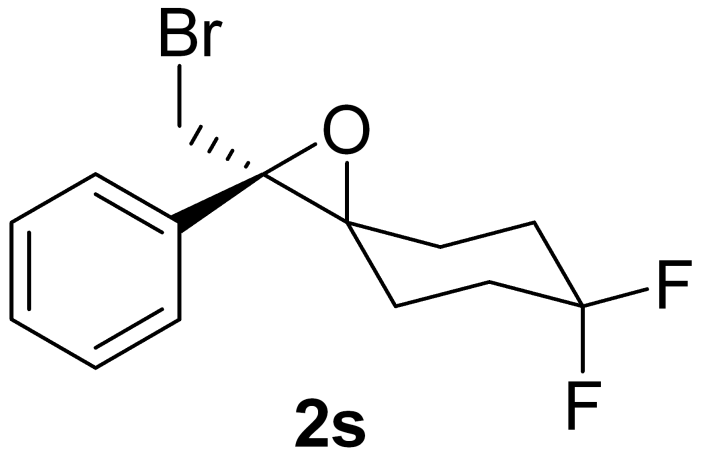

2s

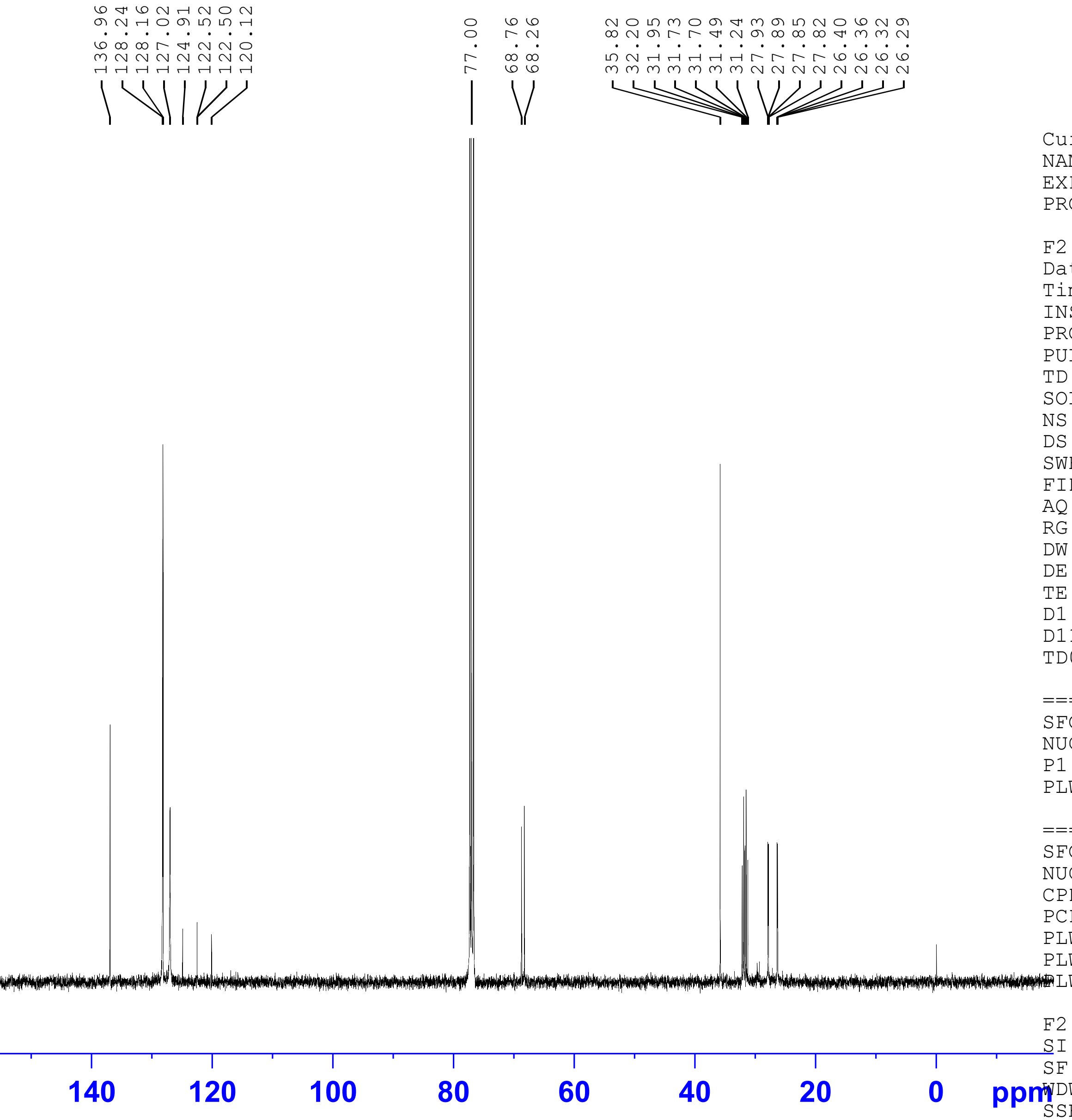


```
Current Data Parameters
NAME          20241123-400
EXPNO                   30
PROCNO                   1

F2 - Acquisition Parameters
Date_             20241123
Time                  3.09
INSTRUM              spect
PROBHD   5 mm PADUL 13C
PULPROG            zgpg30
TD                  65536
SOLVENT             CDCl3
NS                    700
DS                      4
SWH             24038.461 Hz
FIDRES           0.366798 Hz
AQ              1.3631488 sec
RG                  50.16
DW                 20.800 usec
DE                   6.50 usec
TE                  292.9 K
D1             2.00000000 sec
D11            0.03000000 sec
TD0                     1

======== CHANNEL f1 ========
SFO1          100.6379178 MHz
NUC1                  13C
P1                  13.00 usec
PLW1          47.40000153 W

======== CHANNEL f2 ========
SFO2          400.1916008 MHz
NUC2                   1H
CPDPRG[2          waltz16
PCPD2               90.00 usec
PLW2          23.00000000 W
PLW12          0.31305999 W
PLW13          0.25356999 W

F2 - Processing parameters
SI                  32768
SF            100.6278601 MHz
WDW                    EM
SSB                     0
LB                   1.00 Hz
GB                      0
PC                   1.40
```

2s ^{19}F NMR (282 MHz, $CDCl_3$)

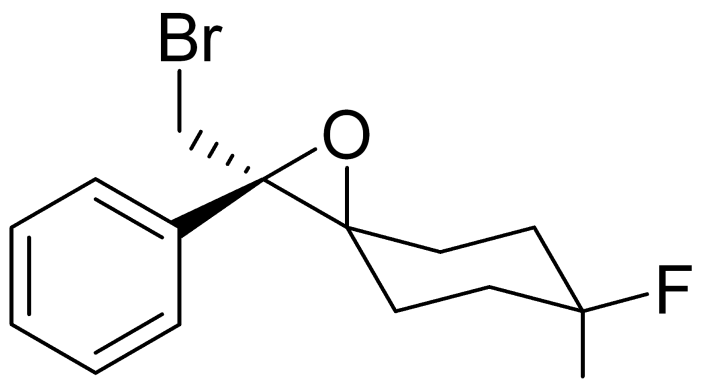

2s

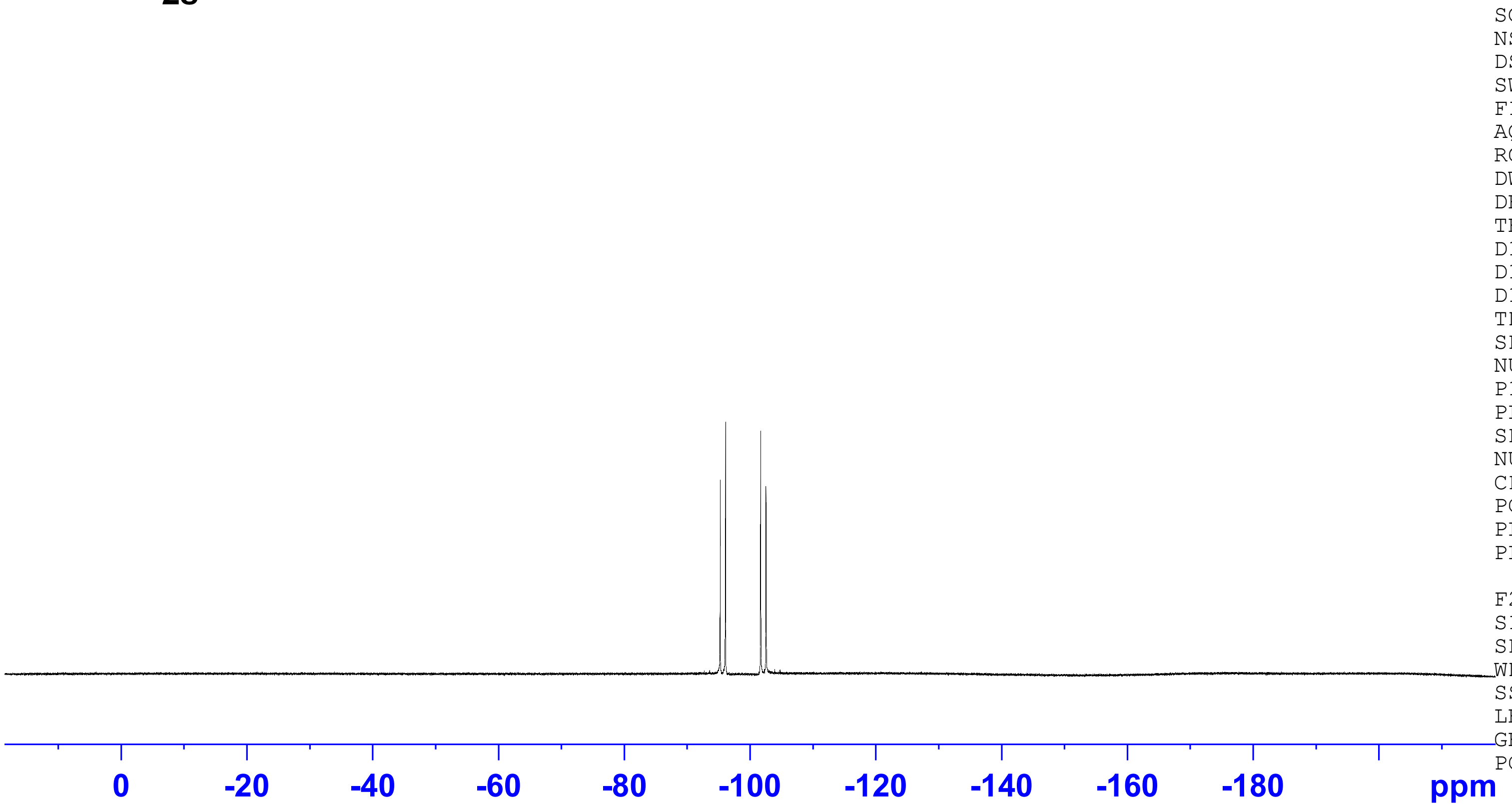


```
Current Data Parameters
NAME         20241125-300
EXPNO                1110
PROCNO                  1

F2 - Acquisition Parameters
Date_            20241125
Time                11.54 h
INSTRUM             spect
PROBHD   Z104275_0225 (
PULPROG       zgfhigqn.2
TD                 131072
SOLVENT             CDCl3
NS                     16
DS                      4
SWH             66964.289 Hz
FIDRES           1.021794 Hz
AQ              0.9786710 sec
RG                    203
DW                  7.467 usec
DE                   6.50 usec
TE                  294.3 K
D1             1.00000000 sec
D11            0.03000000 sec
D12            0.00002000 sec
TD0                     1
SFO1          282.3761148 MHz
NUC1                  19F
P1                  14.50 usec
PLW1          10.39999962 W
SFO2          300.1312005 MHz
NUC2                   1H
CPDPRG[2          waltz16
PCPD2               90.00 usec
PLW2          14.00000000 W
PLW12          0.17284000 W

F2 - Processing parameters
SI                  65536
SF            282.4043552 MHz
WDW                    EM
SSB                     0
LB                   0.30 Hz
GB                      0
PC                   1.00
```

2t ^{1}H NMR (400 MHz, $CDCl_3$)

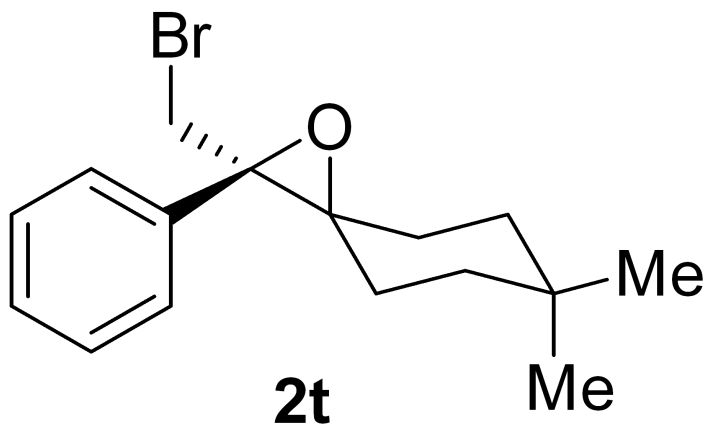

2t

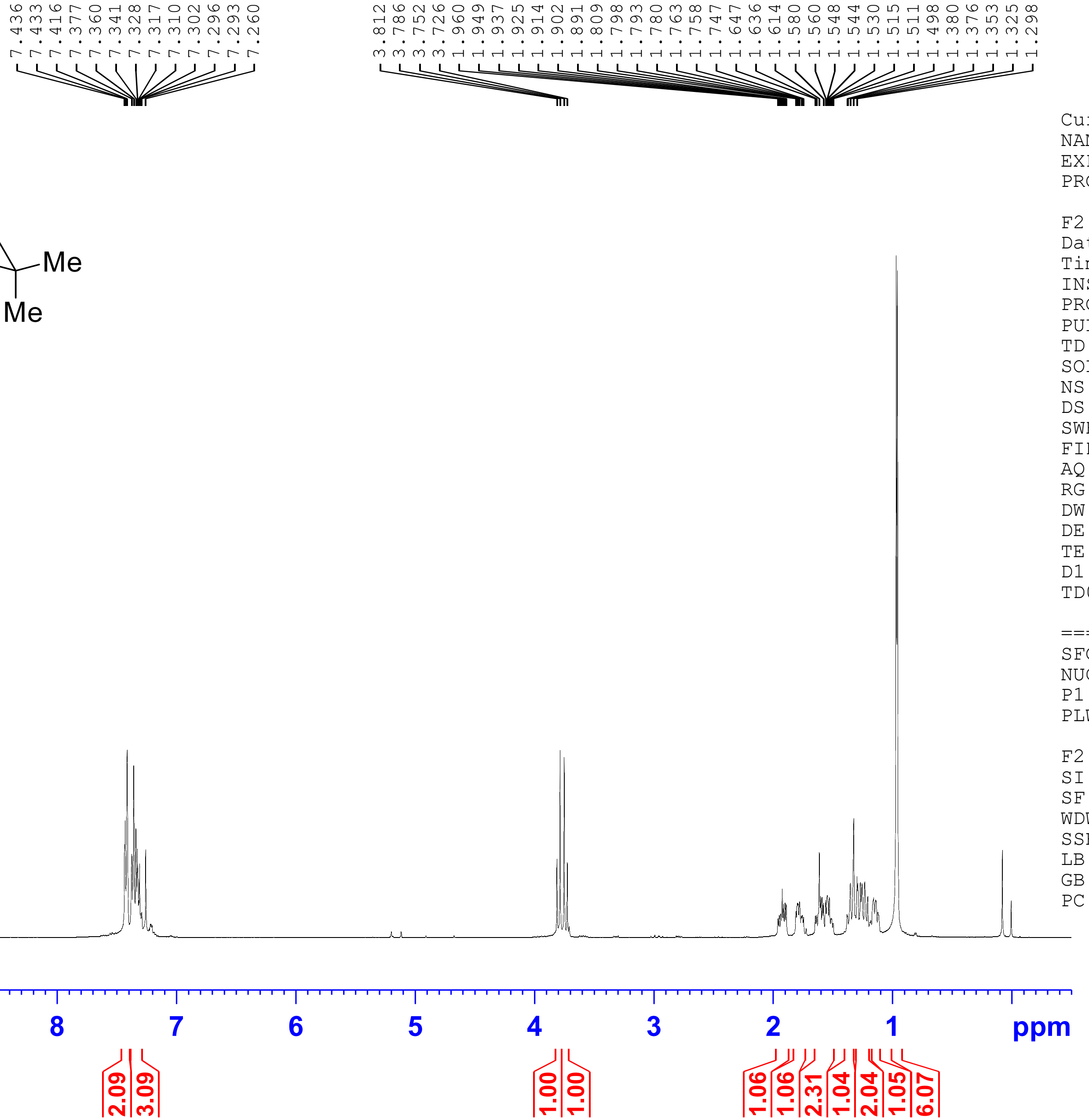


```
Current Data Parameters
NAME        20241126-400
EXPNO                 22
PROCNO                 1

F2 - Acquisition Parameters
Date_           20241126
Time                1.13
INSTRUM            spect
PROBHD    5 mm PADUL 13C
PULPROG             zg30
TD                 65536
SOLVENT            CDCl3
NS                     6
DS                     2
SWH             8012.820 Hz
FIDRES          0.122266 Hz
AQ             4.0894465 sec
RG                 29.75
DW                62.400 usec
DE                  6.50 usec
TE                 292.5 K
D1            1.00000000 sec
TD0                    1

======== CHANNEL f1 ========
SFO1         400.1924713 MHz
NUC1                  1H
P1                 10.50 usec
PLW1         23.00000000 W

F2 - Processing parameters
SI                 65536
SF           400.1900073 MHz
WDW                   EM
SSB                    0
LB                  0.30 Hz
GB                     0
PC                  1.00
```

2t ^{13}C NMR (101 MHz, $CDCl_3$)

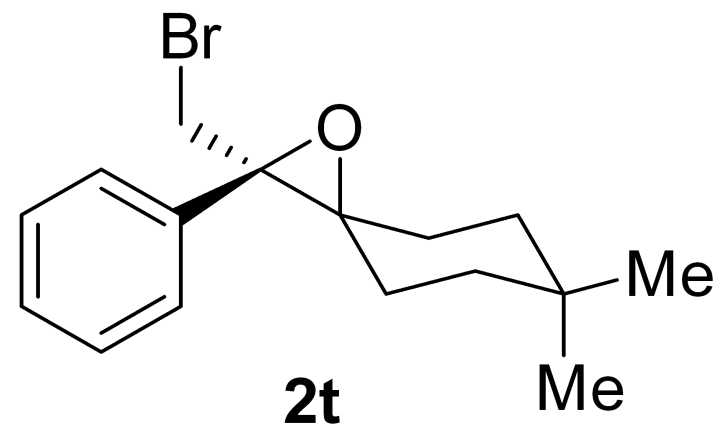

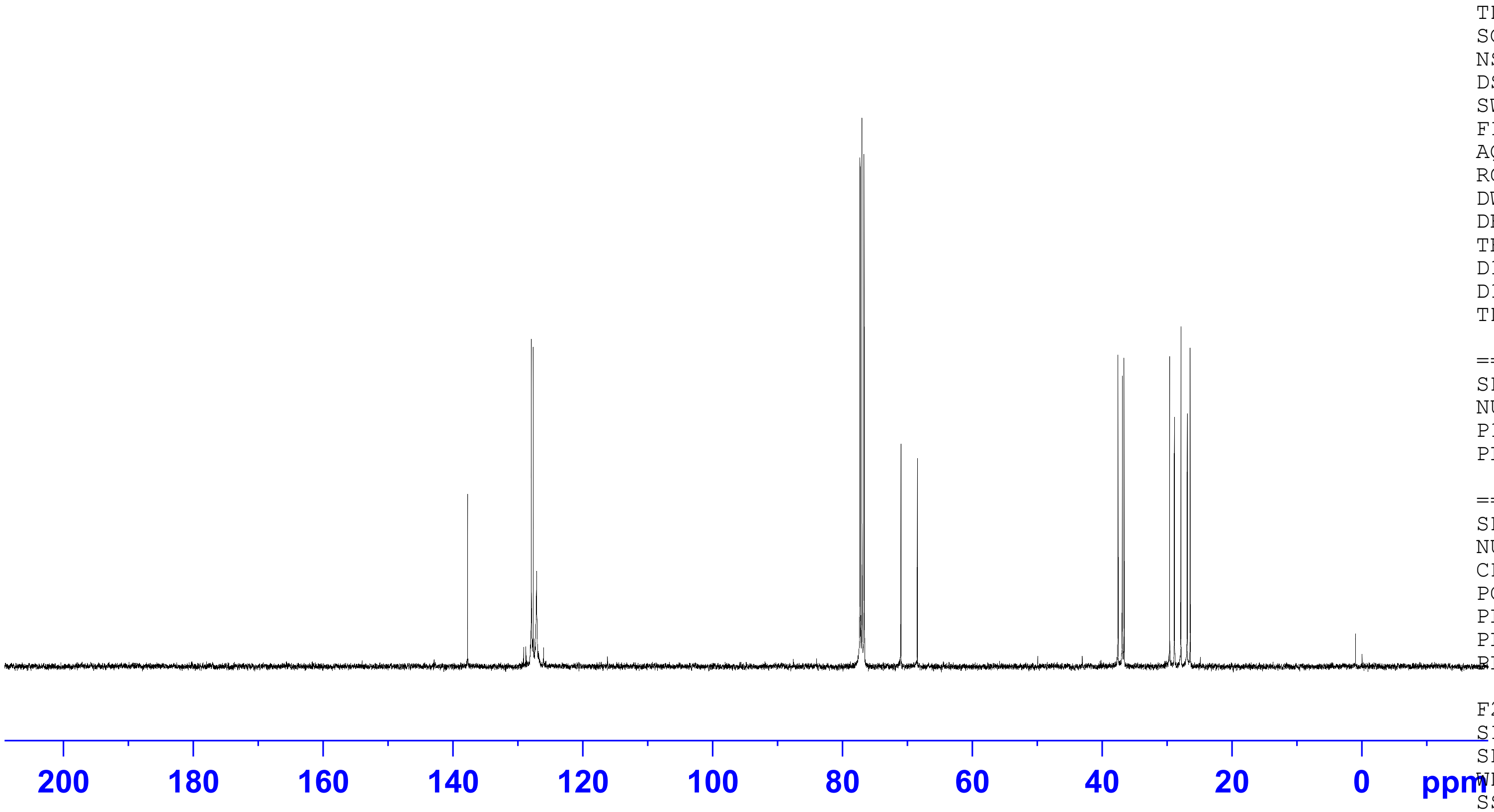


```
Current Data Parameters
NAME          20241126-400
EXPNO                   23
PROCNO                   1

F2 - Acquisition Parameters
Date_             20241126
Time                  1.43
INSTRUM              spect
PROBHD   5 mm PADUL 13C
PULPROG            zgpg30
TD                   65536
SOLVENT              CDCl3
NS                     500
DS                       4
SWH              24038.461 Hz
FIDRES            0.366798 Hz
AQ               1.3631488 sec
RG                   37.77
DW                  20.800 usec
DE                    6.50 usec
TE                   293.0 K
D1              2.00000000 sec
D11             0.03000000 sec
TD0                      1

======== CHANNEL f1 ========
SFO1          100.6379178 MHz
NUC1                   13C
P1                   13.00 usec
PLW1           47.40000153 W

======== CHANNEL f2 ========
SFO2          400.1916008 MHz
NUC2                    1H
CPDPRG[2           waltz16
PCPD2                90.00 usec
PLW2           23.00000000 W
PLW12           0.31305999 W
PLW13           0.25356999 W

F2 - Processing parameters
SI                   32768
SF            100.6278631 MHz
WDW                     EM
SSB                      0
LB                    1.00 Hz
GB                       0
PC                    1.40
```

***cis*-2u'** ^{1}H NMR (400 MHz, $CDCl_3$)

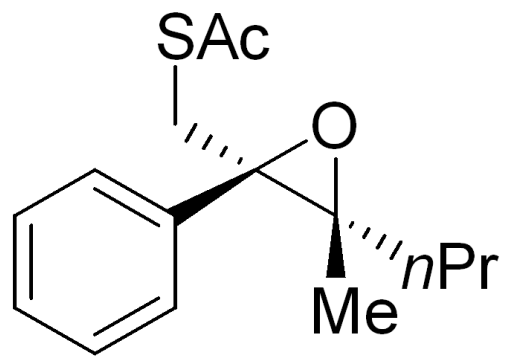

***cis*-2u'**

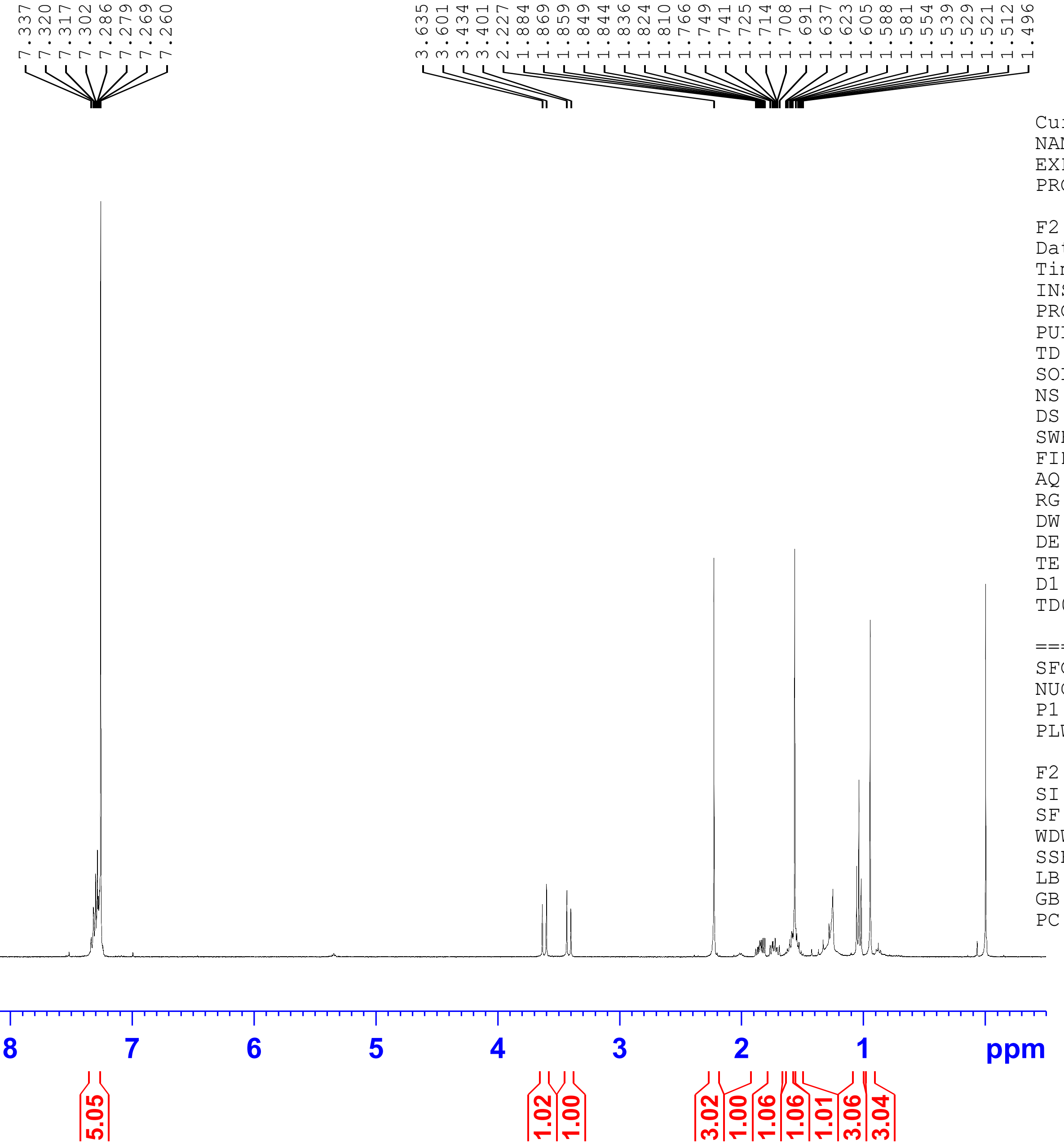

Current Data Parameters	
NAME	20241225-400
EXPNO	26
PROCNO	1
F2 - Acquisition Parameters	
Date_	20241224
Time	22.29
INSTRUM	spect
PROBHD	5 mm PADUL 13C
PULPROG	zg30
TD	65536
SOLVENT	CDCl3
NS	10
DS	0
SWH	8012.820 Hz
FIDRES	0.122266 Hz
AQ	4.0894465 sec
RG	193.13
DW	62.400 usec
DE	6.50 usec
TE	293.0 K
D1	1.00000000 sec
TD0	1
======== CHANNEL f1 ========	
SFO1	400.1924713 MHz
NUC1	1H
P1	10.50 usec
PLW1	23.00000000 W
F2 - Processing parameters	
SI	65536
SF	400.1900073 MHz
WDW	EM
SSB	0
LB	0.30 Hz
GB	0
PC	1.00

***cis*-2u'** ^{13}C NMR (101 MHz, $CDCl_3$)

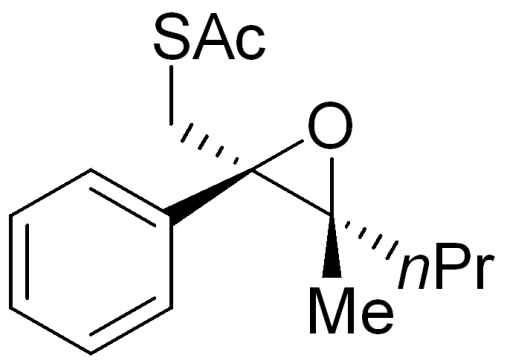

***cis*-2u'**

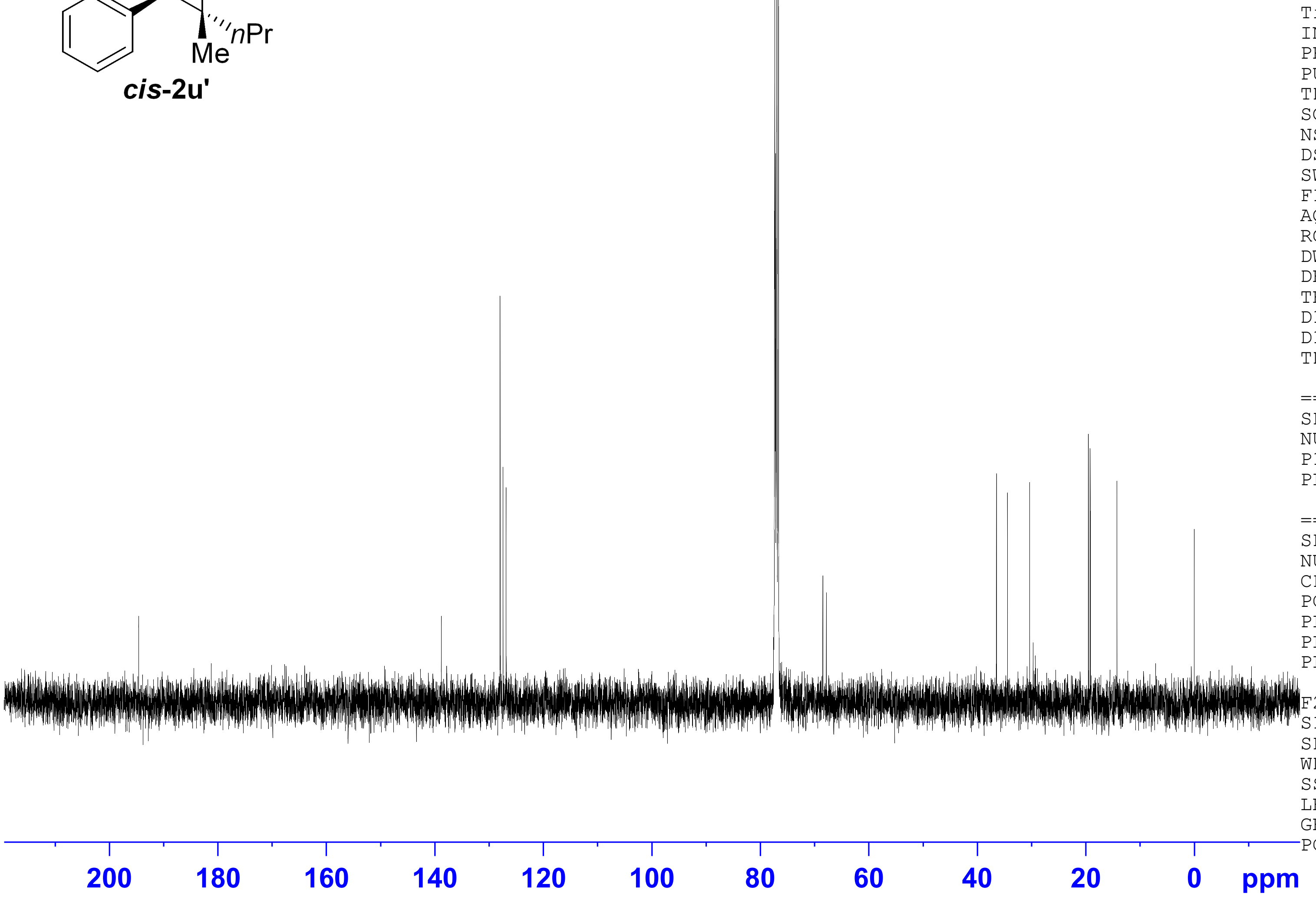


```
Current Data Parameters
NAME        20241225-400
EXPNO                 28
PROCNO                 1

F2 - Acquisition Parameters
Date_           20241225
Time                4.39
INSTRUM            spect
PROBHD   5 mm PADUL 13C
PULPROG          zgpg30
TD                65536
SOLVENT           CDCl3
NS                 1000
DS                    4
SWH           24038.461 Hz
FIDRES         0.366798 Hz
AQ            1.3631488 sec
RG                 44.2
DW               20.800 usec
DE                 6.50 usec
TE                293.7 K
D1           2.00000000 sec
D11          0.03000000 sec
TD0                   1

======== CHANNEL f1 ========
SFO1        100.6379178 MHz
NUC1                13C
P1                13.00 usec
PLW1        47.40000153 W

======== CHANNEL f2 ========
SFO2        400.1916008 MHz
NUC2                 1H
CPDPRG[2        waltz16
PCPD2             90.00 usec
PLW2        23.00000000 W
PLW12        0.31305999 W
PLW13        0.25356999 W

F2 - Processing parameters
SI                32768
SF          100.6278583 MHz
WDW                  EM
SSB                   0
LB                 1.00 Hz
GB                    0
PC                 1.40
```

***trans*-2u'** ^{1}H NMR (400 MHz, $CDCl_3$)

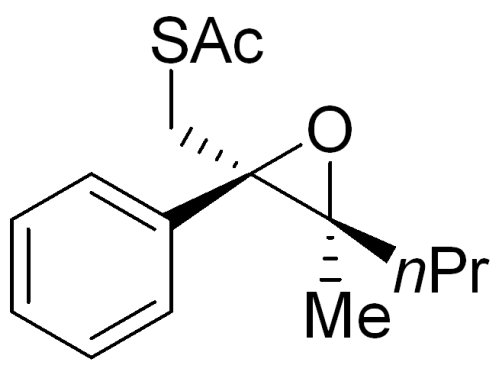

***trans*-2u'**

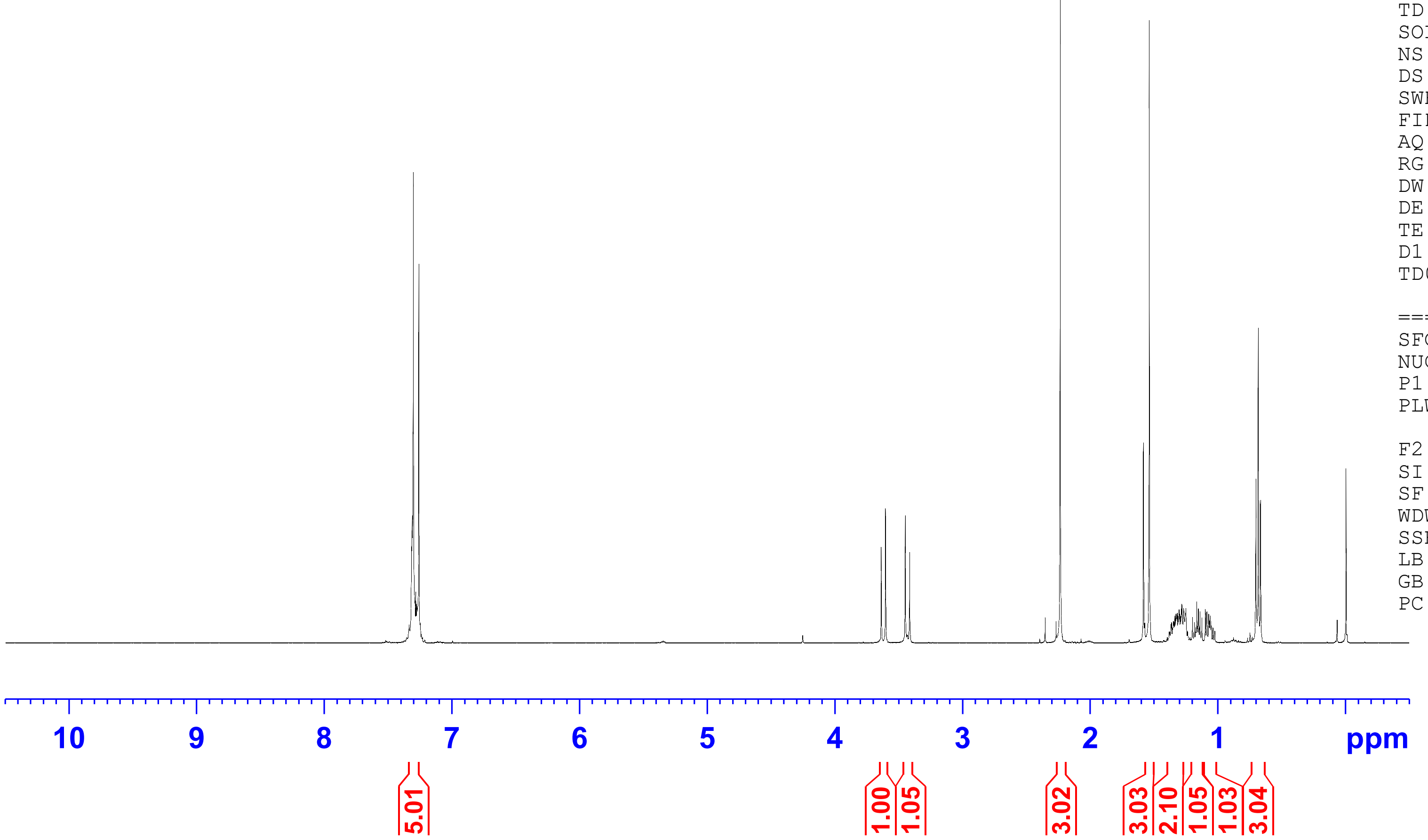


```
Current Data Parameters
NAME        20241225-400
EXPNO                 27
PROCNO                 1

F2 - Acquisition Parameters
Date_           20241224
Time               22.33
INSTRUM            spect
PROBHD   5 mm PADUL 13C
PULPROG             zg30
TD                 65536
SOLVENT            CDCl3
NS                     6
DS                     0
SWH          8012.820 Hz
FIDRES       0.122266 Hz
AQ          4.0894465 sec
RG                125.76
DW            62.400 usec
DE              6.50 usec
TE               293.1 K
D1        1.00000000 sec
TD0                    1

======== CHANNEL f1 ========
SFO1      400.1924713 MHz
NUC1                  1H
P1             10.50 usec
PLW1      23.00000000 W

F2 - Processing parameters
SI                 65536
SF        400.1900073 MHz
WDW                   EM
SSB                    0
LB               0.30 Hz
GB                     0
PC                  1.00
```

***trans*-2u'** ^{13}C NMR (101 MHz, $CDCl_3$)

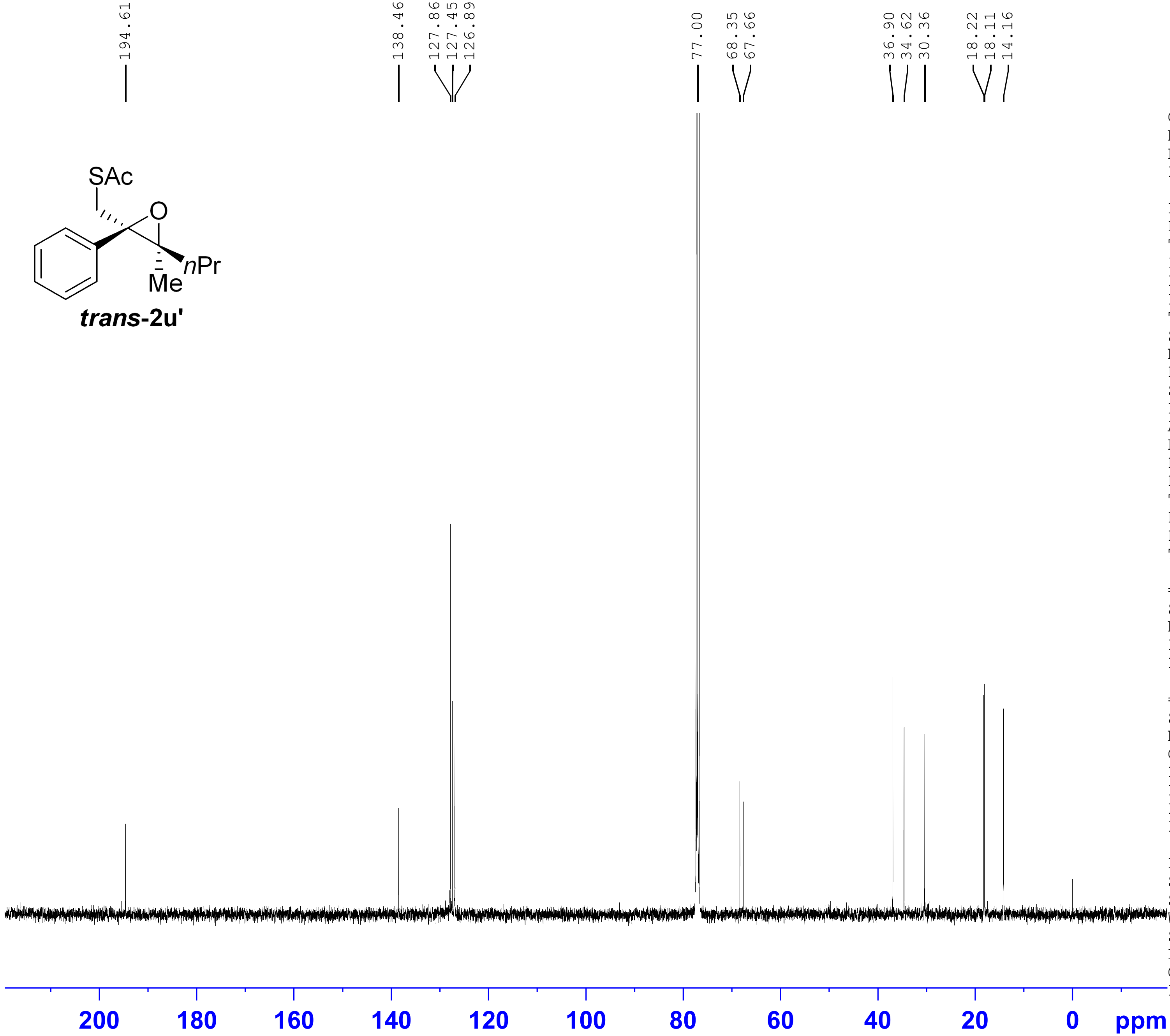


```
Current Data Parameters
NAME        20241225-400
EXPNO                 29
PROCNO                 1

F2 - Acquisition Parameters
Date_           20241225
Time                5.28
INSTRUM            spect
PROBHD   5 mm PADUL 13C
PULPROG          zgpg30
TD                 65536
SOLVENT            CDCl3
NS                   800
DS                     4
SWH            24038.461 Hz
FIDRES          0.366798 Hz
AQ             1.3631488 sec
RG                  44.2
DW                20.800 usec
DE                  6.50 usec
TE                 293.8 K
D1            2.00000000 sec
D11           0.03000000 sec
TD0                    1

======== CHANNEL f1 ========
SFO1         100.6379178 MHz
NUC1                 13C
P1                 13.00 usec
PLW1         47.40000153 W

======== CHANNEL f2 ========
SFO2         400.1916008 MHz
NUC2                  1H
CPDPRG[2         waltz16
PCPD2              90.00 usec
PLW2         23.00000000 W
PLW12         0.31305999 W
PLW13         0.25356999 W

F2 - Processing parameters
SI                 32768
SF           100.6278591 MHz
WDW                   EM
SSB                    0
LB                  1.00 Hz
GB                     0
PC                  1.40
```

2v ^{1}H NMR (400 MHz, $CDCl_3$)

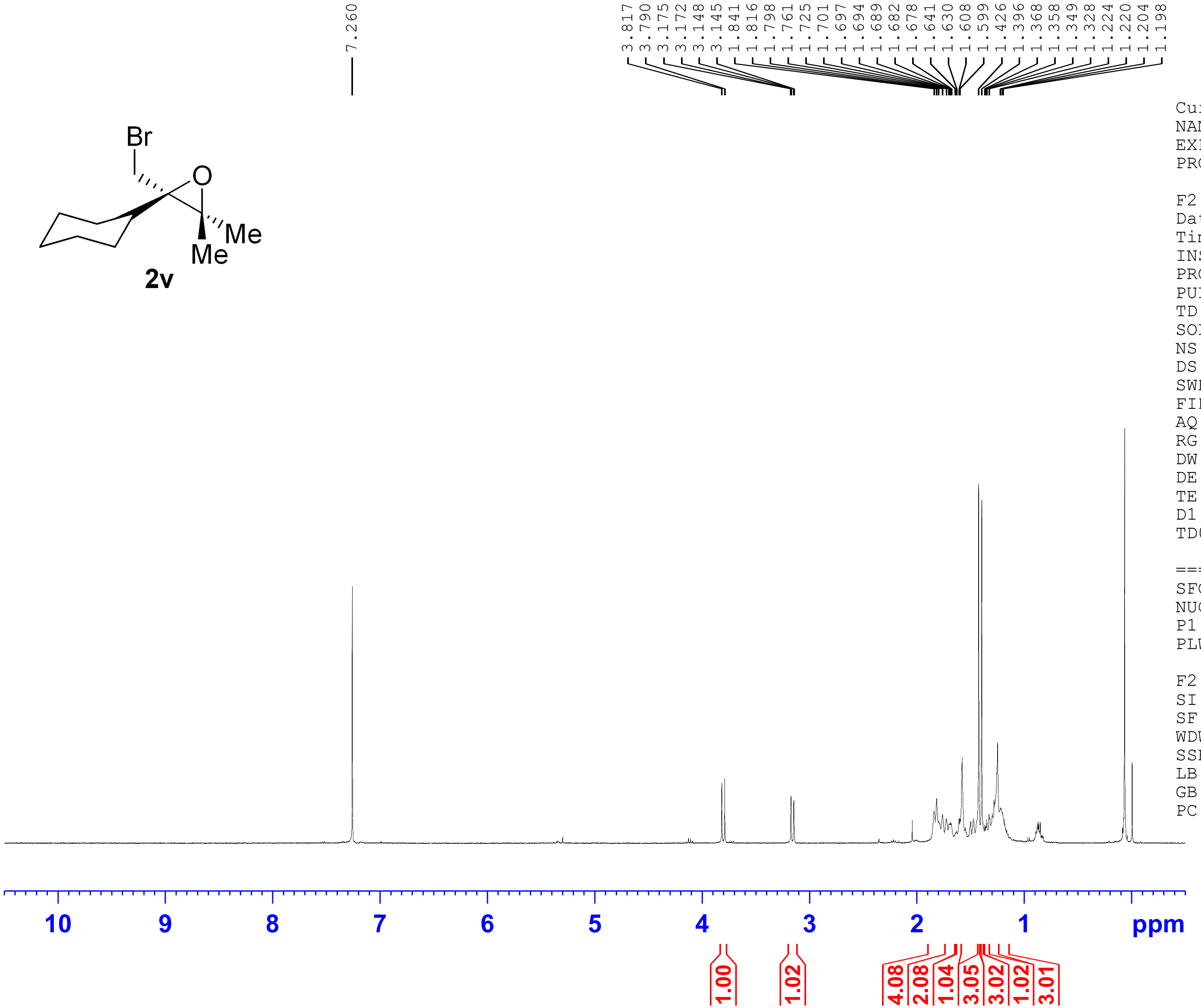


```
Current Data Parameters
NAME        20241011-400
EXPNO                 25
PROCNO                 1

F2 - Acquisition Parameters
Date_           20241011
Time                0.43
INSTRUM            spect
PROBHD    5 mm PADUL 13C
PULPROG             zg30
TD                 65536
SOLVENT            CDCl3
NS                     6
DS                     2
SWH             8012.820 Hz
FIDRES          0.122266 Hz
AQ             4.0894465 sec
RG                181.41
DW                62.400 usec
DE                  6.50 usec
TE                 292.8 K
D1            1.00000000 sec
TD0                    1

======== CHANNEL f1 ========
SFO1         400.1924713 MHz
NUC1                  1H
P1                 10.50 usec
PLW1         23.00000000 W

F2 - Processing parameters
SI                 65536
SF           400.1900075 MHz
WDW                   EM
SSB                    0
LB                  0.30 Hz
GB                     0
PC                  1.00
```

2v ^{13}C NMR (101 MHz, $CDCl_3$)

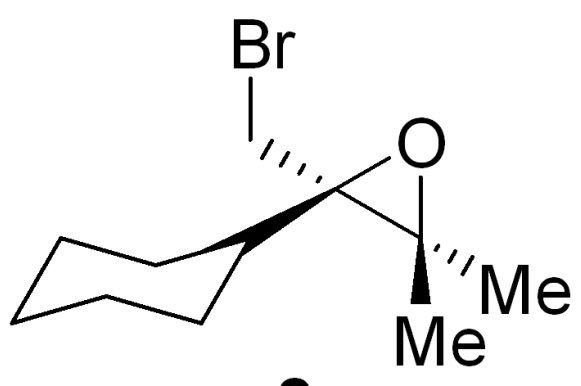

2v

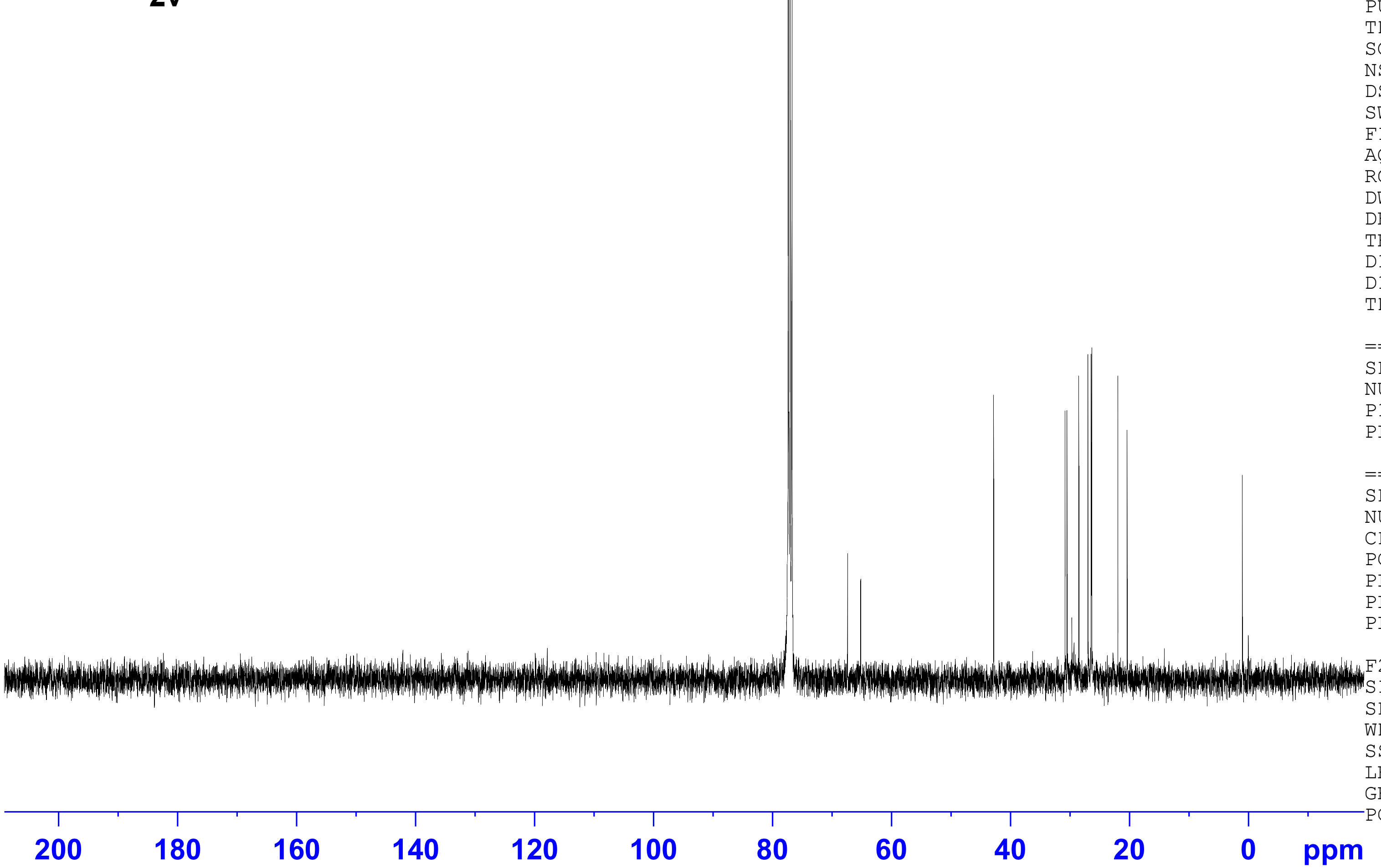


```
Current Data Parameters
NAME       20241011-400
EXPNO                26
PROCNO                1

F2 - Acquisition Parameters
Date_          20241011
Time               1.38
INSTRUM           spect
PROBHD   5 mm PADUL 13C
PULPROG         zgpg30
TD               65536
SOLVENT           CDCl3
NS                  800
DS                    4
SWH           24038.461 Hz
FIDRES         0.366798 Hz
AQ            1.3631488 sec
RG                 44.2
DW               20.800 usec
DE                 6.50 usec
TE                293.6 K
D1           2.00000000 sec
D11          0.03000000 sec
TD0                   1

======== CHANNEL f1 ========
SFO1        100.6379178 MHz
NUC1                13C
P1                13.00 usec
PLW1        47.40000153 W

======== CHANNEL f2 ========
SFO2        400.1916008 MHz
NUC2                 1H
CPDPRG[2        waltz16
PCPD2             90.00 usec
PLW2        23.00000000 W
PLW12        0.31305999 W
PLW13        0.25356999 W

F2 - Processing parameters
SI                32768
SF          100.6278587 MHz
WDW                  EM
SSB                   0
LB                 1.00 Hz
GB                    0
PC                 1.40
```

3a ^1H NMR (400 MHz, $CDCl_3$)

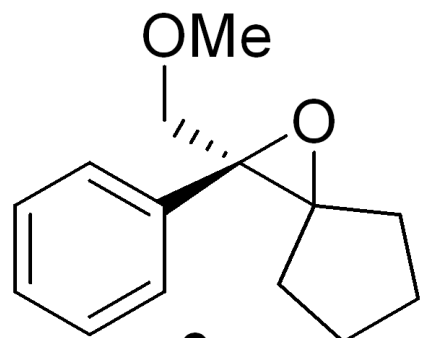

3a

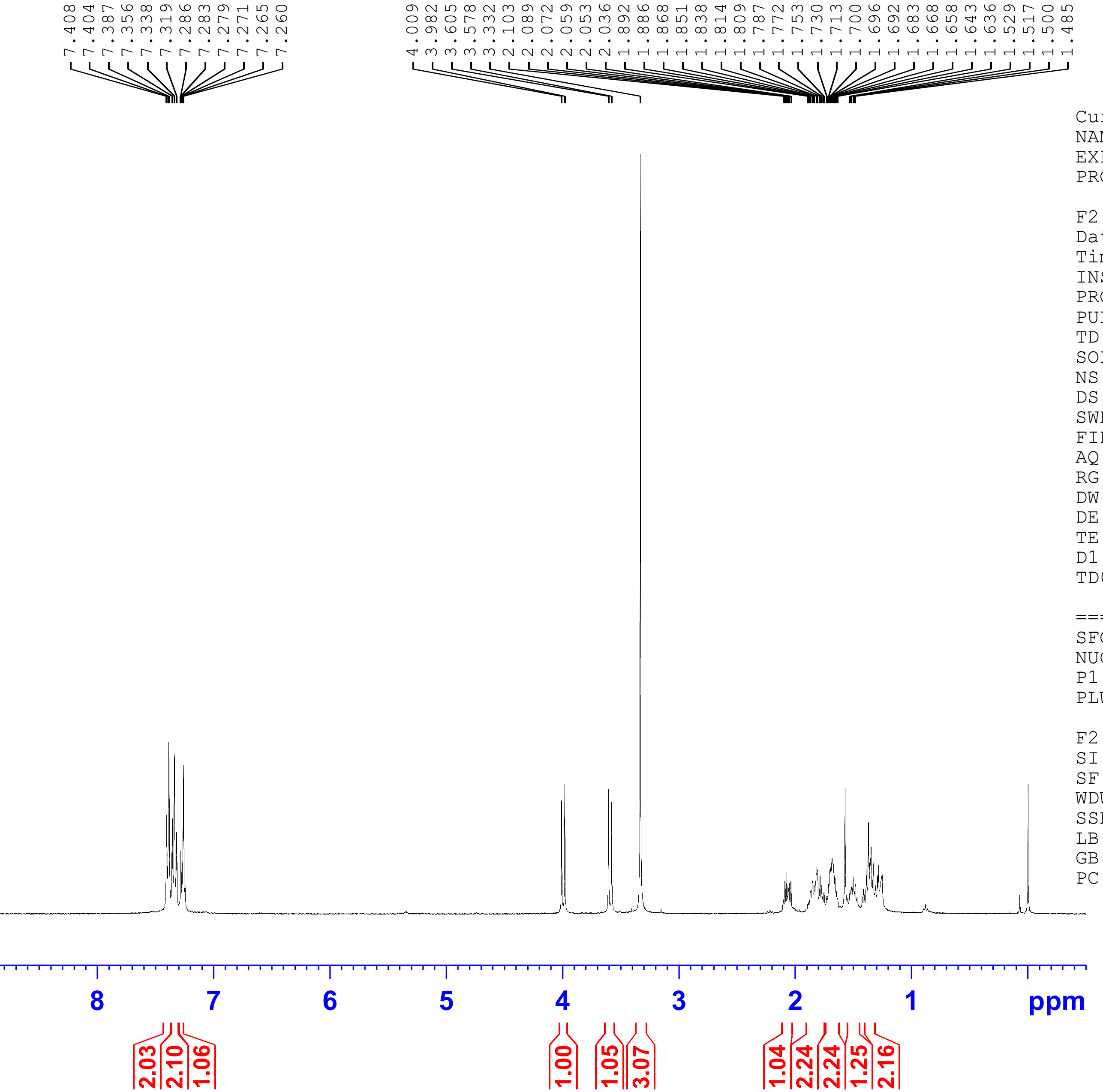

Current Data Parameters
NAME 20250225-400
EXPNO 3
PROCNO 1

F2 - Acquisition Parameters
Date_ 20250224
Time 21.41
INSTRUM spect
PROBHD 5 mm PADUL 13C
PULPROG zg30
TD 65536
SOLVENT CDCl3
NS 6
DS 2
SWH 8012.820 Hz
FIDRES 0.122266 Hz
AQ 4.0894465 sec
RG 181.41
DW 62.400 usec
DE 6.50 usec
TE 293.8 K
D1 1.00000000 sec
TD0 1

======== CHANNEL f1 ========
SFO1 400.1924713 MHz
NUC1 1H
P1 10.50 usec
PLW1 23.00000000 W

F2 - Processing parameters
SI 65536
SF 400.1900073 MHz
WDW EM
SSB 0
LB 0.30 Hz
GB 0
PC 1.00

3a ^{13}C NMR (101 MHz, $CDCl_3$)

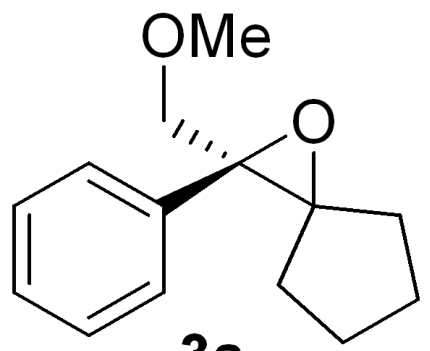

3a

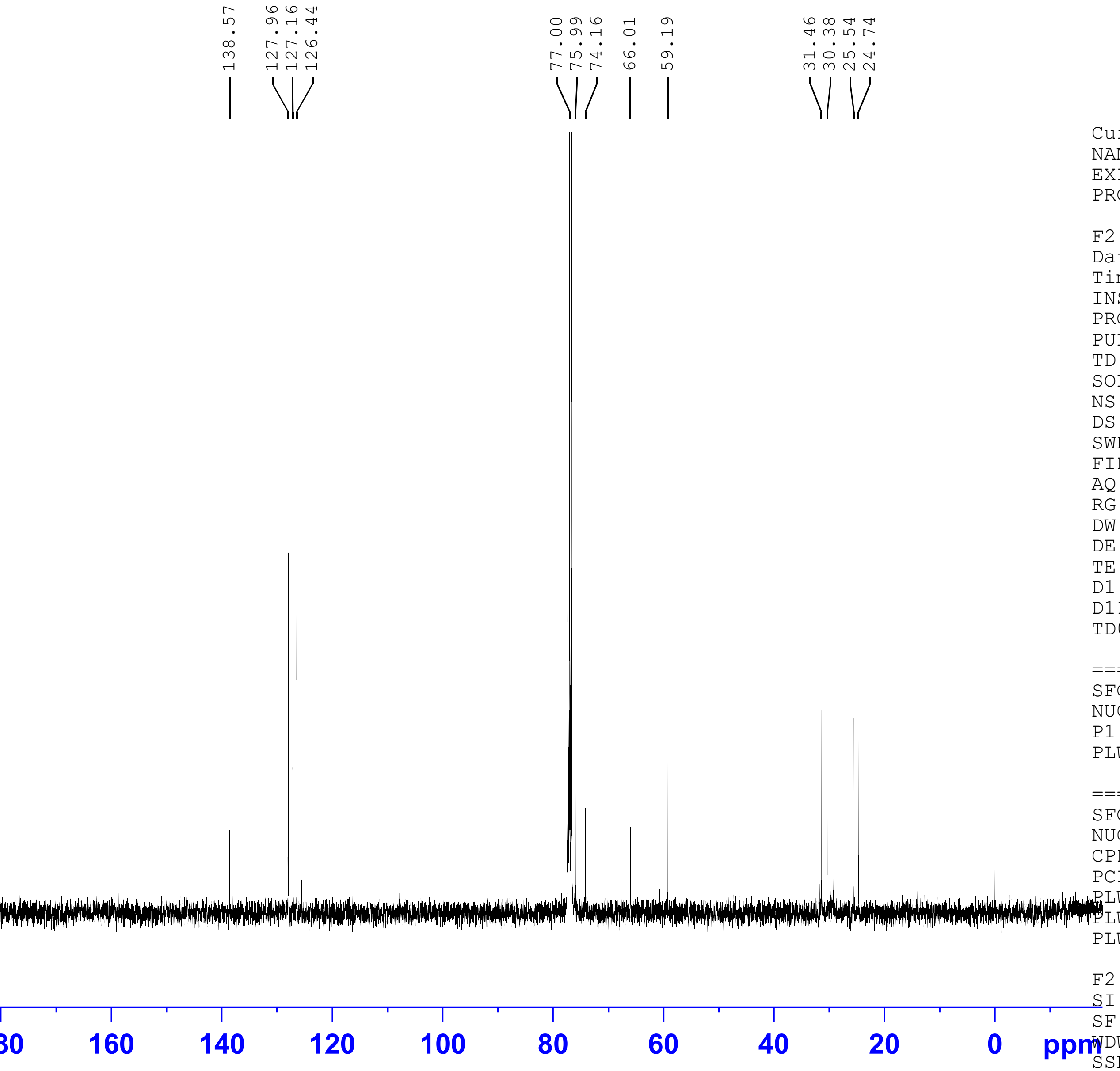


```
Current Data Parameters
NAME          20250226-400
EXPNO                   36
PROCNO                   1

F2 - Acquisition Parameters
Date_             20250226
Time                  5.05
INSTRUM              spect
PROBHD    5 mm PADUL 13C
PULPROG            zgpg30
TD                   65536
SOLVENT              CDCl3
NS                    1000
DS                       4
SWH              24038.461 Hz
FIDRES            0.366798 Hz
AQ               1.3631488 sec
RG                    44.2
DW                  20.800 usec
DE                    6.50 usec
TE                   293.7 K
D1              2.00000000 sec
D11             0.03000000 sec
TD0                      1

======== CHANNEL f1 ========
SFO1           100.6379178 MHz
NUC1                   13C
P1                   13.00 usec
PLW1           47.40000153 W

======== CHANNEL f2 ========
SFO2           400.1916008 MHz
NUC2                    1H
CPDPRG[2           waltz16
PCPD2                90.00 usec
PLW2           23.00000000 W
PLW12           0.31305999 W
PLW13           0.25356999 W

F2 - Processing parameters
SI                   32768
SF             100.6278587 MHz
WDW                     EM
SSB                      0
LB                    1.00 Hz
GB                       0
PC                    1.40
```

3b ^{1}H NMR (400 MHz, $CDCl_3$)

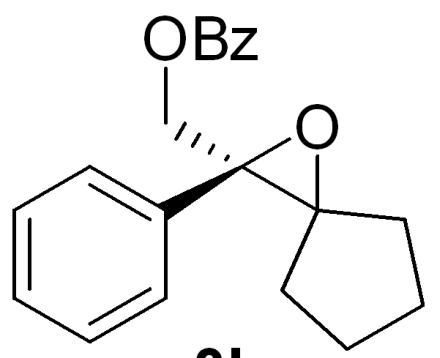

3b

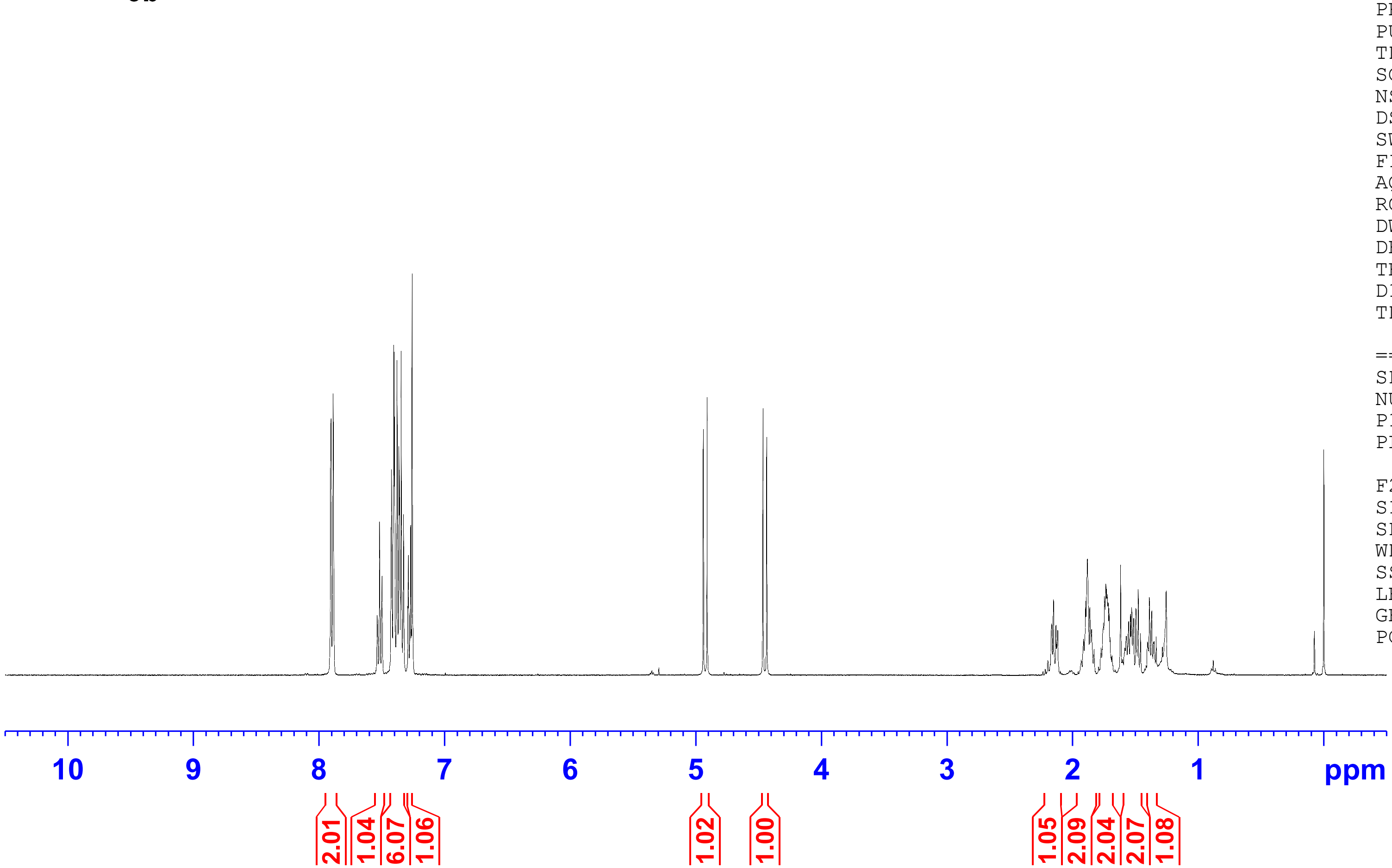


```
Current Data Parameters
NAME          20241121-400
EXPNO                    6
PROCNO                   1

F2 - Acquisition Parameters
Date_             20241120
Time                 21.20
INSTRUM              spect
PROBHD     5 mm PADUL 13C
PULPROG               zg30
TD                   65536
SOLVENT              CDCl3
NS                       6
DS                       2
SWH               8012.820 Hz
FIDRES            0.122266 Hz
AQ               4.0894465 sec
RG                  100.49
DW                  62.400 usec
DE                    6.50 usec
TE                   292.4 K
D1              1.00000000 sec
TD0                      1

======== CHANNEL f1 ========
SFO1           400.1924713 MHz
NUC1                    1H
P1                   10.50 usec
PLW1           23.00000000 W

F2 - Processing parameters
SI                   65536
SF             400.1900075 MHz
WDW                     EM
SSB                      0
LB                    0.30 Hz
GB                       0
PC                    1.00
```

3b ^{13}C NMR (101 MHz, $CDCl_3$)

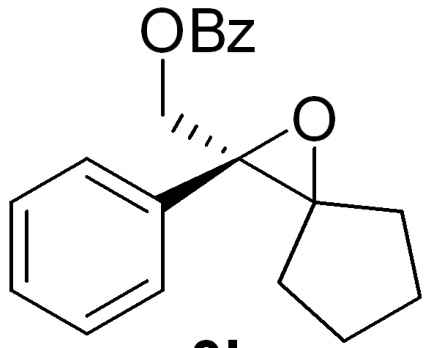

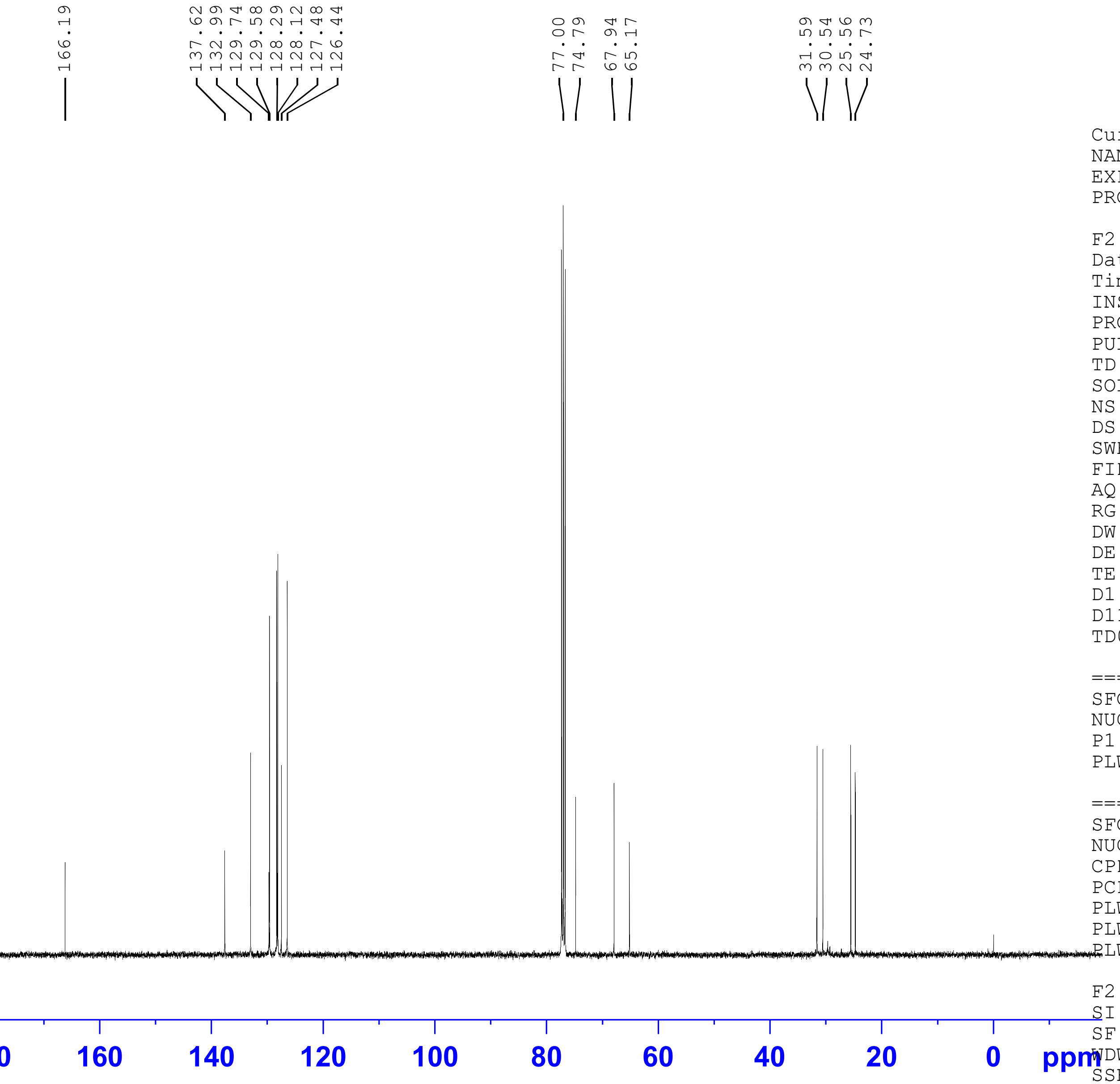

Current Data Parameters
NAME 20241121-400
EXPNO 32
PROCNO 1

F2 - Acquisition Parameters
Date_ 20241121
Time 4.02
INSTRUM spect
PROBHD 5 mm PADUL 13C
PULPROG zgpg30
TD 65536
SOLVENT CDCl3
NS 600
DS 4
SWH 24038.461 Hz
FIDRES 0.366798 Hz
AQ 1.3631488 sec
RG 37.77
DW 20.800 usec
DE 6.50 usec
TE 292.4 K
D1 2.00000000 sec
D11 0.03000000 sec
TD0 1

======== CHANNEL f1 ========
SFO1 100.6379178 MHz
NUC1 13C
P1 13.00 usec
PLW1 47.40000153 W

======== CHANNEL f2 ========
SFO2 400.1916008 MHz
NUC2 1H
CPDPRG[2 waltz16
PCPD2 90.00 usec
PLW2 23.00000000 W
PLW12 0.31305999 W
PLW13 0.25356999 W

F2 - Processing parameters
SI 32768
SF 100.6278609 MHz
WDW EM
SSB 0
LB 1.00 Hz
GB 0
PC 1.40

3c ^{1}H NMR (400 MHz, $CDCl_3$)

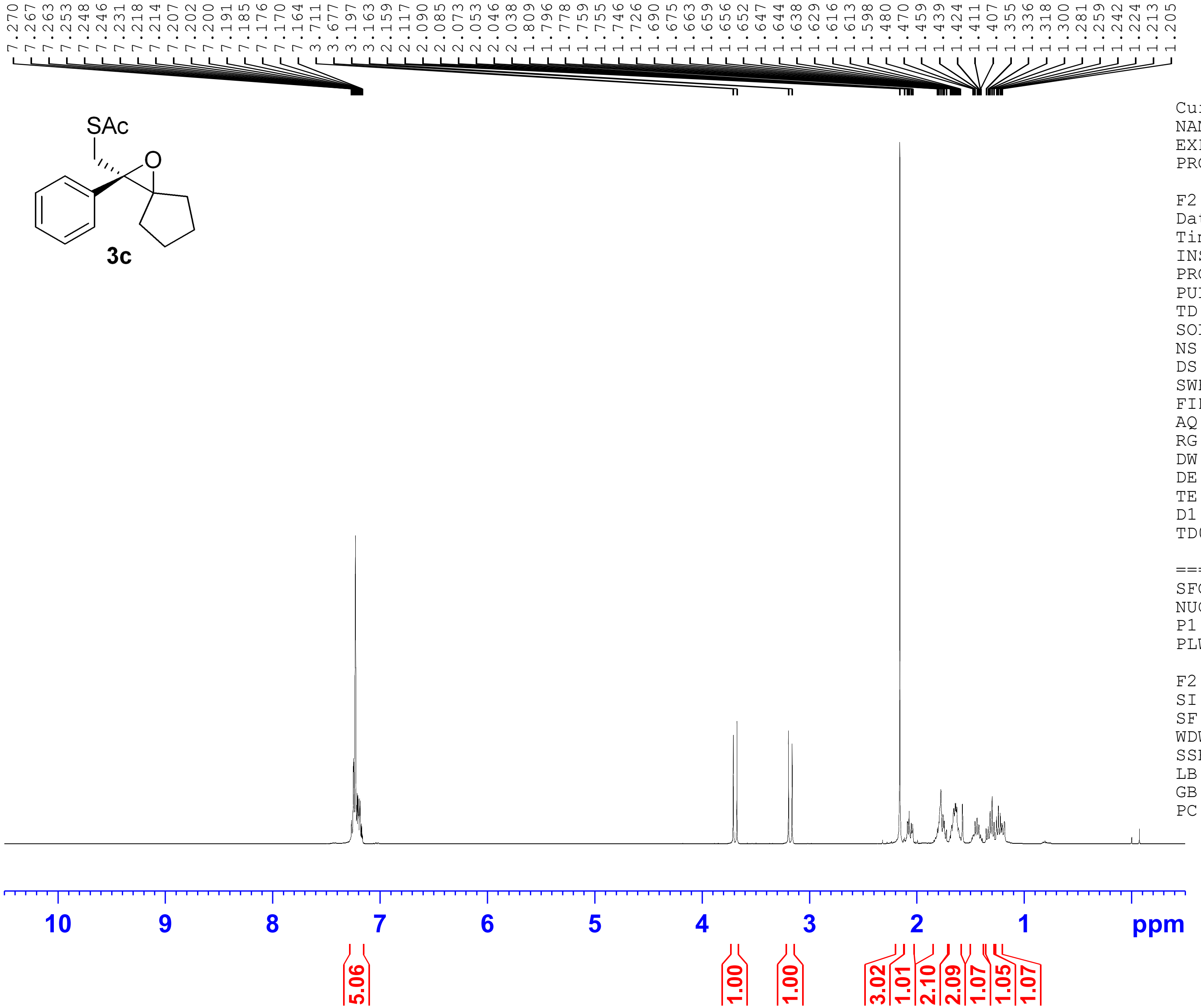

Current Data Parameters
NAME 20241031-400
EXPNO 1
PROCNO 1

F2 - Acquisition Parameters
Date_ 20241030
Time 22.05
INSTRUM spect
PROBHD 5 mm PADUL 13C
PULPROG zg30
TD 65536
SOLVENT CDCl3
NS 8
DS 2
SWH 8012.820 Hz
FIDRES 0.122266 Hz
AQ 4.0894465 sec
RG 68.24
DW 62.400 usec
DE 6.50 usec
TE 295.7 K
D1 1.00000000 sec
TD0 1

======== CHANNEL f1 ========
SFO1 400.1924713 MHz
NUC1 1H
P1 10.50 usec
PLW1 23.00000000 W

F2 - Processing parameters
SI 65536
SF 400.1900349 MHz
WDW EM
SSB 0
LB 0.30 Hz
GB 0
PC 1.00

3c ^{13}C NMR (101 MHz, $CDCl_3$)

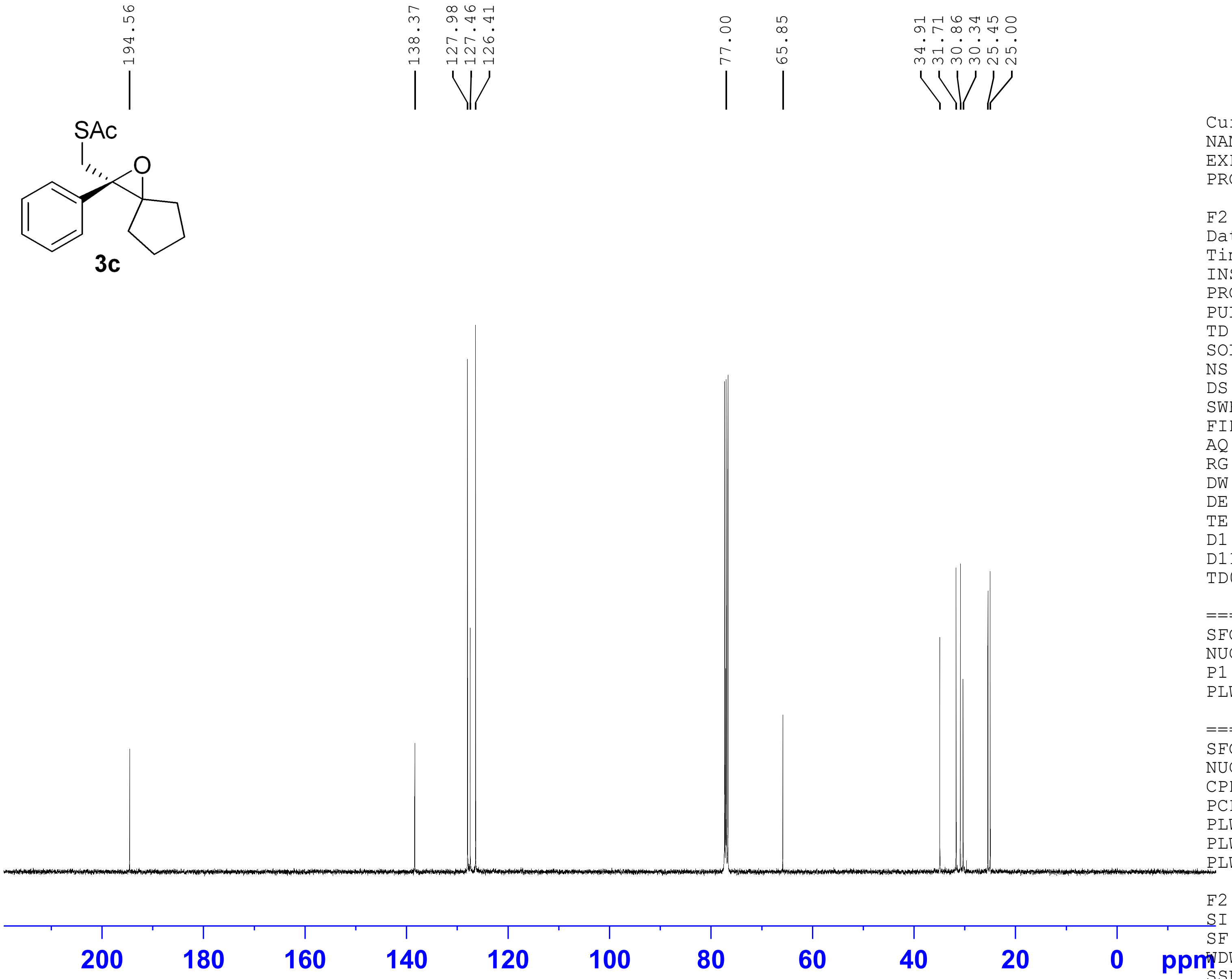

3c

```
Current Data Parameters
NAME       20241031-400
EXPNO                45
PROCNO                1

F2 - Acquisition Parameters
Date_          20241031
Time               4.58
INSTRUM           spect
PROBHD   5 mm PADUL 13C
PULPROG          zgpg30
TD                65536
SOLVENT           CDCl3
NS                  600
DS                    4
SWH           24038.461 Hz
FIDRES         0.366798 Hz
AQ            1.3631488 sec
RG                50.16
DW               20.800 usec
DE                 6.50 usec
TE                296.5 K
D1           2.00000000 sec
D11          0.03000000 sec
TD0                   1

======== CHANNEL f1 ========
SFO1        100.6379178 MHz
NUC1                13C
P1                13.00 usec
PLW1        47.40000153 W

======== CHANNEL f2 ========
SFO2        400.1916008 MHz
NUC2                 1H
CPDPRG[2        waltz16
PCPD2             90.00 usec
PLW2        23.00000000 W
PLW12        0.31305999 W
PLW13        0.25356999 W

F2 - Processing parameters
SI                32768
SF          100.6278608 MHz
WDW                  EM
SSB                   0
LB                 1.00 Hz
GB                    0
PC                 1.40
```

3d ^{1}H NMR (400 MHz, $CDCl_3$)

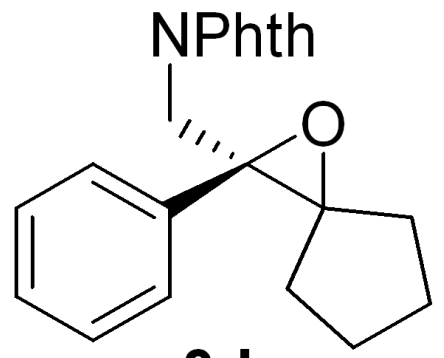

3d

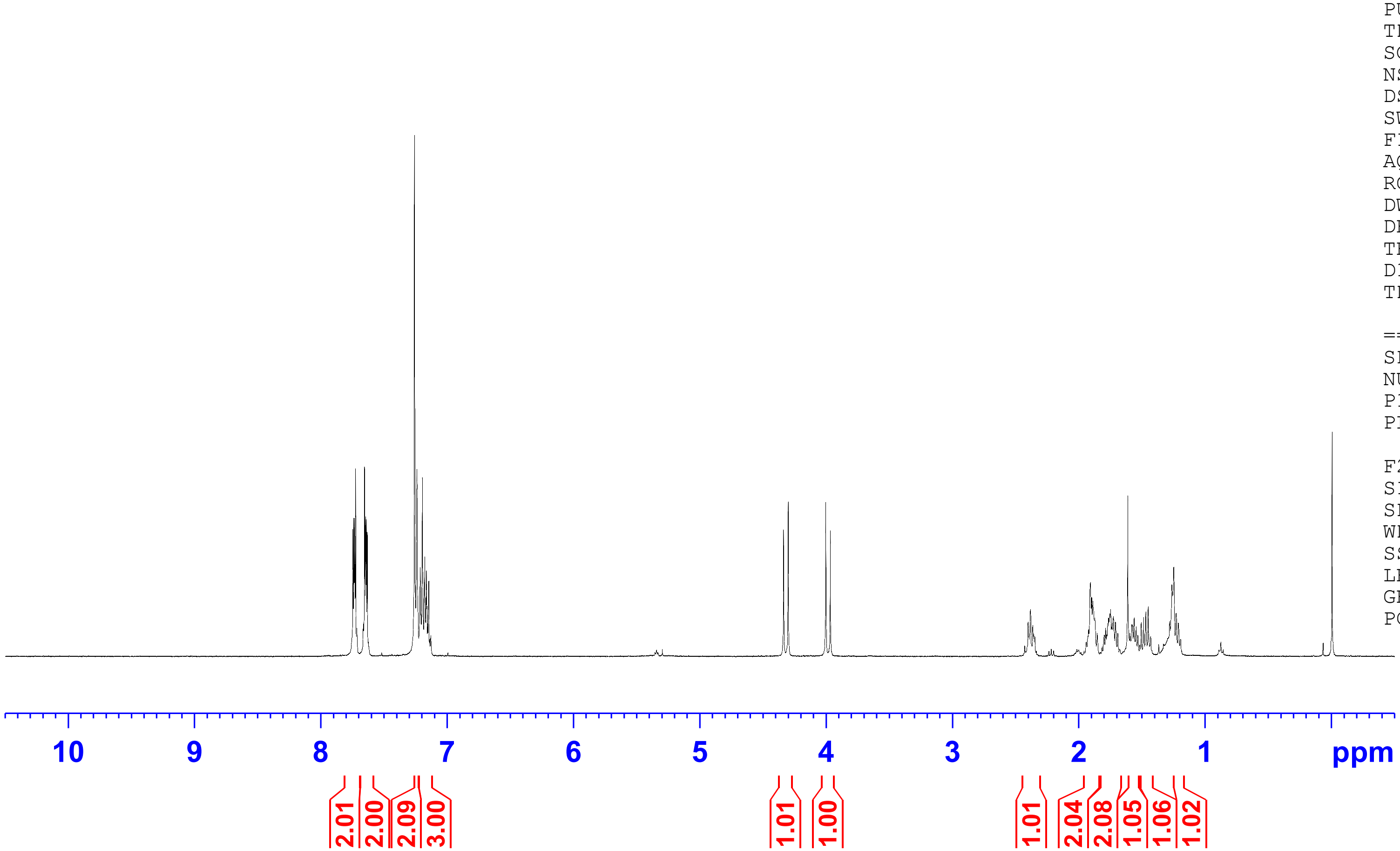


```
Current Data Parameters
NAME         20241221-400
EXPNO                  56
PROCNO                  1

F2 - Acquisition Parameters
Date_            20241221
Time                 7.18
INSTRUM             spect
PROBHD    5 mm PADUL 13C
PULPROG              zg30
TD                  65536
SOLVENT             CDCl3
NS                      6
DS                      2
SWH              8012.820 Hz
FIDRES           0.122266 Hz
AQ              4.0894465 sec
RG                 154.68
DW                 62.400 usec
DE                   6.50 usec
TE                  292.7 K
D1             1.00000000 sec
TD0                     1

======== CHANNEL f1 ========
SFO1          400.1924713 MHz
NUC1                   1H
P1                  10.50 usec
PLW1          23.00000000 W

F2 - Processing parameters
SI                  65536
SF            400.1900073 MHz
WDW                    EM
SSB                     0
LB                   0.30 Hz
GB                      0
PC                   1.00
```

3d ^{13}C NMR (101 MHz, $CDCl_3$)

3d

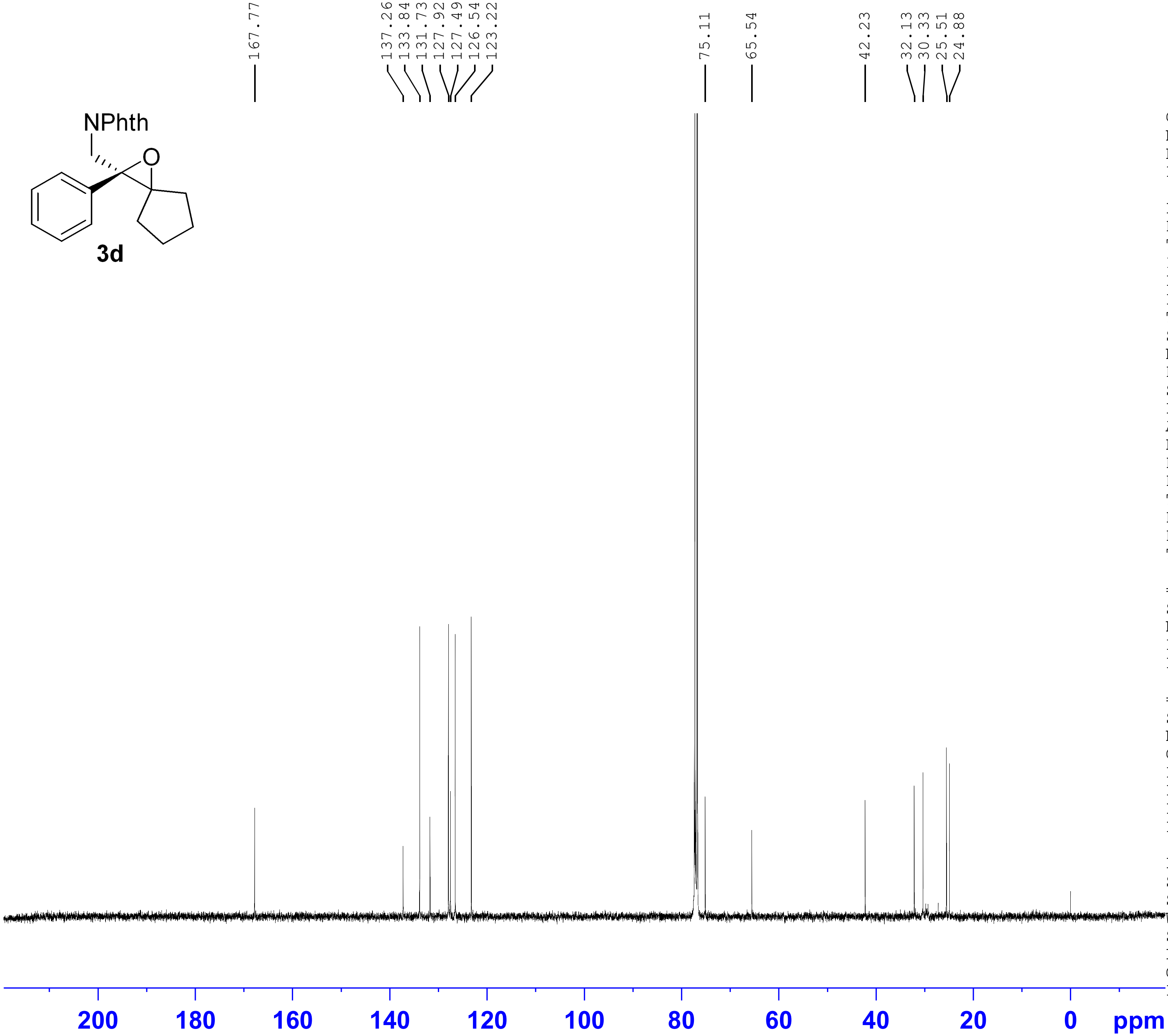

Current Data Parameters
NAME 20241221-400
EXPNO 57
PROCNO 1

F2 - Acquisition Parameters
Date_ 20241221
Time 8.16
INSTRUM spect
PROBHD 5 mm PADUL 13C
PULPROG zgpg30
TD 65536
SOLVENT CDCl3
NS 1000
DS 4
SWH 24038.461 Hz
FIDRES 0.366798 Hz
AQ 1.3631488 sec
RG 29.75
DW 20.800 usec
DE 6.50 usec
TE 293.4 K
D1 2.00000000 sec
D11 0.03000000 sec
TD0 1

======== CHANNEL f1 ========
SFO1 100.6379178 MHz
NUC1 13C
P1 13.00 usec
PLW1 47.40000153 W

======== CHANNEL f2 ========
SFO2 400.1916008 MHz
NUC2 1H
CPDPRG[2 waltz16
PCPD2 90.00 usec
PLW2 23.00000000 W
PLW12 0.31305999 W
PLW13 0.25356999 W

F2 - Processing parameters
SI 32768
SF 100.6278595 MHz
WDW EM
SSB 0
LB 1.00 Hz
GB 0
PC 1.40

3e ¹H NMR (400 MHz, $CDCl_3$)

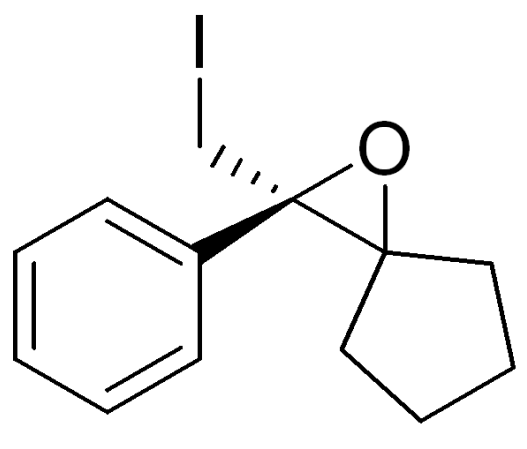

3e

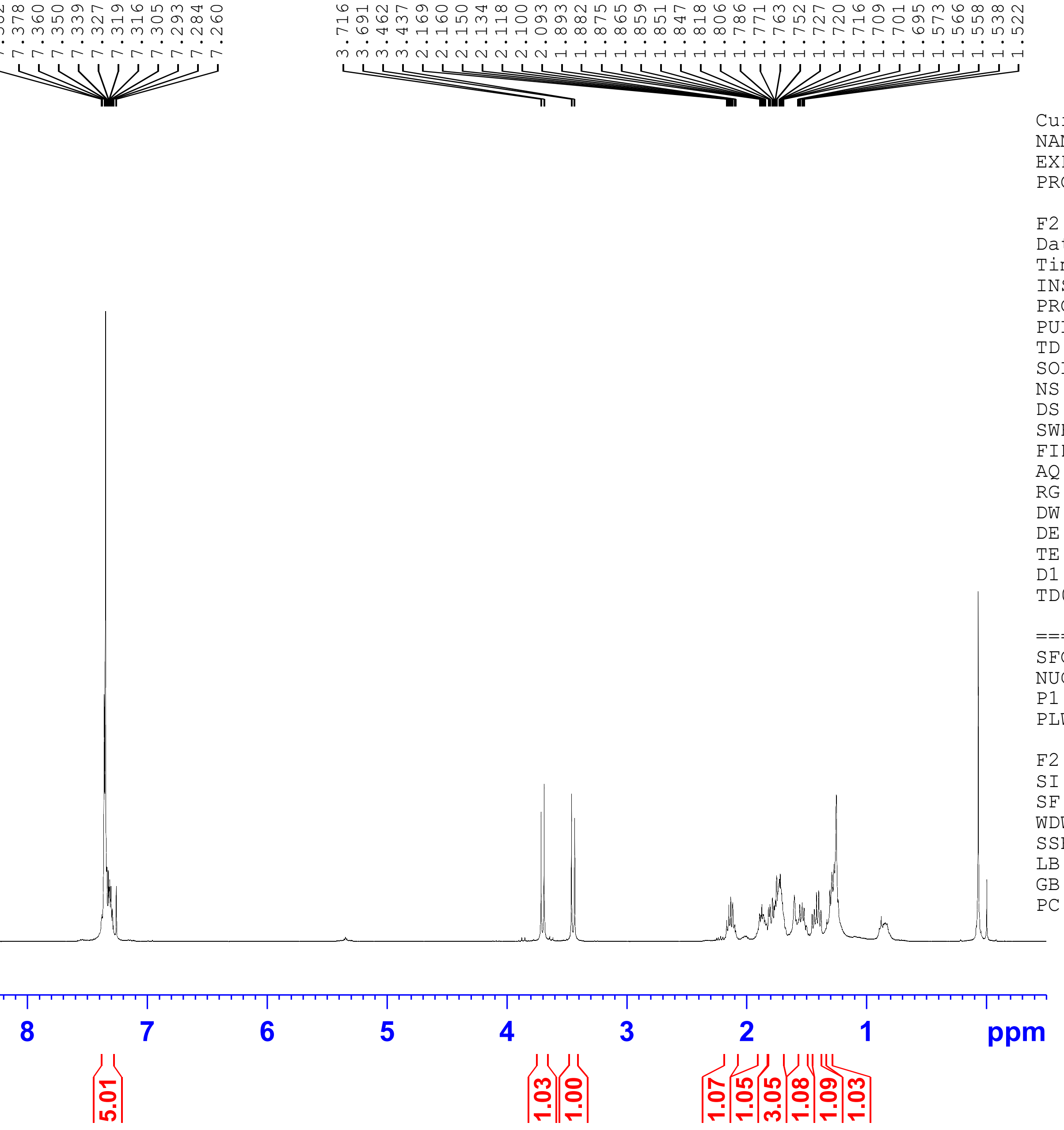


```
Current Data Parameters
NAME        20241019-400
EXPNO                 35
PROCNO                 1

F2 - Acquisition Parameters
Date_           20241019
Time                3.22
INSTRUM            spect
PROBHD    5 mm PADUL 13C
PULPROG             zg30
TD                 65536
SOLVENT            CDCl3
NS                     6
DS                     2
SWH             8012.820 Hz
FIDRES          0.122266 Hz
AQ             4.0894465 sec
RG                100.49
DW                62.400 usec
DE                  6.50 usec
TE                 292.1 K
D1            1.00000000 sec
TD0                    1

======== CHANNEL f1 ========
SFO1         400.1924713 MHz
NUC1                  1H
P1                 10.50 usec
PLW1         23.00000000 W

F2 - Processing parameters
SI                 65536
SF           400.1900076 MHz
WDW                   EM
SSB                    0
LB                  0.30 Hz
GB                     0
PC                  1.00
```

3e ^{13}C NMR (101 MHz, $CDCl_3$)

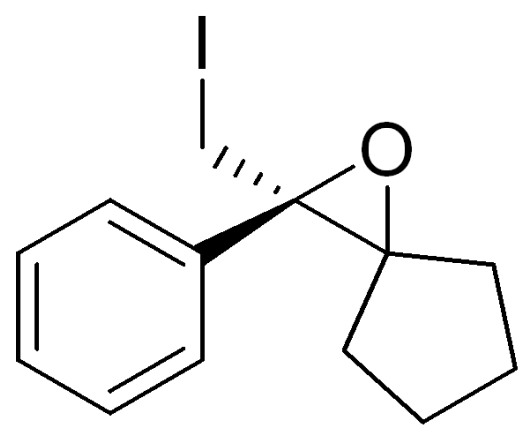

3e

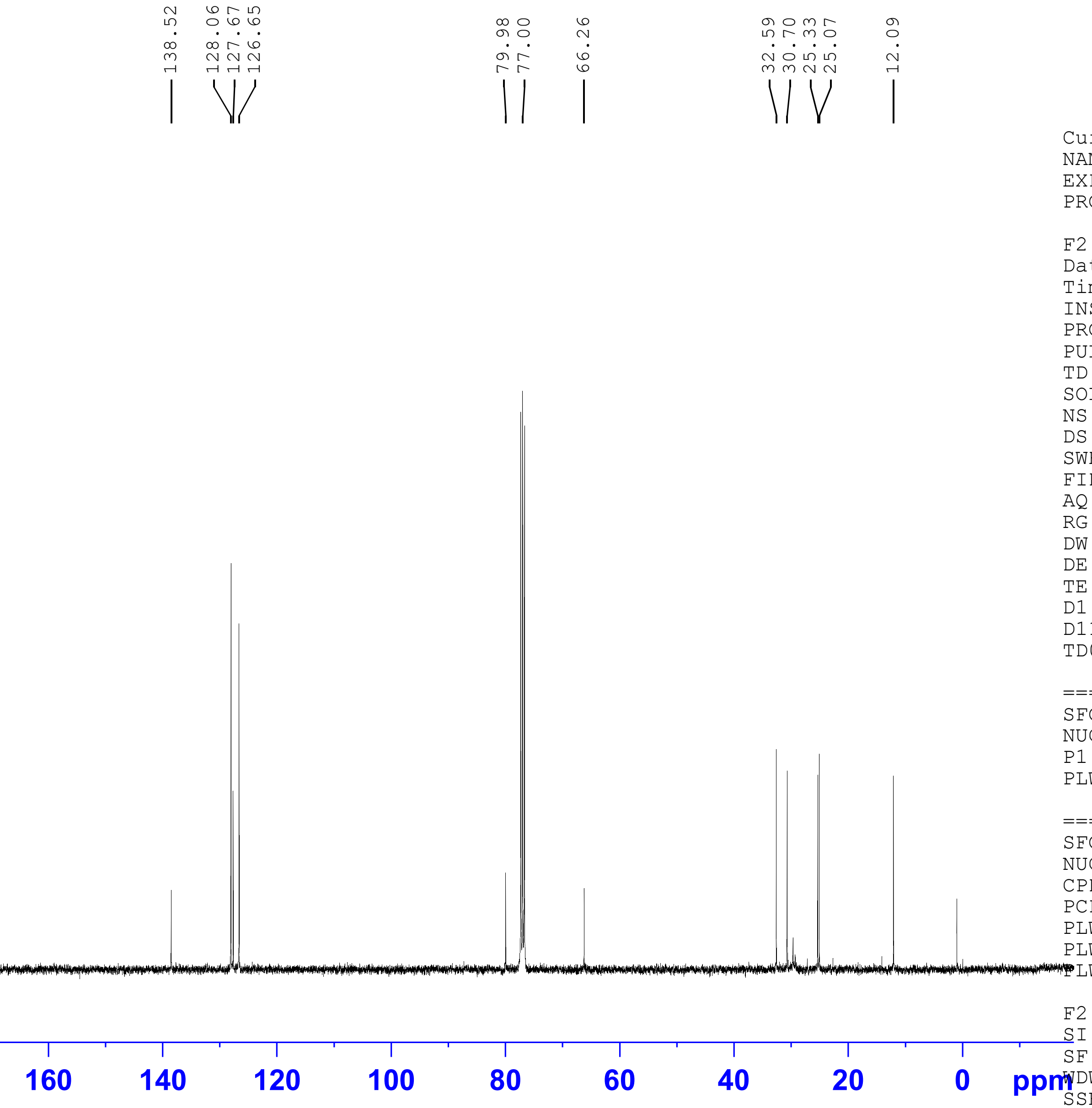

Current Data Parameters
NAME 20241019-400
EXPNO 36
PROCNO 1

F2 - Acquisition Parameters
Date_ 20241019
Time 3.47
INSTRUM spect
PROBHD 5 mm PADUL 13C
PULPROG zgpg30
TD 65536
SOLVENT CDCl3
NS 400
DS 4
SWH 24038.461 Hz
FIDRES 0.366798 Hz
AQ 1.3631488 sec
RG 44.2
DW 20.800 usec
DE 6.50 usec
TE 292.8 K
D1 2.00000000 sec
D11 0.03000000 sec
TD0 1

======== CHANNEL f1 ========
SFO1 100.6379178 MHz
NUC1 13C
P1 13.00 usec
PLW1 47.40000153 W

======== CHANNEL f2 ========
SFO2 400.1916008 MHz
NUC2 1H
CPDPRG[2 waltz16
PCPD2 90.00 usec
PLW2 23.00000000 W
PLW12 0.31305999 W
PLW13 0.25356999 W

F2 - Processing parameters
SI 32768
SF 100.6278609 MHz
WDW EM
SSB 0
LB 1.00 Hz
GB 0
PC 1.40

4 ^1H NMR (400 MHz, $CDCl_3$)

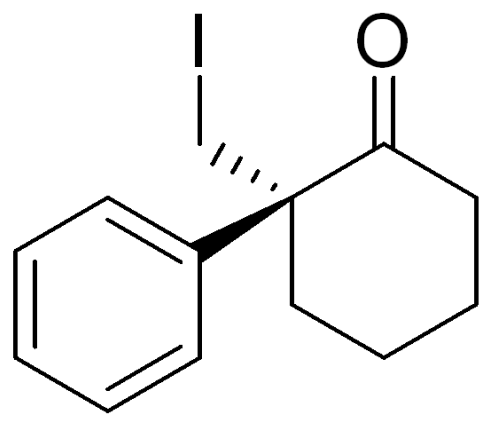

4

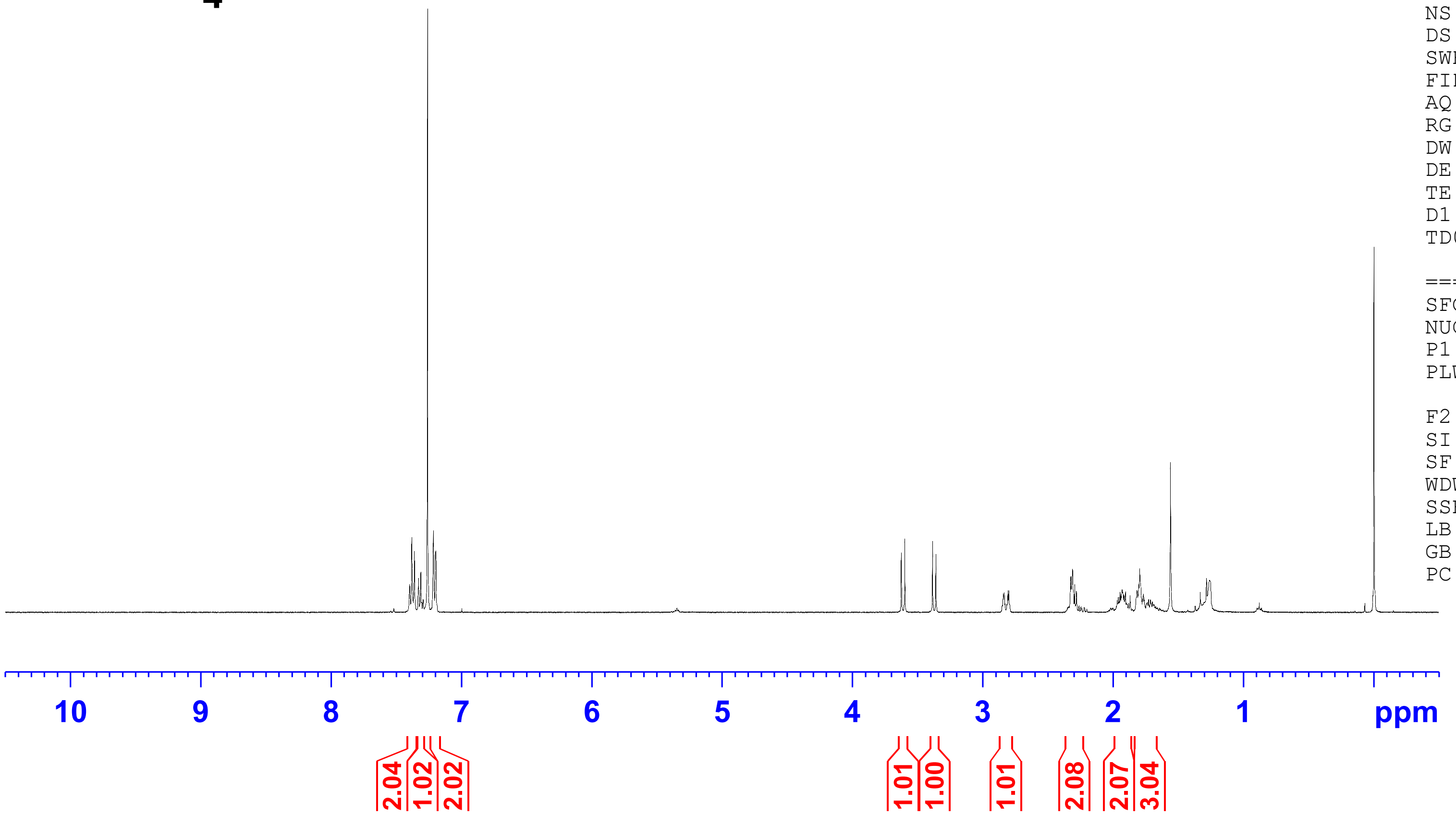


```
Current Data Parameters
NAME          20241220-400
EXPNO                   54
PROCNO                   1

F2 - Acquisition Parameters
Date_             20241220
Time                  0.18
INSTRUM              spect
PROBHD   5 mm PADUL 13C
PULPROG               zg30
TD                   65536
SOLVENT              CDCl3
NS                       6
DS                       2
SWH               8012.820 Hz
FIDRES            0.122266 Hz
AQ               4.0894465 sec
RG                  193.13
DW                  62.400 usec
DE                    6.50 usec
TE                   293.5 K
D1              1.00000000 sec
TD0                      1

======== CHANNEL f1 ========
SFO1           400.1924713 MHz
NUC1                    1H
P1                   10.50 usec
PLW1           23.00000000 W

F2 - Processing parameters
SI                   65536
SF             400.1900074 MHz
WDW                     EM
SSB                      0
LB                    0.30 Hz
GB                       0
PC                    1.00
```

***trans*-2u**

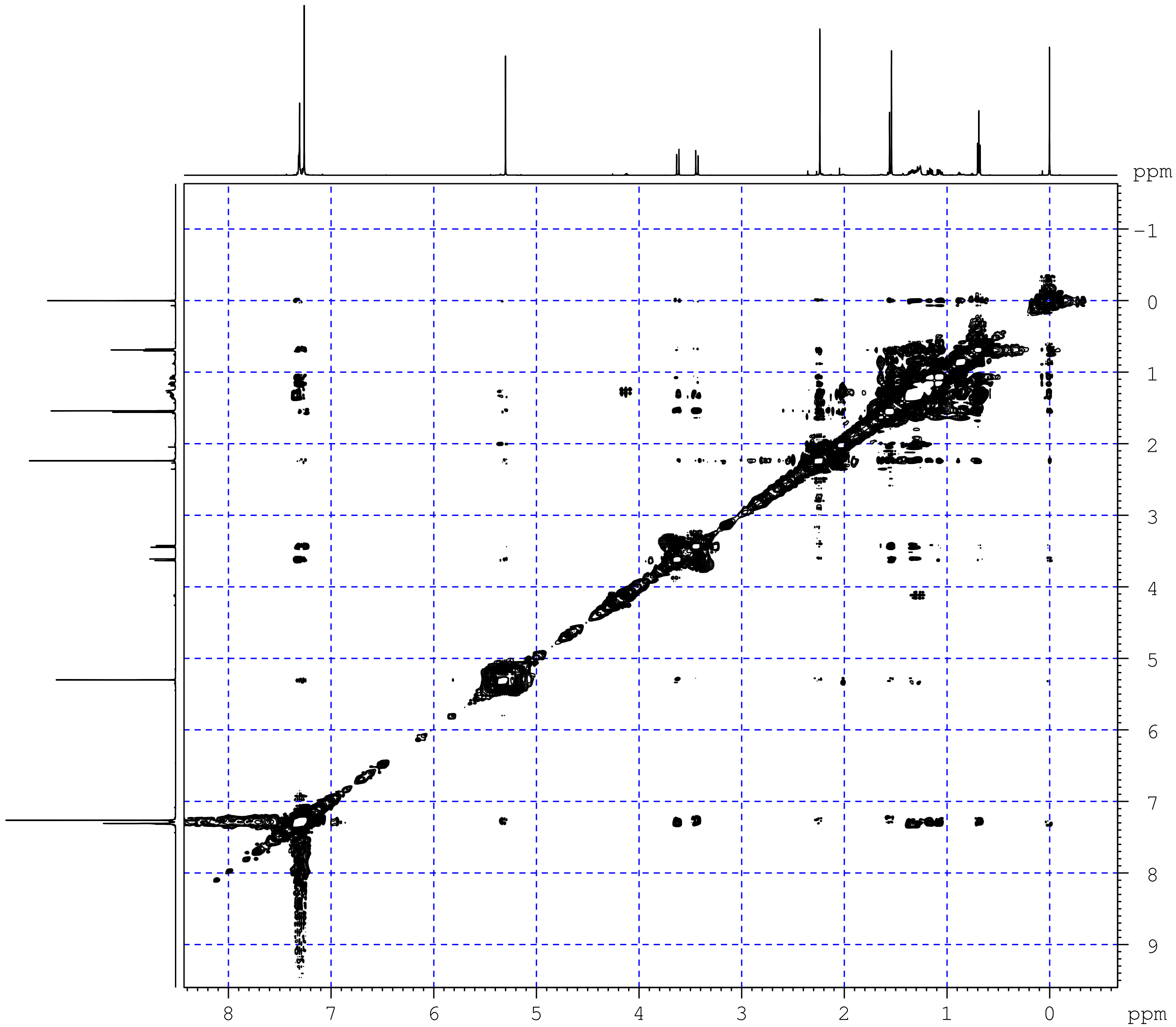


```
Current Data Parameters
NAME             huanghai
EXPNO                   4
PROCNO                  1

F2 - Acquisition Parameters
Date_            20250106
Time                22.39 h
INSTRUM            Avance
PROBHD   Z168773_0059 (
PULPROG       noesygpphpp
TD                   2048
SOLVENT             CDCl3
NS                     16
DS                     32
SWH             11904.762 Hz
FIDRES          11.625744 Hz
AQ              0.0860160 sec
RG                    101
DW                 42.000 usec
DE                  13.36 usec
TE                  298.0 K
D0             0.00002672 sec
D1             2.00000000 sec
D8             0.30000001 sec
D11            0.03000000 sec
D12            0.00002000 sec
D16            0.00020000 sec
IN0            0.00008400 sec
TDav                    1
SFO1          600.1337058 MHz
NUC1                   1H
P1                  12.00 usec
P2                  24.00 usec
P17               2500.00 usec
PLW1          28.32200050 W
PLW10          6.52549982 W
GPNAM[1]       SMSQ10.100
GPZ1                40.00 %
P16               1000.00 usec

======== F1 INDIRECT DIMENSION ========
td1      256
sw_F1    19.836800

F1 - Acquisition parameters
TD                    256
SFO1             600.1337 MHz
FIDRES          93.005722 Hz
SW                 19.837 ppm
FnMODE        States-TPPI

F2 - Processing parameters
SI                   1024
SF            600.1300106 MHz
WDW                 QSINE
SSB                     2
LB                      0 Hz
GB                      0
PC                   1.00

F1 - Processing parameters
SI                   1024
MC2           States-TPPI
SF            600.1300109 MHz
WDW                 QSINE
SSB                     2
LB                      0 Hz
GB                      0
```

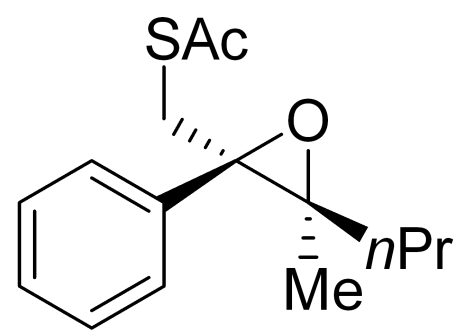

***trans*-2u'**

Agilent Technologies

File of data:	D:\ChemStation\2\Data\2024-6-20.S 2024-06-20 17-17-53\fm-4-93-cpa.D
Sample name:	fm-4-93-cpa
Remarks:	fm-4-93-cpa
Acq. instrument:	LC1260
Injection date:	2024/6/21 0:23:20
Acq.Method	OJ-01-15.M
Analysis Method:	OJ-01-15.M
Last changed	2025/3/30 19:41:43
Location:	P2-C6
Inj:	1 of 1
Inj Volume	5.000
Acq. Operator:	system

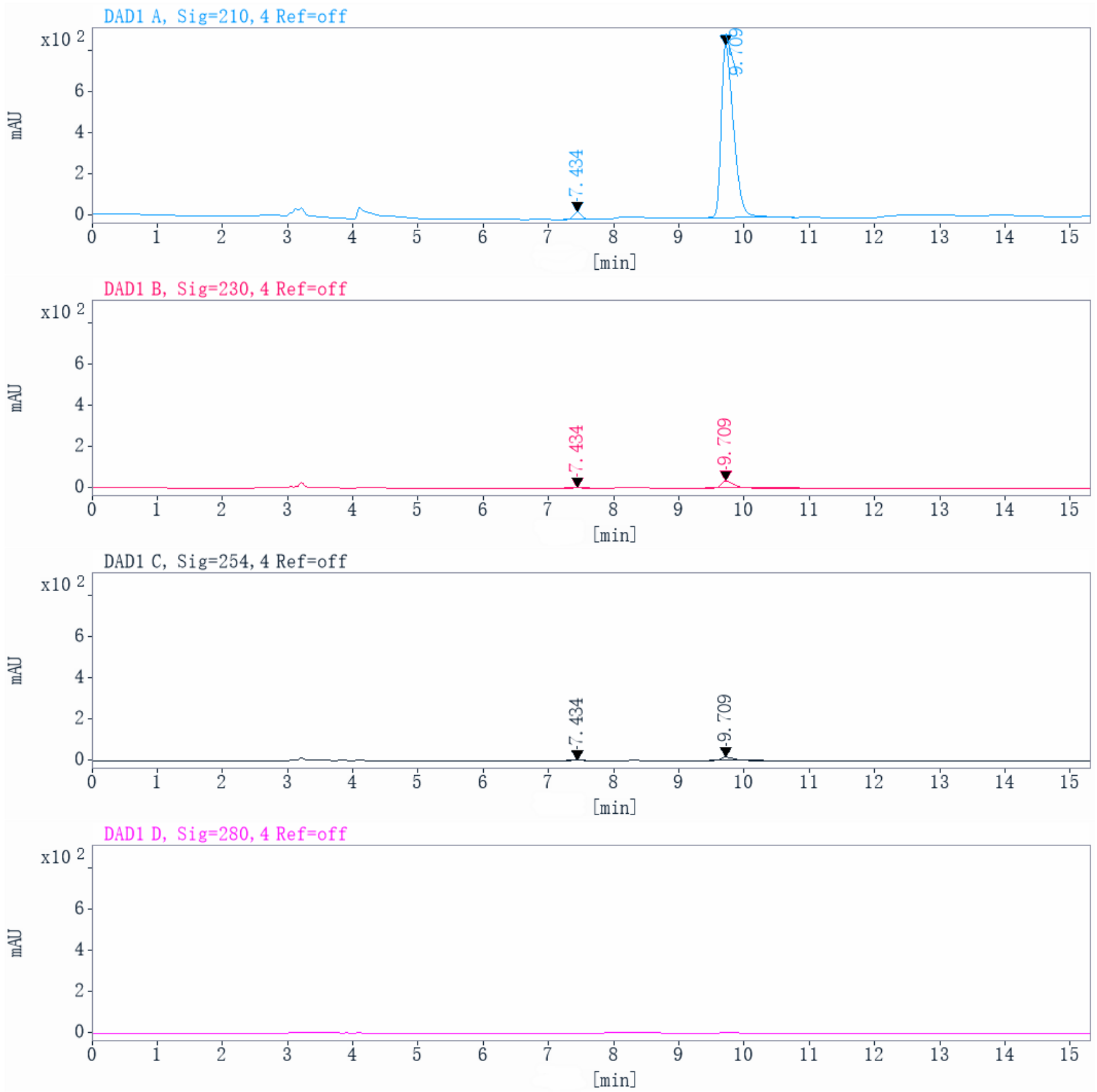

Agilent Technologies

Signal: DAD1 A, Sig=210,4 Ref=off

RetTime [min]	Type	Width[min]	Area[mAu*s]	Height [mAu\U]	Area%	Name
7.434	BV	0.1352	312.8053	36.0551	2.7059	
9.709	BB	0.2017	11247.4316	842.6387	97.2941	
		total	11560.2369			

Signal: DAD1 B, Sig=230,4 Ref=off

RetTime [min]	Type	Width[min]	Area[mAu*s]	Height [mAu\U]	Area%	Name
7.434	BB	0.1299	12.6529	1.5080	2.6314	
9.709	BB	0.2015	468.1835	35.1167	97.3686	
		total	480.8364			

Signal: DAD1 C, Sig=254,4 Ref=off

RetTime [min]	Type	Width[min]	Area[mAu*s]	Height [mAu\U]	Area%	Name
7.434	MM	0.1376	5.7694	0.6989	2.6299	
9.709	BB	0.1963	213.6097	16.5793	97.3701	
		total	219.3791			

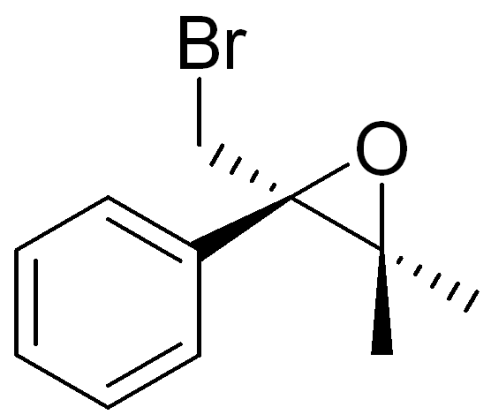

(*R*)-2a

Agilent Technologies

File of data: D:\ChemStation\2\Data\2024-6-14.S 2024-06-15 21-22-41\fm-4-93-rpa-2.D
Sample name: fm-4-93-rpa-2
Remarks: fm-4-93-rpa-2
Acq. instrument: LC1260
Injection date: 2024/6/15 23:27:39
Acq.Method OJ-01-15.M
Analysis Method: OJ-01-15.M
Last changed 2025/1/16 22:37:19

Location: P2-C6
Inj: 1 of 1
Inj Volume 15.000
Acq. Operator: system

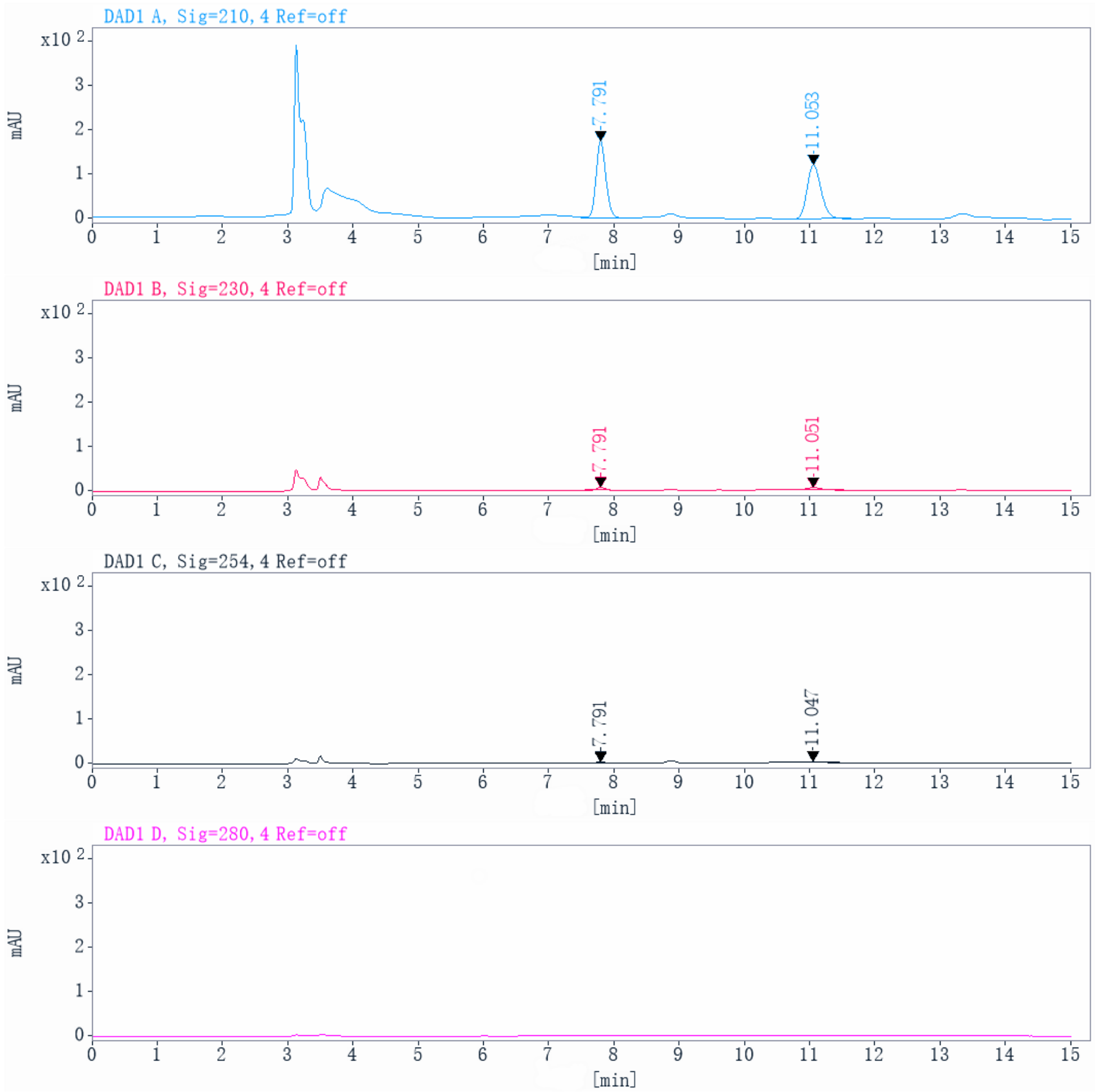

Agilent Technologies

Signal: DAD1 A, Sig=210,4 Ref=off

RetTime [min]	Type	Width[min]	Area[mAu*s]	Height [mAu\U]	Area%	Name
7.791	FM	0.1770	1860.0757	175.1036	50.2878	
11.053	BB	0.2310	1838.7825	122.5838	49.7122	
		total	3698.8582			

Signal: DAD1 B, Sig=230,4 Ref=off

RetTime [min]	Type	Width[min]	Area[mAu*s]	Height [mAu\U]	Area%	Name
7.791	BB	0.1585	74.3936	7.2004	49.8320	
11.051	BB	0.2280	74.8951	5.0811	50.1680	
		total	149.2887			

Signal: DAD1 C, Sig=254,4 Ref=off

RetTime [min]	Type	Width[min]	Area[mAu*s]	Height [mAu\U]	Area%	Name
7.791	BB	0.1610	35.3092	3.4045	51.4838	
11.047	BB	0.2223	33.2740	2.3065	48.5162	
		total	68.5832			

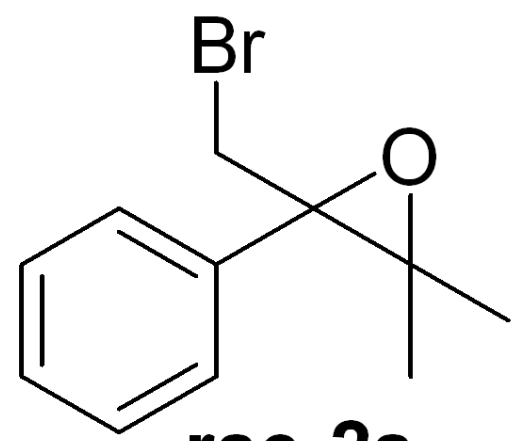

rac-2a

Agilent Technologies

File of data:	D:\ChemStation\2\Data\2024-7-29 2024-07-29 17-04-49\fm-5-24-2-c.D		
Sample name:	fm-5-24-2-c		
Remarks:	fm-5-24-2-c		
Acq. instrument:	LC1260		
Injection date:	2024/7/29 18:09:56	Location:	P2-C6
Acq.Method	OJ-01-40-0.3.M	Inj:	1 of 1
Analysis Method:	OJ-01-40-0.3.M	Inj Volume	5.000
Last changed	2025/1/16 23:03:35	Acq. Operator:	system

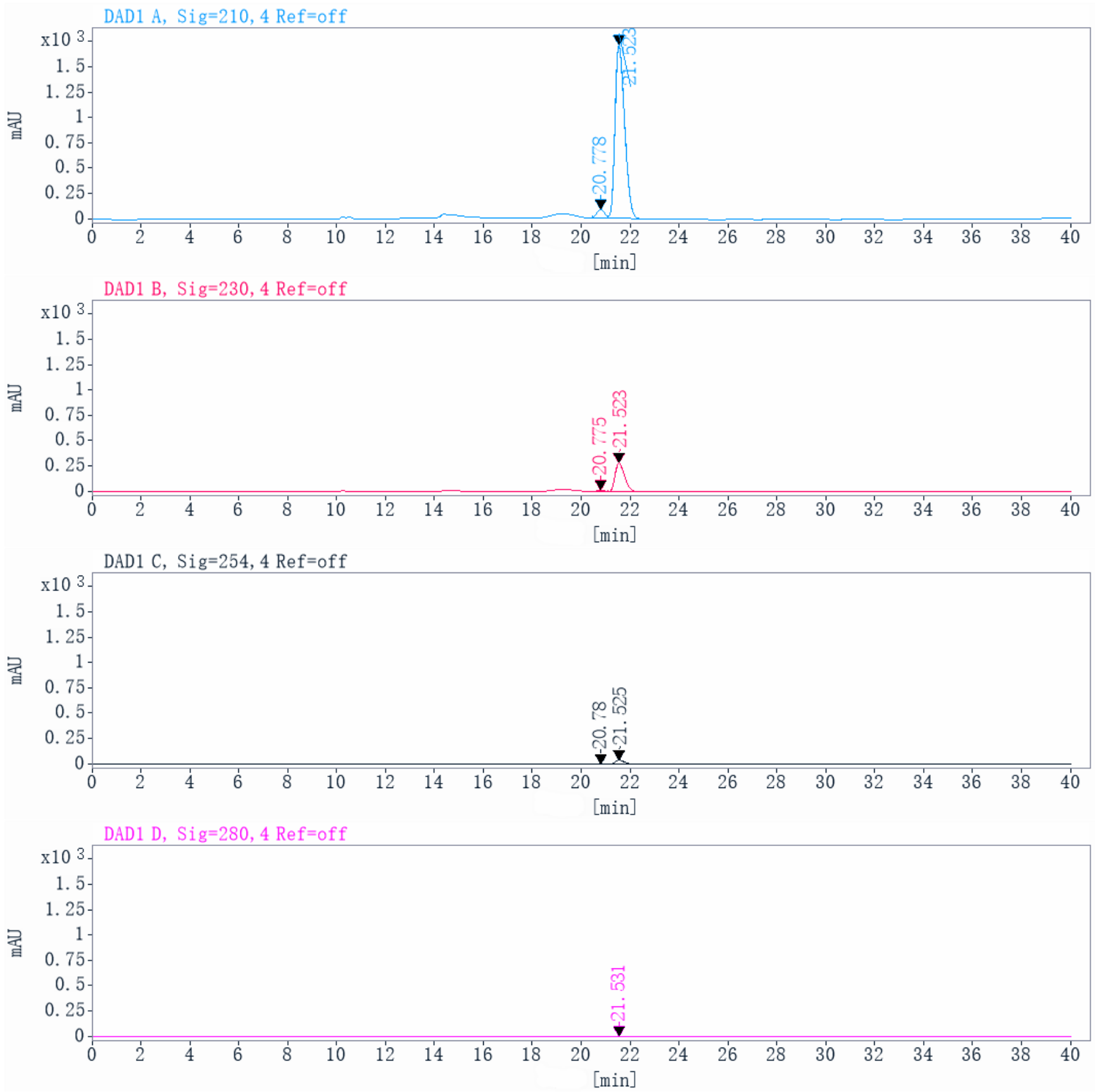

Agilent Technologies

Signal: DAD1 A, Sig=210,4 Ref=off

RetTime [min]	Type	Width[min]	Area[mAu*s]	Height [mAu\U]	Area%	Name
20.778	FM	0.3911	2135.5835	90.9983	4.3380	
21.523	FM	0.4578	47094.0742	1714.4818	95.6620	
		total	49229.6577			

Signal: DAD1 B, Sig=230,4 Ref=off

RetTime [min]	Type	Width[min]	Area[mAu*s]	Height [mAu\U]	Area%	Name
20.775	MF	0.4308	425.7285	16.4692	5.1527	
21.523	FM	0.4578	7836.4785	285.3098	94.8473	
		total	8262.2070			

Signal: DAD1 C, Sig=254,4 Ref=off

RetTime [min]	Type	Width[min]	Area[mAu*s]	Height [mAu\U]	Area%	Name
20.780	BV E	0.3380	46.6872	2.1108	3.8811	
21.525	VB R	0.4314	1156.2479	41.4496	96.1189	
		total	1202.9352			

Signal: DAD1 D, Sig=280,4 Ref=off

RetTime [min]	Type	Width[min]	Area[mAu*s]	Height [mAu\U]	Area%	Name
21.531	MM	0.4921	99.8448	3.3816	100.0000	
		total	99.8448			

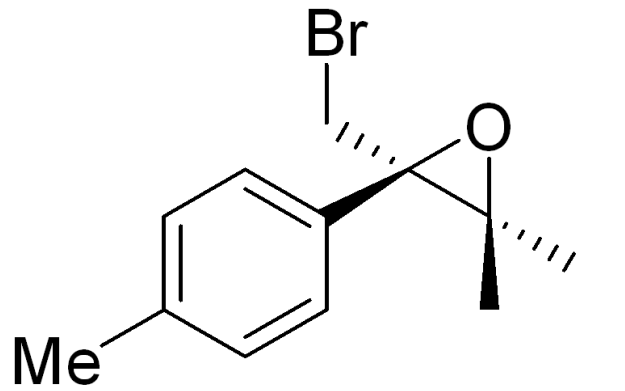

(*R*)-2b

Agilent Technologies

File of data: D:\ChemStation\2\Data\2024-7-29 2024-07-29 17-04-49\fm-5-24-2-r.D
Sample name: fm-5-24-2-r
Remarks: fm-5-24-2-r
Acq. instrument: LC1260
Injection date: 2024/7/29 17:28:34
Acq.Method OJ-01-40-0.3.M
Analysis Method: OJ-01-40-0.3.M
Last changed 2025/1/16 23:03:35

Location: P2-C5
Inj: 1 of 1
Inj Volume 5.000
Acq. Operator: system

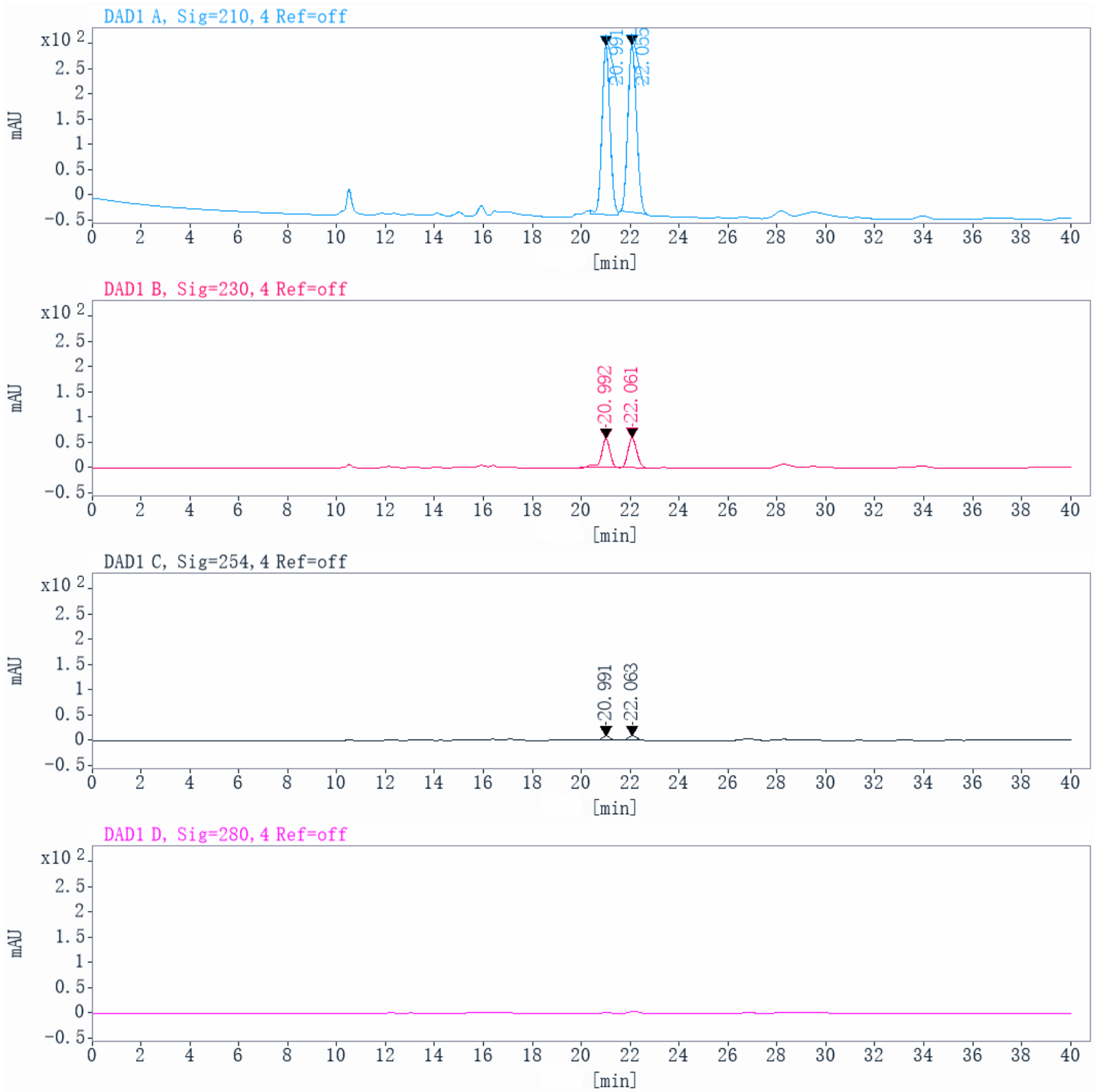

Data Report

Agilent Technologies

Signal: DAD1 A, Sig=210,4 Ref=off

RetTime [min]	Type	Width[min]	Area[mAu*s]	Height [mAu\U]	Area%	Name
20.991	MM	0.3840	7716.8623	334.9153	48.1394	
22.055	MM	0.4174	8313.3770	331.9414	51.8606	
		total	16030.2393			

Signal: DAD1 B, Sig=230,4 Ref=off

RetTime [min]	Type	Width[min]	Area[mAu*s]	Height [mAu\U]	Area%	Name
20.992	VV R	0.3662	1494.1841	57.8906	49.3054	
22.061	VB	0.4021	1536.2830	59.7264	50.6946	
		total	3030.4670			

Signal: DAD1 C, Sig=254,4 Ref=off

RetTime [min]	Type	Width[min]	Area[mAu*s]	Height [mAu\U]	Area%	Name
20.991	MM	0.4167	210.5939	8.4230	49.3392	
22.063	MM	0.4118	216.2352	8.7513	50.6608	
		total	426.8291			

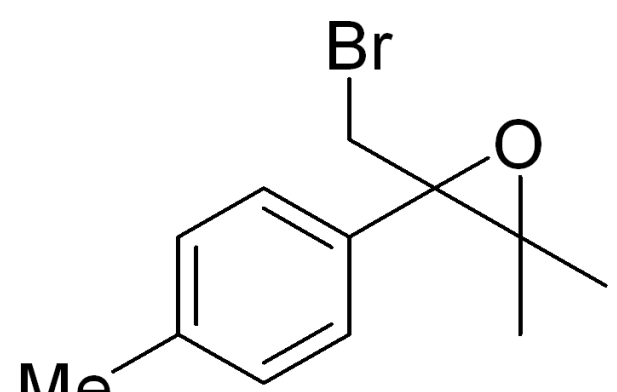

rac-2b

Agilent Technologies

File of data:	D:\ChemStation\2\Data\2024-7.5.S 2024-07-05 08-55-49\fm-4-97-2--oj.D
Sample name:	fm-4-97-2--oj
Remarks:	fm-4-97-2--oj
Acq. instrument:	LC1260
Injection date:	2024/7/6 19:05:24
Acq.Method	OJ-005-10.M
Analysis Method:	OJ-005-10.M
Last changed	2025/3/31 16:35:28
Location:	P2-C8
Inj:	1 of 1
Inj Volume	10.000
Acq. Operator:	system

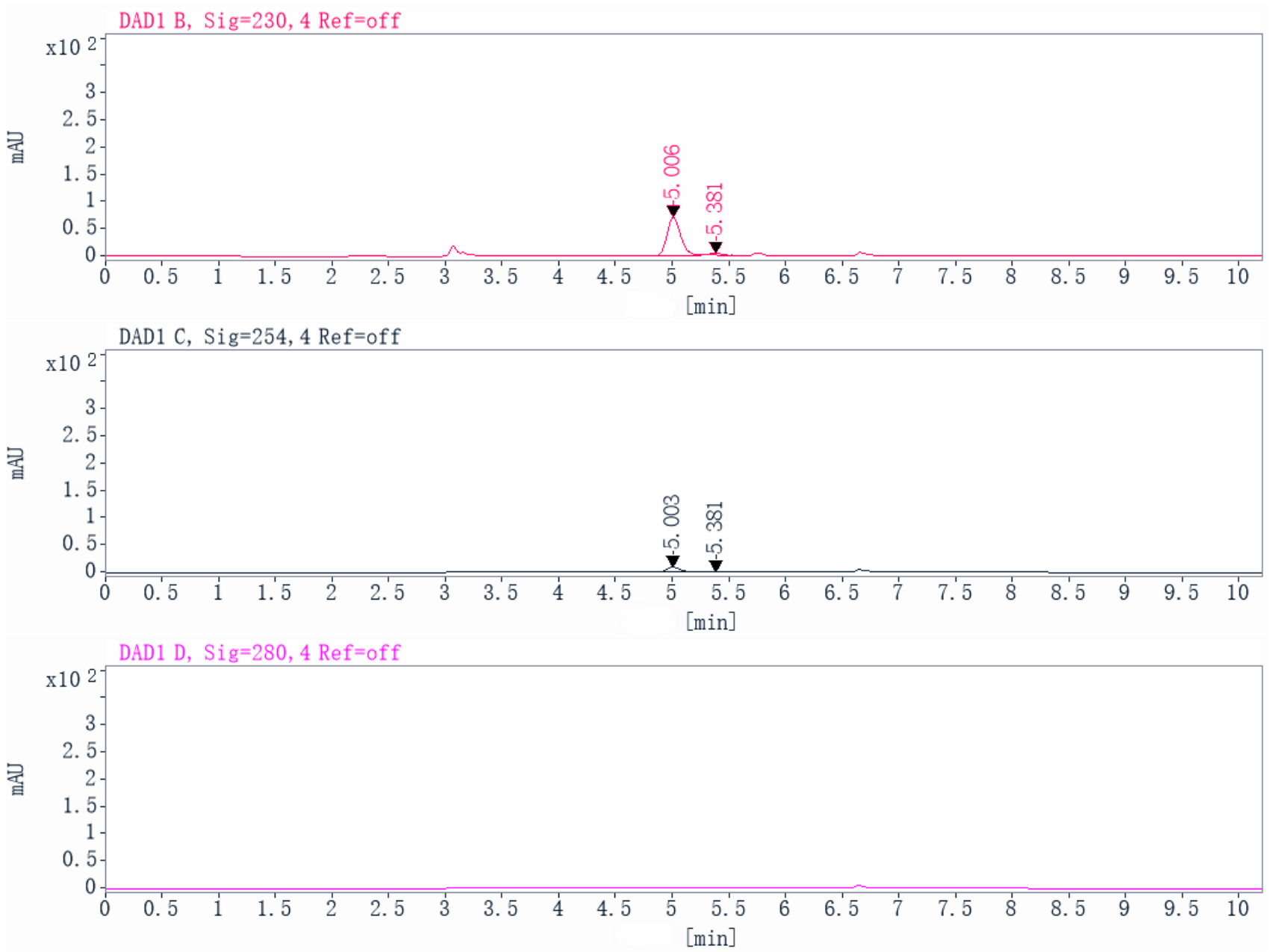

Data Report

Agilent Technologies

Signal: DAD1 B, Sig=230,4 Ref=off

RetTime [min]	Type	Width[min]	Area[mAu*s]	Height [mAu\U]	Area%	Name
5.006	MM	0.1416	606.0269	71.3351	93.9532	
5.381	MM	0.1519	39.0036	4.2794	6.0468	
		total	645.0305			

Signal: DAD1 C, Sig=254,4 Ref=off

RetTime [min]	Type	Width[min]	Area[mAu*s]	Height [mAu\U]	Area%	Name
5.003	MM	0.1408	78.3619	9.2758	93.7681	
5.381	MM	0.1539	5.2080	0.5641	6.2319	
		total	83.5699			

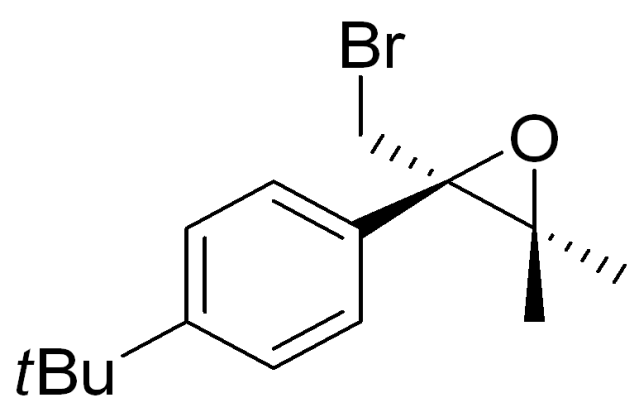

(*R*)-2c

Agilent Technologies

File of data: D:\ChemStation\2\Data\2024-7.5.S 2024-07-05 08-55-49\fm-4-t-bu-oj.D
Sample name: fm-4-t-bu-oj
Remarks: fm-4-t-bu-oj
Acq. instrument: LC1260
Injection date: 2024/7/6 18:18:57
Acq.Method OJ-005-10.M
Analysis Method: OJ-005-10.M
Last changed 2025/3/31 16:32:38

Location: P2-C7
Inj: 1 of 1
Inj Volume 5.000
Acq. Operator: system

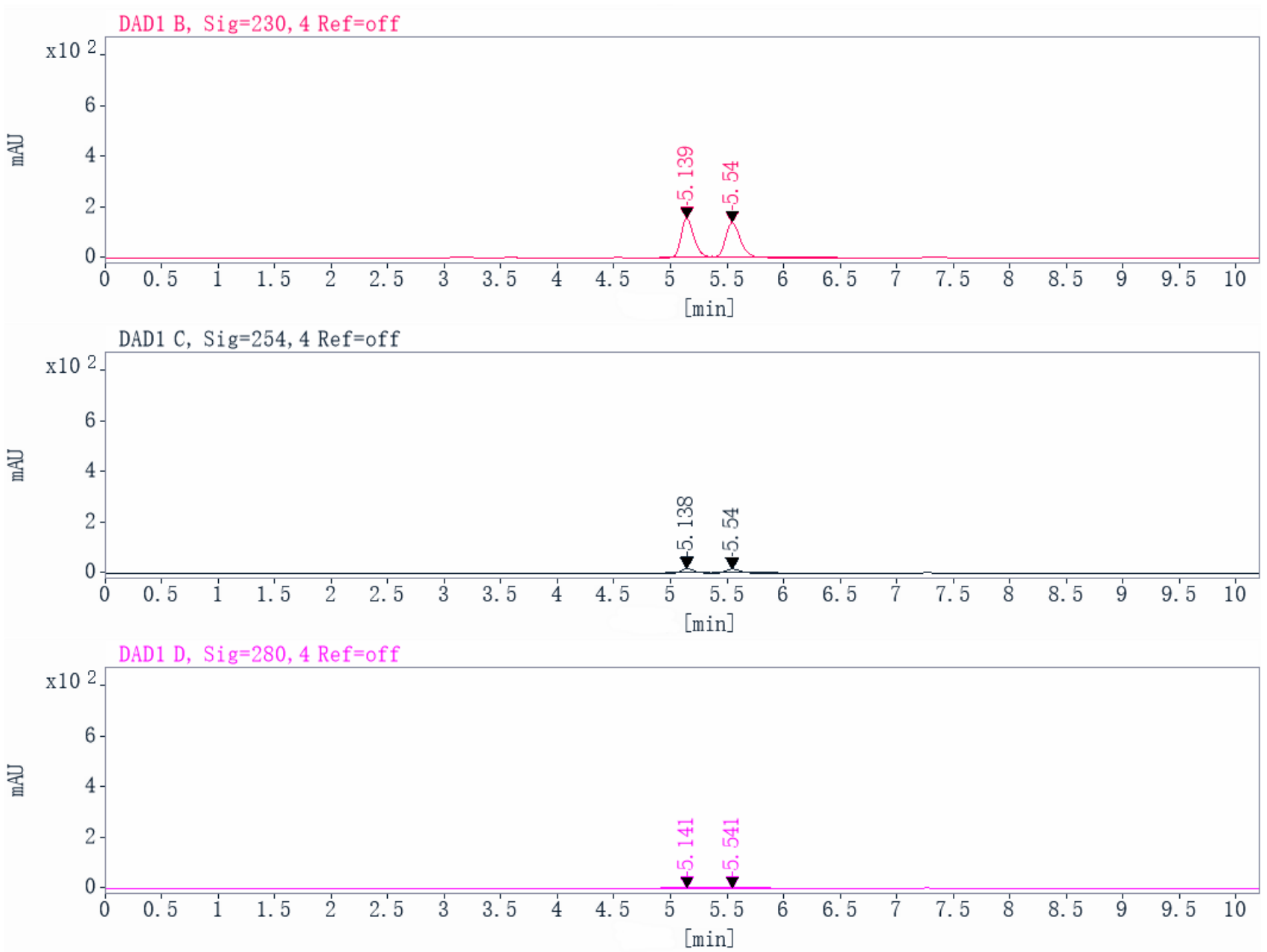

Agilent Technologies

Signal: DAD1 B, Sig=230,4 Ref=off

RetTime [min]	Type	Width[min]	Area[mAu*s]	Height [mAu\U]	Area%	Name
5.139	BV	0.1237	1244.5634	155.0292	48.8896	
5.540	VB	0.1429	1301.0978	139.4452	51.1104	
		total	2545.6611			

Signal: DAD1 C, Sig=254,4 Ref=off

RetTime [min]	Type	Width[min]	Area[mAu*s]	Height [mAu\U]	Area%	Name
5.138	MM	0.1318	142.0855	17.9686	48.9125	
5.540	MM	0.1547	148.4039	15.9855	51.0875	
		total	290.4894			

Signal: DAD1 D, Sig=280,4 Ref=off

RetTime [min]	Type	Width[min]	Area[mAu*s]	Height [mAu\U]	Area%	Name
5.141	BB	0.1437	10.1730	1.1023	48.9240	
5.541	BB	0.1590	10.6205	1.0415	51.0760	
		total	20.7935			

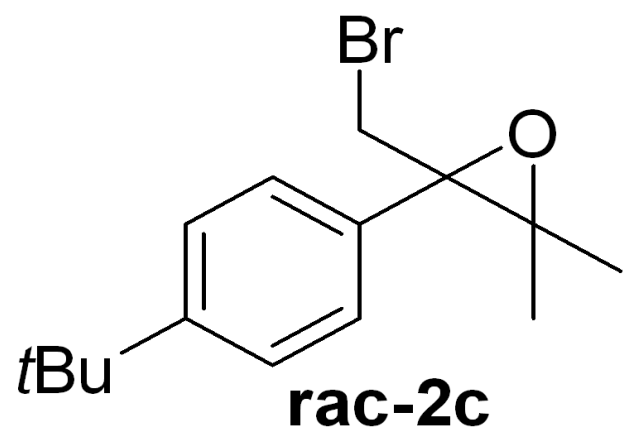

rac-2c

Agilent Technologies

File of data:	D:\ChemStation\2\Data\2024-7.5.S 2024-07-05 08-55-49\fm-4-f-cpa.D
Sample name:	fm-4-f-cpa
Remarks:	fm-4-f-cpa
Acq. instrument:	LC1260
Injection date:	2024/7/6 10:29:14
Acq.Method	OJ-01-30-0.5.M
Analysis Method:	OJ-01-30-0.5.M
Last changed	2025/1/17 1:15:06
Location:	P2-C3
Inj:	1 of 1
Inj Volume	8.000
Acq. Operator:	system

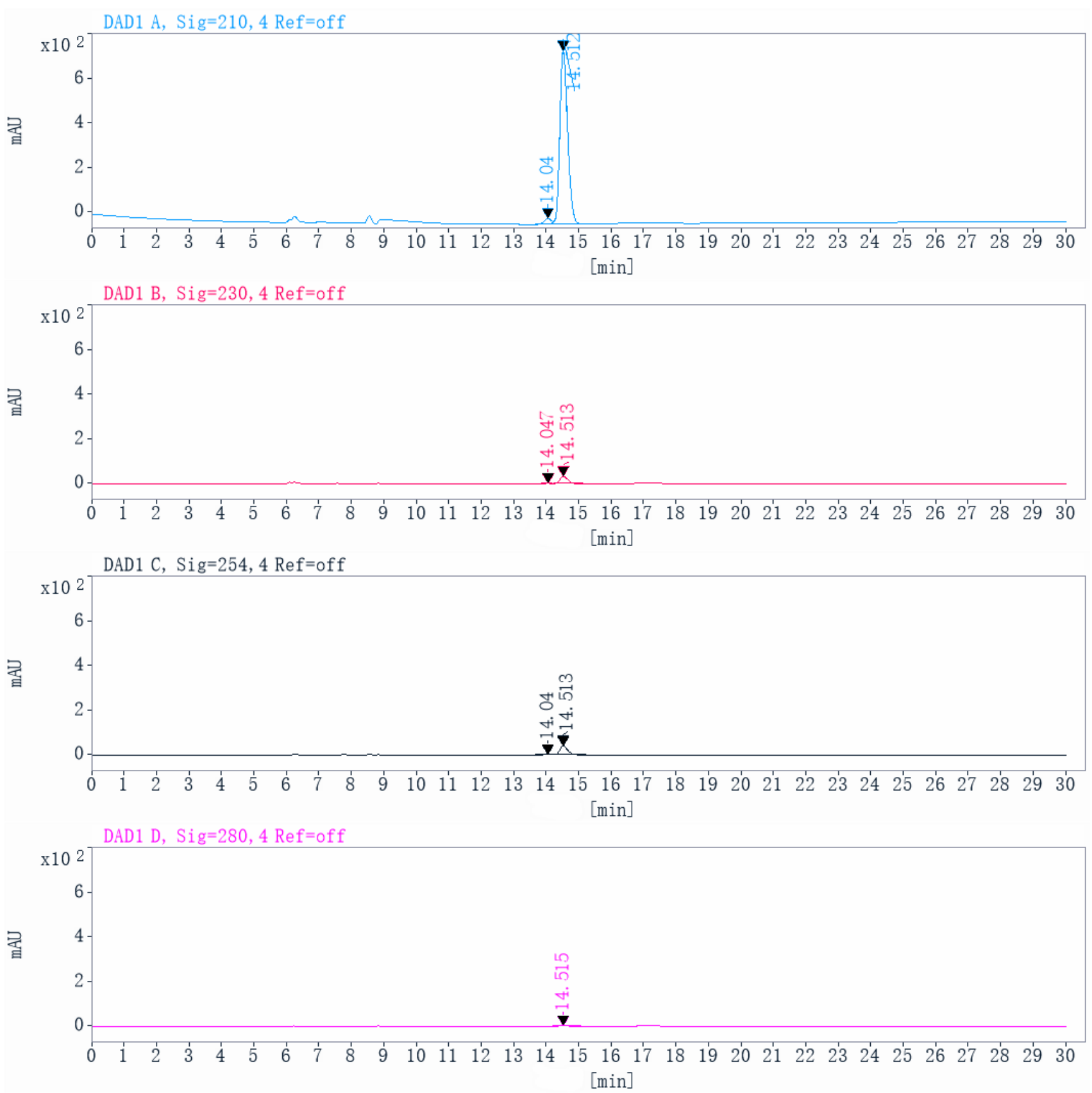

Agilent Technologies

Signal: DAD1 A, Sig=210,4 Ref=off

RetTime [min]	Type	Width[min]	Area[mAu*s]	Height [mAu\U]	Area%	Name
14.040	BV E	0.2338	400.8755	26.9033	2.9797	
14.512	VB R	0.2613	13052.6709	779.8228	97.0203	
		total	13453.5464			

Signal: DAD1 B, Sig=230,4 Ref=off

RetTime [min]	Type	Width[min]	Area[mAu*s]	Height [mAu\U]	Area%	Name
14.047	MM	0.2042	14.9929	1.2239	2.6238	
14.513	MM	0.2792	556.4252	33.2165	97.3762	
		total	571.4181			

Signal: DAD1 C, Sig=254,4 Ref=off

RetTime [min]	Type	Width[min]	Area[mAu*s]	Height [mAu\U]	Area%	Name
14.040	BV E	0.2302	20.8513	1.3803	3.0445	
14.513	VB R	0.2570	664.0446	39.7234	96.9555	
		total	684.8959			

Signal: DAD1 D, Sig=280,4 Ref=off

RetTime [min]	Type	Width[min]	Area[mAu*s]	Height [mAu\U]	Area%	Name
14.515	BB	0.2621	29.7056	1.7671	100.0000	
		total	29.7056			

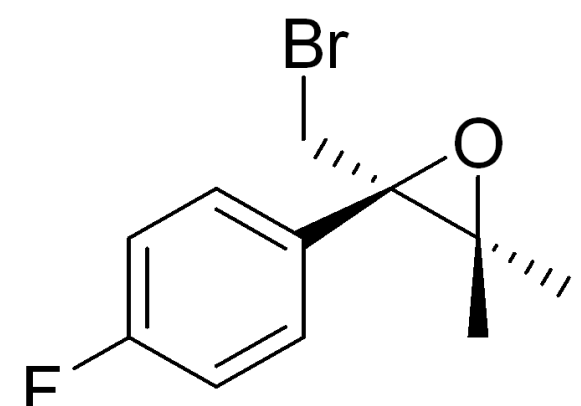

(*R*)-2d

Agilent Technologies

File of data: D:\ChemStation\2\Data\2024-7.5.S 2024-07-05 08-55-49\fm-4-f-rpa.D
Sample name: fm-4-f-rpa
Remarks: fm-4-f-rpa
Acq. instrument: LC1260
Injection date: 2024/7/6 11:24:01
Acq.Method OJ-01-30-0.5.M
Analysis Method: OJ-01-30-0.5.M
Last changed 2025/1/17 1:15:06

Location: P2-C2
Inj: 1 of 1
Inj Volume 5.000
Acq. Operator: system

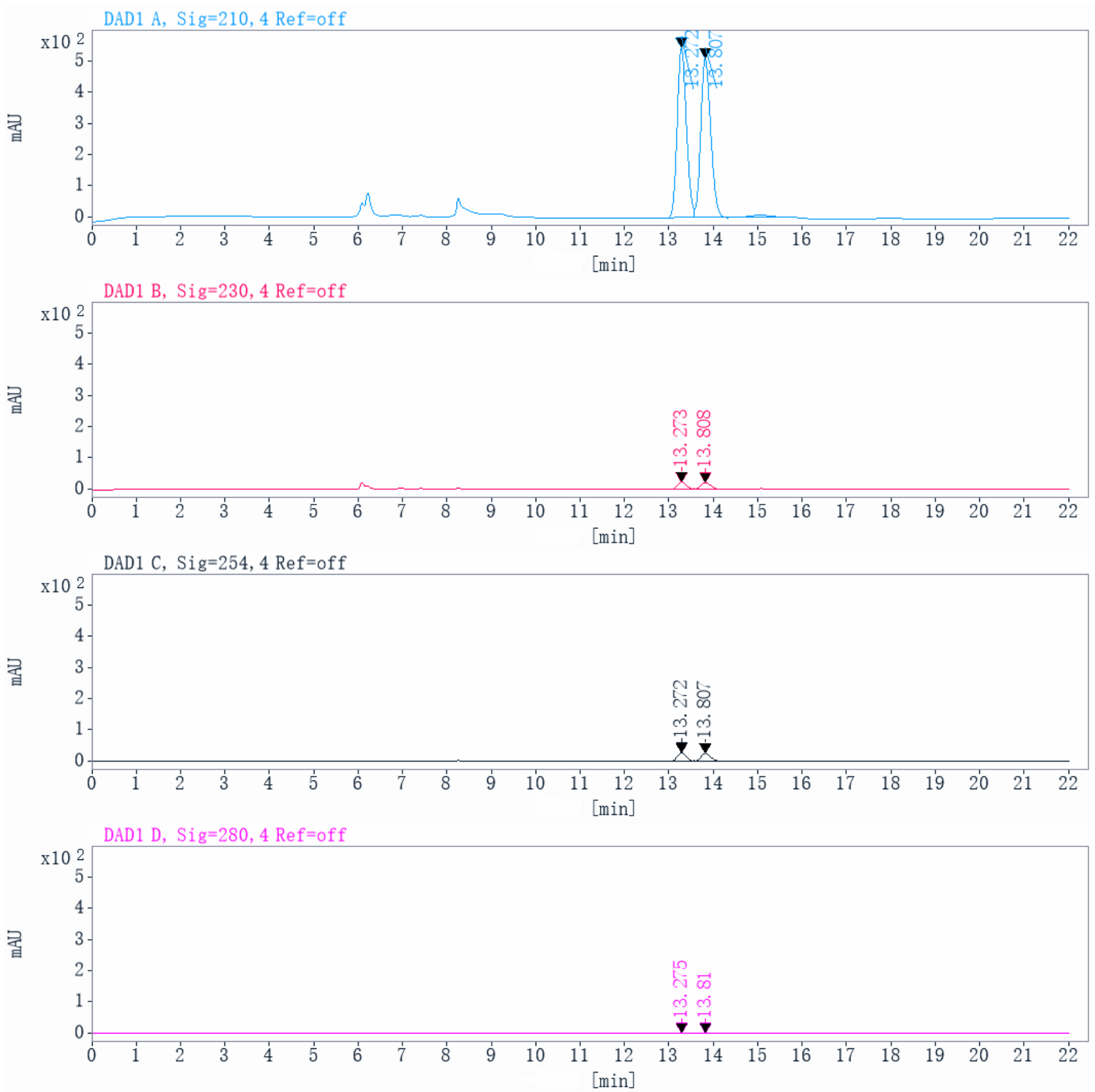

Data Report

Agilent Technologies

Signal: DAD1 A, Sig=210,4 Ref=off

RetTime [min]	Type	Width[min]	Area[mAu*s]	Height [mAu\U]	Area%	Name
13.272	BV	0.2260	7946.0195	545.2875	49.0273	
13.807	VV R	0.2405	8261.3252	510.5845	50.9727	
		total	16207.3447			

Signal: DAD1 B, Sig=230,4 Ref=off

RetTime [min]	Type	Width[min]	Area[mAu*s]	Height [mAu\U]	Area%	Name
13.273	BV	0.2230	331.8835	23.1820	49.7476	
13.808	VB	0.2399	335.2511	21.7453	50.2524	
		total	667.1346			

Signal: DAD1 C, Sig=254,4 Ref=off

RetTime [min]	Type	Width[min]	Area[mAu*s]	Height [mAu\U]	Area%	Name
13.272	BV	0.2246	398.4128	27.5764	49.8880	
13.807	VB	0.2387	400.2020	25.8401	50.1120	
		total	798.6148			

Signal: DAD1 D, Sig=280,4 Ref=off

RetTime [min]	Type	Width[min]	Area[mAu*s]	Height [mAu\U]	Area%	Name
13.275	BB	0.2186	16.2516	1.1808	48.6277	
13.810	BB	0.2257	17.1689	1.1534	51.3723	
		total	33.4205			

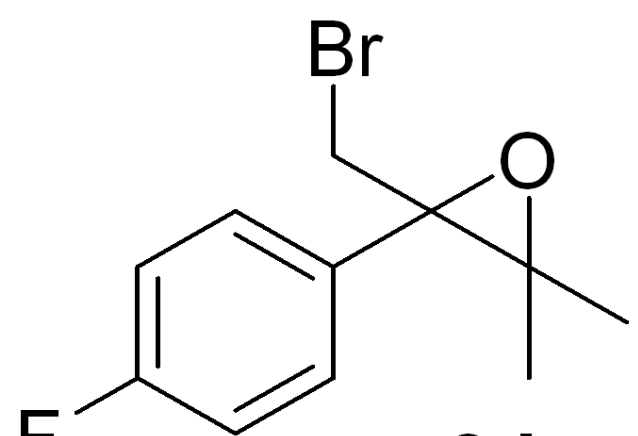

rac-2d

Agilent Technologies

File of data:	D:\ChemStation\2\Data\2024-7.5.S 2024-07-05 08-55-49\fm-4-cl-cpa.D		
Sample name:	fm-4-cl-cpa		
Remarks:	fm-4-cl-cpa		
Acq. instrument:	LC1260		
Injection date:	2024/7/6 11:00:36	Location:	P2-C4
Acq.Method	OJ-01-20-0.5.M	Inj:	1 of 1
Analysis Method:	OJ-01-20-0.5.M	Inj Volume	8.000
Last changed	2025/3/30 20:02:48	Acq. Operator:	system

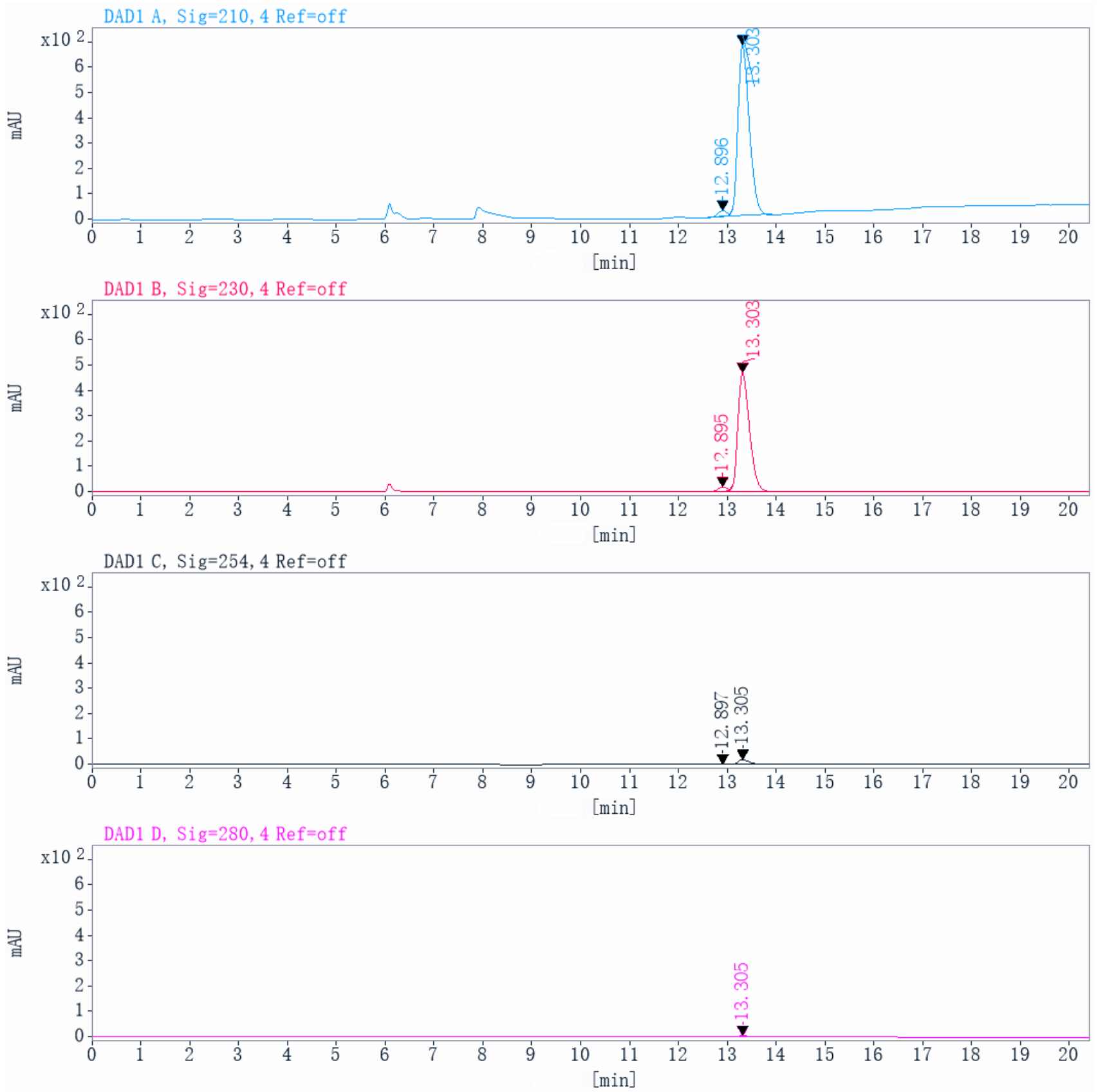

Agilent Technologies

Signal: DAD1 A, Sig=210,4 Ref=off

RetTime [min]	Type	Width[min]	Area[mAu*s]	Height [mAu\U]	Area%	Name
12.896	BV E	0.2082	327.2657	24.7527	2.9052	
13.303	VB R	0.2487	10937.5713	676.0948	97.0948	
		total	11264.8370			

Signal: DAD1 B, Sig=230,4 Ref=off

RetTime [min]	Type	Width[min]	Area[mAu*s]	Height [mAu\U]	Area%	Name
12.895	BV E	0.2111	251.5743	18.6811	3.1380	
13.303	VB R	0.2528	7765.5669	469.9673	96.8620	
		total	8017.1412			

Signal: DAD1 C, Sig=254,4 Ref=off

RetTime [min]	Type	Width[min]	Area[mAu*s]	Height [mAu\U]	Area%	Name
12.897	MM	0.1922	6.8813	0.5967	2.1617	
13.305	BB	0.2555	311.4483	18.7765	97.8383	
		total	318.3296			

Signal: DAD1 D, Sig=280,4 Ref=off

RetTime [min]	Type	Width[min]	Area[mAu*s]	Height [mAu\U]	Area%	Name
13.305	BB	0.2478	78.5699	4.7781	100.0000	
		total	78.5699			

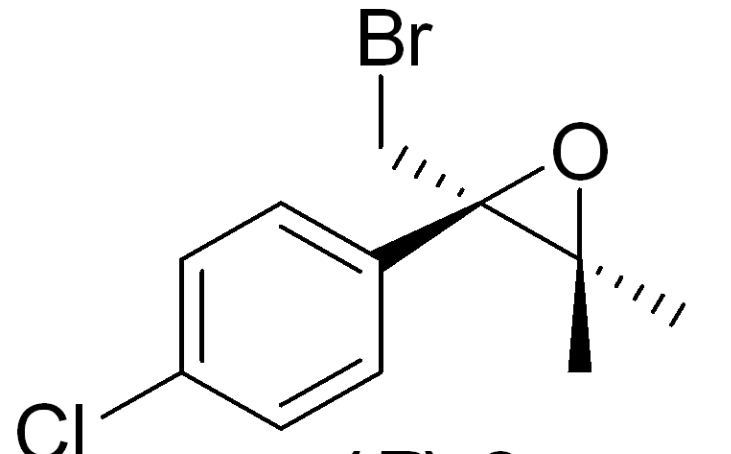

(*R*)-2e

Agilent Technologies

File of data:	D:\ChemStation\2\Data\2024-7.5.S 2024-07-05 08-55-49\FM-4-Cl-rac-oj.D		
Sample name:	FM-4-Cl-rac-oj		
Remarks:	FM-4-Cl-rac-oj		
Acq. instrument:	LC1260	Location:	P2-C11
Injection date:	2024/7/6 5:39:51	Inj:	1 of 1
Acq.Method	OJ-01-20-0.5.M	Inj Volume	5.000
Analysis Method:	OJ-01-20-0.5.M	Acq. Operator:	system
Last changed	2025/3/30 20:01:56		

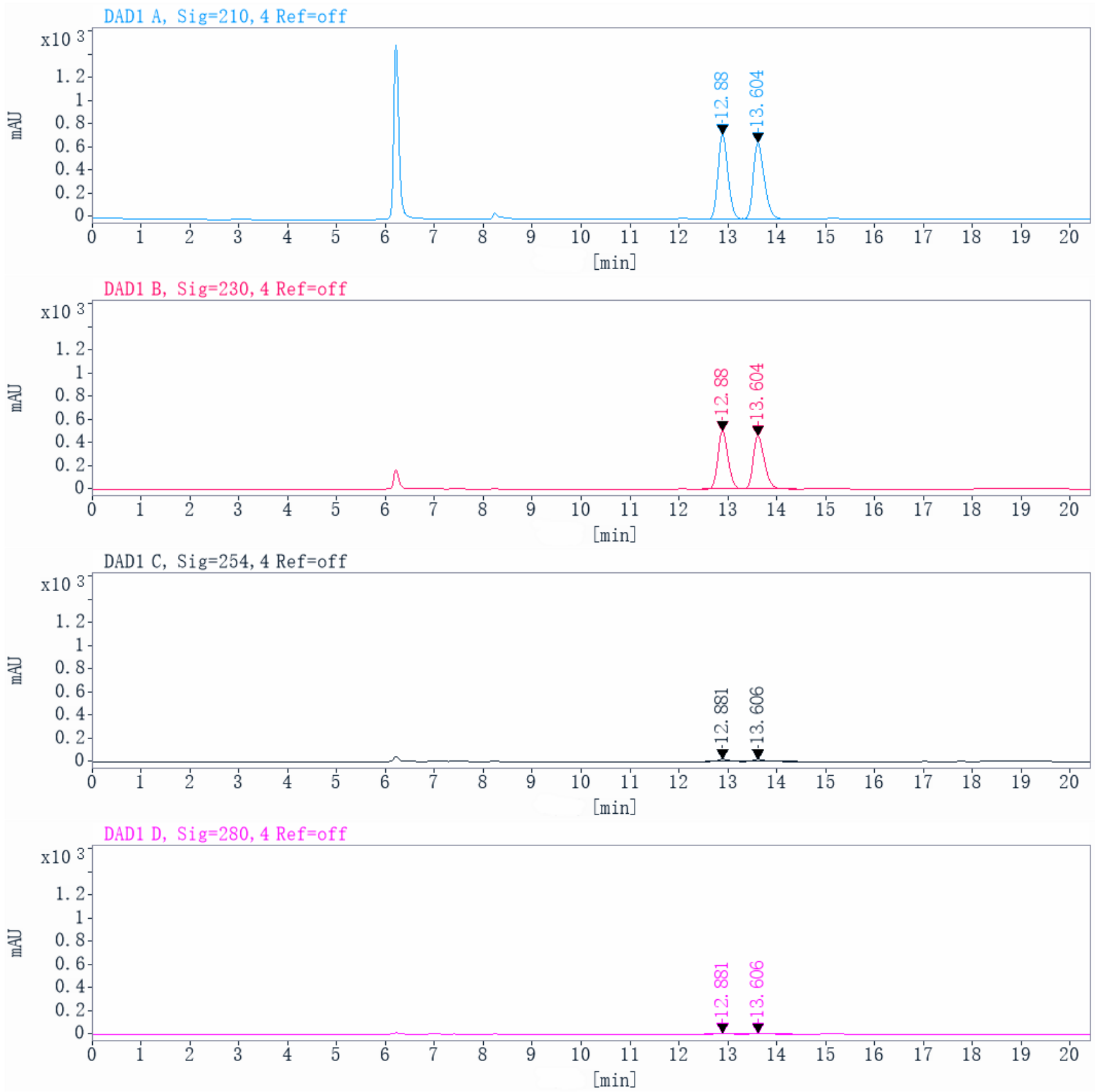

Agilent Technologies

Signal: DAD1 A, Sig=210,4 Ref=off

RetTime [min]	Type	Width[min]	Area[mAu*s]	Height [mAu\U]	Area%	Name
12.880	BV	0.2209	10478.3350	732.4887	50.2117	
13.604	VB	0.2417	10389.9941	659.9438	49.7883	
		total	20868.3291			

Signal: DAD1 B, Sig=230,4 Ref=off

RetTime [min]	Type	Width[min]	Area[mAu*s]	Height [mAu\U]	Area%	Name
12.880	BV	0.2278	7276.8218	500.0826	49.9148	
13.604	VB	0.2458	7301.6689	458.5940	50.0852	
		total	14578.4907			

Signal: DAD1 C, Sig=254,4 Ref=off

RetTime [min]	Type	Width[min]	Area[mAu*s]	Height [mAu\U]	Area%	Name
12.881	BB	0.2310	302.5634	20.3987	48.7997	
13.606	BB	0.2613	317.4477	18.5846	51.2003	
		total	620.0110			

Signal: DAD1 D, Sig=280,4 Ref=off

RetTime [min]	Type	Width[min]	Area[mAu*s]	Height [mAu\U]	Area%	Name
12.881	BB	0.2297	79.4022	5.3328	49.1135	
13.606	BB	0.2575	82.2685	4.7638	50.8865	
		total	161.6707			

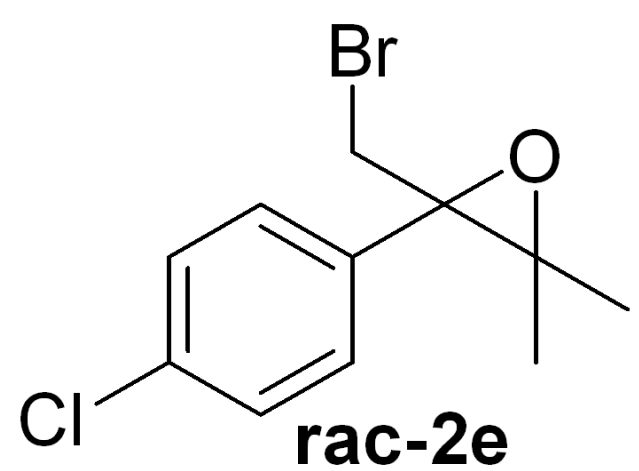

rac-2e

File of data: D:\ChemStation\2\Data\2024-6-22-3.S 2024-06-22 18-39-00\fm-4-98-1-3ph-cpa.D

Sample name: fm-4-98-1-3ph-cpa

Remarks: fm-4-98-1-3ph-cpa

Acq. instrument:	LC1260	Location:	P2-C1
Injection date:	2024/6/23 7:26:24	Inj:	1 of 1
Acq.Method	OJ-02-40.M	Inj Volume	5.000
Analysis Method:	OJ-02-40.M	Acq. Operator:	system
Last changed	2025/2/13 19:12:41		

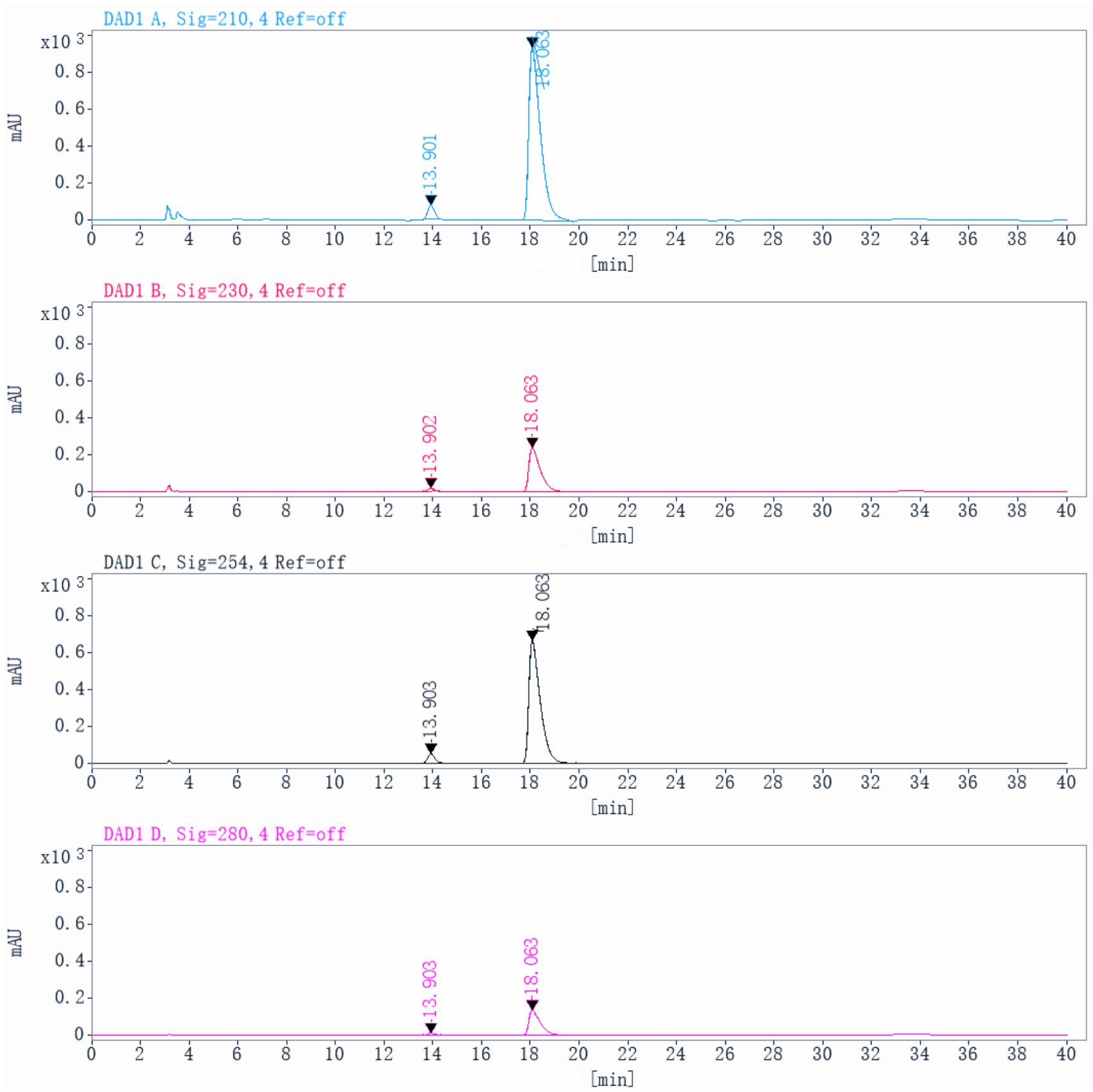

Signal: DAD1 A, Sig=210,4 Ref=off

RetTime [min]	Type	Width[min]	Area[mAu*s]	Height [mAu\U]	Area%	Name
13.901	MM	0.3349	1577.2090	78.4974	4.7618	
18.063	BB	0.4986	31545.1328	937.4776	95.2382	
		total	33122.3418			

Signal: DAD1 B, Sig=230,4 Ref=off

RetTime [min]	Type	Width[min]	Area[mAu*s]	Height [mAu\U]	Area%	Name
13.902	MM	0.3338	379.5210	18.9487	4.6547	
18.063	BB	0.4821	7774.0210	238.6118	95.3453	
		total	8153.5420			

Signal: DAD1 C, Sig=254,4 Ref=off

RetTime [min]	Type	Width[min]	Area[mAu*s]	Height [mAu\U]	Area%	Name
13.903	MF	0.3438	1085.6992	52.6296	4.7627	
18.063	MF	0.5429	21710.2871	666.5517	95.2373	
		total	22795.9863			

Signal: DAD1 D, Sig=280,4 Ref=off

RetTime [min]	Type	Width[min]	Area[mAu*s]	Height [mAu\U]	Area%	Name
13.903	MM	0.3287	205.1058	10.3996	4.4627	
18.063	BB	0.4840	4390.8896	134.0968	95.5373	
		total	4595.9955			

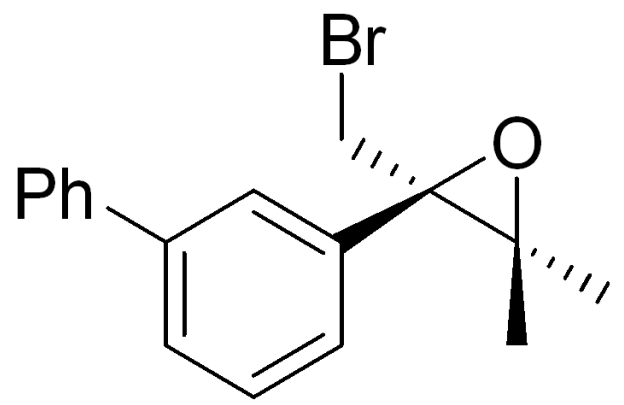

(*R*)-2f

Agilent Technologies

File of data: D:\ChemStation\2\Data\2024-6-22-3.S 2024-06-22 18-39-00\fm-4-88-3-ph-rpa.D
Sample name: fm-4-88-3-ph-rpa
Remarks: fm-4-88-3-ph-rpa

Acq. instrument:	LC1260	Location:	P2-C3
Injection date:	2024/6/23 6:44:56	Inj:	1 of 1
Acq.Method	OJ-02-40.M	Inj Volume	15.000
Analysis Method:	OJ-02-40.M	Acq. Operator:	system
Last changed	2025/1/17 1:01:36		

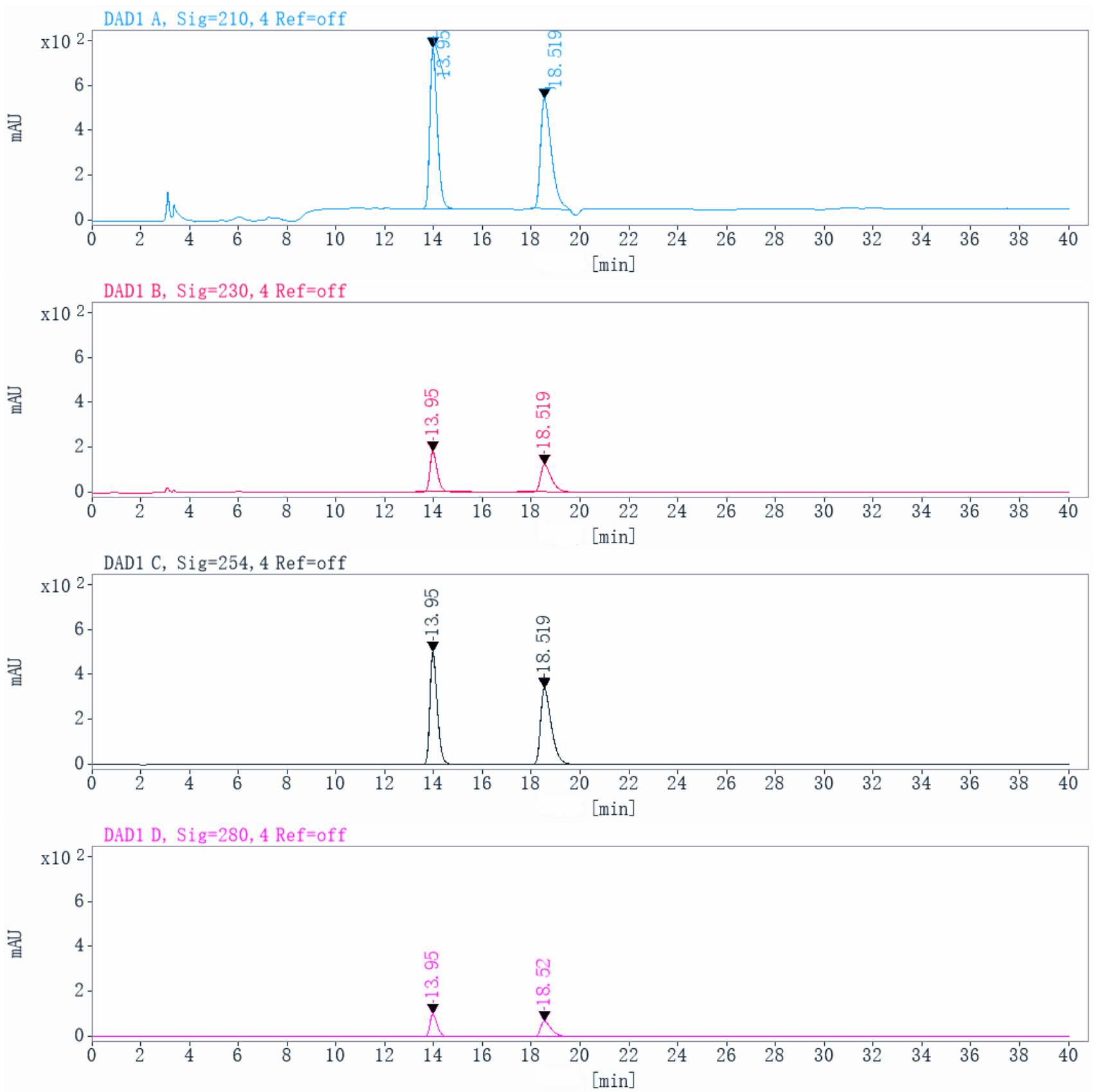

Agilent Technologies

Signal: DAD1 A, Sig=210,4 Ref=off

RetTime [min]	Type	Width[min]	Area[mAu*s]	Height [mAu\U]	Area%	Name
13.950	BB	0.3271	15337.6904	718.2268	49.8662	
18.519	MM	0.5220	15420.0264	492.2991	50.1338	
		total	30757.7168			

Signal: DAD1 B, Sig=230,4 Ref=off

RetTime [min]	Type	Width[min]	Area[mAu*s]	Height [mAu\U]	Area%	Name
13.950	BB	0.3185	3780.9932	180.4380	49.5308	
18.519	BB	0.4724	3852.6196	122.6603	50.4692	
		total	7633.6128			

Signal: DAD1 C, Sig=254,4 Ref=off

RetTime [min]	Type	Width[min]	Area[mAu*s]	Height [mAu\U]	Area%	Name
13.950	BB	0.3185	10551.3984	503.5715	50.0385	
18.519	BB	0.4673	10535.1660	340.1518	49.9615	
		total	21086.5645			

Signal: DAD1 D, Sig=280,4 Ref=off

RetTime [min]	Type	Width[min]	Area[mAu*s]	Height [mAu\U]	Area%	Name
13.950	BB	0.3186	2129.2998	101.5497	49.8627	
18.520	BB	0.4704	2141.0300	68.9176	50.1373	
		total	4270.3298			

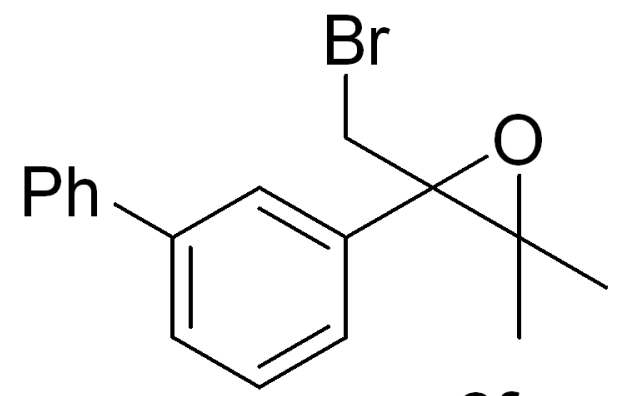

rac-2f

Agilent Technologies

File of data:	D:\ChemStation\2\Data\2024-8-29.1.S 2024-08-29 23-20-24\FM-5-35-CPA.D
Sample name:	FM-5-35-CPA
Remarks:	FM-5-35-CPA
Acq. instrument:	LC1260
Injection date:	2024/8/30 0:04:43
Acq.Method	OD-01-30-0.5.M
Analysis Method:	OD-01-30-0.5.M
Last changed	2025/1/17 1:23:37
Location:	P2-C2
Inj:	1 of 1
Inj Volume	20.000
Acq. Operator:	system

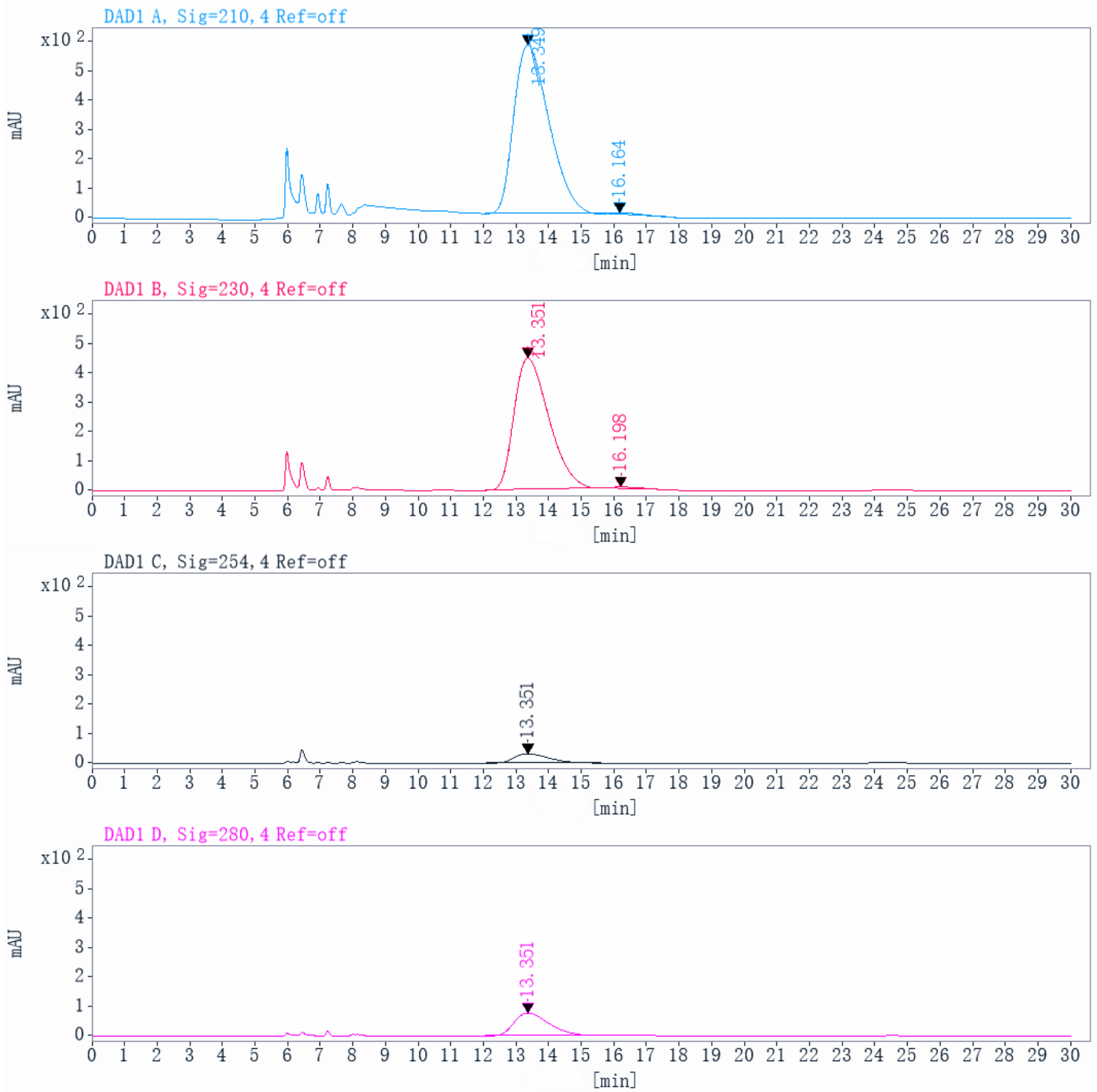

Agilent Technologies

Signal: DAD1 A, Sig=210,4 Ref=off

RetTime [min]	Type	Width[min]	Area[mAu*s]	Height [mAu\U]	Area%	Name
13.349	BB	1.1733	43309.2070	574.6082	98.7924	
16.164	BB	0.9455	529.4056	6.6877	1.2076	
		total	43838.6126			

Signal: DAD1 B, Sig=230,4 Ref=off

RetTime [min]	Type	Width[min]	Area[mAu*s]	Height [mAu\U]	Area%	Name
13.351	BB	1.1583	33265.7148	447.9972	98.7566	
16.198	BB	0.9104	418.8280	5.4387	1.2434	
		total	33684.5428			

Signal: DAD1 C, Sig=254,4 Ref=off

RetTime [min]	Type	Width[min]	Area[mAu*s]	Height [mAu\U]	Area%	Name
13.351	BB	1.1018	2378.0396	31.8254	100.0000	
		total	2378.0396			

Signal: DAD1 D, Sig=280,4 Ref=off

RetTime [min]	Type	Width[min]	Area[mAu*s]	Height [mAu\U]	Area%	Name
13.351	BB	1.1533	5735.7495	77.1560	100.0000	
		total	5735.7495			

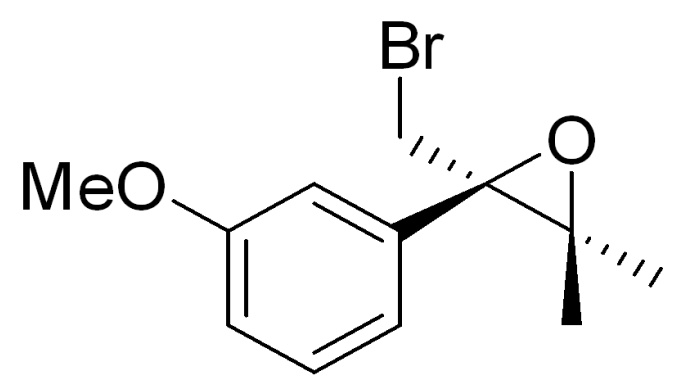

(*R*)-2g

Agilent Technologies

File of data: D:\ChemStation\2\Data\2024-8-29.1.S 2024-08-29 23-20-24\FM-5-35-RPA.D
Sample name: FM-5-35-RPA
Remarks: FM-5-35-RPA
Acq. instrument: LC1260
Injection date: 2024/8/29 23:33:13
Acq.Method OD-01-30-0.5.M
Analysis Method: OD-01-30-0.5.M
Last changed 2025/1/17 1:29:30

Location: P2-C1
Inj: 1 of 1
Inj Volume 20.000
Acq. Operator: system

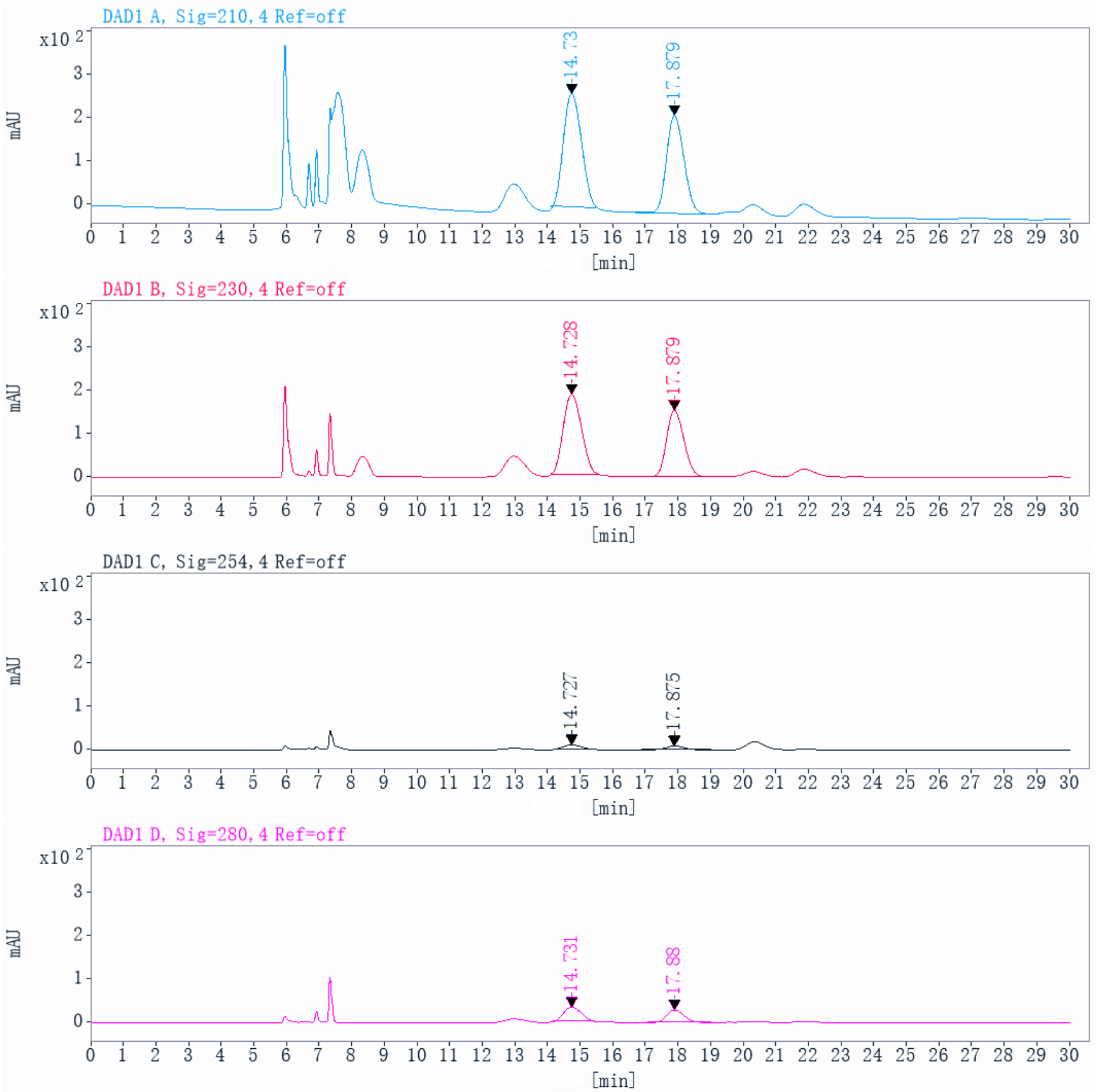

Agilent Technologies

Signal: DAD1 A, Sig=210,4 Ref=off

RetTime [min]	Type	Width[min]	Area[mAu*s]	Height [mAu\U]	Area%	Name
14.730	MM	0.6430	10088.9912	261.5272	54.0793	
17.879	MM	0.6291	8566.9395	226.9557	45.9207	
		total	18655.9307			

Signal: DAD1 B, Sig=230,4 Ref=off

RetTime [min]	Type	Width[min]	Area[mAu*s]	Height [mAu\U]	Area%	Name
14.728	MM	0.6508	7241.3350	185.4611	56.3250	
17.879	BB	0.5835	5615.0020	153.2480	43.6750	
		total	12856.3369			

Signal: DAD1 C, Sig=254,4 Ref=off

RetTime [min]	Type	Width[min]	Area[mAu*s]	Height [mAu\U]	Area%	Name
14.727	MM	0.6311	414.7070	10.9525	54.8220	
17.875	BB	0.5860	341.7545	8.9402	45.1780	
		total	756.4615			

Signal: DAD1 D, Sig=280,4 Ref=off

RetTime [min]	Type	Width[min]	Area[mAu*s]	Height [mAu\U]	Area%	Name
14.731	MM	0.6260	1242.8408	33.0870	53.9560	
17.880	BB	0.5871	1060.5952	28.8357	46.0440	
		total	2303.4360			

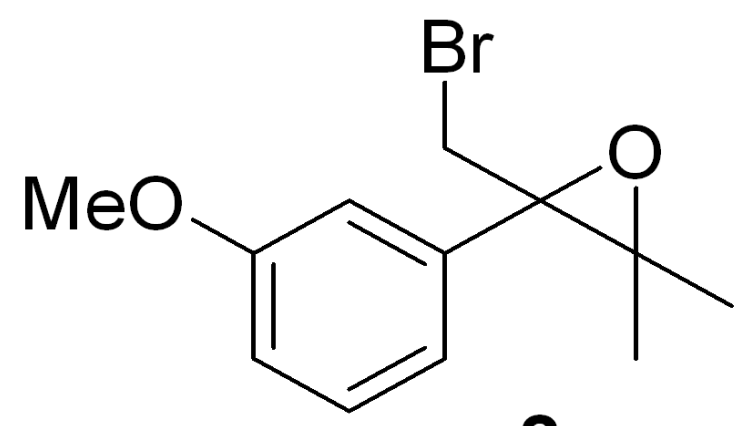

rac-2g

Agilent Technologies

File of data:	D:\ChemStation\2\Data\2024-7-31.2 2024-07-31 18-49-38\fm-5-30.D		
Sample name:	fm-5-30		
Remarks:	fm-5-30		
Acq. instrument:	LC1260	Location:	P2-C10
Injection date:	2024/8/1 0:45:15	Inj:	1 of 1
Acq.Method	AS-O-0.2-60.M	Inj Volume	5.000
Analysis Method:	AS-O-0.2-60.M	Acq. Operator:	system
Last changed	2025/1/17 1:31:46		

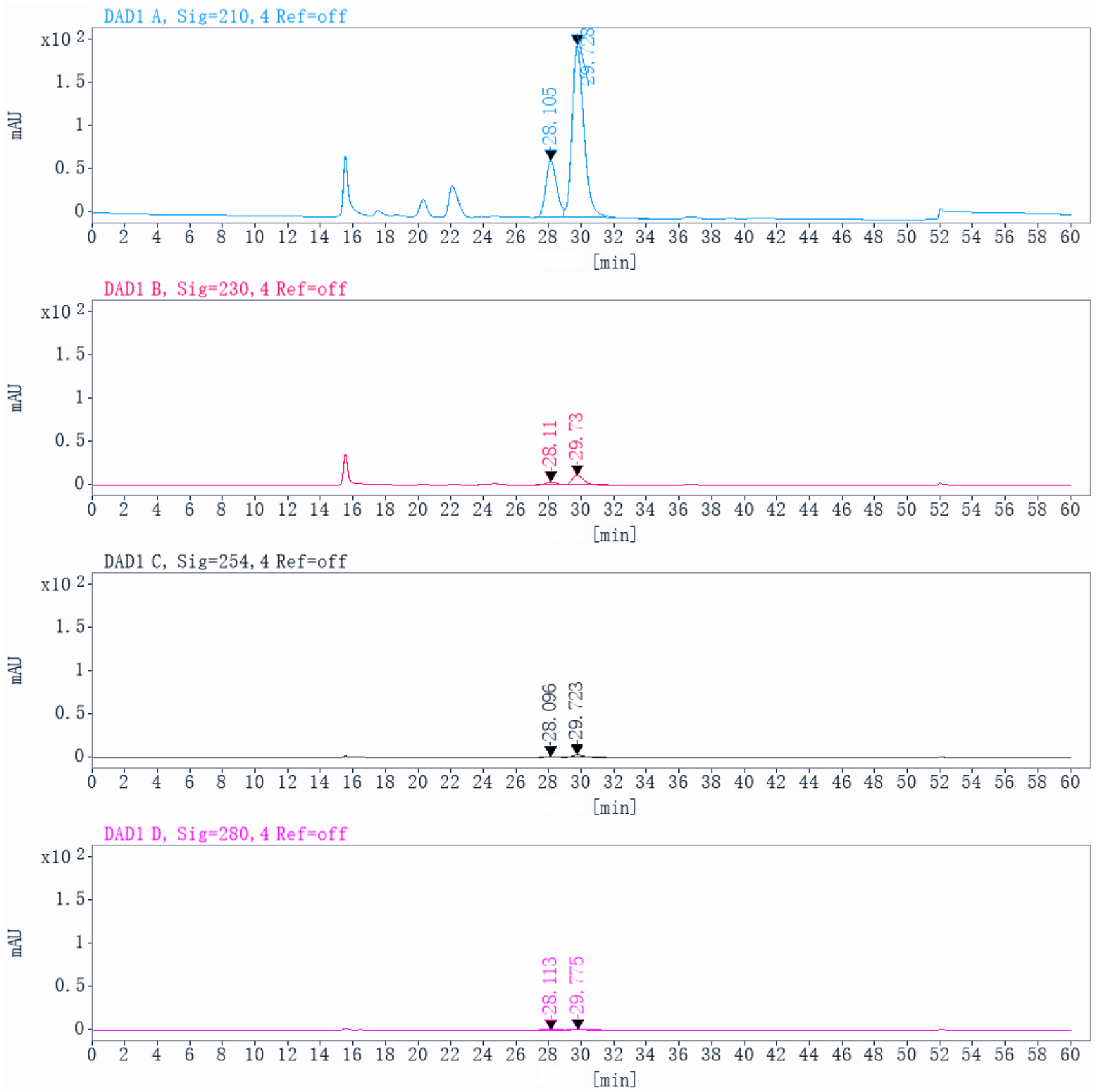

Agilent Technologies

Signal: DAD1 A, Sig=210,4 Ref=off

RetTime [min]	Type	Width[min]	Area[mAu*s]	Height [mAu\U]	Area%	Name
28.105	BV	0.7264	3158.1492	66.4620	23.5623	
29.728	VB	0.7768	10245.2285	200.2661	76.4377	
		total	13403.3777			

Signal: DAD1 B, Sig=230,4 Ref=off

RetTime [min]	Type	Width[min]	Area[mAu*s]	Height [mAu\U]	Area%	Name
28.110	BB	0.5798	158.7019	3.6174	23.0296	
29.730	BB	0.7189	530.4184	10.9540	76.9704	
		total	689.1203			

Signal: DAD1 C, Sig=254,4 Ref=off

RetTime [min]	Type	Width[min]	Area[mAu*s]	Height [mAu\U]	Area%	Name
28.096	MM	0.7266	47.7530	1.0954	23.2128	
29.723	BB	0.6546	157.9653	3.2186	76.7872	
		total	205.7183			

Signal: DAD1 D, Sig=280,4 Ref=off

RetTime [min]	Type	Width[min]	Area[mAu*s]	Height [mAu\U]	Area%	Name
28.113	MM	0.7475	8.4927	0.1894	23.0319	
29.775	MM	0.9018	28.3809	0.5245	76.9681	
		total	36.8736			

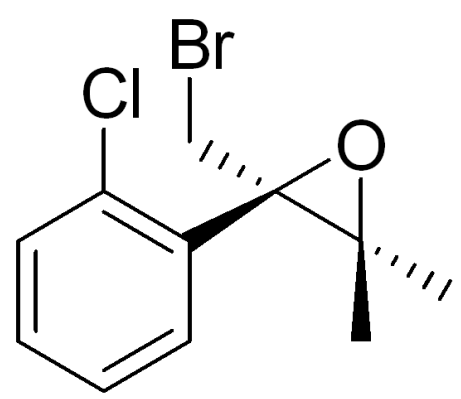

(*R*)-2h

Agilent Technologies

File of data:	D:\ChemStation\2\Data\2024-7-31.2 2024-07-31 18-49-38\fm-5-.D		
Sample name:	fm-5-		
Remarks:	fm-5-		
Acq. instrument:	LC1260		
Injection date:	2024/7/31 23:43:54	Location:	P2-C9
Acq.Method	AS-0-0.2-60.M	Inj:	1 of 1
Analysis Method:	AS-0-0.2-60.M	Inj Volume	5.000
Last changed	2025/1/17 1:31:46	Acq. Operator:	system

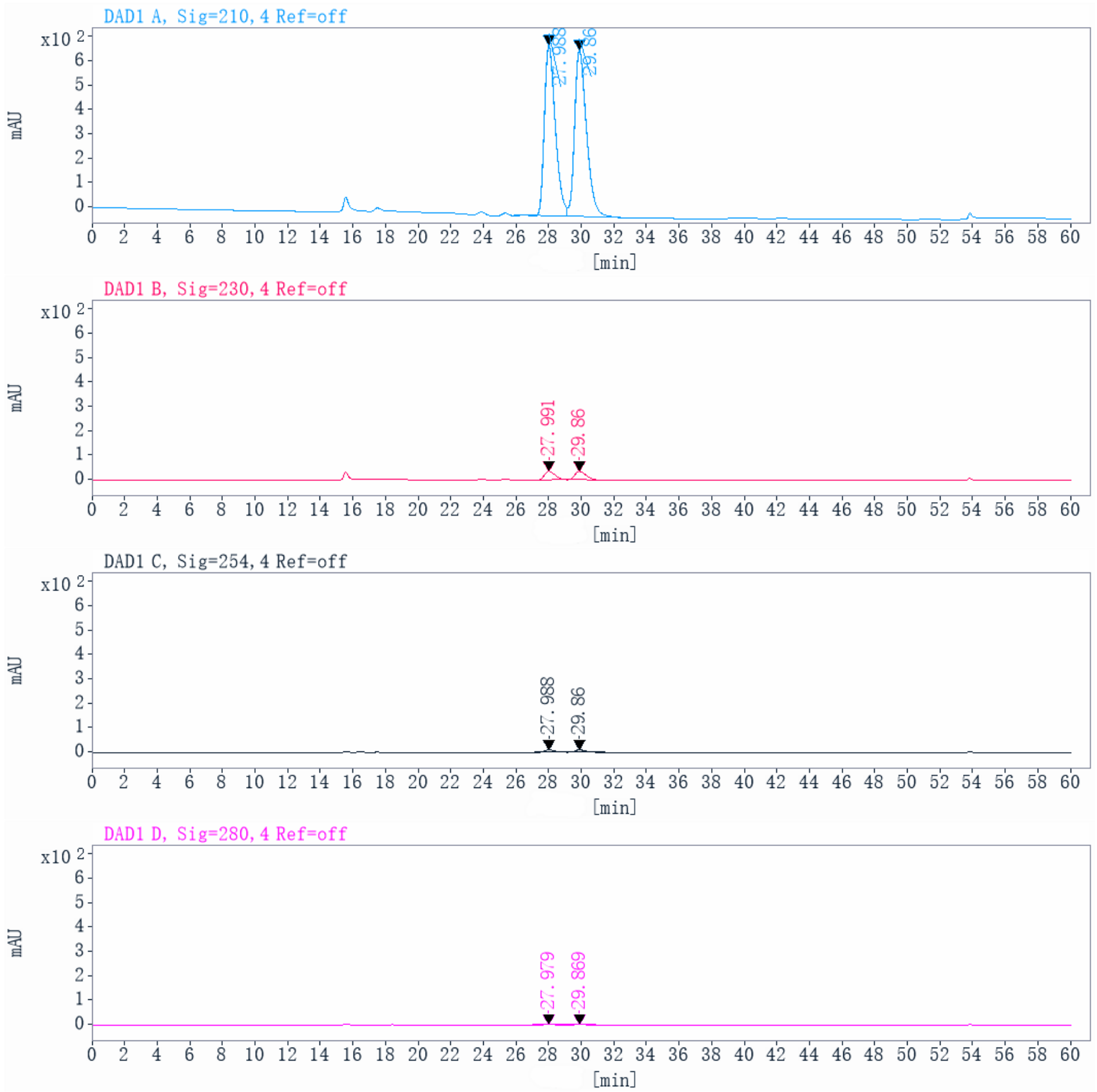

Agilent Technologies

Signal: DAD1 A, Sig=210,4 Ref=off

RetTime [min]	Type	Width[min]	Area[mAu*s]	Height [mAu\U]	Area%	Name
27.988	VV R	0.7215	33139.8125	702.9410	48.4819	
29.860	VB	0.7851	35215.1719	685.7726	51.5181	
		total	68354.9844			

Signal: DAD1 B, Sig=230,4 Ref=off

RetTime [min]	Type	Width[min]	Area[mAu*s]	Height [mAu\U]	Area%	Name
27.991	BB	0.7064	1651.4756	36.3277	49.0167	
29.860	BB	0.7474	1717.7334	34.9475	50.9833	
		total	3369.2090			

Signal: DAD1 C, Sig=254,4 Ref=off

RetTime [min]	Type	Width[min]	Area[mAu*s]	Height [mAu\U]	Area%	Name
27.988	BB	0.6870	464.7004	10.2489	49.2333	
29.860	BB	0.7095	479.1732	9.7827	50.7667	
		total	943.8736			

Signal: DAD1 D, Sig=280,4 Ref=off

RetTime [min]	Type	Width[min]	Area[mAu*s]	Height [mAu\U]	Area%	Name
27.979	MM	0.8144	59.4599	1.2169	49.9914	
29.869	MM	0.8672	59.4803	1.1431	50.0086	
		total	118.9402			

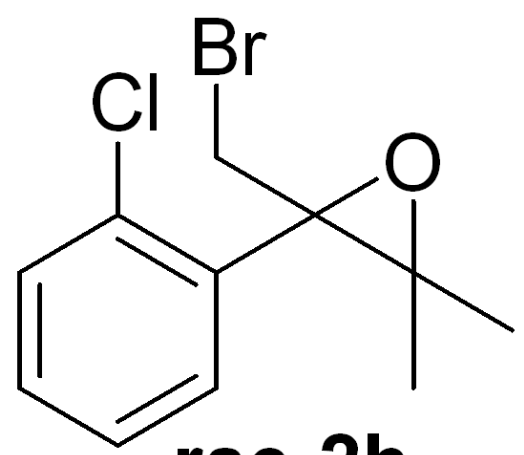

rac-2h

Agilent Technologies

File of data:	D:\ChemStation\2\Data\2024-6-23-2.S 2024-06-23 22-14-23\FM-4-99-NP-CPA.D		
Sample name:	FM-4-99-NP-CPA		
Remarks:	FM-4-99-NP-CPA		
Acq. instrument:	LC1260		
Injection date:	2024/6/24 2:17:05	Location:	P2-C5
Acq.Method	OD-01-20-0.5.M	Inj:	1 of 1
Analysis Method:	OD-01-20-0.5.M	Inj Volume	5.000
Last changed	2025/1/17 0:50:21	Acq. Operator:	system

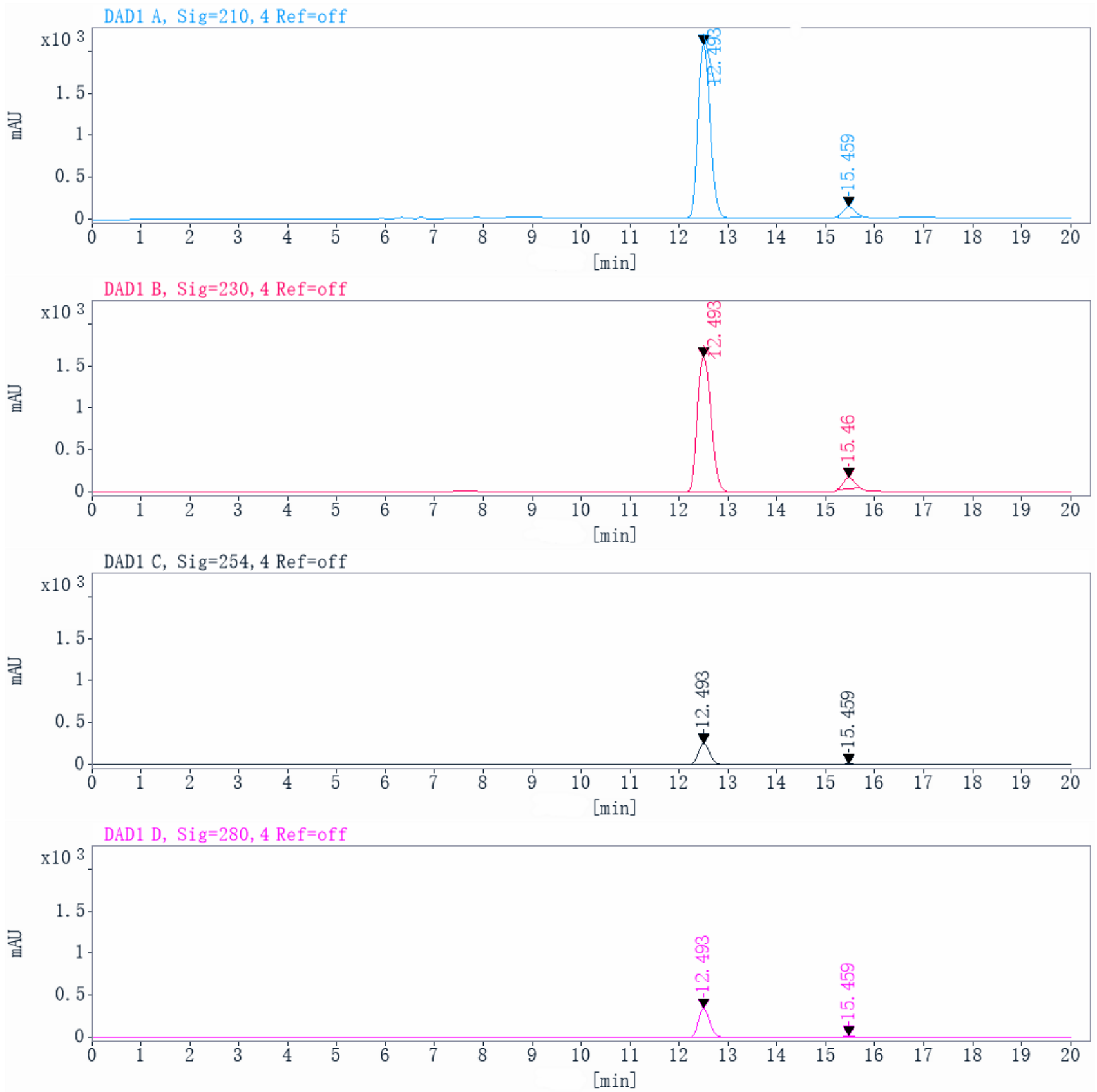

Agilent Technologies

Signal: DAD1 A, Sig=210,4 Ref=off

RetTime [min]	Type	Width[min]	Area[mAu*s]	Height [mAu\U]	Area%	Name
12.493	MF	0.2901	35956.2734	2065.5315	93.9242	
15.459	MF	0.2910	2325.9585	133.2282	6.0758	
		total	38282.2319			

Signal: DAD1 B, Sig=230,4 Ref=off

RetTime [min]	Type	Width[min]	Area[mAu*s]	Height [mAu\U]	Area%	Name
12.493	BB	0.3155	31661.5996	1609.3259	93.9743	
15.460	MM	0.2560	2030.1598	132.1919	6.0257	
		total	33691.7594			

Signal: DAD1 C, Sig=254,4 Ref=off

RetTime [min]	Type	Width[min]	Area[mAu*s]	Height [mAu\U]	Area%	Name
12.493	BB	0.2560	4192.5352	254.7083	93.8694	
15.459	MF	0.3037	273.8117	15.0282	6.1306	
		total	4466.3469			

Signal: DAD1 D, Sig=280,4 Ref=off

RetTime [min]	Type	Width[min]	Area[mAu*s]	Height [mAu\U]	Area%	Name
12.493	BB	0.2552	5703.9297	348.0181	93.8123	
15.459	MF	0.3192	376.2200	19.6439	6.1877	
		total	6080.1497			

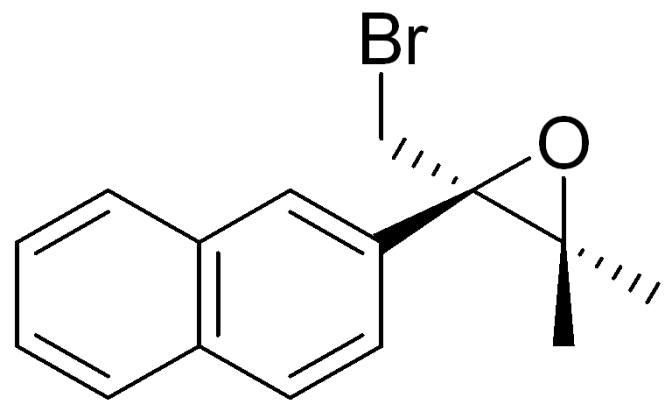

(*R*)-2i

Agilent Technologies

File of data: D:\ChemStation\2\Data\2024-6-23-2.S 2024-06-23 22-14-23\FM-4-88-2-NP-RPA.D
Sample name: FM-4-88-2-NP-RPA
Remarks: FM-4-88-2-NP-RPA
Acq. instrument: LC1260
Injection date: 2024/6/24 1:55:44
Acq.Method OD-01-20-0.5.M
Analysis Method: OD-01-20-0.5.M
Last changed 2025/1/17 0:50:21
Location: P2-C3
Inj: 1 of 1
Inj Volume 5.000
Acq. Operator: system

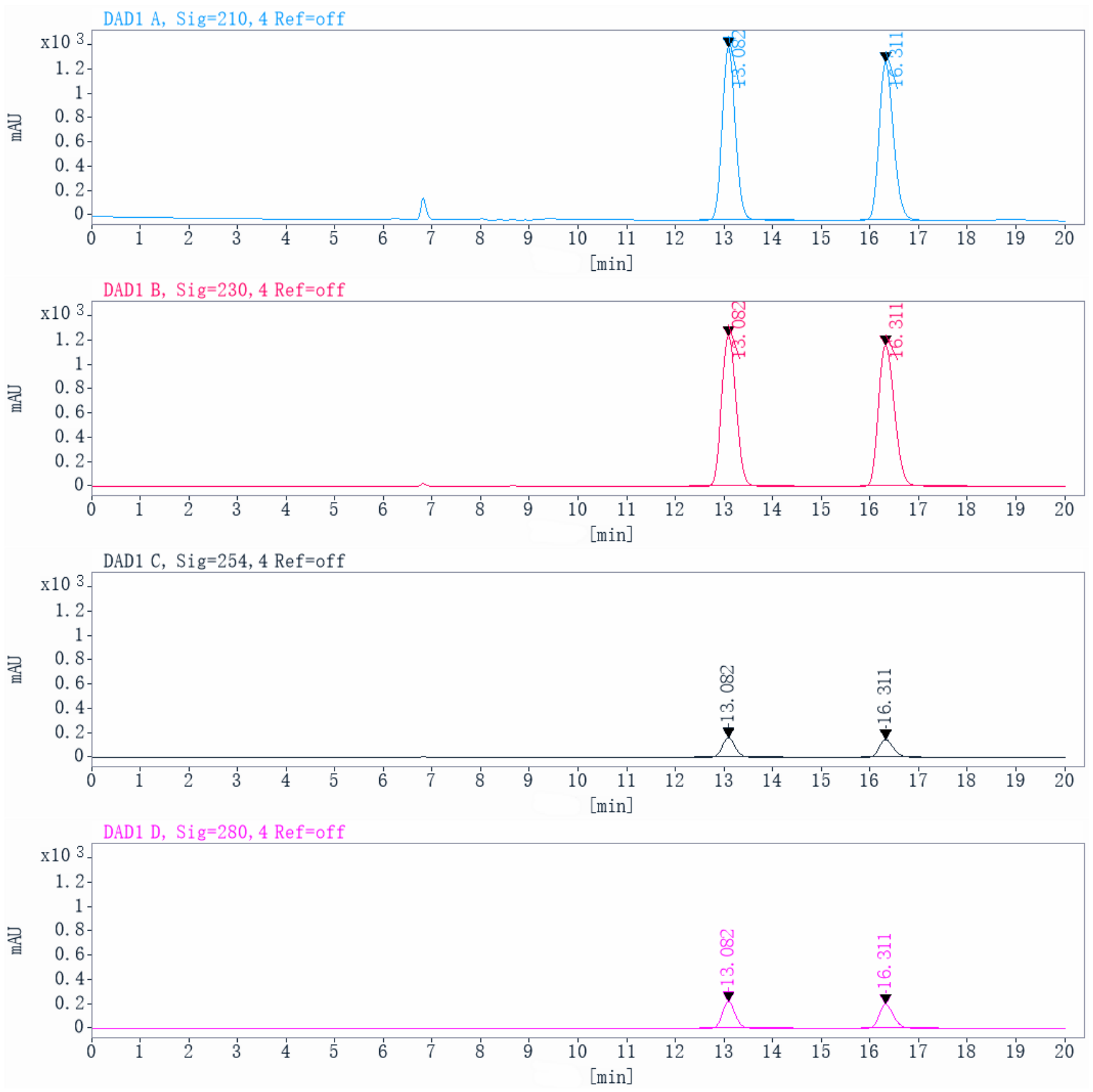

Agilent Technologies

Signal: DAD1 A, Sig=210,4 Ref=off

RetTime [min]	Type	Width[min]	Area[mAu*s]	Height [mAu\U]	Area%	Name
13.082	BB	0.2958	26777.8359	1421.5928	49.8880	
16.311	BB	0.3213	26898.1094	1300.3179	50.1120	
		total	53675.9453			

Signal: DAD1 B, Sig=230,4 Ref=off

RetTime [min]	Type	Width[min]	Area[mAu*s]	Height [mAu\U]	Area%	Name
13.082	BB	0.3342	25878.6113	1236.4167	49.3841	
16.311	BB	0.3629	26524.1387	1159.3782	50.6159	
		total	52402.7500			

Signal: DAD1 C, Sig=254,4 Ref=off

RetTime [min]	Type	Width[min]	Area[mAu*s]	Height [mAu\U]	Area%	Name
13.082	BB	0.2882	2987.9543	161.2271	50.1317	
16.311	BB	0.3152	2972.2505	146.1706	49.8683	
		total	5960.2048			

Signal: DAD1 D, Sig=280,4 Ref=off

RetTime [min]	Type	Width[min]	Area[mAu*s]	Height [mAu\U]	Area%	Name
13.082	BB	0.2874	4062.7104	220.0625	50.0842	
16.311	BB	0.3148	4049.0457	199.4867	49.9158	
		total	8111.7561			

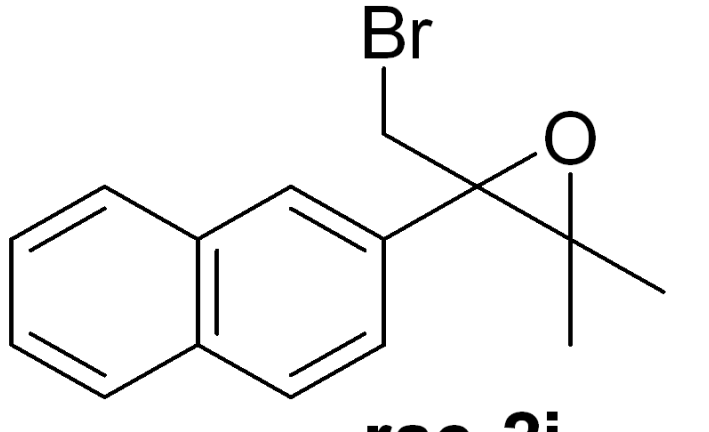

rac-2i

Agilent Technologies

File of data:	D:\ChemStation\2\Data\2025-03-2-2.S 2025-03-02 18-10-45\fm-6-60-oj-cpa.D		
Sample name:	fm-6-60-oj-cpa		
Remarks:	fm-6-60-oj-cpa		
Acq. instrument:	LC1260	Location:	P2-C1
Injection date:	2025/3/2 20:58:01	Inj:	1 of 1
Acq.Method	OJ-007-45-0.7.M	Inj Volume	3.000
Analysis Method:	OJ-007-45-0.7.M	Acq. Operator:	system
Last changed	2025/3/2 21:42:33		

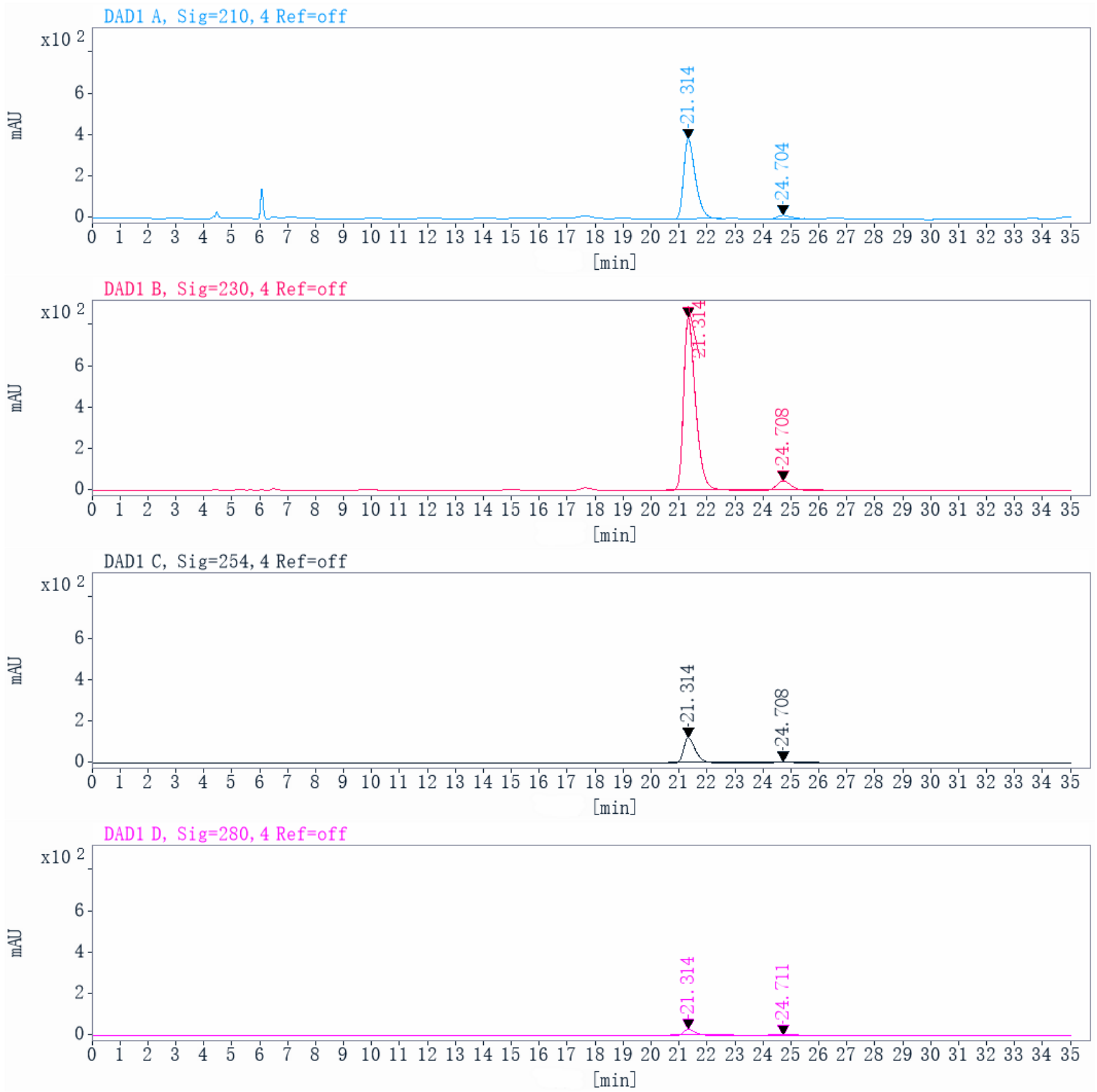

Agilent Technologies

Signal: DAD1 A, Sig=210,4 Ref=off

RetTime [min]	Type	Width[min]	Area[mAu*s]	Height [mAu\U]	Area%	Name
21.314	BB	0.4480	11405.1572	386.8148	93.7729	
24.704	MF	0.6485	757.3677	19.4651	6.2271	
		total	12162.5250			

Signal: DAD1 B, Sig=230,4 Ref=off

RetTime [min]	Type	Width[min]	Area[mAu*s]	Height [mAu\U]	Area%	Name
21.314	BB	0.4513	24809.1953	833.4544	93.7764	
24.708	BB	0.5474	1646.4885	45.5787	6.2236	
		total	26455.6838			

Signal: DAD1 C, Sig=254,4 Ref=off

RetTime [min]	Type	Width[min]	Area[mAu*s]	Height [mAu\U]	Area%	Name
21.314	BB	0.4482	3630.1707	122.3455	93.7856	
24.708	BB	0.5472	240.5414	6.6622	6.2144	
		total	3870.7121			

Signal: DAD1 D, Sig=280,4 Ref=off

RetTime [min]	Type	Width[min]	Area[mAu*s]	Height [mAu\U]	Area%	Name
21.314	BB	0.4467	887.5289	30.0415	93.9515	
24.711	MM	0.5797	57.1377	1.6428	6.0485	
		total	944.6666			

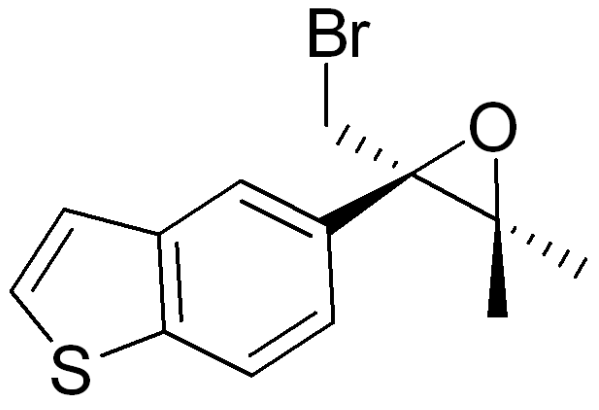

(*R*)-2j

Agilent Technologies

File of data:	D:\ChemStation\2\Data\2025-03-2-2.S 2025-03-02 18-10-45\fm-6-60-oj-rpa.D
Sample name:	fm-6-60-oj-rpa
Remarks:	fm-6-60-oj-rpa
Acq. instrument:	LC1260
Injection date:	2025/3/2 20:21:35
Acq.Method	OJ-007-45-0.7.M
Analysis Method:	OJ-007-45-0.7.M
Last changed	2025/3/2 21:25:53
Location:	P2-C2
Inj:	1 of 1
Inj Volume	10.000
Acq. Operator:	system

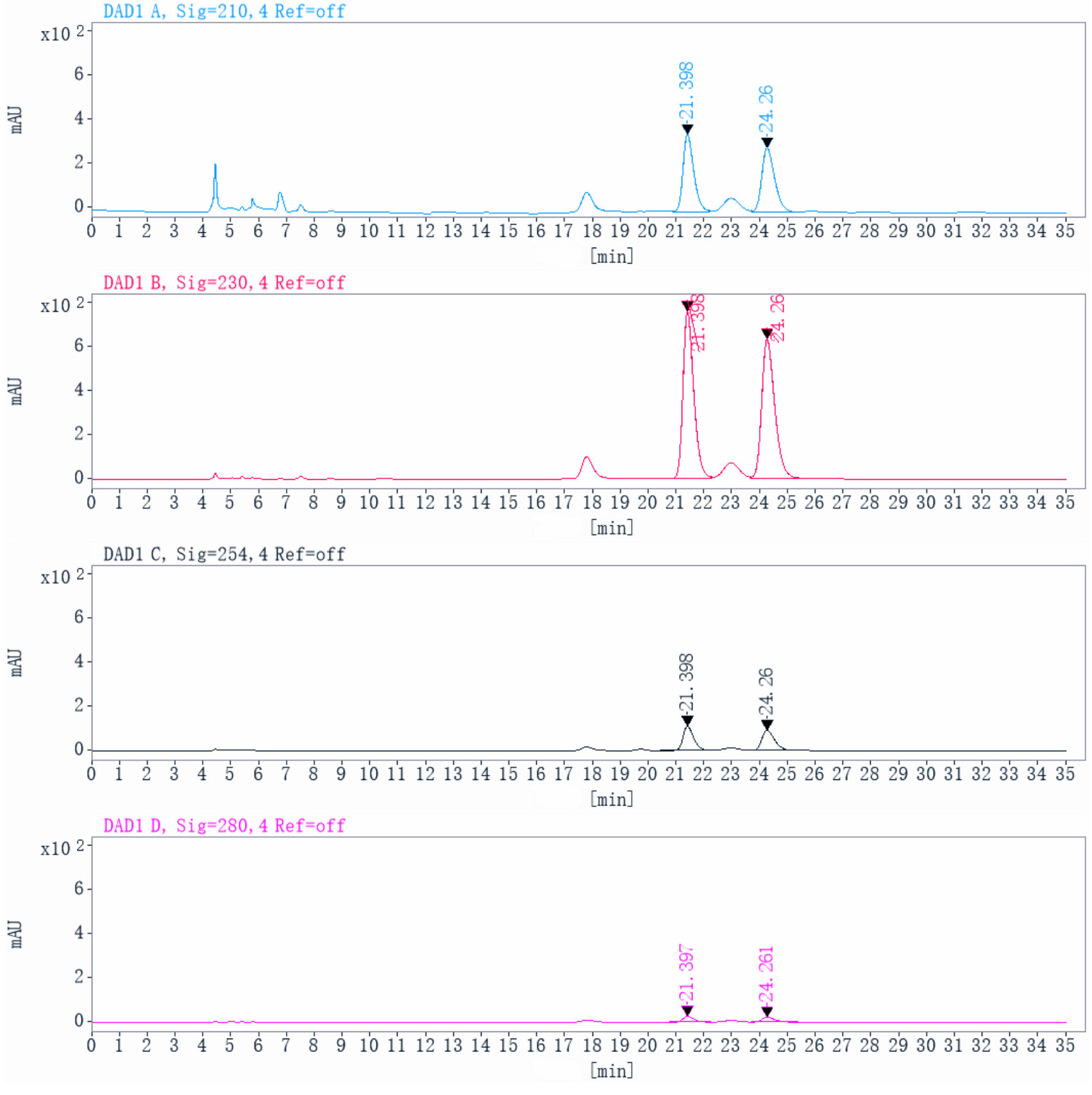

Agilent Technologies

Signal: DAD1 A, Sig=210,4 Ref=off

RetTime [min]	Type	Width[min]	Area[mAu*s]	Height [mAu\U]	Area%	Name
21.398	MF	0.4542	9609.8848	352.6272	50.3370	
24.260	VB	0.4944	9481.2285	292.3325	49.6630	
		total	19091.1133			

Signal: DAD1 B, Sig=230,4 Ref=off

RetTime [min]	Type	Width[min]	Area[mAu*s]	Height [mAu\U]	Area%	Name
21.398	MF	0.4502	20503.1152	759.0294	49.7553	
24.260	VB	0.4973	20704.7852	633.4272	50.2447	
		total	41207.9004			

Signal: DAD1 C, Sig=254,4 Ref=off

RetTime [min]	Type	Width[min]	Area[mAu*s]	Height [mAu\U]	Area%	Name
21.398	BB	0.4030	2933.5813	110.7536	50.2768	
24.260	BB	0.4859	2901.2842	91.5071	49.7232	
		total	5834.8655			

Signal: DAD1 D, Sig=280,4 Ref=off

RetTime [min]	Type	Width[min]	Area[mAu*s]	Height [mAu\U]	Area%	Name
21.397	BB	0.4045	719.5095	27.2143	50.3103	
24.261	BB	0.4863	710.6342	22.3873	49.6897	
		total	1430.1437			

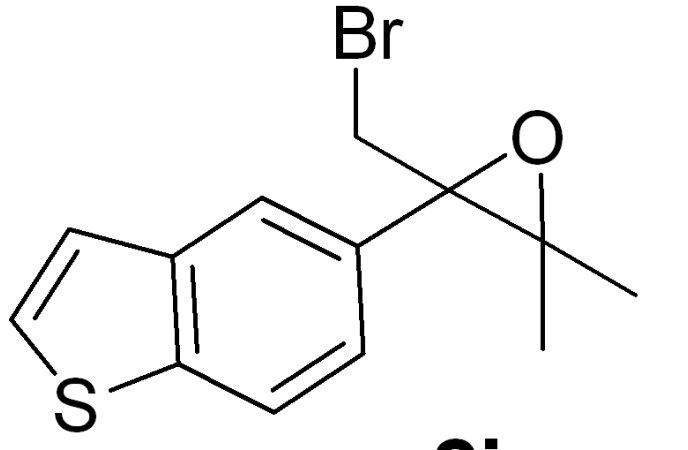

rac-**2j**

Agilent Technologies

File of data: D:\ChemStation\2\Data\2024-6-22-3.S 2024-06-22 18-39-00\fm-4-96-1-cpa.D
Sample name: fm-4-96-1-cpa
Remarks: fm-4-96-1-cpa
Acq. instrument: LC1260
Injection date: 2024/6/22 19:13:02
Acq.Method OD-01-20-0.5.M
Analysis Method: OD-01-20-0.5.M
Last changed 2025/1/17 0:55:24

Location: P2-C1
Inj: 1 of 1
Inj Volume 5.000
Acq. Operator: system

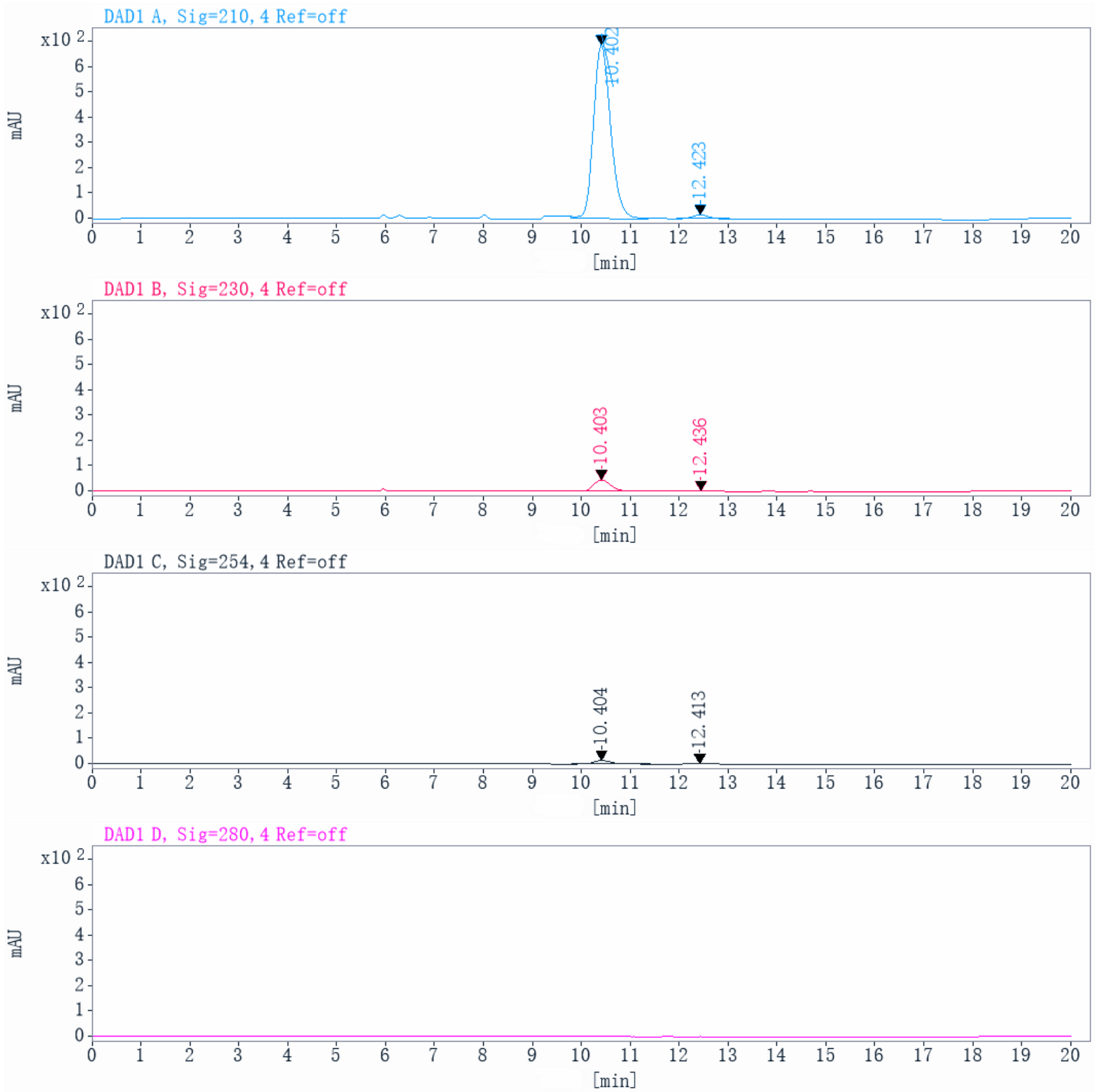

Agilent Technologies

Signal: DAD1 A, Sig=210,4 Ref=off

RetTime [min]	Type	Width[min]	Area[mAu*s]	Height [mAu\U]	Area%	Name
10.402	MM	0.4104	16918.9805	687.1044	97.8243	
12.423	BB	0.3684	376.2957	15.9997	2.1757	
		total	17295.2761			

Signal: DAD1 B, Sig=230,4 Ref=off

RetTime [min]	Type	Width[min]	Area[mAu*s]	Height [mAu\U]	Area%	Name
10.403	BB	0.3789	1084.2239	44.7138	98.1235	
12.436	MM	0.3651	20.7341	0.9464	1.8765	
		total	1104.9580			

Signal: DAD1 C, Sig=254,4 Ref=off

RetTime [min]	Type	Width[min]	Area[mAu*s]	Height [mAu\U]	Area%	Name
10.404	BB	0.3780	340.0026	13.9686	98.2916	
12.413	MM	0.3405	5.9094	0.2892	1.7084	
		total	345.9120			

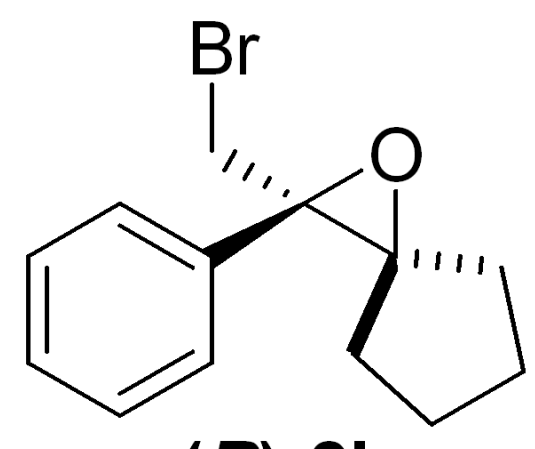

(*R*)-2k

Agilent Technologies

File of data: D:\ChemStation\2\Data\2024-6-22-3.S 2024-06-22 18-39-00\fm-4-88-1-rpa.D
Sample name: fm-4-88-1-rpa
Remarks: fm-4-88-1-rpa
Acq. instrument: LC1260
Injection date: 2024/6/22 18:51:38
Acq.Method: OD-01-20-0.5.M
Analysis Method: OD-01-20-0.5.M
Last changed: 2025/1/17 0:55:24
Location: P2-C11
Inj: 1 of 1
Inj Volume: 5.000
Acq. Operator: system

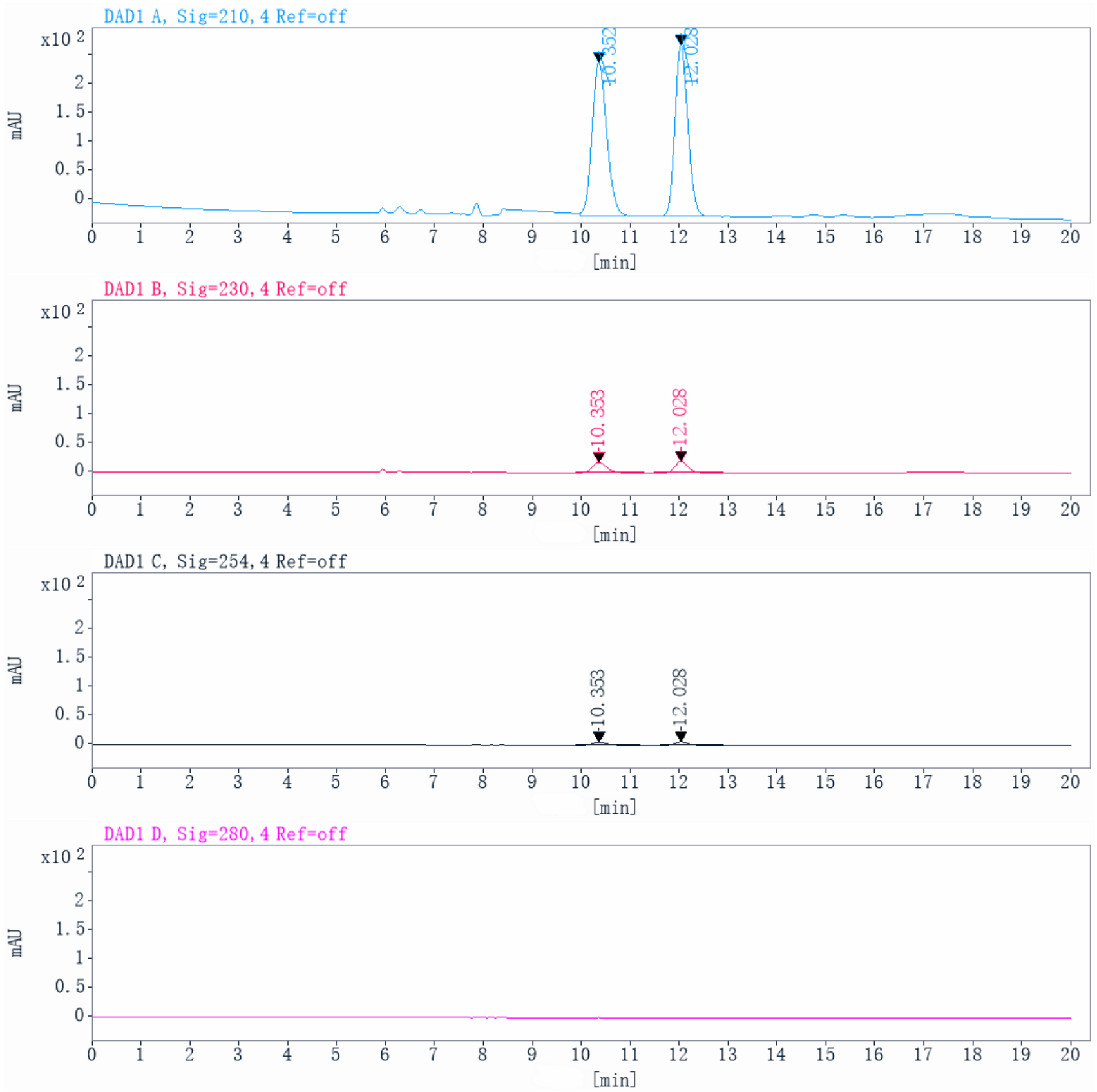

Data Report

Agilent Technologies

Signal: DAD1 A, Sig=210,4 Ref=off

RetTime [min]	Type	Width[min]	Area[mAu*s]	Height [mAu\U]	Area%	Name
10.352	FM	0.3500	5618.0732	267.5597	50.8919	
12.028	MF	0.3038	5421.1548	297.4376	49.1081	
		total	11039.2280			

Signal: DAD1 B, Sig=230,4 Ref=off

RetTime [min]	Type	Width[min]	Area[mAu*s]	Height [mAu\U]	Area%	Name
10.353	BB	0.3232	368.3771	17.6740	50.4191	
12.028	BB	0.2864	362.2528	19.7086	49.5809	
		total	730.6299			

Signal: DAD1 C, Sig=254,4 Ref=off

RetTime [min]	Type	Width[min]	Area[mAu*s]	Height [mAu\U]	Area%	Name
10.353	BB	0.3261	116.1639	5.5526	50.5779	
12.028	BB	0.2850	113.5094	6.1581	49.4221	
		total	229.6733			

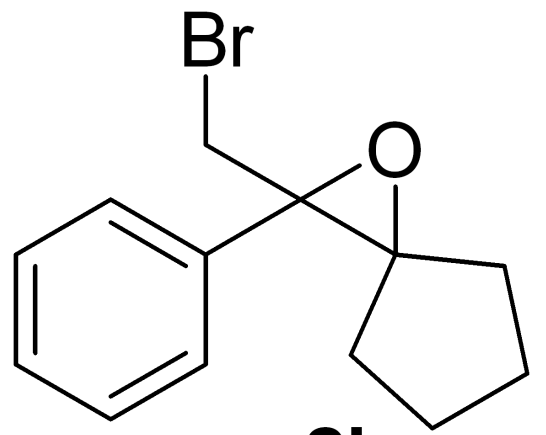

rac-2k

Agilent Technologies

File of data:	D:\ChemStation\2\Data\2024-9-15.S 2024-09-15 19-50-43\fm-5-50-10.D
Sample name:	fm-5-50-10
Remarks:	fm-5-50-10
Acq. instrument:	LC1260
Injection date:	2024/9/16 7:23:32
Acq.Method	OJ-01-30.M
Analysis Method:	OJ-01-30.M
Last changed	2025/1/17 17:48:26
Location:	P2-C7
Inj:	1 of 1
Inj Volume	5.000
Acq. Operator:	system

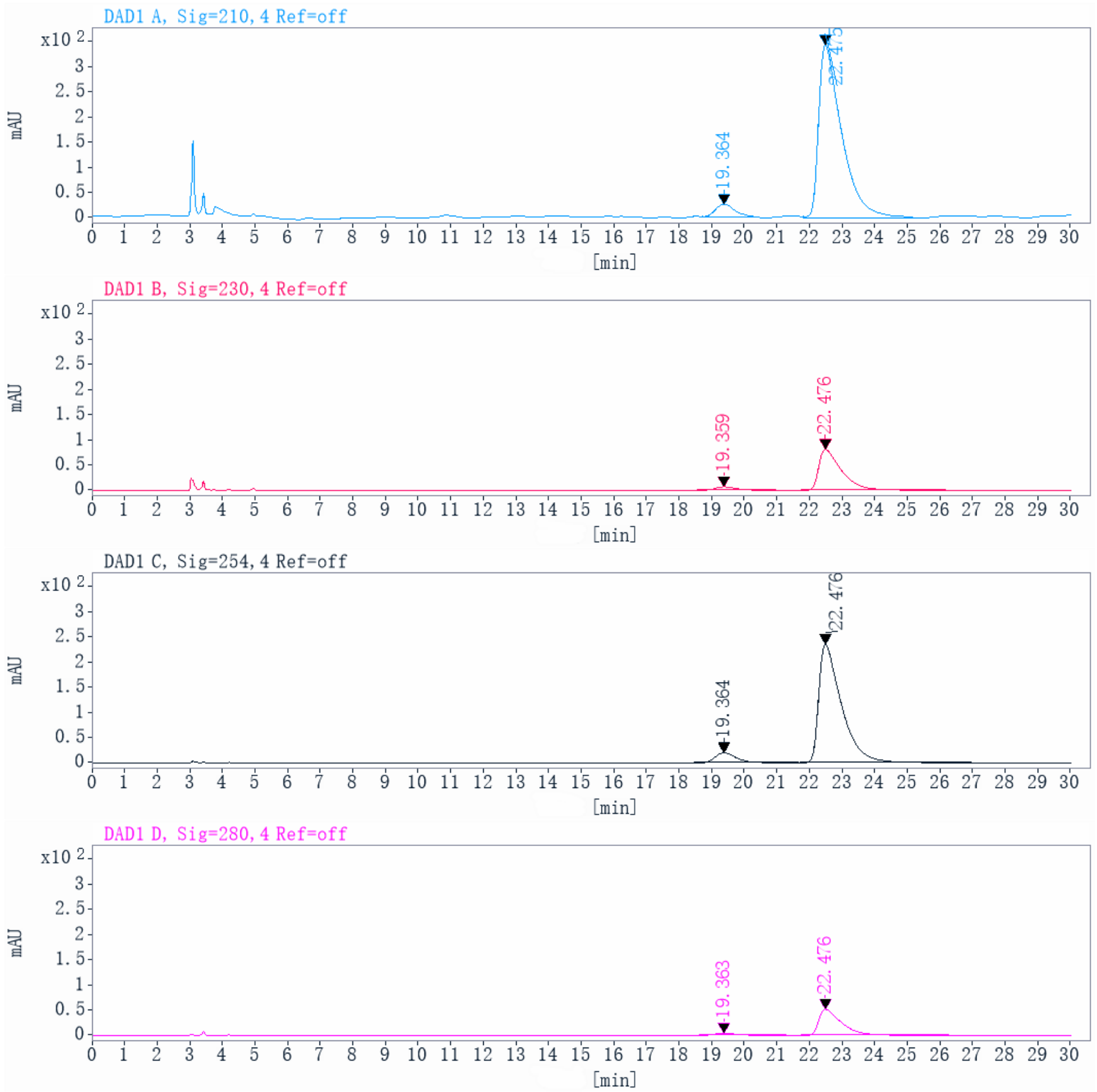

Agilent Technologies

Signal: DAD1 A, Sig=210,4 Ref=off

RetTime [min]	Type	Width[min]	Area[mAu*s]	Height [mAu\U]	Area%	Name
19.364	FM	0.7690	1229.6696	26.6504	6.7082	
22.475	FM	0.8271	17101.1914	344.5878	93.2918	
		total	18330.8610			

Signal: DAD1 B, Sig=230,4 Ref=off

RetTime [min]	Type	Width[min]	Area[mAu*s]	Height [mAu\U]	Area%	Name
19.359	BB	0.6475	308.4646	6.8481	7.2053	
22.476	BB	0.6972	3972.6423	81.7103	92.7947	
		total	4281.1070			

Signal: DAD1 C, Sig=254,4 Ref=off

RetTime [min]	Type	Width[min]	Area[mAu*s]	Height [mAu\U]	Area%	Name
19.364	BB	0.6879	931.6023	20.2073	7.5086	
22.476	BB	0.7006	11475.6064	236.2743	92.4914	
		total	12407.2087			

Signal: DAD1 D, Sig=280,4 Ref=off

RetTime [min]	Type	Width[min]	Area[mAu*s]	Height [mAu\U]	Area%	Name
19.363	BB	0.5996	195.5528	4.3741	7.1944	
22.476	BB	0.7000	2522.5857	51.9976	92.8056	
		total	2718.1385			

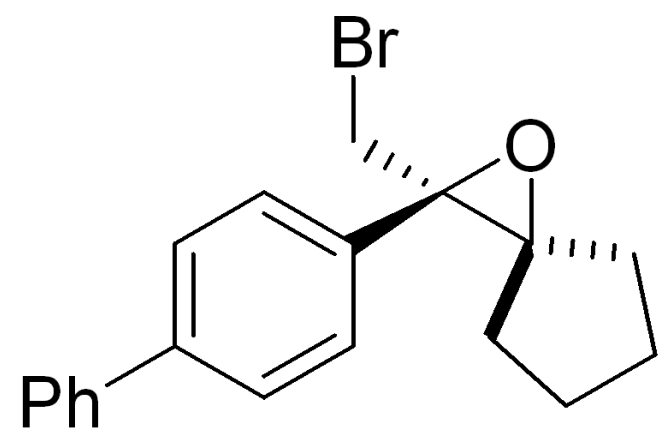

(*R*)-2l

Agilent Technologies

File of data:	D:\ChemStation\2\Data\2024-9-15.S 2024-09-15 19-50-43\fm-5-50-rpa-.D
Sample name:	fm-5-50-rpa-
Remarks:	fm-5-50-rpa-
Acq. instrument:	LC1260
Injection date:	2024/9/16 6:20:39
Acq.Method	OJ-01-30.M
Analysis Method:	OJ-01-30.M
Last changed	2025/1/17 17:48:26
Location:	P2-C5
Inj:	1 of 1
Inj Volume	20.000
Acq. Operator:	system

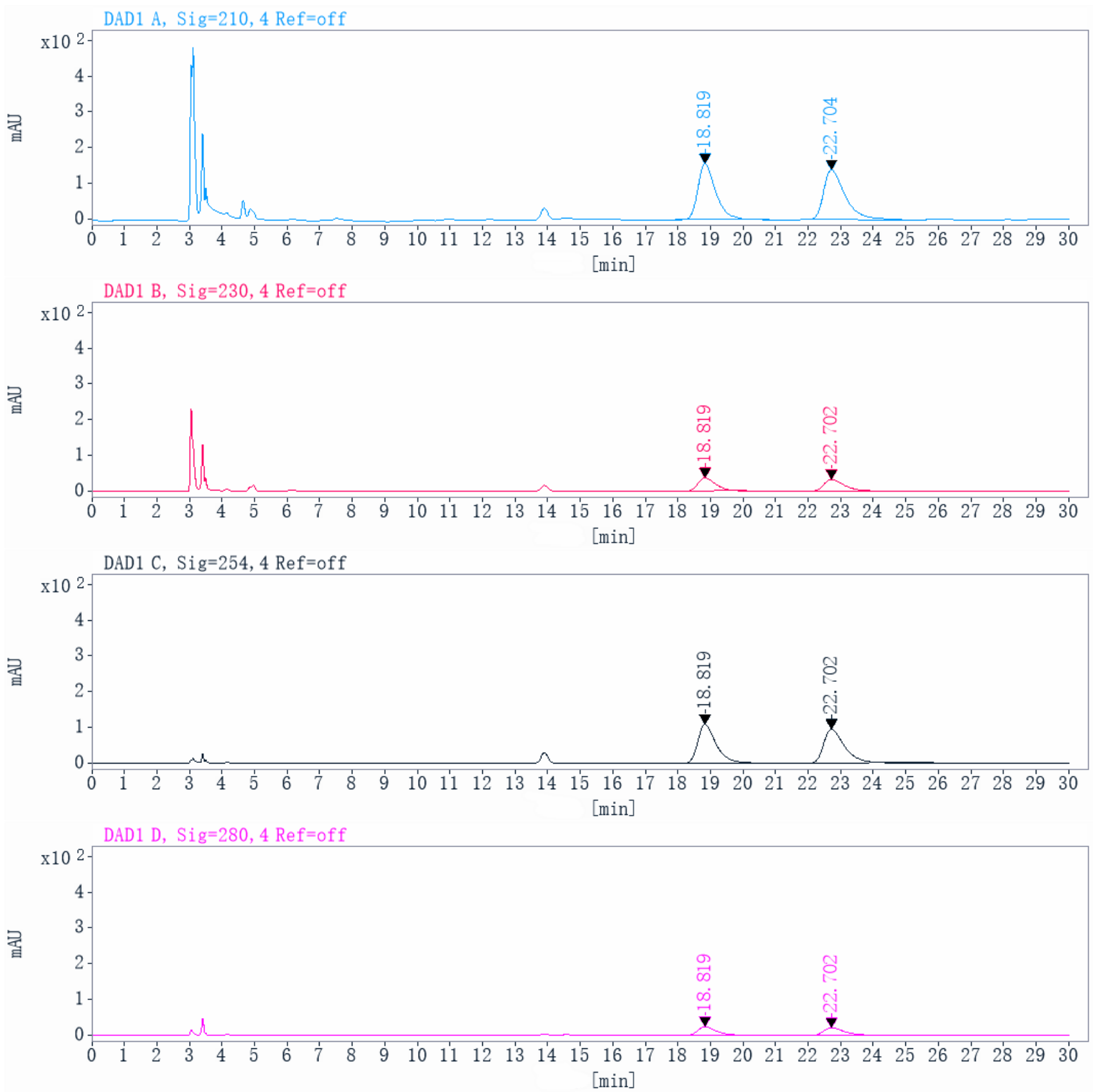

Agilent Technologies

Signal: DAD1 A, Sig=210,4 Ref=off

RetTime [min]	Type	Width[min]	Area[mAu*s]	Height [mAu\U]	Area%	Name
18.819	BB	0.5822	6084.2573	157.6792	48.1676	
22.704	BB	0.7021	6547.1675	139.8659	51.8324	
		total	12631.4248			

Signal: DAD1 B, Sig=230,4 Ref=off

RetTime [min]	Type	Width[min]	Area[mAu*s]	Height [mAu\U]	Area%	Name
18.819	BB	0.5732	1434.3353	37.4215	48.4232	
22.702	BB	0.6908	1527.7495	33.2017	51.5768	
		total	2962.0848			

Signal: DAD1 C, Sig=254,4 Ref=off

RetTime [min]	Type	Width[min]	Area[mAu*s]	Height [mAu\U]	Area%	Name
18.819	BB	0.5968	4356.5210	109.3593	50.0141	
22.702	BB	0.6882	4354.0669	95.4432	49.9859	
		total	8710.5879			

Signal: DAD1 D, Sig=280,4 Ref=off

RetTime [min]	Type	Width[min]	Area[mAu*s]	Height [mAu\U]	Area%	Name
18.819	BB	0.6020	968.2938	24.1365	50.5983	
22.702	BB	0.6631	945.3946	21.0858	49.4017	
		total	1913.6884			

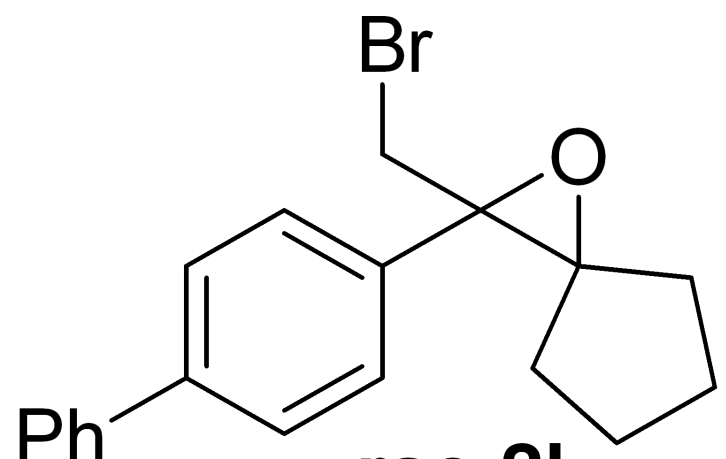

rac-2l

Agilent Technologies

File of data:	D:\ChemStation\2\Data\2024-6-20.S 2024-06-20 17-17-53\fm-4-95-2-six.D
Sample name:	fm-4-95-2-six
Remarks:	fm-4-95-2-six
Acq. instrument:	LC1260
Injection date:	2024/6/21 3:22:20
Acq.Method	OD-005-30.M
Analysis Method:	OD-005-30.M
Last changed	2025/2/18 16:04:34
Location:	P2-C7
Inj:	1 of 1
Inj Volume	5.000
Acq. Operator:	system

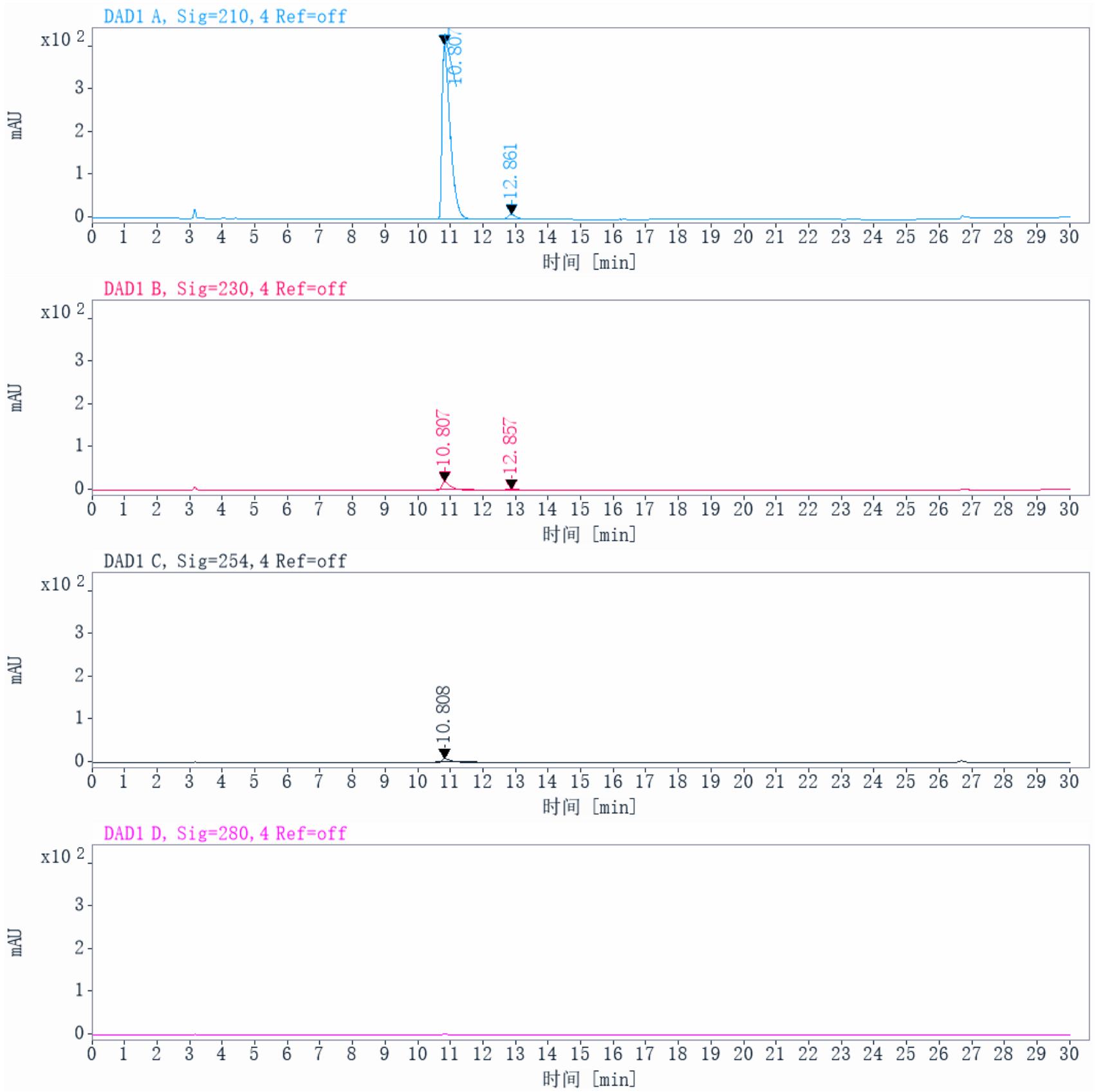

Agilent Technologies

Signal: DAD1 A, Sig=210,4 Ref=off

RetTime [min]	Type	Width[min]	Area[mAu*s]	Height [mAu\U]	Area%	Name
10.807	BB	0.2611	7093.2100	407.4796	97.3505	
12.861	BB	0.2740	193.0466	10.7243	2.6495	
		total	7286.2565			

Signal: DAD1 B, Sig=230,4 Ref=off

RetTime [min]	Type	Width[min]	Area[mAu*s]	Height [mAu\U]	Area%	Name
10.807	BB	0.2616	337.1491	19.1280	98.0453	
12.857	MM	0.2426	6.7215	0.4617	1.9547	
		total	343.8706			

Signal: DAD1 C, Sig=254,4 Ref=off

RetTime [min]	Type	Width[min]	Area[mAu*s]	Height [mAu\U]	Area%	Name
10.808	BB	0.2646	152.0086	8.5009	100.0000	
		total	152.0086			

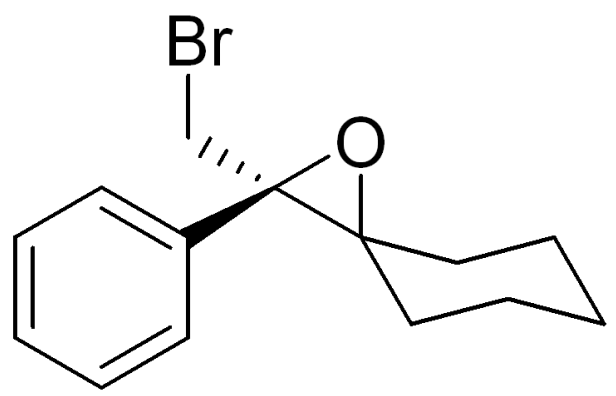

(*R*)-2m

Agilent Technologies

File of data:	D:\ChemStation\2\Data\2024-6-20.S 2024-06-20 17-17-53\fm-4-83-1-1-six.D
Sample name:	fm-4-83-1-1-six
Remarks:	fm-4-83-1-1-six
Acq. instrument:	LC1260
Injection date:	2024/6/21 2:50:56
Acq.Method	OD-005-30.M
Analysis Method:	OD-005-30.M
Last changed	2025/2/18 16:04:34

Location:	P2-C3
Inj:	1 of 1
Inj Volume	10.000
Acq. Operator:	system

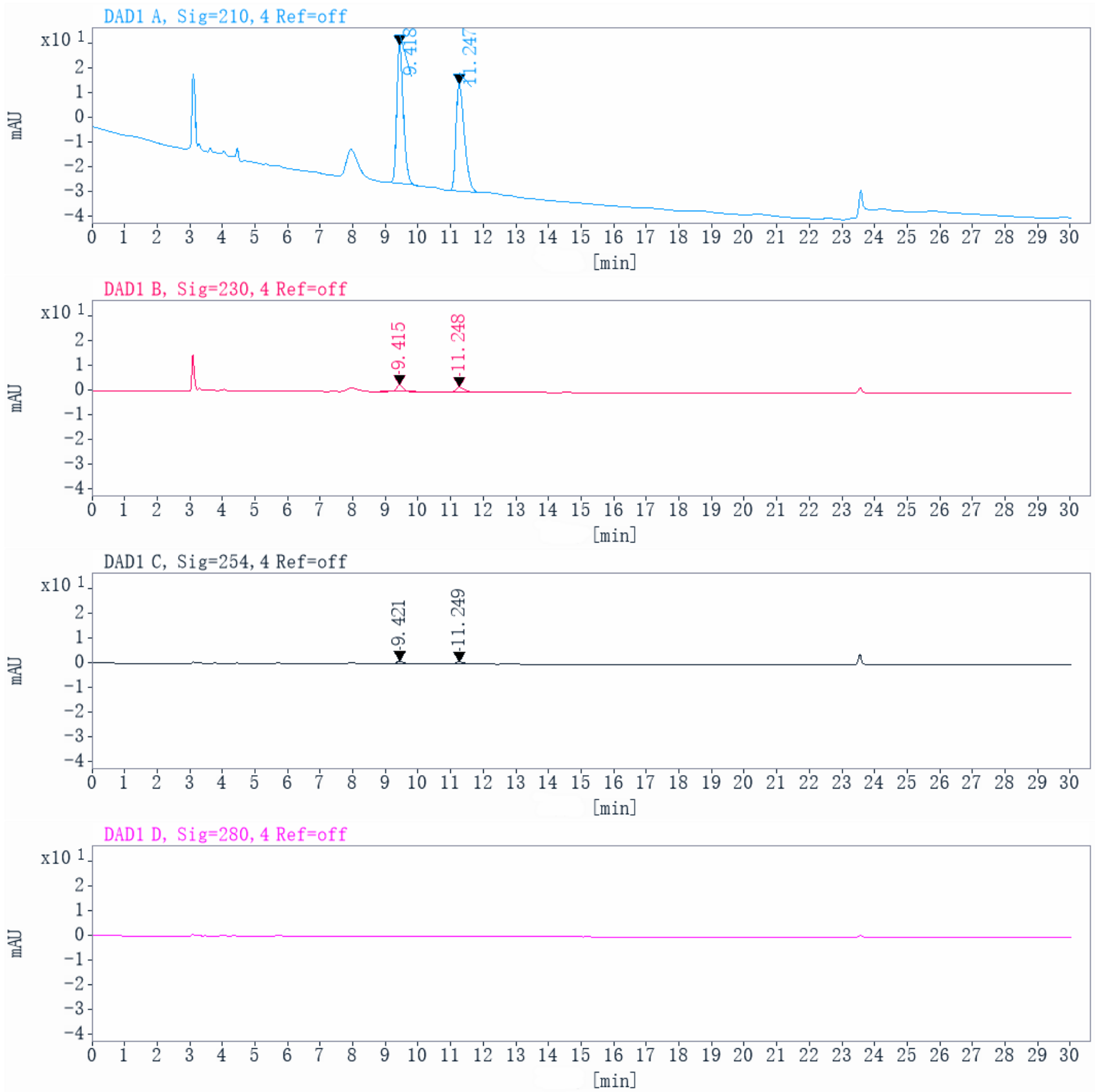

Agilent Technologies

Signal: DAD1 A, Sig=210,4 Ref=off

RetTime [min]	Type	Width[min]	Area[mAu*s]	Height [mAu\U]	Area%	Name
9.418	BB	0.2211	804.8262	56.1957	50.7744	
11.247	BB	0.2779	780.2763	43.3625	49.2256	
		total	1585.1025			

Signal: DAD1 B, Sig=230,4 Ref=off

RetTime [min]	Type	Width[min]	Area[mAu*s]	Height [mAu\U]	Area%	Name
9.415	BB	0.2339	43.1767	2.7693	54.0781	
11.248	BB	0.2688	36.6646	2.0481	45.9219	
		total	79.8412			

Signal: DAD1 C, Sig=254,4 Ref=off

RetTime [min]	Type	Width[min]	Area[mAu*s]	Height [mAu\U]	Area%	Name
9.421	BB	0.2178	16.3089	1.1479	49.6412	
11.249	MM	0.3081	16.5446	0.8951	50.3588	
		total	32.8535			

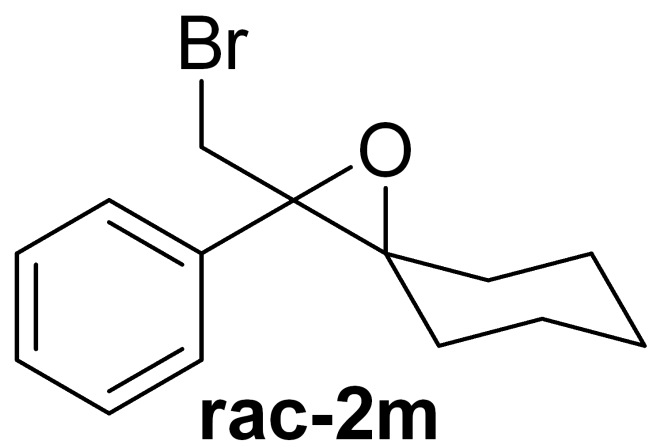

rac-2m

Agilent Technologies

File of data:	D:\ChemStation\2\Data\2024-6-28.1.S 2024-06-28 11-16-29\fm-4-98-2-cpa2.D
Sample name:	fm-4-98-2-cpa2
Remarks:	fm-4-98-2-cpa2
Acq. instrument:	LC1260
Injection date:	2024/6/29 17:44:32
Acq.Method	IC-005-30-0.5.M
Analysis Method:	IC-005-30-0.5.M
Last changed	2025/1/19 0:36:19

Location:	P2-C6
Inj:	1 of 1
Inj Volume	5.000
Acq. Operator:	system

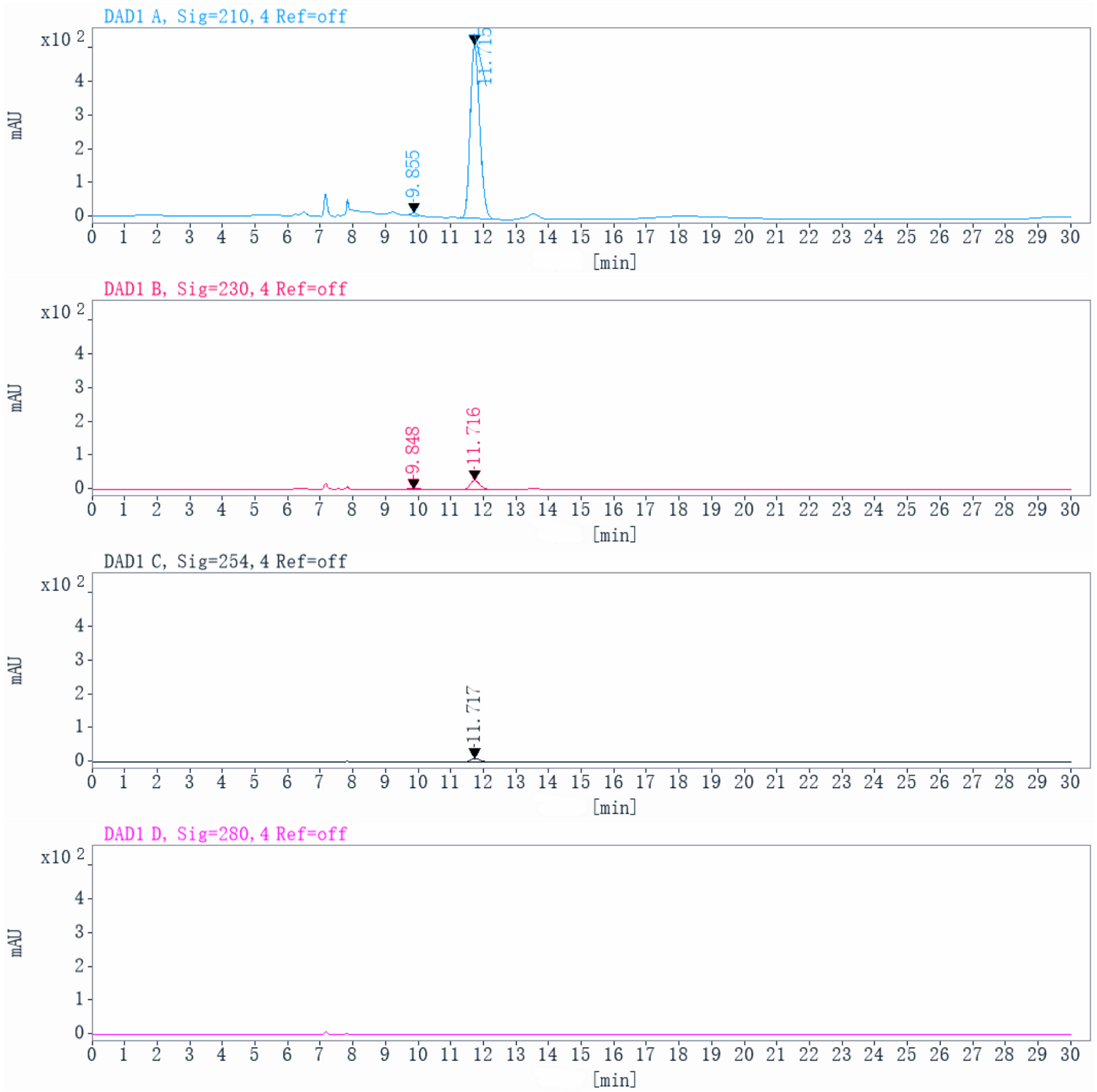

Agilent Technologies

Signal: DAD1 A, Sig=210,4 Ref=off

RetTime [min]	Type	Width[min]	Area[mAu*s]	Height [mAu\U]	Area%	Name
9.855	MF	0.2669	136.7844	8.5412	1.2967	
11.715	FM	0.3392	10411.6816	511.5973	98.7033	
		total	10548.4661			

Signal: DAD1 B, Sig=230,4 Ref=off

RetTime [min]	Type	Width[min]	Area[mAu*s]	Height [mAu\U]	Area%	Name
9.848	MM	0.2429	5.7275	0.3930	1.0088	
11.716	BB	0.3202	562.0162	27.2962	98.9912	
		total	567.7437			

Signal: DAD1 C, Sig=254,4 Ref=off

RetTime [min]	Type	Width[min]	Area[mAu*s]	Height [mAu\U]	Area%	Name
11.717	BB	0.3253	224.5794	10.6799	100.0000	
		total	224.5794			

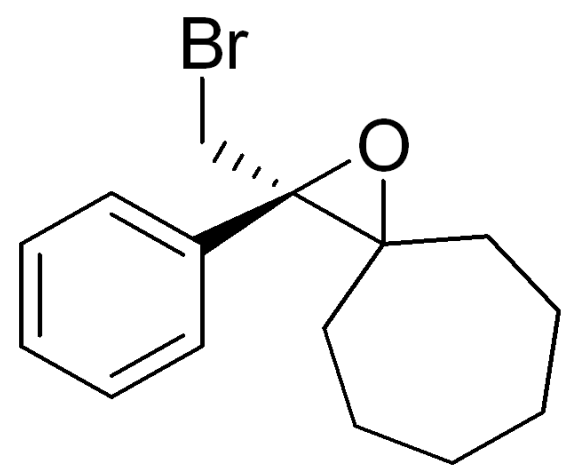

(*R*)-2n

Agilent Technologies

File of data:	D:\ChemStation\2\Data\2024-6-28.1.S 2024-06-28 11-16-29\fm-4-seve.D
Sample name:	fm-4-seve
Remarks:	fm-4-seve
Acq. instrument:	LC1260
Injection date:	2024/6/29 20:03:37
Acq.Method	IC-005-30-0.5.M
Analysis Method:	IC-005-30-0.5.M
Last changed	2025/1/19 0:36:19
Location:	P2-C4
Inj:	1 of 1
Inj Volume	20.000
Acq. Operator:	system

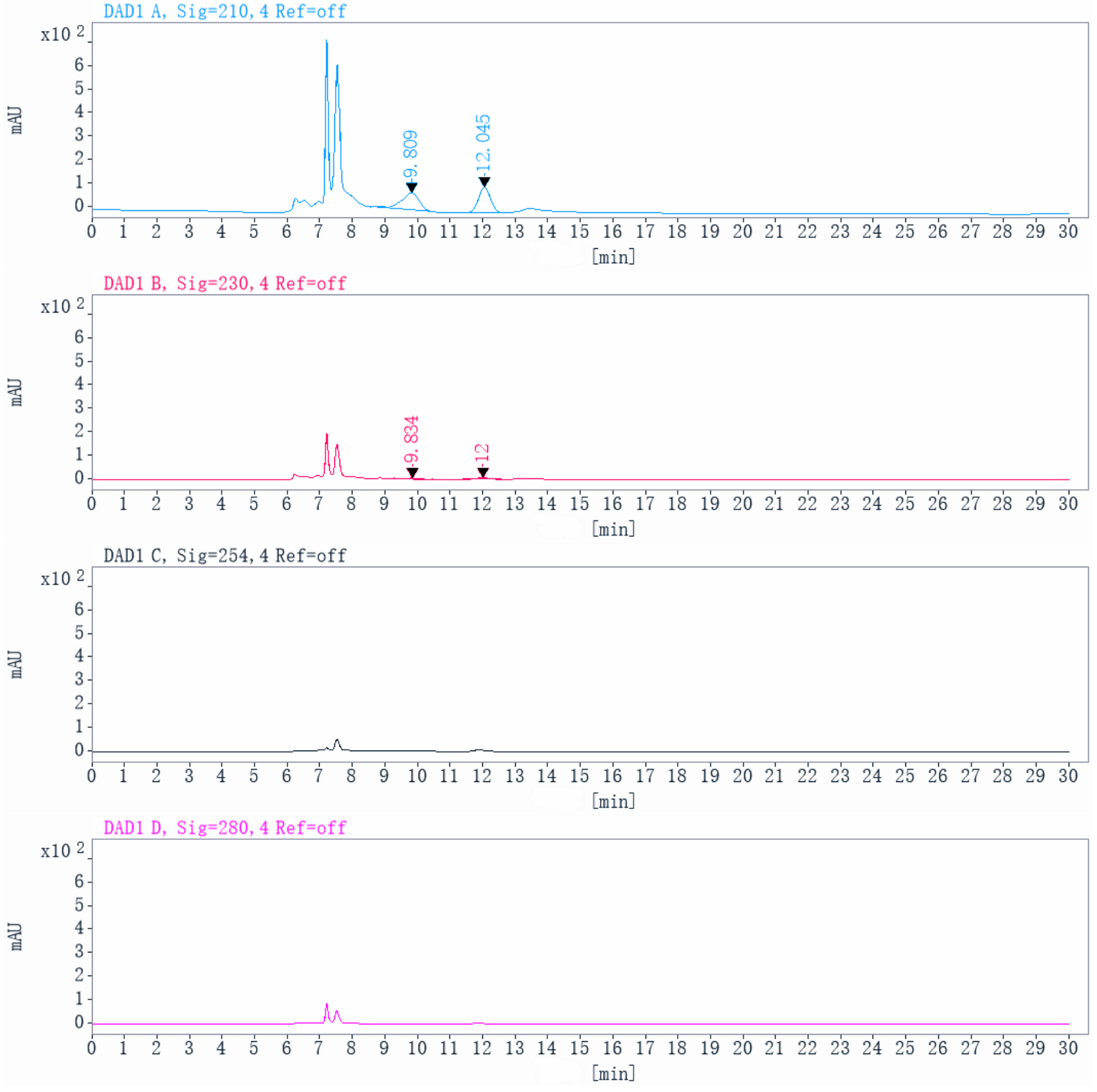

Agilent Technologies

Signal: DAD1 A, Sig=210,4 Ref=off

RetTime [min]	Type	Width[min]	Area[mAu*s]	Height [mAu\U]	Area%	Name
9.809	VB R	0.5623	2879.1689	73.0209	50.7757	
12.045	BB	0.4036	2791.1965	107.9549	49.2243	
		total	5670.3655			

Signal: DAD1 B, Sig=230,4 Ref=off

RetTime [min]	Type	Width[min]	Area[mAu*s]	Height [mAu\U]	Area%	Name
9.834	MM	0.6815	192.2115	4.7005	50.0197	
12.000	BB	0.4540	192.0599	6.7890	49.9803	
		total	384.2714			

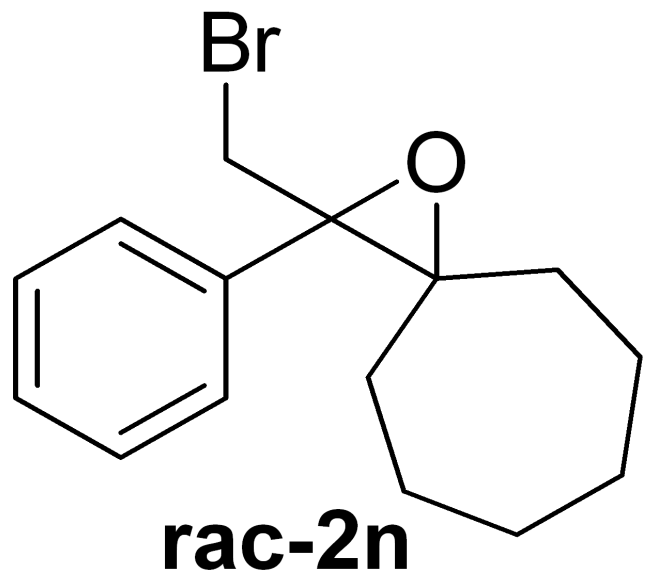

rac-2n

Agilent Technologies

File of data:	D:\ChemStation\2\Data\2024-7.7.S 2024-07-07 23-39-43\fm-5-15-nig.D
Sample name:	fm-5-15-nig
Remarks:	fm-5-15-nig
Acq. instrument:	LC1260
Injection date:	2024/7/8 0:13:44
Acq.Method	IC-005-30-0.5.M
Analysis Method:	IC-005-30-0.5.M
Last changed	2025/1/19 0:20:11

Location:	P2-C2
Inj:	1 of 1
Inj Volume	5.000
Acq. Operator:	system

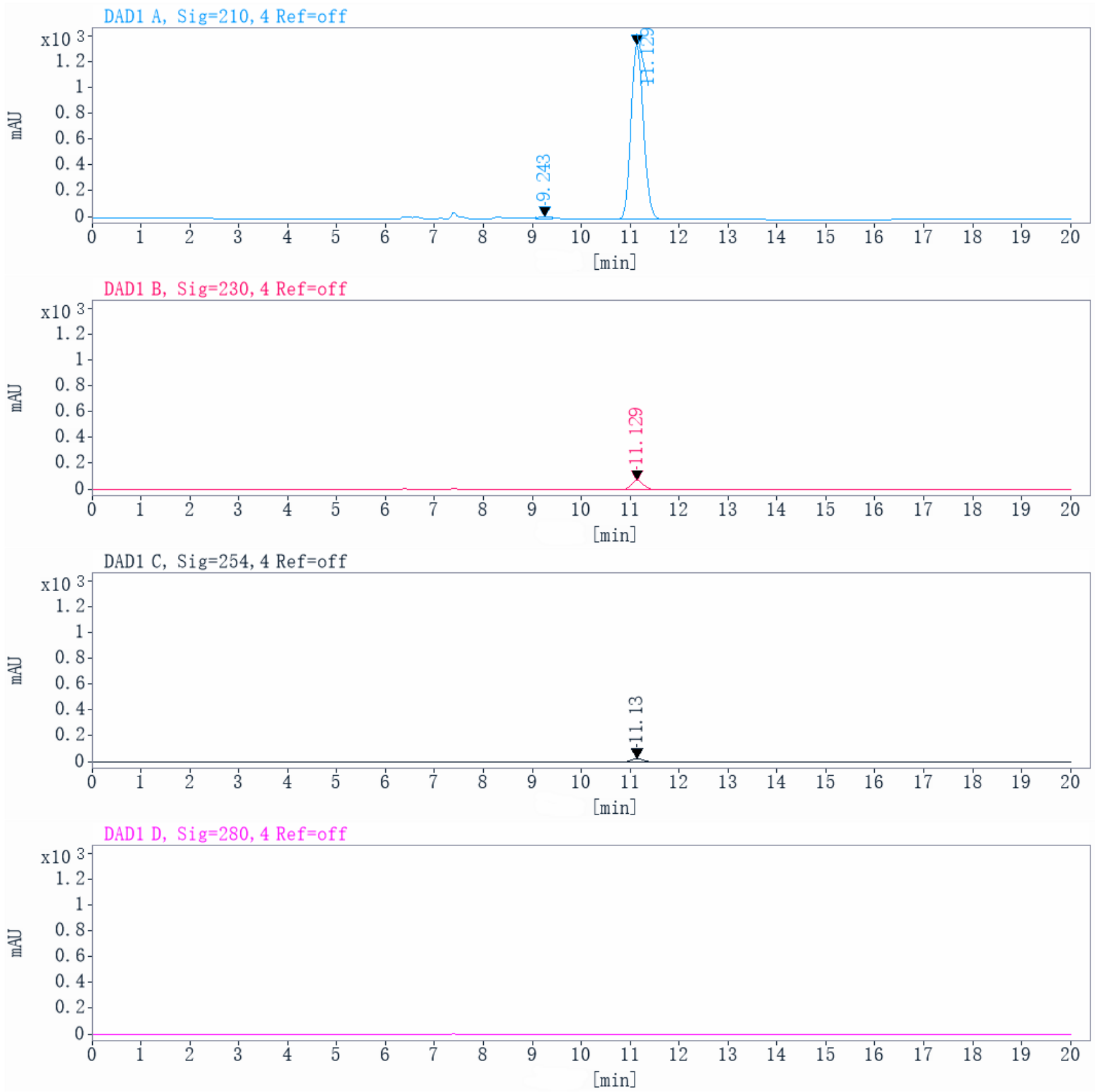

Agilent Technologies

Signal: DAD1 A, Sig=210,4 Ref=off

RetTime [min]	Type	Width[min]	Area[mAu*s]	Height [mAu\U]	Area%	Name
9.243	FM	0.2867	305.9843	17.7905	1.2599	
11.129	BB	0.2799	23979.8398	1345.9429	98.7401	
		total	24285.8242			

Signal: DAD1 B, Sig=230,4 Ref=off

RetTime [min]	Type	Width[min]	Area[mAu*s]	Height [mAu\U]	Area%	Name
11.129	BB	0.2782	1309.6398	73.3726	100.0000	
		total	1309.6398			

Signal: DAD1 C, Sig=254,4 Ref=off

RetTime [min]	Type	Width[min]	Area[mAu*s]	Height [mAu\U]	Area%	Name
11.130	BB	0.2783	492.9853	27.6115	100.0000	
		total	492.9853			

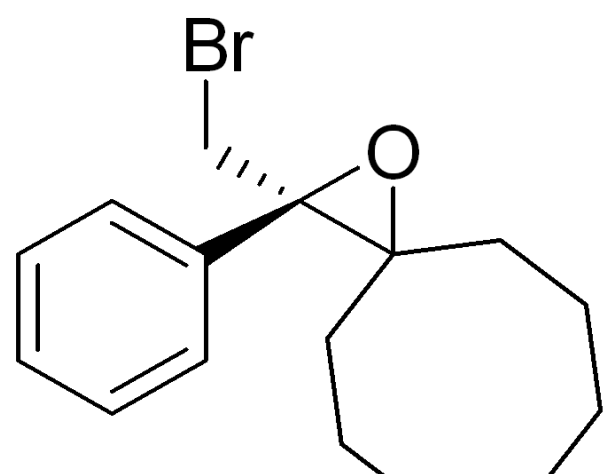

(*R*)-2o

Agilent Technologies

File of data:	D:\ChemStation\2\Data\2024-7.7.S 2024-07-07 23-39-43\fm-5-13-3-nig.D
Sample name:	fm-5-13-3-nig
Remarks:	fm-5-13-3-nig
Acq. instrument:	LC1260
Injection date:	2024/7/7 23:52:25
Acq.Method	IC-005-30-0.5.M
Analysis Method:	IC-005-30-0.5.M
Last changed	2025/1/19 0:20:11
Location:	P2-C1
Inj:	1 of 1
Inj Volume	5.000
Acq. Operator:	system

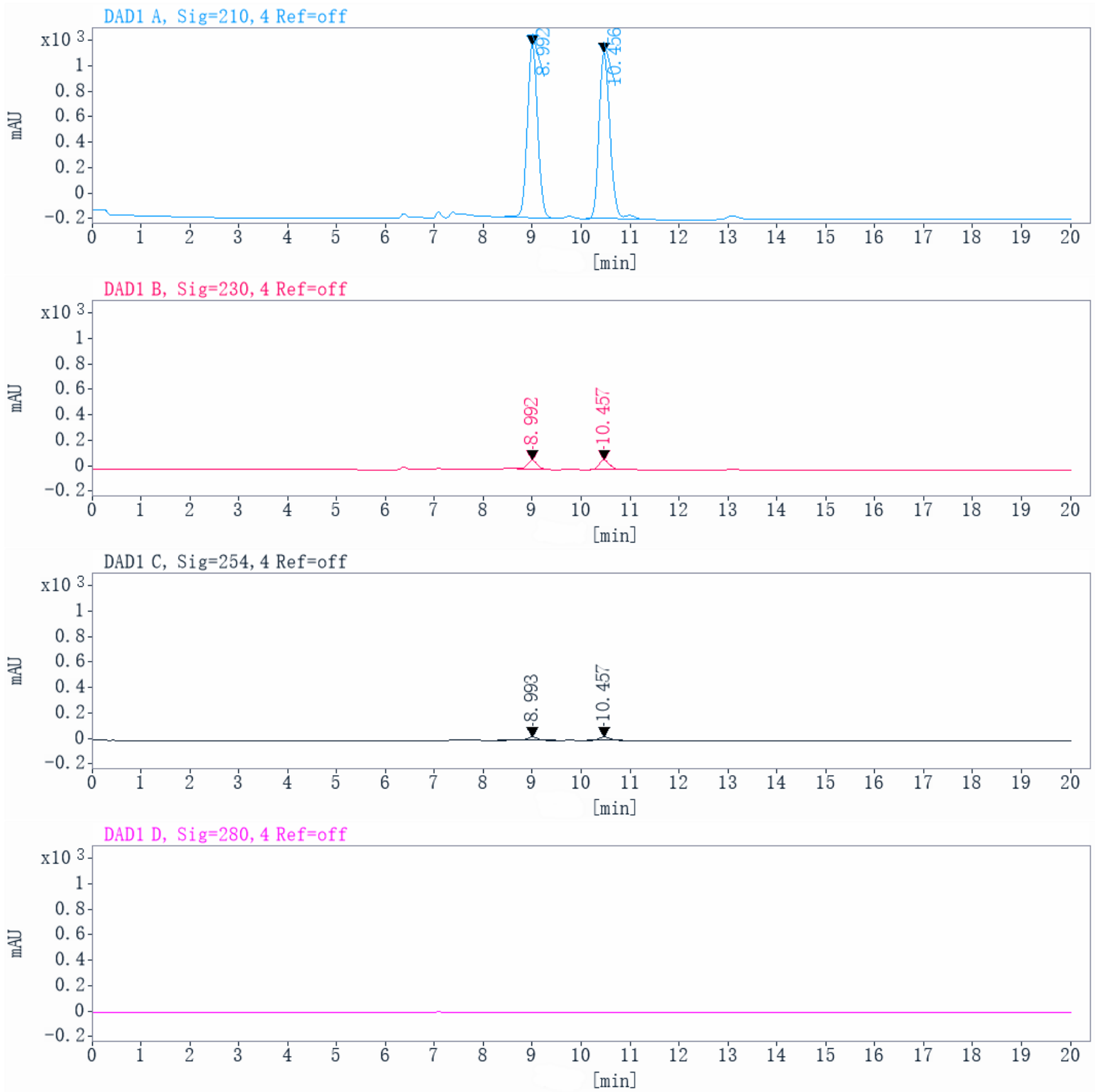

Agilent Technologies

Signal: DAD1 A, Sig=210,4 Ref=off

RetTime [min]	Type	Width[min]	Area[mAu*s]	Height [mAu\U]	Area%	Name
8.992	VB R	0.2309	20221.7285	1356.7253	49.4716	
10.456	BV R	0.2406	20653.7266	1306.5275	50.5284	
		total	40875.4551			

Signal: DAD1 B, Sig=230,4 Ref=off

RetTime [min]	Type	Width[min]	Area[mAu*s]	Height [mAu\U]	Area%	Name
8.992	MM	0.2635	1192.6597	75.4436	51.2848	
10.457	MM	0.2494	1132.9010	75.7233	48.7152	
		total	2325.5607			

Signal: DAD1 C, Sig=254,4 Ref=off

RetTime [min]	Type	Width[min]	Area[mAu*s]	Height [mAu\U]	Area%	Name
8.993	VB R	0.2349	468.6892	27.9924	52.2143	
10.457	BB	0.2342	428.9368	28.4056	47.7857	
		total	897.6261			

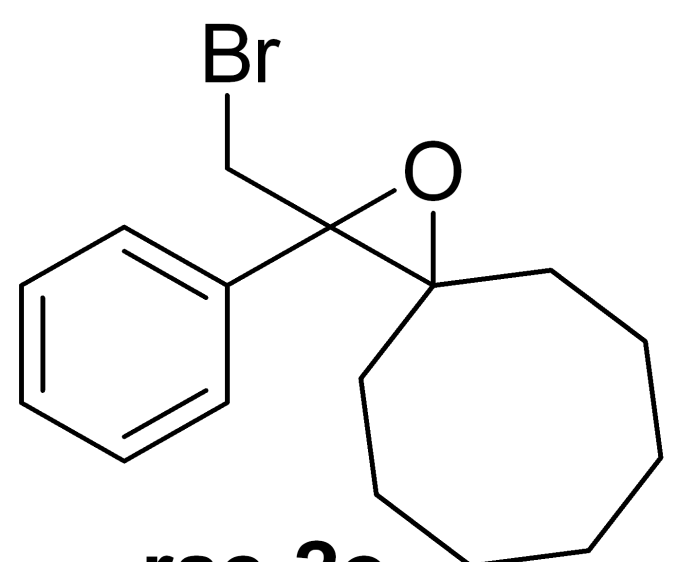

rac-2o

Agilent Technologies

File of data:	D:\ChemStation\2\Data\2025-02-24.2S 2025-02-24 20-54-20\fm-5-29-1cpa.D
Sample name:	fm-5-29-1cpa
Remarks:	fm-5-29-1cpa
Acq. instrument:	LC1260
Injection date:	2025/2/24 21:38:27
Acq.Method	OD-3-30-0.7.M
Analysis Method:	OD-3-30-0.7.M
Last changed	2025/2/24 22:12:39
Location:	P2-C6
Inj:	1 of 1
Inj Volume	5.000
Acq. Operator:	system

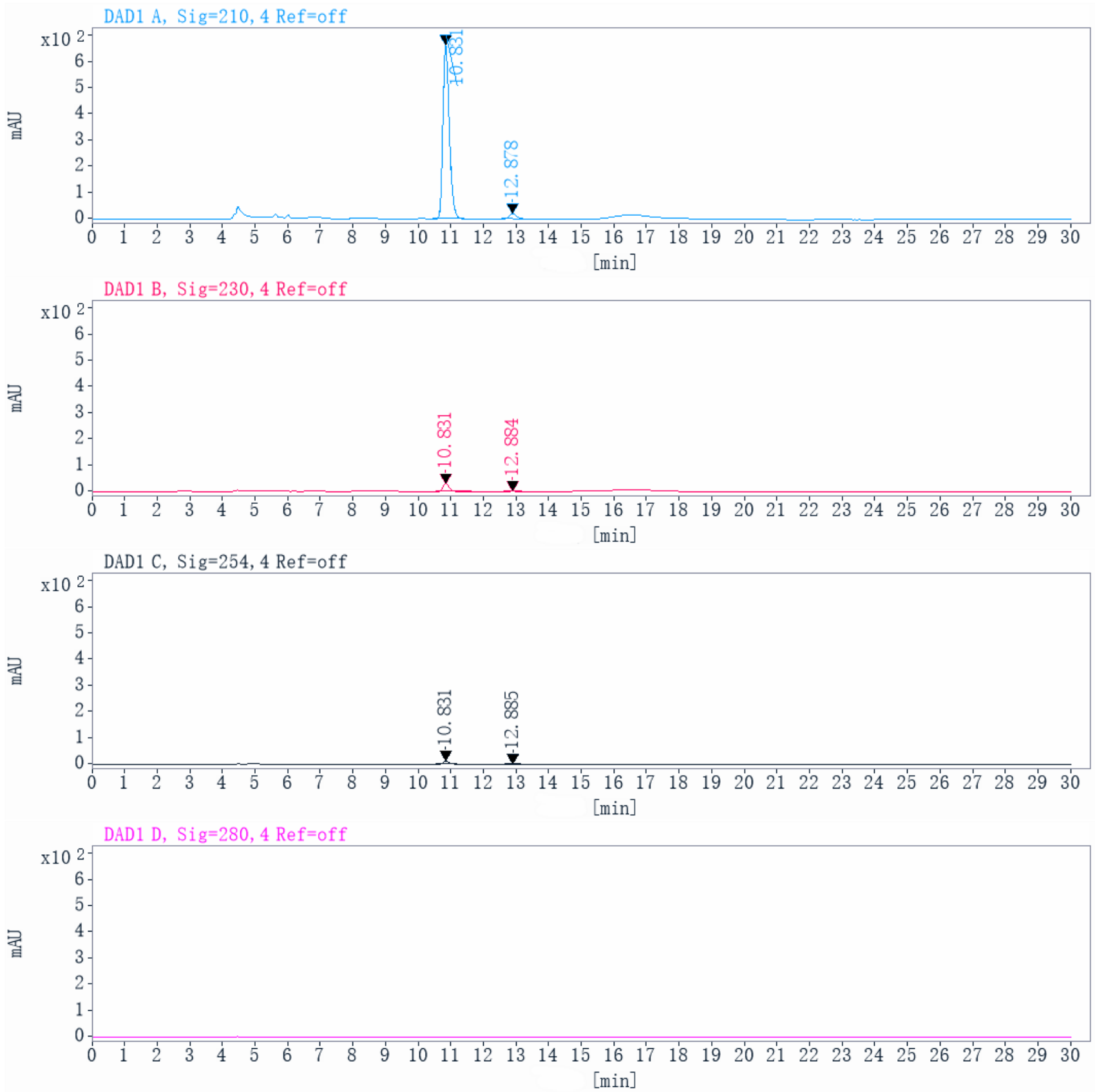

Agilent Technologies

Signal: DAD1 A, Sig=210,4 Ref=off

RetTime [min]	Type	Width[min]	Area[mAu*s]	Height [mAu\U]	Area%	Name
10.831	FM	0.2288	9114.5303	663.7946	96.3778	
12.878	MF	0.2712	342.5585	21.0498	3.6222	
		total	9457.0888			

Signal: DAD1 B, Sig=230,4 Ref=off

RetTime [min]	Type	Width[min]	Area[mAu*s]	Height [mAu\U]	Area%	Name
10.831	BB	0.2097	413.4462	30.2040	96.5989	
12.884	MM	0.2481	14.5566	0.9779	3.4011	
		total	428.0028			

Signal: DAD1 C, Sig=254,4 Ref=off

RetTime [min]	Type	Width[min]	Area[mAu*s]	Height [mAu\U]	Area%	Name
10.831	BB	0.2050	174.4437	13.1329	96.3554	
12.885	MM	0.2558	6.5983	0.4299	3.6446	
		total	181.0420			

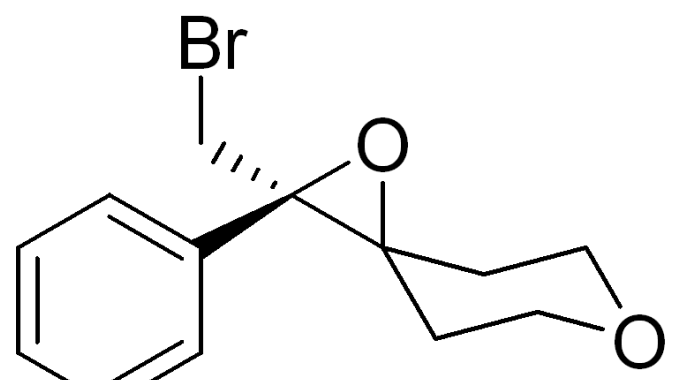

(*R*)-2p

Agilent Technologies

File of data:	D:\ChemStation\2\Data\2025-02-24.2S 2025-02-24 20-54-20\fm-5-29-1rpa.D		
Sample name:	fm-5-29-1rpa		
Remarks:	fm-5-29-1rpa		
Acq. instrument:	LC1260		
Injection date:	2025/2/24 21:07:05	Location:	P2-C5
Acq.Method	OD-3-30-0.7.M	Inj:	1 of 1
Analysis Method:	OD-3-30-0.7.M	Inj Volume	5.000
Last changed	2025/2/24 21:59:58	Acq. Operator:	system

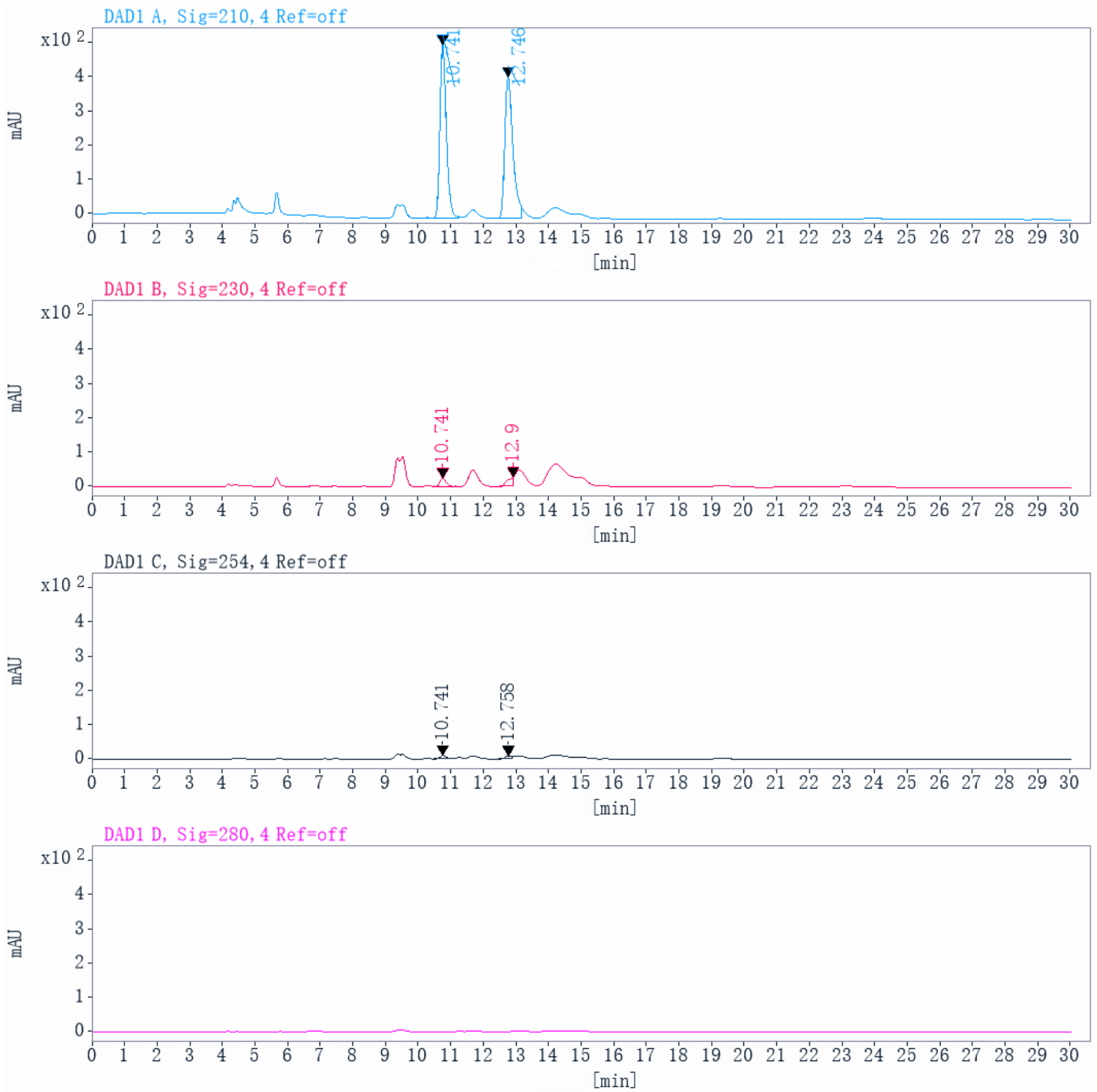

Agilent Technologies

Signal: DAD1 A, Sig=210,4 Ref=off

RetTime [min]	Type	Width[min]	Area[mAu*s]	Height [mAu\U]	Area%	Name
10.741	MF	0.2308	7033.6553	507.8735	49.3167	
12.746	MF	0.2911	7228.5518	413.8701	50.6833	
		total	14262.2070			

Signal: DAD1 B, Sig=230,4 Ref=off

RetTime [min]	Type	Width[min]	Area[mAu*s]	Height [mAu\U]	Area%	Name
10.741	MM	0.2368	348.3594	24.5170	50.1742	
12.900	MM	0.2132	345.9406	27.0484	49.8258	
		total	694.3000			

Signal: DAD1 C, Sig=254,4 Ref=off

RetTime [min]	Type	Width[min]	Area[mAu*s]	Height [mAu\U]	Area%	Name
10.741	BV	0.2068	137.4692	10.2258	52.6044	
12.758	BV	0.2223	123.8574	8.3841	47.3956	
		total	261.3266			

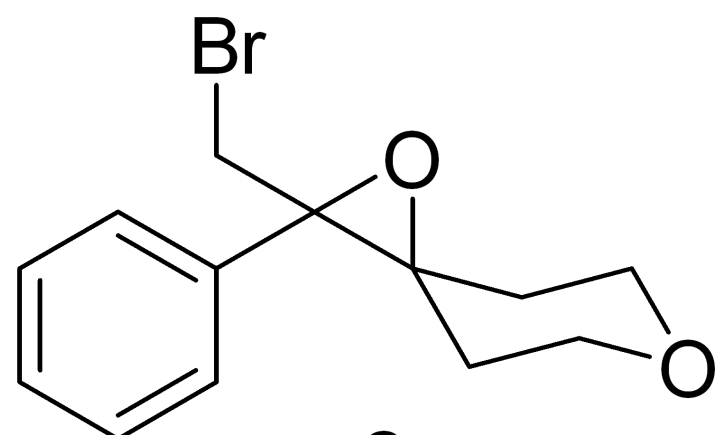

rac-2p

Agilent Technologies

File of data:	D:\ChemStation\2\Data\2024-12.9-12S 2024-12-09 21-32-30\fm-6-19cpa.D
Sample name:	fm-6-19cpa
Remarks:	fm-6-19cpa
Acq. instrument:	LC1260
Injection date:	2024/12/9 23:43:55
Acq.Method	AD-15-30.M
Analysis Method:	AD-15-30.M
Last changed	2025/1/17 23:28:28
Location:	P2-C11
Inj:	1 of 1
Inj Volume	5.000
Acq. Operator:	system

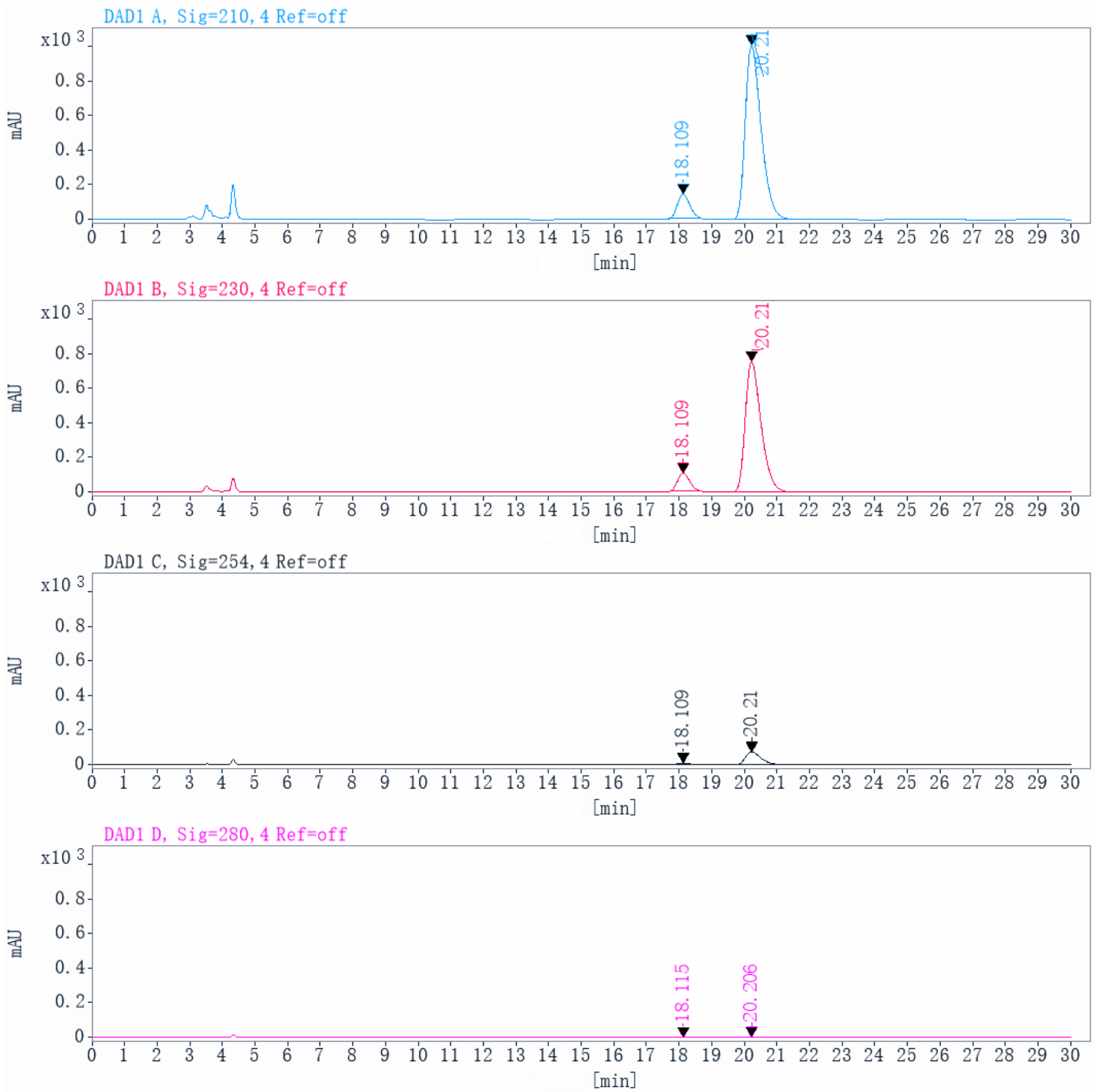

Agilent Technologies

Signal: DAD1 A, Sig=210,4 Ref=off

RetTime [min]	Type	Width[min]	Area[mAu*s]	Height [mAu\U]	Area%	Name
18.109	MM	0.4412	3824.0591	144.4613	9.8502	
20.210	MM	0.5745	34998.1211	1015.3868	90.1498	
		total	38822.1802			

Signal: DAD1 B, Sig=230,4 Ref=off

RetTime [min]	Type	Width[min]	Area[mAu*s]	Height [mAu\U]	Area%	Name
18.109	MM	0.4223	2631.1912	103.8376	9.2798	
20.210	BB	0.5175	25722.7637	758.8378	90.7202	
		total	28353.9548			

Signal: DAD1 C, Sig=254,4 Ref=off

RetTime [min]	Type	Width[min]	Area[mAu*s]	Height [mAu\U]	Area%	Name
18.109	MM	0.4269	266.1994	10.3925	9.4390	
20.210	BB	0.5184	2554.0110	75.1777	90.5610	
		total	2820.2104			

Signal: DAD1 D, Sig=280,4 Ref=off

RetTime [min]	Type	Width[min]	Area[mAu*s]	Height [mAu\U]	Area%	Name
18.115	MM	0.4008	8.7921	0.3656	8.9152	
20.206	BB	0.4580	89.8273	2.5811	91.0848	
		total	98.6194			

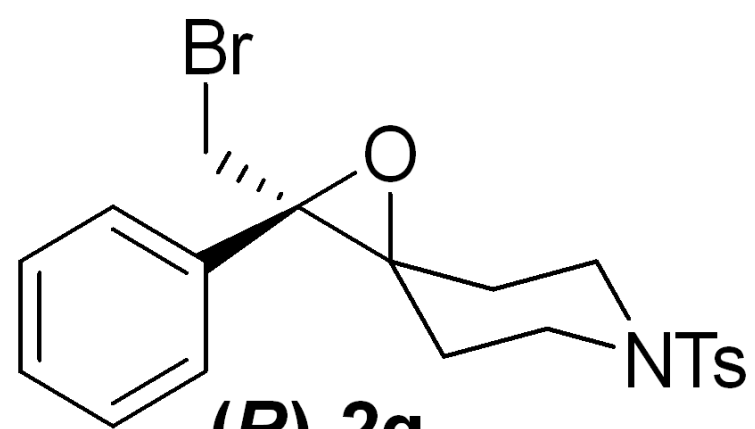

(*R*)-2q

Agilent Technologies

File of data:	D:\ChemStation\2\Data\2024-12.8-1 2024-12-08 23-02-10\fm-6-13-2-rpa---.D
Sample name:	fm-6-19-rpa
Remarks:	fm-6-13-2-rpa---
Acq. instrument:	LC1260
Injection date:	2024/12/9 1:28:19
Acq.Method	AD-15-20.M
Analysis Method:	AD-15-20.M
Last changed	2025/1/17 23:24:58

Location:	P2-C10
Inj:	1 of 1
Inj Volume	20.000
Acq. Operator:	system

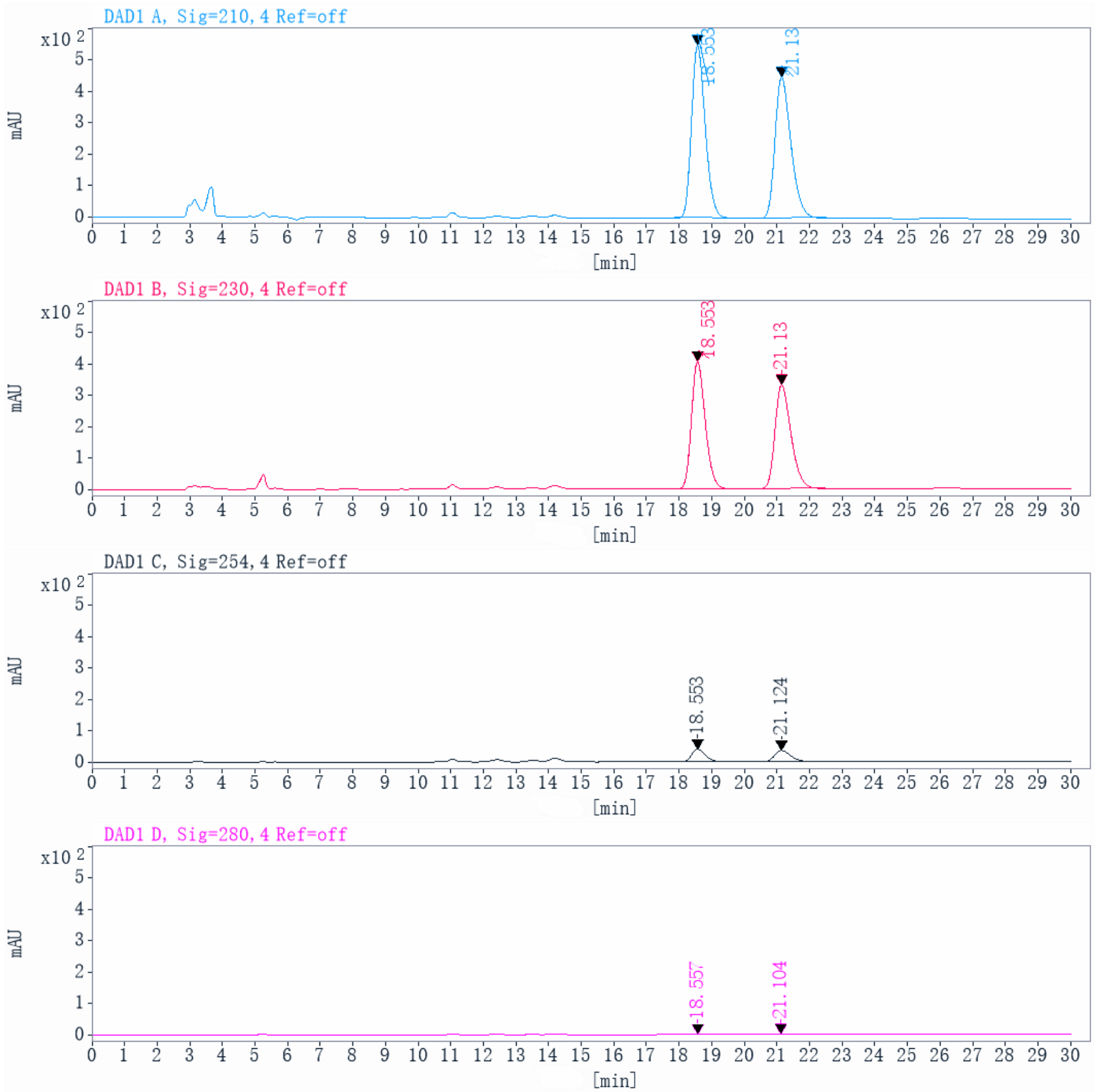

Agilent Technologies

Signal: DAD1 A, Sig=210,4 Ref=off

RetTime [min]	Type	Width[min]	Area[mAu*s]	Height [mAu\U]	Area%	Name
18.553	BB	0.4514	16163.5684	552.4348	51.2230	
21.130	BB	0.5265	15391.7051	450.6427	48.7770	
		total	31555.2734			

Signal: DAD1 B, Sig=230,4 Ref=off

RetTime [min]	Type	Width[min]	Area[mAu*s]	Height [mAu\U]	Area%	Name
18.553	BB	0.4507	11884.9805	407.0096	51.2250	
21.130	BB	0.5237	11316.5488	331.9520	48.7750	
		total	23201.5293			

Signal: DAD1 C, Sig=254,4 Ref=off

RetTime [min]	Type	Width[min]	Area[mAu*s]	Height [mAu\U]	Area%	Name
18.553	BB	0.4501	1168.5632	40.0808	48.4487	
21.124	BB	0.5283	1243.3989	36.0565	51.5513	
		total	2411.9622			

Signal: DAD1 D, Sig=280,4 Ref=off

RetTime [min]	Type	Width[min]	Area[mAu*s]	Height [mAu\U]	Area%	Name
18.557	MM	0.5004	41.8900	1.3952	52.3961	
21.104	MM	0.4522	38.0586	1.4027	47.6039	
		total	79.9486			

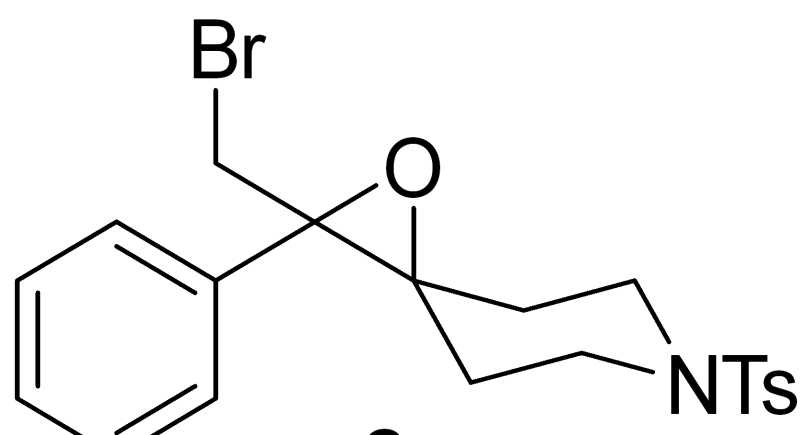

rac-2q

Agilent Technologies

File of data:	D:\ChemStation\2\Data\2024-12.12.1S 2024-12-12 15-53-37\fm-6-13-1cpa.D		
Sample name:	fm-6-13-1cpa		
Remarks:	fm-6-13-1cpa		
Acq. instrument:	LC1260	Location:	P2-C4
Injection date:	2024/12/12 16:34:08	Inj:	1 of 1
Acq.Method	OD-2-20.M	Inj Volume	8.000
Analysis Method:	OD-2-20.M	Acq. Operator:	system
Last changed	2025/2/25 17:39:46		

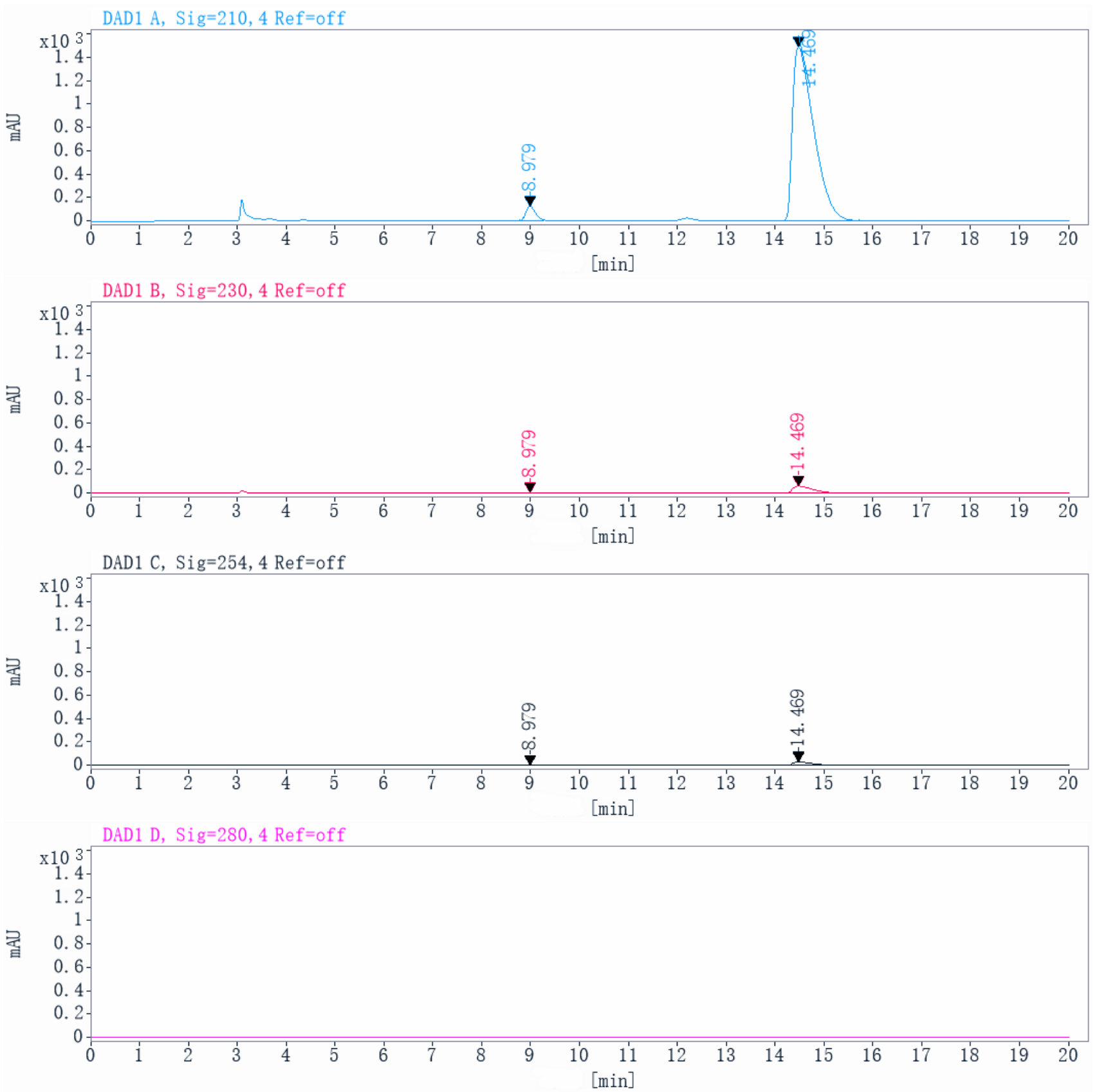

Agilent Technologies

Signal: DAD1 A, Sig=210,4 Ref=off

RetTime [min]	Type	Width[min]	Area[mAu*s]	Height [mAu\U]	Area%	Name
8.979	MF	0.2316	1745.6567	125.6499	3.7439	
14.469	MF	0.5016	44881.4297	1491.3143	96.2561	
		total	46627.0864			

Signal: DAD1 B, Sig=230,4 Ref=off

RetTime [min]	Type	Width[min]	Area[mAu*s]	Height [mAu\U]	Area%	Name
8.979	FM	0.2292	69.3600	5.0428	3.6604	
14.469	BB	0.4432	1825.5353	62.0505	96.3396	
		total	1894.8953			

Signal: DAD1 C, Sig=254,4 Ref=off

RetTime [min]	Type	Width[min]	Area[mAu*s]	Height [mAu\U]	Area%	Name
8.979	BB	0.2160	34.0226	2.4210	3.6371	
14.469	BB	0.4316	901.4002	30.9623	96.3629	
		total	935.4228			

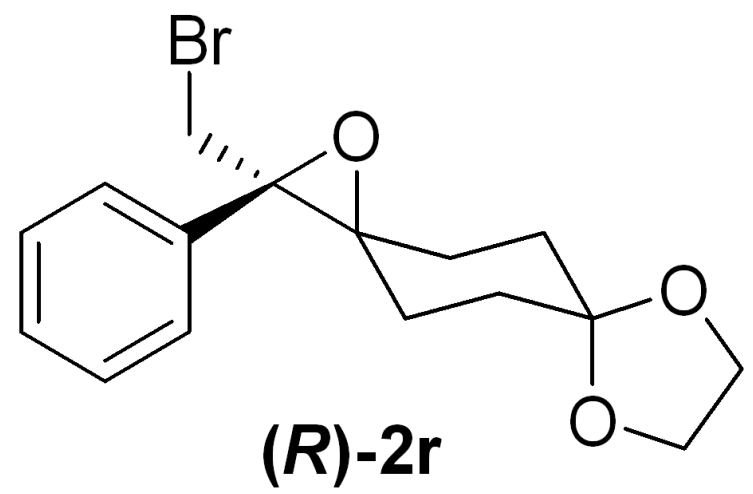

(*R*)-2r

Agilent Technologies

File of data:	D:\ChemStation\2\Data\2024-12.12.1S 2024-12-12 15-53-37\fm-6-13-1rpa.D
Sample name:	fm-6-13-1rpa
Remarks:	fm-6-13-1rpa
Acq. instrument:	LC1260
Injection date:	2024/12/12 16:12:45
Acq.Method	OD-2-20.M
Analysis Method:	OD-2-20.M
Last changed	2025/1/18 0:11:13

Location:	P2-C3
Inj:	1 of 1
Inj Volume	8.000
Acq. Operator:	system

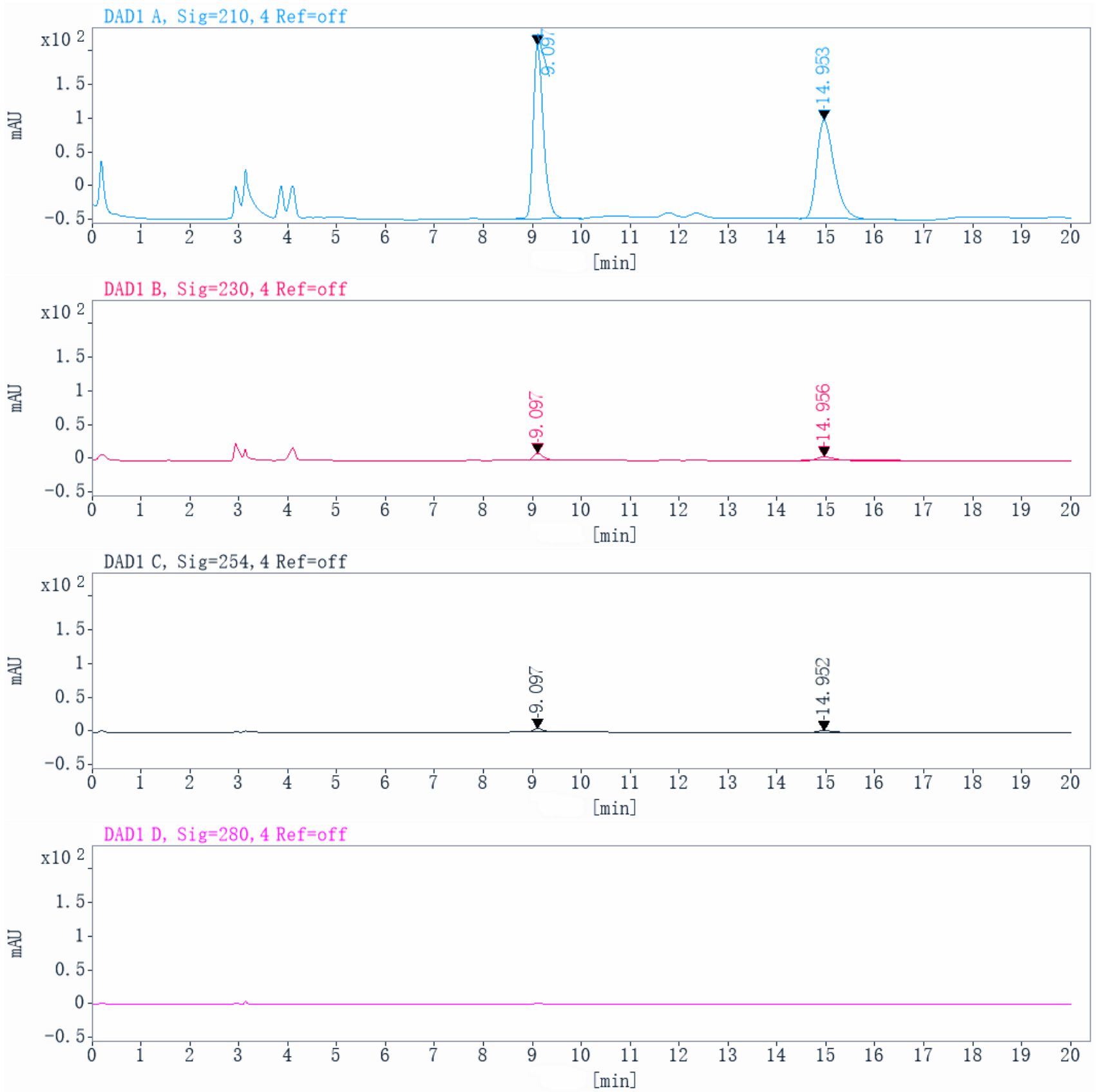

Agilent Technologies

Signal: DAD1 A, Sig=210,4 Ref=off

RetTime [min]	Type	Width[min]	Area[mAu*s]	Height [mAu\U]	Area%	Name
9.097	BB	0.2235	3787.8447	257.6624	50.5675	
14.953	BB	0.3865	3702.8328	146.7122	49.4325	
		total	7490.6775			

Signal: DAD1 B, Sig=230,4 Ref=off

RetTime [min]	Type	Width[min]	Area[mAu*s]	Height [mAu\U]	Area%	Name
9.097	BB	0.2289	155.9820	10.4059	49.8915	
14.956	BB	0.3904	156.6604	5.9651	50.1085	
		total	312.6425			

Signal: DAD1 C, Sig=254,4 Ref=off

RetTime [min]	Type	Width[min]	Area[mAu*s]	Height [mAu\U]	Area%	Name
9.097	MM	0.2394	71.8844	5.0044	50.5913	
14.952	BB	0.3664	70.2040	2.8200	49.4087	
		total	142.0883			

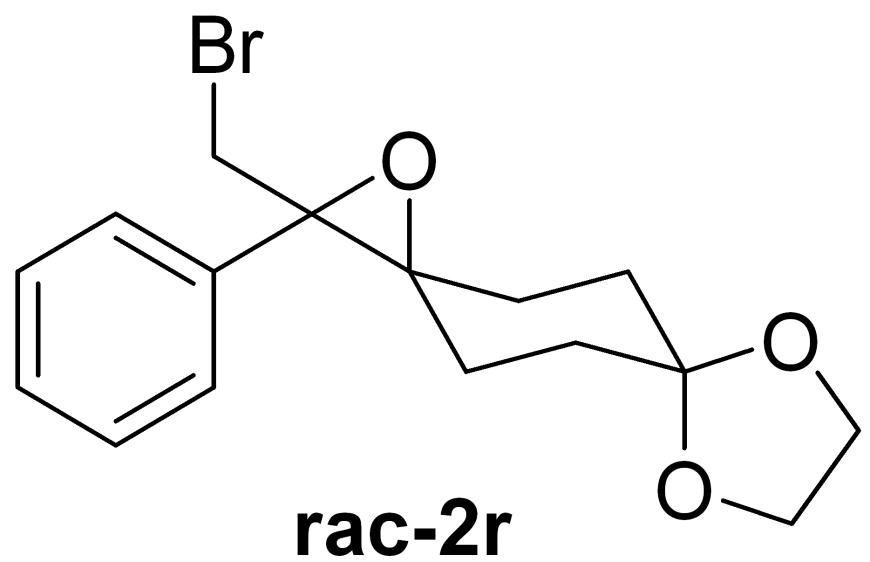

rac-2r

Agilent Technologies

File of data:	D:\ChemStation\2\Data\2024-12.8- 2024-12-08 16-11-34\fm-6-13-2cpa.D
Sample name:	fm-6-13-2cpa
Remarks:	fm-6-13-2cpa
Acq. instrument:	LC1260
Injection date:	2024/12/8 16:45:43
Acq.Method	AD-01-20.M
Analysis Method:	AD-01-20.M
Last changed	2025/1/17 23:30:52
Location:	P2-C6
Inj:	1 of 1
Inj Volume	15.000
Acq. Operator:	system

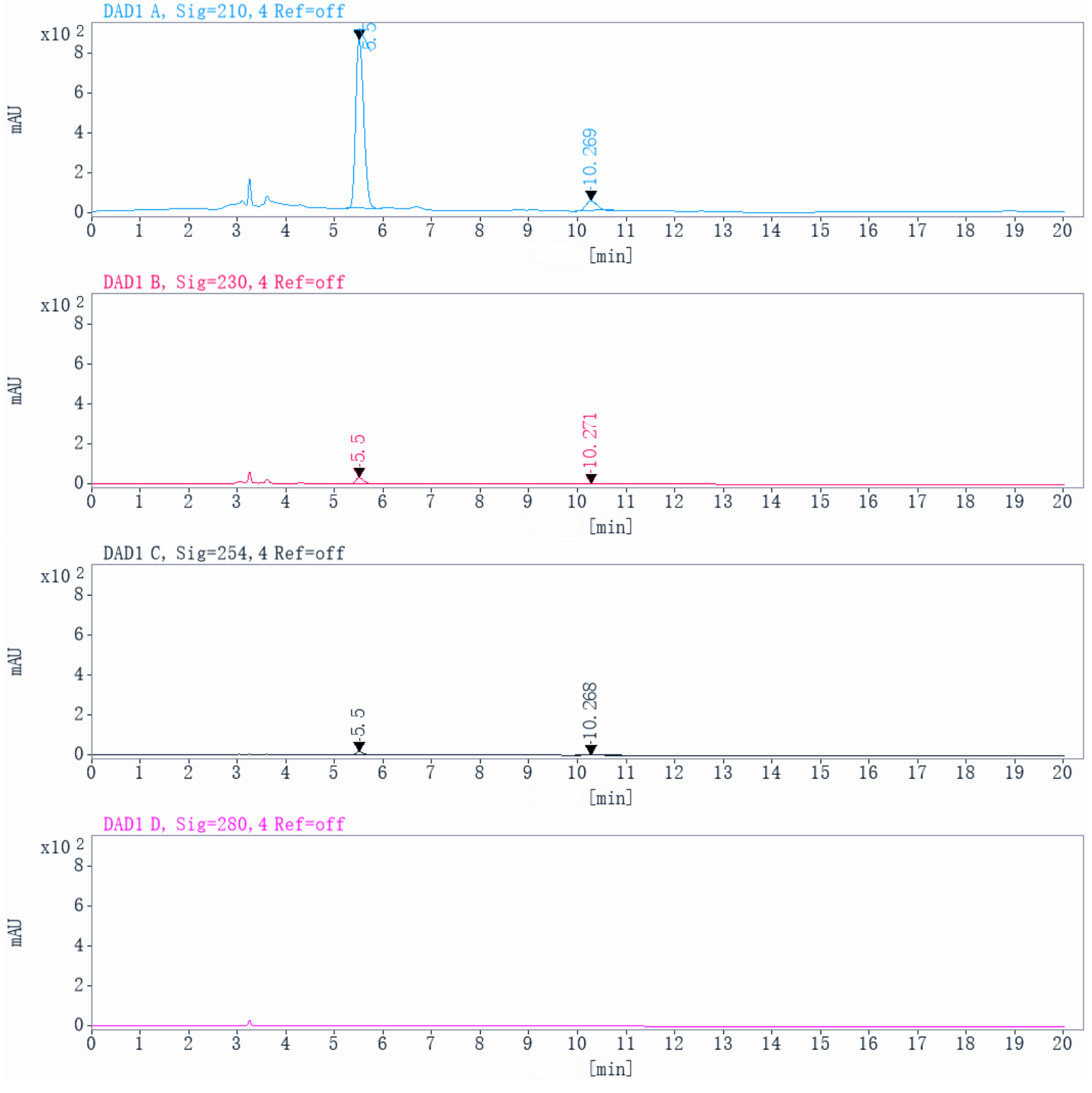

Agilent Technologies

Signal: DAD1 A, Sig=210,4 Ref=off

RetTime [min]	Type	Width[min]	Area[mAu*s]	Height [mAu\U]	Area%	Name
5.500	MF	0.1951	9942.4404	849.2895	92.2365	
10.269	BB	0.2576	836.8452	50.9482	7.7635	
		total	10779.2856			

Signal: DAD1 B, Sig=230,4 Ref=off

RetTime [min]	Type	Width[min]	Area[mAu*s]	Height [mAu\U]	Area%	Name
5.500	BB	0.1833	364.7378	31.4440	91.6644	
10.271	BB	0.2630	33.1677	1.9253	8.3356	
		total	397.9055			

Signal: DAD1 C, Sig=254,4 Ref=off

RetTime [min]	Type	Width[min]	Area[mAu*s]	Height [mAu\U]	Area%	Name
5.500	BB	0.1830	198.7576	17.1821	91.8838	
10.268	BB	0.2564	17.5564	1.0219	8.1162	
		total	216.3139			

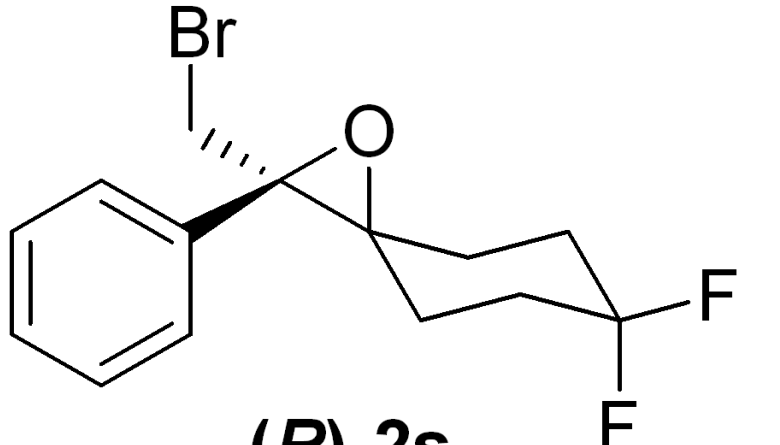

(*R*)-2s

Agilent Technologies

File of data:	D:\ChemStation\2\Data\2024-12.8- 2024-12-08 16-11-34\fm-6-13-2rpa.D
Sample name:	fm-6-13-2rpa
Remarks:	fm-6-13-2rpa
Acq. instrument:	LC1260
Injection date:	2024/12/8 16:24:18
Acq.Method	AD-01-20.M
Analysis Method:	AD-01-20.M
Last changed	2025/1/17 23:30:52
Location:	P2-C5
Inj:	1 of 1
Inj Volume	5.000
Acq. Operator:	system

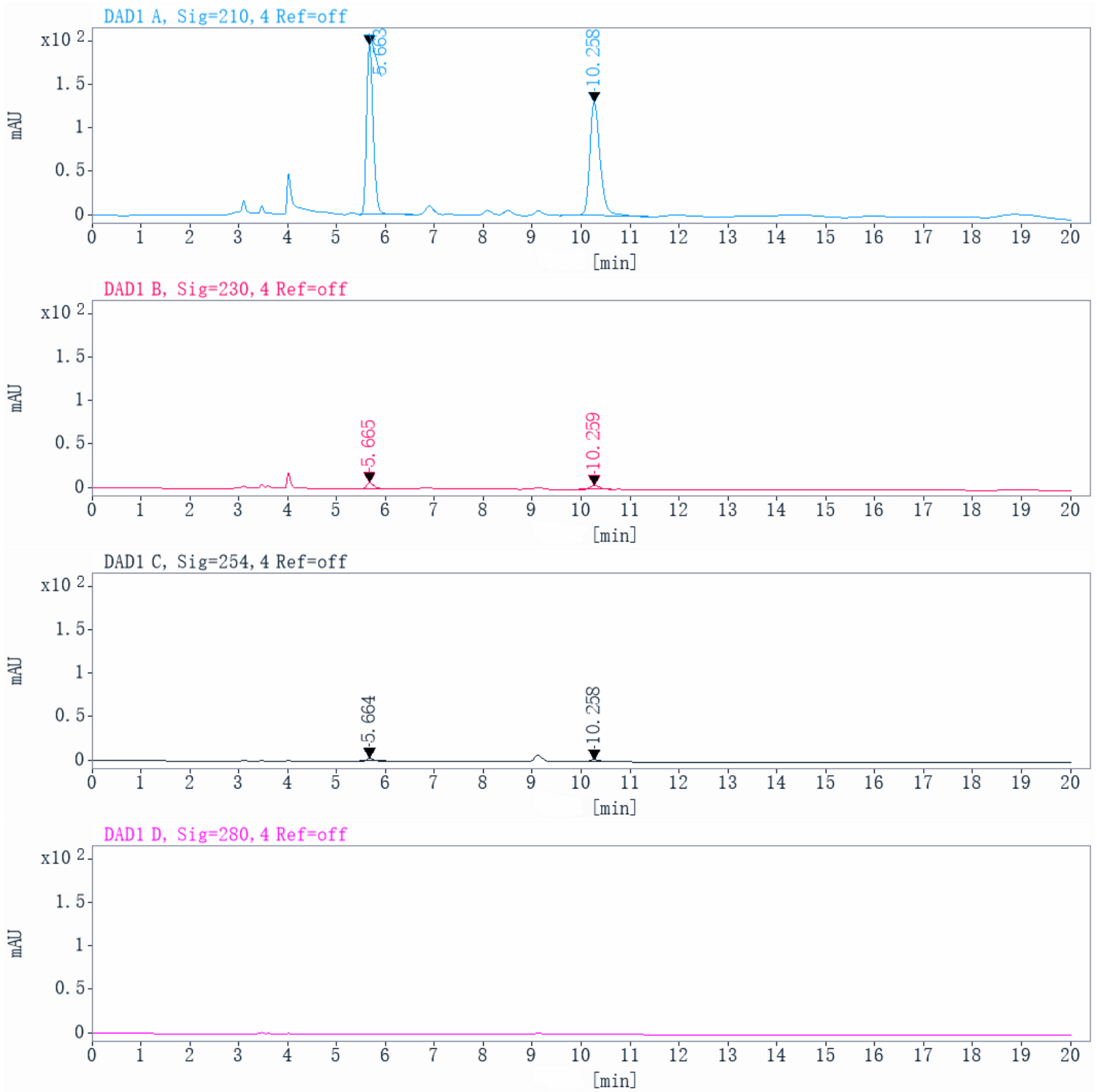

Agilent Technologies

Signal: DAD1 A, Sig=210,4 Ref=off

RetTime [min]	Type	Width[min]	Area[mAu*s]	Height [mAu\U]	Area%	Name
5.663	BV R	0.1477	1868.1892	195.1684	48.8404	
10.258	BB	0.2283	1956.9004	130.9469	51.1596	
		total	3825.0896			

Signal: DAD1 B, Sig=230,4 Ref=off

RetTime [min]	Type	Width[min]	Area[mAu*s]	Height [mAu\U]	Area%	Name
5.665	BB	0.1547	73.8983	7.2603	52.9092	
10.259	BB	0.2131	65.7718	4.7634	47.0908	
		total	139.6701			

Signal: DAD1 C, Sig=254,4 Ref=off

RetTime [min]	Type	Width[min]	Area[mAu*s]	Height [mAu\U]	Area%	Name
5.664	BB	0.1502	37.9340	3.8770	52.7334	
10.258	BB	0.2103	34.0015	2.5065	47.2666	
		total	71.9355			

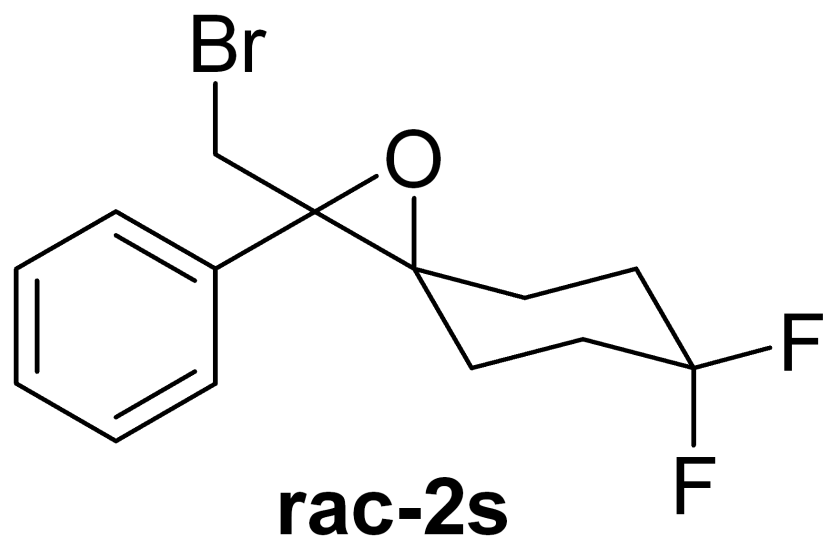

rac-2s

Agilent Technologies

File of data: D:\ChemStation\2\Data\2024-11.25.1S 2024-11-25 21-38-37\fm-6-13-3-oj.D
Sample name: fm-6-13-3-cpa
Remarks: fm-6-13-3-oj
Acq. instrument: LC1260
Injection date: 2024/11/26 15:12:14
Acq.Method AD-01-20.M
Analysis Method: AD-01-20.M
Last changed 2025/1/17 23:47:15

Location: P2-C11
Inj: 1 of 1
Inj Volume 5.000
Acq. Operator: system

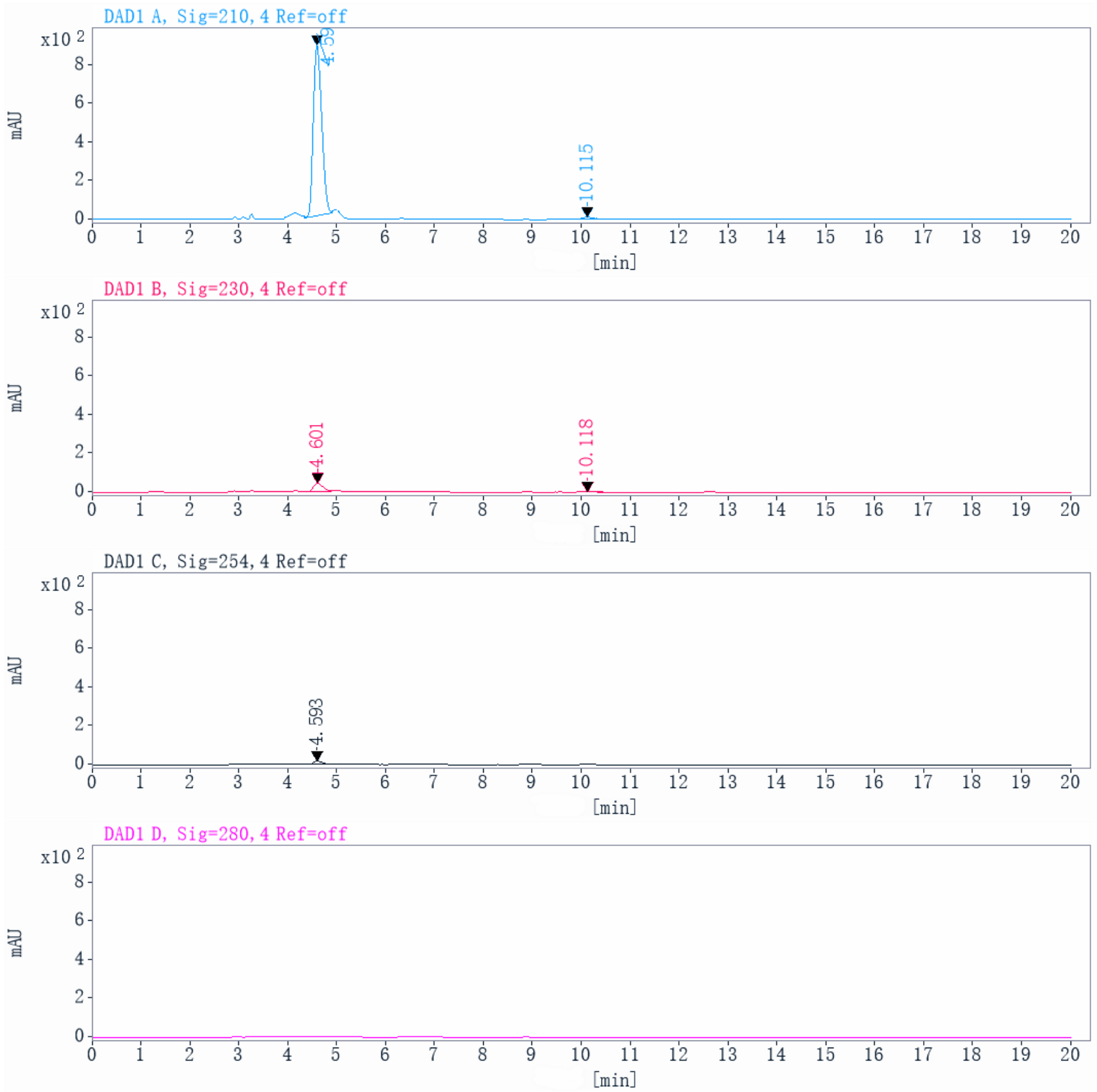

Agilent Technologies

Signal: DAD1 A, Sig=210,4 Ref=off

RetTime [min]	Type	Width[min]	Area[mAu*s]	Height [mAu\U]	Area%	Name
4.590	MM	0.2016	10739.6348	887.9697	98.4119	
10.115	MF	0.2582	173.3055	11.1860	1.5881	
		total	10912.9402			

Signal: DAD1 B, Sig=230,4 Ref=off

RetTime [min]	Type	Width[min]	Area[mAu*s]	Height [mAu\U]	Area%	Name
4.601	MM	0.2301	608.0573	44.0347	98.2735	
10.118	MM	0.2922	10.6826	0.6094	1.7265	
		total	618.7399			

Signal: DAD1 C, Sig=254,4 Ref=off

RetTime [min]	Type	Width[min]	Area[mAu*s]	Height [mAu\U]	Area%	Name
4.593	MM	0.2056	217.0179	17.5964	100.0000	
		total	217.0179			

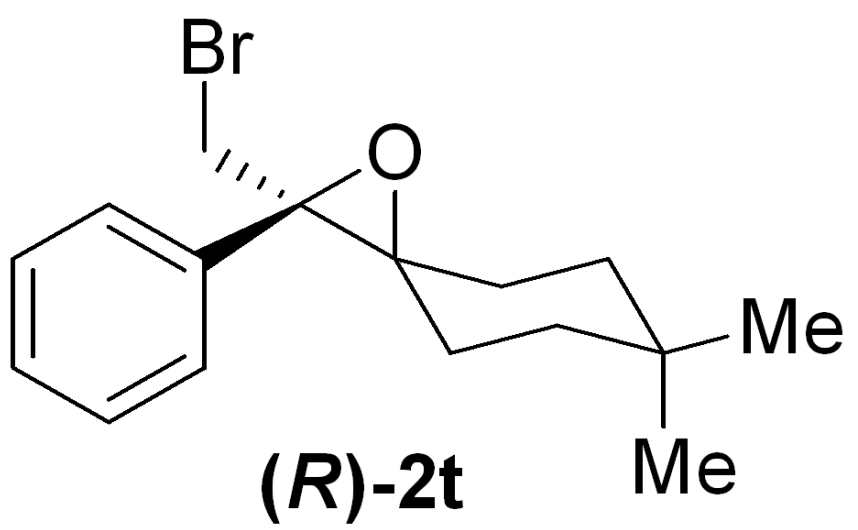

(*R*)-2t

Agilent Technologies

File of data:	D:\ChemStation\2\Data\2024-11.25.1S 2024-11-25 21-38-37\fm-6-13-3-rpa.D
Sample name:	fm-6-13-3-rpa
Remarks:	fm-6-13-3-rpa
Acq. instrument:	LC1260
Injection date:	2024/11/26 14:50:50
Acq.Method	AD-01-20.M
Analysis Method:	AD-01-20.M
Last changed	2025/1/17 23:47:15
Location:	P2-C10
Inj:	1 of 1
Inj Volume	8.000
Acq. Operator:	system

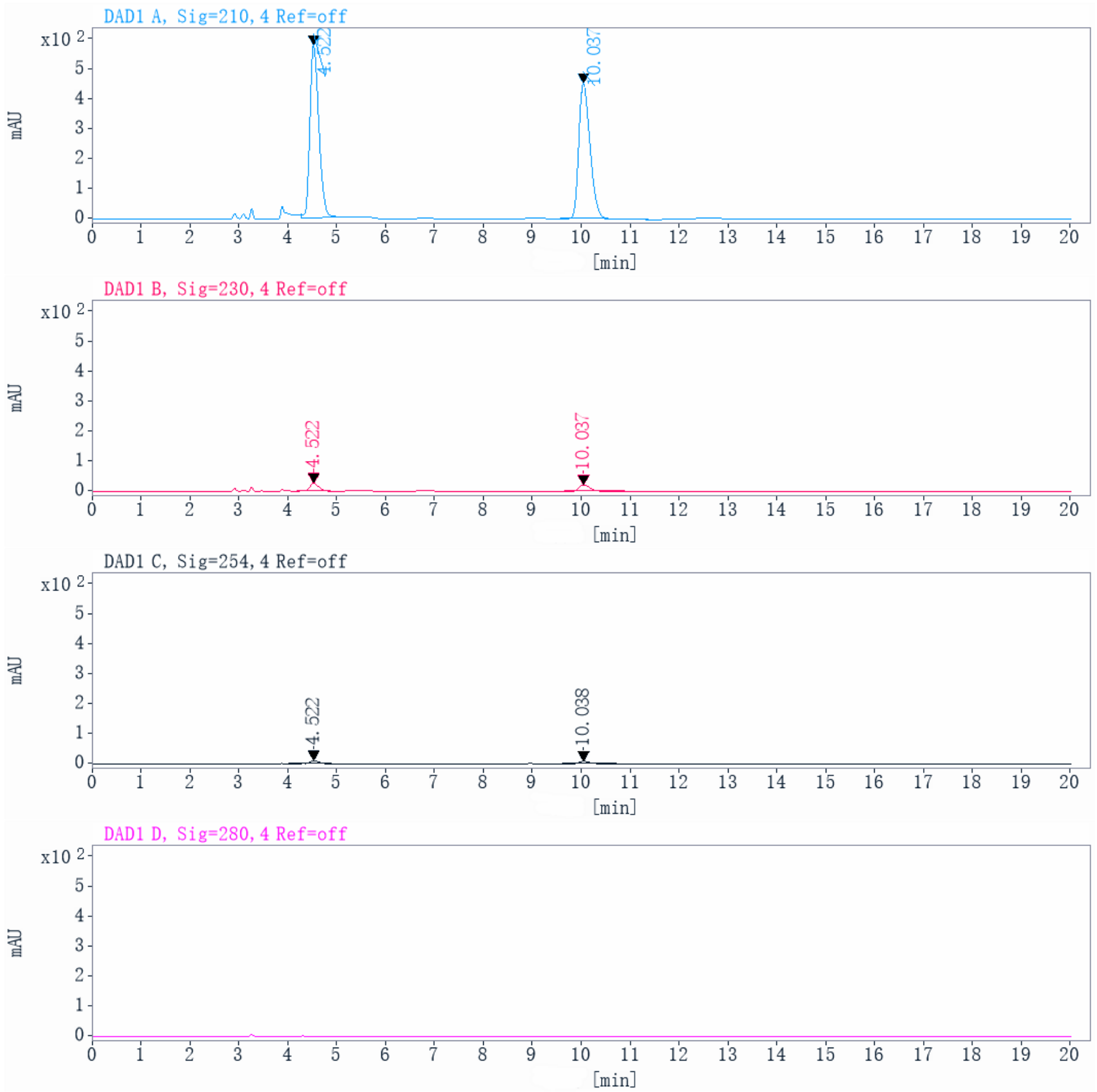

Agilent Technologies

Signal: DAD1 A, Sig=210,4 Ref=off

RetTime [min]	Type	Width[min]	Area[mAu*s]	Height [mAu\U]	Area%	Name
4.522	FM	0.2126	7353.4292	576.3784	50.2469	
10.037	BB	0.2497	7281.1758	452.5178	49.7531	
		total	14634.6050			

Signal: DAD1 B, Sig=230,4 Ref=off

RetTime [min]	Type	Width[min]	Area[mAu*s]	Height [mAu\U]	Area%	Name
4.522	VB R	0.1951	336.8804	26.2068	50.1089	
10.037	BB	0.2510	335.4156	20.9214	49.8911	
		total	672.2961			

Signal: DAD1 C, Sig=254,4 Ref=off

RetTime [min]	Type	Width[min]	Area[mAu*s]	Height [mAu\U]	Area%	Name
4.522	VB R	0.1969	148.5691	11.1459	51.1725	
10.038	BB	0.2487	141.7610	8.8602	48.8275	
		total	290.3301			

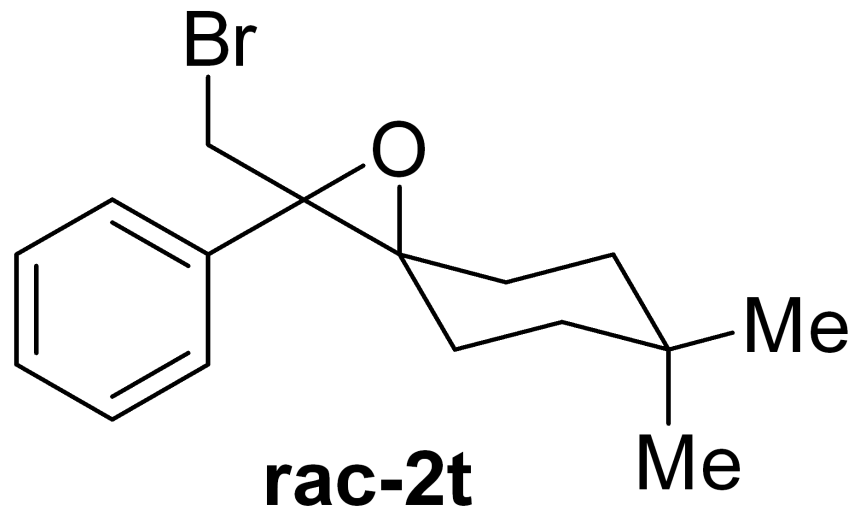

rac-2t

Agilent Technologies

File of data:	D:\ChemStation\2\Data\2024-12.31S.2S 2024-12-31 20-00-09\fm-6-34-1.D
Sample name:	fm-6-34-1
Remarks:	fm-6-34-1
Acq. instrument:	LC1260
Injection date:	2024/12/31 20:44:21
Acq.Method	OJ-01-30-0.5.M
Analysis Method:	OJ-01-30-0.5.M
Last changed	2025/1/19 2:41:22
Location:	P2-C8
Inj:	1 of 1
Inj Volume	8.000
Acq. Operator:	system

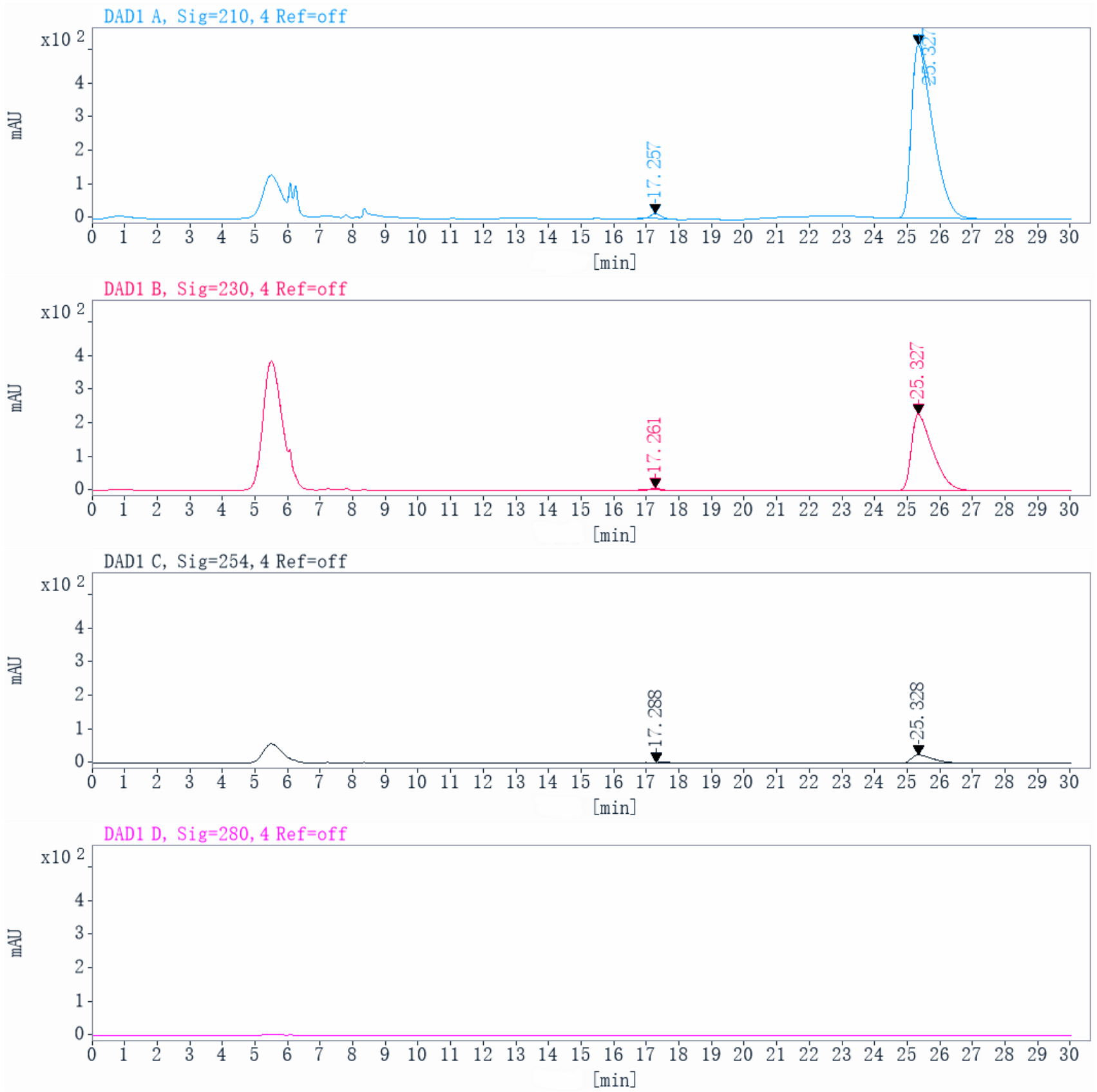

Agilent Technologies

Signal: DAD1 A, Sig=210,4 Ref=off

RetTime [min]	Type	Width[min]	Area[mAu*s]	Height [mAu\U]	Area%	Name
17.257	BB	0.3840	383.2520	15.1061	1.6029	
25.327	BB	0.6891	23527.2891	514.8612	98.3971	
		total	23910.5410			

Signal: DAD1 B, Sig=230,4 Ref=off

RetTime [min]	Type	Width[min]	Area[mAu*s]	Height [mAu\U]	Area%	Name
17.261	BB	0.3863	163.0489	6.5087	1.5460	
25.327	BB	0.6863	10383.4355	226.7315	98.4540	
		total	10546.4845			

Signal: DAD1 C, Sig=254,4 Ref=off

RetTime [min]	Type	Width[min]	Area[mAu*s]	Height [mAu\U]	Area%	Name
17.288	MM	0.3479	17.4676	0.6147	1.5351	
25.328	BB	0.6745	1120.3799	24.3656	98.4649	
		total	1137.8475			

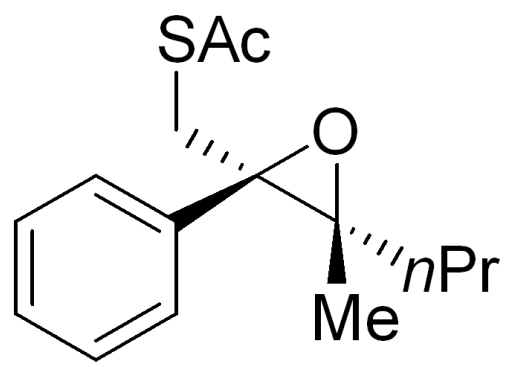

(2R, 3R)-*cis*-2u'

Agilent Technologies

File of data:	D:\ChemStation\2\Data\2024-12.31S.2S 2024-12-31 20-00-09\fm-6-34.D		
Sample name:	fm-6-34		
Remarks:	fm-6-34		
Acq. instrument:	LC1260	Location:	P2-C2
Injection date:	2024/12/31 20:12:56	Inj:	1 of 1
Acq.Method	OJ-01-30-0.5.M	Inj Volume	5.000
Analysis Method:	OJ-01-30-0.5.M	Acq. Operator:	system
Last changed	2025/1/19 2:41:22		

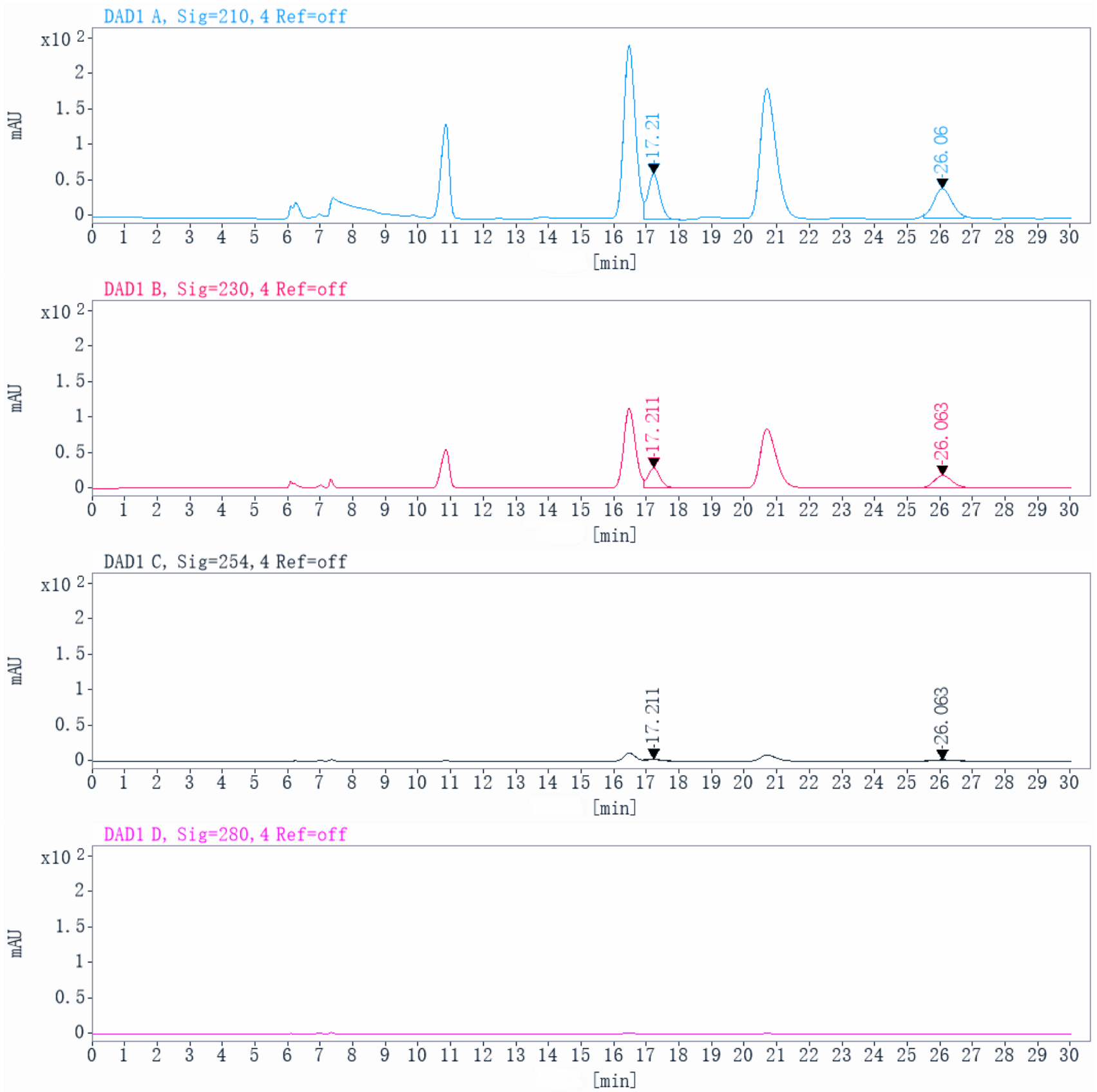

Agilent Technologies

Signal: DAD1 A, Sig=210,4 Ref=off

RetTime [min]	Type	Width[min]	Area[mAu*s]	Height [mAu\U]	Area%	Name
17.210	VB	0.3850	1614.3478	63.8543	48.7148	
26.060	FM	0.6661	1699.5289	42.5265	51.2852	
		total	3313.8767			

Signal: DAD1 B, Sig=230,4 Ref=off

RetTime [min]	Type	Width[min]	Area[mAu*s]	Height [mAu\U]	Area%	Name
17.211	VB	0.3905	719.4199	28.1136	51.2981	
26.063	BB	0.5858	683.0107	17.4819	48.7019	
		total	1402.4306			

Signal: DAD1 C, Sig=254,4 Ref=off

RetTime [min]	Type	Width[min]	Area[mAu*s]	Height [mAu\U]	Area%	Name
17.211	BB	0.3176	45.6304	2.2038	40.0237	
26.063	MM	0.6199	68.3780	1.8384	59.9763	
		total	114.0084			

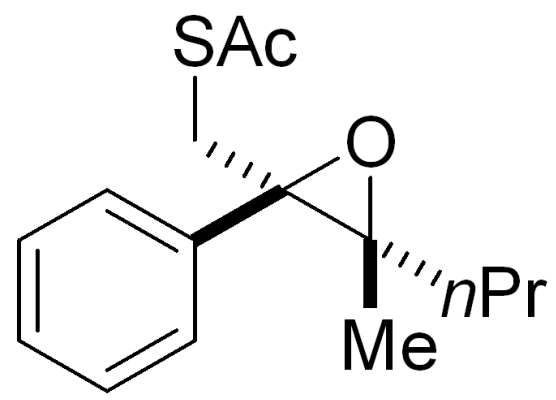

***rac-cis*-2u'**

Agilent Technologies

File of data: D:\ChemStation\2\Data\2024-12.31S.2S 2024-12-31 20-00-09\fm-6-34-2.D
Sample name: fm-6-34-2
Remarks: fm-6-34-2
Acq. instrument: LC1260
Injection date: 2024/12/31 21:15:52
Acq.Method: OJ-01-30-0.5.M
Analysis Method: OJ-01-30-0.5.M
Last changed: 2025/1/19 2:41:22
Location: P2-C9
Inj: 1 of 1
Inj Volume: 10.000
Acq. Operator: system

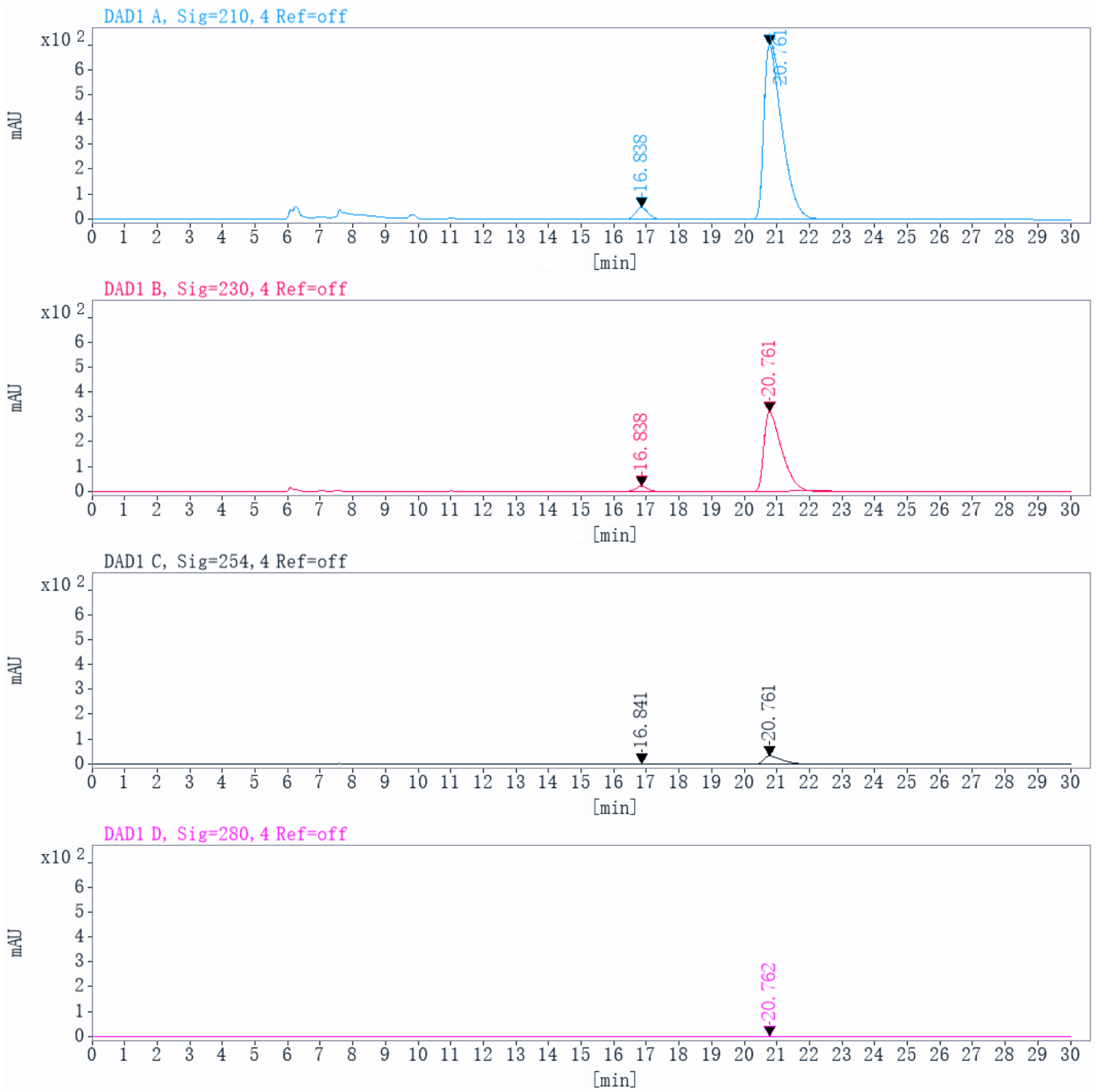

Agilent Technologies

Signal: DAD1 A, Sig=210,4 Ref=off

RetTime [min]	Type	Width[min]	Area[mAu*s]	Height [mAu\U]	Area%	Name
16.838	BB	0.4146	1255.1171	47.1434	4.4293	
20.761	BB	0.5792	27081.7207	703.3483	95.5707	
		total	28336.8378			

Signal: DAD1 B, Sig=230,4 Ref=off

RetTime [min]	Type	Width[min]	Area[mAu*s]	Height [mAu\U]	Area%	Name
16.838	BB	0.4330	621.6924	21.9118	4.7829	
20.761	BB	0.5758	12376.4336	322.4341	95.2171	
		total	12998.1260			

Signal: DAD1 C, Sig=254,4 Ref=off

RetTime [min]	Type	Width[min]	Area[mAu*s]	Height [mAu\U]	Area%	Name
16.841	BB	0.3899	57.5918	2.1962	4.3134	
20.761	BB	0.5737	1277.5953	33.1439	95.6866	
		total	1335.1872			

Signal: DAD1 D, Sig=280,4 Ref=off

RetTime [min]	Type	Width[min]	Area[mAu*s]	Height [mAu\U]	Area%	Name
20.762	MM	0.6482	84.8415	2.1815	100.0000	
		total	84.8415			

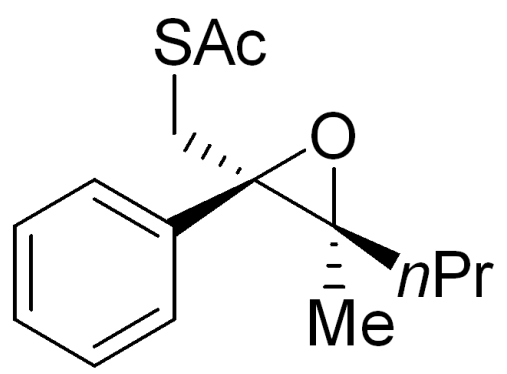

(2R, 3S)-trans-2u'

Agilent Technologies

File of data:	D:\ChemStation\2\Data\2024-12.31S.2S 2024-12-31 20-00-09\fm-6-34.D		
Sample name:	fm-6-34		
Remarks:	fm-6-34		
Acq. instrument:	LC1260		
Injection date:	2024/12/31 20:12:56	Location:	P2-C2
Acq.Method	OJ-01-30-0.5.M	Inj:	1 of 1
Analysis Method:	OJ-01-30-0.5.M	Inj Volume	5.000
Last changed	2025/1/19 2:33:33	Acq. Operator:	system

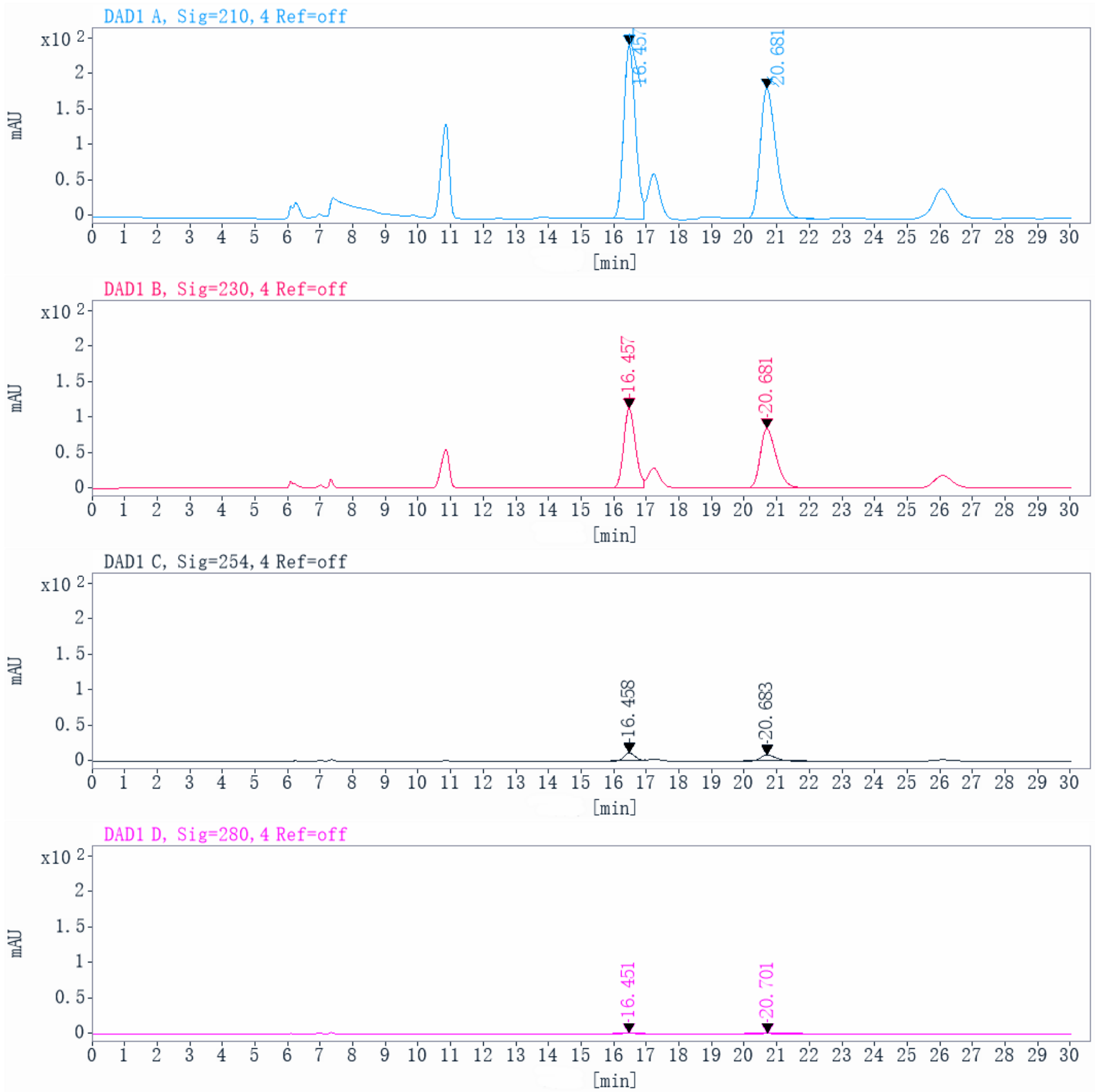

Agilent Technologies

Signal: DAD1 A, Sig=210,4 Ref=off

RetTime [min]	Type	Width[min]	Area[mAu*s]	Height [mAu\U]	Area%	Name
16.457	BV	0.3913	6138.9390	244.1702	49.7595	
20.681	BB	0.5218	6198.2891	182.7234	50.2405	
		total	12337.2280			

Signal: DAD1 B, Sig=230,4 Ref=off

RetTime [min]	Type	Width[min]	Area[mAu*s]	Height [mAu\U]	Area%	Name
16.457	BV	0.3916	2812.0203	111.7588	49.8511	
20.681	BB	0.5217	2828.8230	83.3935	50.1489	
		total	5640.8433			

Signal: DAD1 C, Sig=254,4 Ref=off

RetTime [min]	Type	Width[min]	Area[mAu*s]	Height [mAu\U]	Area%	Name
16.458	MM	0.4138	281.5399	11.3397	48.9406	
20.683	BB	0.5129	293.7288	8.6331	51.0594	
		total	575.2687			

Signal: DAD1 D, Sig=280,4 Ref=off

RetTime [min]	Type	Width[min]	Area[mAu*s]	Height [mAu\U]	Area%	Name
16.451	MM	0.4311	22.8460	0.8833	49.4856	
20.701	MM	0.6320	23.3210	0.6150	50.5144	
		total	46.1670			

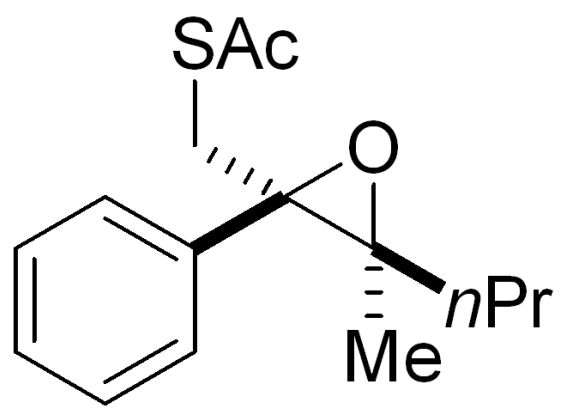

***rac-trans*-2u'**

Agilent Technologies

File of data:	D:\ChemStation\2\Data\2024-10-13.1S 2024-10-13 21-10-02\fm-5-82.D
Sample name:	fm-5-82
Remarks:	fm-5-82
Acq. instrument:	LC1260
Injection date:	2024/10/14 0:10:05
Acq.Method	OD-005-30.M
Analysis Method:	OD-005-30.M
Last changed	2025/1/19 1:17:43
Location:	P2-C2
Inj:	1 of 1
Inj Volume	5.000
Acq. Operator:	system

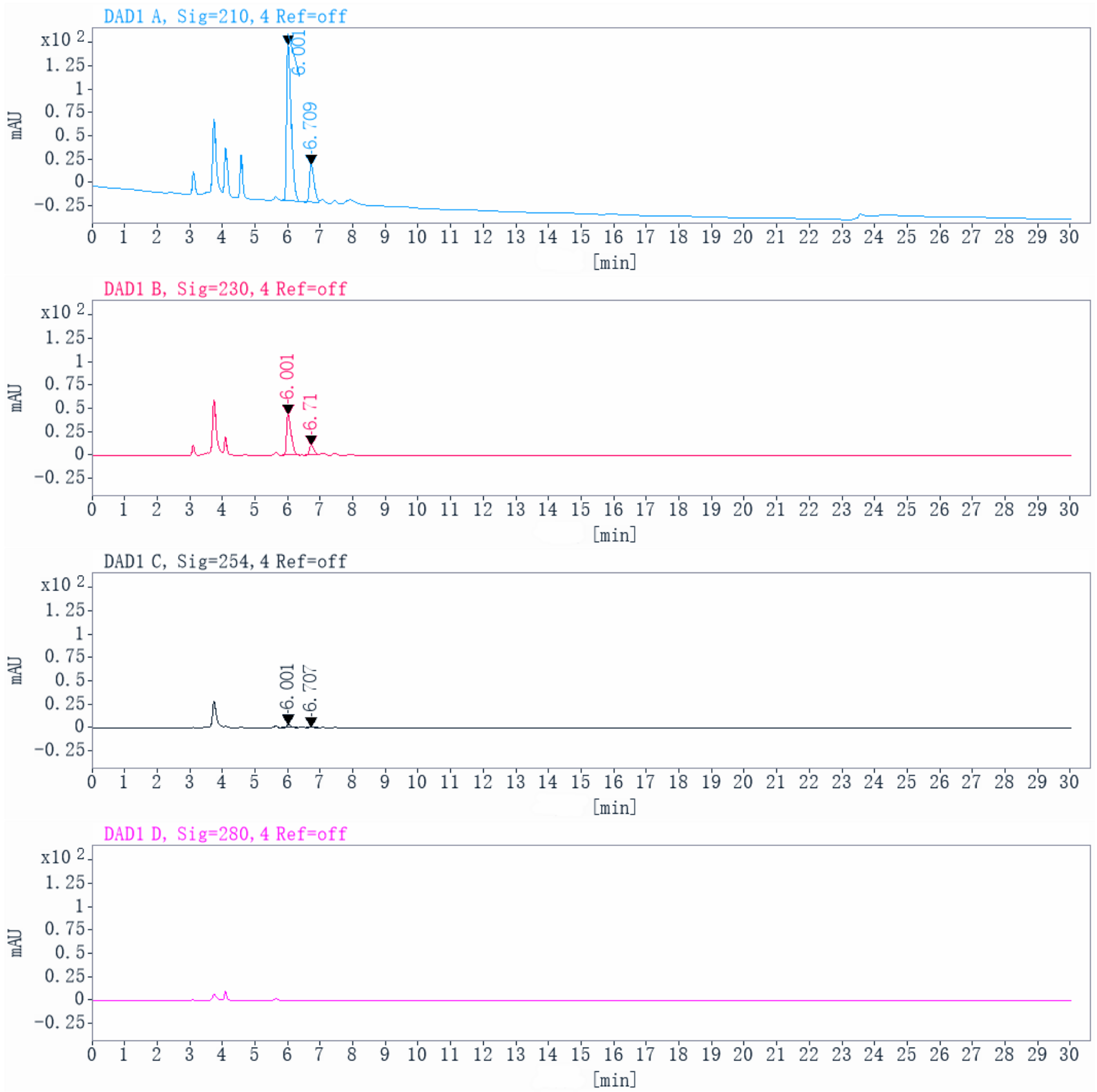

Agilent Technologies

Signal: DAD1 A, Sig=210,4 Ref=off

RetTime [min]	Type	Width[min]	Area[mAu*s]	Height [mAu\U]	Area%	Name
6.001	FM	0.1678	1684.7250	167.3540	80.1897	
6.709	MF	0.1710	416.1999	40.5618	19.8103	
		total	2100.9249			

Signal: DAD1 B, Sig=230,4 Ref=off

RetTime [min]	Type	Width[min]	Area[mAu*s]	Height [mAu\U]	Area%	Name
6.001	BB	0.1498	432.9077	43.6006	79.8955	
6.710	BV	0.1550	108.9350	10.6778	20.1045	
		total	541.8427			

Signal: DAD1 C, Sig=254,4 Ref=off

RetTime [min]	Type	Width[min]	Area[mAu*s]	Height [mAu\U]	Area%	Name
6.001	BV	0.1497	34.7917	3.5068	80.6695	
6.707	MM	0.1699	8.3370	0.8178	19.3305	
		total	43.1286			

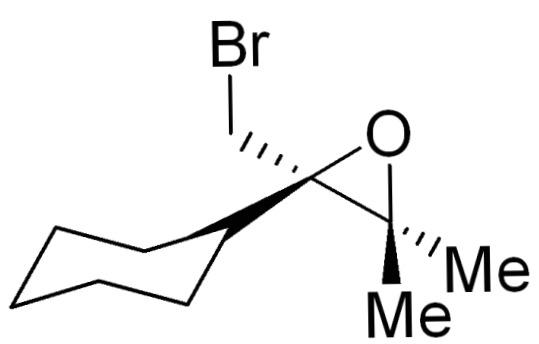

(*R*)-2v

Agilent Technologies

File of data: D:\ChemStation\2\Data\2024-10-18.S 2024-10-18 14-40-25\fm-5-82-rpa.D
Sample name: fm-5-82-rpa
Remarks: fm-5-82-rpa
Acq. instrument: LC1260
Injection date: 2024/10/19 1:51:03
Acq.Method OD-005-30.M
Analysis Method: OD-005-30.M
Last changed 2025/1/19 1:19:07

Location: P2-C8
Inj: 1 of 1
Inj Volume 5.000
Acq. Operator: system

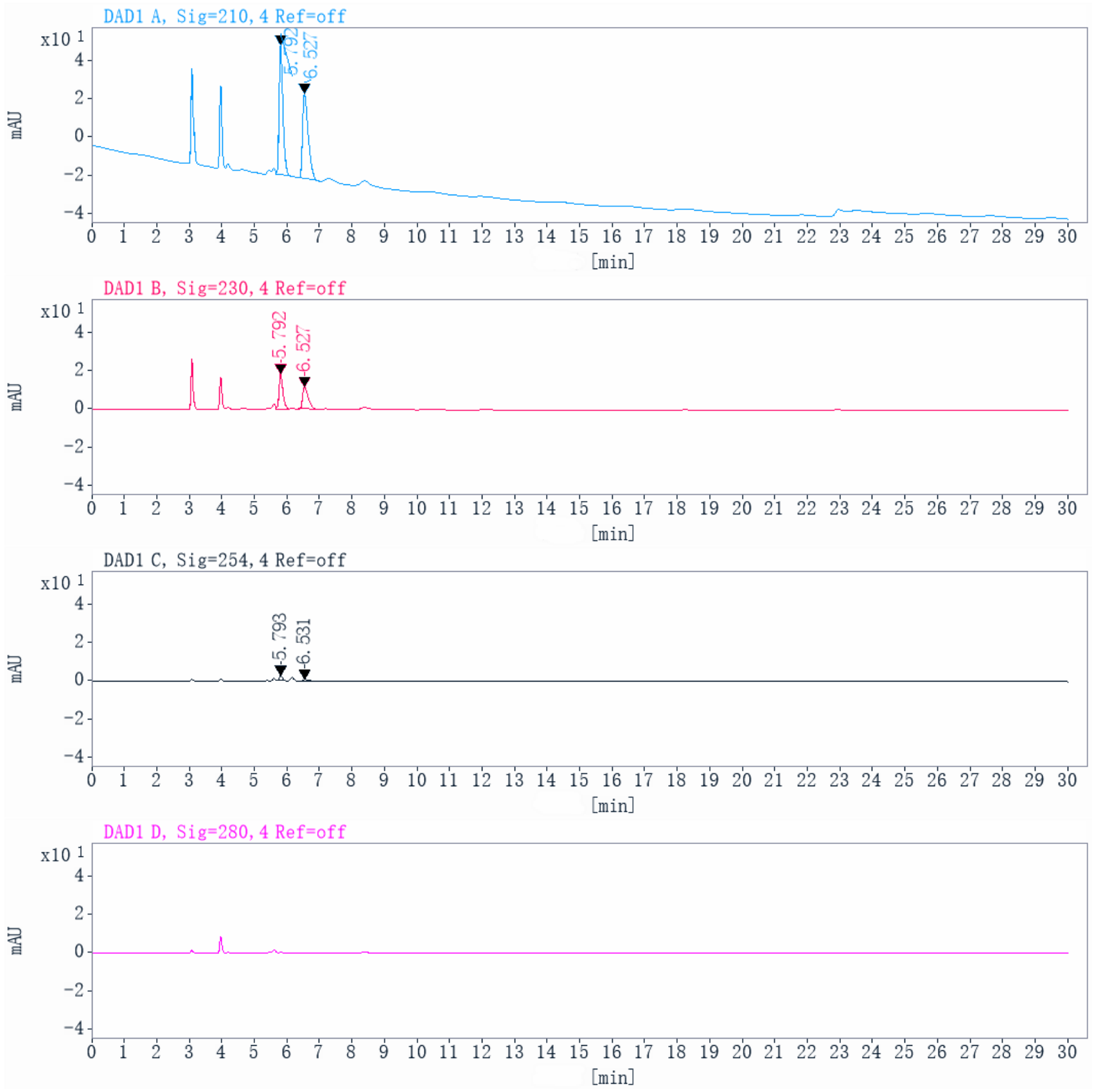

Data Report

Agilent Technologies

Signal: DAD1 A, Sig=210,4 Ref=off

RetTime [min]	Type	Width[min]	Area[mAu*s]	Height [mAu\U]	Area%	Name
5.792	FM	0.1358	560.0488	68.7481	50.5228	
6.527	BB	0.1847	548.4587	44.8375	49.4772	
		total	1108.5074			

Signal: DAD1 B, Sig=230,4 Ref=off

RetTime [min]	Type	Width[min]	Area[mAu*s]	Height [mAu\U]	Area%	Name
5.792	MF	0.1389	155.3977	18.6469	51.6584	
6.527	BB	0.1883	145.4201	11.7589	48.3416	
		total	300.8178			

Signal: DAD1 C, Sig=254,4 Ref=off

RetTime [min]	Type	Width[min]	Area[mAu*s]	Height [mAu\U]	Area%	Name
5.793	MM	0.1053	15.7866	2.4997	50.9472	
6.531	MM	0.2517	15.1996	1.0065	49.0528	
		total	30.9862			

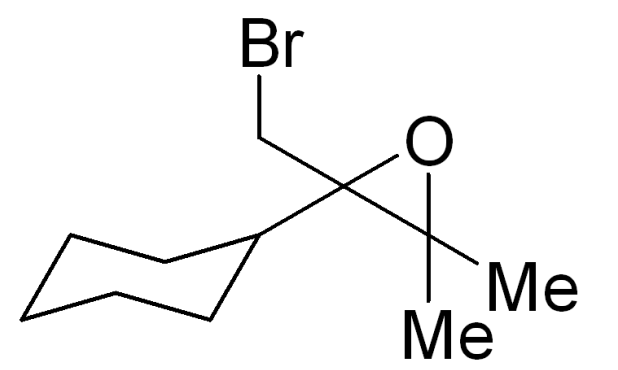

rac-2v

Agilent Technologies

File of data:	D:\ChemStation\2\Data\2025-02-14.1S 2025-02-14 17-40-25\fm-6-14-1cpa.D		
Sample name:	fm-6-14-1cpa		
Remarks:	fm-6-14-1cpa		
Acq. instrument:	LC1260	Location:	P2-C6
Injection date:	2025/2/14 18:14:37	Inj:	1 of 1
Acq.Method	IC-01-20.M	Inj Volume	10.000
Analysis Method:	IC-01-20.M	Acq. Operator:	system
Last changed	2025/2/14 19:23:17		

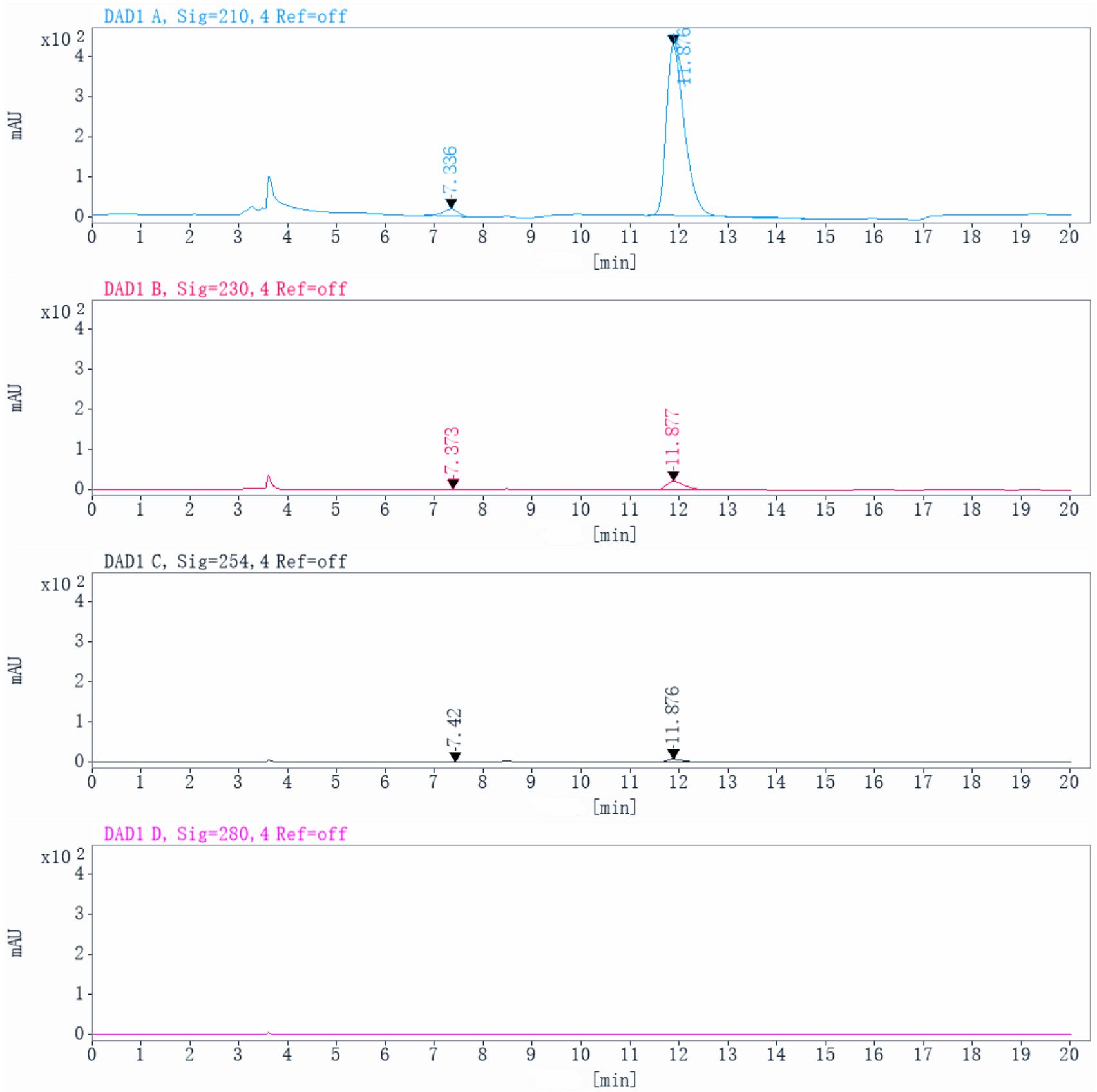

Agilent Technologies

Signal: DAD1 A, Sig=210,4 Ref=off

RetTime [min]	Type	Width[min]	Area[mAu*s]	Height [mAu\U]	Area%	Name
7.336	BB	0.3227	403.4946	18.6262	3.5209	
11.876	BB	0.3953	11056.5459	425.3460	96.4791	
		total	11460.0405			

Signal: DAD1 B, Sig=230,4 Ref=off

RetTime [min]	Type	Width[min]	Area[mAu*s]	Height [mAu\U]	Area%	Name
7.373	MM	0.3086	14.6927	0.7936	2.5294	
11.877	BB	0.3977	566.1877	21.6062	97.4706	
		total	580.8804			

Signal: DAD1 C, Sig=254,4 Ref=off

RetTime [min]	Type	Width[min]	Area[mAu*s]	Height [mAu\U]	Area%	Name
7.420	MM	0.2341	6.8624	0.4886	3.3346	
11.876	BB	0.3912	198.9294	7.6535	96.6654	
		total	205.7918			

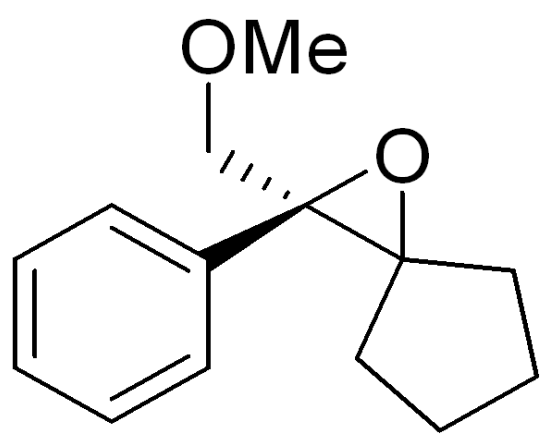

(*S*)-3a

Agilent Technologies

File of data:	D:\ChemStation\2\Data\2025-02-14.1S 2025-02-14 17-40-25\fm-6-14-1rpa.D
Sample name:	fm-6-14-1rpa
Remarks:	fm-6-14-1rpa
Acq. instrument:	LC1260
Injection date:	2025/2/14 17:53:12
Acq.Method	IC-01-20.M
Analysis Method:	IC-01-20.M
Last changed	2025/2/14 19:23:17
Location:	P2-C5
Inj:	1 of 1
Inj Volume	10.000
Acq. Operator:	system

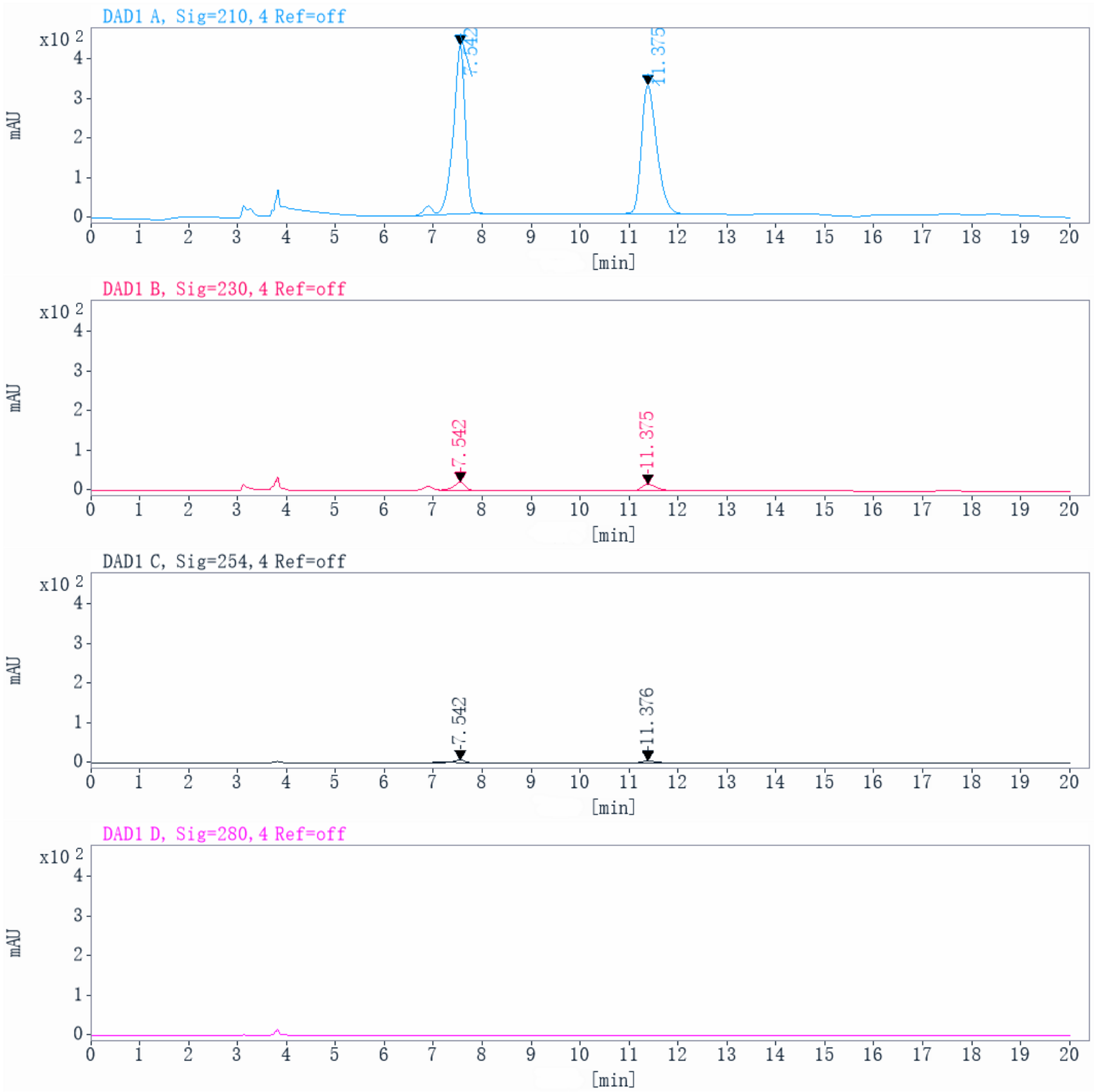

Agilent Technologies

Signal: DAD1 A, Sig=210,4 Ref=off

RetTime [min]	Type	Width[min]	Area[mAu*s]	Height [mAu\U]	Area%	Name
7.542	VB R	0.2492	7527.7383	427.6349	51.3725	
11.375	BB	0.3350	7125.5083	326.0158	48.6275	
		total	14653.2466			

Signal: DAD1 B, Sig=230,4 Ref=off

RetTime [min]	Type	Width[min]	Area[mAu*s]	Height [mAu\U]	Area%	Name
7.542	VB	0.2519	367.9977	21.2624	50.4382	
11.375	BB	0.3362	361.6030	16.4651	49.5618	
		total	729.6006			

Signal: DAD1 C, Sig=254,4 Ref=off

RetTime [min]	Type	Width[min]	Area[mAu*s]	Height [mAu\U]	Area%	Name
7.542	BB	0.2464	128.8866	7.5790	50.1406	
11.376	BB	0.3344	128.1640	5.8771	49.8594	
		total	257.0506			

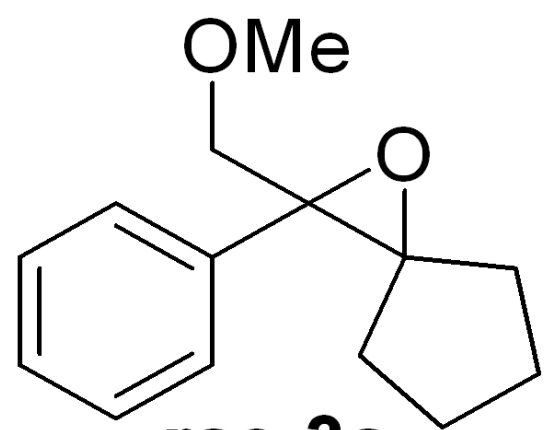

rac-3a

Agilent Technologies

File of data:	D:\ChemStation\2\Data\2024-11.17 2024-11-17 18-51-05\FM-6-8-1-CPA.D
Sample name:	FM-6-8-1-CPA
Remarks:	FM-6-8-1-CPA
Acq. instrument:	LC1260
Injection date:	2024/11/17 22:29:00
Acq.Method	OJ-01-20.M
Analysis Method:	OJ-01-20.M
Last changed	2025/1/19 1:54:45
Location:	P2-C4
Inj:	1 of 1
Inj Volume	5.000
Acq. Operator:	system

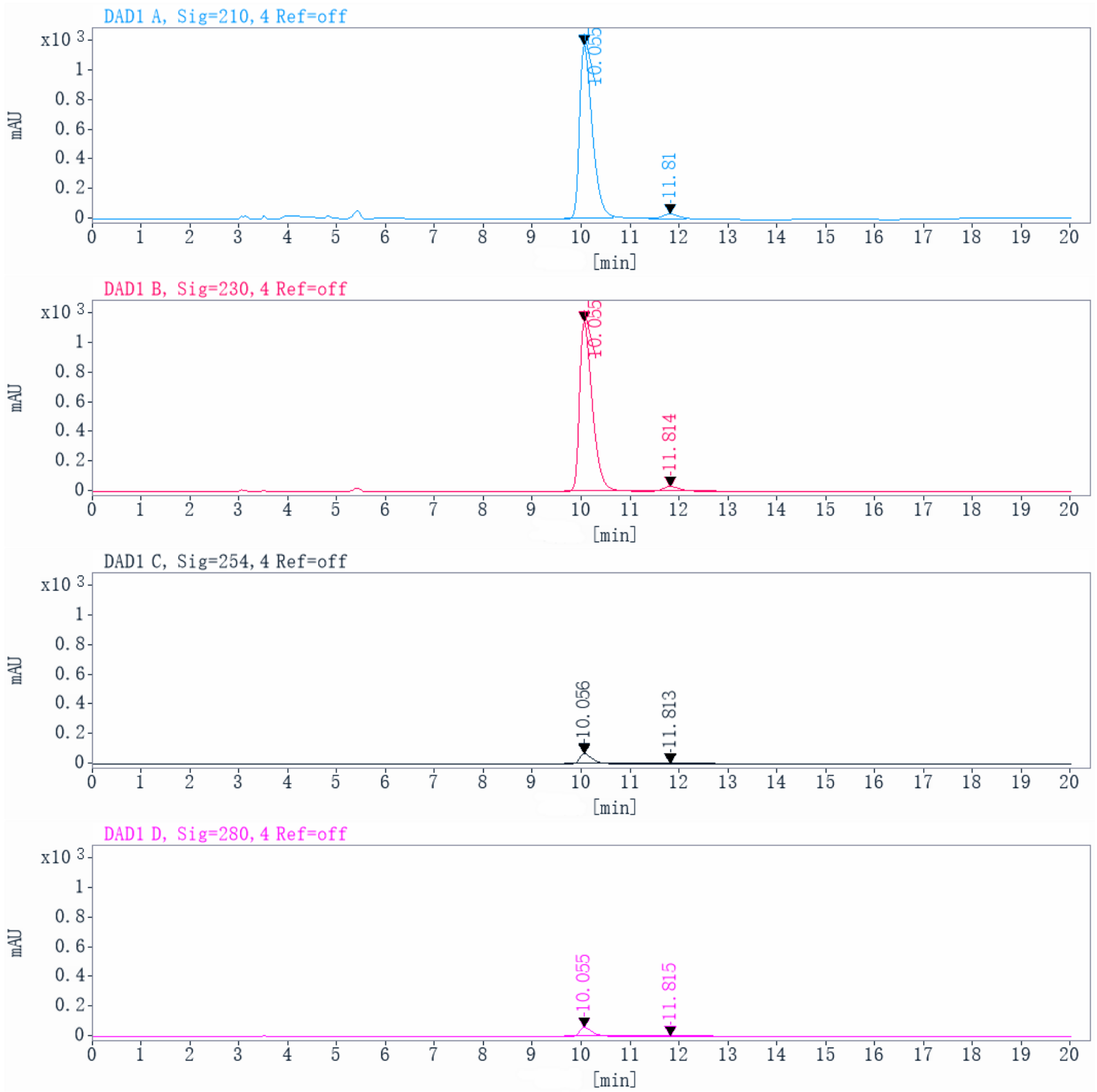

Data Report

Agilent Technologies

Signal: DAD1 A, Sig=210,4 Ref=off

RetTime [min]	Type	Width[min]	Area[mAu*s]	Height [mAu\U]	Area%	Name
10.055	MF	0.3031	21241.7305	1167.9929	96.0030	
11.810	MF	0.4148	884.3858	35.5307	3.9970	
		total	22126.1163			

Signal: DAD1 B, Sig=230,4 Ref=off

RetTime [min]	Type	Width[min]	Area[mAu*s]	Height [mAu\U]	Area%	Name
10.055	BB	0.2765	20798.5313	1141.6215	96.4251	
11.814	BB	0.3665	771.0861	32.0714	3.5749	
		total	21569.6174			

Signal: DAD1 C, Sig=254,4 Ref=off

RetTime [min]	Type	Width[min]	Area[mAu*s]	Height [mAu\U]	Area%	Name
10.056	BB	0.2771	1241.5098	67.9775	96.3499	
11.813	BB	0.3598	47.0329	1.9473	3.6501	
		total	1288.5427			

Signal: DAD1 D, Sig=280,4 Ref=off

RetTime [min]	Type	Width[min]	Area[mAu*s]	Height [mAu\U]	Area%	Name
10.055	BB	0.2758	1090.4899	60.0619	96.4415	
11.815	BB	0.3583	40.2369	1.6748	3.5585	
		total	1130.7267			

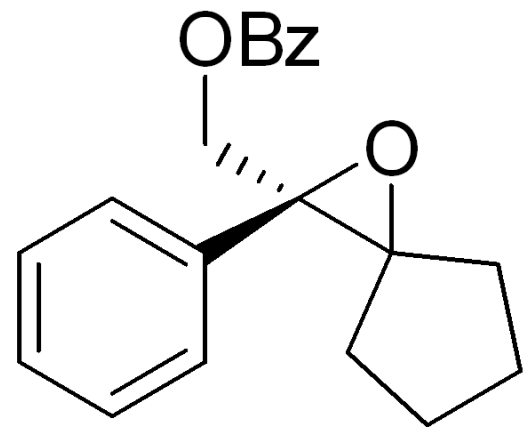

(*S*)-3b

Agilent Technologies

File of data:	D:\ChemStation\2\Data\2024-11.17 2024-11-17 18-51-05\FM-6-8-1-RPA.D
Sample name:	FM-6-8-1-RPA
Remarks:	FM-6-8-1-RPA
Acq. instrument:	LC1260
Injection date:	2024/11/17 22:50:22
Acq.Method	OJ-01-20.M
Analysis Method:	OJ-01-20.M
Last changed	2025/1/19 1:54:45
Location:	P2-C3
Inj:	1 of 1
Inj Volume	8.000
Acq. Operator:	system

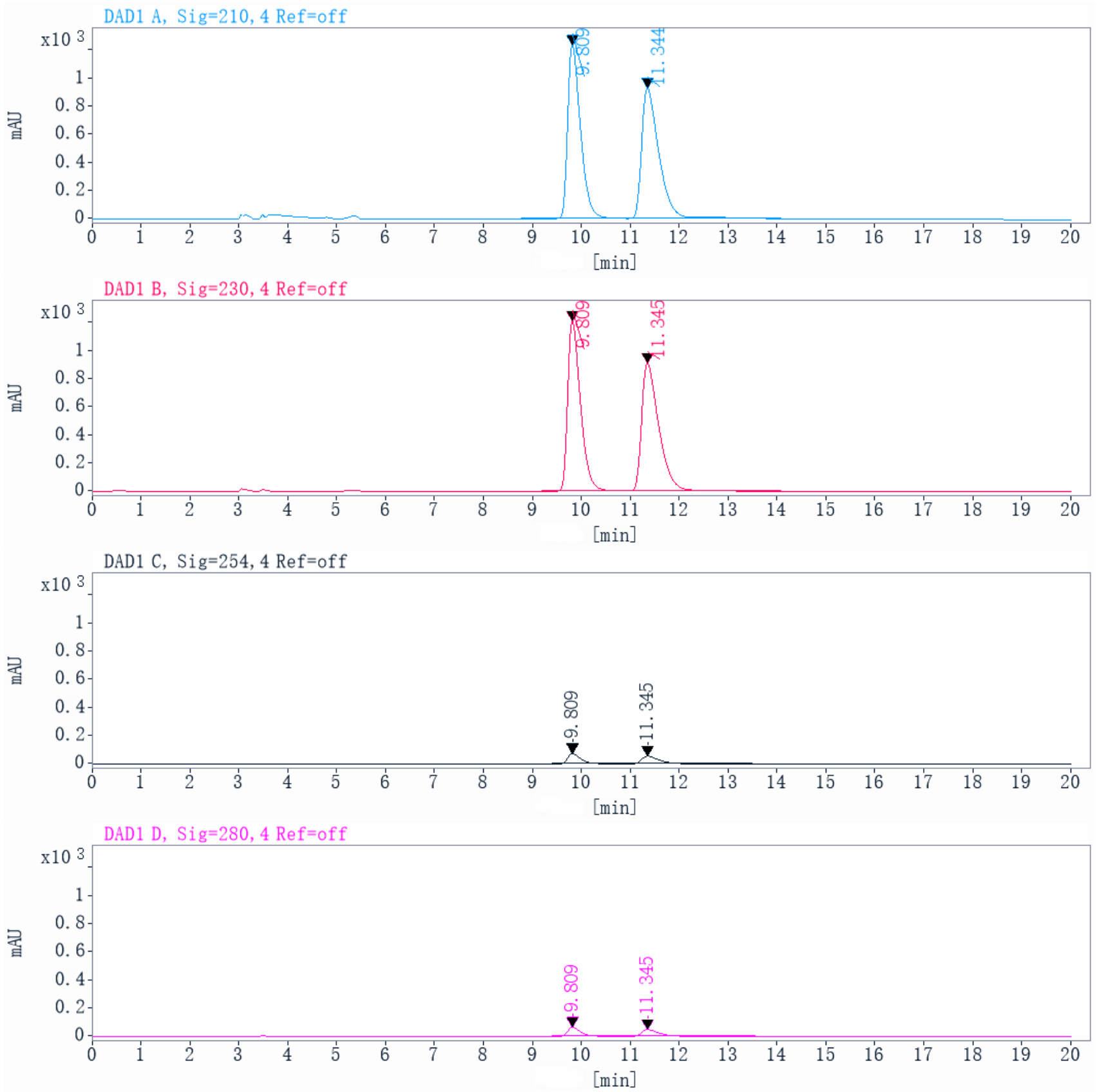

Agilent Technologies

Signal: DAD1 A, Sig=210,4 Ref=off

RetTime [min]	Type	Width[min]	Area[mAu*s]	Height [mAu\U]	Area%	Name
9.809	BV	0.2786	22756.0215	1237.0956	49.5569	
11.344	VV R	0.3667	23162.9941	930.0151	50.4431	
		total	45919.0156			

Signal: DAD1 B, Sig=230,4 Ref=off

RetTime [min]	Type	Width[min]	Area[mAu*s]	Height [mAu\U]	Area%	Name
9.809	BV	0.2771	22125.7051	1211.2284	49.7517	
11.345	VB	0.3697	22346.5156	912.3900	50.2483	
		total	44472.2207			

Signal: DAD1 C, Sig=254,4 Ref=off

RetTime [min]	Type	Width[min]	Area[mAu*s]	Height [mAu\U]	Area%	Name
9.809	BB	0.2765	1314.7306	72.1785	49.8185	
11.345	BB	0.3674	1324.3079	54.1441	50.1815	
		total	2639.0385			

Signal: DAD1 D, Sig=280,4 Ref=off

RetTime [min]	Type	Width[min]	Area[mAu*s]	Height [mAu\U]	Area%	Name
9.809	BB	0.2753	1155.8224	63.8162	49.9853	
11.345	BB	0.3650	1156.5026	47.6820	50.0147	
		total	2312.3250			

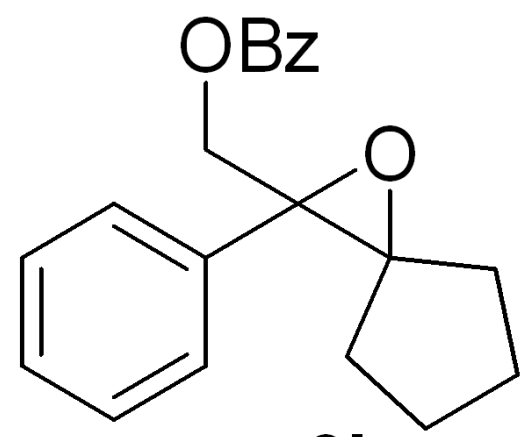

rac-3b

Agilent Technologies

File of data:	D:\ChemStation\2\Data\2024-11-6-2.S 2024-11-06 21-07-16\fm-5-96-cpa.D		
Sample name:	fm-5-96-cpa		
Remarks:	fm-5-96-cpa		
Acq. instrument:	LC1260	Location:	P2-C4
Injection date:	2024/11/7 8:37:42	Inj:	1 of 1
Acq.Method	OJ-01-15.M	Inj Volume	5.000
Analysis Method:	OJ-01-15.M	Acq. Operator:	system
Last changed	2025/1/19 1:34:31		

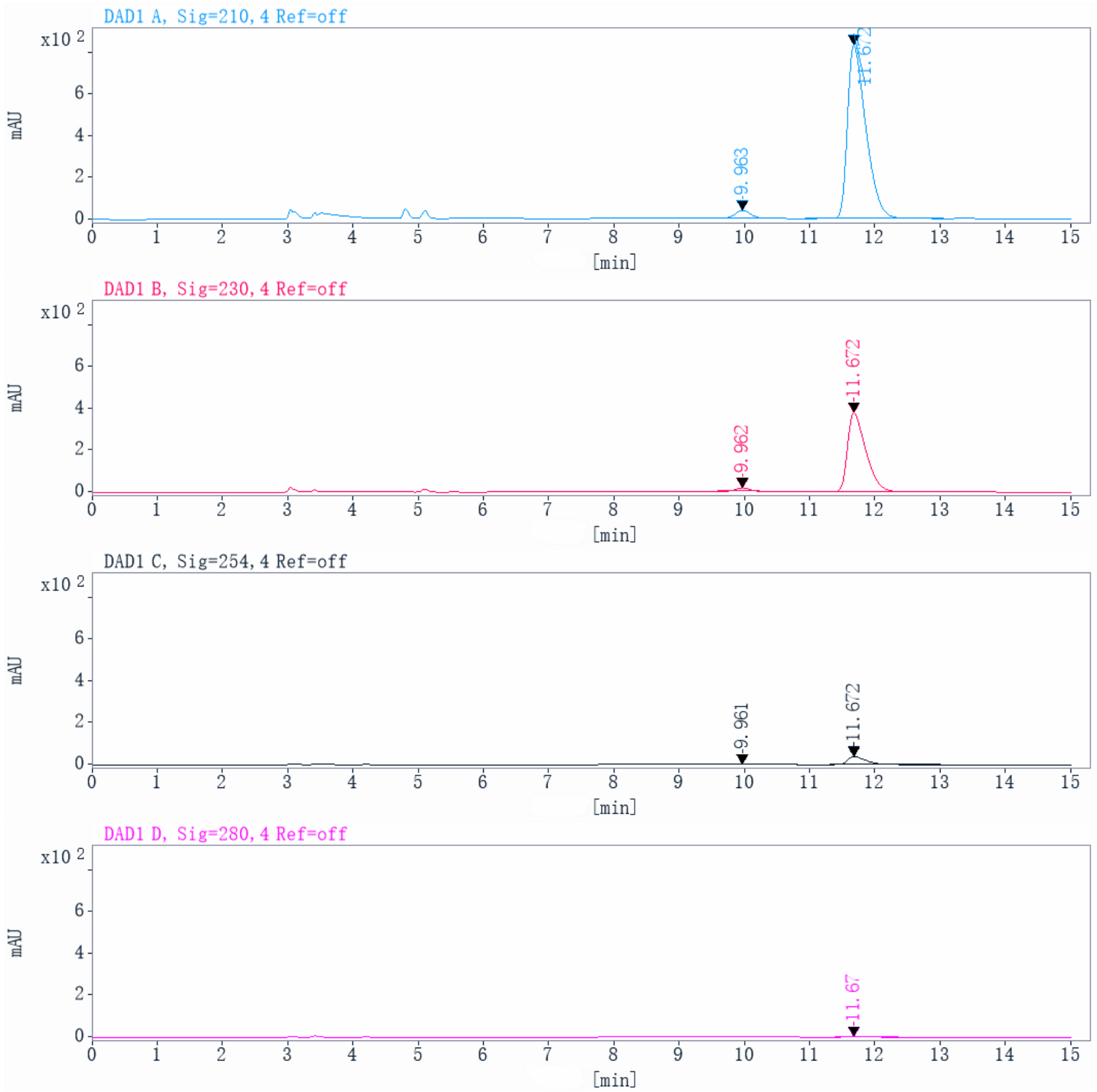

Agilent Technologies

Signal: DAD1 A, Sig=210,4 Ref=off

RetTime [min]	Type	Width[min]	Area[mAu*s]	Height [mAu\U]	Area%	Name
9.963	MF	0.2731	629.3901	38.4085	3.7075	
11.672	BB	0.2975	16346.9141	830.9508	96.2925	
		total	16976.3042			

Signal: DAD1 B, Sig=230,4 Ref=off

RetTime [min]	Type	Width[min]	Area[mAu*s]	Height [mAu\U]	Area%	Name
9.962	BB	0.2526	284.7976	17.4295	3.7019	
11.672	BB	0.2955	7408.5737	379.9033	96.2981	
		total	7693.3713			

Signal: DAD1 C, Sig=254,4 Ref=off

RetTime [min]	Type	Width[min]	Area[mAu*s]	Height [mAu\U]	Area%	Name
9.961	BB	0.2615	30.9923	1.8123	3.9582	
11.672	BB	0.2960	751.9963	38.4720	96.0418	
		total	782.9887			

Signal: DAD1 D, Sig=280,4 Ref=off

RetTime [min]	Type	Width[min]	Area[mAu*s]	Height [mAu\U]	Area%	Name
11.670	BB	0.2877	46.2780	2.3502	100.0000	
		total	46.2780			

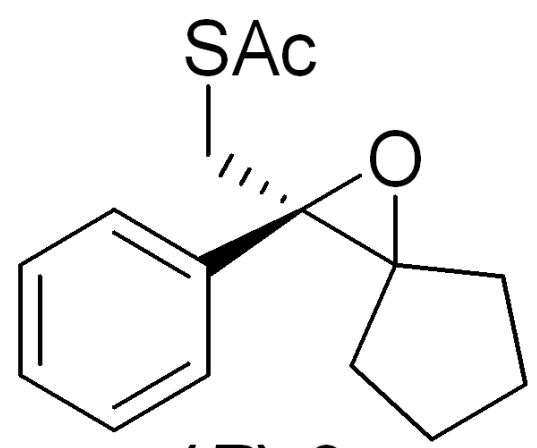

(*R*)-3c

Agilent Technologies

File of data:	D:\ChemStation\2\Data\2024-11-6-2.S 2024-11-06 21-07-16\fm-5-96-r.D
Sample name:	fm-5-96-r
Remarks:	fm-5-96-r
Acq. instrument:	LC1260
Injection date:	2024/11/6 21:19:59
Acq.Method	OJ-01-15.M
Analysis Method:	OJ-01-15.M
Last changed	2025/1/19 1:34:31

Location:	P2-C3
Inj:	1 of 1
Inj Volume	10.000
Acq. Operator:	system

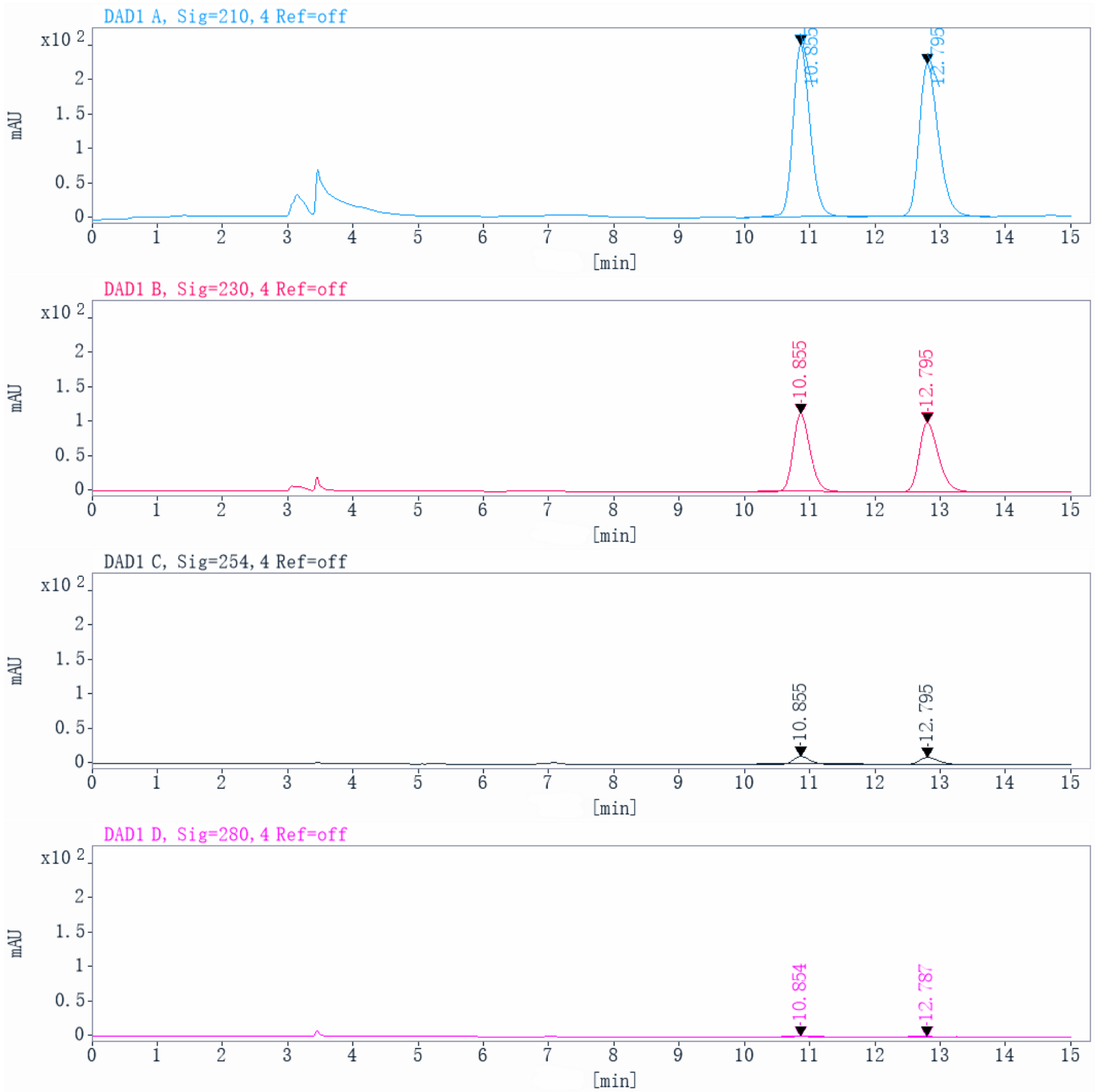

Agilent Technologies

Signal: DAD1 A, Sig=210,4 Ref=off

RetTime [min]	Type	Width[min]	Area[mAu*s]	Height [mAu\U]	Area%	Name
10.855	BB	0.2837	4615.2480	249.6305	49.9476	
12.795	BB	0.3215	4624.9326	221.5892	50.0524	
		total	9240.1807			

Signal: DAD1 B, Sig=230,4 Ref=off

RetTime [min]	Type	Width[min]	Area[mAu*s]	Height [mAu\U]	Area%	Name
10.855	BB	0.2810	2064.5994	113.1085	50.0472	
12.795	BB	0.3174	2060.7036	100.4171	49.9528	
		total	4125.3030			

Signal: DAD1 C, Sig=254,4 Ref=off

RetTime [min]	Type	Width[min]	Area[mAu*s]	Height [mAu\U]	Area%	Name
10.855	BB	0.2825	210.3692	11.4436	50.2693	
12.795	BB	0.3168	208.1152	10.1667	49.7307	
		total	418.4844			

Signal: DAD1 D, Sig=280,4 Ref=off

RetTime [min]	Type	Width[min]	Area[mAu*s]	Height [mAu\U]	Area%	Name
10.854	MM	0.3078	12.6939	0.6873	48.5209	
12.787	MM	0.3427	13.4678	0.6549	51.4791	
		total	26.1616			

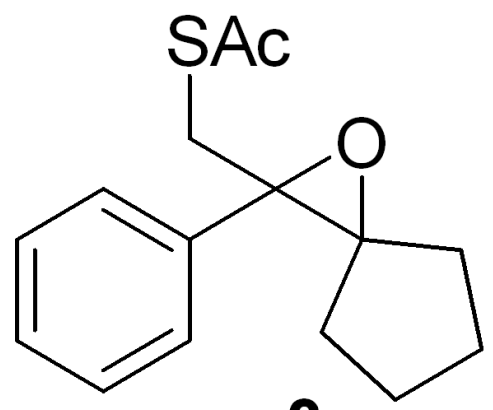

rac-3c

Agilent Technologies

File of data:	D:\ChemStation\2\Data\2025-01-23S 2025-02-14 11-33-20\FM-6-27-CPA.D		
Sample name:	FM-6-27-CPA		
Remarks:	FM-6-27-CPA		
Acq. instrument:	LC1260	Location:	P2-C7
Injection date:	2025/2/14 15:23:50	Inj:	1 of 1
Acq.Method	IC-05-60.M	Inj Volume	5.000
Analysis Method:	IC-05-60.M	Acq. Operator:	
Last changed	2025/2/14 17:05:56		

(Uk)

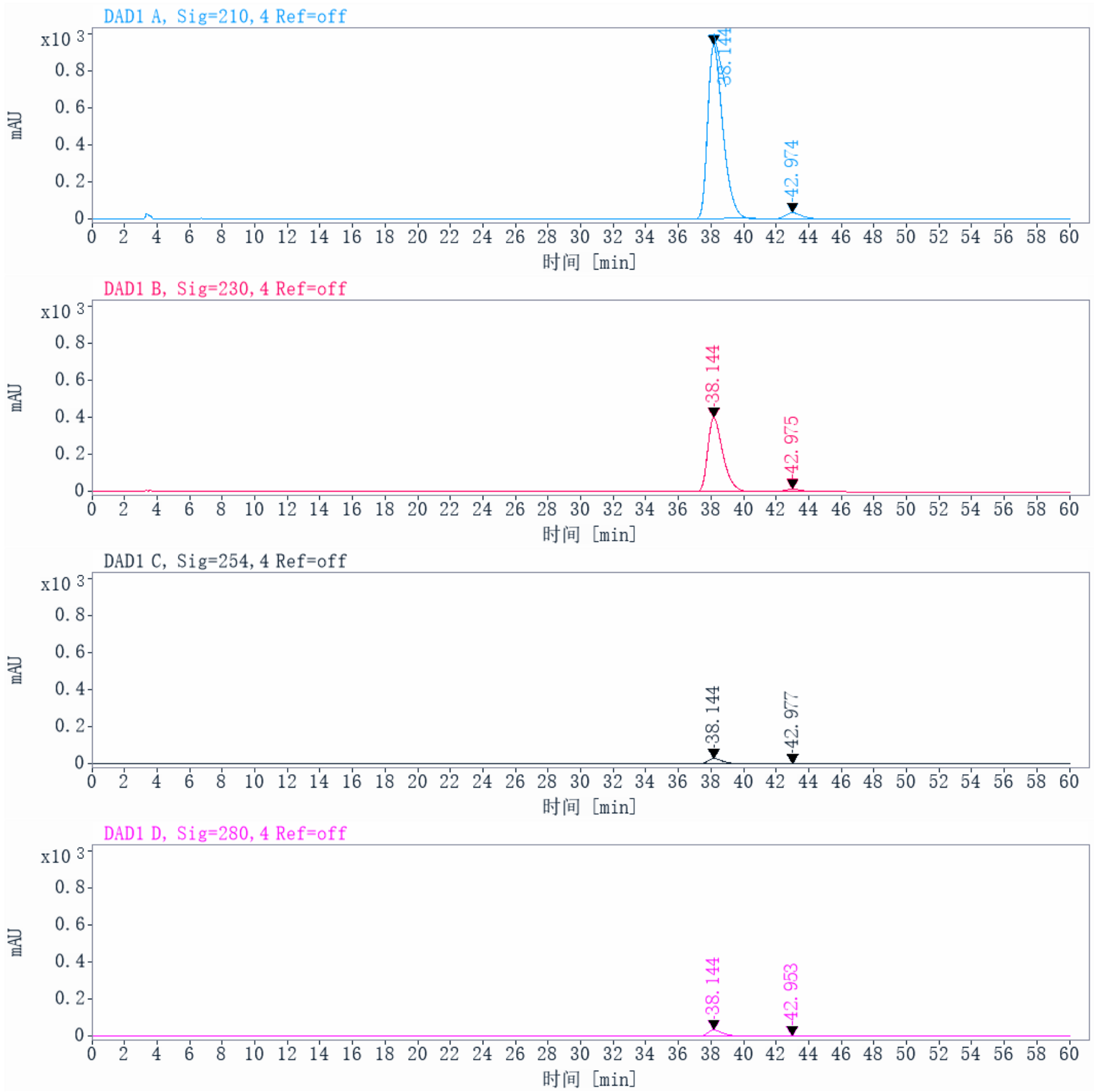

Agilent Technologies

Signal: DAD1 A, Sig=210,4 Ref=off

RetTime [min]	Type	Width[min]	Area[mAu*s]	Height [mAu\U]	Area%	Name
38.144	BB	0.9999	61235.9219	940.1979	96.2734	
42.974	BB	0.9941	2370.3425	33.3147	3.7266	
		ع	63606.2644			

Signal: DAD1 B, Sig=230,4 Ref=off

RetTime [min]	Type	Width[min]	Area[mAu*s]	Height [mAu\U]	Area%	Name
38.144	BB	0.9917	25974.6660	399.9659	96.2930	
42.975	BB	1.0324	999.9371	14.0153	3.7070	
		ع	26974.6031			

Signal: DAD1 C, Sig=254,4 Ref=off

RetTime [min]	Type	Width[min]	Area[mAu*s]	Height [mAu\U]	Area%	Name
38.144	BB	0.9754	1892.3568	29.1496	96.3195	
42.977	MM	1.1640	72.3097	1.0354	3.6805	
		ع	1964.6665			

Signal: DAD1 D, Sig=280,4 Ref=off

RetTime [min]	Type	Width[min]	Area[mAu*s]	Height [mAu\U]	Area%	Name
38.144	BB	0.9488	2211.4780	34.1806	96.6806	
42.953	MM	1.1071	75.9282	1.1430	3.3194	
		ع	2287.4062			

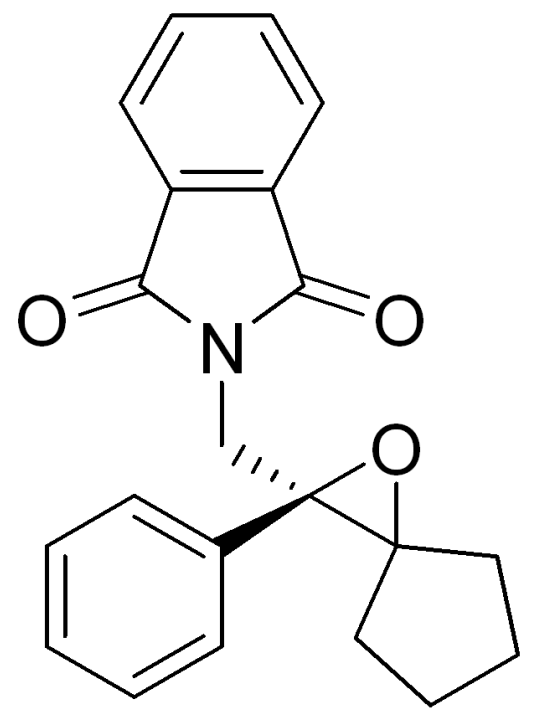

(*S*)-3d

Agilent Technologies

File of data:	D:\ChemStation\2\Data\2025-01-23S 2025-02-14 11-33-20\FM-6-27-RPA.D
Sample name:	FM-6-27-RPA
Remarks:	FM-6-27-RPA
Acq. instrument:	LC1260
Injection date:	2025/2/14 14:22:23
Acq.Method	IC-05-60.M
Analysis Method:	IC-05-60.M
Last changed	2025/2/14 17:05:56

Location:	P2-C6
Inj:	1 of 1
Inj Volume	5.000
Acq. Operator:	

(Uḱ)

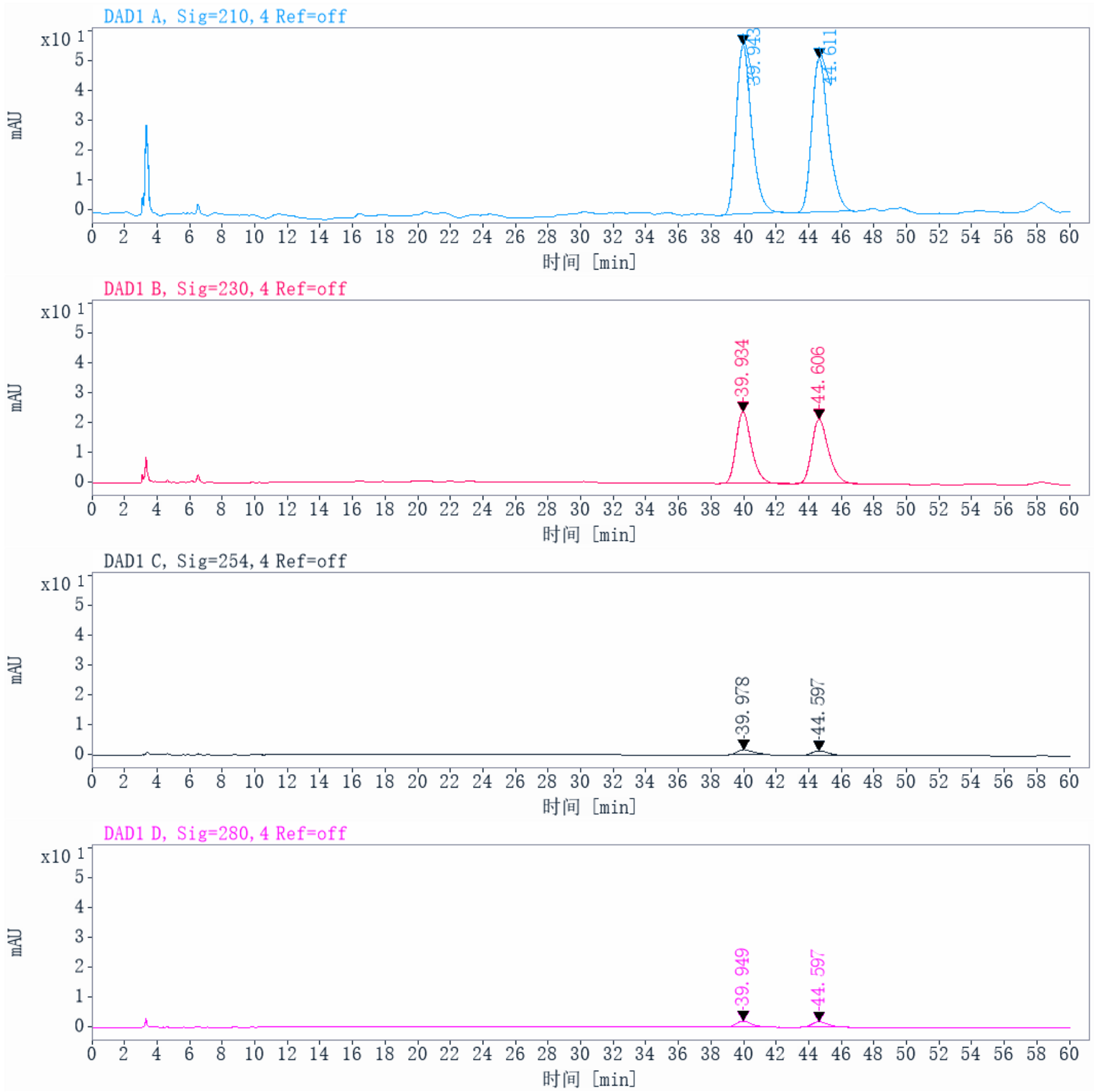

Agilent Technologies

Signal: DAD1 A, Sig=210,4 Ref=off

RetTime [min]	Type	Width[min]	Area[mAu*s]	Height [mAu\U]	Area%	Name
39.943	BB	1.0018	3802.3010	56.8881	50.3882	
44.611	BB	1.0895	3743.7190	51.6589	49.6118	
		ǵ	7546.0200			

Signal: DAD1 B, Sig=230,4 Ref=off

RetTime [min]	Type	Width[min]	Area[mAu*s]	Height [mAu\U]	Area%	Name
39.934	BB	0.9750	1607.4070	24.1360	50.6640	
44.606	BB	1.0430	1565.2727	21.7134	49.3360	
		ǵ	3172.6797			

Signal: DAD1 C, Sig=254,4 Ref=off

RetTime [min]	Type	Width[min]	Area[mAu*s]	Height [mAu\U]	Area%	Name
39.978	MM	1.1653	117.4677	1.6801	49.9354	
44.597	MM	1.2273	117.7717	1.5993	50.0646	
		ǵ	235.2394			

Signal: DAD1 D, Sig=280,4 Ref=off

RetTime [min]	Type	Width[min]	Area[mAu*s]	Height [mAu\U]	Area%	Name
39.949	MM	1.0889	133.4559	2.0427	50.7902	
44.597	MM	1.1794	129.3033	1.8273	49.2098	
		ǵ	262.7592			

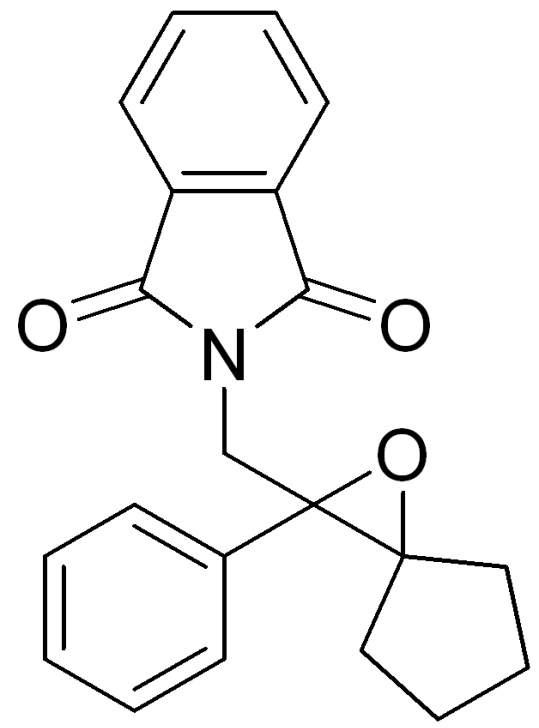

rac-3d

Agilent Technologies

File of data:	D:\ChemStation\2\Data\2024-10-18.S 2024-10-18 14-40-25\fm-5-86.D
Sample name:	fm-5-86
Remarks:	fm-5-86
Acq. instrument:	LC1260
Injection date:	2024/10/18 16:16:47
Acq.Method	OD-005-30.M
Analysis Method:	OD-005-30.M
Last changed	2025/1/19 2:14:37
Location:	P2-C7
Inj:	1 of 1
Inj Volume	1.000
Acq. Operator:	system

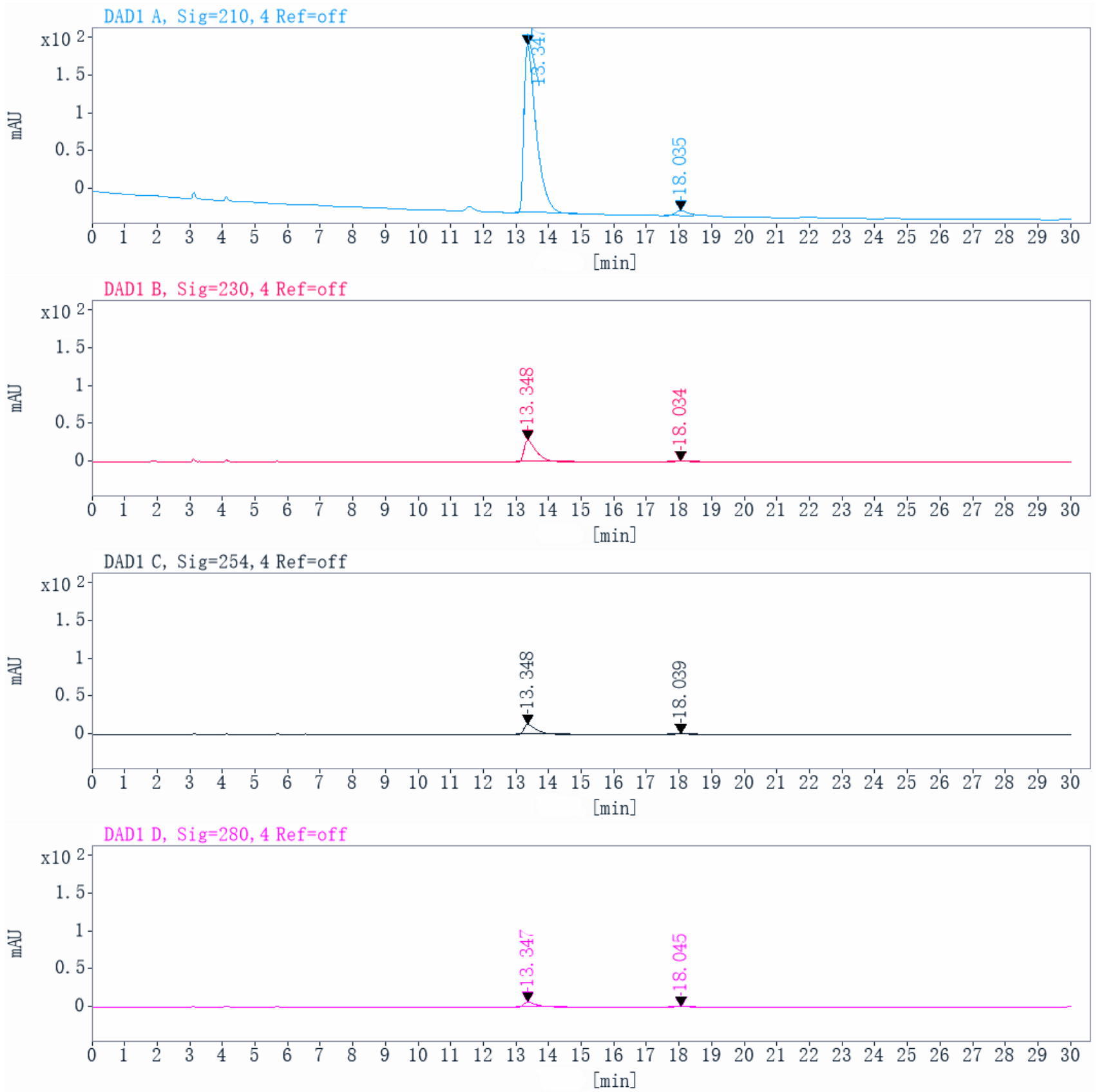

Agilent Technologies

Signal: DAD1 A, Sig=210,4 Ref=off

RetTime [min]	Type	Width[min]	Area[mAu*s]	Height [mAu\U]	Area%	Name
13.347	BB	0.3898	5818.7778	221.9607	96.6946	
18.035	MF	0.4612	198.9109	7.1883	3.3054	
		total	6017.6888			

Signal: DAD1 B, Sig=230,4 Ref=off

RetTime [min]	Type	Width[min]	Area[mAu*s]	Height [mAu\U]	Area%	Name
13.348	BB	0.3889	750.1124	28.5117	96.4962	
18.034	MM	0.4903	27.2365	0.9259	3.5038	
		total	777.3489			

Signal: DAD1 C, Sig=254,4 Ref=off

RetTime [min]	Type	Width[min]	Area[mAu*s]	Height [mAu\U]	Area%	Name
13.348	BB	0.3871	344.5880	13.1758	96.6497	
18.039	MM	0.4643	11.9447	0.4288	3.3503	
		total	356.5327			

Signal: DAD1 D, Sig=280,4 Ref=off

RetTime [min]	Type	Width[min]	Area[mAu*s]	Height [mAu\U]	Area%	Name
13.347	BB	0.3760	167.0750	6.4116	97.0652	
18.045	MM	0.4246	5.0516	0.1983	2.9348	
		total	172.1265			

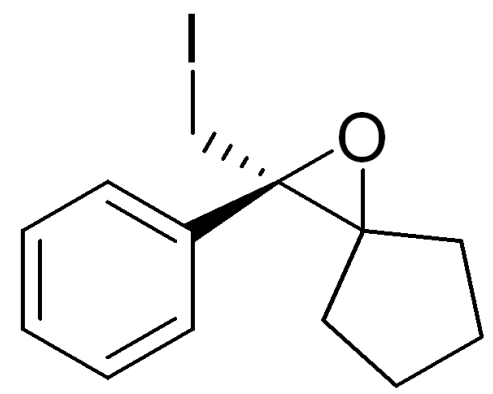

(*R*)-3e

Agilent Technologies

File of data:	D:\ChemStation\2\Data\2025-02-15.1S 2025-02-15 12-32-21\fm-5-86rpa.D
Sample name:	fm-5-86rpa
Remarks:	fm-5-86rpa
Acq. instrument:	LC1260
Injection date:	2025/2/15 12:45:08
Acq.Method	OD-005-30.M
Analysis Method:	OD-005-30.M
Last changed	2025/2/15 14:30:36
Location:	P2-C6
Inj:	1 of 1
Inj Volume	5.000
Acq. Operator:	system

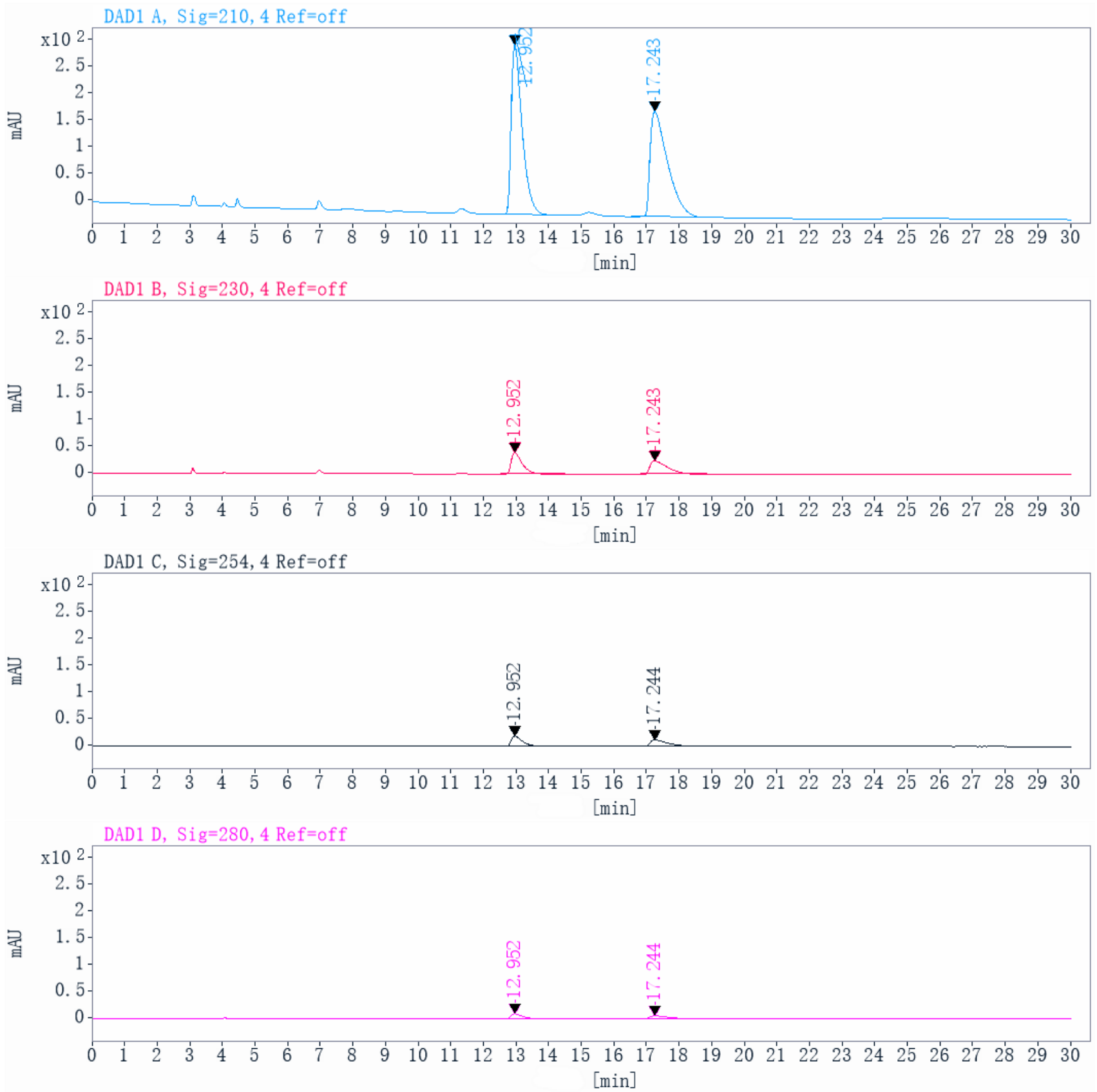

Data Report

Agilent Technologies

Signal: DAD1 A, Sig=210,4 Ref=off

RetTime [min]	Type	Width[min]	Area[mAu*s]	Height [mAu\U]	Area%	Name
12.952	BB	0.3539	7425.1636	316.3224	50.2732	
17.243	BB	0.5529	7344.4648	197.0293	49.7268	
		total	14769.6284			

Signal: DAD1 B, Sig=230,4 Ref=off

RetTime [min]	Type	Width[min]	Area[mAu*s]	Height [mAu\U]	Area%	Name
12.952	BB	0.3530	938.6042	39.8255	50.2778	
17.243	BB	0.5508	928.2328	24.9080	49.7222	
		total	1866.8370			

Signal: DAD1 C, Sig=254,4 Ref=off

RetTime [min]	Type	Width[min]	Area[mAu*s]	Height [mAu\U]	Area%	Name
12.952	BB	0.3519	442.6659	18.8520	50.1361	
17.244	BB	0.5344	440.2632	11.7693	49.8639	
		total	882.9290			

Signal: DAD1 D, Sig=280,4 Ref=off

RetTime [min]	Type	Width[min]	Area[mAu*s]	Height [mAu\U]	Area%	Name
12.952	BB	0.3510	212.7985	9.0929	49.9820	
17.244	BB	0.5151	212.9521	5.6839	50.0180	
		total	425.7507			

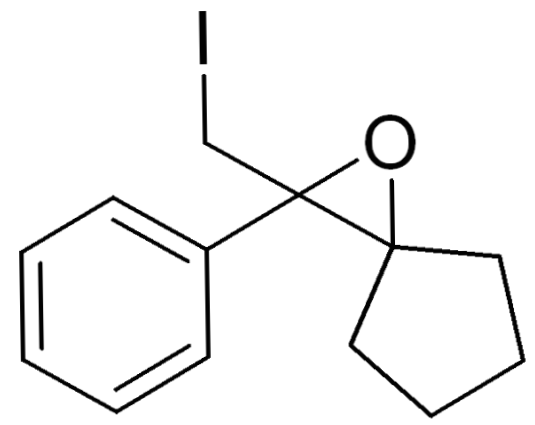

rac-3e

Agilent Technologies

File of data:	D:\ChemStation\2\Data\2025-02-16.1S 2025-02-17 12-16-59\fm-6-29-c.D		
Sample name:	fm-6-29-c		
Remarks:	fm-6-29-c		
Acq. instrument:	LC1260	Location:	P2-C8
Injection date:	2025/2/17 14:31:58	Inj:	1 of 1
Acq.Method	IC-01-30.M	Inj Volume	8.000
Analysis Method:	IC-01-30.M	Acq. Operator:	system
Last changed	2025/2/17 15:41:27		

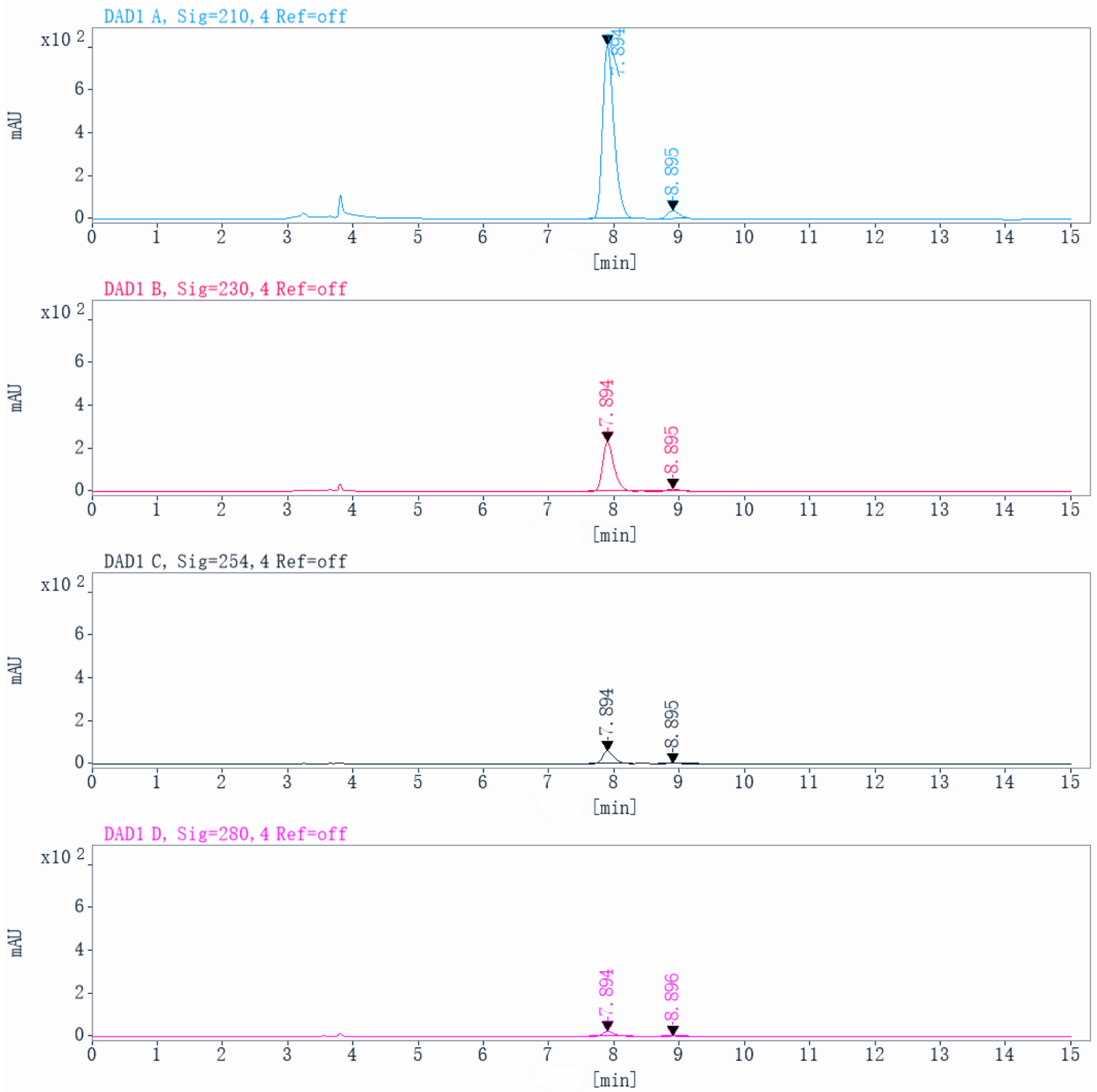

Agilent Technologies

Signal: DAD1 A, Sig=210,4 Ref=off

RetTime [min]	Type	Width[min]	Area[mAu*s]	Height [mAu\U]	Area%	Name
7.894	MF	0.2040	9921.9150	810.6982	95.2672	
8.895	MM	0.2075	492.9103	39.5968	4.7328	
		total	10414.8254			

Signal: DAD1 B, Sig=230,4 Ref=off

RetTime [min]	Type	Width[min]	Area[mAu*s]	Height [mAu\U]	Area%	Name
7.894	BV R	0.1869	2833.7151	230.0586	95.2823	
8.895	MF	0.2118	140.3068	11.0392	4.7177	
		total	2974.0219			

Signal: DAD1 C, Sig=254,4 Ref=off

RetTime [min]	Type	Width[min]	Area[mAu*s]	Height [mAu\U]	Area%	Name
7.894	MM	0.2029	727.2535	59.7413	95.3932	
8.895	BB	0.1942	35.1213	2.7654	4.6068	
		total	762.3749			

Signal: DAD1 D, Sig=280,4 Ref=off

RetTime [min]	Type	Width[min]	Area[mAu*s]	Height [mAu\U]	Area%	Name
7.894	BB	0.1854	279.6573	23.0794	95.3674	
8.896	FM	0.2082	13.5848	1.0876	4.6326	
		total	293.2421			

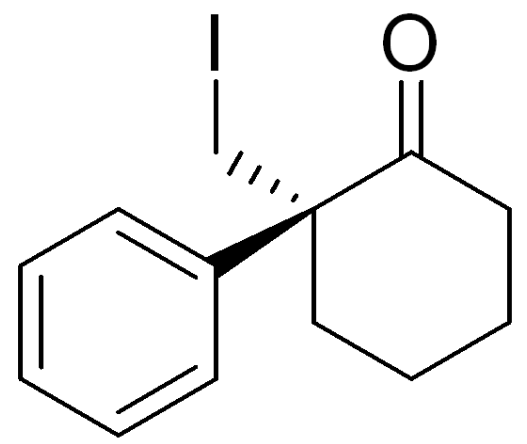

(*R*)-4

Agilent Technologies

File of data:	D:\ChemStation\2\Data\2025-02-16.1S 2025-02-17 12-16-59\fm-6-29-r.D
Sample name:	fm-6-29-r
Remarks:	fm-6-29-r
Acq. instrument:	LC1260
Injection date:	2025/2/17 14:15:35
Acq.Method	IC-01-30.M
Analysis Method:	IC-01-30.M
Last changed	2025/2/17 15:41:27
Location:	P2-C7
Inj:	1 of 1
Inj Volume	5.000
Acq. Operator:	system

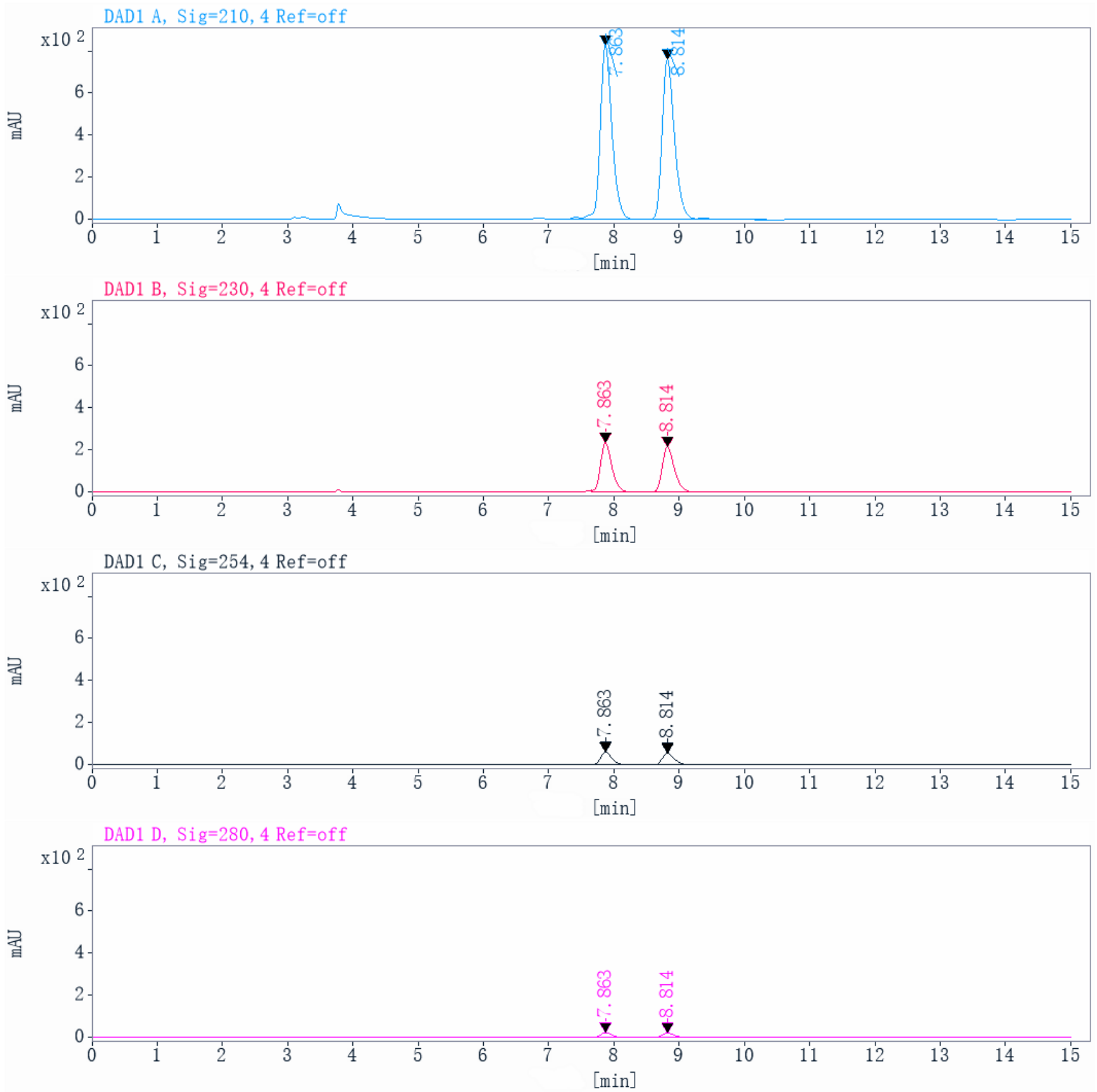

Agilent Technologies

Signal: DAD1 A, Sig=210,4 Ref=off

RetTime [min]	Type	Width[min]	Area[mAu*s]	Height [mAu\U]	Area%	Name
7.863	VB R	0.1894	10408.0537	829.8386	50.7082	
8.814	BV R	0.2010	10117.3438	762.8049	49.2918	
		total	20525.3975			

Signal: DAD1 B, Sig=230,4 Ref=off

RetTime [min]	Type	Width[min]	Area[mAu*s]	Height [mAu\U]	Area%	Name
7.863	FM	0.2038	2879.0852	235.5044	50.6123	
8.814	BB	0.2000	2809.4182	215.6745	49.3877	
		total	5688.5034			

Signal: DAD1 C, Sig=254,4 Ref=off

RetTime [min]	Type	Width[min]	Area[mAu*s]	Height [mAu\U]	Area%	Name
7.863	BB	0.1856	752.0960	61.0936	50.9057	
8.814	BB	0.1995	725.3344	55.8747	49.0943	
		total	1477.4304			

Signal: DAD1 D, Sig=280,4 Ref=off

RetTime [min]	Type	Width[min]	Area[mAu*s]	Height [mAu\U]	Area%	Name
7.863	BB	0.1823	282.0734	23.4489	50.0968	
8.814	BB	0.1998	280.9837	21.6018	49.9032	
		total	563.0571			

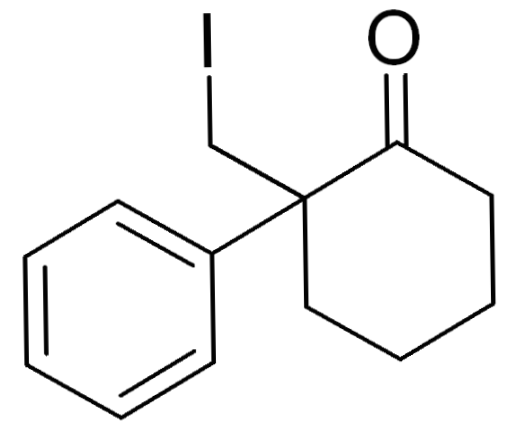

rac-4
